# Phenylalkyl Acetophenones and Anacardic Acids from Knema oblongifolia with Synthetic Analogues as Anti-infectives and Antibacterial Agents

**DOI:** 10.1021/acsbiomedchemau.5c00052

**Published:** 2025-06-09

**Authors:** Olivier Auguste Kirchhoffer, Jahn Nitschke, Alexandre Luscher, Louis-Félix Nothias, Laurence Marcourt, Nabil Hanna, Antonio Grondin, Thilo Köhler, Emerson Ferreira Queiroz, Thierry Soldati, Jean-Luc Wolfender

**Affiliations:** † Institute of Pharmaceutical Sciences of Western Switzerland, 27212 University of Geneva, CMU, Geneva 1211, Switzerland; ‡ School of Pharmaceutical Sciences, University of Geneva, CMU, Geneva 1211, Switzerland; § Department of Biochemistry, Faculty of Sciences, University of Geneva, Quai Ernest-Ansermet 30, Geneva 1205, Switzerland; ∥ Department of Microbiology and Molecular Medicine, University of Geneva, CMU, Geneva 1211, Switzerland; ⊥ Université Côte d’Azur, CNRS, ICN, Nice 06103, France; # Green Mission Department, 113971 Herbal Products Laboratory, Pierre Fabre Research Institute, Toulouse 31100, France

**Keywords:** antimicrobial resistance, natural products, plant extract library, anti-infective, 3R-infection model, HPLC bioactivity profiling, hemisynthesis

## Abstract

The present study investigates the potential anti-infective and antibacterial properties of phenylalkyl acetophenones and anacardic acids isolated from the ethyl acetate extract of the leaves of Knema oblongifolia, along with synthetic derivatives that were generated. As antibiotic resistance grows, the discovery of new anti-infective agents becomes crucial. The study utilizes a phenotypic screening approach, employing a 3R infection model with *Mycobacterium marinum* (Mm) and *Dictyostelium discoideum* (Dd) as proxies for *Mycobacterium tuberculosis* and human macrophages. This model helps to distinguish between general antibiotics and specific anti-infectives that inhibit bacterial growth inside host cells. A previous screening carried out on a collection of 1600 plant natural extracts revealed *K. oblongifolia* as a significant source of anti-infective compounds. The ethyl acetate extract of this plant exhibited a strong inhibition of Mm intracellular growth in the infection model while minimally affecting bacterial growth in broth. HPLC bioactivity profiling of this extract based on a high-resolution microfractionation strategy uncovered that the activity was associated with different LC-peaks spread over the chromatogram. LC–MS-based metabolite profiling of the extract revealed that they shared common substructural elements. Based on such information, fractionation of the extract at a larger scale led to the isolation of 12 bioactive natural products (NPs): four newly described acetophenone NPs and eight salicylic acid derivatives (three of which were new). These NPs were further tested for their activities against Mm (antibacterial and anti-infective), *Pseudomonas aeruginosa*, and *Staphylococcus aureus*. Additionally, the study involved de novo synthesis of derivatives based on the backbones of the isolated acetophenones to enhance their bioactivity. Hemisynthesis on one of the isolated natural acetophenone was also carried out and resulted in an increase in potency but no increase in selectivity toward the inhibition of Mm intracellular growth. Overall, biological activity assessments revealed that some of the synthetic analogues generated were better candidates in terms of both selectivity and potency, with an improved activity profile compared to natural analogues. The best synthetic candidate reached an IC_50_ of 0.59 μM for the inhibition of intracellular bacterial growth during infection (anti-infective activity).

## Graphical abstract

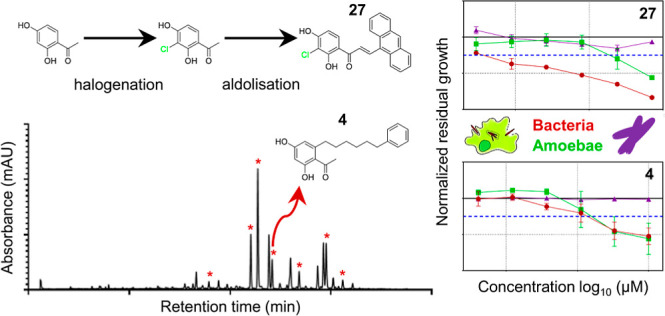

## Introduction

Antibiotics have had a paradigm-changing impact on medicine since their discovery in the middle of the 20th century, saving innumerable human lives since their introduction into commercial use.^1^ Yet, the efficacy of those “magic bullets” has been gradually eroded due to overconsumption and injudicious use in clinical contexts. While their effects have largely been taken for granted by the public, this hides a looming threat in the emergence of multidrug-resistant (MDR) and extremely drug-resistant (XDR) bacterial pathogens. It was estimated that by 2050, about 10 million people each year would be dying from antibiotic-resistant infections, which would make it the largest cause of death globally.^2,3^ One bacterial pathogen that is particularly worrying for the future of antibiotic treatments is *Mycobacterium tuberculosis* (Mtb), with an estimated death toll of 1.3 million people as of 2022.^4^

Historically natural products (NPs) have been a rich source of chemical diversity, with about 50% of all drugs approved in the past four decades having been derived from nature.^5^ Several methods, mostly phenotypic-based, have been established to assess antimicrobial activities of NPs, notably disk well diffusion, agar dilution, or broth dilution, to name a few.^6^ The era of target-based drug discovery, which started in the 1990s, has generally failed to deliver significant progress in the discovery of antimicrobials. Phenotypic screening approaches have therefore returned to fashion recently, especially as they increasingly enable the discovery of new targets.^7–9^ This renewed attention toward phenotypic screening was enabled by the development of more complex and specific phenotypic models, notably models which capture the intracellular nature of Mtb’s pathogenicity.^10,11^ In the current study, a 3R-infection model that mimics the behavior of Mtb when it infects human macrophages was established, using *Mycobacterium marinum* (Mm, ATCC BAA-535) and *Dictyostelium discoideum* (Dd, ATCC MYA-4120), respectively, as stand-ins for Mtb and macrophages.^12–14^ The goal of this assay is to find “anti-infective” samples that would affect only the growth of the bacteria in the infection model and would therefore be less prone to resistance mechanisms. To distinguish between anti-infectives and antibiotics, a second assay where Mm was cultured in broth was carried out.^14^

This approach also provided a double readout on the growth of both the bacteria and the amoeba, making it possible to distinguish between “strict anti-infectives” (only inhibiting bacterial growth in infection) and “anti-infective Dd inhibitors” (inhibiting the growth of both bacteria and amoeba).^14^

In addition to stringent biological assays, it is also crucial to have access to a large source of chemical diversity to tackle the challenges of antibiotics resistance. For this, an internal collection of 1600 registered Natural plant Extracts (NEs) previously described, which contains species that encompass about 30% of all known botanical families,^15^ was screened for anti-infective activities with the Dd-Mm anti-infective assay.^14,16^ Based on that screening, this work focused on the phytochemical investigation of the ethyl acetate extract of *Knema oblongifolia* (King) Warb., Myristicaceae (leaves), which was highlighted as a strong anti-infective. The aim was then to identify its active principles, obtain a series of structurally related natural and synthetic derivatives, characterize their activities, and determine structural features possibly responsible for the observed activity.

The investigation included high-resolution bioactivity profiling to target bioactive compounds, large-scale isolation of anti-infective entities, and synthetic derivatization of the backbone of some of the identified NPs. In addition to the anti-infective potency assessment of the isolated NPs, antibacterial assays on one strain of *Staphylococcus aureus* (Sa, Gram-positive)^17^ and one strain of *Pseudomonas aeruginosa* (Pa, Gram-negative)^18,19^ were also carried out to assess general antibacterial activities and contextualize them with the antimycobacterial activities.

## Results and Discussion

### Screening of 1600 Plant Extracts for Anti-infective Activities

The biological screening^14,16^ of a 1600 NE collection previously described^15^ initiated this study. For this, a model based on the infection of the amoeba host Dd by Mm was used.^14,20^ Two readouts were obtained from this anti-infective assay: “anti-infectives” were the samples that reduced the growth of Mm by at least 50% during infection, while “Dd inhibitors” were those that reduced the growth of Dd by at least 50%. A second assay to assess the antibiotic activity of samples was also carried out with Mm grown in broth (without any host). This approach allowed for the discrimination of antibiotics from anti-infectives, with the latter acting predominantly during infection. NEs were therefore considered hits if they were anti-infectives but not antibiotic. Extracts classified as “anti-infective Dd inhibitors” were also considered hits, despite the ideal scenario being that of “strict anti-infectives”. Indeed, components responsible for the anti-infective effect in an extract might not necessarily be the same as those responsible for inhibiting the growth of the amoeba.

Among the hit extracts that this screening yielded, the ethyl acetate extract of *K. oblongifolia* leaves (King) Warb., Myristicaceae (Q5438126) was one of the anti-infective Dd inhibitors with a striking anti-infective activity but also affecting the growth of the amoeba. To our knowledge, no phytochemical investigations were reported for this species at the time of writing (see WikiData query), it was therefore chosen as the focus of the current study.

### Biological Activities of the Extract of *K. oblongifolia* (Leaves)

The ethyl acetate extract of *K. oblongifolia* leaves strikingly inhibited (by 155%) the intracellular growth of Mm during infection compared to the DMSO control (Figure 1C,D) while leaving the bacteria almost unaffected in broth (Figure 1A,B). Note that a growth inhibition of over 100% effectively corresponds to a reduction of the initial Mm load present inside Dd at the start of the infection, which means that the extract is bactericidal. The extract also decreased the cell mass of the amoeba compared to the initial value, which indicates cytotoxicity (Figure 1E,F). Despite the latter “red flag”, the extract was nevertheless considered for further study because it exerted selective anti-infective activity without activity against the bacteria in broth. The hope was also for the anti-infective and the cytotoxic activity to be disentangled during fractionation.

**Figure 1:**
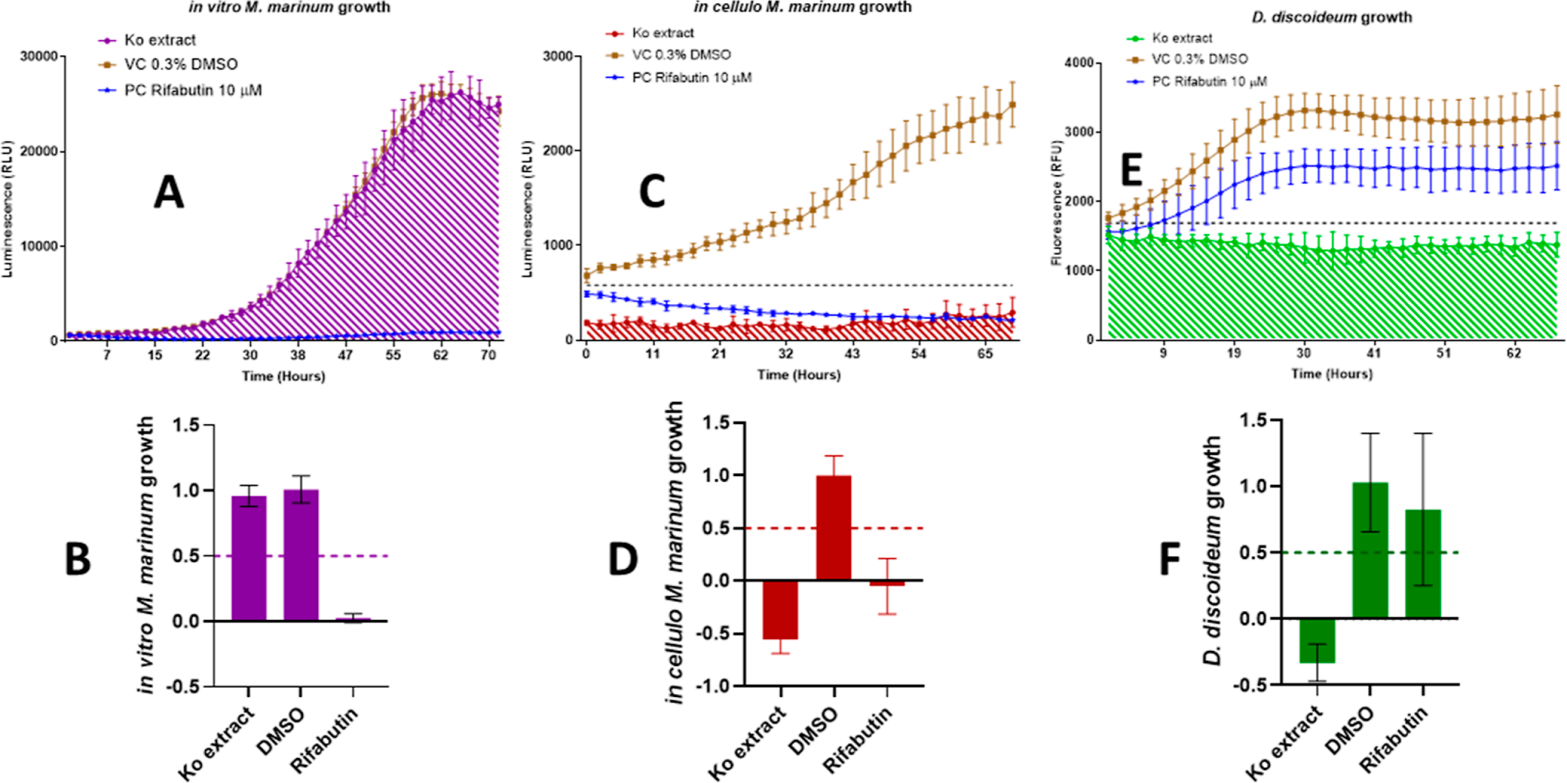
Biological activities of the ethyl acetate extract of *K. oblongifolia* leaves (Ko extract) (A) *in vitro* Mm growth curves; (B) normalized *in vitro* Mm growth, inhibited by 4% with the extract; (C) *in cellulo* Mm growth curves; (D) normalized *in cellulo* Mm growth, inhibited by 155% with the extract; (E) *in cellulo* Dd growth curves; (F) normalized *in cellulo* Dd growth, inhibited by 133% with the extract; vehicle control (VC): DMSO 0.3%; positive control (PC): rifabutin (10 μM); and extract tested at 25 μg/mL. 50% growth inhibition is the threshold (represented by a dotted line in B, D, and F) set for a NE to be considered bioactive in each respective category. The dotted lines in panels A, C, and E represent the median of luminescence/fluorescence of all wells at time 0. Each experiment was carried out in 3 biological replicates, 1 technical replicate.

### Phytochemical Investigations on*K. oblongifolia* (Leaves)

In order to disentangle the activities of the various NPs in the extract, an HPLC bioactivity profiling method^21^ was used to monitor the activity on the Dd-Mm model. For this, the extract was subjected to generic metabolite profiling by UHPLC-PDA-CAD-HRMS/MS. The UV-PDA trace revealed that the extract was rich in UV-active NPs. The Charged Aerosol Detection (CAD) trace, which is known to provide a semiquantitative readout, revealed that it fitted well with the UV trace at 329 nm. Indeed, for almost all CAD-detected peaks, a proportional UV peak was detected, UV detection was therefore well adapted to study this plant extract (Supporting Information Figure 1).

Dereplication based on tandem MS spectra (MS^2^) recorded by data-dependent analysis using the computational MS framework software SIRIUS^22^ was largely inconclusive.

The HPLC bioactivity profiling was done by a microfractionation into a 96-well plate in a single chromatographic run on an aliquot of 13 mg. Individual microfractions were dried, all diluted in a conserved volume, and tested for anti-infective activities. Following the color patterns established in Figure 1, individual values for bacterial growth in infection for each microfraction are displayed in red on the chromatogram, the growth of the amoeba in green, and the bacterial growth in broth in purple (Figure 2).

**Figure 2:**
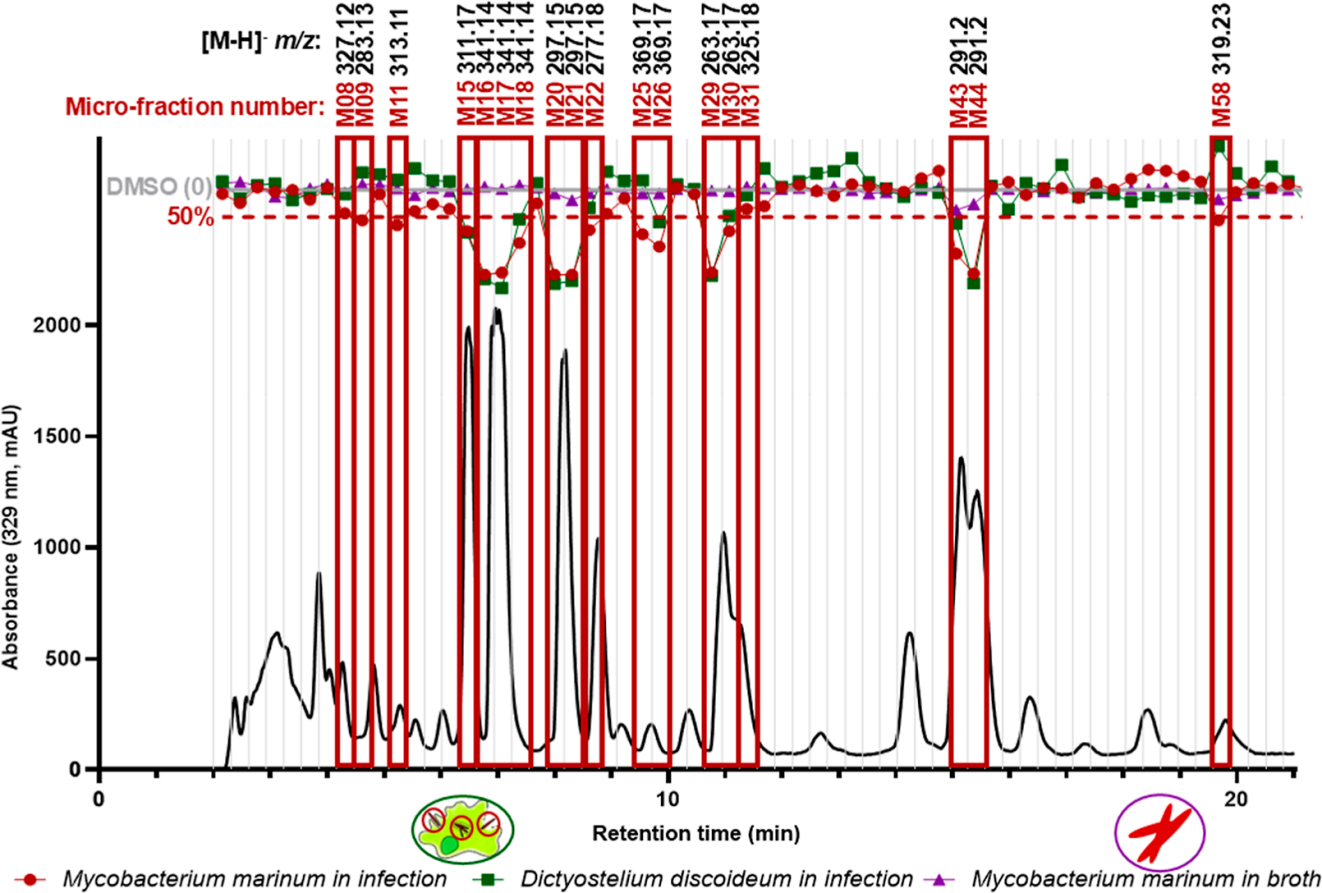
Semipreparative HPLC-PDA microfractionation of the ethyl acetate extract of *K. oblongifolia* leaves (30 min chromatographic). Vertical lines correspond to the 92 microfractions that were tested for their anti-infective activities in infection (in red), their effect on the host in infection (in green) as well as antibiotic activities *in broth* (in purple) with 3 biological replicates, and 1 technical replicate for each experiment. Ten zones (hit microfractions) with distinct *m*/*z* values detected and with inhibitory activities above 50% can be observed. Two more microfractions with distinct *m*/*z* values and inhibitory activities close to 50% (but not above) were also considered hits (M08 and M31). The 30 min chromatogram was truncated between 0 and 21 min to focus on bioactive regions.

A total of 16 microfractions (**M**) among the 92 collected from the EtOAc extract of *K. oblongifolia* displayed over 50% inhibition of intracellular bacterial growth and were flagged as anti-infective. Subsequent UHPLC-PDA-CAD-HRMS/MS analyses of these microfractions and adjacent ones revealed 12 distinct target [M-H]^−^ MS features (*m*/*z* @ RT), two of which came from adjacent microfractions that were close to passing the 50% bioassay cutoff (**M08** and **M31** with 57% and 65% inhibition of intracellular bacterial growth, respectively). These two microfractions were considered due to their low toxicity toward the amoeba (in red, Figure 3), since it appeared that many of the active microfractions were also affecting the growth of the amoeba.

**Figure 3:**
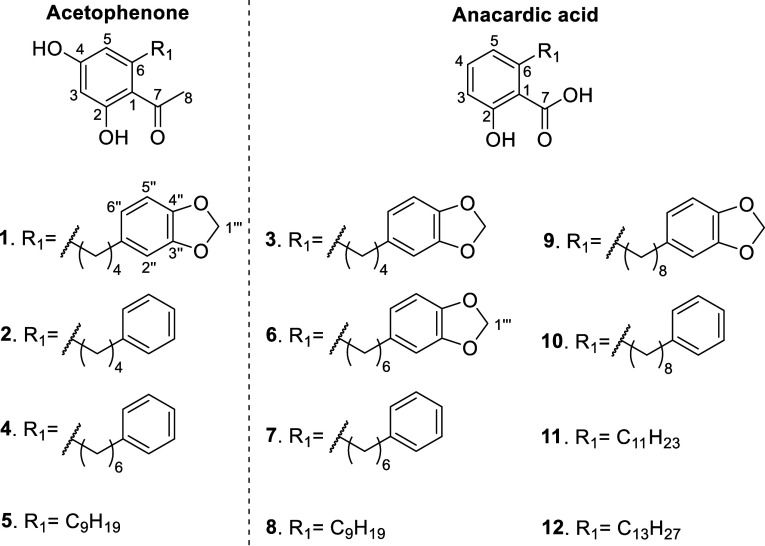
Compounds isolated from *K. oblongifolia* (leaves).

Dereplication based on tandem MS spectra (MS^2^) recorded by data-dependent analysis of the 12 bioactive microfractions using SIRIUS (in both PI and NI modes) was largely inconclusive.^22^ The annotations were generally of low confidence according to their associated quality scores (by CSI/FingerID^23^), and the proposed structures did not correspond to those expected based on phytochemical reports on the genus (*Knema*) level. Nevertheless, molecular formulas (MF) could be established with confidence at the MS1 level based on HRMS data in both positive- (PI) and negative-ion (NI) mode (see Table 1). Three of them could be matched with NPs reported in the literature of the genus *Knema*. **M25**-**26** corresponded to kneglobularic acid B, previously isolated from *Knema globularia*, along with kneglobularic acid A which corresponded to **M30**-**31**.^24^ Khookerianic acid A was matching the molecular formula found in **M29**-**30** and was reported in *Knema hookeriana*.^25^ All of them were derived from a common salicylic acid scaffold and belong to the family of anacardic acids.

**Table 1:** Bioactive Microfractions with Their Major Ion *m/z* and Characteristic Fragment Flagged in MS/MS Analyses^a^

microfraction number	*m*/*z* NI	*m*/*z* PI	molecular formula	MS^2^ fragment	compound *n*°
**M08**	327.1237 [M – H]^−^	329.1382 [M + H]^+^	C_19_H_20_O_5_	149.0598 [C_9_H_9_O_2_^+^]	**1**
**M09**	283.1336 [M – H]^−^	285.1483 [M + H]^+^	C_18_H_20_O_3_	149.0598 [C_9_H_9_O_2_^+^]	**2**
**M11**	313.1079 [M – H]^−^	297.1482 [M-H_2_O + H]^+^	C_18_H_18_O_5_	107.0492 [C_7_H_7_O^+^]	**3**
**M15**	311.1651 [M – H]^−^	313.1796 [M + H]^+^	C_20_H_24_O_3_	149.0598 [C_9_H_9_O_2_^+^]	**4**
**M16–18**	341.1390 [M – H]^−^	325.1430 [M-H_2_O + H]^+^	C_20_H_22_O_5_	107.0491 [C_7_H_7_O^+^]	**6**
**M20–21**	297.1495 [M – H]^−^	281.1530 [M-H_2_O + H]^+^	C_19_H_22_O_3_	107.0491 [C_7_H_7_O^+^]	**7**
**M22**	277.1809 [M – H]^−^	279.1949 [M + H]^+^	C_17_H_26_O_3_	149.0598 [C_9_H_9_O_2_^+^]	**5**
**M25–26**	369.1703 [M – H]^−^	353.1744 [M-H_2_O + H]^+^	C_22_H_26_O_5_	107.0490 [C_7_H_7_O^+^]	**9**
**M29–30**	263.1651 [M – H]^−^	265.1794 [M + H]^+^	C_16_H_24_O_3_	107.0491 [C_7_H_7_O^+^]	**8**
**M30–31**	325.1806 [M – H]^−^	309.1845 [M-H_2_O + H]^+^	C_21_H_26_O_3_	107.0491 [C_7_H_7_O^+^]	**10**
**M43–44**	291.1964 [M – H]^−^	293.2106 [M + H]^+^	C_18_H_28_O_3_	107.0491 [C_7_H_7_O^+^]	**11**
**M58**	319.2276 [M – H]^−^	303.2319 [M-H_2_O + H]^+^	C_20_H_32_O_3_	107.0491 [C_7_H_7_O^+^]	**12**

a For each microfraction of interest: *m/z* in positive (PI) and negative ion mode (NI), molecular formulae, and characteristic fragment found in the MS^2^ spectrum and the associated compound number corresponding to eventually purified compound.

Manual inspection of MS^2^ spectra then revealed some fragmentation patterns that appeared across the different fractions. Two fragment ions were found repeatedly and in a mutually exclusive manner in the different fractions targeted (Table 1), one with an *m*/*z* of 149.0597 (C_9_H_9_O^+^) and another with an *m*/*z* of 107.0491 (C_7_H_7_O^+^). This observation suggested that the main compounds highlighted as being bioactive exhibited repeated structural patterns. The C_7_H_7_O^+^ fragment is in fact a plausible fragment of anacardic acids and was found in all three sets of microfractions (**M25**-**26**, **M29**-**30**, and **M30**-**31**) annotated as anacardic acid derivatives (Table 1).

Several bioactive HPLC peaks could not be fully dereplicated at this level, and several MF were not previously reported in the *Knema* genus. To establish biological activity for individual compounds and unambiguously determine their structure, targeted isolations were carried out to purify all of the NPs of interest listed in Table 1.

### Isolation of Targeted Bioactive NPs

For complete de novo structural identification and bioactivity characterization, isolation of compounds corresponding to MS^2^ spectra highlighted by HPLC bioactivity profiling was necessary. This was done at a larger scale using flash-UV chromatography (0.5 g of extract used against 13.05 mg used for the HPLC bioactivity profiling process). First, the standard conditions were optimized at the analytical scale using an HPLC before being geometrically transferred^26^ to the flash-UV chromatography scale. Using this approach, 12 NPs were isolated in amounts ranging from 2.4 to 38 mg (Figure 3).

Full de novo structure identifications of the isolated compounds revealed that they were acetophenone and anacardic acid derivatives. A total of five structures among those in Figure 3 were known (**8**–**12**). As expected from previous dereplications, khookerianic acid A (**8**, **M29**-**30**) and khookerianic acid C (**10**, **M30**-**31**) were previously isolated from *K. hookeriana* (Q5457252).^25^ Kneglobularic acid B (**9**, **M25**-**26**) and kneglobularic acid A (other name for **10**) were previously isolated from *K. globularia* (Q5399660).^24^ Anagigantic acid (**11**) was first isolated from the pericarp of *Anacardium giganteum* (Q9673175)^27^ and 6-tridecylsalicylic acid (**12**) was isolated from the brown algae *Caulocystis cephalornithos* (Q29146806).^28^

Additionally, seven new structures were identified and were named knemolone A–D (**1–2**, **4**–**5**) and knemolic acid A–C (**3**, **6**–**7**) based on characteristic 2D-NMR correlations (Figure 4).

**Figure 4:**
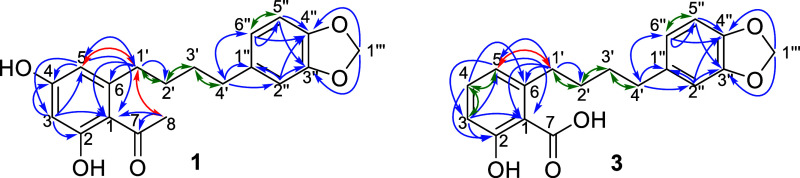
Representative 2D NMR interactions for acetophenone (1) and salicylic acid scaffolds (3). Blue: HMBC correlations, green: COSY correlations, and red: ROESY correlations.

Knemolone A–D (**1–2**, **4–5**) were isolated as yellow oils with [M-H]^−^ of *m*/*z* 327.1237/283.1337/311.1651/277.1808 corresponding to MF of C_19_H_20_O_5_ (error −0.31 ppm)/C_18_H_20_O_3_ (error −1.06 ppm)/C_20_H_24_O_3_ (error −0.64 ppm)/C_17_H_26_O_3_ (error −0.36 ppm), respectively. The ^1^H and ^13^C NMR data (Table 2 and Supporting Information S1.3./S2.3./S4.3./S5.3. and S1.4./S2.4./S4.4./S5.4.) displayed evidence of the resacetophenone moiety in these four compounds being reminiscent of that described in khookerianone A.^25^ Similarly, the alkyl chain connected to the aromatic ring at C-6 with correlations described in that report was also observed in the data of all the new knemolones.

**Table 2:** ^1^H and ^13^C NMR Data of Knemolones A–D 1–2 and 4–5

position	1		2		4		5	
	^1^ *H*	^13^ *C*	^1^ *H*	^13^ *C*	^1^ *H*	^13^ *C*	^1^ *H*	^13^ *C*
1		115.5		115.6		115.5		115.5
2		166.1		166.1		166.1		166.1
3	6.24 d (2.6)	101.9	6.24 d (2.4)	100.9	6.24 q (2.6)	101.8	6.24 d (2.6)	101.8
4		160.9		160.8		161.0		160.9
5	6.22 d (2.5)	110.6	6.21 d (2.5)	110.6	6.24 q (2.6)	110.7	6.26 d (2.6)	110.7
6		147.5		147.5		147.9		148.0
7		204.2		204.3		204.4		204.4
8	2.60 s	32.3	2.59 s	32.3	2.62 s	32.3	2.64 s	32.3
1′	2.85 t (7.5)	36.3	2.86 t (7.5)	36.3	2.82 t (8.0)	36.4	2.83 t (8.1)	36.5
2′	1.64 m	31.7	1.64 m	31.7	1.55–1.67 m	32.3	1.59 m	32.4
3′	1.64 m	31.6	1.71 m	31.5	1.38 m	29.7	1.36 m	29.9
4′	2.56 t (7.1)	35.5	2.64 t (7.5)	35.8	1.38 m	29.1	1.22–1.30 m	29.6
5′					1.55–1.67 m	31.5	1.22–1.30 m	29.6
6′					2.60 t (7.7)	36.0	1.22–1.30 m	29.4
7′							1.22–1.30 m	32.0
8′							1.22–1.30 m	22.8
9′							0.88 t (6.9)	14.2
1″		136.0		142.1		142.7		
2″	6.65 s	108.3	7.28 t (7.5)	128.5	7.28 t (7.5)	128.4		
3′’		147.7	7.14–7.22 m	128.5	7.14–7.21 m	128.5		
4″		145.8	7.14–7.22 m	126.0	7.14–7.21 m	125.8		
5′’	6.72 d (7.8)	108.9	7.14–7.22 m	128.5	7.14–7.21 m	128.5		
6″	6.60 d (7.8)	121.2	7.28 t (7.5)	128.5	7.28 t (7.5)	128.4		
1‴	5.92 s	100.9						

Differences appeared in the lengths of the alkyl chains and its termination. Knemolone A (**1**), B (**2**), and C (**4**) all showed evidence of the other end of the alkyl chain being connected to another aromatic ring at the C-1a position (δ_*C*_ 136.0/142.1/142.7), with HMBC correlations between H-4′ (δ_*H*_ 2.56/2.64) and H-6′ (δ_*H*_ 2.60) and C-2″ (δ_*C*_ 108.3/128.5/128.4) and C-6″ (δ_*C*_ 121.2/128.5/128.4). This aromatic ring appeared to be trisubstituted in **1** as opposed to monosubstituted and terminal in **2** and **4** (see δ_*H*_ in Table 2). Compound **1** was substituted at C-3′’ and C-4″ with a dioxolane cycle, evidenced by the HMBC correlations between the dioxolane OCH_2_O (C-1‴, δ_*H*_ 5.92) and C-3″ (δ_*C*_ 147.7) and C-4″ (δ_*C*_ 145.8). Knemolone D (**5**), on the other hand, had no second aromatic ring, instead having a terminal methyl group at the end of a longer chain of 9 carbons at C-9′ (δ_*H*_ 0.88) correlating with C-8′ (δ_*C*_ 22.8) and C-7′ (δ_*C*_ 32.0).

Knemolic acids A–C (**3**, **6–7**) were isolated as light green amorphous powders with [M-H]^−^ of *m*/*z* 313.1079/341.1391/297.1495 corresponding to MF of C_18_H_18_O_5_ (error −0.64 ppm)/C_20_H_22_O_5_ (error −0.88 ppm)/C_19_H_22_O_3_ (error −0.34 ppm), respectively. The ^1^H and ^13^C NMR data (Table 3 and Supporting Information S3.2./S6.3./S7.3. and S3.3./S6.4./S7.4.) for knemolic acid A was in fact similar to that of knemolone A (**1**), except for positions 1–7. Instead of a tetrasubstituted resacetophenone scaffold, compound **3** showed evidence of a trisubstituted anacardic acid scaffold. C-7 (δ_*C*_ 174.0) has a chemical shift in line with that of a carboxylic acid, which is much lower than that of the previously observed ketone. This carboxylic acid group was positioned on C-1 (δ_*C*_ 110.5) as it was the last available point of substitution of the aromatic ring not yet assigned. The chemical shift of C-1 was also in agreement with the presence of an acid group and a neighboring hydroxyl group on C-2 (δ_*C*_ 163.8). Chemical shifts on that aromatic ring were also in line with those reported for khookerianic acid C.^25^

**Table 3:** ^1^H and ^13^C NMR Data of Knemolic Acids A–C 3 and 6–7

position	3		6		7	
	^1^ *H*	^13^ *C*	^1^ *H*	^13^ *C*	^1^ *H*	^13^ *C*
1		110.5		110.5		110.5
2		163.8		163.8		163.8
3	6.86 dd (8.4, 1.3)	116.1	6.87 dd (8.3, 1.2)	116.0	6.87 dd (8.3, 1.2)	116.0
4	7.34 t (7.9)	135.3	7.36 t (7.9)	135.6	7.36 t (7.5)	135.5
5	6.74 dd (7.5, 1.3)	122.8	6.76 dd (7.5, 1.2)	122.9	6.76 dd (7.5, 1.3)	122.9
6		147.1		147.8		147.8
7		174.0		175.9		175.8
1′	2.98 t (7.3)	36.5	2.97 t (8.0)	36.6	2.97 t (8.0)	36.6
2′	1.63 m	31.6	1.59 m	32.0	1.62 m	32.1
3′	1.63 m	31.9	1.37 m	29.1	1.40 m	29.8
4′	2.55 t (7.0)	35.6	1.37 m	29.7	1.40 m	29.2
5′			1.59 m	31.8	1.62 m	31.6
6′			2.52 t (7.7)	35.8		36.1
1″		136.5		136.8		143.0
2″	6.67 d (1.8)	108.2	6.67 d (1.8)	109.0	7.27 dd (8.3, 7.0)	128.4
3′’		147.6		147.6	7.15–7.20 m	128.6
4″		145.6		145.5	7.15–7.20 m	125.7
5″	6.71 d (7.8)	109.0	6.71 d (6.71)	108.2	7.15–7.20 m	128.6
6″	6.61 dd (7.9, 1.8)	121.2	6.61 dd (7.9, 1.7)	121.2	7.27 dd (8.3, 7.0)	128.4
1‴	5.91 s	100.9	5.90 s	100.8		

In the end, 4 new acetophenone derivatives (**1**–**2**, **4**–**5**) and 8 salicylic acid derivatives (**3**, **6**–**12**), of which 3 were newly described NPs (**3**, **6**, **7**), were isolated. Once the structures were elucidated, it became clear that the C_9_H_9_O^+^ fragment (Table 1) was characteristic to all the acetophenone derivatives, while the C_7_H_7_O^+^ fragment was characteristic to all salicylic acid derivatives.

The purity of each identified NP was assessed using an NMR-based process (described in Supporting Information Figure 2), which revealed varying levels of impurities for the 12 isolated NPs (Supporting Information Table 1). The following part on biological results was refined to focus on compounds that were considered pure (at least 90% purity according to the described metric, leaving compounds **7** and **8**, Table 4), but all 12 samples containing the NPs have been tested, and the full data with associated purity scores can be found in Supporting Information Table 2. Additionally, a commercial standard of similar chemical structure (anacardic acid) was also tested to confirm that the anti-infective activities observed were indeed associated with the studied chemical structures. For all compounds, the level of impurities did not preclude their unambiguous structural identification (by NMR and HRMS).

**Table 4:** Biological Activities of NPs Isolated from *K. oblongifolia* Ethyl Acetate Leaf Extract^a^

compound	IC_50_ on Mm in infection (μM)	IC_50_ on Dd in infection (μM)	IC_50_ on Mm *in broth* (μM)	MIC on Sa (mg·L–1)
**7**	7.41	7.41	not active	8
**8**	2.47	2.47	not active	8
**anacardic acid**	22.22	not active	not active	16
**rifabutin**	0.2	not active	0.03	not tested
**vancomycin**	not tested	not tested	not tested	1

a Anacardic was added as a standard of the same chemical class as some isolated NPs, rifabutin as the positive control for antimycobacterial activity against Mm, and vancomycin for antibacterial activities against *Staphylococcus aureus* (Sa).

### Bioactivities of Isolated NPs

Most of the isolated compounds followed a similar pattern of activity when it came to their effects on Mm. They were broadly inactive against the bacterium alone (“in broth”), while in the infection model, most of them affected both Mm and the host Dd in a similar manner (Table 4). This followed the initial pattern of activity observed at the extract level, with no separation of the anti-infective effect from the unwanted inhibition of the amoeba growth. Salicylic acid derivatives were generally more active than the acetophenones on bacterial growth in infection.

All 12 compounds were also tested against Gram-negative *P. aeruginosa* (Pa) and Gram-positive *S. aureus* (Sa). While none of them displayed specific activity against Pa, most of them showed some activity when tested against Sa with minimum inhibitory concentrations (MICs) reaching as low as 8 μg.mL^–1^ (Table 4). Readouts on those two additional bacteria seemed to suggest that Mm in broth behaved more closely toward Pa than toward Sa in the case of these isolated NPs.

Anacardic acid was already reported as active against Sa^29^ at a dose of 25 μg mL^–1^, which is broadly in line with the experimental MIC value observed at 16 μg mL^–1^ (Table 4).

In the end, it was decided that the acetophenone scaffold was worth investigating as there was more chemical novelty in NPs isolated in this category, while more extensive reports exist on the biological activities of salicylic acid derivatives/anacardic acids.^25,29,30^

### Synthetic Derivatization of a Bioactive NP Scaffold

Synthetic derivatization was pursued using the acetophenone scaffold, which offered a balance between moderate anti-infective properties and good antibacterial activity against Sa for certain derivatives. We opted to introduce halogen atoms (chlorine and bromine in particular) into this scaffold, as such substitutions can boost biological activities.^31–33^ Halogen bonding is the typical type of interactions halogens can build with a target, where they behave as Lewis acids (driven by a positively charged region known as a “σ-hole” on the halogen) and can interact with Lewis bases.^34^ For these derivatizations, cheaply available and abundant 2,4-dihydroxyacetophenone (**13**, Figure 5) was used as the starting material.

**Figure 5:**
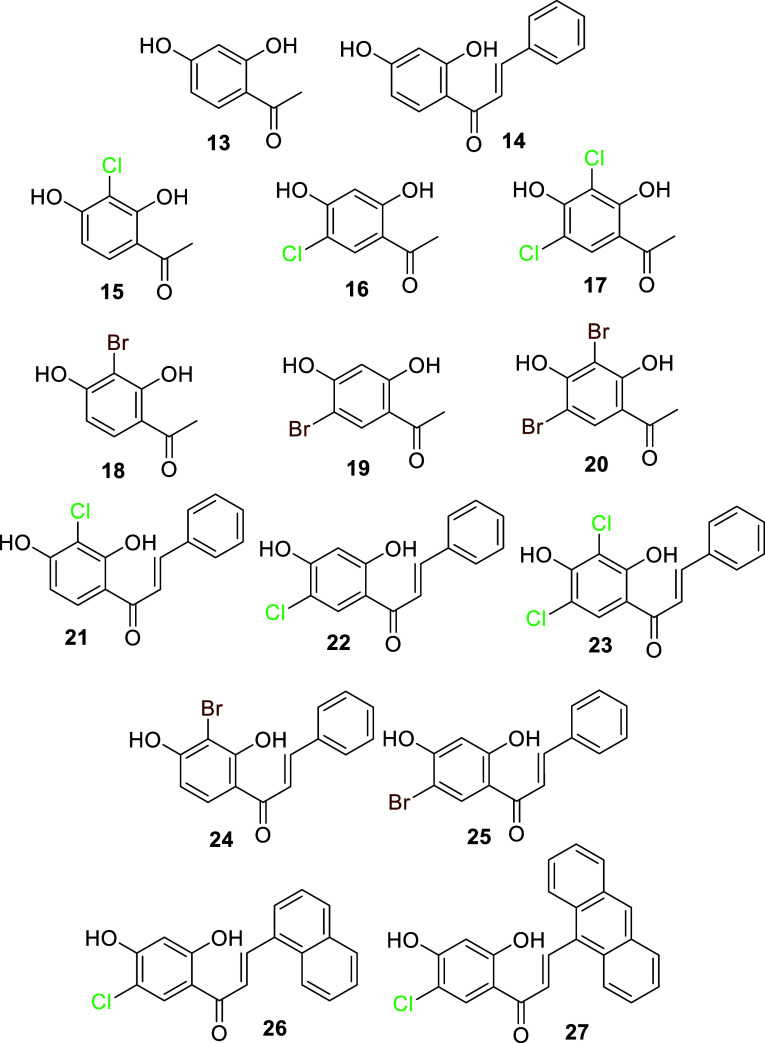
Compounds synthesized from derivatization of the 2,4-dihydroxyacetophenone scaffold.

The first step of halogenation reaction^35^ was chosen to alter the electronegativity of the 2,4-dihydroxyacetophenone cycle. Both chlorine and bromine could be added in a nonselective manner through an Electrophilic Aromatic Substitution (EAS) mechanism on positions 3- and/or 5- of the starting material **13**. Bioactivity measurements on the halogenated acetophenone derivatives suggested that chlorination (**15**–**17**) increased the potency of compounds on both anti-infective and Dd-inhibition (Table 5). Bromination (**18**–**20**) did not seem to affect activity readouts in a significant manner.

**Table 5:** Biological Activities of Acetophenone-Based Synthetic Derivatives, with Rifabutin and Vancomycin as Positive Controls for Antimycobacterial and Antibacterial Activities, Respectively

compound	IC_50_ on Mm in infection (μM)	IC_50_ on Dd in infection (μM)	IC_50_ on Mm *in broth* (μM)	MIC on Sa (mg·L^–1^)
**13**	66.67	not active	not active	not active
**14**	2.47	7.41	66.67	32
**15**	22.22	22.22	not active	not active
**16**	22.22	22.22	not active	not active
**17**	66.67	66.67	not active	not active
**18**	66.67	66.67	not active	not active
**19**	66.67	22.22	not active	not active
**20**	66.67	66.67	not active	not active
**21**	1.76	0.59	47.60	32–64
**22**	1.76	1.76	47.60	32
**23**	1.76	1.76	47.60	4
**24**	2.47	2.47	not active	64
**25**	0.82	0.82	66.67	not active
**26**	0.59	1.47	47.60	not active
**27**	0.59	15.87	not active	2
**rifabutin**	0.2	not active	0.03	not tested
**vancomycin**	not tested	not tested	not tested	1

In the second step, an aldolization reaction was carried out on the ketone moiety to introduce a phenyl-containing side chain in analogy to compounds **2** and **4**. It seemed at first that this addition even on nonhalogenated starting material **13** generated toxicity toward the amoeba, with compound **14** having a similar effect on both the amoeba and the bacteria. This was also reflected in all halogenated derivatives that have undergone aldolization (**15**–**20** vs **21**–**27**), where the absolute anti-infective activity had increased but so did the activity on the growth of the amoeba. Eventually, the 5-chloro-chalcone scaffold (**22**) was chosen for further derivatization based mainly on higher yields during the chlorination reaction (39.3%) compared to the dichlorinated analogue (**23**, 3.54%).

Derivatizing the aldehyde moiety used in the aldolization reaction^36^ was another method used to reduce the toxicity observed with compounds derived from benzaldehyde. Both naphthaldehyde and anthracene-carboxaldehyde were chosen as more bulky alternatives to benzaldehyde that would retain the same types of π–π stacking interactions. In practice, the naphthalene group did not reduce the toxicity toward the amoeba for compound **26** compared to its benzaldehyde-derived analogue **22**. The bulkiness of the anthracene-carboxaldehyde moiety, however, appeared to have a positive effect as it reduced toxicity toward the amoeba to an IC_50_ of 15.87 μM for compound **27** (Table 5). In the end, compound **27** was the best candidate with improved activity compared to its natural analogues, with an IC_50_ of 0.59 μM on intracellular Mm and 15.87 μM against Dd growth, representing a 25-fold specificity window, making it a promising strict anti-infective candidate for further investigations.

As all NPs previously introduced were inactive against Pa, synthetic derivatives were not tested against that strain. However, they were all tested against Sa and some showed improved activity, with the overall best candidate also being compound **27**. Interestingly, in the case of *S. aureus*, there was a difference in activity between the monochlorinated chalcone derivatives (**21** and **22** with MICs at 32 μg mL^–1^) and the dichlorinated chalcone (**23**), which had significantly lower MICs at 4 μg mL^–1^. This distinction was not observable with Mm in infection, where all three patterns of chlorination yielded identical IC_50_ values (1.76 μM).

### Hemisynthesis to Derivatize a Bioactive NP

Additional experiments to correlate biological readouts between NPs and synthetic analogues were made through hemisynthesis. The same chlorination reaction was carried out on an isolated aliquot of compound **4**, resulting in both its 3-chloro- (**28**) and 3,5-dichloro-derivatives (**29**) being successfully isolated (Figure 6, Table 6). No monochlorinated 5-chloro-derivative was observed in sufficient quantity to be isolated, which can be explained by strong para-directing effects in the EAS mechanism exerted by the hydroxyl groups present in the molecule.^37^ In that respect, the hydroxyl group at position 6 had the most notable effect in directing the chlorination at position 3 as it was unsubstituted. The formation of the dichlorinated analogue (**29**) was likely due to some excess chlorinating reagent being involved in the second EAS step on position 5 of monochlorinated product **28**.

**Figure 6:**
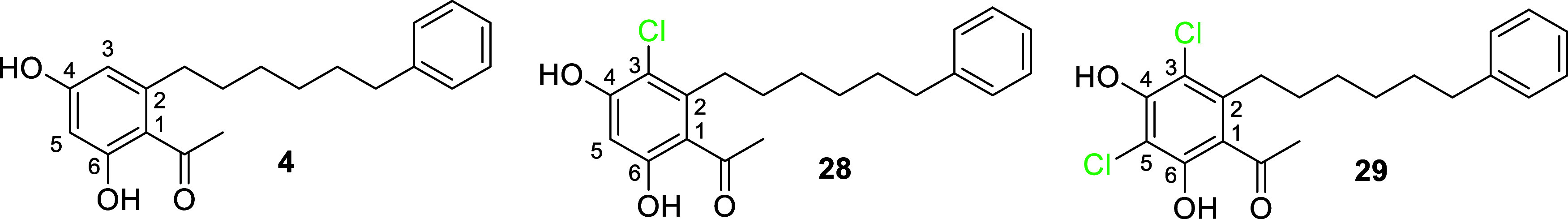
Hemisynthetic chlorinated derivatives of compound **4**.

**Table 6:** Biological Activities of Hemisynthetic Chlorinated Derivatives of Compound **4**

compound	IC_50_ on Mm in infection (μM)	IC_50_ on Dd in infection (μM)	IC_50_ on Mm *in broth* (μM)	MIC on Sa (μg·mL^–1^)
**4**	22.22	22.22	not active	4
**28**	2.47	0.82	22.22	4
**29**	2.47	7.41	not active	4–8

Chlorination of **4** resulted in an increase of potency for both anti-infective activity and Dd inhibition, while the activity on Sa remained largely unchanged. This difference in bioactivity between both experiments (anti-infective on Mm vs antibacterial on Sa) suggested that the compounds may be acting through different mechanisms of action.

## Conclusion

This bioguided study eventually led to the identification of 12 NPs as a first phytochemical report on the species *K. oblongifolia*, among which 7 structures were newly described. The two prominent structural backbones found in the plant (2,4-dihydroxyacetophenone and salicylic acid) were associated with growth inhibition of both Mm and Dd, but limited to the infection model, with no activity observed on the naked mycobacteria. Both scaffolds were also active on Gram-positive Sa, which is in line with extensive reports of such antibacterial activities especially for anacardic acid derivatives.^38,39^ In terms of anti-infective activities, however, the unmodified NPs presented limited selectivity toward Mm as they were equally active to reduce the growth of Dd.

Subsequent synthetic derivatization of the 2,4-dihydroxyacetophenone scaffold resulted in a series of derivatives with improved inhibitory activity on both Mm in infection and*S. aureus*. The chlorination of the acetophenone had a beneficial effect on the anti-infective activity, as did the reduction of size of the aliphatic chain compared to natural analogues and the increase in size of the aromatic unsubstituted parts of the NPs. Compound **27** was the best candidate for both models, having an IC_50_ of 0.59 μM on Mm (with an improvement in its selectivity against Mm as the IC_50_ on the amoeba stood at 15.87 μM) as well as an MIC of 2 μg mL^–1^ on Sa. Such activities recorded on a low MW molecule (374 g·mol^–1^) held promise in terms of room for further functional improvements in structure–activity relationship investigations. This compound is also a promising candidate for the relatively simple chemistry and a cheap two-step synthetic route it relies on.

Chlorination on the NP **4** also showed a beneficial effect in improving both its absolute value of IC_50_ against Mm and its therapeutic window compared to the host growth inhibition (dichlorination mostly). It seemed though that fully synthetic analogues inspired from the isolated NPs showed more potential in terms of anti-infective activities.

## Experimental Section

### General Experimental Procedures

NMR data were collected on a Bruker Avance III HD 600 MHz NMR spectrometer equipped with a QCI 5 mm cryoprobe and a SampleJet automated sample changer (Bruker BioSpin, Rheinstetten, Germany). Chemical shifts are presented in parts per million (δ), referencing the residual CDCl_3_ (for NPs, δH 7.26; δC 77.0) or DMSO-*d*_6_ (for synthetic molecules, δH 2.50; δC 39.5) signals as internal standards for 1H and 13C NMR, respectively, with coupling constants (J) reported in Hz. Full assignments were determined through 2D NMR experiments (COSY, NOESY, HSQC, and HMBC). HRMS data were acquired using an Orbitrap Exploris 120 mass spectrometer (Thermo Scientific, Germany) with a heated electrospray (H-ESI) source. Fraction contents were monitored using a multidetection UHPLC-PDA-ELSD-MS platform (Waters) equipped with a single quadrupole detector and heated electrospray ionization. Analytical HPLC utilized an Agilent Model 1260 system with a photodiode array detector (Agilent Technologies, Santa Clara, CA, USA). Semipreparative HPLC was conducted on a modular system (puriFlash-MS 4250, INTERCHIM, Montluçon, France) equipped with a quaternary pump, a UV detector module, and a fraction collector.

### Plant Material

The plant containing the compounds of interest was *K. oblongifolia* (King) Warb. This plant belongs to the Pierre Fabre Laboratories (PFL) collection with over 17,000 unique samples collected worldwide. The PFL collection was registered with the European Commission under accession number 03-FR-2020. This registration certifies that the collection meets the criteria set out in the EU ABS Regulation, which implements at the EU level the requirements of the Nagoya Protocol regarding access to genetic resources and the fair and equitable sharing of benefits arising from their utilization (Sharing nature, 2022). PFL supplied all of the vegetal material (ground dry material). The plant material was dried for 3 days at 55 °C in an oven; then the material was ground and stored in plastic pots at controlled temperature and humidity in PFL facilities. The leaves were the chosen plant part used for the plant studied, with their following unique IDs within the PFL collection: V113295 (*K. oblongifolia* leaves).

### Plant Extraction

The plant material was extracted in a Thermo Scientific Dionex ASE 350 Accelerated Solvent Extractor. The roots of *K. oblongifolia* (35.78 g) were extracted in a 100 mL pressure-resistant stainless steel extraction cell using the ASE system. At the bottom and top of the cell, a cellulose filter (Dionex) was added to prevent solid particles from reaching the system. The cell was loaded in the tray then pressurized and extracted with hexane, ethyl acetate, and methanol, respectively. The rinse volume was set at 60% and the temperature at 40 °C, with 3 cycles for each solvent (6 cycles for the ethyl acetate extracts) and a static time set at 5 min. The resulting extracts were collected in round-bottom flasks, combined and evaporated to dryness on a rotary evaporator (Büchi Rotavapor R114 Labortechnik AG, Switzerland) for each solvent to constitute the final extracts. The following extraction yields were obtained:*K. oblongifolia* hexane 659.4 mg (1.84%), ethyl acetate 628.1 mg (1.76%), and methanol 1.1617 g (3.25%).

### UHPLC-PDA-ELSD-MS Analyses of Fractions

Aliquots (50 μL) of fractions were analyzed by UHPLC-PDA-ELSD-MS. The conditions for the ESI (Waters Acquity QDA Detector) were set as follows: capillary voltage 0.8 kV (negative ion mode) or 1.2 kV (positive ion mode), cone voltage 15 V, probe temperature 600 °C, source temperature 120 °C. Detection was performed in negative (NI) then positive ion mode (PI) with an *m*/*z* range of 150–1250 Da. The separation was done on an Acquity UPLC BEH C18 column (50 × 2.1 mm inner diameter, 1.7 μm; Waters) at 0.6 mL/min, 40 °C with H_2_O (A) and MeCN (B) both containing 0.1% formic acid. The following gradient was applied for the separation: from 5 to 100% of B from 0 to 7 min, 1 min at 100% B, and a re-equilibration step of 2 min. The ELSD was set at 45 °C, with a gain of 3. The PDA detector (Waters Acquity) was set in the range from 190 to 500 nm, with a resolution of 1.2 nm. Sampling rate was set at 20 points/s.

### UHPLC-HRMS/MS of Extracts, Fractions, and Pure Compounds

Analyses were performed with a Waters Acquity UHPLC system coupled to a Corona Veo RS Charged Aerosol Detector (CAD, Thermo Scientific, Germany) and an Orbitrap Exploris 120 mass spectrometer (Thermo Scientific, Germany). The Orbitrap employed a heated electrospray ionization source (H-ESI) with the following parameters: spray voltage: +3.5 kV; ion transfer tube temperature: 320.00 °C; vaporizer temperature: 320.00 °C; S-lens RF: 45 (arb units); sheath gas flow rate: 35.00 (arb units); sweep gas (arb): 1; and auxiliary gas flow rate: 10.00 (arb. units). Control of the instruments was done using Thermo Scientific Xcalibur software v. 4.6.67.17. Full scans were acquired at a resolution of 30,000 fwhm (at *m*/*z* 200) and MS2 scans at 15,000 fwhm in the range of 100–1000 *m*/*z*, with 1 microscan, time (ms): 200, an RF lens (%): 70; AGC target custom (Normalized AGC target (%): 300); maximum injection time (ms): 130; microscans: 1; data type: profile; Use EASY-IC­(TM): ON. The settings for dynamic exclusion mode were customized; exclude after n times: 1; exclusion duration (s): 5; mass tolerance: ppm; low: 10, high: 10, exclude isotopes: true. Apex detention: Desired Apex Window (%): 50. Isotope exclusion: assigned and unassigned with an exclusion window (*m*/*z*) for unassigned isotopes: 8. The intensity threshold was set to 2.5 × 10^5^, and a targeted mass exclusion list was used.

The centroid data-dependent MS2 scan acquisition events were performed in discovery mode, triggered by Apex detection with a trigger detection (%) of 300 with a maximum injection time of 120 ms, performing 1 microscan. The top 3 abundant precursors (charge states 1 and 2) within an isolation window of 1.2 *m*/*z* were considered for MS/MS analysis. For precursor fragmentation in the HCD mode, a normalized collision energy of 15, 30, and 45% was used. Data was recorded in profile mode (Use EASY-IC­(TM): ON).

The chromatographic separation was done on a Waters BEH C18 column (50 × 2.1 mm i.d., 1.7 μm, Waters, Milford, MA) using the following gradient (time (min), %B): 5%B from 0 to 0.5 min; from 5%B to 100%B between 0.5 and 7 min; 100%B from 7 to 8 min, from 100%B to 5%B from 8 to 8.10 min; and 5%B from 8.10 to 10 min. The mobile phases were (A) H_2_O and (B) acetonitrile, both containing 0.1% formic acid. The flow rate was set to 600 μL/min, the injection volume was 2 μL, and the column was kept at 40 °C. The PDA detector was used from 210 to 400 nm with a resolution of 1.2 nm. The CAD detector was kept at 40 °C, with 5 bar of N_2_ and power function 1 for a data collection rate of 20 Hz.

### HPLC-PDA Gradient Optimizations on the Crude Extract

The analysis was carried out using an HP 1260 system equipped with a diode-array detection unit from Agilent Technologies in Santa Clara, CA, United States. An INTERCHIM puriFlash HQ C18 column (250 × 4.6 mm inner diameter, 15 μm, Montluçon, France) was employed. Detection was performed using a PDA, with UV wavelengths set at 220, 254, 280, and 329 nm. UV spectra between 190 and 500 nm were recorded with a threshold of 10 mAU and setting increments of 2 nm. HPLC conditions involved a mobile phase of H_2_O (A) and MeOH (B), both containing 0.1% FA. The flow rate was set at 1 mL/min, with an injection volume of 10 μL. The separation temperature was maintained at 25 °C, and the sample concentration was 10 mg/mL dissolved in MeOH. The gradient of the mobile phases was programmed as follows: an initial hold of 3 min at 70% B, followed by a gradient flow of 70%–87.5% of B over 39 min, followed by 3 min during which the gradient was kept at 87.5%, then another gradient flow of 87.5%–100% of B over 19 min, ending with a 6 min washing step with 100% B. These optimized HPLC analytical conditions were geometrically transferred by gradient transfer to the flash-LC scale.^26^

### UV-Flash Chromatography on the Crude Extract

The ethyl acetate extract of *K. oblongifolia* (leaves) was purified with a Büchi Flash chromatography system (Büchi Pump Module C-605, UV Photometer C-640, Control Unit C-620, Fraction Collector C-660), using an INTERCHIM puriFlash HQ C18 column (120 g, 210 × 30 mm i.d., 15 μm, Moluçon, France). 0.5 g of extract was mixed in the stationary phase (C18 Zeoprep 40–63 μm) and sand (50–70 mesh particle size) in a proportion of 1:1:1 and then introduced in a dry load cell. The detection was performed by a UV Photometer with the parameters set as follows: UV wavelengths were 220, 254, 280, and 329 nm. The mobile phase was composed of H_2_O (A) and technical grade methanol (B), both containing 0.1% F.A (flow rate of 30 mL/min). The gradient slope was set as follows: an initial hold of 4 min at 70% B, a gradient flow of 70%–87.5% of B in 56 min, followed by a hold of 4 min at 87.5%, then another gradient flow of 87.5%–100% in 27 min, followed by 9 min of washing at 100% B. The separation yielded 70 fractions of 50 mL each (F01–F70) that were dried using a multiunit evaporator (Multivapor, Büchi Labortechnik AG, Switzerland). The following compounds were identified after HRMS and NMR analyses for confirmations: **1** (F08, 2.9 mg, RT 12 min), **2** (F09, 2.8 mg, RT 13 min), **3** (F14, 2.4 mg, RT 22 min), **4** (F15, 6.7 mg, RT 24 min), **5** (F20, 5.3 mg, RT 32 min), **6** (F22–23, 20.8 mg, RT 35 min), **7** (F24–25, 11.8 mg, RT 39 min), **8** (F30–31, 8.6 mg, RT 48 min), **9** (F33, 2.5 mg, RT 53 min), **10** (F34–35, 5 mg, RT 56 min), **11** (F39–40, 6.7 mg, RT 64 min), and **12** (F48, 3.1 mg, RT 79 min).

Knemolone A (**1**) Yellow amorphous solid; UV (MeOH) λ_max_ (log ε) 201 (3.83), 222 (4.09), 285 (3.35) nm; NMR see in Table 2, NP-MRD ID: NP0333015; HRESIMS *m*/*z* 327.1237 [M-H]^−^ (calcd for C_19_H_19_O_5_^–^ 327.1238, Δ = −0.31 ppm), *m*/*z* 329.1382 [M + H]^+^ (calcd for C_19_H_21_O_5_^+^ 329.1384, Δ = −0.61 ppm), MS/MS spectrum: CCMSLIB00012475055.

Knemolone B (**2**) Yellow amorphous solid; UV (MeOH) λ_max_ (log ε) 222 (3.95), 282 (3.03) nm; NMR see in Table 2, NP-MRD ID: NP0333016; HRESIMS *m*/*z* 283.1337 [M-H]^−^ (calcd for C_18_H_19_O_3_^–^ 283.1340, Δ = −1.06 ppm), *m*/*z* 285.1483 [M + H]^+^ (calcd for C_18_H_21_O_3_^+^ 285.1485, Δ = −0.70 ppm), MS/MS spectrum: CCMSLIB00012475054.

Knemolic acid A (**3**) Yellow amorphous solid; UV (MeOH) λ_max_ (log ε) 219 (4.04), 288 (3.06) nm; NMR see in Table 3, NP-MRD ID: NP0333017; HRESIMS *m*/*z* 313.1079 [M-H]^−^ (calcd for C_18_H_17_O_5_^–^ 313.1081, Δ = −0.64 ppm), *m*/*z* 297.1119 [M-H_2_O + H]^+^ (calcd for C_18_H_17_O_4_^+^ 297.1121, Δ = −0.67 ppm), MS/MS spectrum: CCMSLIB00012475067.

Knemolone C (**4**) Yellow amorphous solid; UV (MeOH) λ_max_ (log ε) 193 (5.10), 217 (4.79), 282 (4.33) nm; NMR see in Table 2, NP-MRD ID: NP0333018; HRESIMS *m*/*z* 311.1651 [M-H]^−^ (calcd for C_20_H_23_O_3_^–^ 311.1653, Δ = −0.64 ppm), *m*/*z* 313.1796 [M + H]^+^ (calcd for C_20_H_25_O_3_^+^ 313.1789, Δ = 2.23 ppm), MS/MS spectrum: CCMSLIB00012475059.

Knemolone D (**5**) Yellow amorphous solid; UV (MeOH) λ_max_ (log ε) 196 (4.17), 222 (4.44), 282 (3.96) nm; NMR see in Table 2, NP-MRD ID: NP0333019; HRESIMS *m*/*z* 277.1808 [M-H]^−^ (calcd for C_17_H_25_O_3_^–^ 277.1809, Δ = −0.36 ppm), *m*/*z* 279.1949 [M + H]^+^ (calcd for C_17_H_27_O_3_^+^ 279.1955, Δ = −2.15 ppm), MS/MS spectrum: CCMSLIB00012475056.

Knemolic acid B (**6**) Yellow-green amorphous solid; UV (MeOH) λ_max_ (log ε) 196 (5.11), 214 (5.08), 233 (4.82), 288 (4.57), 310 (4.33) nm; NMR see in Table 3, NP-MRD ID: NP0333020; HRESIMS *m*/*z* 341.1391 [M-H]^−^ (calcd for C_20_H_21_O_5_^–^ 341.1394, Δ = −0.88 ppm), *m*/*z* 325.1430 [M-H_2_O + H]^+^ (calcd for C_20_H_21_O_4_^+^ 325.1434, Δ = −1.23 ppm), MS/MS spectrum: CCMSLIB00012475058.

Knemolic acid C (**7**) Yellow-green amorphous solid; UV (MeOH) λ_max_ (log ε) 193 (5.01), 210 (4.94), 240 (4.33), 310 (3.98) nm; NMR see in Table 3, NP-MRD ID: NP0333021; HRESIMS *m*/*z* 297.1495 [M-H]^−^ (calcd for C_19_H_21_O_3_^–^ 297.1496, Δ = −0.34 ppm), *m*/*z* 281.1530 [M-H_2_O + H]^+^ (calcd for C_19_H_21_O_2_^+^ 281.1536, Δ = −2.13 ppm), MS/MS spectrum: CCMSLIB00012475057.

Khookerianic acid A (**8**) Green amorphous solid; UV (MeOH) λ_max_ (log ε) 211 (4.51), 310 (3.62) nm; ^1^H NMR (CDCl_3_, 600 MHz): δ 7.36 (1H, t, *J* = 7.9 Hz), 6.87 (1H, dd, *J* = 8.3, 1.1 Hz), 6.80–6.73 (1H, m), 3.00–2.95 (2H, m), 1.64–1.55 (2H, m), 1.41–1.33 (2H, m), 1.34–1.22 (10H, m), 0.87 (3H, t, *J* = 7.0 Hz); ^13^C NMR (CDCl_3_, 151 MHz): δ 175.7, 163.8, 147.9, 135.5, 122.9, 116.0, 110.6, 36.6, 32.2, 32.0, 30.0, 29.7, 29.6, 29.5, 22.8, 14.3 (NP-MRD ID: NP0333022); HRESIMS *m*/*z* 263.1651 [M-H]^−^ (calcd for C_16_H_23_O_3_^–^ 263.1653, Δ = −0.76 ppm), *m*/*z* 265.1794 [M + H]^+^ (calcd for C_16_H_25_O_3_^+^ 265.1798, Δ = −1.51 ppm), MS/MS spectrum: CCMSLIB00012475061.

Kneglobularic acid B (**9**) Green amorphous solid; UV (MeOH) λ_max_ (log ε) 224 (3.93), 288 (3.66) nm; ^1^H NMR (CDCl_3_, 600 MHz): δ 7.34 (1H, t, *J* = 7.9 Hz), 6.85 (1H, dd, *J* = 8.3, 1.2 Hz), 6.75 (1H, dd, *J* = 7.5, 1.1 Hz), 6.71 (1H, d, *J* = 7.8 Hz), 6.67 (1H, d, *J* = 1.7 Hz), 6.61 (1H, dd, *J* = 7.8, 1.7 Hz), 5.91 (2H, s), 2.94 (2H, t, *J* = 7.9 Hz), 2.51 (2H, t, *J* = 7.8 Hz), 1.60–1.53 (4H, m), 1.32–1.28 (8H, m); ^13^C NMR (CDCl_3_, 151 MHz): δ 174.2, 163.7, 147.5, 145.5, 137.0, 135.3, 122.7, 121.2, 115.9, 110.5, 109.0, 108.2, 100.8, 36.6, 35.8, 32.2, 31.8, 29.9, 29.5, 29.5, 29.2 (NP-MRD ID: NP0333023); HRESIMS *m*/*z* 369.1705 [M-H]^−^ (calcd for C_22_H_25_O_5_^–^ 369.1707, Δ = −0.54 ppm), *m*/*z* 353.1744 [M-H_2_O + H]^+^ (calcd for C_22_H_25_O_4_^+^ 353.1747, Δ = −0.85 ppm), MS/MS spectrum: CCMSLIB00012475062.

Khookerianic acid C/Kneglobularic acid A (**10**) Green amorphous solid; UV (MeOH) λ_max_ (log ε) 191 (4.58), 212 (4.58), 310 (3.55) nm; ^1^H NMR (CDCl_3_, 600 MHz): δ 7.35 (1H, t, *J* = 7.9 Hz), 7.28–7.24 (2H, m), 7.19–7.15 (3H, m), 6.86 (1H, dd, *J* = 8.4, 1.2 Hz), 6.76 (1H, dd, *J* = 7.5, 1.2 Hz), 2.96 (2H, t, *J* = 7.9 Hz), 2.60 (2H, t, *J* = 7.8 Hz), 1.62–1.57 (4H, m), 1.36–1.29 (8H, m); ^13^C NMR (CDCl_3_, 151 MHz) δ 175.1, 163.8, 147.7, 143.1, 135.5, 128.5, 128.4, 125.7, 122.8, 116.0, 110.5, 36.6, 36.1, 32.2, 31.6, 29.9, 29.6, 29.5, 29.4 (NP-MRD ID: NP0333024); HRESIMS *m*/*z* 325.1808 [M-H]^−^ (calcd for C_21_H_25_O_3_^–^ 325.1809, Δ = −0.31 ppm), *m*/*z* 309.1845 [M-H_2_O + H]^+^ (calcd for C_21_H_25_O_2_^+^ 309.1849, Δ = −1.29 ppm), MS/MS spectrum: CCMSLIB00012475063.

Anagigantic acid (**11**) Dark-green amorphous solid; UV (MeOH) λ_max_ (log ε) 213 (4.46), 311 (3.58) nm; ^1^H NMR (CDCl_3_, 600 MHz): δ 7.35 (1H, t, *J* = 7.9 Hz), 6.86 (1H, d, *J* = 8.3 Hz), 6.77 (1H, d, *J* = 7.4 Hz), 2.96 (2H, t, *J* = 7.8 Hz), 1.59 (2H, p, *J* = 7.6 Hz), 1.36 (2H, dd, *J* = 10.3, 5.2 Hz), 1.27 (14H, d, *J* = 13.0 Hz), 0.87 (3H, t, *J* = 7.0 Hz); ^13^C NMR (CDCl_3_, 151 MHz) δ 175.45, 163.76, 147.85, 135.48, 122.86, 115.98, 110.55, 36.64, 32.19, 32.07, 29.97, 29.83, 29.80, 29.78, 29.65, 29.50, 22.84, 14.27 (NP-MRD ID: NP0333025); HRESIMS *m*/*z* 291.1964 [M-H]^−^ (calcd for C_18_H_27_O_3_^–^ 291.1966, Δ = −0.69 ppm), *m*/*z* 293.2108 [M + H]^+^ (calcd for C_18_H_29_O_3_^+^ 293.2111, Δ = −1.02 ppm), MS/MS spectrum: CCMSLIB00012475070.

6-Tridecylsalicylic acid (**12**) Dark-green amorphous solid; UV (MeOH) λ_max_ (log ε) 225 (4.00), 311 (2.80) nm; ^1^H NMR (CDCl_3_, 600 MHz): δ 7.34 (1H, dd, *J* = 8.3, 7.5 Hz), 6.85 (1H, dd, *J* = 8.4, 1.2 Hz), 6.76 (1H, dd, *J* = 7.5, 1.2 Hz), 2.98–2.92 (2H, m), 1.58 (2H, p, *J* = 7.6 Hz), 1.35 (2H, s), 1.27–1.24 (18H, m), 0.88 (3H, t, *J* = 7.0 Hz); ^13^C NMR (CDCl_3_, 151 MHz): δ 174.5, 163.8, 147.7, 135.3, 122.8, 115.9, 110.5, 36.7, 32.2, 32.1, 30.0, 29.8, 29.8, 29.8, 29.8, 29.7, 29.5, 22.8, 14.3 (NP-MRD ID: NP0333026); HR–ESI–MS *m*/*z* 319.2277 [M-H]^−^ (calcd for C_20_H_31_O_3_^–^ 319.2279, Δ = −0.63 ppm), *m*/*z* 303.2318 [M-H_2_O + H]^+^ (calcd for C_20_H_31_O_2_^+^ 303.2319, Δ = −0.33 ppm), MS/MS spectrum: CCMSLIB00012475069.

### Generic Procedure Used for Halogenation Reactions

This procedure was inspired by that described in Wu et al. (2020).^35^ To a solution of 2,4-dihydroxyacetophenone (5 g, 1 equiv) in EtOH (50 mL) was added con. H_2_SO_4_ (1 equiv) at room temperature; the mixture was stirred for 5 min. Then, N-chlorosuccinimide (NCS) or N-bromosuccinimide (NBS) (1 equiv) was added to the mixture. The reaction was monitored by using an UHPLC-PDA-ELSD-MS instrument. With NBS, the reaction was generally instantaneous, while with NCS, the reaction was left to stir for 24 h. The mixture was evaporated to dryness and redissolved in EtOAc (500 mL). The organic layer was washed with H_2_O (2 × 500 mL) and then brine (500 mL), before being dried with MgSO4 and evaporated to dryness to obtain 6.3314 g (with NCS) and 4.8777 g (with NBS) of the product as a white solid (NCS)/yellow solid (NBS) mixture of 3 compounds (2 monohalogenated isomers +1 dihalogenated compound).

For the chlorination reaction, a mass of 2.92 g of the mixture was then subjected to flash chromatography using a BGB Scorpius C18e-HP column (155 g, 220 × 48 mm i.d., 30 μm, Bockten, Switzerland). The mobile phases were (A) H_2_O and (B) technical grade methanol, both containing 0.1% F.A (flow rate of 50 mL/min). The gradient slope was set as follows: an initial hold of 2 min at 20% B, a gradient flow of 20%–40% of B in 59 min, followed by a gradient flow of 47 min from 40%–65%, then another gradient flow of 65%–100% in 16 min. The separation yielded 112 fractions of 50 mL each (F01–F112) that were combined according to their respective peaks and evaporated to dryness. The following compounds were identified after HRMS and NMR analyses for confirmations: **15** (F46–59, 990.2 mg, 35.0%, RT 43–57 min), **16** (F61–78, 1.1116 g, 39.3%, RT 57–73 min), and **17** (F81–87, 100 mg, 3.54%, RT 75–81 min).

For the bromination reaction, a mass of 4.6 g of the mixture was then subjected to flash chromatography using a BGB Scorpius C18e-HP column (155 g, 220 × 48 mm i.d., 30 μm, Bockten, Switzerland). The mobile phases were (A) H_2_O and (B) technical grade methanol, both containing 0.1% F.A (flow rate of 50 mL/min). The gradient slope was set as follows: an isocratic gradient of 65% B during 74 min, followed by a gradient flow of 12 min from 65%–100% followed by an isocratic washing step at 100% B for 17 min. The separation yielded 103 fractions of 50 mL each (F01–F103) that were combined according to their respective peaks and evaporated to dryness. The following compounds were identified after HRMS and NMR analyses for confirmations: **18** (F28–47, 1.7603 g, 24.7%, RT 30–49 min), **19** (F49–88, 1.2801 g, 18.0%, RT 50–89 min), and **17** (F90–92, 622.4 mg, 6.52%, RT 90–92 min).

### Generic Procedure Used for All Aldolization Reactions

This procedure was inspired by that described in Devakaram et al. (2011).^36^ To a solution of ketone (200 mg, 1 equiv) in MeOH (10 mL), an aldehyde (1 equiv) was added, then 10 mL of a solution of 60% KOH in water was added, and the reaction was left to stir for 24 h. The reaction was monitored using an UHPLC-PDA-ELSD-MS instrument. Once over, the reaction was quenched with a cold 1 M HCl solution (100 mL), and the mixture was extracted with DCM (2 × 20 mL). The organic layer was dried with MgSO_4_ before being evaporated under reduced pressure to result in 248.9 mg of the aldolized product as a yellow powder (93%).

2,4-Dihydroxyacetophenone (**13**), white solid; ^1^H NMR (DMSO, 600 MHz): δ 12.60 (1H, s), 10.61 (1H, s), 7.75 (1H, d, *J* = 8.8 Hz), 6.37 (1H, dd, *J* = 8.8, 2.4 Hz), 6.24 (1H, d, *J* = 2.3 Hz), 2.52 (3H, s); ^13^C NMR (DMSO, 151 MHz): δ 202.7, 164.9, 164.2, 133.7, 112.9, 108.1, 102.3, 26.4; HR–ESI–MS *m*/*z* 153.0545 [M + H]^+^ (calcd for C_8_H_9_O_3_^+^ 153.0546, Δ = −0.65 ppm), *m*/*z* 151.0400 [M-H]^−^ (calcd for C_8_H_7_O_3_^–^ 151.0401, Δ = −0.66 ppm).

2,4-Dihydroxychalcone (**14**), yellow solid; ^1^H NMR (DMSO, 600 MHz): δ 13.38 (1H, d, *J* = 1.6 Hz), 10.75 (1H, s), 8.21 (1H, dd, *J* = 9.0, 3.3 Hz), 7.98 (1H, dd, *J* = 15.5, 3.4 Hz), 7.90 (2H, td, *J* = 4.9, 2.8 Hz), 7.80 (1H, dd, *J* = 15.4, 2.8 Hz), 7.47 (3H, td, *J* = 4.6, 2.0 Hz), 6.43 (1H, dt, *J* = 8.9, 2.2 Hz), 6.30 (1H, t, *J* = 2.0 Hz); ^13^C NMR (DMSO, 151 MHz): δ 191.5, 165.8, 165.3, 143.7, 134.6, 133.2, 130.7, 129.0, 128.9, 121.3, 113.0, 108.3, 102.6; HR–ESI–MS *m*/*z* 241.0856 [M + H]^+^ (calcd for C_15_H_13_O_3_^+^ 241.0859, Δ = −1.24 ppm), *m*/*z* 239.0711 [M-H]^−^ (calcd for C_15_H_11_O_3_^–^ 239.0714, Δ = −1.25 ppm).

3-Chloro-2,4-dihydroxyacetophenone (**15**), white solid; ^1^H NMR (DMSO, 600 MHz): δ 13.36 (1H, s), 7.77 (1H, dd, *J* = 9.0, 0.9 Hz), 6.59 (1H, d, *J* = 8.9 Hz), 2.57 (3H, s); ^13^C NMR (DMSO, 151 MHz): δ 203.4, 160.5, 159.9, 131.2, 113.0, 107.7, 106.7, 26.3; HR–ESI–MS *m*/*z* 187.0154 [M + H]^+^ (calcd for C_8_H_8_ClO_3_^+^ 187.0156, Δ = −1.07 ppm), *m*/*z* 185.0010 [M-H]^−^ (calcd for C_8_H_6_ClO_3_^–^ 185.0011, Δ = −0.54 ppm).

5-Chloro-2,4-dihydroxyacetophenone (**16**), white solid; ^1^H NMR (DMSO, 600 MHz): δ 12.34 (1H, s), 11.43 (1H, s), 7.89 (1H, s), 6.47 (1H, s), 2.55 (3H, s); ^13^C NMR (DMSO, 151 MHz): δ 202.1, 162.1, 159.9, 132.5, 113.7, 111.3, 103.5, 27.0; HR–ESI–MS *m*/*z* 187.0155 [M + H]^+^ (calcd for C_8_H_8_ClO_3_^+^ 187.0156, Δ = −0.53 ppm), *m*/*z* 185.0009 [M-H]^−^ (calcd for C_8_H_6_ClO_3_^–^ 185.0011, Δ = −1.08 ppm).

3,5-Dichloro-2,4-dihydroxyacetophenone (**17**), white solid; ^1^H NMR (DMSO, 600 MHz): δ 13.18 (1H, s), 7.99 (1H, s), 2.61 (3H, s); ^13^C NMR (DMSO, 151 MHz): δ 203.3, 158.2, 155.9, 130.4, 113.2, 112.2, 108.9, 26.6; HR–ESI–MS *m*/*z* 220.9767 [M + H]^+^ (calcd for C_8_H_7_Cl_2_O_3_^+^ 220.9767, Δ = 0 ppm), *m*/*z* 218.9619 [M-H]^−^ (calcd for C_8_H_5_Cl_2_O_3_^–^ 218.9621, Δ = −0.91 ppm).

3-Bromo-2,4-dihydroxyacetophenone (**18**), light brown solid; ^1^H NMR (DMSO, 600 MHz): δ 13.50 (1H, s), 11.50 (1H, s), 7.81 (1H, d, *J* = 8.8 Hz), 6.59 (1H, d, *J* = 8.9 Hz), 2.57 (3H, s); ^13^C NMR (DMSO, 151 MHz): δ 203.3, 161.6, 160.9, 132.1, 113.1, 107.6, 97.2, 26.2; HR–ESI–MS *m*/*z* 230.9650 [M + H]^+^ (calcd for C_8_H_8_BrO_3_^+^ 230.9651, Δ = −0.43 ppm), *m*/*z* 228.9505 [M-H]^−^ (calcd for C_8_H_6_BrO_3_^–^ 228.9506, Δ = −0.44 ppm).

5-Bromo-2,4-dihydroxyacetophenone (**19**), light yellow solid; ^1^H NMR (DMSO, 600 MHz): δ 12.34 (1H, s), 11.48 (1H, s), 8.01 (1H, s), 6.47 (1H, s), 2.55 (3H, s); ^13^C NMR (DMSO, 151 MHz): δ 202.0, 162.6, 160.8, 135.6, 114.4, 103.3, 100.0, 27.0; HR–ESI–MS *m*/*z* 230.9650 [M + H]^+^ (calcd for C_8_H_8_BrO_3_^+^ 230.9651, Δ = −0.43 ppm), *m*/*z* 228.9505 [M-H]^−^ (calcd for C_8_H_6_BrO_3_^–^ 228.9506, Δ = −0.44 ppm).

3,5-Dibromo-2,4-dihydroxyacetophenone (**20**), light yellow solid; ^1^H NMR (DMSO, 600 MHz): δ 13.34 (1H, s), 12.34 (0H, s), 8.14 (1H, s), 2.62 (3H, s); ^13^C NMR (DMSO, 151 MHz): δ 203.2, 159.7, 157.5, 134.3, 114.3, 100.8, 99.5, 26.5; HR–ESI–MS *m*/*z* 308.8756 [M + H]^+^ (calcd for C_8_H_7_Br_2_O_3_^+^ 308.8756, Δ = 0 ppm), *m*/*z* 306.8615 [M-H]^−^ (calcd for C_8_H_5_Br_2_O_3_^–^ 306.8611, Δ = 1.30 ppm).

3-Chloro-2,4-dihydroxychalcone (**21**), yellow solid; ^1^H NMR (DMSO, 600 MHz): δ 14.21 (1H, s), 11.56 (1H, s), 8.23 (1H, d, *J* = 9.0 Hz), 8.00 (1H, d, *J* = 15.5 Hz), 7.92 (2H, dd, *J* = 6.6, 2.9 Hz), 7.86 (1H, d, *J* = 15.4 Hz), 7.50–7.46 (3H, m), 6.63 (1H, d, *J* = 8.9 Hz); ^13^C NMR (DMSO, 151 MHz): δ 193.0, 161.5, 144.6, 134.5, 130.9, 130.6, 129.2, 129.0, 120.8, 113.1, 107.8, 106.9; HR–ESI–MS *m*/*z* 275.0467 [M + H]^+^ (calcd for C_15_H_12_ClO_3_^+^ 275.0469, Δ = −0.72 ppm), *m*/*z* 273.0322 [M-H]^−^ (calcd for C_15_H_10_ClO_3_^–^ 273.0324, Δ = −0.73 ppm).

5-Chloro-2,4-dihydroxychalcone (**22**), yellow solid; ^1^H NMR (DMSO, 600 MHz): δ 13.23 (1H, s), 8.42 (1H, s), 8.04 (1H, d, *J* = 15.4 Hz), 7.95 (2H, dd, *J* = 6.6, 3.0 Hz), 7.81 (1H, d, *J* = 15.4 Hz), 7.47 (3H, ddt, *J* = 5.7, 3.9, 2.2 Hz), 6.50 (1H, s); ^13^C NMR (DMSO, 151 MHz): δ 191.09, 163.8, 144.4, 134.6, 132.0, 130.8, 129.3, 128.9, 121.3, 113.6, 111.9, 103.7; HR–ESI–MS *m*/*z* 275.0467 [M + H]^+^ (calcd for C_15_H_12_ClO_3_^+^ 275.0469, Δ = −0.73 ppm), *m*/*z* 273.0323 [M-H]^−^ (calcd for C_15_H_10_ClO_3_^–^ 273.0324, Δ = −0.37 ppm).

3,5-Dichloro-2,4-dihydroxychalcone (**23**), yellow solid; ^1^H NMR (DMSO, 600 MHz): δ 14.15 (1H, s), 8.53 (1H, s), 8.08 (1H, d, *J* = 15.4 Hz), 8.00–7.96 (2H, m), 7.88 (1H, d, *J* = 15.4 Hz), 7.49 (3H, dd, *J* = 4.8, 1.9 Hz); ^13^C NMR (DMSO, 151 MHz): δ 190.1, 159.9, 144.3, 134.4, 131.1, 129.6, 129.5, 128.9, 120.7; HR–ESI–MS *m*/*z* 309.0078 [M + H]^+^ (calcd for C_15_H_11_Cl_2_O_3_^+^ 309.0080, Δ = −0.65 ppm), *m*/*z* 306.9933 [M-H]^−^ (calcd for C_15_H_9_Cl_2_O_3_^–^ 306.9934, Δ = −0.33 ppm).

3-Bromo-2,4-dihydroxychalcone (**24**), light brown solid; ^1^H NMR (DMSO, 600 MHz): δ 14.34 (1H, s), 13.50 (1H, s), 8.27 (1H, d, *J* = 9.0 Hz), 8.01 (1H, d, *J* = 15.4 Hz), 7.92 (2H, dd, *J* = 6.6, 2.9 Hz), 7.87 (1H, d, *J* = 15.4 Hz), 7.50–7.46 (3H, m), 6.63 (2H, dd, *J* = 28.9, 8.8 Hz); ^13^C NMR (DMSO, 151 MHz): δ 191.7, 162.5, 161.6, 144.7, 134.5, 131.5, 131.0, 129.2, 129.0, 120.7, 113.3, 107.7, 97.6; HR–ESI–MS *m*/*z* 318.9964 [M + H]^+^ (calcd for C_15_H_12_BrO_3_^+^ 318.9964, Δ = 0 ppm), *m*/*z* 316.9818 [M-H]^−^ (calcd for C_15_H_10_BrO_3_^–^ 316.9819, Δ = −0.32 ppm).

5-Bromo-2,4-dihydroxychalcone (**25**), light brown solid; ^1^H NMR (DMSO, 600 MHz): δ 13.23 (1H, s), 11.62 (1H, s), 8.53 (1H, s), 8.04 (1H, d, *J* = 15.4 Hz), 7.96–7.92 (2H, m), 7.81 (1H, d, *J* = 15.4 Hz), 7.46 (3H, t, *J* = 3.1 Hz), 6.52 (1H, d, *J* = 1.1 Hz); ^13^C NMR (DMSO, 151 MHz): δ 191.1, 164.3, 161.2, 144.5, 135.0, 134.6, 130.8, 129.3, 128.9, 121.3, 114.5, 103.5, 100.6; HR–ESI–MS *m*/*z* 318.9963 [M + H]^+^ (calcd for C_15_H_12_BrO_3_^+^ 318.9964, Δ = −0.31 ppm), *m*/*z* 316.9819 [M-H]^−^ (calcd for C_15_H_10_BrO_3_^–^ 316.9819, Δ = 0 ppm).

(E)-1-(5-Chloro-2,4-dihydroxyphenyl)-3-(naphthalen-1-yl)­prop-2-en-1-one (**26**), yellow solid; ^1^H NMR (DMSO, 600 MHz): δ 13.19 (1H, s), 8.64 (1H, dd, *J* = 15.2, 3.5 Hz), 8.43 (1H, d, *J* = 3.9 Hz), 8.35 (1H, d, *J* = 7.1 Hz), 8.30 (1H, d, *J* = 8.4 Hz), 8.13 (1H, dd, *J* = 15.2, 4.3 Hz), 8.08 (1H, d, *J* = 8.0 Hz), 8.01 (1H, d, *J* = 8.0 Hz), 7.69–7.56 (3H, m), 6.54 (1H, d, *J* = 2.4 Hz); ^13^C NMR (DMSO, 151 MHz): δ 190.9, 163.8, 160.4, 140.0, 133.4, 132.1, 131.3, 131.2, 131.0, 128.9, 127.4, 126.3, 126.2, 125.7, 123.7, 122.9, 113.8, 112.0, 103.8; HR–ESI–MS *m*/*z* 325.0626 [M + H]^+^ (calcd for C_19_H_14_ClO_3_^+^ 325.0626, Δ = 0 ppm), *m*/*z* 323.0477 [M-H]^−^ (calcd for C_29_H_12_ClO_3_^–^ 323.0480, Δ = −0.93 ppm).

(E)-3-(Anthracen-9-yl)-1-(5-chloro-2,4-dihydroxyphenyl)­prop-2-en-1-one (**27**), yellow solid; ^1^H NMR (DMSO, 600 MHz): δ 13.28 (1H, s), 8.64 (1H, s), 8.35 (2H, dd, *J* = 8.7, 1.3 Hz), 8.26 (1H, d, *J* = 15.8 Hz), 8.16–8.11 (3H, m), 7.94 (1H, dd, *J* = 14.6, 11.1 Hz), 7.66 (1H, d, *J* = 14.7 Hz), 7.58 (4H, dddd, *J* = 15.6, 7.8, 6.5, 1.4 Hz), 7.08 (1H, dd, *J* = 15.7, 11.1 Hz), 6.46 (1H, s); ^13^C NMR (DMSO, 151 MHz): δ 189.4, 163.5, 139.7, 131.9, 131.4, 130.9, 129.9, 128.9, 128.2, 126.8, 125.6, 125.2, 118.1, 113.6, 103.8; HR–ESI–MS *m*/*z* 375.0780 [M + H]^+^ (calcd for C_23_H_16_ClO_3_^+^ 375.0782, Δ = −0.80 ppm), *m*/*z* 373.0635 [M-H]^−^ (calcd for C_23_H_14_ClO_3_^–^ 373.0637, Δ = −0.54 ppm).

### Procedure for the Halogenation Reaction on Knemolone C (**4**)

This procedure was inspired by that described in Wu et al. (2020).^35^ To a solution of Knemolone C (**4**, 3.05 mg 1 equiv) in EtOH (0.5 mL) was added con. H_2_SO_4_ (0.549 μL, 1.05 equiv) at room temperature, and the mixture was stirred for 5 min. Then, N-chlorosuccinimide (NCS) (1.304 mg, 1 equiv) was added to the mixture. The reaction was monitored using an UHPLC-PDA-ELSD-MS instrument (reaction was left to stir for 24 h). The mixture was evaporated to dryness and redissolved in EtOAc (10 mL). The organic layer was washed with H_2_O (2 × 10 mL) and then brine (10 mL), before being dried with MgSO_4_ and evaporated to dryness to obtain 2.07 mg of the product as a white solid mixture, which was then dissolved in 200 μL of EtOAc and subjected to HPLC microfractionation using a XBridge BEH C18 OBD Prep column (130 Å, 250 × 10 mm, 5 μm, Waters Corporation, Milford, MA). The mobile phases were (A) H_2_O and (B) HPLC grade acetonitrile, both containing 0.1% F.A (flow rate of 4.7 mL/min). The gradient slope was set as follows: an initial hold at 50% of B during 1 min, followed by a gradient flow of 19 min from 50%–80% followed by a gradient flow from 80%–100% of B for 5 min, and finally a washing step of 5 min at 100% B. The separation yielded 96 fractions (A01–H12) that were combined according to their respective peaks and evaporated to dryness. The following compounds were identified after HRMS and NMR analyses for confirmations: **28** (E3–E4, 0.81 mg, 23.9%, RT 16 min) and **29** (E11–E12, 0.25 mg, 6.74%, RT 19 min).

1-(3-Chloro-4,6-dihydroxy-2-(6-phenylhexyl)­phenyl)­ethan-1-one (**28**); ^1^H NMR (DMSO, 600 MHz): δ 10.23 (1H, s), 9.98 (1H, s), 7.29–7.23 (2H, m), 7.20–7.12 (3H, m), 6.44 (1H, s), 2.55 (2H, t, *J* = 7.8 Hz), 2.49–2.46 (2H, m), 2.39 (3H, s), 1.55 (2H, p, *J* = 7.4 Hz), 1.42 (2H, p, *J* = 6.9 Hz), 1.30 (4H, dq, *J* = 9.4, 4.1 Hz); ^13^C NMR (DMSO, 151 MHz): δ 203.4, 154.4, 154.0, 142.3, 138.6, 128.2, 128.2, 125.6, 122.0, 111.2, 101.3, 35.1, 32.4, 30.9, 30.2, 29.4, 28.9, 28.2; HR–ESI–MS *m*/*z* 347.1408 [M + H]^+^ (calcd for C_20_H_24_ClO_3_^+^ 347.1408, Δ = 0 ppm), *m*/*z* 345.1269 [M-H]^−^ (calcd for C_20_H_22_ClO_3_^–^ 345.1263, Δ = 1.74 ppm).

1-(3,5-Dichloro-2,4-dihydroxy-6-(6-phenylhexyl)­phenyl)­ethan-1-one (**29**); ^1^H NMR (DMSO, 600 MHz): δ 10.12 (1H, s), 8.13 (1H, s), 7.26 (2H, t, *J* = 7.6 Hz), 7.20–7.13 (3H, m), 2.56 (2H, t, *J* = 7.5 Hz), 2.52 (2H, t, *J* = 1.9 Hz), 2.43 (3H, s), 1.56 (2H, p, *J* = 7.5 Hz), 1.44 (2H, p, *J* = 7.8 Hz), 1.31 (4H, dq, *J* = 11.5, 5.6 Hz); ^13^C NMR (151 MHz, DMSO): δ 201.9, 142.2, 142.2, 128.2, 128.0, 125.6, 35.0, 30.8, 28.5, 28.1, 28.1 (partial data, sample quantity too low for adequate ^13^C NMR resolution); HR–ESI–MS ± no corresponding ion observed (calcd for C_20_H_23_Cl_2_O_3_^+^ 381.1019), (calcd for C_20_H_25_Cl_2_O_3_^–^ 379.0873).

### Bioactivity Screening (Mm)

Plant extracts, fractions, or isolated compounds were stored at −20 °C and wrapped in aluminum foil if necessary. All manipulations with extracts, fractions, or compounds were performed under a sterile hood. Extracts, fractions, and compounds were resuspended throughout in DMSO to best solubilize extracts with diverse constituents. Extracts, fractions, or compounds were added to the assay plate in a 1:100 dilution. Assay solutions of extracts, fractions, and compounds were prepared in 96-well plates, stored at −20 °C, and thawed before the experiment at room temperature or warmer with or without shaking/vortexing to obtain a clear assay solution. Extracts were tested at 25 μg/mL and purified compounds in a dose–response curve of 6 concentrations with 100 μM being the top concentration and a 1:3 dilution step between each testing concentration. Since fractions were not weighed individually, the injection mass was used to calculate an average mass per fraction and thus a nominal mass concentration. Fractions were tested at a nominal concentration of 10 μg/mL.

As described in Nitschke et al.^40^ and Mottet et al.,^41^ Dd Ax2­(ka) expressing mCherry from the act5 chromosomal locus^42^ was infected with Mm M strain expressing the lux operon (luxCDABE)^43,44^ by spinoculation.

Briefly, the day before the experiment, Mm was cultivated in 7H9 broth (Becton Dickinson, Difco Middlebrook 7H9) supplemented with 10% OADC (Becton Dickinson) and 0.05% tyloxapol (Sigma-Aldrich) and 50 μg/mL kanamycin at 32 °C overnight with continuous shaking. Additionally, the day before the experiment, 10^7^ amoebae were plated in HL5-C in a 10 cm Petri dish (Falcon). On the day of the experiment, a volume of the Mm culture corresponding to a multiplicity of infection of 25 with respect to the number of amoebae in the Petri dish was added to the semiconfluent amoebae monolayer. Subsequently, the Petri dishes were centrifuged twice at 500*g*, as described in Mottet et al.^41^ To remove extracellular bacteria, dishes were rinsed with fresh HL5-C, and the infected cell population was resuspended in HL5-C with 5 U/mL penicillin and 5 μg/mL streptomycin (Gibco) to inhibit the extracellular growth of bacteria during the course of the experiment.

For testing fractions and purified compounds, 20 μL of infected cell suspension was plated into each well of a 384-well plate (Interchim FP-BA8240) to an effective cell number of 1 × 10^4^ cells per well. Fractions or compounds including a vehicle control (0.3% DMSO final concentration) and a positive control (rifabutin, 10 μM final concentration) were added using an electronic multipipette (Sartorius). Subsequently, the well plates were sealed with a gas impermeable membrane (H769.1, Carl Roth), briefly centrifuged, and intracellular bacterial growth was monitored using an Agilent BioTek H1 plate reader and an Agilent BioTek BioStack plate stacker by recording luminescence over 72 h at 25 °C with readings taken every hour. Fluorescence was also recorded to monitor amoeba growth.

For testing bacteria in broth, the preculture was diluted to a bacterial density of 3.75 × 10^5^ bacteria per mL in 7H9 medium. Plating bacteria and compounds or fractions was performed analogously to the infection assay described above. Bacteria growth was monitored with the Agilent BioTek H1 plate reader by recording luminescence at 32 °C.

For both assays, growth curves were obtained by measuring the luminescence and fluorescence as a proxy for bacterial growth and host growth, respectively, for 72 h with time-points taken every hour. The “normalized residual growth” was computed by calculating the area under the curve (AUC, trapezoid method) and normalizing it to the vehicle control (0.3% DMSO, set at 1) and a baseline curve (set at 0). The baseline curve was calculated by taking the median of the first measurement of all wells in a plate and extrapolating it over the complete time course. The threshold for hit detection was arbitrarily fixed at a cutoff of normalized residual growth ≤0.5. Normalized values were averaged over technical and biological replicates (all experiments on isolated compounds have at least *n* = 3 and *N* = 3, whereas the primary extract screening and microfractions testing had values of *n* = 1 and *N* = 3).

This procedure was also applied to screening extracts with slight modifications. The day before the experiment we preplated 10 μL of HL5-C using a dispenser (Thermo Multidrop), subsequently we preplated 2.2 μL of dissolved extracts from 96-well plates into quadrants 1, 2, and 3 of 384-well plates, whereas quadrant 4 was used for positive and vehicle controls. Preplating of extracts was performed using a liquid handler (Agilent Bravo). In total, 24 extract plates were distributed in triplicate into eight 384-well plates, amounting to 24 assay plates. The prepared plates were sealed and stored at 4 °C overnight. On the day of the experiment, nine 10 cm Petri dishes were infected (as described before) and grouped into three pools. The cell suspension was adjusted to 10^6^ cells/mL, and 10 μL was plated into the 24 assay plates, resulting in 10^4^ cells per well, as used for conventional infection experiments. Plates were sealed and placed in the plate stacker that supplied the plate reader. The same procedure was used to screen the same extracts on Mm in a broth. For both assays, the first time points and a time point after 72 h were recorded. Subsequently, we normalized the end point, first with the median of the full assay plate at the first time point and second with the end point of the vehicle controls in the respective assay plate.

For IC_50_ estimation, we used a rudimentary approach of interpolating the sample concentration between the two normalized residual growth values, which were closest to a value of 0.5.

### Bioactivity Screening (Pa and Sa)

MICs were determined in Mueller–Hinton (MH) broth according to CLSI guidelines^45^ and were repeated at least on three different occasions.

The Newman strain of*S. aureus* ^17^ and the UCBPP-PA14 strain of*P. aeruginosa* ^18^ were used in this study.

## Supplementary Material



## Data Availability

All data relative to the above-mentioned collection of 1600 NEs was described in Allard et al. (2023)^15^ at 10.1093/gigascience/giac124. The raw NMR data of all isolated compounds presented in this study is available through the NP-MRD platform via the NP-MRD IDs provided for each compound in the experimental section. The raw HRMS/MS data of all isolated compounds presented in this study is accessible through the GNPS platform via the spectrum IDs provided for each compound in the experimental section.

## Notes

Raw data associated with the isolated compounds, chromatographic separations, process for the purity estimation, chromatograms of the *K. oblongifolia* extract, description of the NMR-based purity assessment method, purity and bioactivity tables for isolated NPs, and all the raw HRMS and NMR data for all isolated compounds (NPs and synthetic) (PDF)

## References

[1] Aminov R. I. (2010). A Brief
History of the Antibiotic Era: Lessons Learned and Challenges for
the Future. Front. Microbiol..

[2] Banin E., Hughes D., Kuipers O. P. (2017). Editorial: Bacterial
Pathogens, Antibiotics
and Antibiotic Resistance. FEMS Microbiol. Rev..

[3] O’Neill, J. *Tackling Drug-Resistant Infections Globally: Final Report and Recommendations*; Review on Antimicrobial Resistance: United Kingdom. 2016. https://amr-review.org/sites/default/files/160525_Finalpaper_withcover.pdf (accessed Apr 25, 2024).

[4] Geneva: World Health Organisation . Global Tuberculosis Report 2022; Geneva: World Health Organisation, 2022.

[5] Newman D. J., Cragg G. M. (2020). Natural Products
as Sources of New Drugs over the Nearly
Four Decades from 01/1981 to 09/2019. J. Nat.
Prod..

[6] Gonzalez-Pastor R., Carrera-Pacheco S. E., Zúñiga-Miranda J., Rodríguez-Pólit C., Mayorga-Ramos A., Guamán L. P., Barba-Ostria C. (2023). Current Landscape
of Methods to Evaluate
Antimicrobial Activity of Natural Extracts. Molecules.

[7] Xie Y., Feng Y., Di Capua A., Mak T., Buchko G. W., Myler P. J., Liu M., Quinn R. J. (2020). A Phenotarget
Approach
for Identifying an Alkaloid Interacting with the Tuberculosis Protein
Rv1466. Mar. Drugs.

[8] Zheng W., Thorne N., McKew J. C. (2013). Phenotypic Screens as a Renewed Approach
for Drug Discovery. Drug Discovery Today.

[9] Payne D. J., Gwynn M. N., Holmes D. J., Pompliano D. L. (2007). Drugs for
Bad Bugs: Confronting the Challenges of Antibacterial Discovery. Nat. Rev. Drug Discovery.

[10] Brodin P., Poquet Y., Levillain F., Peguillet I., Larrouy-Maumus G., Gilleron M., Ewann F., Christophe T., Fenistein D., Jang J., Jang M.-S., Park S.-J., Rauzier J., Carralot J.-P., Shrimpton R., Genovesio A., Gonzalo-Asensio J. A., Puzo G., Martin C., Brosch R., Stewart G. R., Gicquel B., Neyrolles O. (2010). High Content
Phenotypic Cell-Based Visual Screen Identifies Mycobacterium Tuberculosis
Acyltrehalose-Containing Glycolipids Involved in Phagosome Remodeling. Public Libr. Sci. Pathog..

[11] Kalsum S., Otrocka M., Andersson B., Welin A., Schön T., Jenmalm-Jensen A., Lundbäck T., Lerm M. (2022). A High Content Screening
Assay for Discovery of Antimycobacterial Compounds Based on Primary
Human Macrophages Infected with Virulent Mycobacterium Tuberculosis. Tuberculosis.

[12] Tobin D. M., Ramakrishnan L. (2008). Comparative
Pathogenesis of *Mycobacterium Marinum* and *Mycobacterium Tuberculosis*. Cell.
Microbiol..

[13] Habjan E., Ho V. Q. T., Gallant J., van Stempvoort G., Jim K. K., Kuijl C., Geerke D. P., Bitter W., Speer A. (2021). An Anti-Tuberculosis Compound Screen Using a Zebrafish Infection
Model Identifies an Aspartyl-tRNA Synthetase Inhibitor. Dis. Model. Mech..

[14] Nitschke J., Huber R., Vossio S., Moreau D., Marcourt L., Gindro K., Queiroz E. F., Soldati T., Hanna N. (2024). Discovery
of Anti-Infective Compounds against Mycobacterium Marinum after Biotransformation
of Simple Natural Stilbene Scaffolds by a Fungal Secretome. bioRxiv.

[15] Allard P.-M., Gaudry A., Quirós-Guerrero L.-M., Rutz A., Dounoue-Kubo M., Walker T. W. N., Defossez E., Long C., Grondin A., David B., Wolfender J.-L. (2022). Open and
Reusable Annotated Mass Spectrometry Dataset of a Chemodiverse Collection
of 1,600 Plant Extracts. GigaScience.

[16] Nitschke, J. The Dictyostelium Discoideum - Mycobacterium Marinum Platform in Natural Product Drug Discovery; Université de Genève, 2024; . 10.13097/archive-ouverte/unige:179581.

[17] Duthie E. S., Lorenz L. L. (1952). Staphylococcal Coagulase:
Mode of Action and Antigenicity. Microbiology.

[18] Rahme L. G., Stevens E. J., Wolfort S. F., Shao J., Tompkins R. G., Ausubel F. M. (1995). Common Virulence
Factors for Bacterial Pathogenicity
in Plants and Animals. Science.

[19] Grace A., Sahu R., Owen D. R., Dennis V. A. (2022). *Pseudomonas
Aeruginosa* Reference Strains PAO1 and PA14: A Genomic, Phenotypic,
and Therapeutic Review. Front. Microbiol..

[20] Kirchhoffer O. A., Nitschke J., Allard P.-M., Marcourt L., David B., Grondin A., Hanna N., Queiroz E. F., Soldati T., Wolfender J.-L. (2023). Targeted Isolation of Natural Analogs
of Anti-Mycobacterial
Hit Compounds Based on the Metabolite Profiling of a Large Collection
of Plant Extracts. Front. Nat. Prod.

[21] Hamburger M. (2019). HPLC-Based
Activity Profiling for Pharmacologically and Toxicologically Relevant
Natural Products – Principles and Recent Examples. Pharm. Biol..

[22] Dührkop K., Fleischauer M., Ludwig M., Aksenov A. A., Melnik A. V., Meusel M., Dorrestein P. C., Rousu J., Böcker S. (2019). SIRIUS 4:
A Rapid Tool for Turning Tandem Mass Spectra into Metabolite Structure
Information. Nat. Methods.

[23] Dührkop K., Shen H., Meusel M., Rousu J., Böcker S. (2015). Searching
Molecular Structure Databases with Tandem Mass Spectra Using CSI:FingerID. Proc. Natl. Acad. Sci. U.S.A..

[24] Sriphana U., Yenjai C., Koatthada M. (2016). Cytotoxicity
of Chemical Constituents
from the Roots of *Knema Globularia*. Phytochem. Lett..

[25] Gény C., Rivière G., Bignon J., Birlirakis N., Guittet E., Awang K., Litaudon M., Roussi F., Dumontet V. (2016). Anacardic Acids from *Knema Hookeriana* as Modulators of Bcl-xL/Bak and Mcl-1/Bid
Interactions. J. Nat. Prod..

[26] Guillarme D., Nguyen D. T. T., Rudaz S., Veuthey J.-L. (2008). Method Transfer
for Fast Liquid Chromatography in Pharmaceutical Analysis: Application
to Short Columns Packed with Small Particle. Part II: Gradient Experiments. Eur. J. Pharm. Biopharm..

[27] Sharma, N. K. ; Sharma, V. N. Structure of Anagigantic Acid Isolated from *Anacardium Giganteum* . Indian J. Chem. 1966, 4, 504.

[28] Kazlauskas R., Mulder J., Murphy P., Wells R. (1980). New Metabolites from
the Brown Alga Caulocystis Cephalornithos. Aust.
J. Chem..

[29] Hemshekhar M., Sebastin
Santhosh M., Kemparaju K., Girish K. S. (2012). Emerging Roles of
Anacardic Acid and Its Derivatives: A Pharmacological Overview. Basic Clin. Pharmacol. Toxicol..

[30] Muroi H., Kubo I. (1996). Antibacterial Activity
of Anacardic Acid and Totarol, Alone and in
Combination with Methicillin, against Methicillinresistant *Staphylococcus Aureus*. J. Appl. Bacteriol..

[31] Faleye O. S., Boya B. R., Lee J.-H., Choi I., Lee J. (2024). Halogenated
Antimicrobial Agents to Combat Drug-Resistant Pathogens. Pharmacol. Rev..

[32] Neuenschwander A., Rocha V. P. C., Bastos T. M., Marcourt L., Morin H., da Rocha C. Q., Grimaldi G. B., de Sousa K. A. F., Borges J. N., Rivara-Minten E., Wolfender J.-L., Soares M. B. P., Queiroz E. F. (2020). Production
of Highly Active Antiparasitic Compounds from the Controlled Halogenation
of the *Arrabidaea brachypoda* Crude
Plant Extract. J. Nat. Prod..

[33] Huber R., Marcourt L., Héritier M., Luscher A., Guebey L., Schnee S., Michellod E., Guerrier S., Wolfender J.-L., Scapozza L., Köhler T., Gindro K., Queiroz E. F. (2023). Generation
of Potent Antibacterial Compounds through Enzymatic and Chemical Modifications
of the Trans-δ-Viniferin Scaffold. Sci.
Rep..

[34] Wilcken R., Zimmermann M. O., Lange A., Joerger A. C., Boeckler F. M. (2013). Principles
and Applications of Halogen Bonding in Medicinal Chemistry and Chemical
Biology. J. Med. Chem..

[35] Wu Y.-Q., Lu H.-J., Zhao W.-T., Zhao H.-Y., Lin Z.-Y., Zhang D.-F., Huang H.-H. (2020). A Convenient and Efficient H2SO4-Promoted
Regioselective Monobromination of Phenol Derivatives Using N-Bromosuccinimide. Synth. Commun..

[36] Devakaram R., Black D. StC., Andrews K. T., Fisher G. M., Davis R. A., Kumar N. (2011). Synthesis and Antimalarial Evaluation
of Novel Benzopyrano­[4,3-b]­Benzopyran
Derivatives. Bioorg. Med. Chem..

[37] Pérez P., Domingo L. R., Duque-Noreña M., Chamorro E. (2009). A Condensed-to-Atom
Nucleophilicity Index. An Application to the Director Effects on the
Electrophilic Aromatic Substitutions. J. Mol.
Struct. THEOCHEM.

[38] Saedtler M., Förtig N., Ohlsen K., Faber F., Masota N., Kowalick K., Holzgrabe U., Meinel L. (2020). Antibacterial Anacardic
Acid Derivatives. ACS Infect. Dis..

[39] Hamad F. B., Mubofu E. B. (2015). Potential Biological
Applications of Bio-Based Anacardic
Acids and Their Derivatives. Int. J. Mol. Sci..

[40] Nitschke J., Huber R., Vossio S., Moreau D., Marcourt L., Gindro K., Queiroz E. F., Soldati T., Hanna N. (2024). Discovery
of Anti-Infective Compounds against *Mycobacterium Marinum* after Biotransformation of Simple Natural Stilbenes by a Fungal
Secretome. Front. Microbiol..

[41] Mottet, M. ; Bosmani, C. ; Hanna, N. ; Nitschke, J. ; Lefrançois, L. H. ; Soldati, T. Novel Single-Cell and High-Throughput Microscopy Techniques to Monitor *Dictyostelium Discoideum*–*Mycobacterium Marinum* (*M. Marinum*) Infection Dynamics. In Mycobacteria, Protocols. Methods in Molecular Biology; Parish, T. , Kumar, A. , Eds.; Springer US: New York, NY, 2021; pp 183–203. 10.1007/978-1-0716-1460-0_7. 34235653

[42] Paschke P., Knecht D. A., Williams T. D., Thomason P. A., Insall R. H., Chubb J. R., Kay R. R., Veltman D. M. (2019). Genetic
Engineering
of *Dictyostelium Discoideum* Cells Based on Selection
and Growth on Bacteria. J. Vis. Exp..

[43] Andreu N., Zelmer A., Fletcher T., Elkington P. T., Ward T. H., Ripoll J., Parish T., Bancroft G. J., Schaible U., Robertson B. D., Wiles S. (2010). Optimisation of Bioluminescent
Reporters for Use with Mycobacteria. PLoS One.

[44] Arafah, S. ; Kicka, S. ; Trofimov, V. ; Hagedorn, M. ; Andreu, N. ; Wiles, S. ; Robertson, B. ; Soldati, T. Setting Up and Monitoring an Infection of *Dictyostelium Discoideum* with Mycobacteria. In Dictyostelium discoideum Protocols; Eichinger, L. , Rivero, F. , Eds.; Humana Press: Totowa, NJ, 2013; pp 403–417. 10.1007/978-1-62703-302-2_22. 23494320

[45] Weinstein, M. P. ; Patel, J. B. Methods for Dilution Antimicrobial Susceptibility Tests for Bacteria that Grow Aerobically: M07-A11; Documents/Clinical and Laboratory Standards Institute; Committee for Clinical Laboratory Standards: Wayne, PA, 2018.

