# Supplementary material for: Phenylalkyl Acetophenones and Anacardic Acids from Knema oblongifolia with Synthetic Analogues as Anti-infectives and Antibacterial Agents
Source: ACS Bio Med Chem Au. 2025 Jun 9;5(4):650–64. doi: 10.1021/acsbiomedchemau.5c00052 (PMC12371487; doi:10.1021/acsbiomedchemau.5c00052)
Supplement: Supplementary file 1 [file bg5c00052_si_001.pdf]

Supplementary Material

Phenylalkyl acetophenones and anacardic acids from *Knema oblongifolia* with synthetic analogues as anti-infectives and anti-bacterial agents

Olivier A. Kirchhoffer[1,2], Jahn Nitschke[3], Alexandre Luscher[4], Louis-Félix Nothias[1,2,5], Laurence Marcourt[1,2], Nabil Hanna[3], Antonio Grondin[6], Thilo Köhler[4], Emerson F. Queiroz[1,2*], Thierry Soldati[3] and Jean-Luc Wolfender[1,2*].

[1]*Institute of Pharmaceutical Sciences of Western Switzerland, University of Geneva, CMU, 1211 Geneva, Switzerland.*

[2]*School of Pharmaceutical Sciences, University of Geneva, CMU, 1211 Geneva, Switzerland.*

[3]*Department of Biochemistry, Faculty of Sciences, University of Geneva, Quai Ernest-Ansermet 30, 1205 Geneva, Switzerland.*

[4]*Department of Microbiology and Molecular Medicine, University of Geneva, CMU, 1211 Geneva, Switzerland.*

[5]*Université Côte d'Azur, CNRS, ICN, 06103 Nice, France.*

[6]*Green Mission Department, Herbal Products Laboratory, Pierre Fabre Research Institute, 31100 Toulouse, France.*

***Corresponding Authors:**

Jean-Luc Wolfender - orcid.org/0000-0002-0125-952X; Email: jean-luc.wolfender@unige.ch; Emerson Ferreira Queiroz - orcid.org/0000-0001-9567-1664; Email: emerson.ferreira@unige.ch; Institute of Pharmaceutical Sciences of Western Switzerland, School of Pharmaceutical Sciences, University of Geneva, CMU, 1211 Geneva, Switzerland.

Table of content

Supplementary Figures 11
Supplementary Tables 13
Supplementary Data: NMR and HRMS 15
Natural Products from *Knema oblongifolia* (leaves) 16
1. Knemolone A: 16
HRESIMS- spectrum of **Knemolone A** in MeOH. 17
HRESIMS+ spectrum of **Knemolone A** in MeOH. 17
^{1}H NMR spectrum of **Knemolone A** in $CDCl_3$ at 600 MHz. 18
^{13}C DEPTQ NMR spectrum of **Knemolone A** in $CDCl_3$ at 151 MHz. 18
HSQC NMR spectrum of **Knemolone A** in $CDCl_3$ at 600 MHz. 19
HMBC NMR spectrum of **Knemolone A** in $CDCl_3$ at 600 MHz. 19
COSY NMR spectrum of **Knemolone A** in $CDCl_3$ at 600 MHz. 20
ROESY NMR spectrum of **Knemolone A** in $CDCl_3$ at 600 MHz. 20
2. Knemolone B: 21
HRESIMS- spectrum of **Knemolone B** in MeOH. 21
HRESIMS+ spectrum of **Knemolone B** in MeOH. 22
^{1}H NMR spectrum of **Knemolone B** in $CDCl_3$ at 600 MHz. 22
^{13}C DEPTQ NMR spectrum of **Knemolone B** in $CDCl_3$ at 151 MHz. 23
HSQC NMR spectrum of **Knemolone B** in $CDCl_3$ at 600 MHz. 23
HMBC NMR spectrum of **Knemolone B** in $CDCl_3$ at 600 MHz. 24
ROESY NMR spectrum of **Knemolone B** in $CDCl_3$ at 600 MHz. 25
3. Knemolic acid A: 26
HRESIMS- spectrum of **Knemolic acid A** in MeOH. 26
HRESIMS/MS+ fragmentation spectrum of **Knemolic acid A** in MeOH. 27
^{1}H NMR spectrum of **Knemolic acid A** in $CDCl_3$ at 600 MHz. 27
^{13}C DEPTQ NMR spectrum of **Knemolic acid A** in $CDCl_3$ at 151 MHz. 28
HSQC NMR spectrum of **Knemolic acid A** in $CDCl_3$ at 600 MHz. 28
COSY NMR spectrum of **Knemolic acid A** in $CDCl_3$ at 600 MHz. 30
ROESY NMR spectrum of **Knemolic acid A** in $CDCl_3$ at 600 MHz. 30
4. Knemolone C: 31
HRESIMS- spectrum of **Knemolone C** in MeOH. 31
HRESIMS+ spectrum of **Knemolone C** in MeOH. 32
^{1}H NMR spectrum of **Knemolone C** in $CDCl_3$ at 600 MHz. 32
^{13}C DEPTQ NMR spectrum of **Knemolone C** in $CDCl_3$ at 151 MHz. 33

HMBC NMR spectrum of **Knemolone C** in $CDCl_3$ at 600 MHz. 34
COSY NMR spectrum of **Knemolone C** in $CDCl_3$ at 600 MHz. 34
ROESY NMR spectrum of **Knemolone C** in $CDCl_3$ at 600 MHz. 35
5. Knemolone D: 36
HRESIMS- spectrum of **Knemolone D** in MeOH. 36
HRESIMS+ spectrum of **Knemolone D** in MeOH. 37
^{1}H NMR spectrum of **Knemolone D** in $CDCl_3$ at 600 MHz. 37
^{13}C DEPTQ NMR spectrum of **Knemolone D** in $CDCl_3$ at 151 MHz. 38
HSQC NMR spectrum of **Knemolone D** in $CDCl_3$ at 600 MHz. 38
HMBC NMR spectrum of **Knemolone D** in $CDCl_3$ at 600 MHz. 39
ROESY NMR spectrum of **Knemolone D** in $CDCl_3$ at 600 MHz. 40
6. Knemolic acid B: 41
HRESIMS- spectrum of **Knemolic acid B** in MeOH. 41
HRESIMS+ spectrum of **Knemolic acid B** in MeOH. 42
^{1}H NMR spectrum of **Knemolic acid B** in $CDCl_3$ at 600 MHz. 42
^{13}C DEPTQ NMR spectrum of **Knemolic acid B** in $CDCl_3$ at 151 MHz. 43
HSQC NMR spectrum of **Knemolic acid B** in $CDCl_3$ at 600 MHz. 43
HMBC NMR spectrum of **Knemolic acid B** in $CDCl_3$ at 600 MHz. 44
COSY NMR spectrum of **Knemolic acid B** in $CDCl_3$ at 600 MHz. 44
ROESY NMR spectrum of **Knemolic acid B** in $CDCl_3$ at 600 MHz. 45
7. Knemolic acid C: 46
HRESIMS- spectrum of **Knemolic acid C** in MeOH. 46
HRESIMS+ spectrum of **Knemolic acid C** in MeOH. 47
^{1}H NMR spectrum of **Knemolic acid C** in $CDCl_3$ at 600 MHz. 47
^{13}C DEPTQ NMR spectrum of **Knemolic acid C** in $CDCl_3$ at 151 MHz. 48
HSQC NMR spectrum of **Knemolic acid C** in $CDCl_3$ at 600 MHz. 48
HMBC NMR spectrum of **Knemolic acid C** in $CDCl_3$ at 600 MHz. 49
COSY NMR spectrum of **Knemolic acid C** in $CDCl_3$ at 600 MHz. 49
ROESY NMR spectrum of **Knemolic acid C** in $CDCl_3$ at 600 MHz. 50
8. Khookerianic acid A: 51
HRESIMS- spectrum of **Khookerianic acid A** in MeOH. 51
HRESIMS+ spectrum of **Khookerianic acid A** in MeOH. 52
^{1}H NMR spectrum of **Khookerianic acid A** in $CDCl_3$ at 600 MHz. 52
^{13}C DEPTQ NMR spectrum of **Khookerianic acid A** in $CDCl_3$ at 151 MHz. 53
HSQC NMR spectrum of **Khookerianic acid A** in $CDCl_3$ at 600 MHz. 53

HMBC NMR spectrum of **Khookerianic acid A** in $CDCl_3$ at 600 MHz. 54
COSY NMR spectrum of **Khookerianic acid A** in $CDCl_3$ at 600 MHz.........................54
ROESY NMR spectrum of **Khookerianic acid A** in $CDCl_3$ at 600 MHz. 55
9. Kneglobularic acid B: 56
HRESIMS- spectrum of **Kneglobularic acid B** in MeOH..56
HRESIMS+ spectrum of **Kneglobularic acid B** in MeOH...57
^{1}H NMR spectrum of **Kneglobularic acid B** in $CDCl_3$ at 600 MHz.............................57
^{13}C DEPTQ NMR spectrum of **Kneglobularic acid B** in $CDCl_3$ at 151 MHz. 58
HSQC NMR spectrum of **Kneglobularic acid B** in $CDCl_3$ at 600 MHz.......................58
HMBC NMR spectrum of **Kneglobularic acid B** in $CDCl_3$ at 600 MHz.......................59
COSY NMR spectrum of **Kneglobularic acid B** in $CDCl_3$ at 600 MHz.......................59
ROESY NMR spectrum of **Kneglobularic acid B** in $CDCl_3$ at 600 MHz. 60
10. Khookerianic acid C/Kneglobularic acid A: 61
HRESIMS+ spectrum of **Khookerianic acid C/Kneglobularic acid A** in MeOH. 61
HRESIMS- spectrum of **Khookerianic acid C/Kneglobularic acid A** in MeOH. 62
^{1}H NMR spectrum of **Khookerianic acid C/Kneglobularic acid A** in $CDCl_3$ at 600 MHz. 62
^{13}C DEPTQ NMR spectrum of **Khookerianic acid C/Kneglobularic acid A** in $CDCl_3$ at 151 MHz..63
HSQC NMR spectrum of **Khookerianic acid C/Kneglobularic acid A** in $CDCl_3$ at 600 MHz. 63
HMBC NMR spectrum of **Khookerianic acid C/Kneglobularic acid A** in $CDCl_3$ at 600 MHz. 64
COSY NMR spectrum of **Khookerianic acid C/Kneglobularic acid A** in $CDCl_3$ at 600 MHz. 64
ROESY NMR spectrum of **Khookerianic acid C/Kneglobularic acid A** in $CDCl_3$ at 600 MHz..65
11. Anagigantic acid: 66
HRESIMS- spectrum of **Anagigantic acid** in MeOH..66
HRESIMS+ spectrum of **Anagigantic acid** in MeOH...67
^{1}H NMR spectrum of **Anagigantic acid** in $CDCl_3$ at 600 MHz....................................67
^{13}C DEPTQ NMR spectrum of **Anagigantic acid** in $CDCl_3$ at 151 MHz.......................68
HSQC NMR spectrum of **Anagigantic acid** in $CDCl_3$ at 600 MHz..............................68
COSY NMR spectrum of **Anagigantic acid** in $CDCl_3$ at 600 MHz..............................69
ROESY NMR spectrum of **Anagigantic acid** in $CDCl_3$ at 600 MHz. 70
12. 6-Tridecylsalicylic acid: 71
HRESIMS- spectrum of **6-Tridecylsalicylic acid** in MeOH...71

HRESIMS+ spectrum of **6-Tridecylsalicylic acid** in MeOH..72
^{1}H NMR spectrum of **6-Tridecylsalicylic acid** in $CDCl_3$ at 600 MHz...........................72
^{13}C DEPTQ NMR spectrum of **6-Tridecylsalicylic acid** in $CDCl_3$ at 151 MHz. 73
HSQC NMR spectrum of **6-Tridecylsalicylic acid** in $CDCl_3$ at 600 MHz.....................73
HMBC NMR spectrum of **6-Tridecylsalicylic acid** in $CDCl_3$ at 600 MHz....................74
COSY NMR spectrum of **6-Tridecylsalicylic acid** in $CDCl_3$ at 600 MHz.....................74
ROESY NMR spectrum of **6-Tridecylsalicylic acid** in $CDCl_3$ at 600 MHz. 75
Synthetic derivatives of 2,4-dihydroxyacetophenone 76
13. 2,4-dihydroxyacetophenone: 77
HRESIMS+ spectrum of **2,4-dihydroxyacetophenone** in MeOH. 77
HRESIMS- spectrum of **2,4-dihydroxyacetophenone** in MeOH. 78
^{1}H NMR spectrum of **2,4-dihydroxyacetophenone** in DMSO-d_6 at 600 MHz. 78
^{13}C DEPTQ NMR spectrum of **2,4-dihydroxyacetophenone** in DMSO-d_6 at 151 MHz. 79
HSQC NMR spectrum of **2,4-dihydroxyacetophenone** in DMSO-d_6 at 600 MHz........79
HMBC NMR spectrum of **2,4-dihydroxyacetophenone** in DMSO-d_6 at 600 MHz.......80
COSY NMR spectrum of **2,4-dihydroxyacetophenone** in DMSO-d_6 at 600 MHz........80
ROESY NMR spectrum of **2,4-dihydroxyacetophenone** in DMSO-d_6 at 600 MHz. 81
14. 2,4-dihydroxychalcone:..82
HRESIMS+ spectrum of **2,4-dihydroxychalcone** in MeOH. 82
HRESIMS- spectrum of **2,4-dihydroxychalcone** in MeOH. 83
^{1}H NMR spectrum of **2,4-dihydroxychalcone** in DMSO-d_6 at 600 MHz. 83
^{13}C DEPTQ NMR spectrum of **2,4-dihydroxychalcone** in DMSO-d_6 at 151 MHz........84
HSQC NMR spectrum of **2,4-dihydroxychalcone** in DMSO-d_6 at 600 MHz. 84
HMBC NMR spectrum of **2,4-dihydroxychalcone** in DMSO-d_6 at 600 MHz. 85
COSY NMR spectrum of **2,4-dihydroxychalcone** in DMSO-d_6 at 600 MHz. 85
ROESY NMR spectrum of **2,4-dihydroxychalcone** in DMSO-d_6 at 600 MHz..............86
15. 3-chloro-2,4-dihydroxyacetophenone: 87
HRESIMS+ spectrum of **3-chloro-2,4-dihydroxyacetophenone** in MeOH. 87
HRESIMS- spectrum of **3-chloro-2,4-dihydroxyacetophenone** in MeOH. 88
^{1}H NMR spectrum of **3-chloro-2,4-dihydroxyacetophenone** in DMSO-d_6 at 600 MHz. 88
^{13}C DEPTQ NMR spectrum of **3-chloro-2,4-dihydroxyacetophenone** in DMSO-d_6 at 151 MHz..89
HSQC NMR spectrum of **3-chloro-2,4-dihydroxyacetophenone** in DMSO-d_6 at 600 MHz. 89

HMBC NMR spectrum of **3-chloro-2,4-dihydroxyacetophenone** in DMSO-d_6 at 600 MHz. 90

16. 5-chloro-2,4-dihydroxyacetophenone: 91

HRESIMS+ spectrum of **5-chloro-2,4-dihydroxyacetophenone** in MeOH. 91

HRESIMS+ spectrum of **5-chloro-2,4-dihydroxyacetophenone** in MeOH. 92

^{1}H NMR spectrum of **5-chloro-2,4-dihydroxyacetophenone** in DMSO-d_6 at 600 MHz. 92

^{13}C DEPTQ NMR spectrum of **5-chloro-2,4-dihydroxyacetophenone** in DMSO-d_6 at 151 MHz. 93

HSQC NMR spectrum of **5-chloro-2,4-dihydroxyacetophenone** in DMSO-d_6 at 600 MHz. 93

HMBC NMR spectrum of **5-chloro-2,4-dihydroxyacetophenone** in DMSO-d_6 at 600 MHz. 94

17. 3,5-di-chloro-2,4-dihydroxyacetophenone: 95

HRESIMS+ spectrum of **3,5-di-chloro-2,4-dihydroxyacetophenone** in MeOH. 95

HRESIMS- spectrum of **3,5-di-chloro-2,4-dihydroxyacetophenone** in MeOH. 96

^{1}H NMR spectrum of **3,5-di-chloro-2,4-dihydroxyacetophenone** in DMSO-d_6 at 600 MHz. 96

^{13}C DEPTQ NMR spectrum of **3,5-di-chloro-2,4-dihydroxyacetophenone** in DMSO-d_6 at 151 MHz. 97

HSQC NMR spectrum of **3,5-di-chloro-2,4-dihydroxyacetophenone** in DMSO-d_6 at 600 MHz. 97

HMBC NMR spectrum of **3,5-di-chloro-2,4-dihydroxyacetophenone** in DMSO-d_6 at 600 MHz. 98

18. 3-bromo-2,4-dihydroxyacetophenone: 99

HRESIMS+ spectrum of **3-bromo-2,4-dihydroxyacetophenone** in MeOH. 99

HRESIMS- spectrum of **3-bromo-2,4-dihydroxyacetophenone** in MeOH. 100

^{1}H NMR spectrum of **3-bromo-2,4-dihydroxyacetophenone** in DMSO-d_6 at 600 MHz. 100

^{13}C DEPTQ NMR spectrum of **3-bromo-2,4-dihydroxyacetophenone** in DMSO-d_6 at 151 MHz. 101

HSQC NMR spectrum of **3-bromo-2,4-dihydroxyacetophenone** in DMSO-d_6 at 600 MHz. 101

HMBC NMR spectrum of **3-bromo-2,4-dihydroxyacetophenone** in DMSO-d_6 at 600 MHz. 102

COSY NMR spectrum of **3-bromo-2,4-dihydroxyacetophenone** in DMSO-d_6 at 600 MHz. 102

ROESY NMR spectrum of **3-bromo-2,4-dihydroxyacetophenone** in DMSO-d_6 at 600 MHz. 103

19. 5-bromo-2,4-dihydroxyacetophenone: 104

HRESIMS+ spectrum of **5-bromo-2,4-dihydroxyacetophenone** in MeOH. 104
HRESIMS- spectrum of **5-bromo-2,4-dihydroxyacetophenone** in MeOH. 105
^{1}H NMR spectrum of **5-bromo-2,4-dihydroxyacetophenone** in DMSO-d_6 at 600 MHz. 105
^{13}C DEPTQ NMR spectrum of **5-bromo-2,4-dihydroxyacetophenone** in DMSO-d_6 at 151 MHz...106
HSQC NMR spectrum of **5-bromo-2,4-dihydroxyacetophenone** in DMSO-d_6 at 600 MHz. 106
HMBC NMR spectrum of **5-bromo-2,4-dihydroxyacetophenone** in DMSO-d_6 at 600 MHz. 107
COSY NMR spectrum of **5-bromo-2,4-dihydroxyacetophenone** in DMSO-d_6 at 600 MHz. 107
ROESY NMR spectrum of **5-bromo-2,4-dihydroxyacetophenone** in DMSO-d_6 at 600 MHz. 108
20. 3,5-di-bromo-2,4-dihydroxyacetophenone:..109
HRESIMS+ spectrum of **3,5-di-bromo-2,4-dihydroxyacetophenone** in MeOH. 109
HRESIMS- spectrum of **3,5-di-bromo-2,4-dihydroxyacetophenone** in MeOH. 110
^{1}H NMR spectrum of **3,5-di-bromo-2,4-dihydroxyacetophenone** in DMSO-d_6 at 600 MHz. 110
^{13}C DEPTQ NMR spectrum of **3,5-di-bromo-2,4-dihydroxyacetophenone** in DMSO-d_6 at 151 MHz...111
HMBC NMR spectrum of **3,5-di-bromo-2,4-dihydroxyacetophenone** in DMSO-d_6 at 600 MHz...112
ROESY NMR spectrum of **3,5-di-bromo-2,4-dihydroxyacetophenone** in DMSO-d_6 at 600 MHz...113
21. 3-chloro-2,4-dihydroxychalcone:..114
HRESIMS+ spectrum of **3-chloro-2,4-dihydroxychalcone** in MeOH. 114
HRESIMS- spectrum of **3-chloro-2,4-dihydroxychalcone** in MeOH. 115
^{1}H NMR spectrum of **3-chloro-2,4-dihydroxychalcone** in DMSO-d_6 at 600 MHz. 115
^{13}C DEPTQ NMR spectrum of **3-chloro-2,4-dihydroxychalcone** in DMSO-d_6 at 151 MHz. 116
HSQC NMR spectrum of **3-chloro-2,4-dihydroxychalcone** in DMSO-d_6 at 600 MHz. 116
HMBC NMR spectrum of **3-chloro-2,4-dihydroxychalcone** in DMSO-d_6 at 600 MHz. 117
22. 5-chloro-2,4-dihydroxychalcone:..118
HRESIMS+ spectrum of **5-chloro-2,4-dihydroxychalcone** in MeOH. 118
HRESIMS- spectrum of **5-chloro-2,4-dihydroxychalcone** in MeOH. 119
^{1}H NMR spectrum of **5-chloro-2,4-dihydroxychalcone** in DMSO-d_6 at 600 MHz. 119

^{13}C DEPTQ NMR spectrum of **5-chloro-2,4-dihydroxychalcone** in DMSO-d_6 at 151 MHz. 120

HSQC NMR spectrum of **5-chloro-2,4-dihydroxychalcone** in DMSO-d_6 at 600 MHz. 120

HMBC NMR spectrum of **5-chloro-2,4-dihydroxychalcone** in DMSO-d_6 at 600 MHz. 121

23. 3,5-di-chloro-2,4-dihydroxychalcone: 122

HRESIMS+ spectrum of **3,5-di-chloro-2,4-dihydroxychalcone** in MeOH. 122

HRESIMS- spectrum of **3,5-di-chloro-2,4-dihydroxychalcone** in MeOH. 123

^{1}H NMR spectrum of **3,5-di-chloro-2,4-dihydroxychalcone** in DMSO-d_6 at 600 MHz. 123

^{13}C DEPTQ NMR spectrum of **3,5-di-chloro-2,4-dihydroxychalcone** in DMSO-d_6 at 151 MHz. 124

HSQC NMR spectrum of **3,5-di-chloro-2,4-dihydroxychalcone** in DMSO-d_6 at 600 MHz. 124

HMBC NMR spectrum of **3,5-di-chloro-2,4-dihydroxychalcone** in DMSO-d_6 at 600 MHz. 125

24. 3-bromo-2,4-dihydroxychalcone: 126

HRESIMS+ spectrum of **3-bromo-2,4-dihydroxychalcone** in MeOH. 126

HRESIMS- spectrum of **3-bromo-2,4-dihydroxychalcone** in MeOH. 127

^{1}H NMR spectrum of **3-bromo-2,4-dihydroxychalcone** in DMSO-d_6 at 600 MHz. 127

^{13}C DEPTQ NMR spectrum of **3-bromo-2,4-dihydroxychalcone** in DMSO-d_6 at 151 MHz. 128

HSQC NMR spectrum of **3-bromo-2,4-dihydroxychalcone** in DMSO-d_6 at 600 MHz. 128

HMBC NMR spectrum of **3-bromo-2,4-dihydroxychalcone** in DMSO-d_6 at 600 MHz. 129

COSY NMR spectrum of **3-bromo-2,4-dihydroxychalcone** in DMSO-d_6 at 600 MHz. 129

ROESY NMR spectrum of **3-bromo-2,4-dihydroxychalcone** in DMSO-d_6 at 600 MHz. 130

25. 5-bromo-2,4-dihydroxychalcone: 131

HRESIMS+ spectrum of **5-bromo-2,4-dihydroxychalcone** in MeOH. 131

HRESIMS- spectrum of **5-bromo-2,4-dihydroxychalcone** in MeOH. 132

^{1}H NMR spectrum of **5-bromo-2,4-dihydroxychalcone** in DMSO-d_6 at 600 MHz. 132

^{13}C DEPTQ NMR spectrum of **5-bromo-2,4-dihydroxychalcone** in DMSO-d_6 at 151 MHz. 133

HSQC NMR spectrum of **5-bromo-2,4-dihydroxychalcone** in DMSO-d_6 at 600 MHz. 133

HMBC NMR spectrum of **5-bromo-2,4-dihydroxychalcone** in DMSO-d_6 at 600 MHz. 134
COSY NMR spectrum of **5-bromo-2,4-dihydroxychalcone** in DMSO-d_6 at 600 MHz. 134
ROESY NMR spectrum of **5-bromo-2,4-dihydroxychalcone** in DMSO-d_6 at 600 MHz. 135
26. (E)-1-(5-chloro-2,4-dihydroxyphenyl)-3-(naphthalen-1-yl)prop-2-en-1-one: 136
HRESIMS+ spectrum of **(E)-1-(5-chloro-2,4-dihydroxyphenyl)-3-(naphthalen-1-yl)prop-2-en-1-one** in MeOH. 136
HRESIMS- spectrum of **(E)-1-(5-chloro-2,4-dihydroxyphenyl)-3-(naphthalen-1-yl)prop-2-en-1-one** in MeOH. 137
^{1}H NMR spectrum of **(E)-1-(5-chloro-2,4-dihydroxyphenyl)-3-(naphthalen-1-yl)prop-2-en-1-one** in DMSO-d_6 at 600 MHz. 137
^{13}C DEPTQ NMR spectrum of **(E)-1-(5-chloro-2,4-dihydroxyphenyl)-3-(naphthalen-1-yl)prop-2-en-1-one** in DMSO-d_6 at 151 MHz. 138
HSQC NMR spectrum of **(E)-1-(5-chloro-2,4-dihydroxyphenyl)-3-(naphthalen-1-yl)prop-2-en-1-one** in DMSO-d_6 at 600 MHz........138
HMBC NMR spectrum of **(E)-1-(5-chloro-2,4-dihydroxyphenyl)-3-(naphthalen-1-yl)prop-2-en-1-one** in DMSO-d_6 at 600 MHz........139
COSY NMR spectrum of **(E)-1-(5-chloro-2,4-dihydroxyphenyl)-3-(naphthalen-1-yl)prop-2-en-1-one** in DMSO-d_6 at 600 MHz........139
ROESY NMR spectrum of **(E)-1-(5-chloro-2,4-dihydroxyphenyl)-3-(naphthalen-1-yl)prop-2-en-1-one** in DMSO-d_6 at 600 MHz........140
27. (E)-3-(anthracen-9-yl)-1-(5-chloro-2,4-dihydroxyphenyl)prop-2-en-1-one: 141
HRESIMS+ spectrum of **(E)-3-(anthracen-9-yl)-1-(5-chloro-2,4-dihydroxyphenyl)prop-2-en-1-one** in MeOH. 141
HRESIMS- spectrum of **(E)-3-(anthracen-9-yl)-1-(5-chloro-2,4-dihydroxyphenyl)prop-2-en-1-one** in MeOH. 142
^{1}H NMR spectrum of **(E)-3-(anthracen-9-yl)-1-(5-chloro-2,4-dihydroxyphenyl)prop-2-en-1-one** in DMSO-d_6 at 600 MHz. 142
^{13}C DEPTQ NMR spectrum of **(E)-3-(anthracen-9-yl)-1-(5-chloro-2,4-dihydroxyphenyl)prop-2-en-1-one** in DMSO-d_6 at 151 MHz........143
HSQC NMR spectrum of **(E)-3-(anthracen-9-yl)-1-(5-chloro-2,4-dihydroxyphenyl)prop-2-en-1-one** in DMSO-d_6 at 600 MHz........143
HMBC NMR spectrum of **(E)-3-(anthracen-9-yl)-1-(5-chloro-2,4-dihydroxyphenyl)prop-2-en-1-one** in DMSO-d_6 at 600 MHz........144
COSY NMR spectrum of **(E)-3-(anthracen-9-yl)-1-(5-chloro-2,4-dihydroxyphenyl)prop-2-en-1-one** in DMSO-d_6 at 600 MHz........144
ROESY NMR spectrum of **(E)-3-(anthracen-9-yl)-1-(5-chloro-2,4-dihydroxyphenyl)prop-2-en-1-one** in DMSO-d_6 at 600 MHz........145
28. 1-(3-chloro-4,6-dihydroxy-2-(6-phenylhexyl)phenyl)ethan-1-one: 146

HRESIMS+ spectrum of **1-(3-chloro-4,6-dihydroxy-2-(6-phenylhexyl)phenyl)ethan-1-one** in MeOH. 146

HRESIMS- spectrum of **1-(3-chloro-4,6-dihydroxy-2-(6-phenylhexyl)phenyl)ethan-1-one** in MeOH. 147

^{1}H NMR spectrum of **1-(3-chloro-4,6-dihydroxy-2-(6-phenylhexyl)phenyl)ethan-1-one** in DMSO-d_6 at 600 MHz. 147

^{13}C DEPTQ NMR spectrum of **1-(3-chloro-4,6-dihydroxy-2-(6-phenylhexyl)phenyl)ethan-1-one** in DMSO-d_6 at 151 MHz. 148

HSQC NMR spectrum of **1-(3-chloro-4,6-dihydroxy-2-(6-phenylhexyl)phenyl)ethan-1-one** in DMSO-d_6 at 600 MHz. 148

HMBC NMR spectrum of **1-(3-chloro-4,6-dihydroxy-2-(6-phenylhexyl)phenyl)ethan-1-one** in DMSO-d_6 at 600 MHz. 149

COSY NMR spectrum of **1-(3-chloro-4,6-dihydroxy-2-(6-phenylhexyl)phenyl)ethan-1-one** in DMSO-d_6 at 600 MHz. 149

ROESY NMR spectrum of **1-(3-chloro-4,6-dihydroxy-2-(6-phenylhexyl)phenyl)ethan-1-one** in DMSO-d_6 at 600 MHz. 150

29. 1-(3,5-dichloro-2,4-dihydroxy-6-(6-phenylhexyl)phenyl)ethan-1-one: 151

^{1}H NMR spectrum of **1-(3,5-dichloro-2,4-dihydroxy-6-(6-phenylhexyl)phenyl)ethan-1-one** in DMSO-d_6 at 600 MHz. 151

HSQC NMR spectrum of **1-(3,5-dichloro-2,4-dihydroxy-6-(6-phenylhexyl)phenyl)ethan-1-one** in DMSO-d_6 at 600 MHz. 152

HMBC NMR spectrum of **1-(3,5-dichloro-2,4-dihydroxy-6-(6-phenylhexyl)phenyl)ethan-1-one** in DMSO-d_6 at 600 MHz. 152

COSY NMR spectrum of **1-(3,5-dichloro-2,4-dihydroxy-6-(6-phenylhexyl)phenyl)ethan-1-one** in DMSO-d_6 at 600 MHz. 153

ROESY NMR spectrum of **1-(3,5-dichloro-2,4-dihydroxy-6-(6-phenylhexyl)phenyl)ethan-1-one** in DMSO-d_6 at 600 MHz. 153

Supplementary Figures

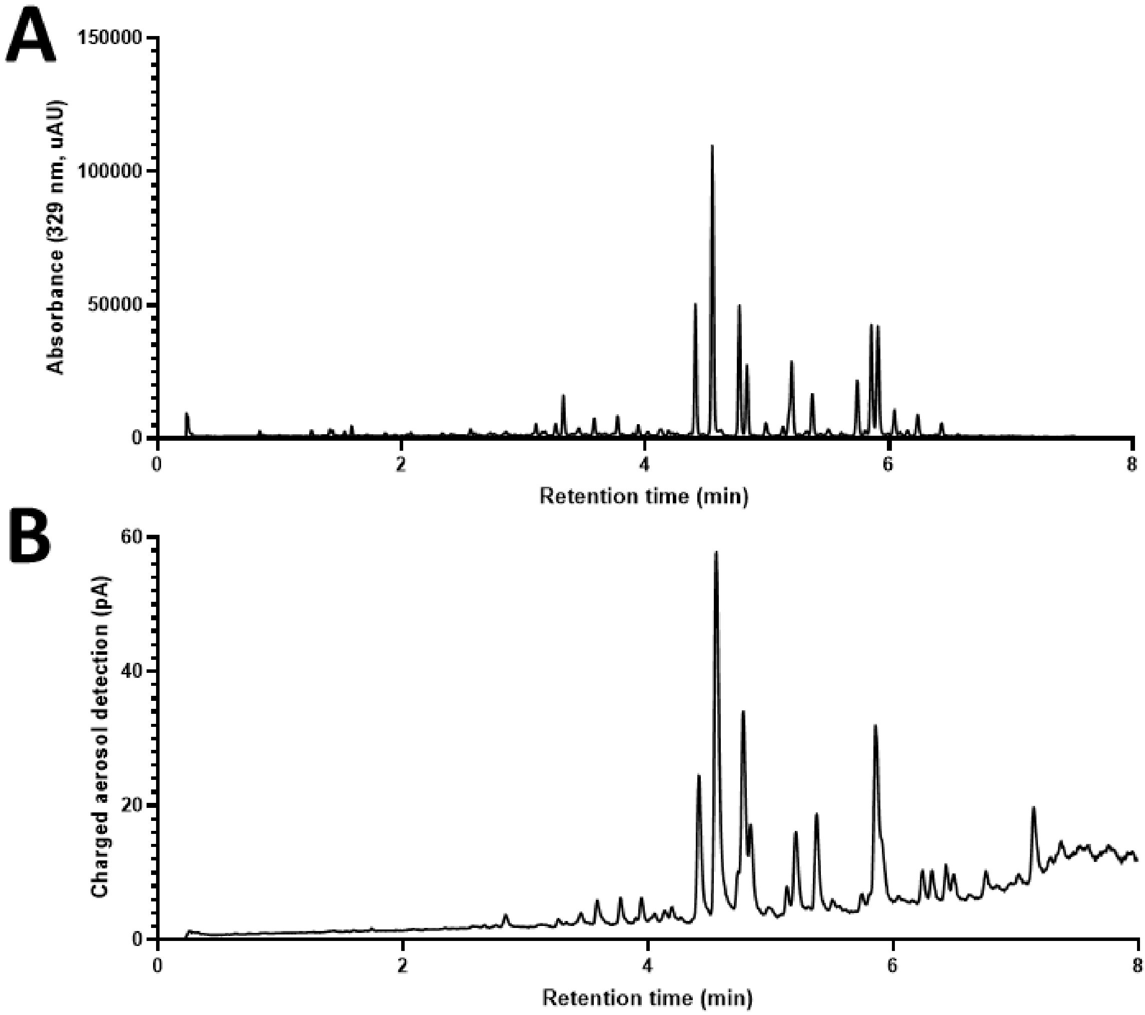

Supplementary Figure 1 - Chromatographic separations of the ethyl acetate extract of *K. oblongifolia*. (standard 5% to 100% acetonitrile gradient in 8 min). A): UV chromatogram at 329 nm of the extract. B): CAD chromatogram of the extract.

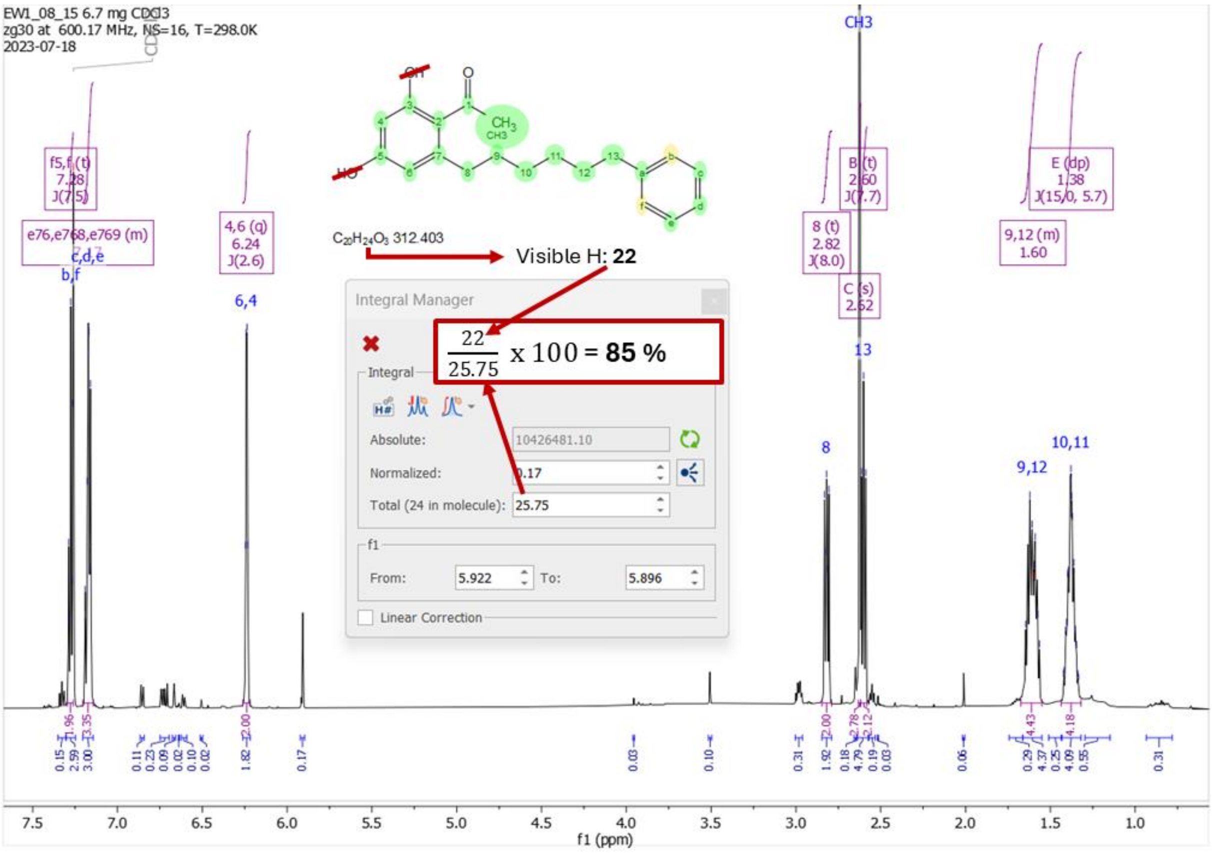

Supplementary Figure 2 – Process for the purity estimation - ^{1}H NMR spectrum of compound 4, with all visible impurity peaks integrated, giving a **measured total integral value** of **25.75** (excluding the solvent peak), compared to the expected **22 visible H** atoms, corresponding to purity of **85%**.

Supplementary Tables

Supplementary Table 1 – Calculated purity scores for all 12 isolated NPs *: Compounds with purity considered as acceptable according to calculated values (>90%); *n.b.*: For all compounds, the level of impurities did not preclude their unambiguous structural identification (by NMR & HRMS).

**Compound number**	**Compound name**	**Total absolute integral**	**Number of ^{1}H (without OH counted)**	**Purity**
**1**	Knemolone A	51.34	18	**35%**
**2**	Knemolone B	28.92	18	**62%**
**3**	Knemolic acid A	39.66	16	**40%**
**4**	Knemolone C	25.75	22	**85%**
**5**	Knemolone D	32.44	24	**74%**
**6**	Knemolic acid B	23.75	20	**84%**
**7**	Knemolic acid C	20.86	20	**96%***
**8**	Khookerianic acid A	24.43	22	**90%***
**9**	Kneglobularic acid B	39.86	24	**60%**
**10**	Khookerianic acid C	32.01	24	**75%**
**11**	Anagigantic acid	35.43	26	**73%**
**12**	6-Tridecylsalicylic acid	67.48	30	**44%**
**28**	3-chloro-knemolone C	23.31 (OH visible)	23 (with OH)	**99%**
**29**	3,5-dichloro-knemolone C	20.68	20	**97%**

Supplementary Table 2 - Biological activities of NPs isolated from *K. oblongifolia* ethyl acetate leaves extract. Anacardic was added as a standard of the same chemical class as some isolated NPs, Rifabutin as positive control for anti-mycobacterial activity against Mm and Vancomycin for antibacterial activities against *Staphylococcus aureus*. Purity scores calculated were added as a point of caution to be considered alongside measure biological readouts.

Compound	IC_{50} on Mm in infection (µM)	IC_{50} on Dd in infection (µM)	IC_{50} on Mm *in broth* (µM)	MIC on Sa ($mg.L^{-1}$)	Calculated purity
**1**	22.22	22.22	Not active	Not active	**35%**
**2**	22.22	22.22	Not active	32	**62%**
**3**	66.67	66.67	Not active	8	**40%**
**4**	22.22	22.22	Not active	4	**85%**
**5**	Not active	22.22	Not active	2	**74%**
**6**	7.41	7.41	Not active	8	**84%**
**7**	7.41	7.41	Not active	8	**96%**
**8**	2.47	2.47	Not active	8	**90%**
**9**	7.41	22.22	Not active	8	**60%**
**10**	2.47	2.47	Not active	8	**75%**
**11**	2.47	2.47	Not active	8	**73%**
**12**	66.67	66.67	Not active	Not active	**44%**
**Anacardic acid**	22.22	Not active	Not active	16	Commercial standard
**Rifabutin**	0.2	Not active	0.03	-	Commercial standard
**Vancomycin**	-	-	-	1	Commercial standard

Supplementary Data: NMR and HRMS

Nuclear Magnetic Resonance (NMR) measurements

All Nuclear Magnetic Resonance (NMR) measurements were recorded on a Bruker Avance Neo 600 MHz NMR spectrometer (Cryoprobe QCI 5-mm) equipped with a SampleJet automated sample changer (Bruker BioSpin, Rheinstetten, Germany). Chemical shifts are presented in parts per million (δ), referencing the residual $CDCl_3$ (for NPs, δ_H 7.26; δ_C 77.0) or DMSO-d_6 (for synthetic molecules, δ_H 2.50; δ_C 39.5) signals as internal standards for 1H and ^{13}C NMR, respectively, with coupling constants (*J*) reported in Hz. Additional 2D experiments (HSQC, HMBC, COSY and ROESY) as well as comparisons with literature were used when performing complete structural assignments.

UHPLC-DAD-HRMS/MS of extracts, fractions and pure compounds

Analyses were performed with a Waters Acquity UHPLC system coupled to a Corona Veo RS Charged Aerosol Detector (CAD, Thermo Scientific, Germany) and an Orbitrap Exploris 120 mass spectrometer (Thermo Scientific, Germany). The Orbitrap employed a heated electrospray ionization source (H-ESI) with the following parameters: spray voltage: +3.5 kV; ion transfer tube temperature: 320.00 °C; vaporizer temperature: 320.00 °C; S-lens RF: 45 (arb units); sheath gas flow rate: 35.00 (arb units); Sweep Gas (arb): 1 and auxiliary gas flow rate: 10.00 (arb. units). Control of the instruments was done using Thermo Scientific Xcalibur software v. 4.6.67.17. Full scans were acquired at a resolution of 30,000 fwhm (at m/ z 200) and MS2 scans at 15000 fwhm in the range of 100−1000 m/ z, with 1 microscan, time (ms): 200, an RF lens (%): 70; AGC target custom (Normalized AGC target (%): 300); maximum injection time (ms): 130; Microscans: 1; data type: profile; Use EASY-IC(TM): ON. The settings for dynamic exclusion mode were customized; Exclude after n times: 1; Exclusion duration (s): 5; Mass tolerance: ppm; low: 10, high: 10, Exclude isotopes: true. Appex detention: Desired Apex Window (%): 50. Isotope Exclusion: Assigned and unassigned with an exclusion window (m/z) for unassigned isotopes: 8. The Intensity threshold was set to 2.5E5 and a targeted mass exclusion list was used.

The centroid data-dependent MS2 (dd-MS2) scan acquisition events were performed in discovery mode, triggered by Apex detection with a trigger detection (%) of 300 with a maximum injection time of 120 ms, performing 1 microscan. The top 3 abundant precursors (charge states 1 and 2) within an isolation window of 1.2 m/z were considered for MS/MS analysis. For precursor fragmentation in the HCD mode, a normalized collision energy of 15, 30 and 45 % was used. Data was recorded in profile mode (Use EASY-IC(TM): ON).

The chromatographic separation was done on a Waters BEH C18 column (50 × 2.1 mm i.d., 1.7 µm, Waters, Milford, MA) using the following gradient (time (min), %B): 5%B from 0 to 0.5 min; from 5%B to 100%B between 0.5 and 7 min; 100%B from 7 to 8 min, from 100%B to 5%B from 8 to 8.10 min; 5%B from 8.10 to 10 min. The mobile phases were (A) water and (B) acetonitrile both containing 0.1% FA. The flow rate was set to 600 µL/min, the injection volume was 2 µL and the column was kept at 40 °C. The PDA detector was used from 210 to 400 nm with a resolution of 1.2 nm. The CAD detector was kept at 40 °C, with 5 bar N_2 and power function 1 for a data collection rate of 20 Hz.

Natural Products from *Knema oblongifolia* (leaves)

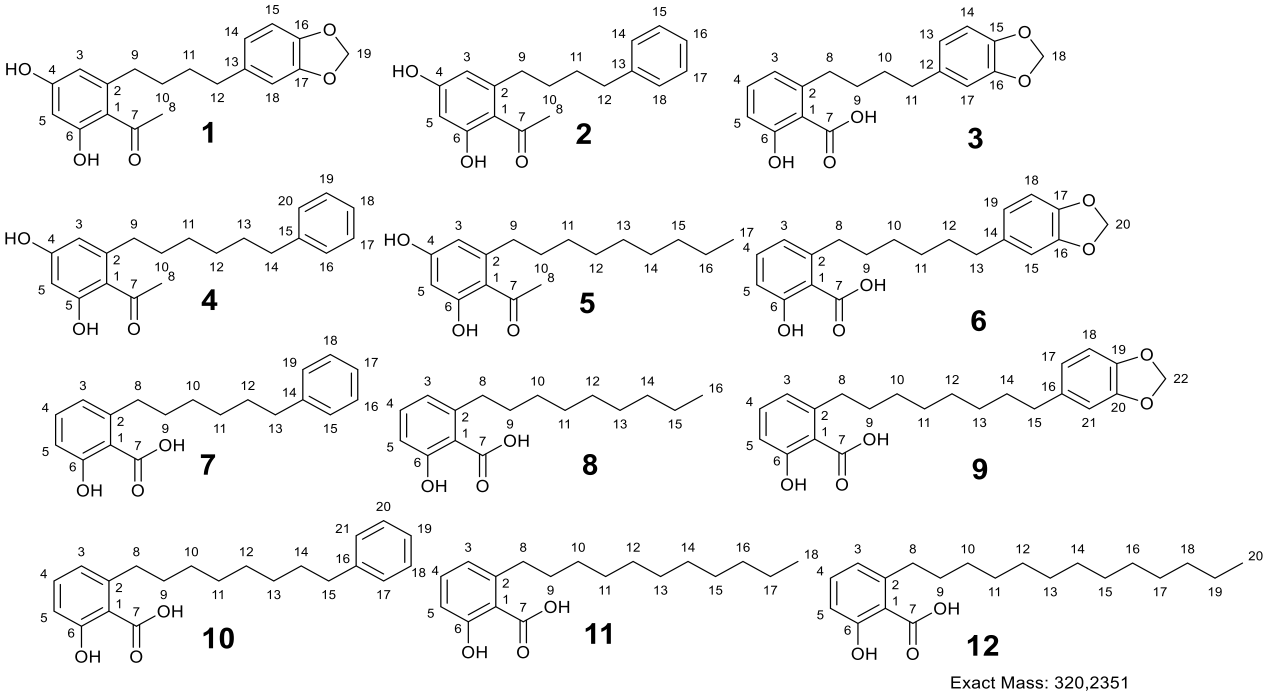

1. Knemolone A:

Experimental:

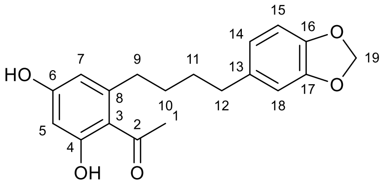

Knemolone A (**1**) Yellow amorphous solid; UV (MeOH) λ_{max} (log ε) 201 (3.83), 222 (4.09), 285 (3.35) nm; ^{1}H NMR ($CDCl_3$, 600 MHz) δ 6.72 (1H, d, J = 7.8 Hz), 6.65 (1H, s), 6.60 (1H, d, J = 7.8 Hz), 6.24 (1H, d, J = 2.6 Hz), 6.22 (1H, d, J = 2.5 Hz), 5.92 (2H, s), 2.85 (2H, t, J = 7.5 Hz), 2.60 (2H, s), 2.56 (2H, t, J = 7.1 Hz), 1.64 (4H, dp, J = 11.8, 7.5 Hz); ^{13}C NMR ($CDCl_3$, 151 MHz) δ 204.2, 166.1, 160.9, 147.7, 147.5, 145.8, 136.0, 121.2, 115.5, 110.7, 108.9, 108.3, 101.9, 100.9, 36.3, 35.5, 32.3, 31.7, 31.6 (NP-MRD ID: NP0333015); HRESIMS *m/z* 327.1237 $[M-H]^-$ (calcd for $C_{19}H_{19}O_5^-$ 327.1238, Δ = -0.31 ppm), *m/z* 329.1382 $[M+H]^+$ (calcd for $C_{19}H_{21}O_5^+$ 329.1384, Δ = -0.61 ppm), MS/MS spectrum: CCMSLIB00012475055.

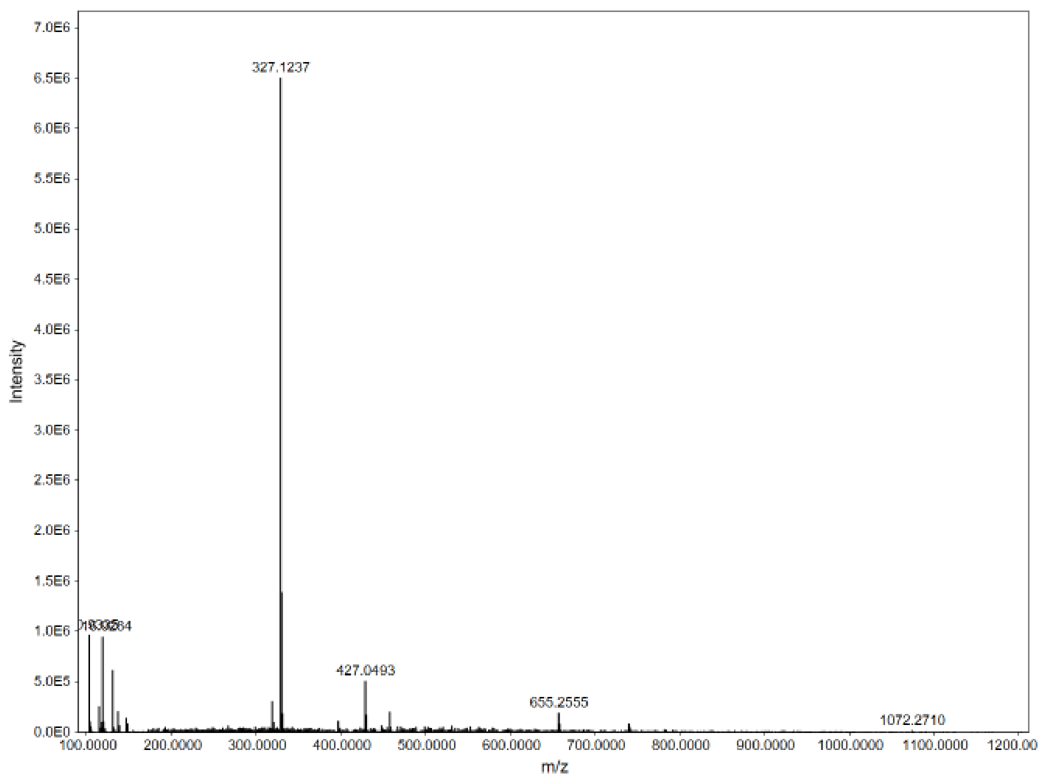

HRESIMS- spectrum of **Knemolone A** in MeOH.

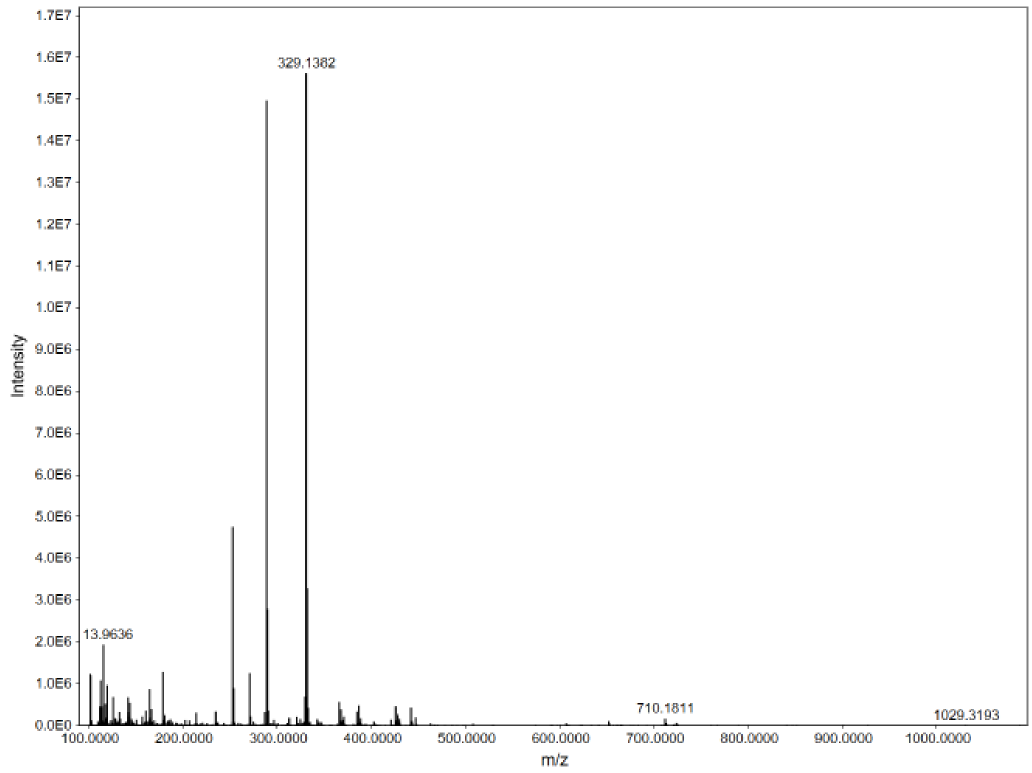

HRESIMS+ spectrum of **Knemolone A** in MeOH.

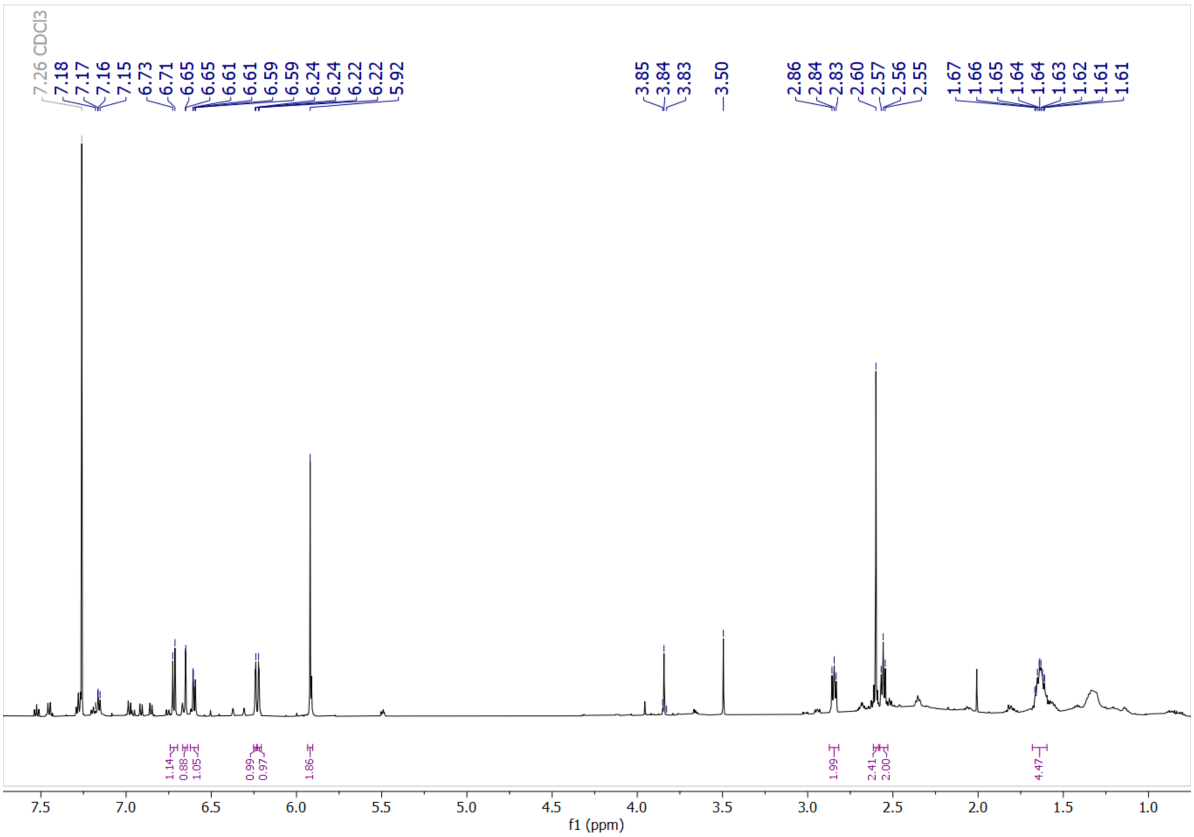

^{1}H NMR spectrum of **Knemolone A** in $CDCl_3$ at 600 MHz.

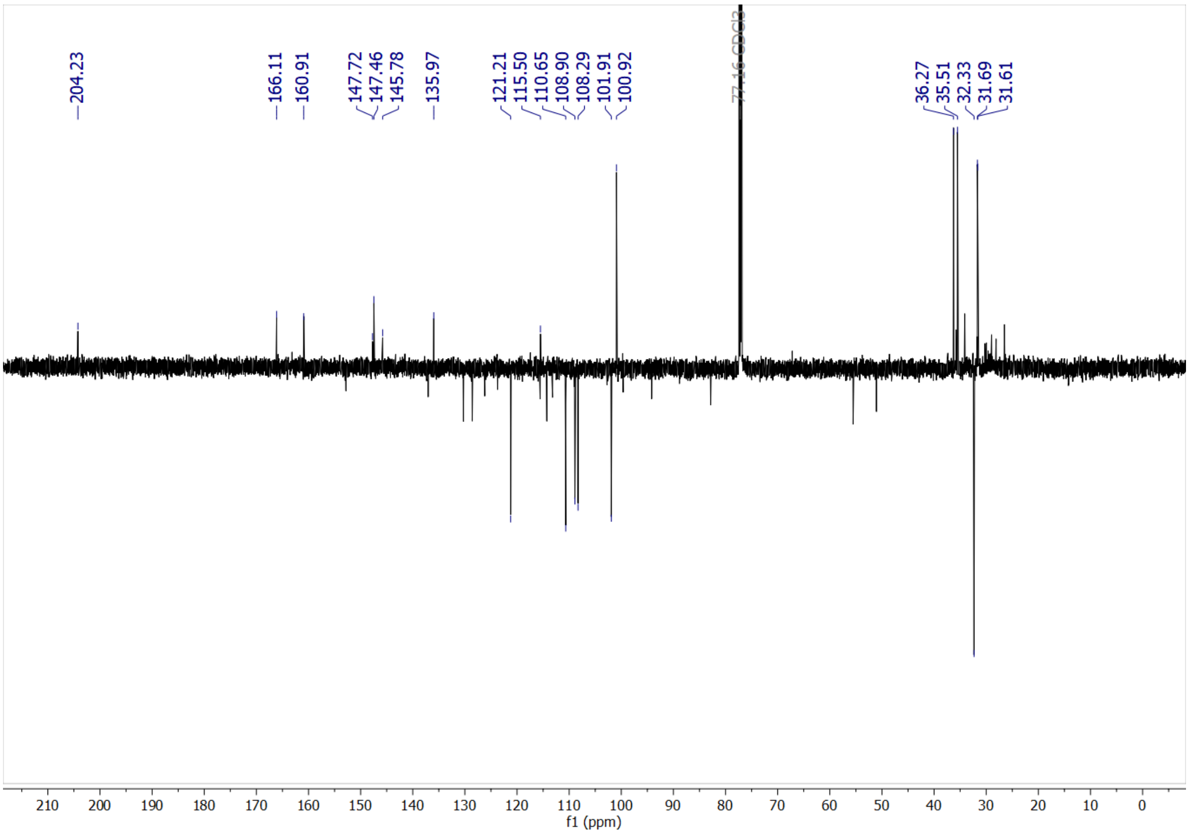

^{13}C DEPTQ NMR spectrum of **Knemolone A** in $CDCl_3$ at 151 MHz.

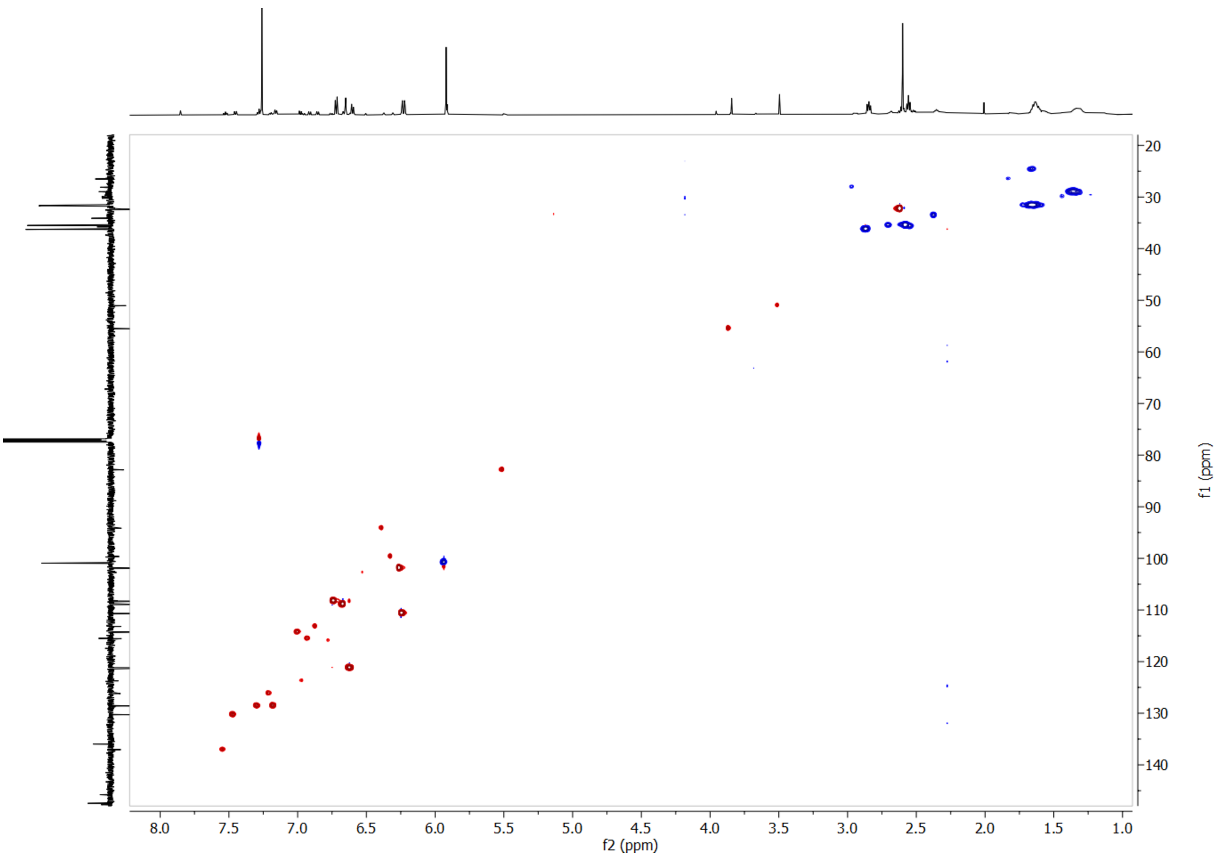

HSQC NMR spectrum of **Knemolone A** in $CDCl_3$ at 600 MHz.

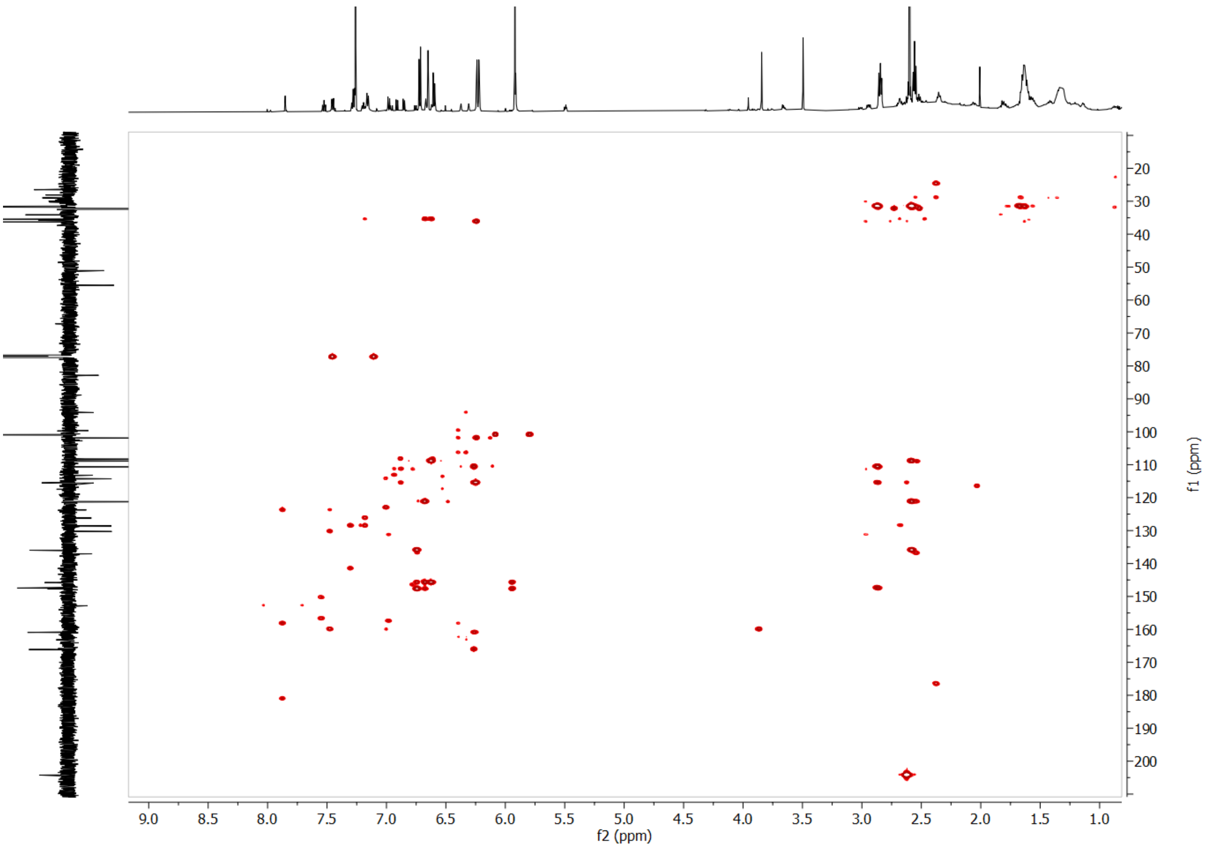

HMBC NMR spectrum of **Knemolone A** in $CDCl_3$ at 600 MHz.

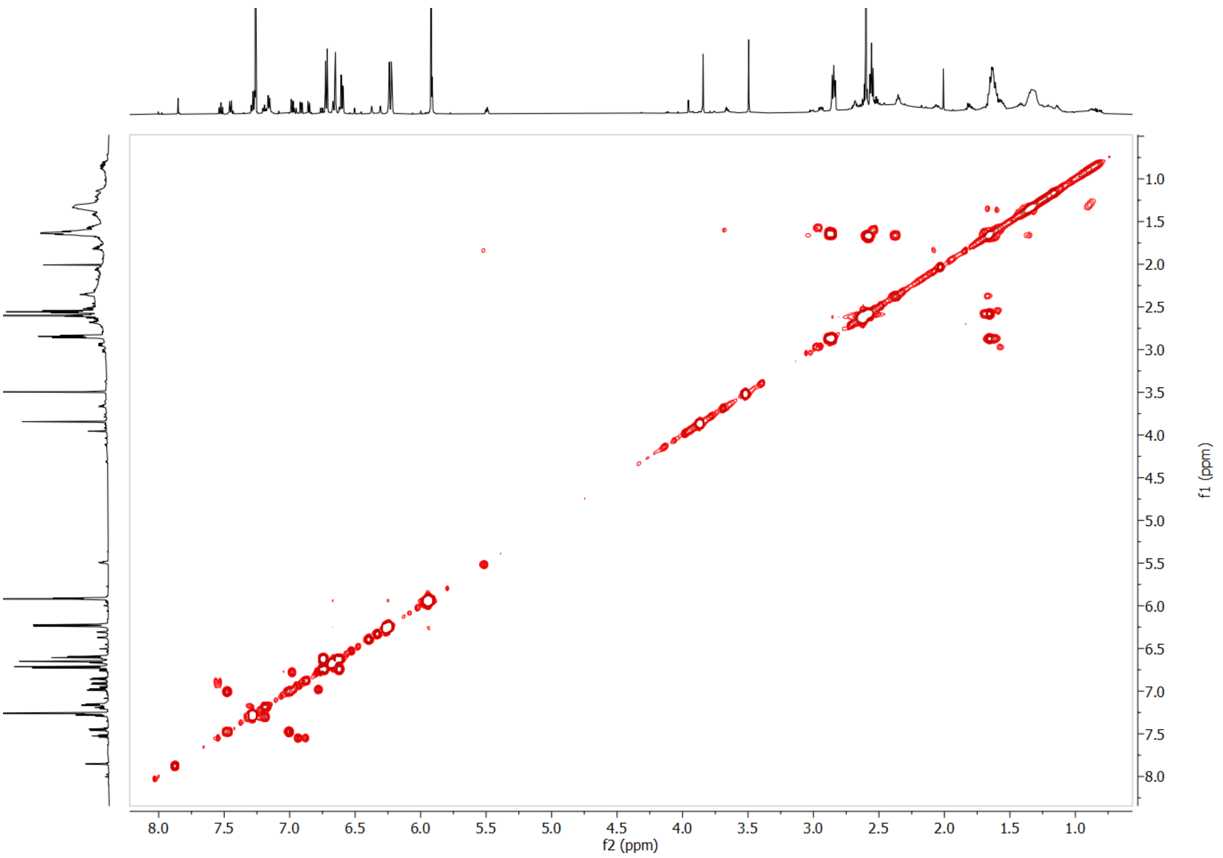

COSY NMR spectrum of **Knemolone A** in $CDCl_3$ at 600 MHz.

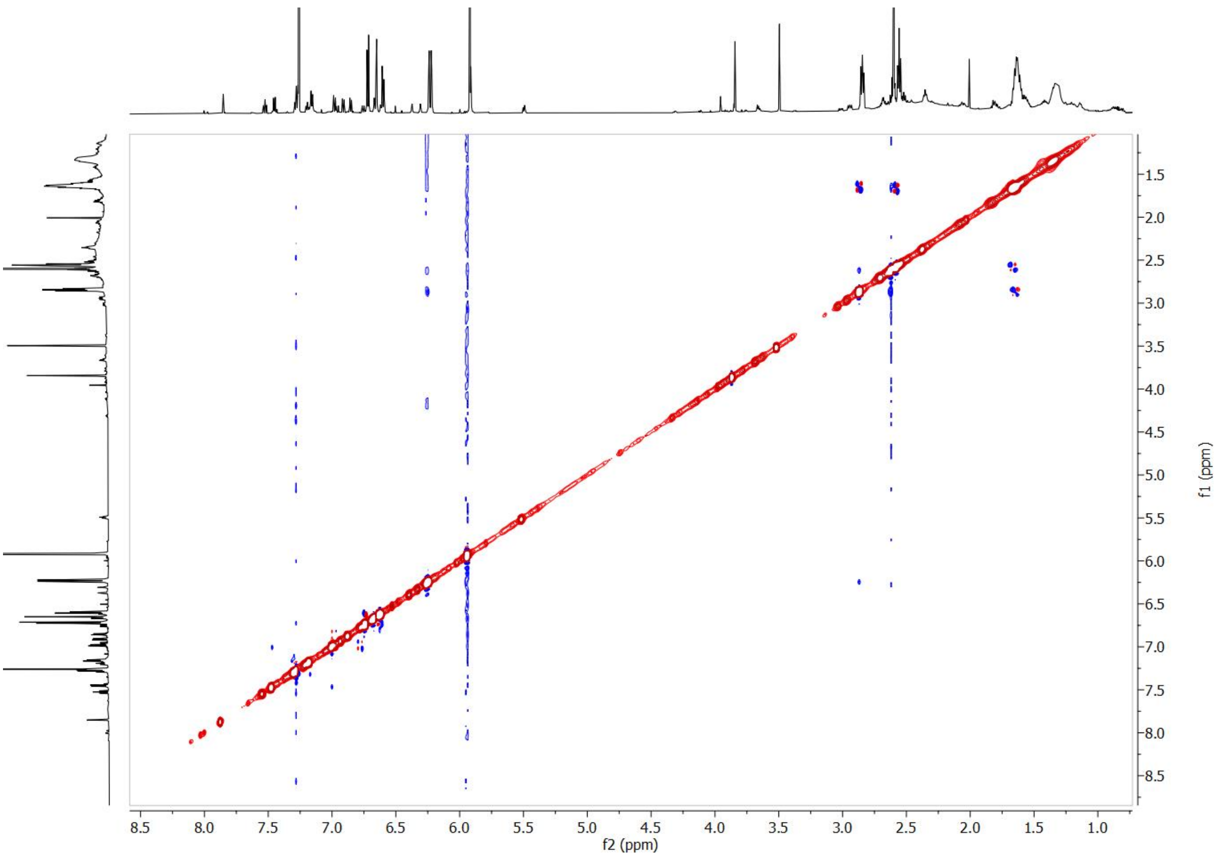

ROESY NMR spectrum of **Knemolone A** in $CDCl_3$ at 600 MHz.

2. Knemolone B:

Experimental:

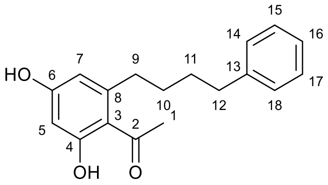

Knemolone B (**2**) Yellow amorphous solid; UV (MeOH) λ_{max} (log ε) 222 (3.95), 282 (3.03) nm; ^{1}H NMR ($CDCl_3$, 600 MHz) δ 7.28 (2H, t, J = 7.5 Hz), 7.22 – 7.14 (3H, m), 6.24 (1H, d, J = 2.4 Hz), 6.21 (1H, d, J = 2.5 Hz), 2.86 (1H, t, J = 7.5 Hz), 2.64 (2H, t, J = 7.5 Hz), 2.59 (3H, s), 1.71 (1H, p, J = 7.1 Hz), 1.64 (1H, p, J = 7.2 Hz); ^{13}C NMR (151 MHz, $CDCl_3$) δ 204.3, 166.1, 160.8, 147.5, 142.1, 128.5, 128.5, 126.0, 110.6, 101.9, 36.3, 35.8, 32.3, 31.7, 31.5 (NP-MRD ID: NP0333016); HRESIMS *m/z* 283.1337 $[M-H]^-$ (calcd for $C_{18}H_{19}O_3^-$ 283.1340, Δ = -1.06 ppm), *m/z* 285.1483 $[M+H]^+$ (calcd for $C_{18}H_{21}O_3^+$ 285.1485, Δ = -0.70 ppm), MS/MS spectrum: CCMSLIB00012475054.

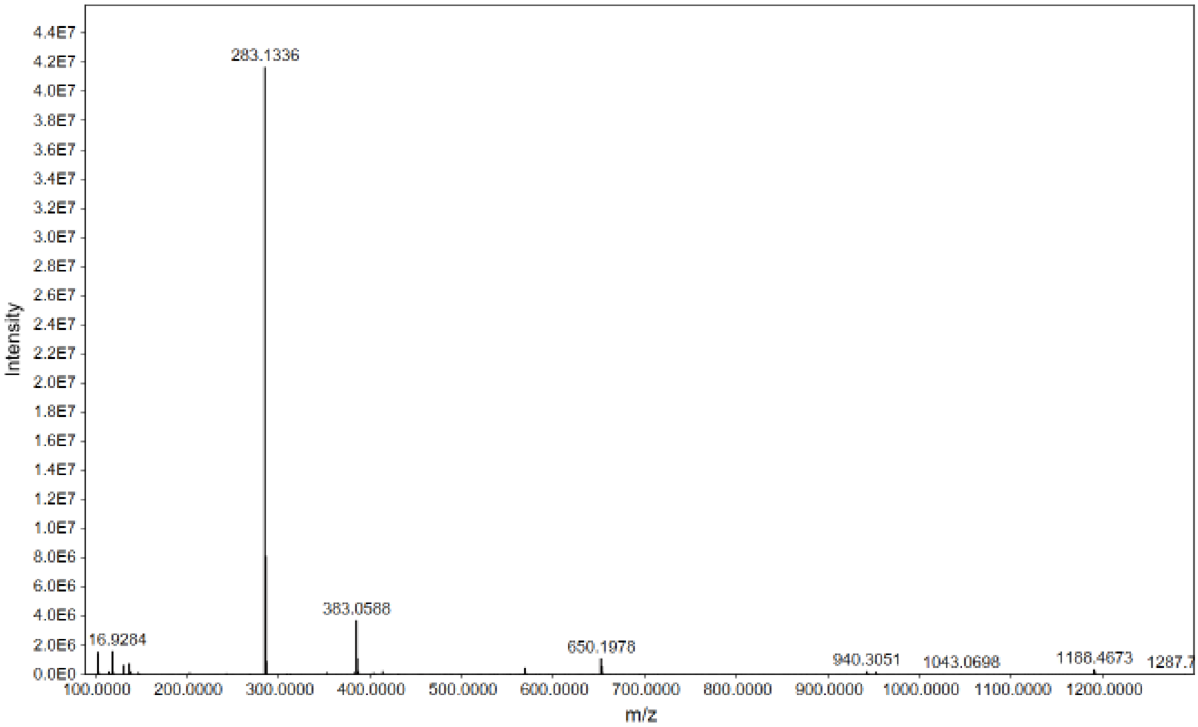

HRESIMS- spectrum of **Knemolone B** in MeOH.

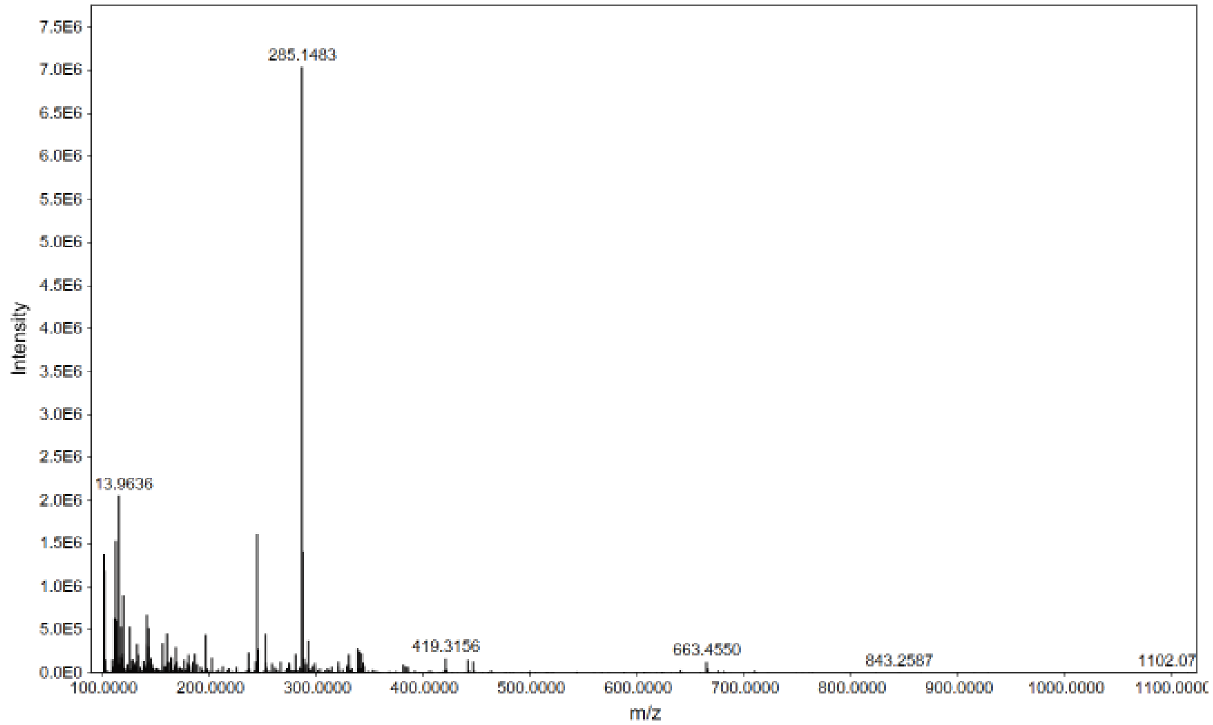

HRESIMS+ spectrum of **Knemolone B** in MeOH.

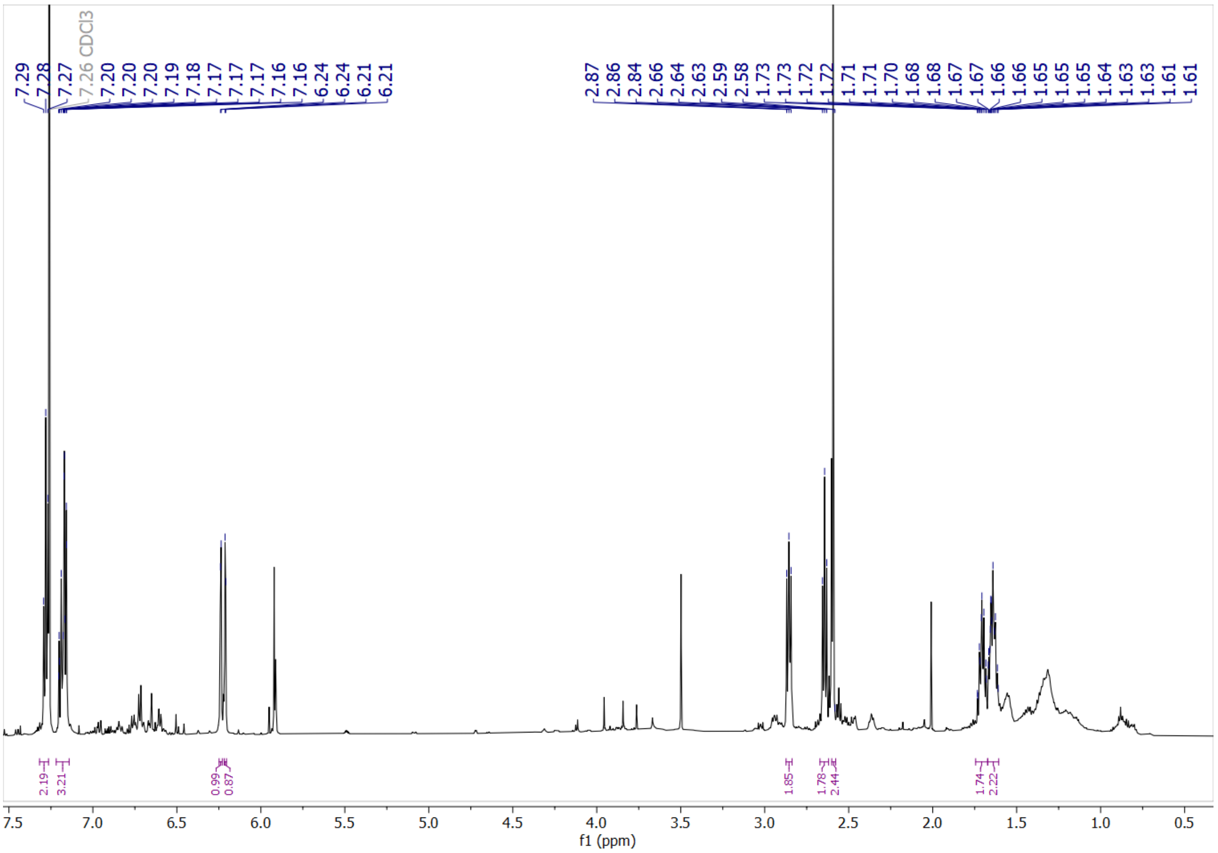

^{1}H NMR spectrum of **Knemolone B** in $CDCl_3$ at 600 MHz.

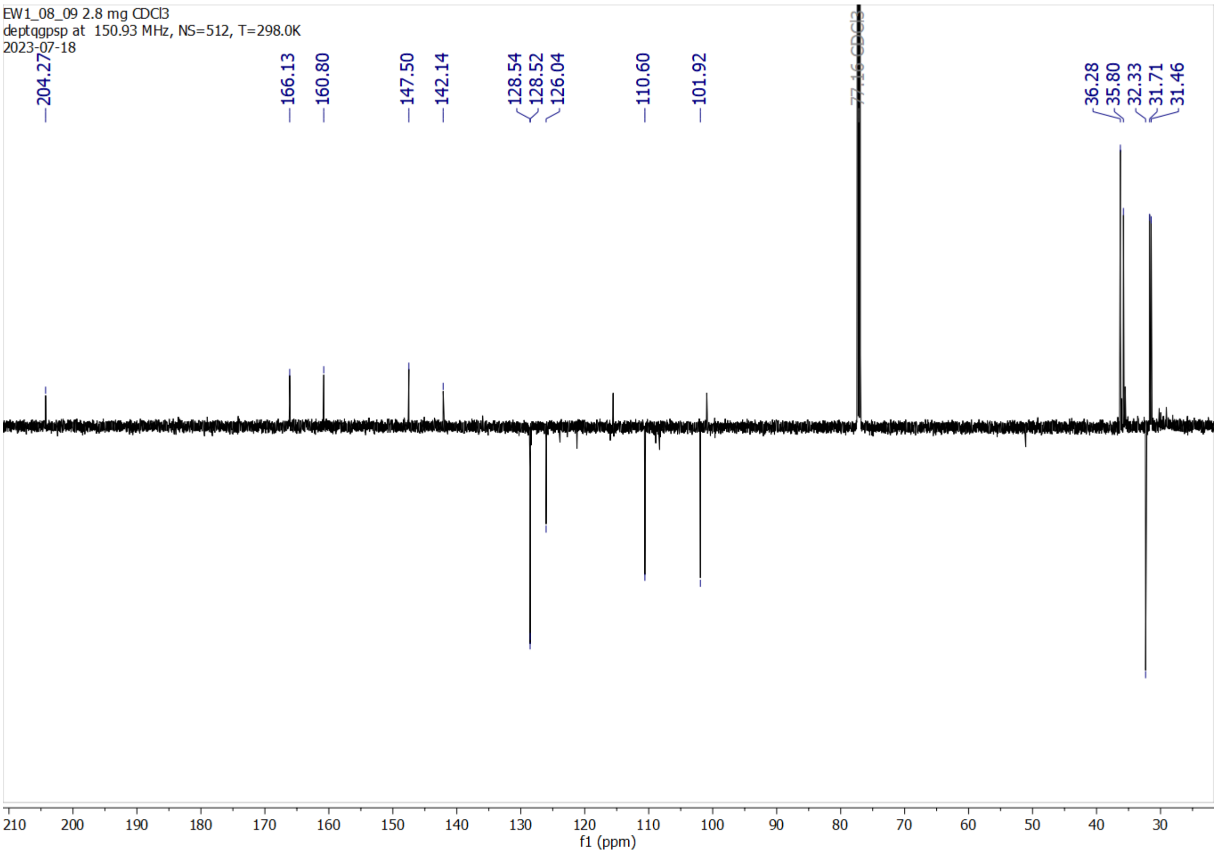

^{13}C DEPTQ NMR spectrum of **Knemolone B** in $CDCl_3$ at 151 MHz.

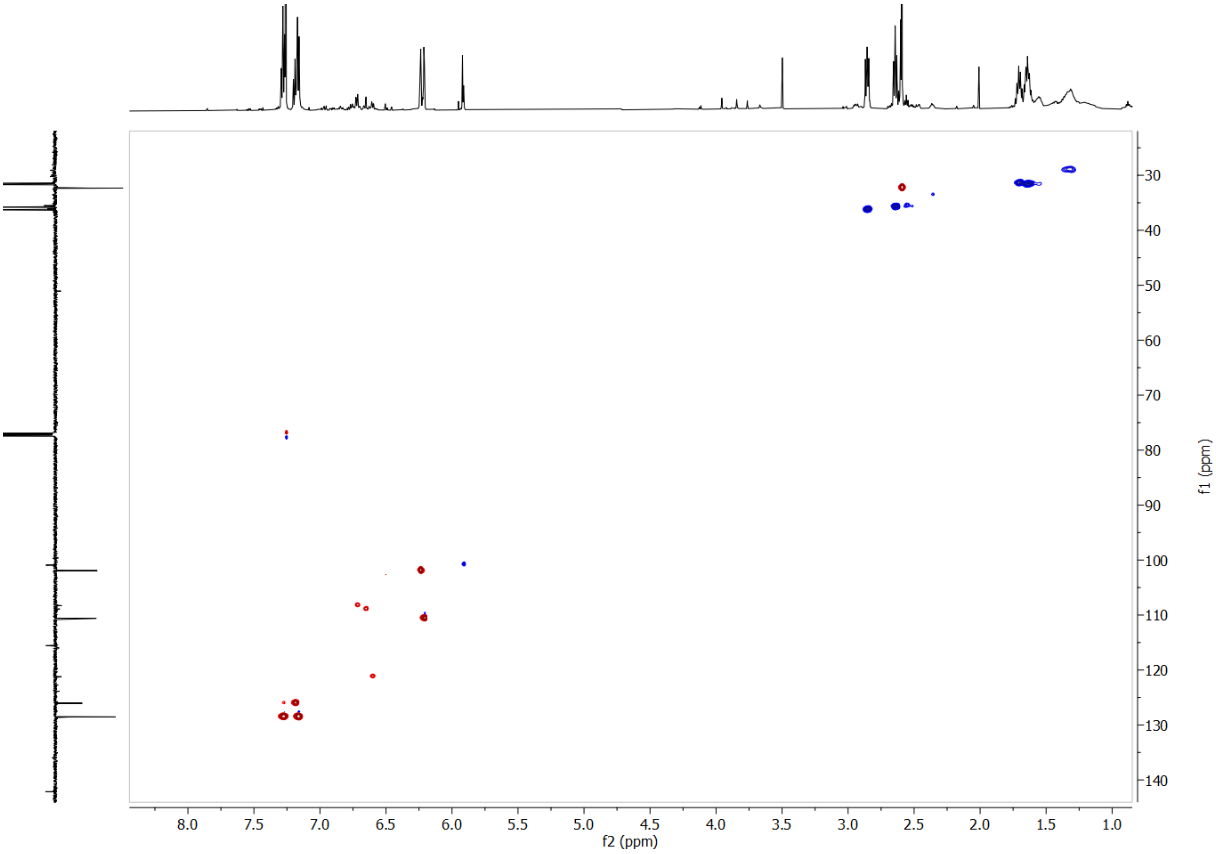

HSQC NMR spectrum of **Knemolone B** in $CDCl_3$ at 600 MHz.

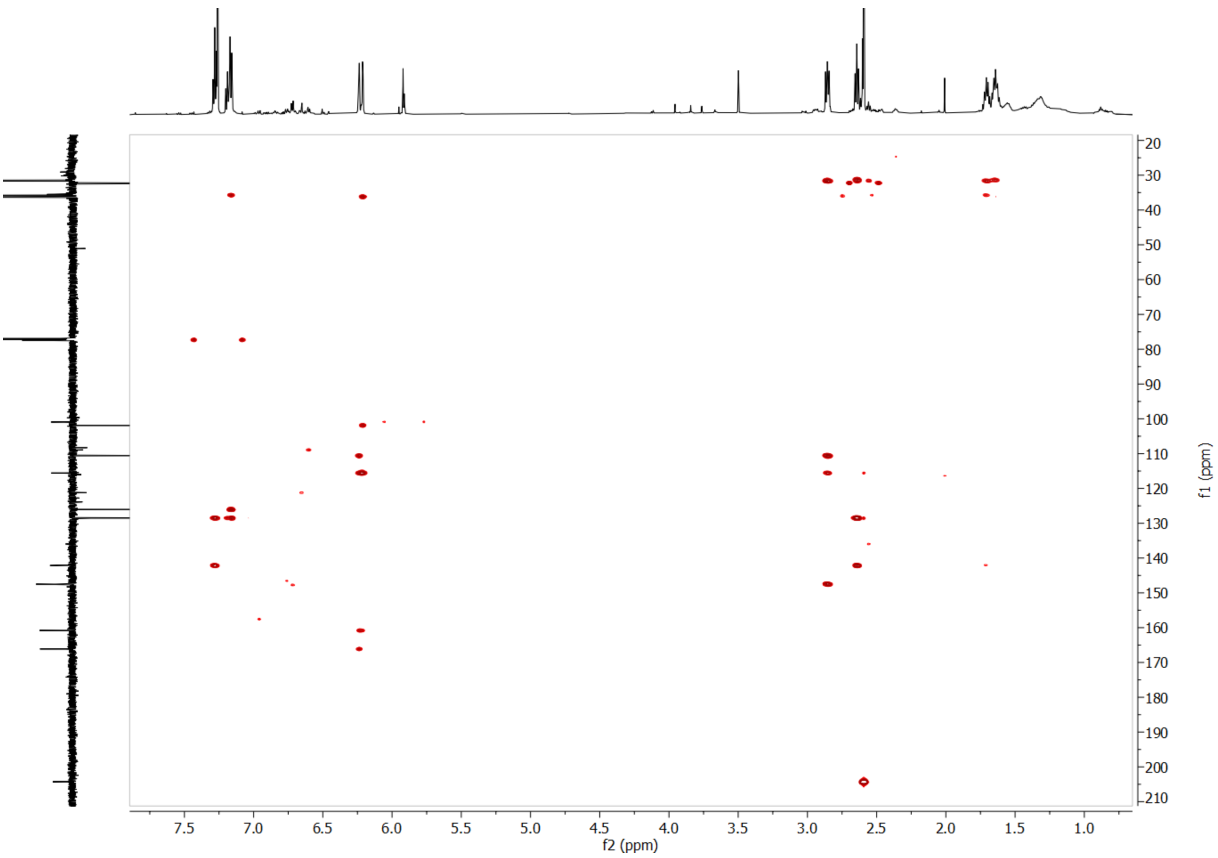

HMBC NMR spectrum of **Knemolone B** in $CDCl_3$ at 600 MHz.

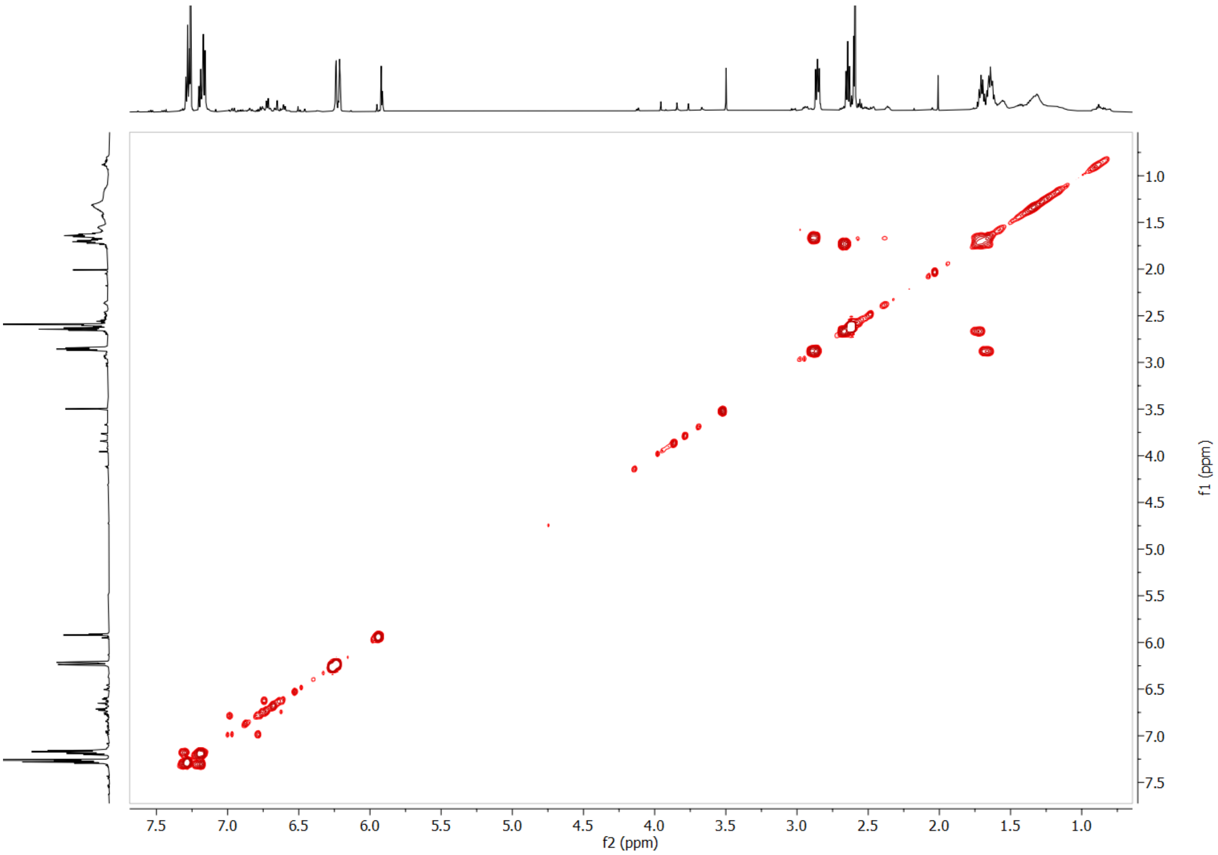

COSY NMR spectrum of **Knemolone B** in $CDCl_3$ at 600 MHz.

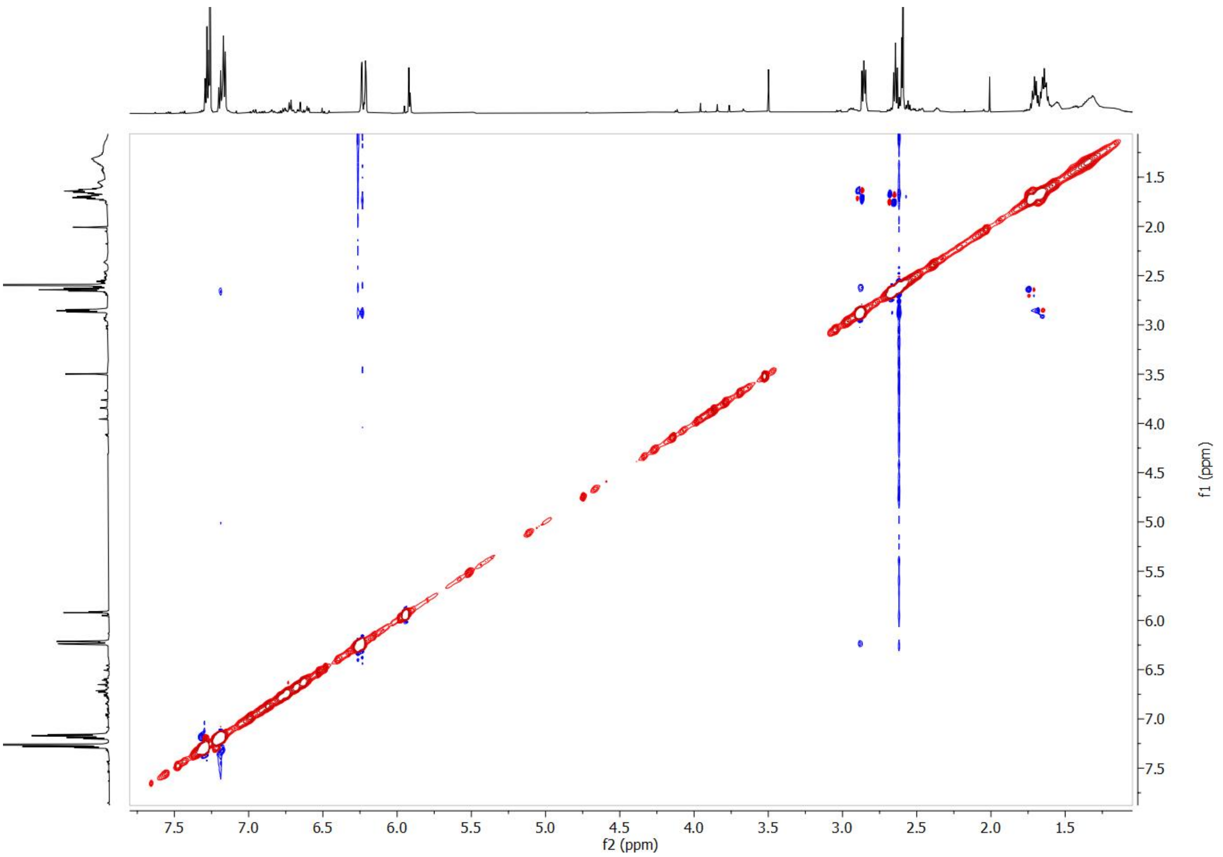

ROESY NMR spectrum of **Knemolone B** in $CDCl_3$ at 600 MHz.

3. Knemolic acid A:

Experimental:

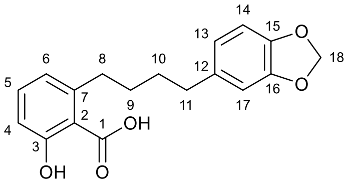

Knemolic acid A (**3**) Yellow amorphous solid; UV (MeOH) λ_{max} (log ε) 219 (4.04), 288 (3.06) nm; ^{1}H NMR ($CDCl^3$, 600 MHz) δ 7.34 (1H, t, J = 7.9 Hz), 6.86 (1H, dd, J = 8.4, 1.3 Hz), 6.74 (1H, dd, J = 7.5, 1.3 Hz), 6.71 (1H, d, J = 7.8 Hz), 6.67 (1H, d, J = 1.8 Hz), 6.61 (1H, dd, J = 7.9, 1.8 Hz), 5.91 (2H, s), 2.98 (2H, t, J = 7.3 Hz), 2.55 (2H, t, J = 7.0 Hz), 1.63 (4H, dp, J = 9.2, 5.2 Hz); ^{13}C NMR (151 MHz, $CDCl_3$) δ 174.0, 163.8, 147.6, 147.1, 145.6, 136.5, 135.3, 122.8, 121.2, 116.0, 110.5, 108.9, 108.2, 100.8, 36.5, 35.6, 31.9, 31.6 (NP-MRD ID: NP0333017); HRESIMS *m/z* 313.1079 $[M-H]^-$ (calcd for $C_{18}H_{17}O_5^-$ 313.1081, Δ = -0.64 ppm), *m/z* 297.1119 $[M-H_2O+H]^+$ (calcd for $C_{18}H_{17}O_4^+$ 297.1121, Δ = -0.67 ppm), MS/MS spectrum: CCMSLIB00012475067.

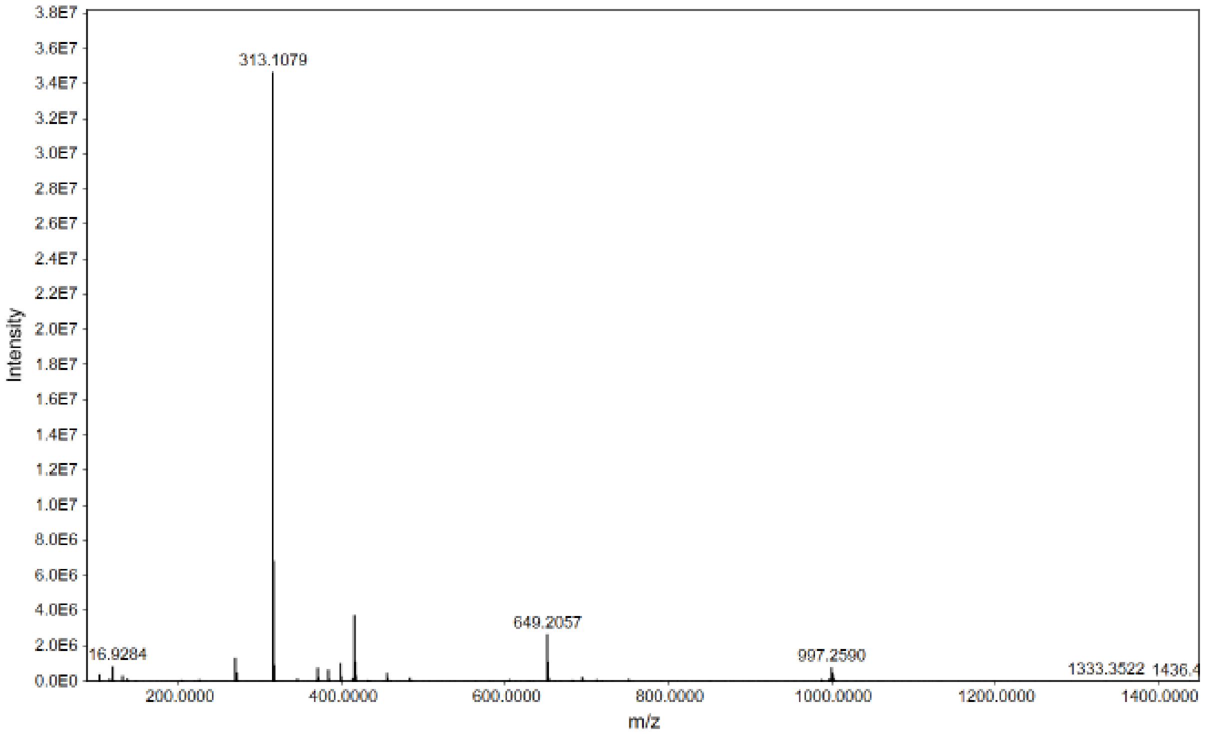

HRESIMS- spectrum of **Knemolic acid A** in MeOH.

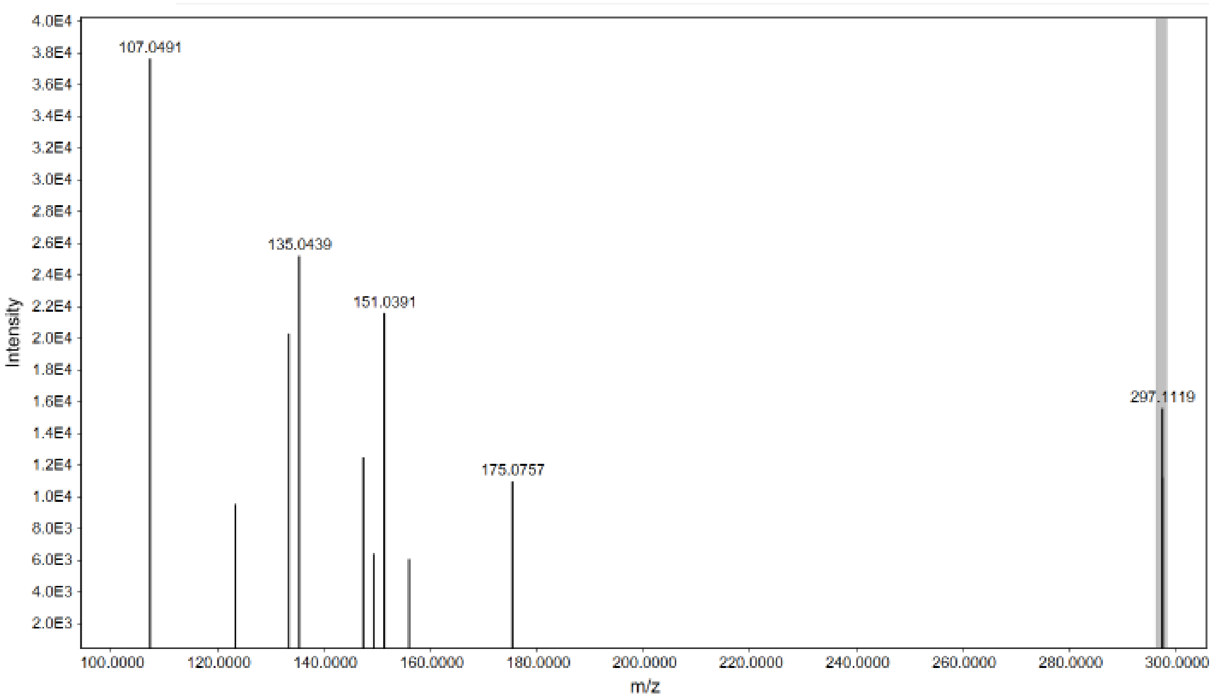

HRESIMS/MS+ fragmentation spectrum of **Knemolic acid A** in MeOH.

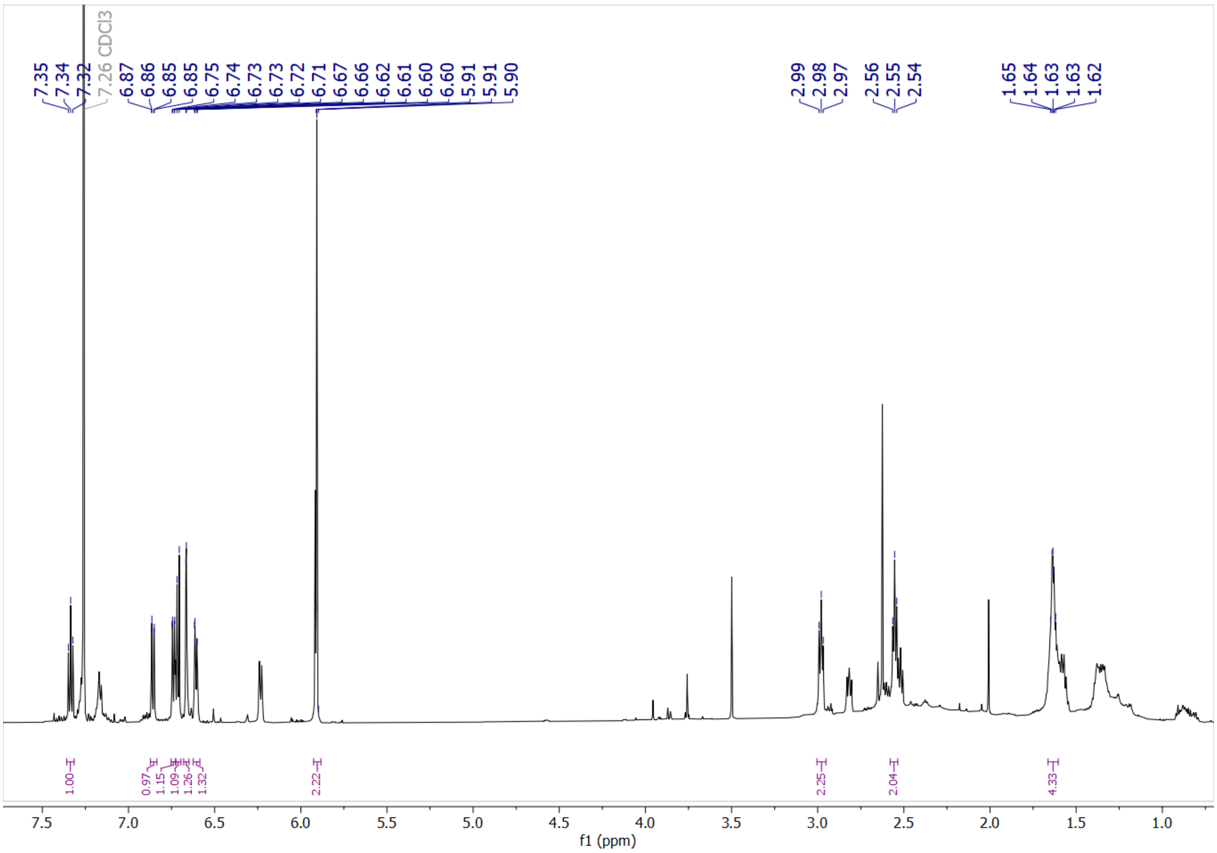

^{1}H NMR spectrum of **Knemolic acid A** in $CDCl_3$ at 600 MHz.

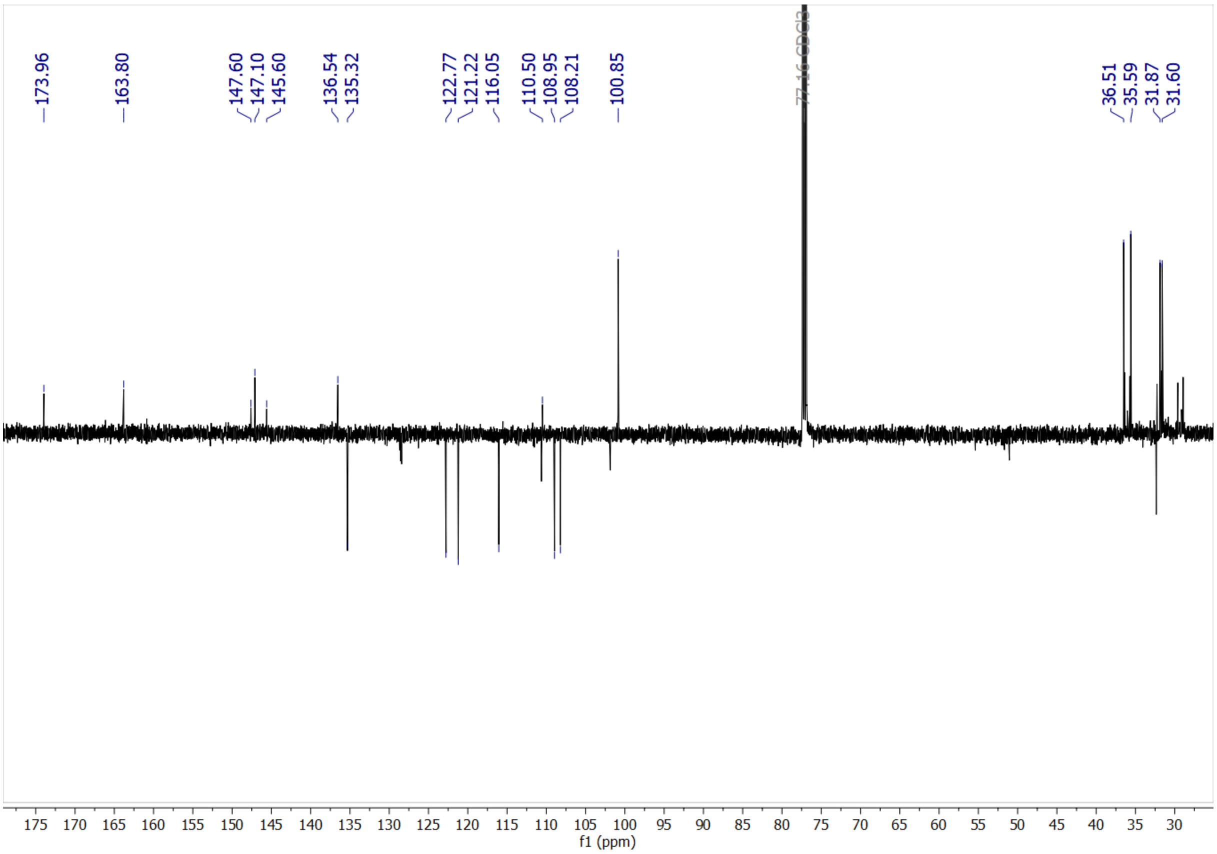

^{13}C DEPTQ NMR spectrum of **Knemolic acid A** in $CDCl_3$ at 151 MHz.

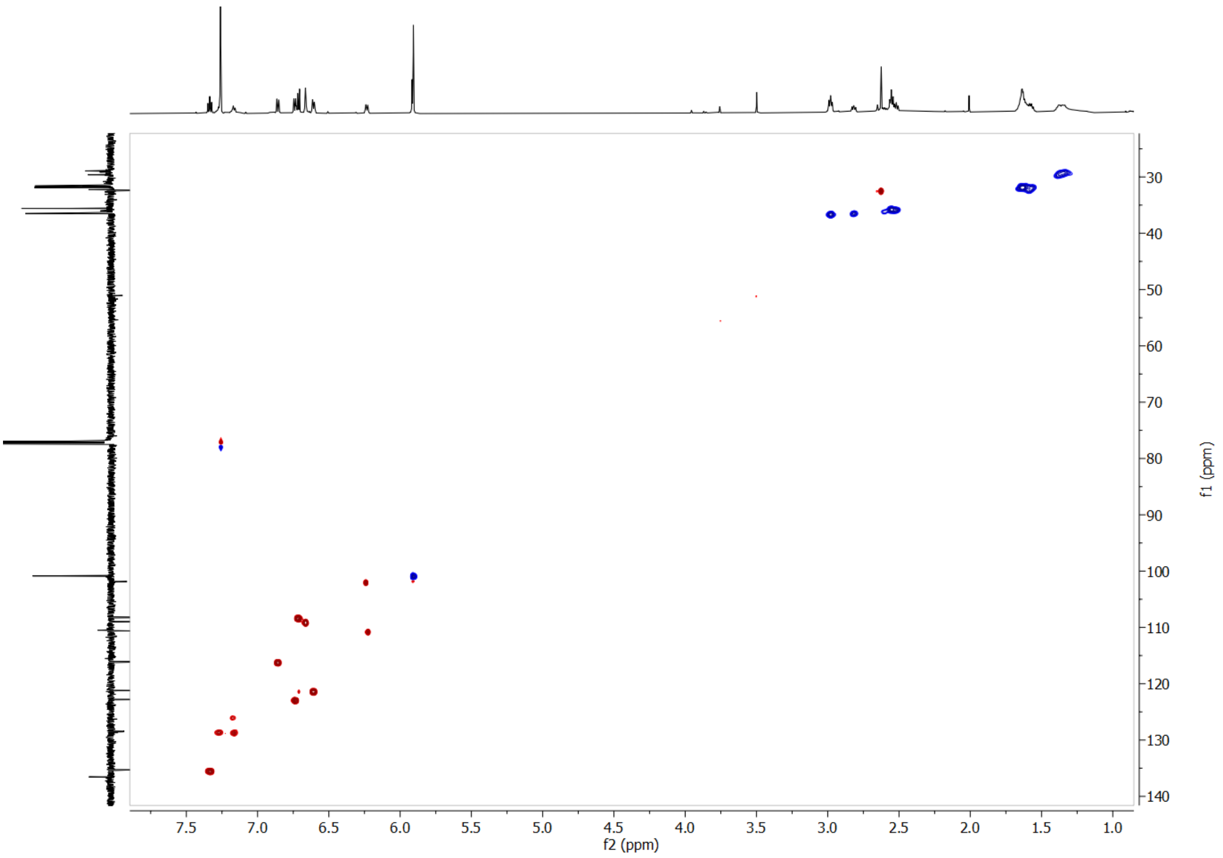

HSQC NMR spectrum of **Knemolic acid A** in $CDCl_3$ at 600 MHz.

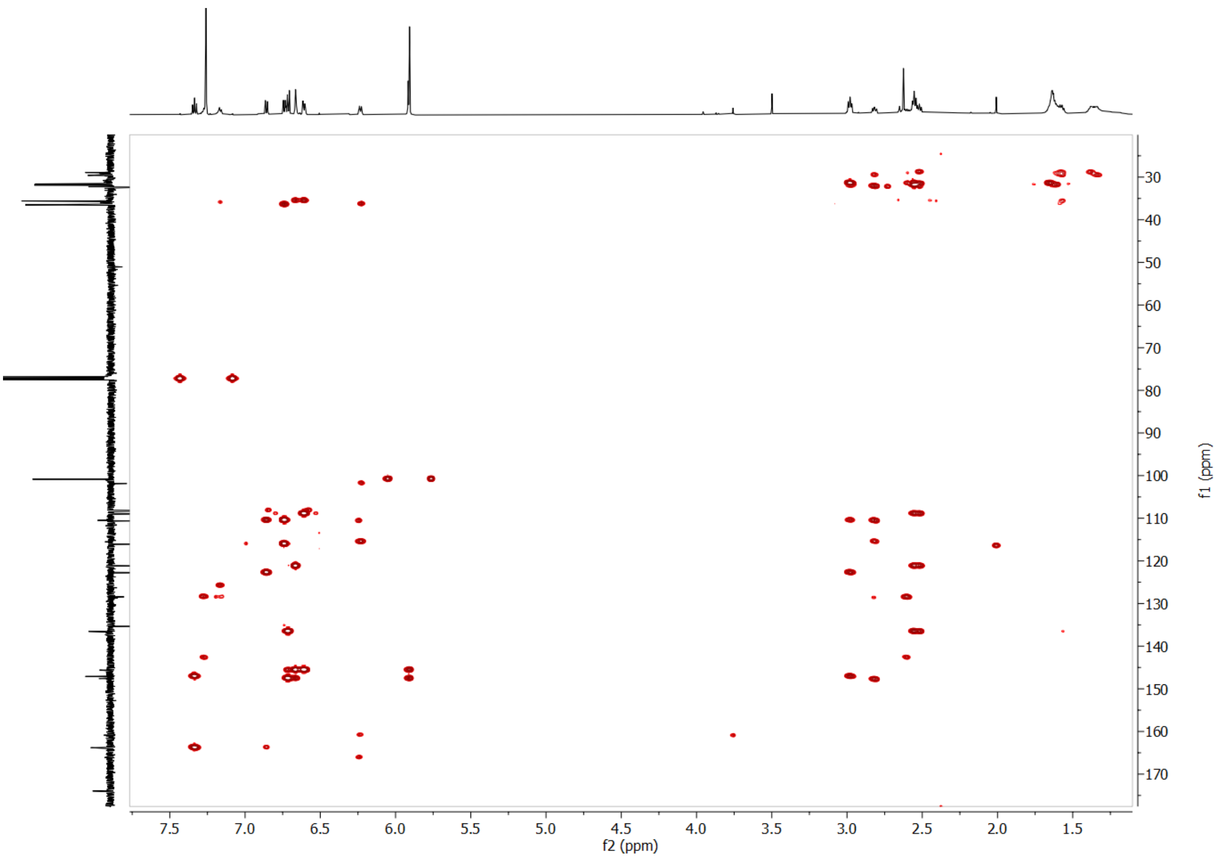

HMBC NMR spectrum of **Knemolic acid A** in $CDCl_3$ at 600 MHz.

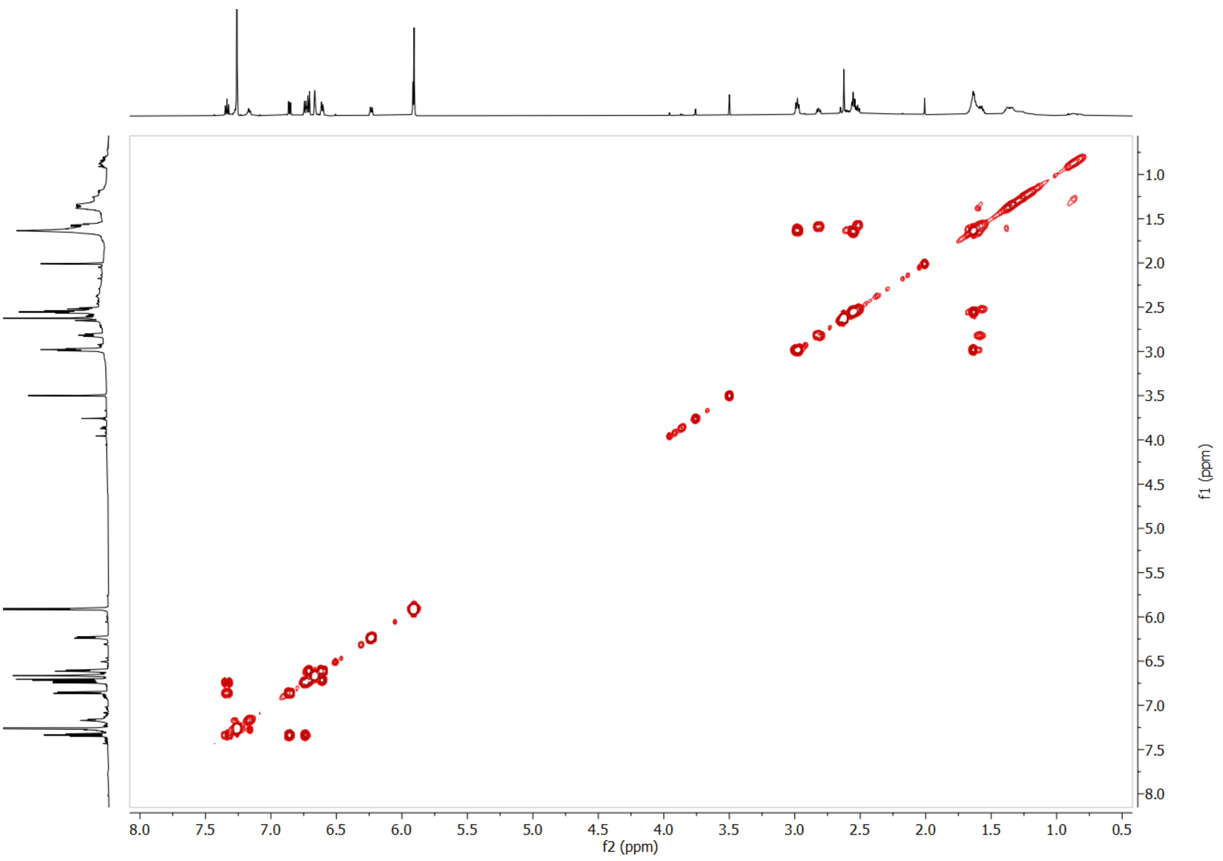

COSY NMR spectrum of **Knemolic acid A** in $CDCl_3$ at 600 MHz.

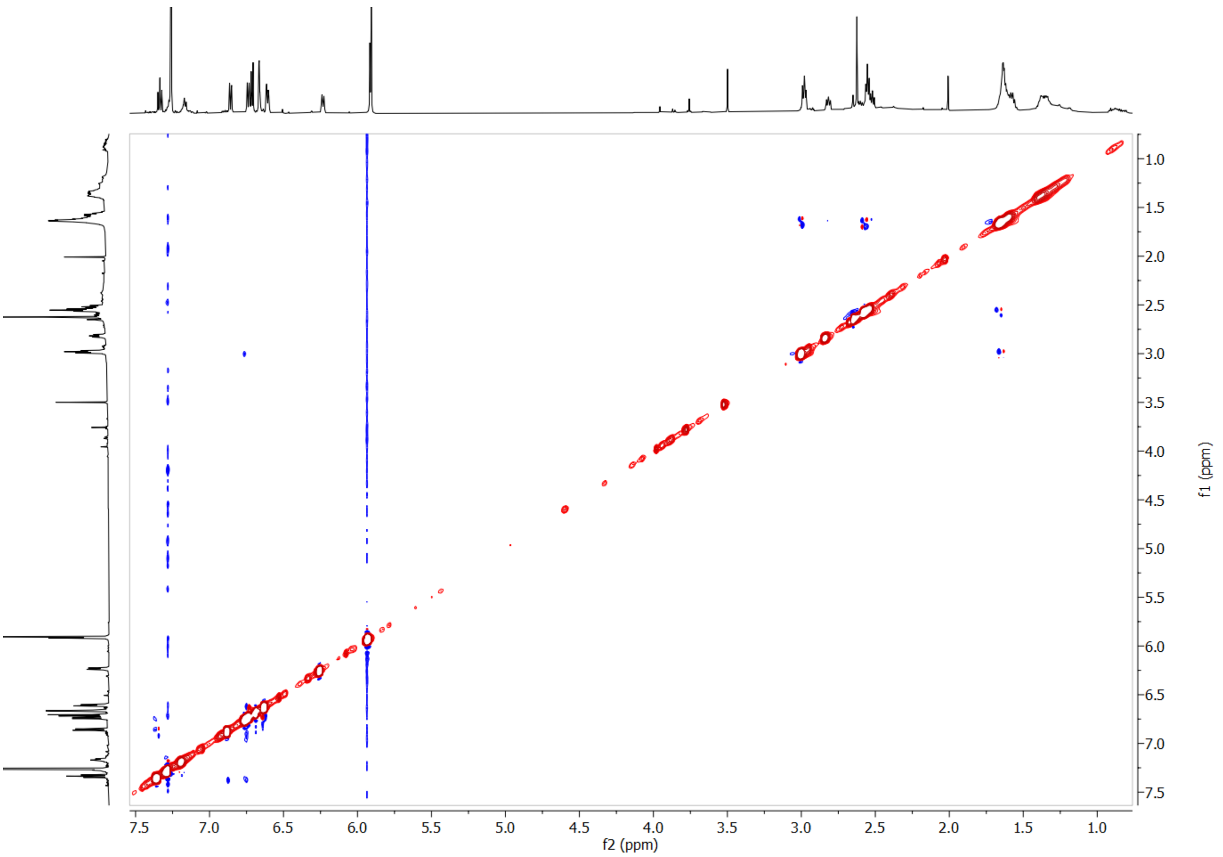

ROESY NMR spectrum of **Knemolic acid A** in $CDCl_3$ at 600 MHz.

4. Knemolone C:

Experimental:

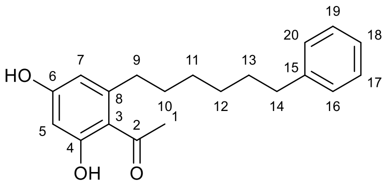

Knemolone C (**4**) Yellow amorphous solid; UV (MeOH) λ_{max} (log ε) 193 (5.10), 217 (4.79), 282 (4.33) nm; ^{1}H NMR ($CDCl_3$, 600 MHz) δ 7.28 (2H, t, J = 7.5 Hz), 7.21 – 7.14 (3H, m), 6.24 (2H, q, J = 2.6 Hz), 2.82 (2H, t, J = 8.0 Hz), 2.62 (3H, s), 2.60 (2H, t, J = 7.7 Hz), 1.67 – 1.55 (4H, m), 1.38 (4H, dp, J = 15.0, 5.7 Hz); ^{13}C NMR ($CDCl_3$, 151 MHz) δ 204.4, 166.1, 161.0, 147.9, 142.7, 128.5, 128.4, 125.8, 115.5, 110.7, 101.8, 36.4, 36.0, 32.3, 32.3, 31.5, 29.7, 29.1 (NP-MRD ID: NP0333018); HRESIMS *m/z* 311.1651 $[M-H]^-$ (calcd for $C_{20}H_{23}O_3^-$ 311.1653, Δ = -0.64 ppm), *m/z* 313.1796 $[M+H]^+$ (calcd for $C_{20}H_{25}O_3^+$ 313.1789, Δ = 2.23 ppm), MS/MS spectrum: CCMSLIB00012475059.

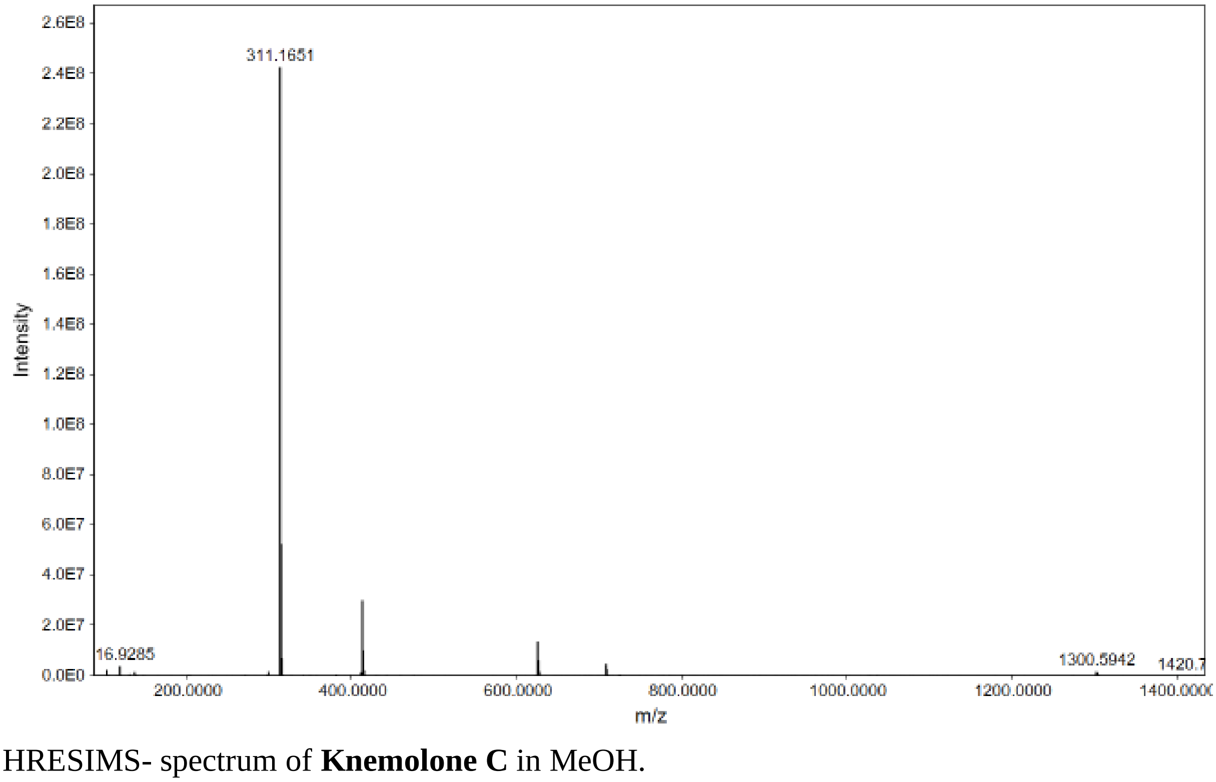

HRESIMS- spectrum of **Knemolone C** in MeOH.

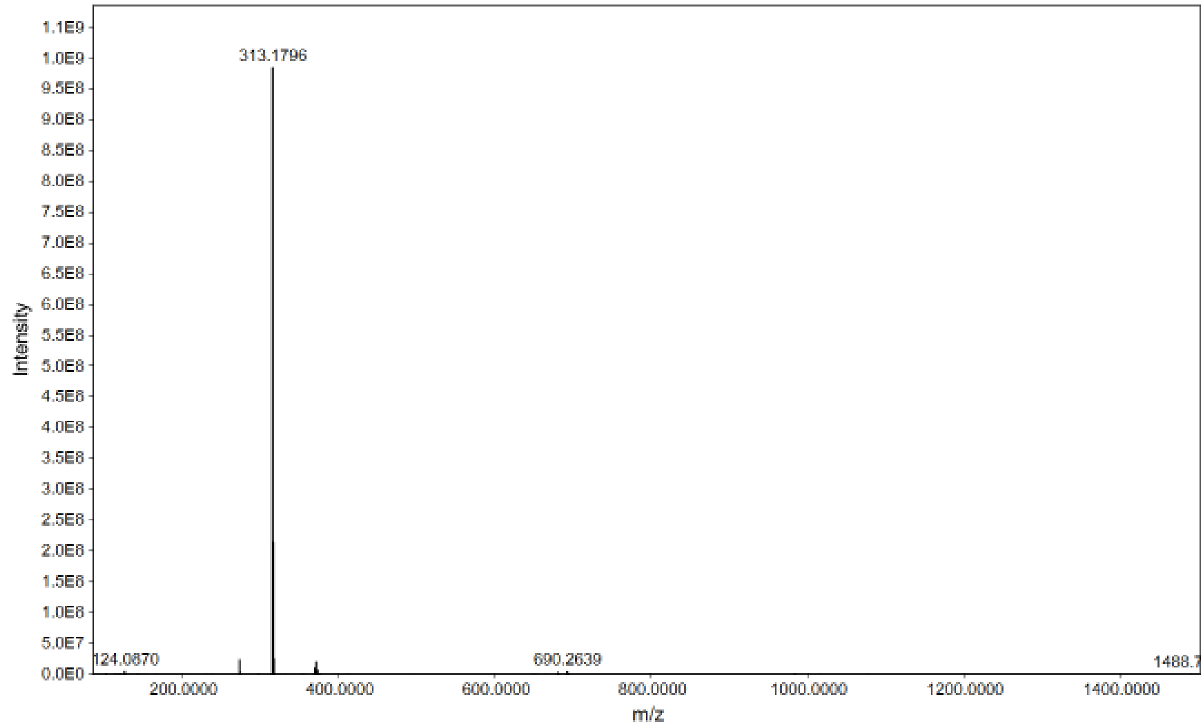

HRESIMS+ spectrum of **Knemolone C** in MeOH.

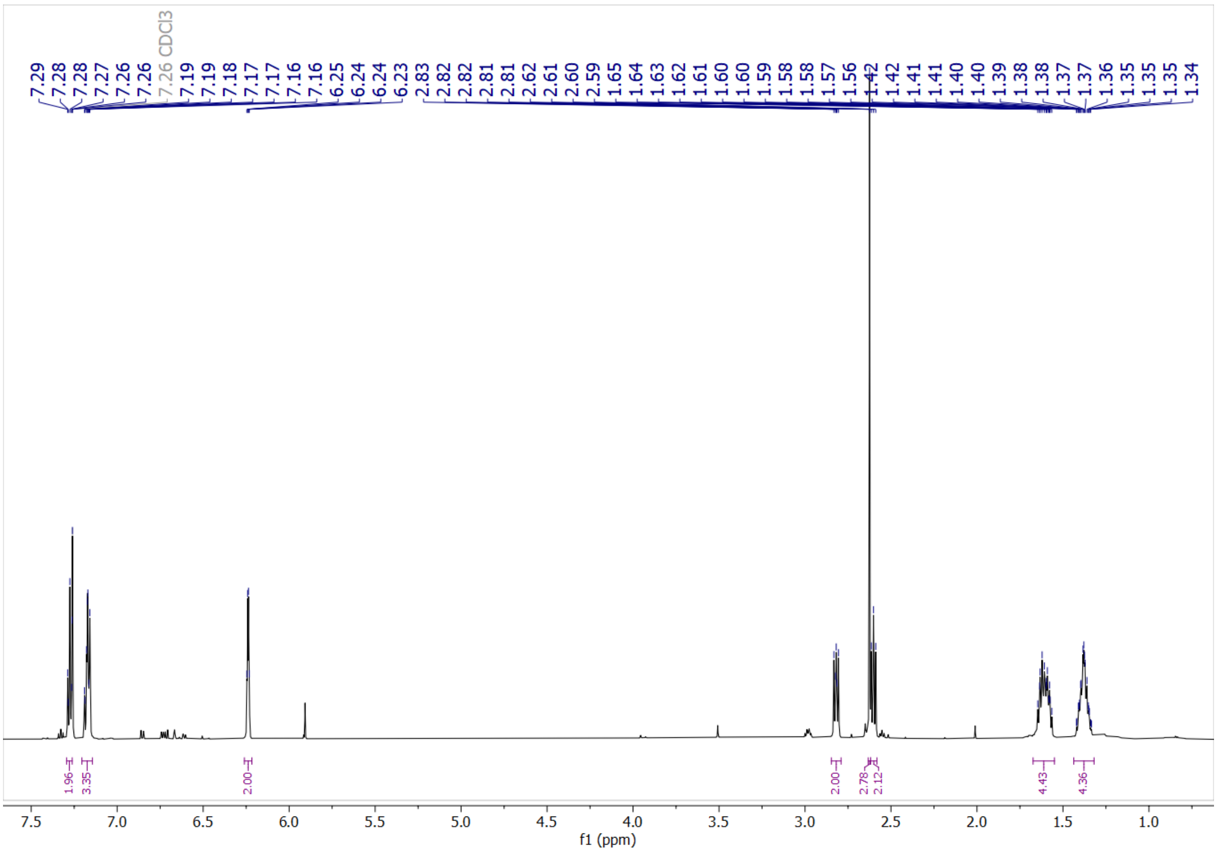

^{1}H NMR spectrum of **Knemolone C** in $CDCl_3$ at 600 MHz.

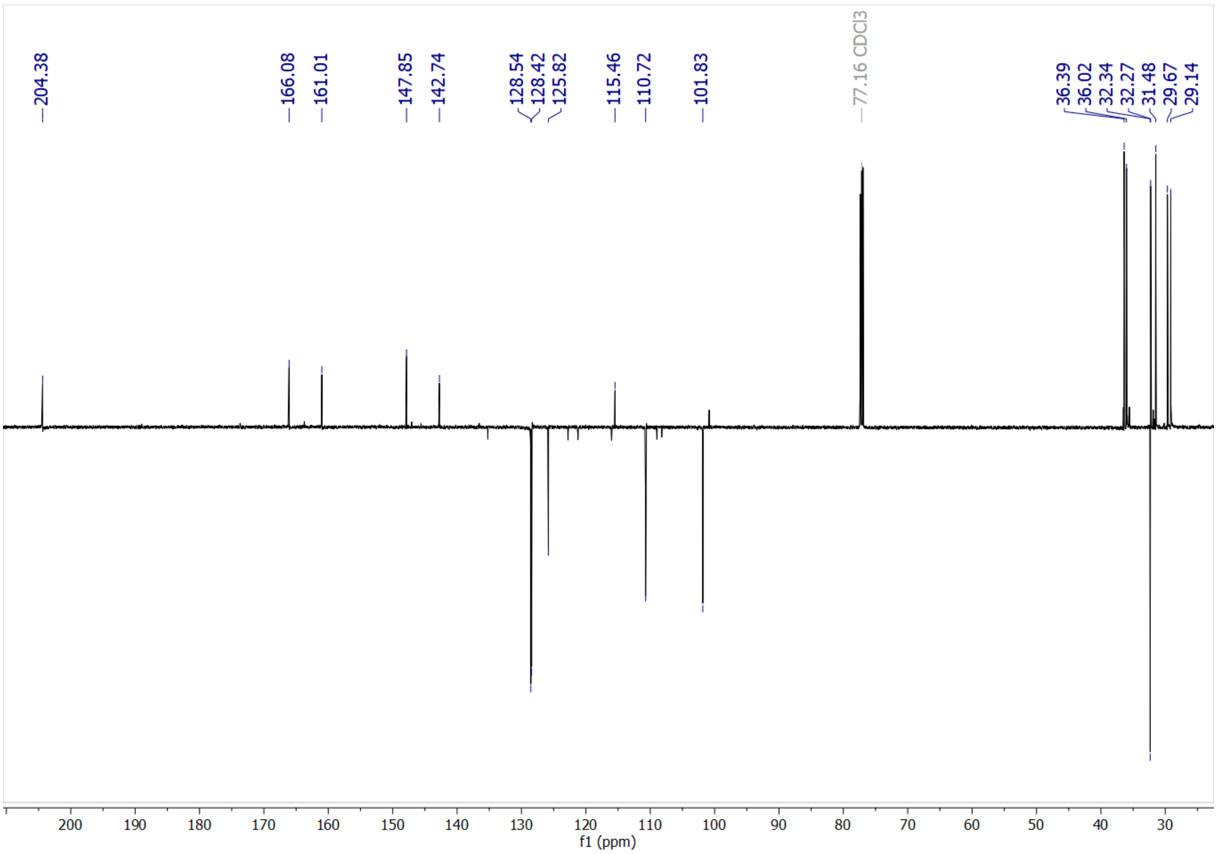

^{13}C DEPTQ NMR spectrum of **Knemolone C** in $CDCl_3$ at 151 MHz.

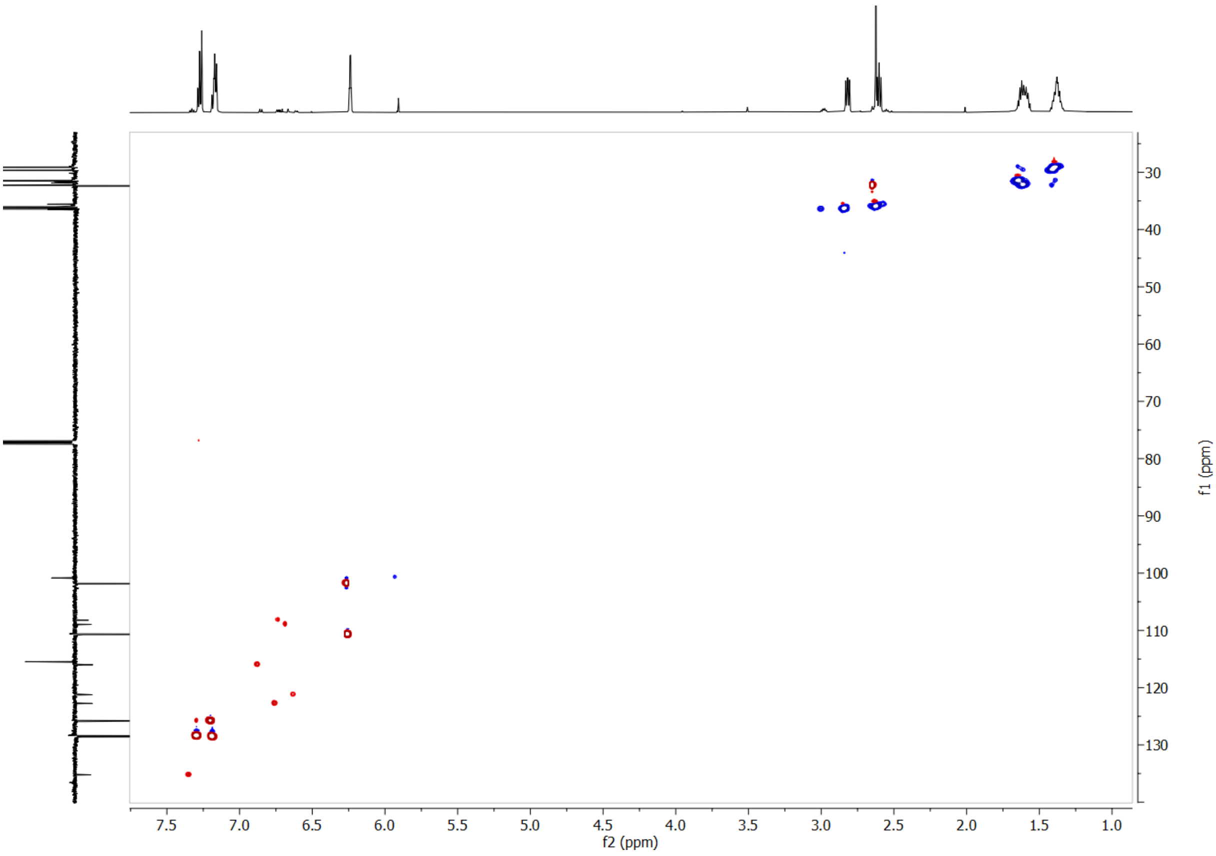

HSQC NMR spectrum of **Knemolone C** in $CDCl_3$ at 600 MHz.

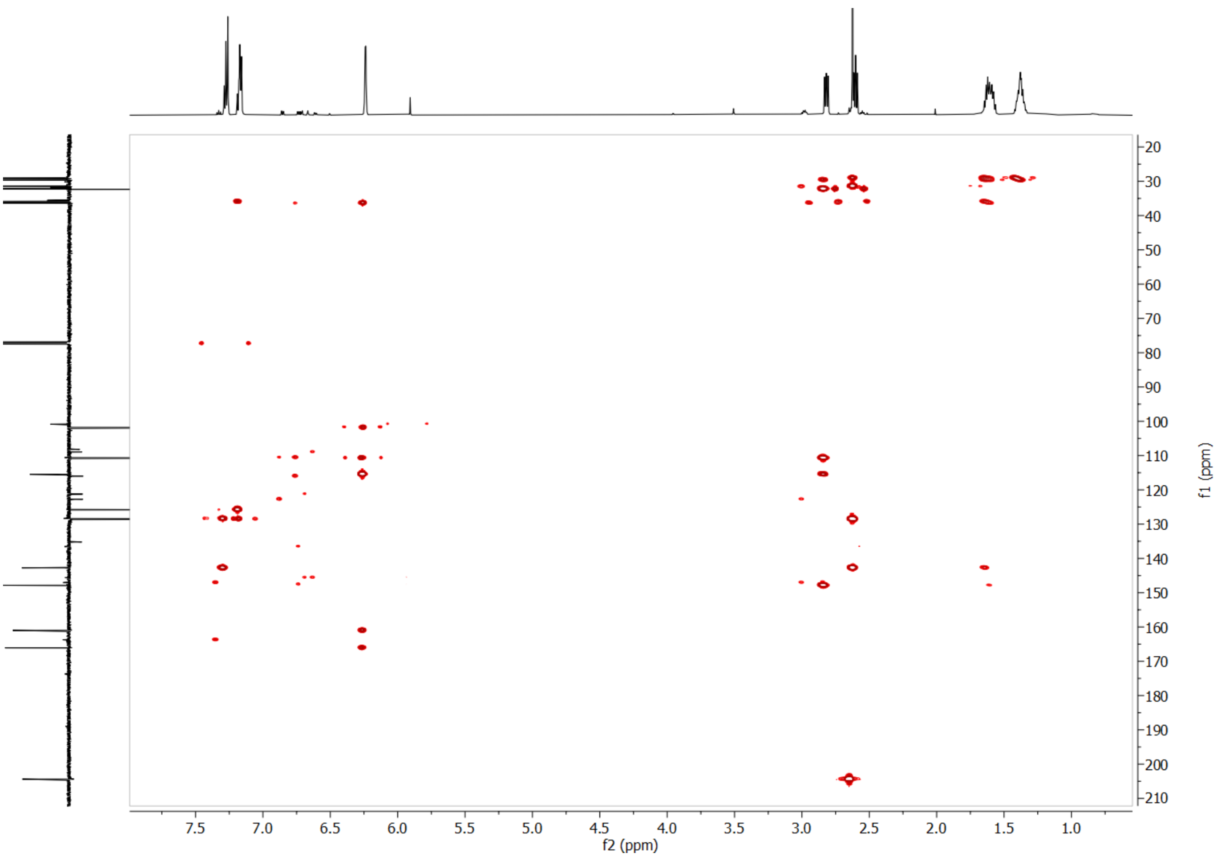

HMBC NMR spectrum of **Knemolone C** in $CDCl_3$ at 600 MHz.

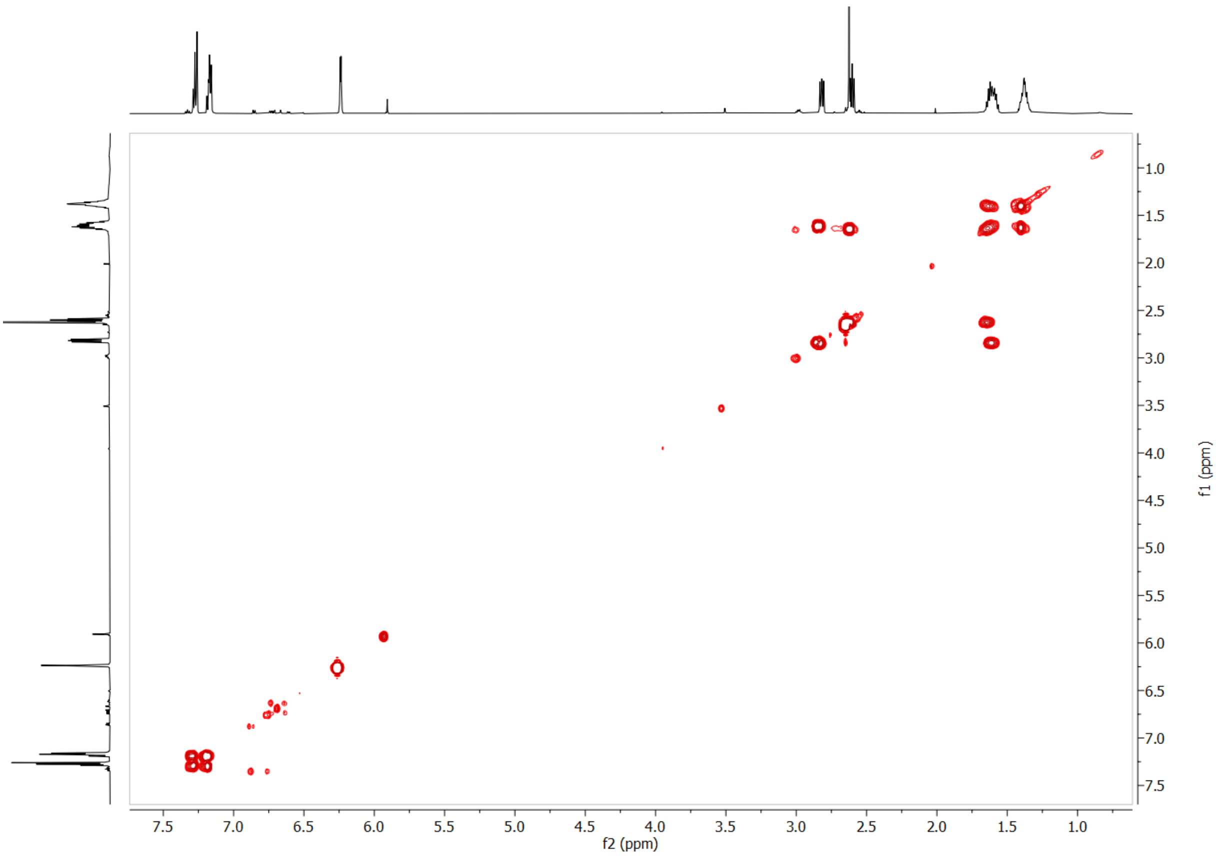

COSY NMR spectrum of **Knemolone C** in $CDCl_3$ at 600 MHz.

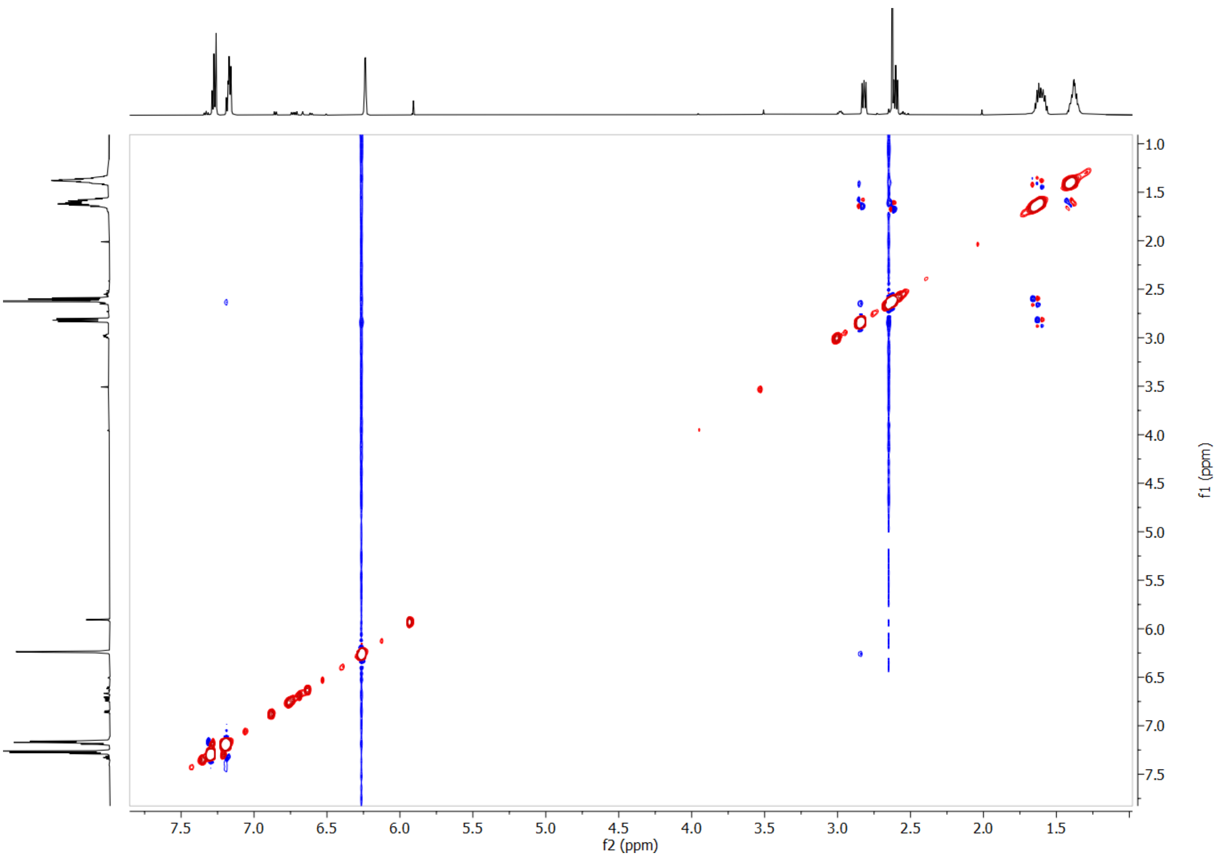

ROESY NMR spectrum of **Knemolone C** in $CDCl_3$ at 600 MHz.

5. Knemolone D:

Experimental:

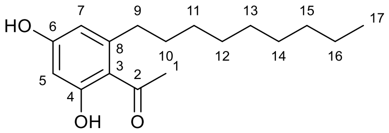

Knemolone D (**5**) Yellow amorphous solid; UV (MeOH) λ_{max} (log ε) 196 (4.17), 222 (4.44), 282 (3.96) nm; ^{1}H NMR ($CDCl_3$, 600 MHz) δ 6.26 (1H, d, J = 2.6 Hz), 6.24 (1H, d, J = 2.6 Hz), 2.83 (2H, t, J = 8.1 Hz), 2.64 (3H, s), 1.59 (2H, p, J = 7.7 Hz), 1.36 (2H, p, J = 6.7 Hz), 1.30 – 1.22 (10H, m), 0.88 (3H, t, J = 6.9 Hz); ^{13}C NMR ($CDCl_3$, 151 MHz) δ 204.4, 166.1, 160.9, 148.0, 115.5, 110.7, 101.8, 36.5, 32.4, 32.3, 32.0, 29.9, 29.6, 29.6, 29.4, 22.8, 14.2 (NP-MRD ID: NP0333019); HRESIMS *m/z* 277.1808 [M-H]$^-$ (calcd for $C_{17}H_{25}O_3^-$ 277.1809, Δ = -0.36 ppm), *m/z* 279.1949 [M+H]$^+$ (calcd for $C_{17}H_{27}O_3^+$ 279.1955, Δ = -2.15 ppm), MS/MS spectrum: CCMSLIB00012475056.

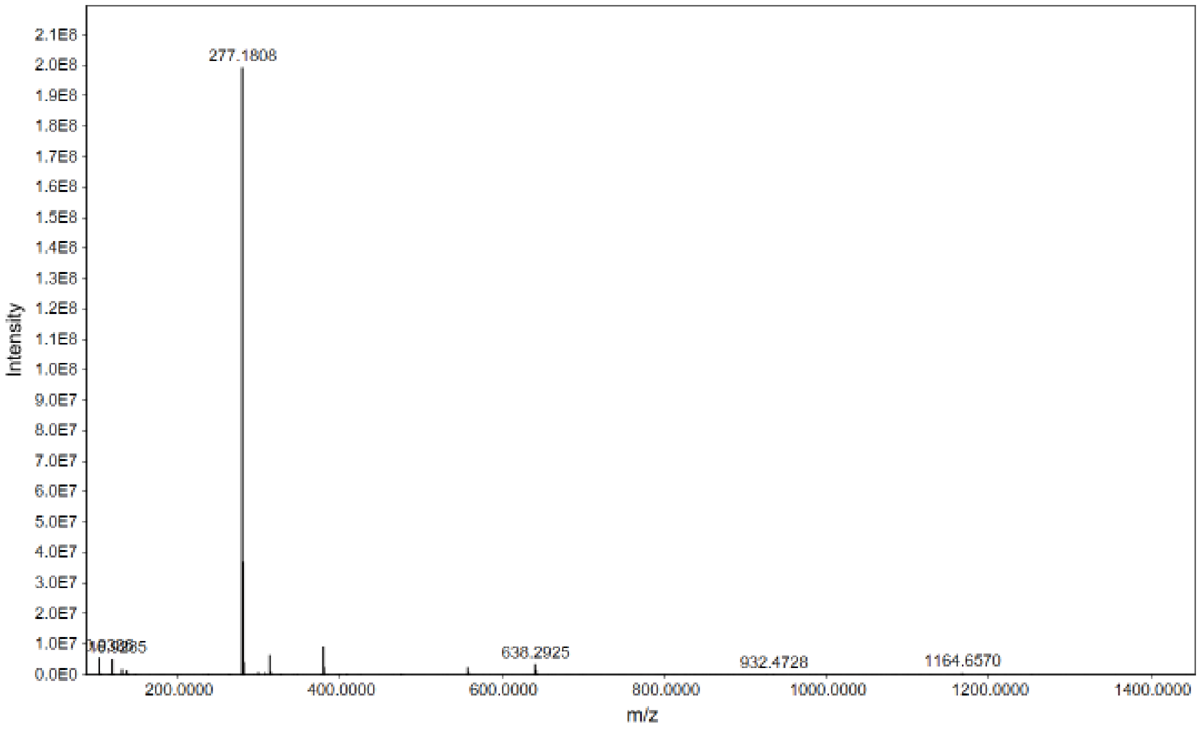

HRESIMS- spectrum of **Knemolone D** in MeOH.

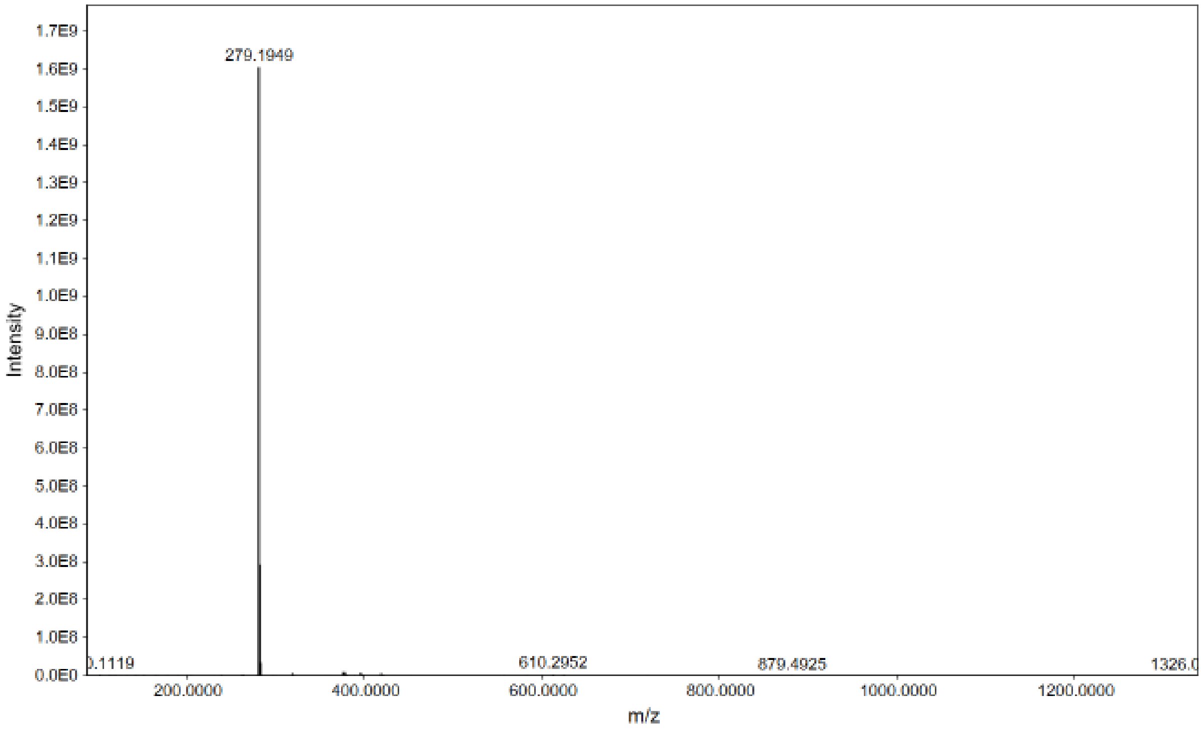

HRESIMS+ spectrum of **Knemolone D** in MeOH.

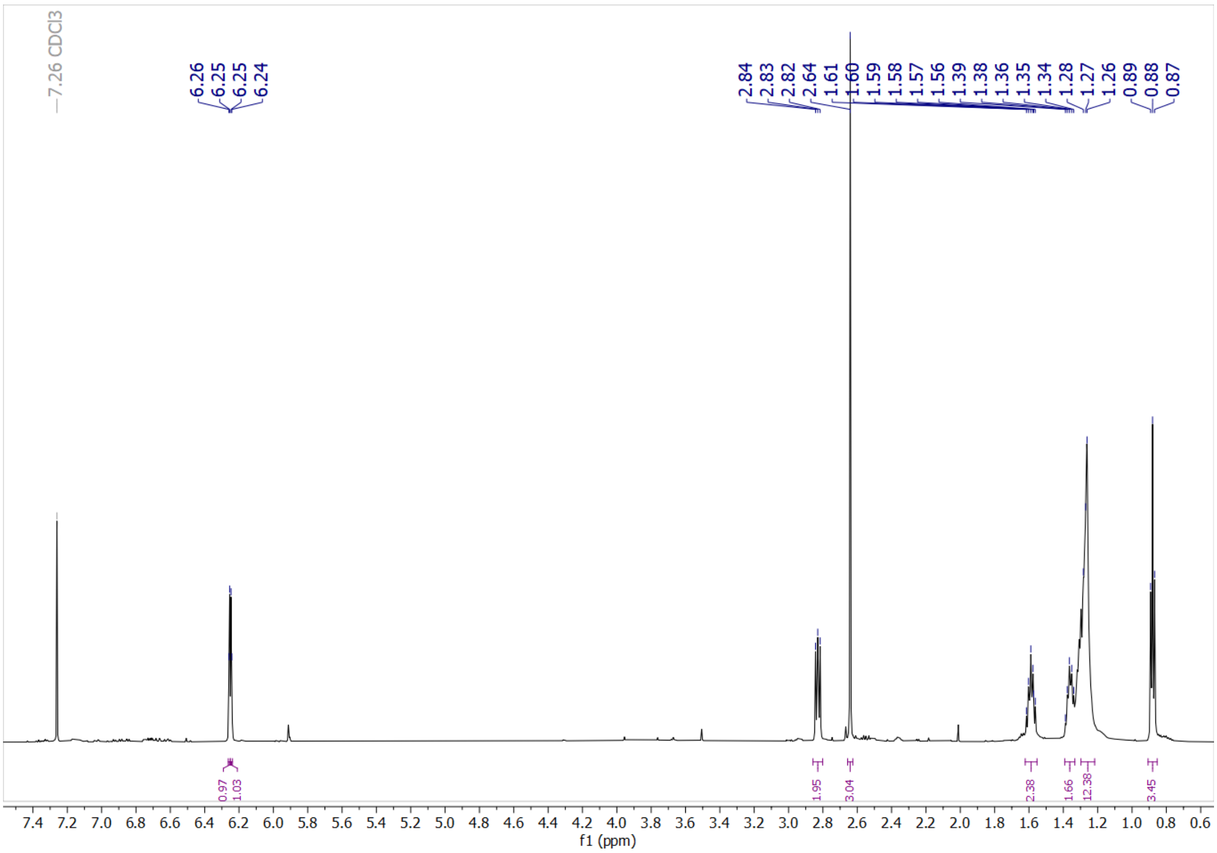

^{1}H NMR spectrum of **Knemolone D** in $CDCl_3$ at 600 MHz.

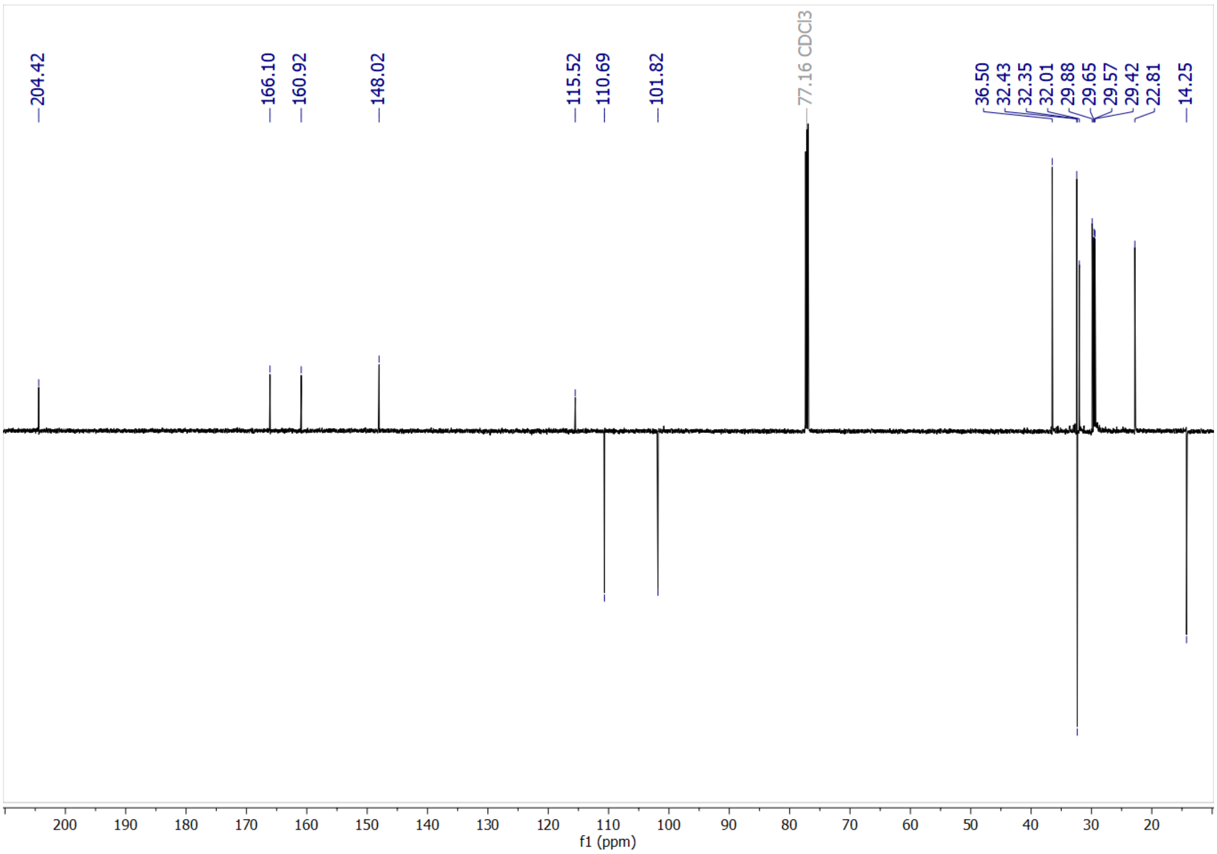

^{13}C DEPTQ NMR spectrum of **Knemolone D** in $CDCl_3$ at 151 MHz.

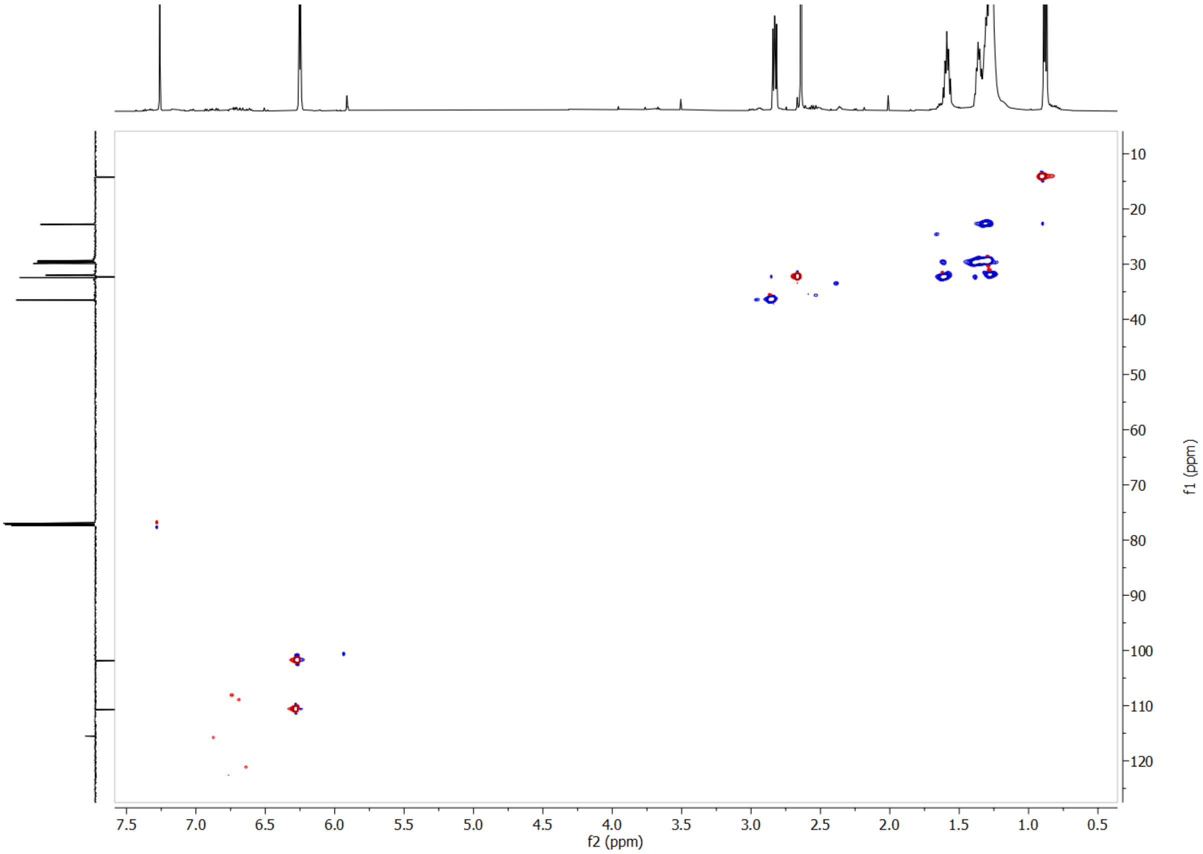

HSQC NMR spectrum of **Knemolone D** in $CDCl_3$ at 600 MHz.

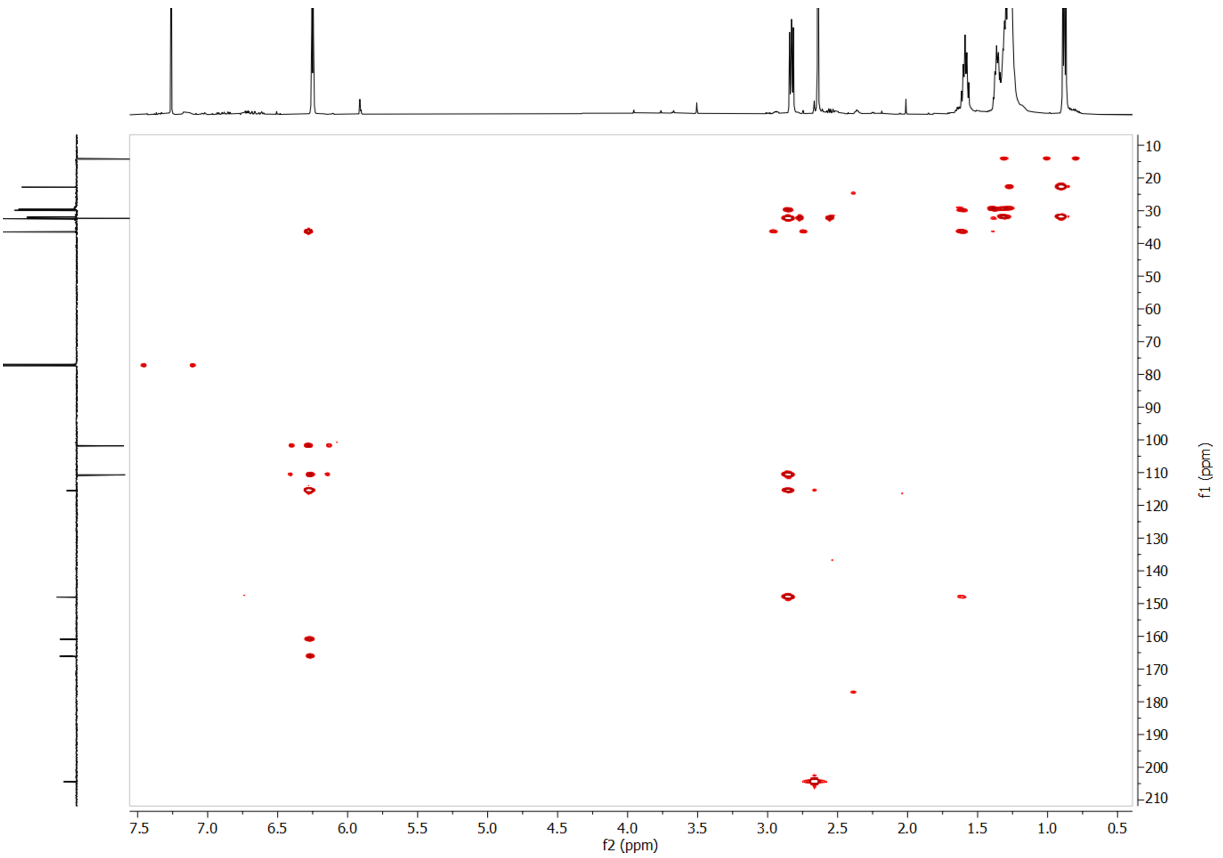

HMBC NMR spectrum of **Knemolone D** in $CDCl_3$ at 600 MHz.

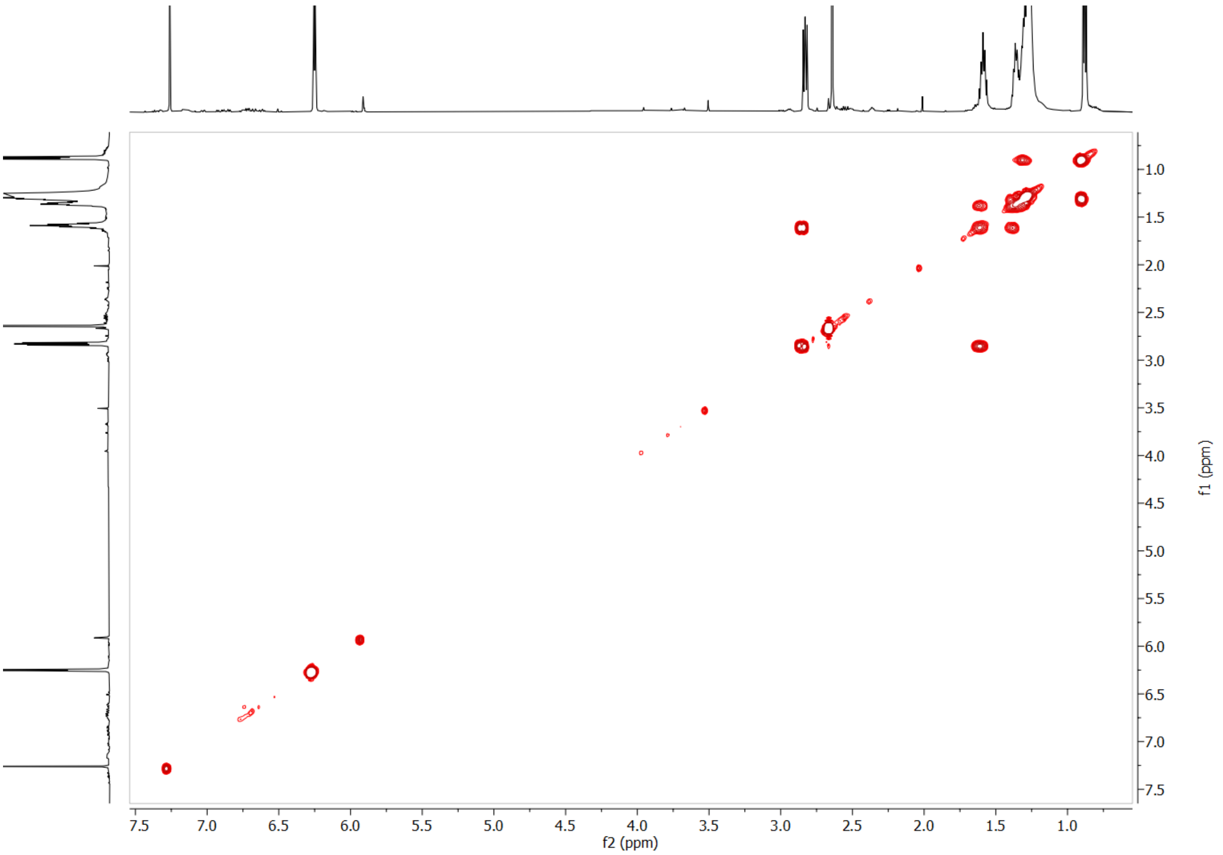

COSY NMR spectrum of **Knemolone D** in $CDCl_3$ at 600 MHz.

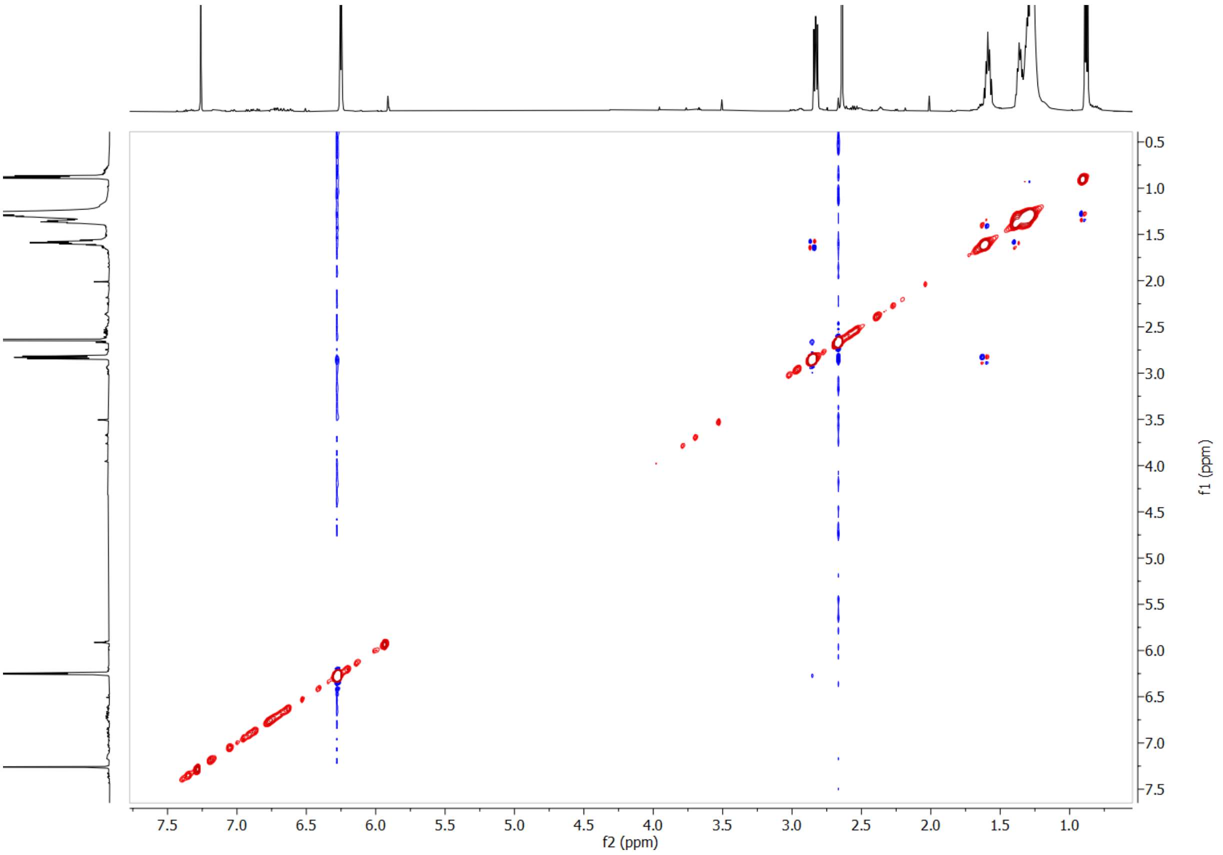

ROESY NMR spectrum of **Knemolone D** in $CDCl_3$ at 600 MHz.

6. Knemolic acid B:

Experimental:

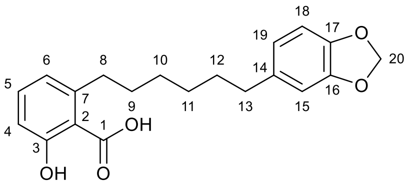

Knemolic acid B (**6**) Yellow-green amorphous solid; UV (MeOH) λ_{max} (log ε) 196 (5.11), 214 (5.08), 233 (4.82), 288 (4.57), 310 (4.33) nm; ^{1}H NMR ($CDCl_3$, 600 MHz) δ 7.36 (1H, t, J = 7.9 Hz), 6.87 (1H, dd, J = 8.3, 1.2 Hz), 6.76 (1H, dd, J = 7.5, 1.2 Hz), 6.71 (1H, d, J = 7.8 Hz), 6.67 (1H, d, J = 1.8 Hz), 6.61 (1H, dd, J = 7.9, 1.7 Hz), 5.90 (2H, s), 2.97 (2H, t, J = 8.0 Hz), 2.52 (2H, t, J = 7.7 Hz), 1.59 (4H, dp, J = 14.7, 7.3 Hz), 1.37 (4H, dp, J = 13.2, 3.7 Hz); ^{13}C NMR ($CDCl_3$, 151 MHz) δ 175.9, 163.8, 147.8, 147.6, 145.5, 136.8, 135.6, 122.9, 121.2, 116.0, 110.5, 109.0, 108.2, 100.8, 36.6, 35.8, 32.0, 31.8, 29.7, 29.1 (NP-MRD ID: NP0333020); HRESIMS *m/z* 341.1391 $[M-H]^-$ (calcd for $C_{20}H_{21}O_5^-$ 341.1394, Δ = -0.88 ppm), *m/z* 325.1430 $[M-H_2O+H]^+$ (calcd for $C_{20}H_{21}O_4^+$ 325.1434, Δ = -1.23 ppm), MS/MS spectrum: CCMSLIB00012475058.

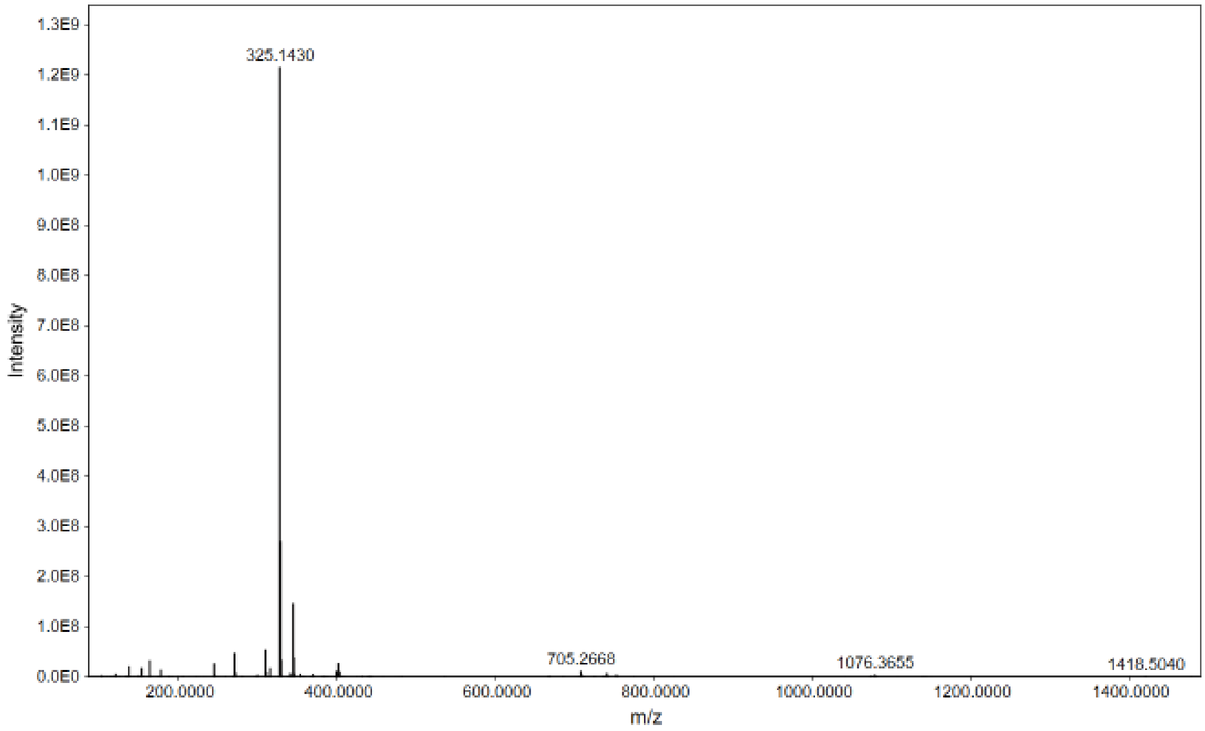

HRESIMS- spectrum of **Knemolic acid B** in MeOH.

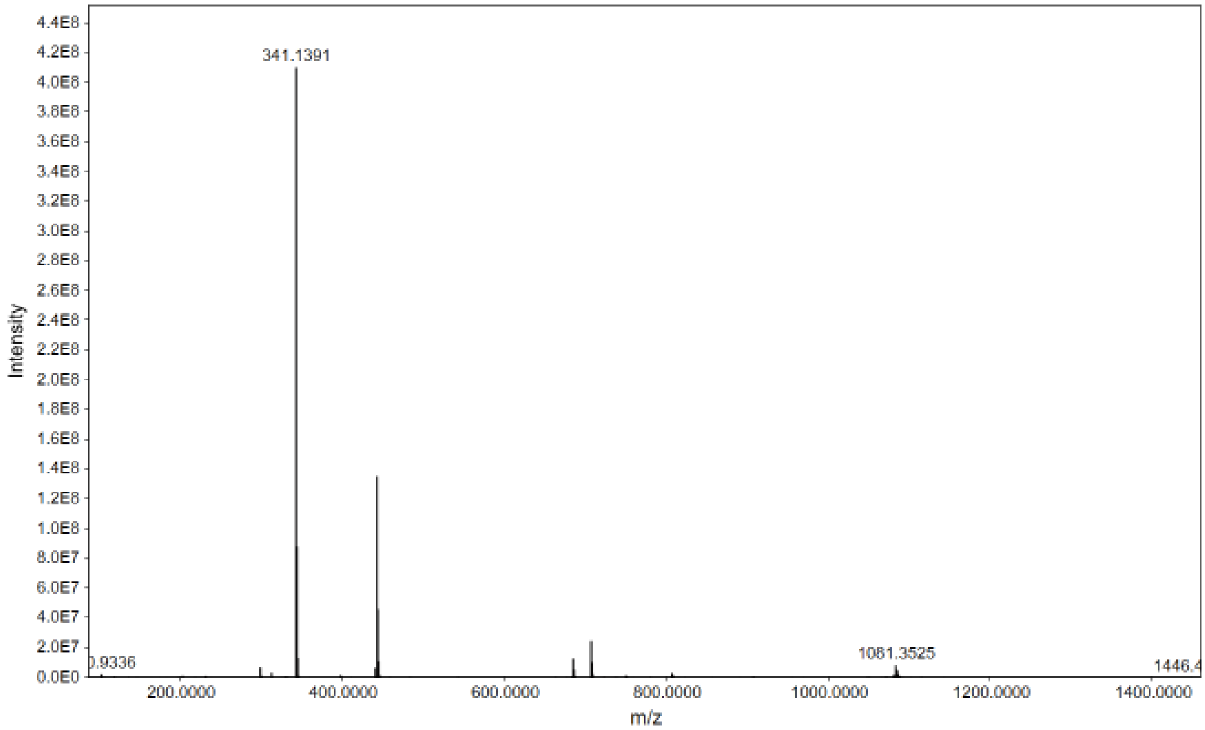

HRESIMS+ spectrum of **Knemolic acid B** in MeOH.

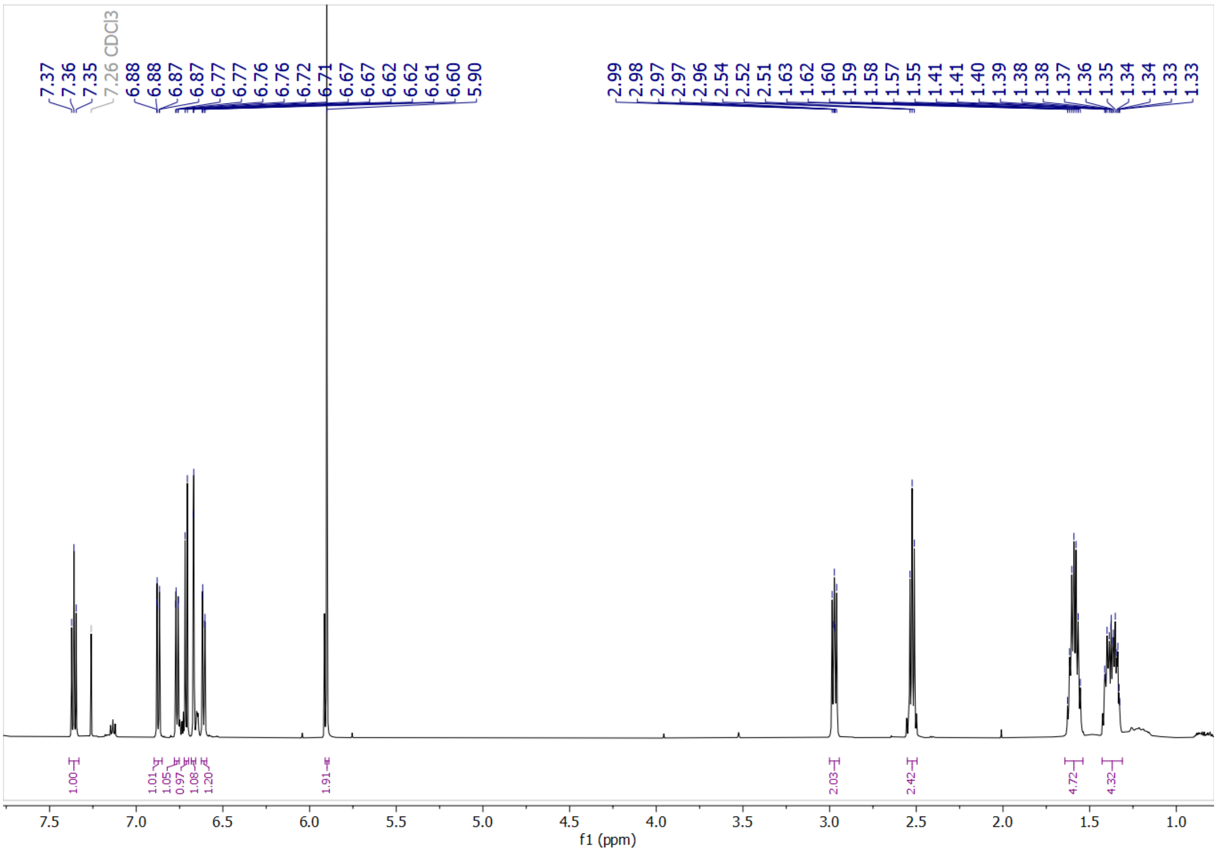

^{1}H NMR spectrum of **Knemolic acid B** in $CDCl_3$ at 600 MHz.

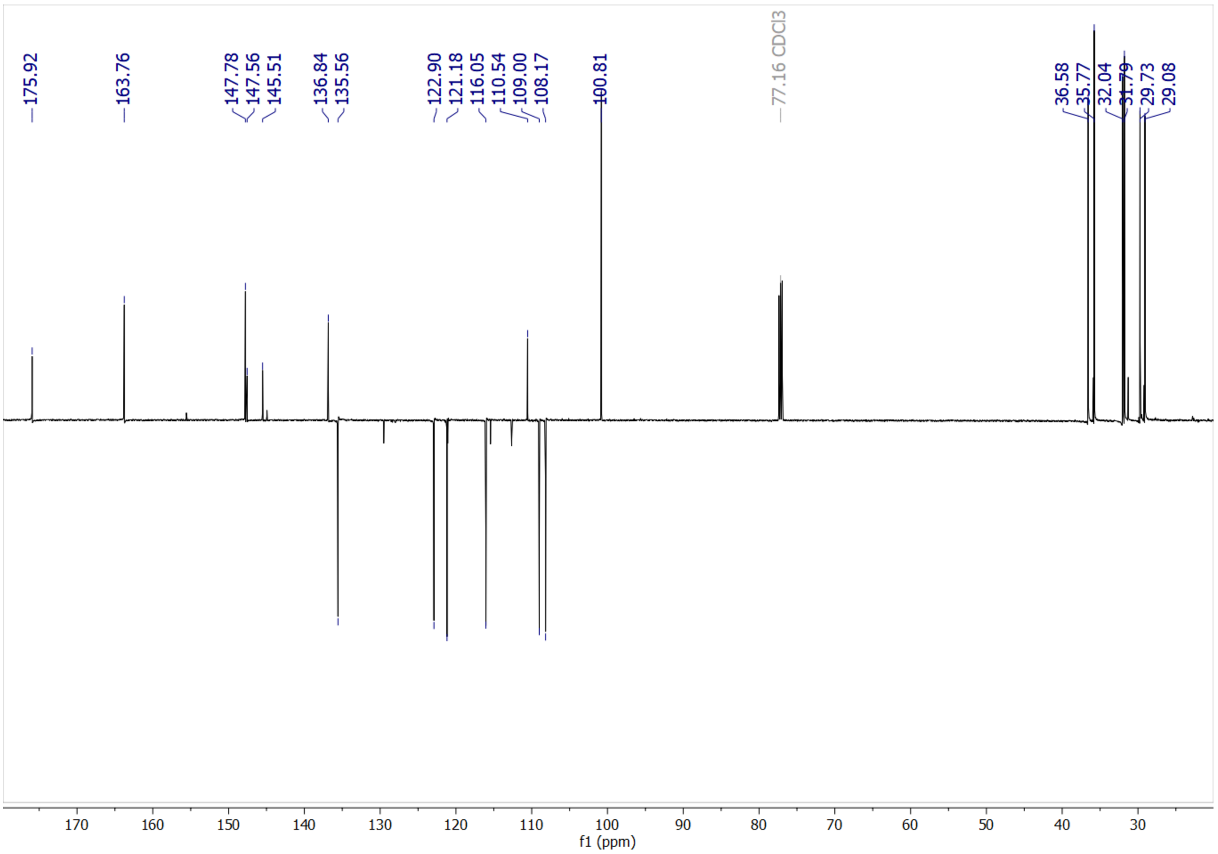

^{13}C DEPTQ NMR spectrum of **Knemolic acid B** in $CDCl_3$ at 151 MHz.

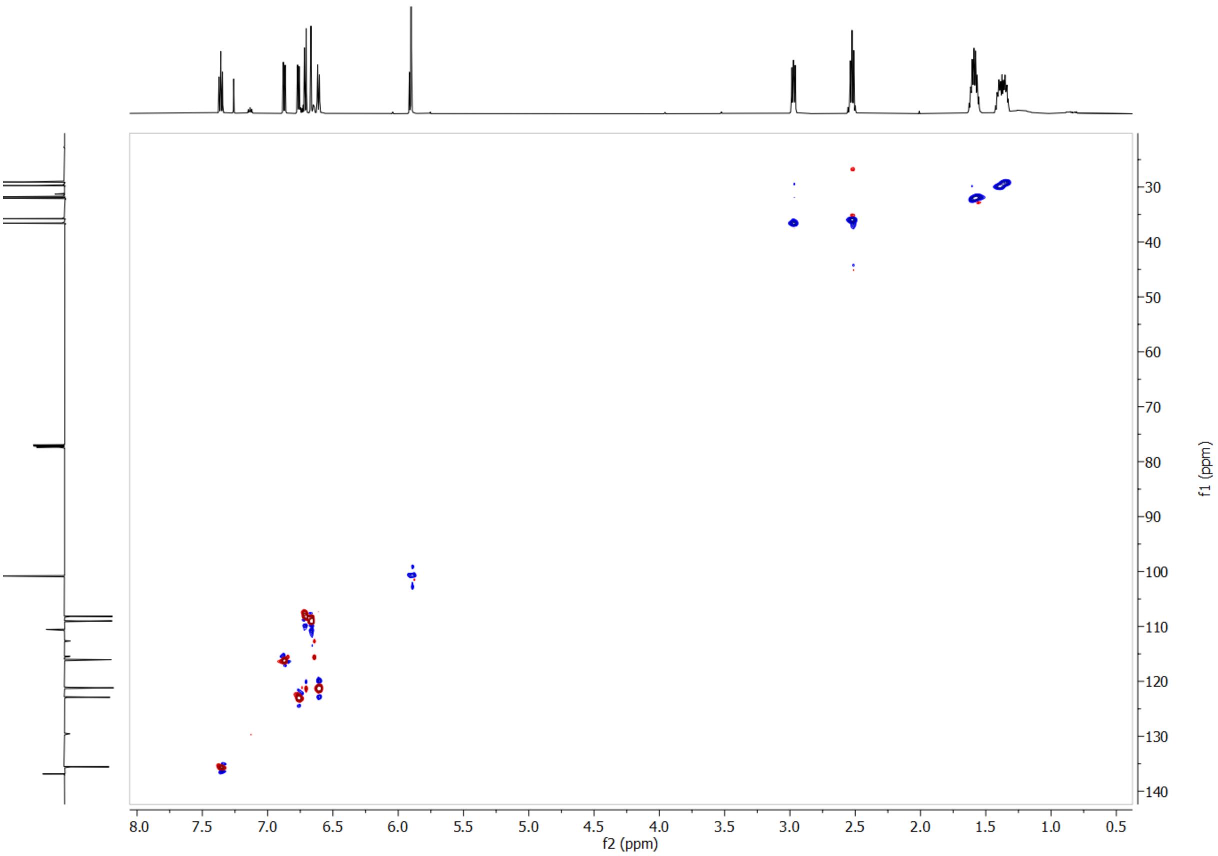

HSQC NMR spectrum of **Knemolic acid B** in $CDCl_3$ at 600 MHz.

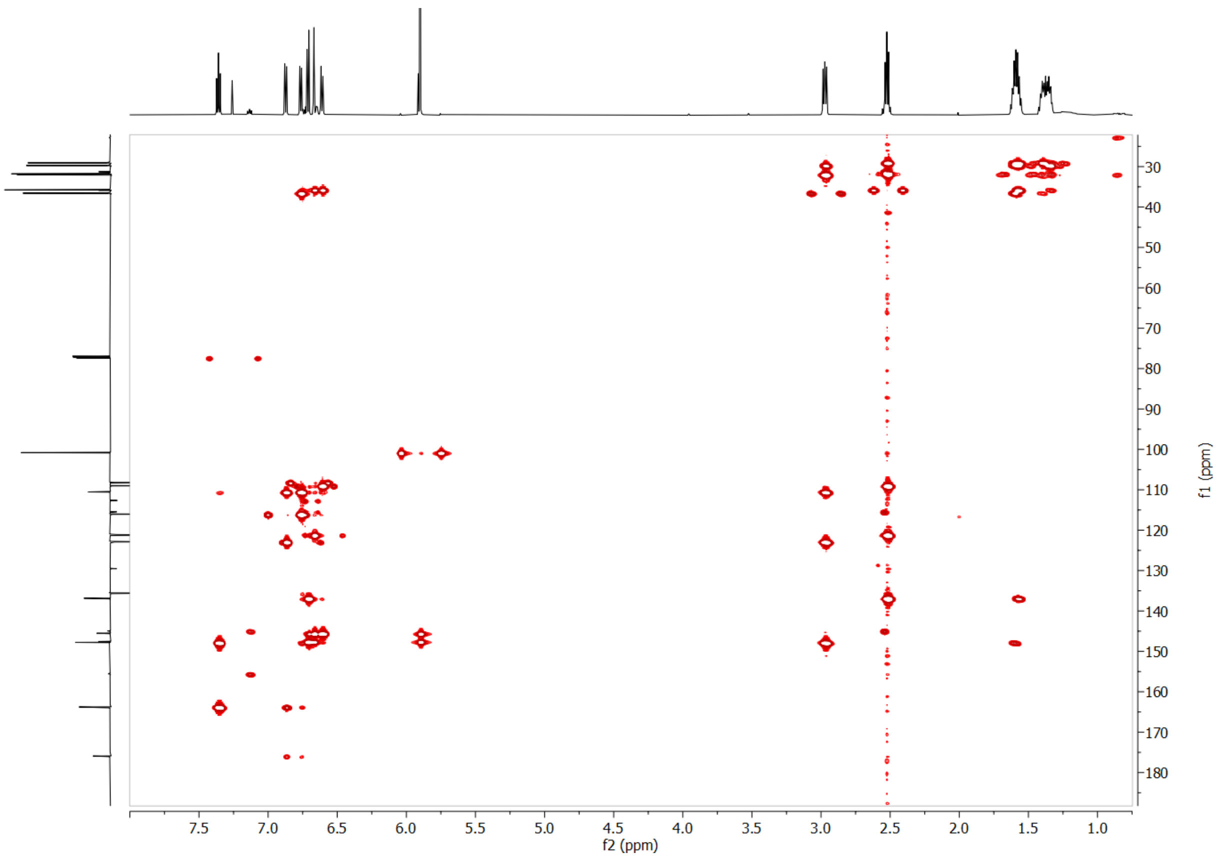

HMBC NMR spectrum of **Knemolic acid B** in $CDCl_3$ at 600 MHz.

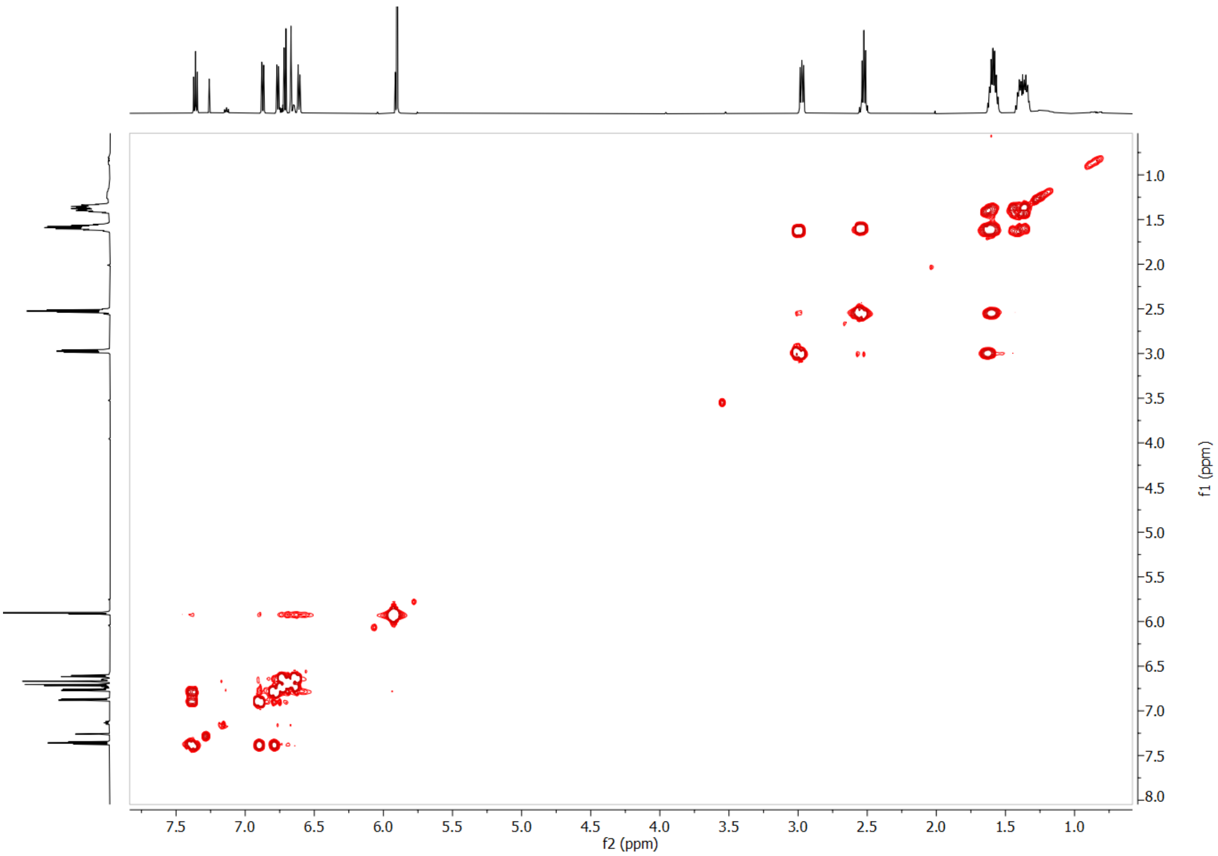

COSY NMR spectrum of **Knemolic acid B** in $CDCl_3$ at 600 MHz.

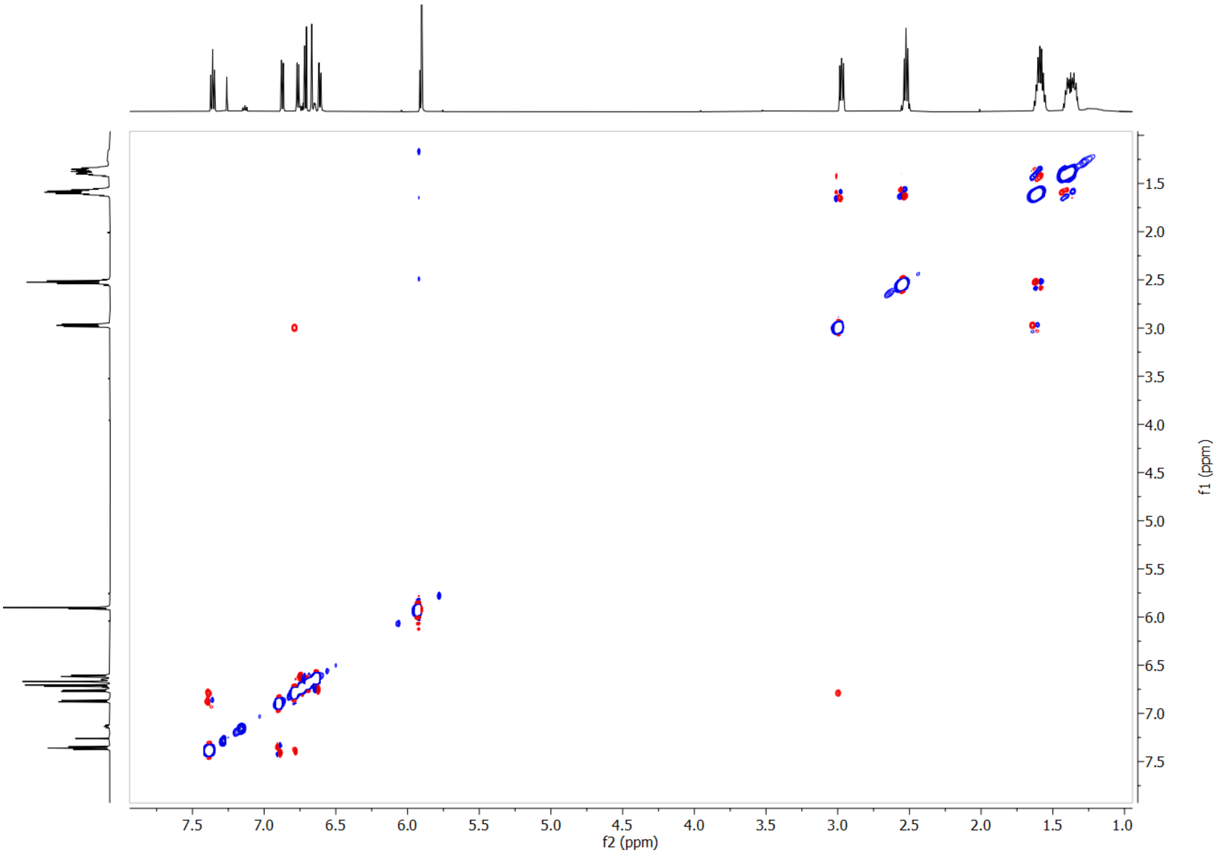

ROESY NMR spectrum of **Knemolic acid B** in $CDCl_3$ at 600 MHz.

7. Knemolic acid C:

Experimental:

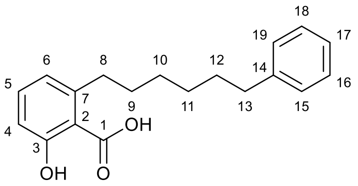

Knemolic acid C (**7**) Yellow-green amorphous solid; UV (MeOH) λ_{max} (log ε) 193 (5.01), 210 (4.94), 240 (4.33), 310 (3.98) nm; ^{1}H NMR ($CDCl_3$, 600 MHz) δ 7.36 (1H, t, J = 7.5 Hz), 7.27 (2H, dd, J = 8.3, 7.0 Hz), 7.20 – 7.15 (3H, m), 6.87 (1H, dd, J = 8.3, 1.2 Hz), 6.76 (1H, dd, J = 7.5, 1.3 Hz), 2.97 (2H, t, J = 8.0 Hz), 2.61 (2H, t, J = 7.7 Hz), 1.62 (4H, dp, J = 15.3, 7.9 Hz), 1.40 (4H, dp, J = 10.6, 3.2 Hz); ^{13}C NMR ($CDCl_3$, 151 MHz) δ 175.8, 163.8, 147.8, 147.7, 143.0, 135.5, 128.6, 128.4, 125.7, 122.9, 116.0, 110.5, 36.6, 36.1, 32.0, 31.6, 29.8, 29.2 (NP-MRD ID: NP0333021); HRESIMS *m/z* 297.1495 [M-H]$^-$ (calcd for $C_{19}H_{21}O_3^-$ 297.1496, Δ = -0.34 ppm), *m/z* 281.1530 [M-H_2O+H]$^+$ (calcd for $C_{19}H_{21}O_2^+$ 281.1536, Δ = -2.13 ppm), MS/MS spectrum: CCMSLIB00012475057.

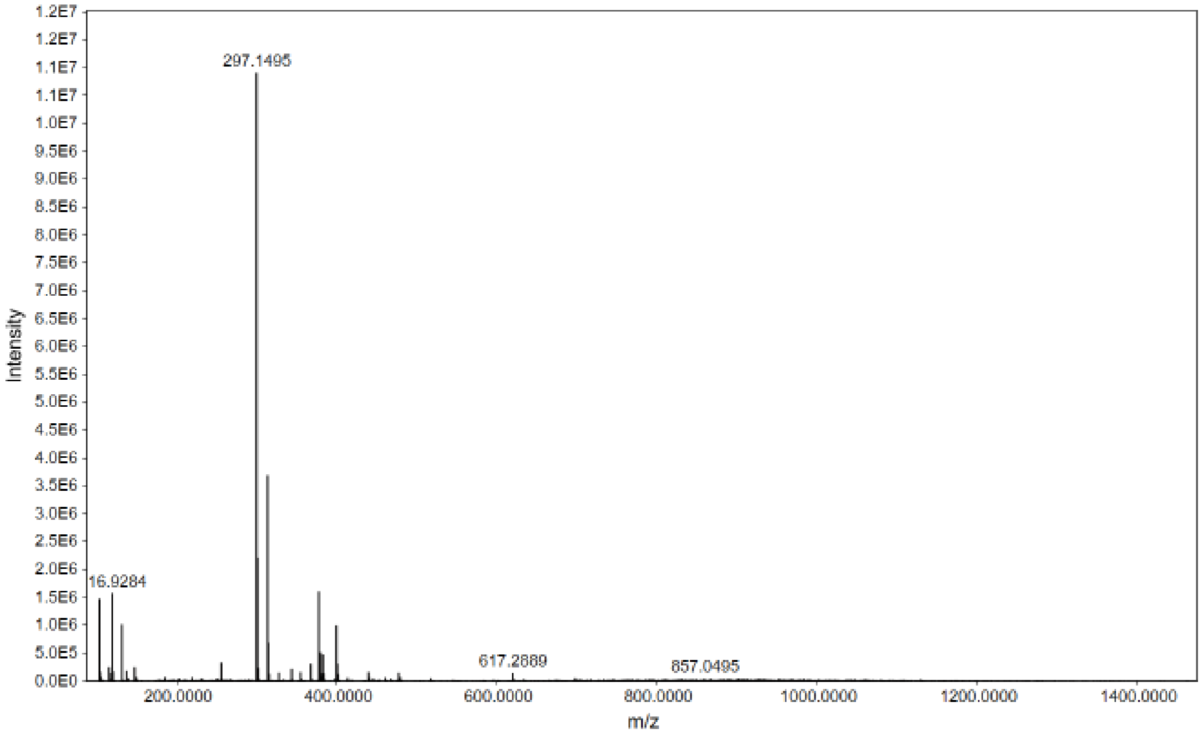

HRESIMS- spectrum of **Knemolic acid C** in MeOH.

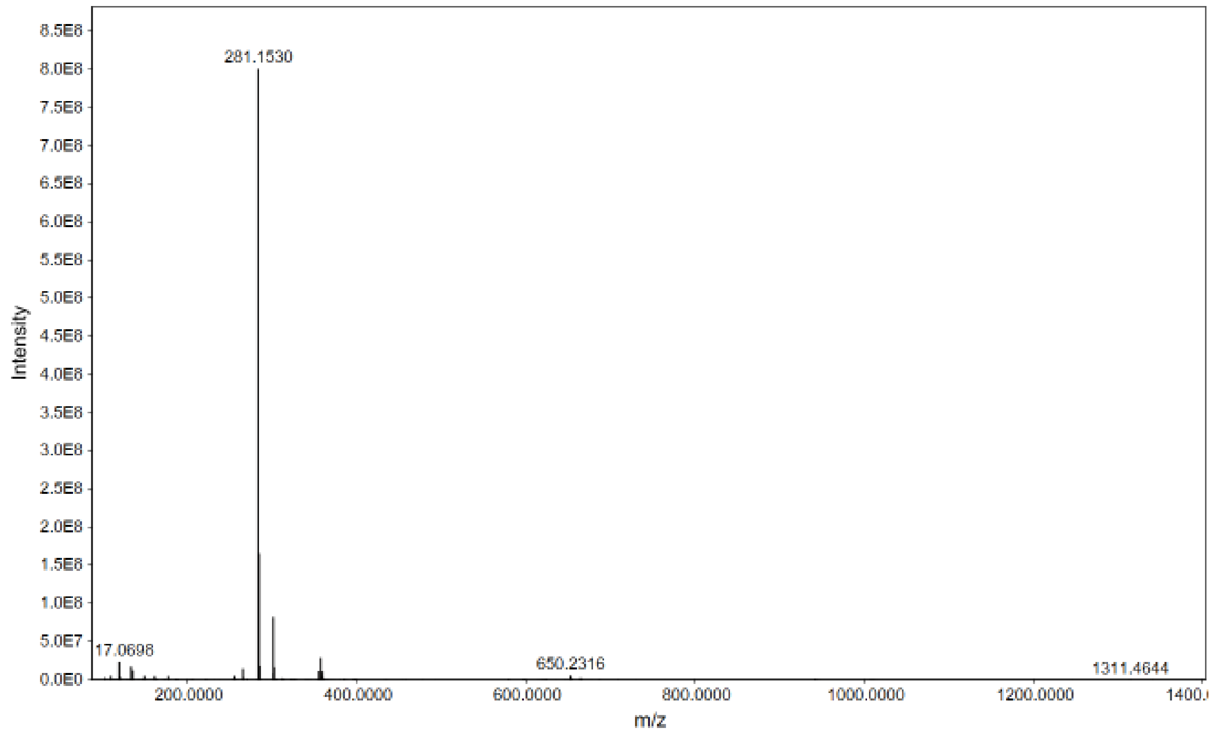

HRESIMS+ spectrum of **Knemolic acid C** in MeOH.

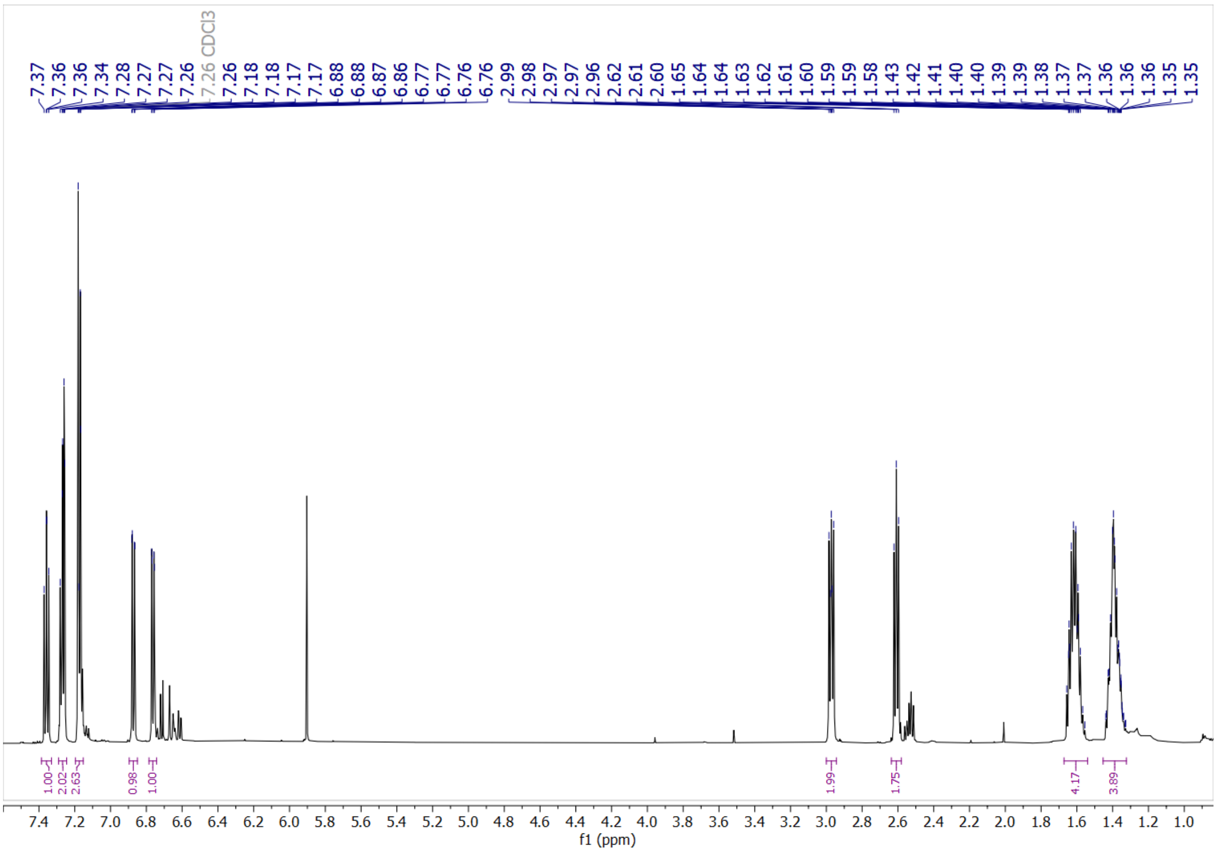

^{1}H NMR spectrum of **Knemolic acid C** in $CDCl_3$ at 600 MHz.

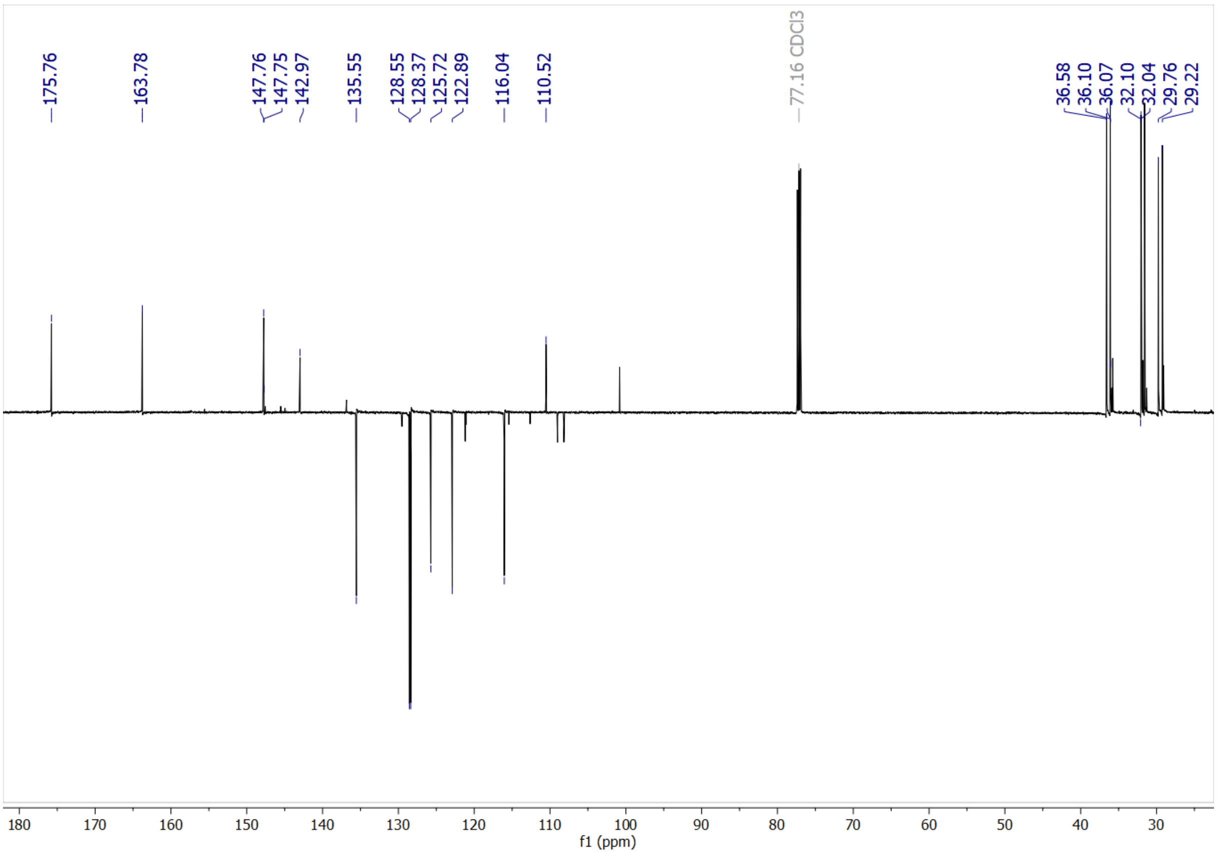

^{13}C DEPTQ NMR spectrum of **Knemolic acid C** in $CDCl_3$ at 151 MHz.

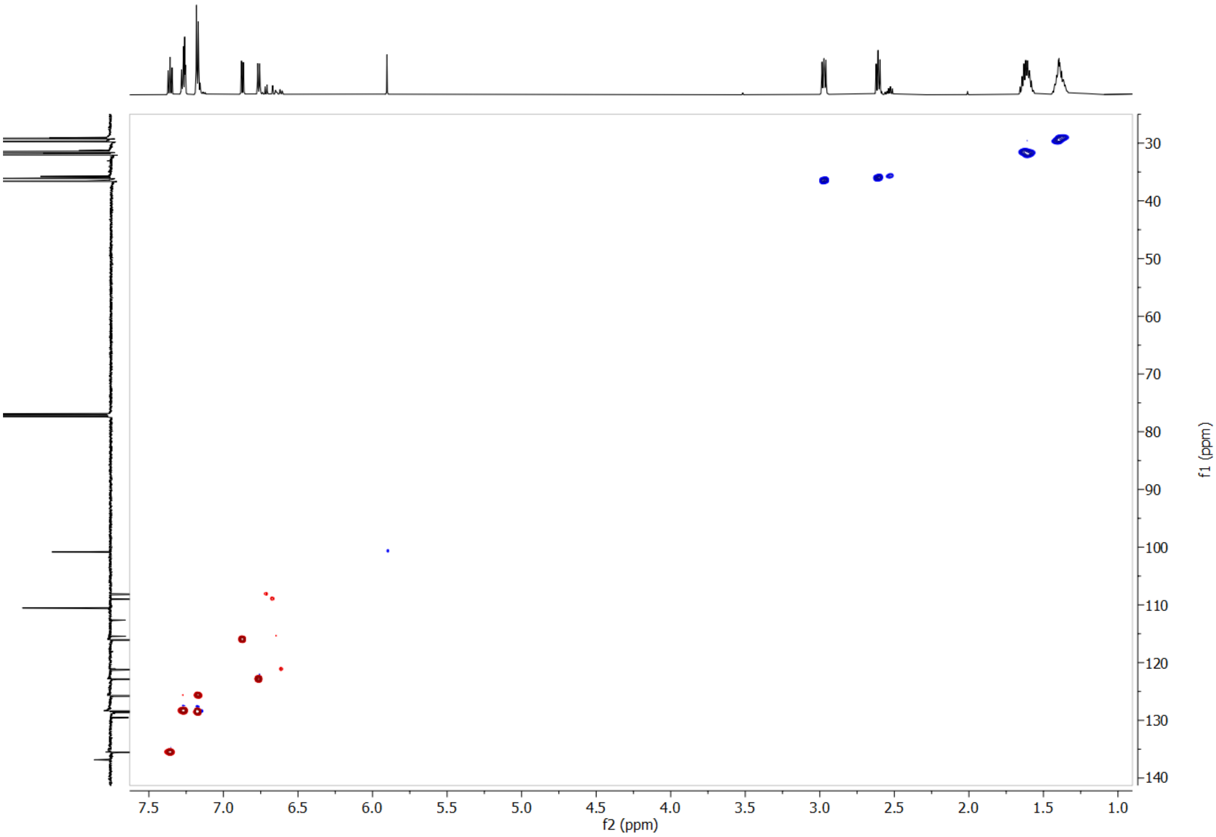

HSQC NMR spectrum of **Knemolic acid C** in $CDCl_3$ at 600 MHz.

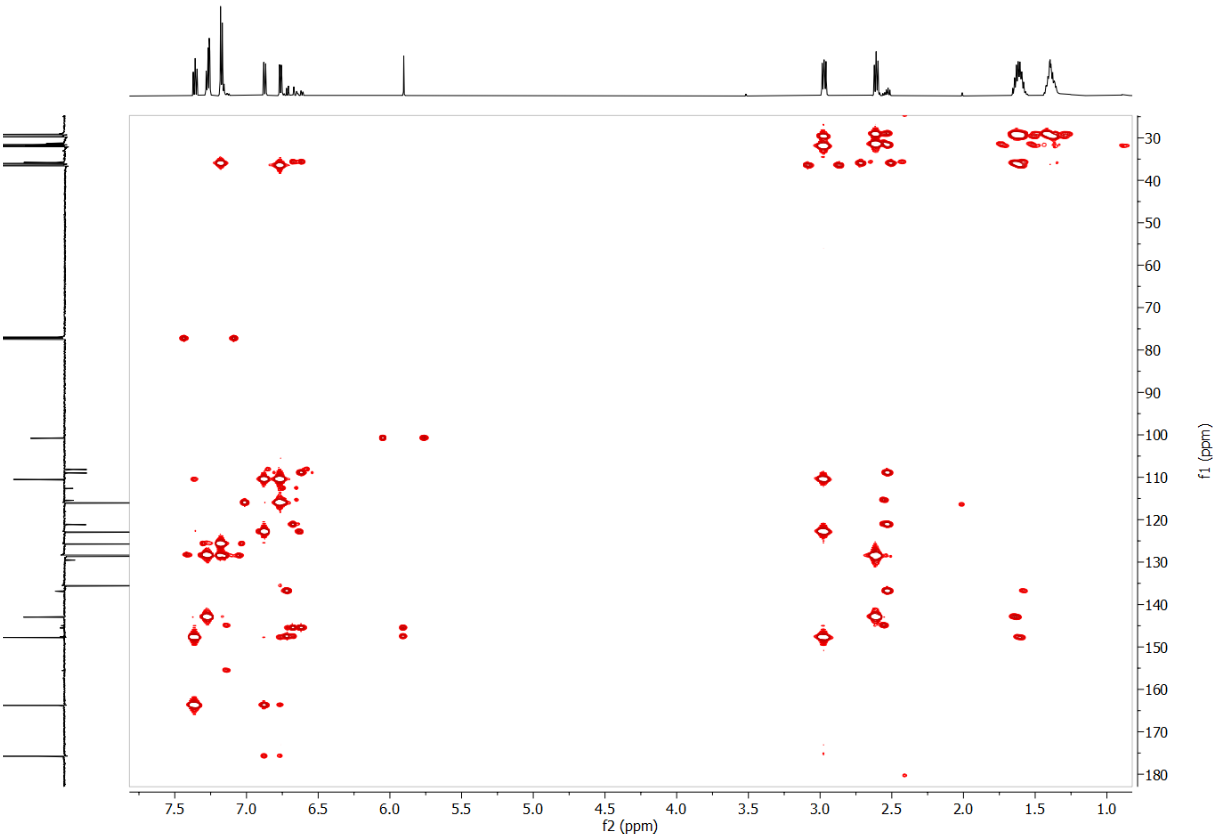

HMBC NMR spectrum of **Knemolic acid C** in $CDCl_3$ at 600 MHz.

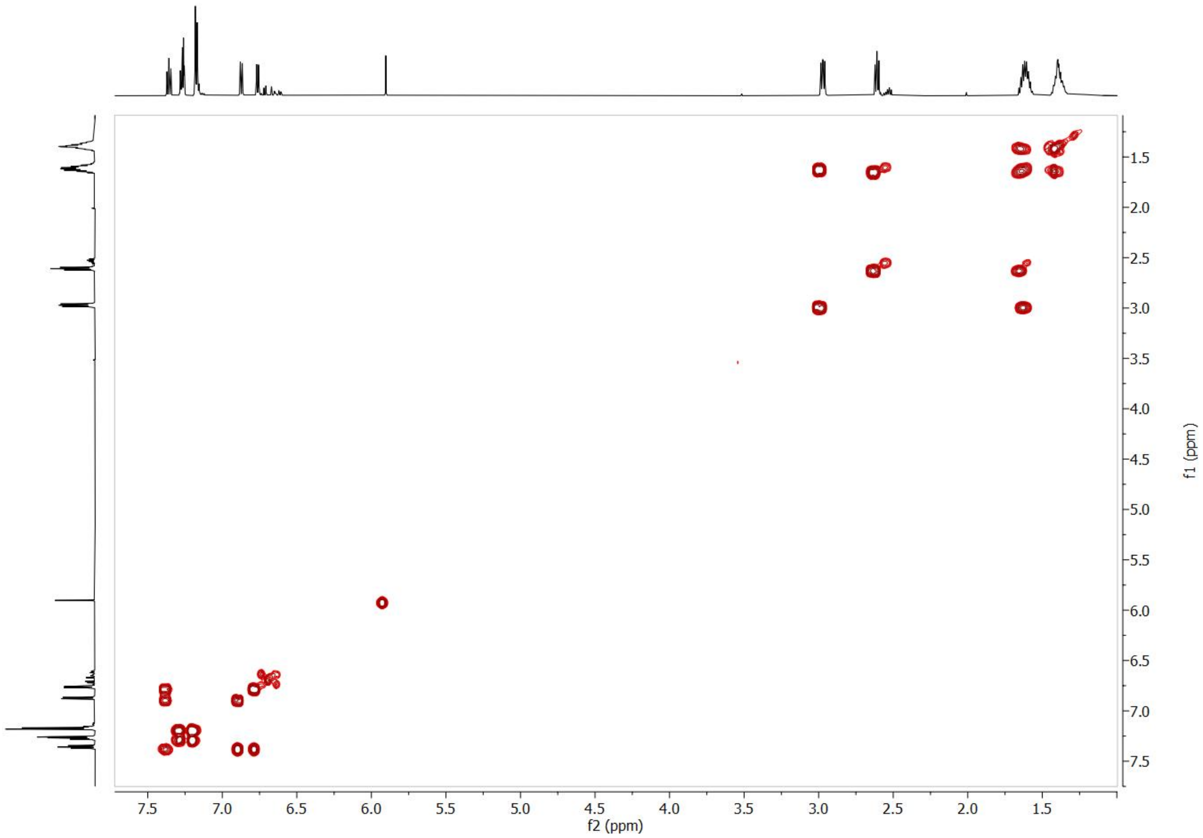

COSY NMR spectrum of **Knemolic acid C** in $CDCl_3$ at 600 MHz.

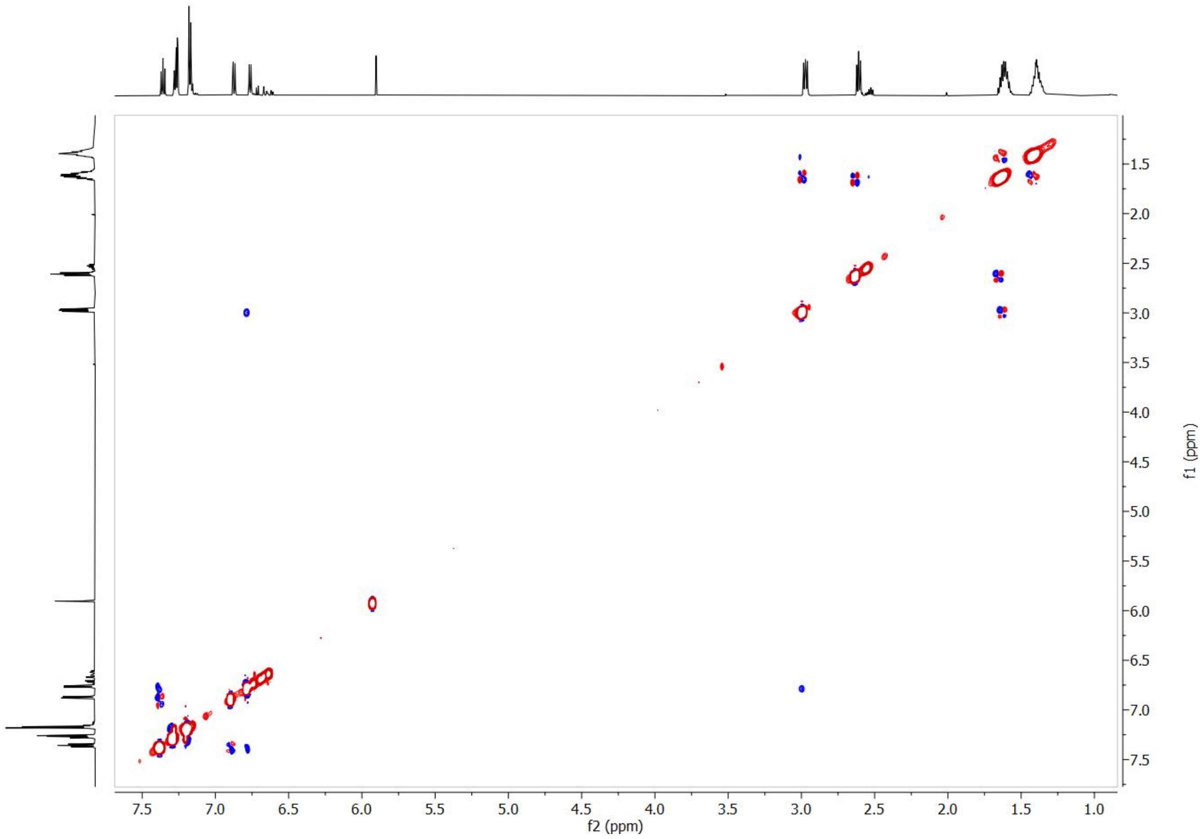

ROESY NMR spectrum of **Knemolic acid C** in $CDCl_3$ at 600 MHz.

8. Khookerianic acid A:

Experimental:

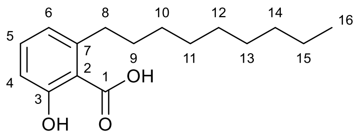

Khookerianic acid A (**8**) Green amorphous solid; UV (MeOH) λ_{max} (log ε) 211 (4.51), 310 (3.62) nm; ^{1}H NMR ($CDCl_3$, 600 MHz) δ 7.36 (1H, t, J = 7.9 Hz), 6.87 (1H, dd, J = 8.3, 1.1 Hz), 6.80 – 6.73 (1H, m), 3.00 – 2.95 (2H, m), 1.64 – 1.55 (2H, m), 1.41 – 1.33 (2H, m), 1.34 – 1.22 (10H, m), 0.87 (3H, t, J = 7.0 Hz); ^{13}C NMR ($CDCl_3$, 151 MHz) δ 175.7, 163.8, 147.9, 135.5, 122.9, 116.0, 110.6, 36.6, 32.2, 32.0, 30.0, 29.7, 29.6, 29.5, 22.8, 14.3 (NP-MRD ID: NP0333022); HRESIMS *m/z* 263.1651 [M-H]$^-$ (calcd for $C_{16}H_{24}O_3^-$ 263.1653, Δ = -0.76 ppm), *m/z* 265.1794 [M+H]$^+$ (calcd for $C_{16}H_{25}O_3^+$ 265.1798, Δ = -1.51 ppm), MS/MS spectrum: CCMSLIB00012475061.

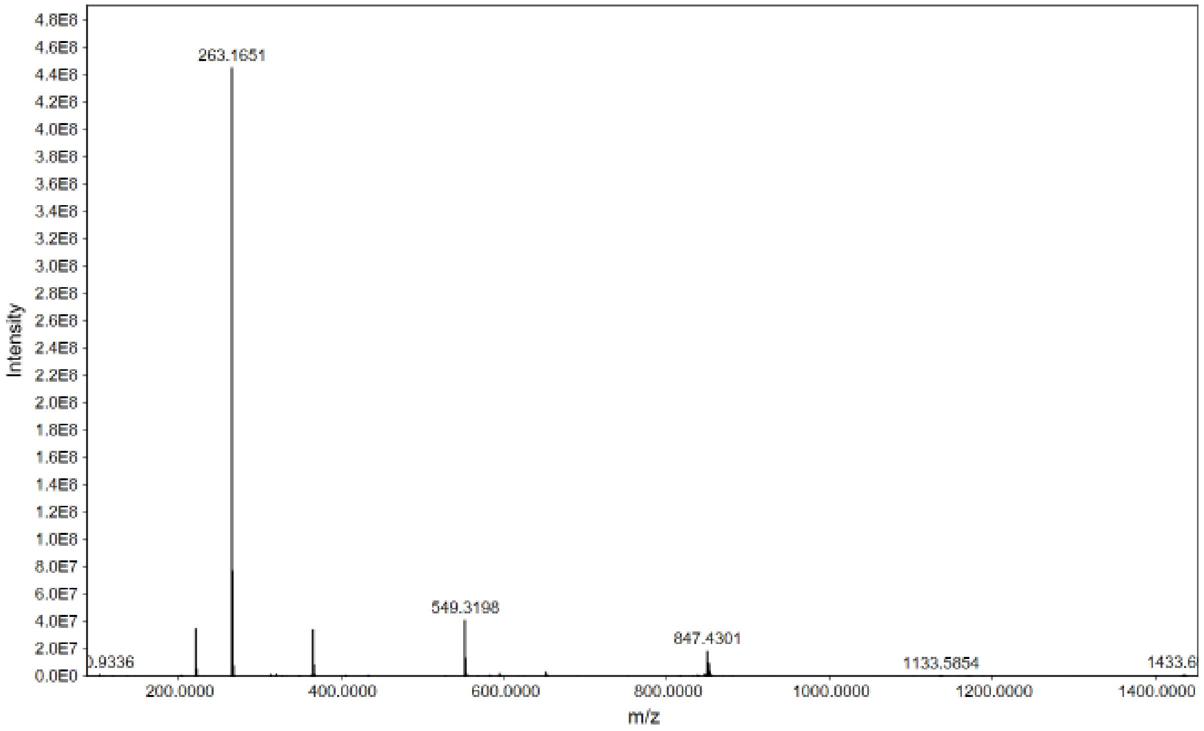

HRESIMS- spectrum of **Khookerianic acid A** in MeOH.

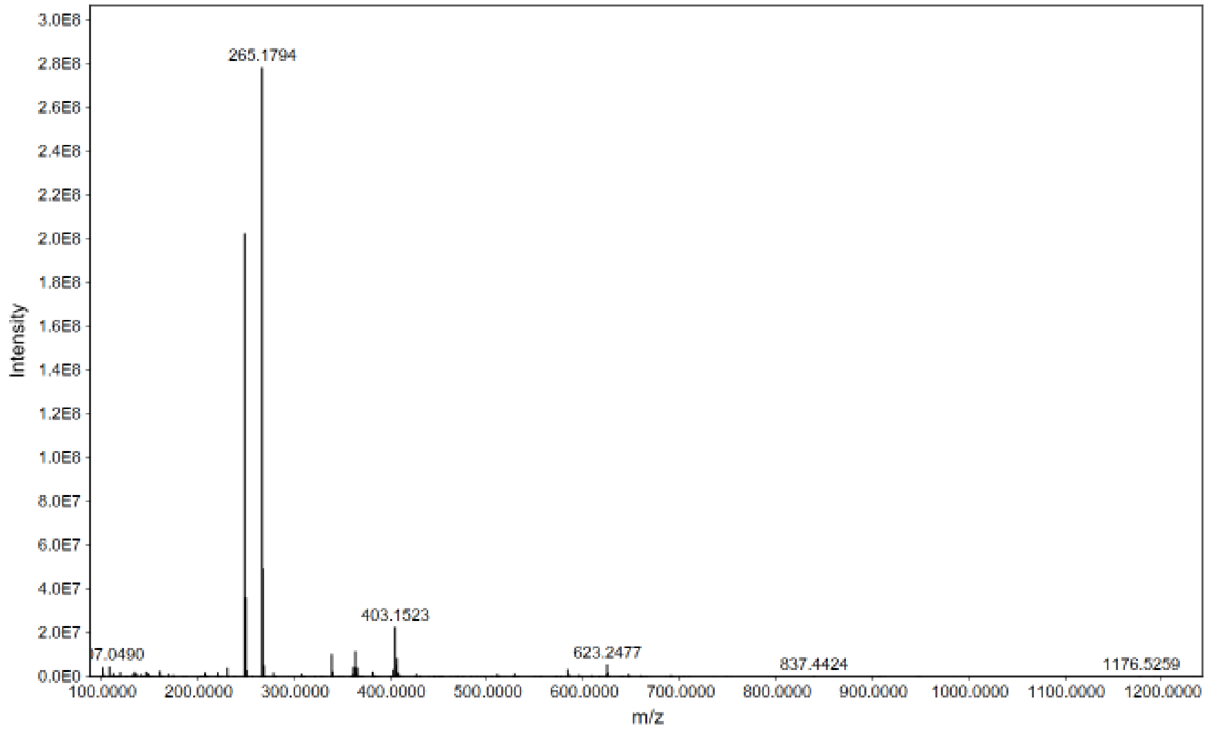

HRESIMS+ spectrum of **Khookerianic acid A** in MeOH.

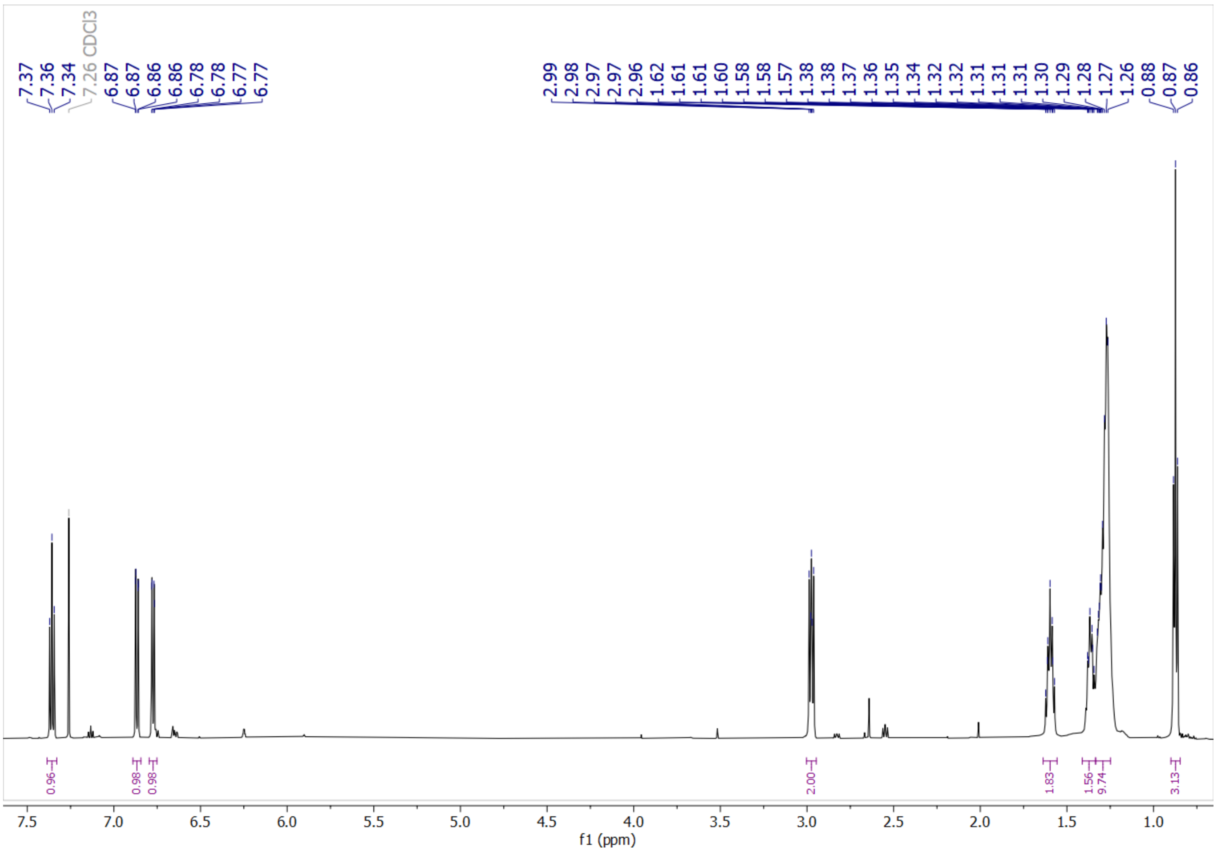

^{1}H NMR spectrum of **Khookerianic acid A** in $CDCl_3$ at 600 MHz.

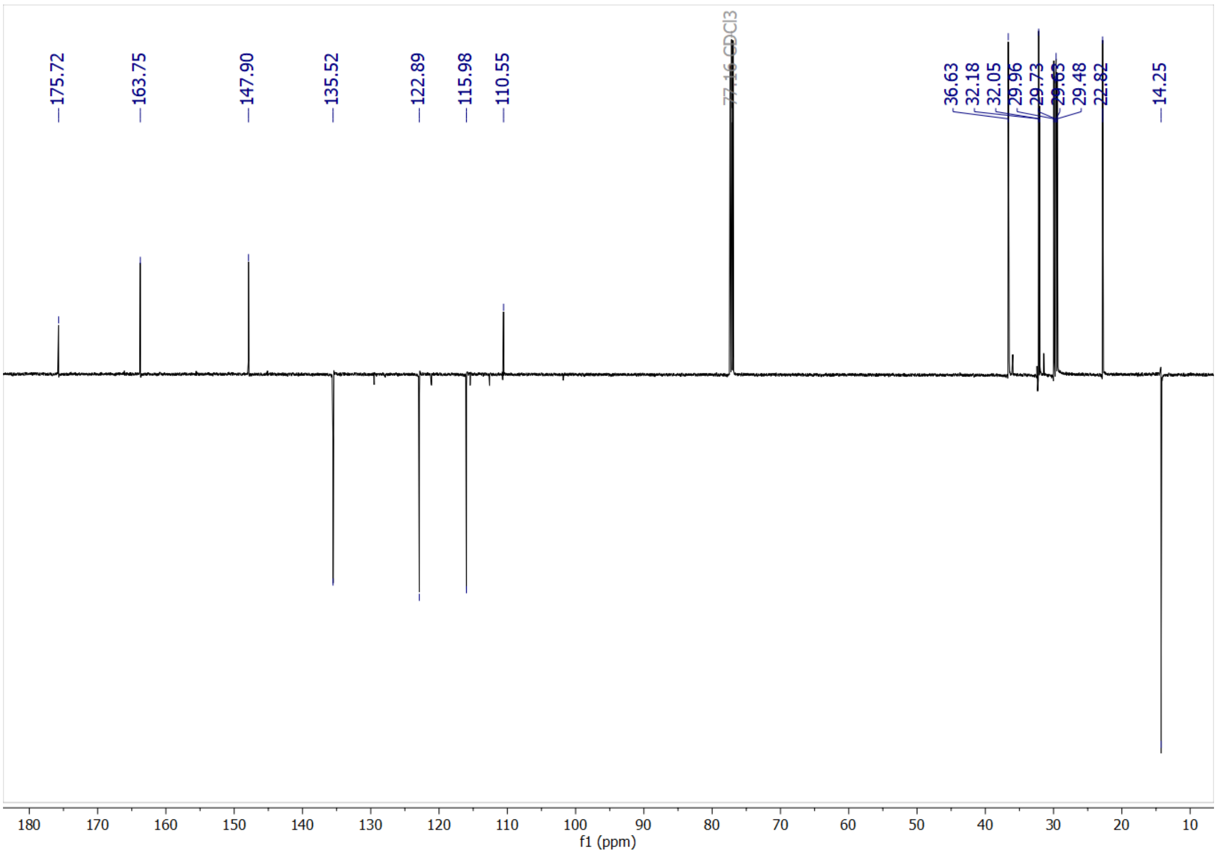

^{13}C DEPTQ NMR spectrum of **Khookerianic acid A** in $CDCl_3$ at 151 MHz.

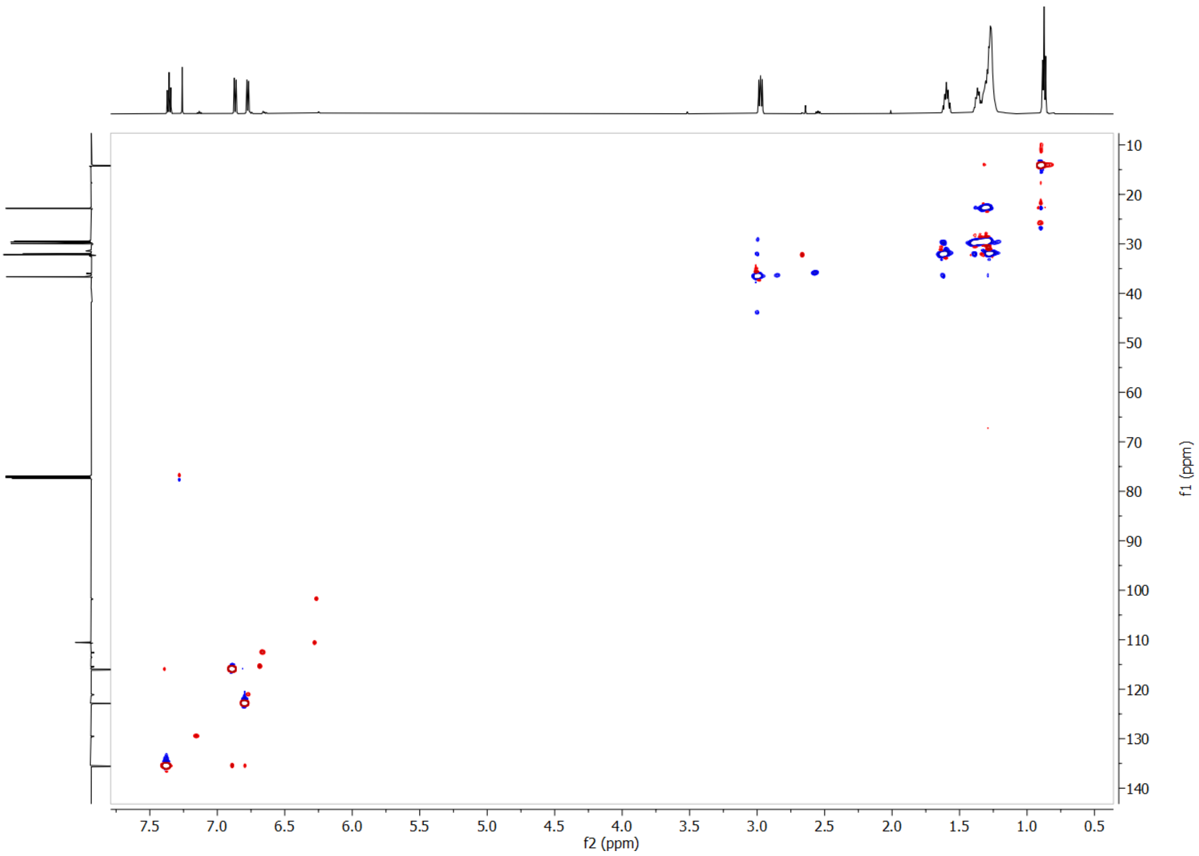

HSQC NMR spectrum of **Khookerianic acid A** in $CDCl_3$ at 600 MHz.

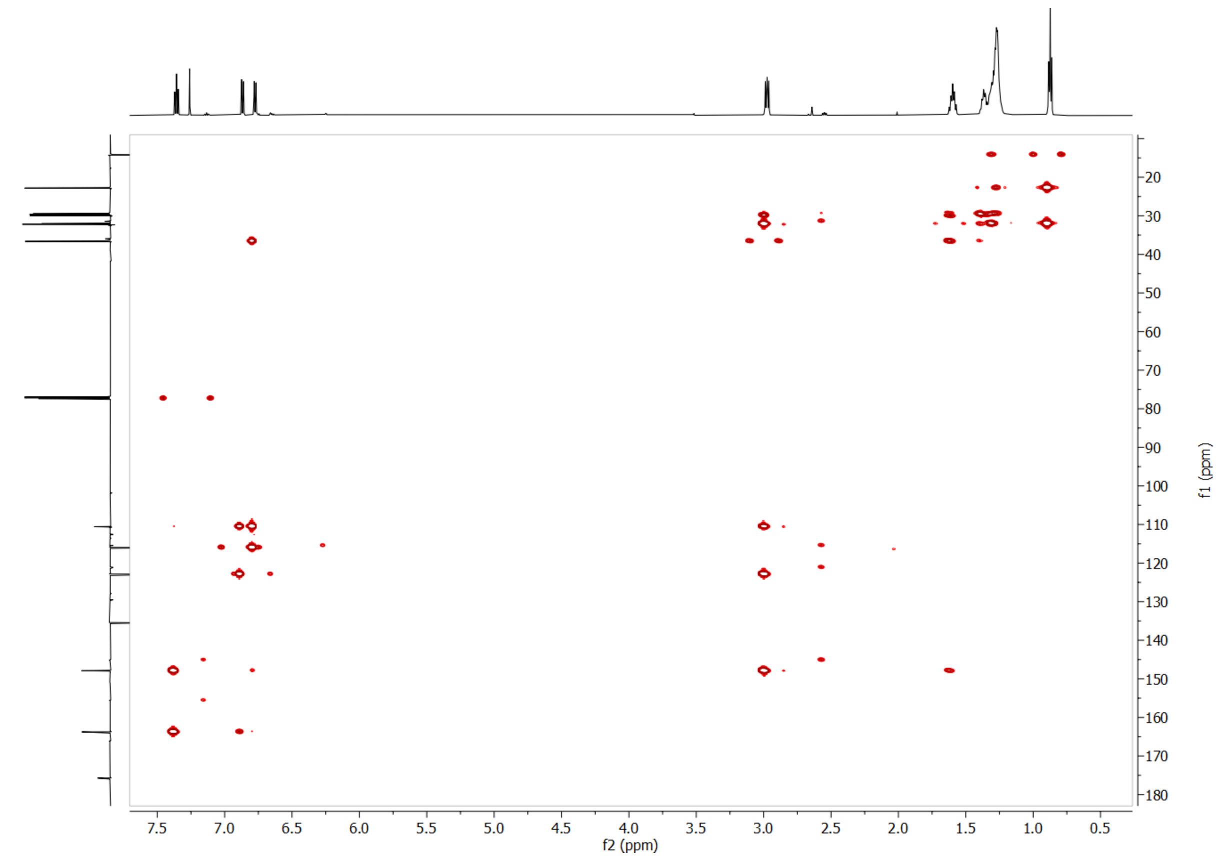

HMBC NMR spectrum of **Khookerianic acid A** in $CDCl_3$ at 600 MHz.

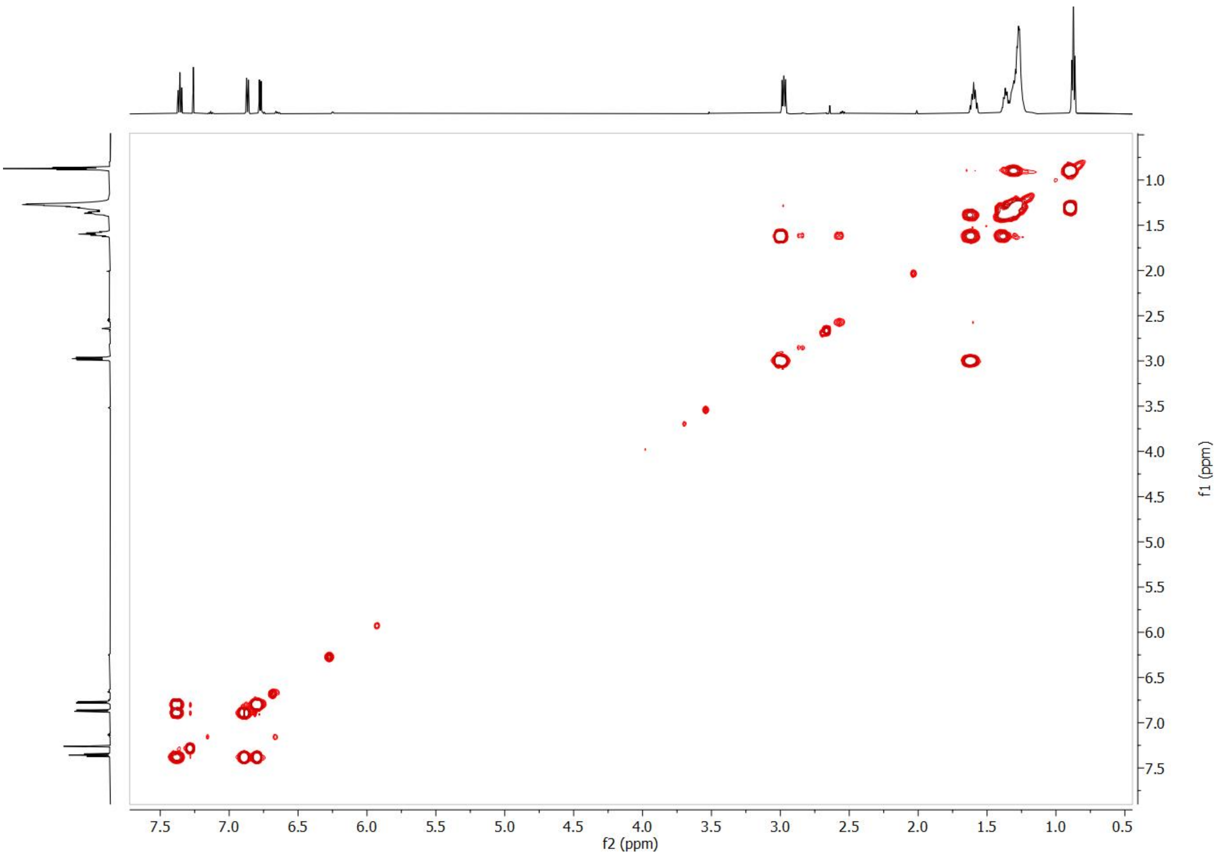

COSY NMR spectrum of **Khookerianic acid A** in $CDCl_3$ at 600 MHz.

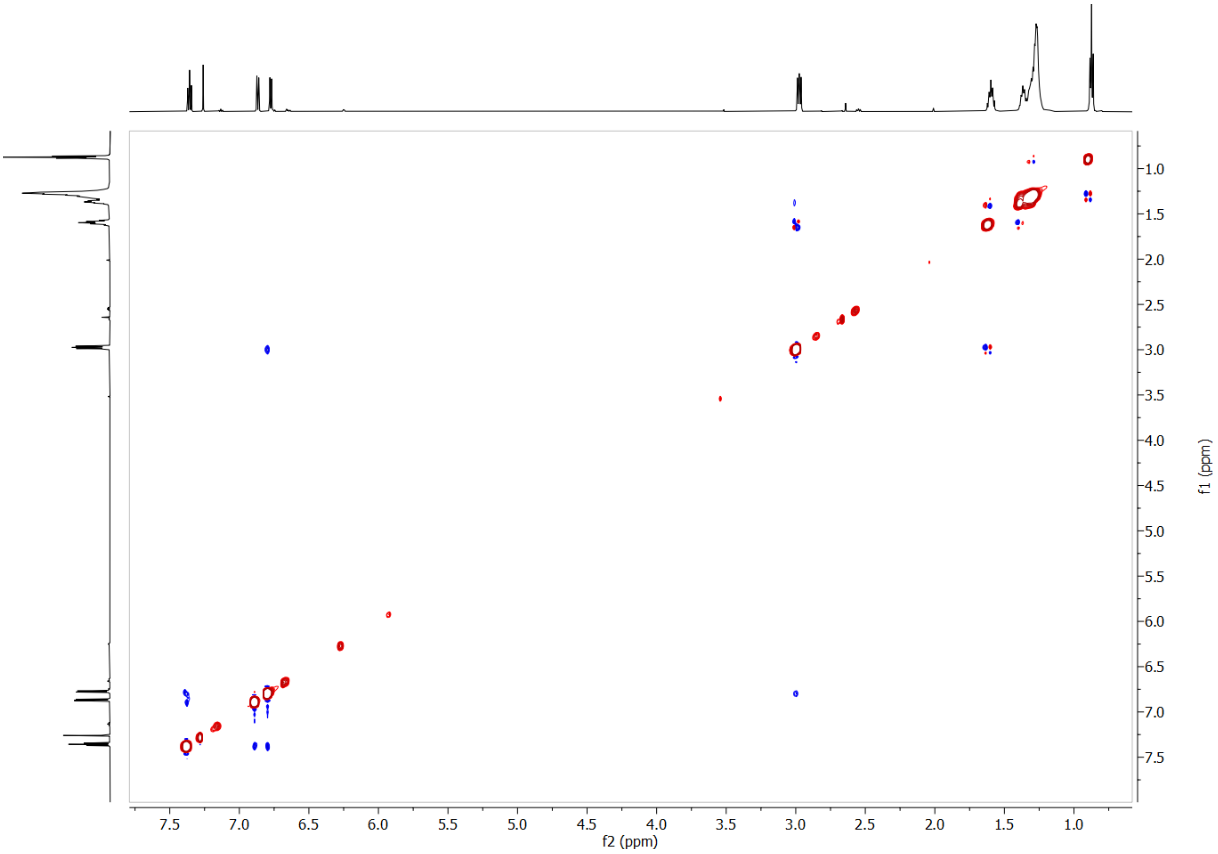

ROESY NMR spectrum of **Khookerianic acid A** in $CDCl_3$ at 600 MHz.

9. Kneglobularic acid B:

Experimental:

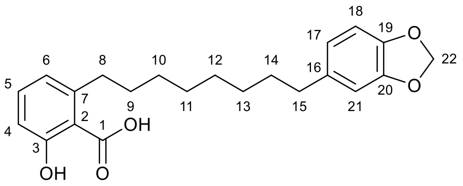

Kneglobularic acid B (**9**) Green amorphous solid; UV (MeOH) λ_{max} (log ε) 224 (3.93), 288 (3.66) nm; ^{1}H NMR ($CDCl_3$, 600 MHz) δ 7.34 (1H, t, J = 7.9 Hz), 6.85 (1H, dd, J = 8.3, 1.2 Hz), 6.75 (1H, dd, J = 7.5, 1.1 Hz), 6.71 (1H, d, J = 7.8 Hz), 6.67 (1H, d, J = 1.7 Hz), 6.61 (1H, dd, J = 7.8, 1.7 Hz), 5.91 (2H, s), 2.94 (2H, t, J = 7.9 Hz), 2.51 (2H, t, J = 7.8 Hz), 1.60 – 1.53 (4H, m), 1.32 – 1.28 (8H, m); ^{13}C NMR ($CDCl_3$, 151 MHz) δ 174.2, 163.7, 147.5, 145.5, 137.0, 135.3, 122.7, 121.2, 115.9, 110.5, 109.0, 108.2, 100.8, 36.6, 35.8, 32.2, 31.8, 29.9, 29.5, 29.5, 29.2 (NP-MRD ID: NP0333023); HRESIMS *m/z* 369.1705 $[M-H]^-$ (calcd for $C_{22}H_{25}O_5^-$ 369.1707, Δ = -0.54 ppm), *m/z* 353.1744 $[M-H_2O+H]^+$ (calcd for $C_{22}H_{25}O_4^+$ 353.1747, Δ = -0.85 ppm), MS/MS spectrum: CCMSLIB00012475062.

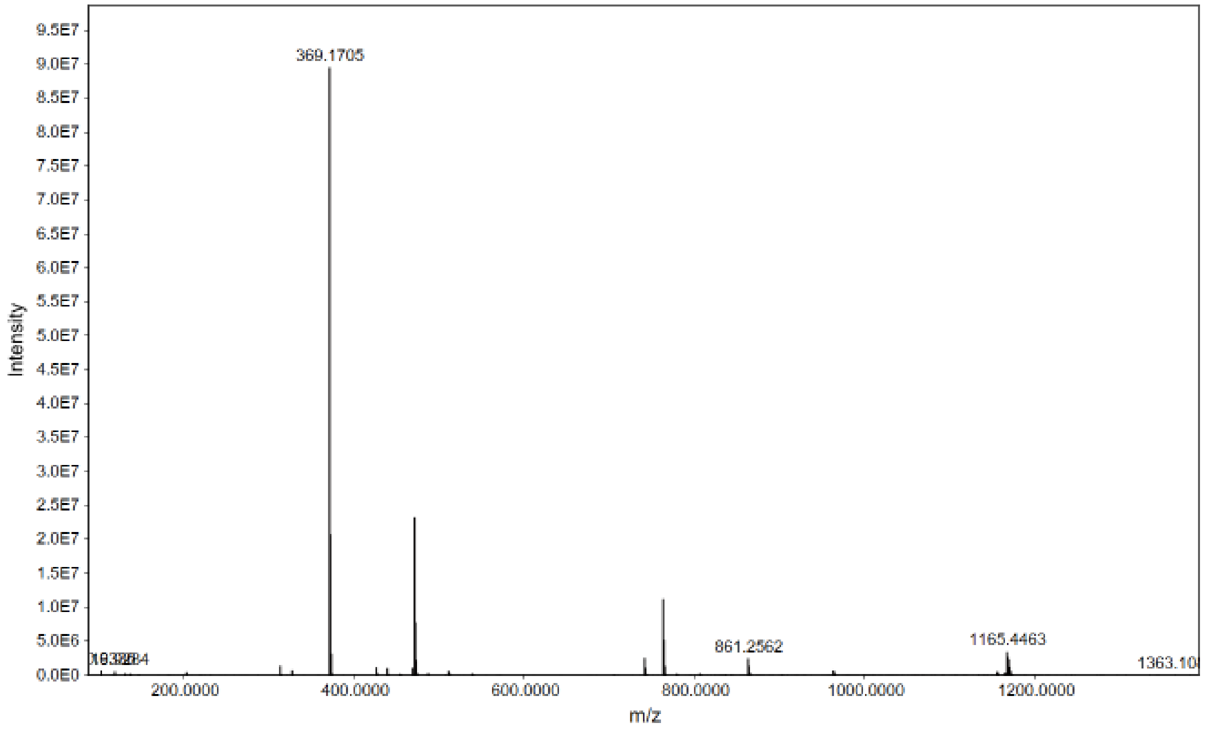

HRESIMS- spectrum of **Kneglobularic acid B** in MeOH.

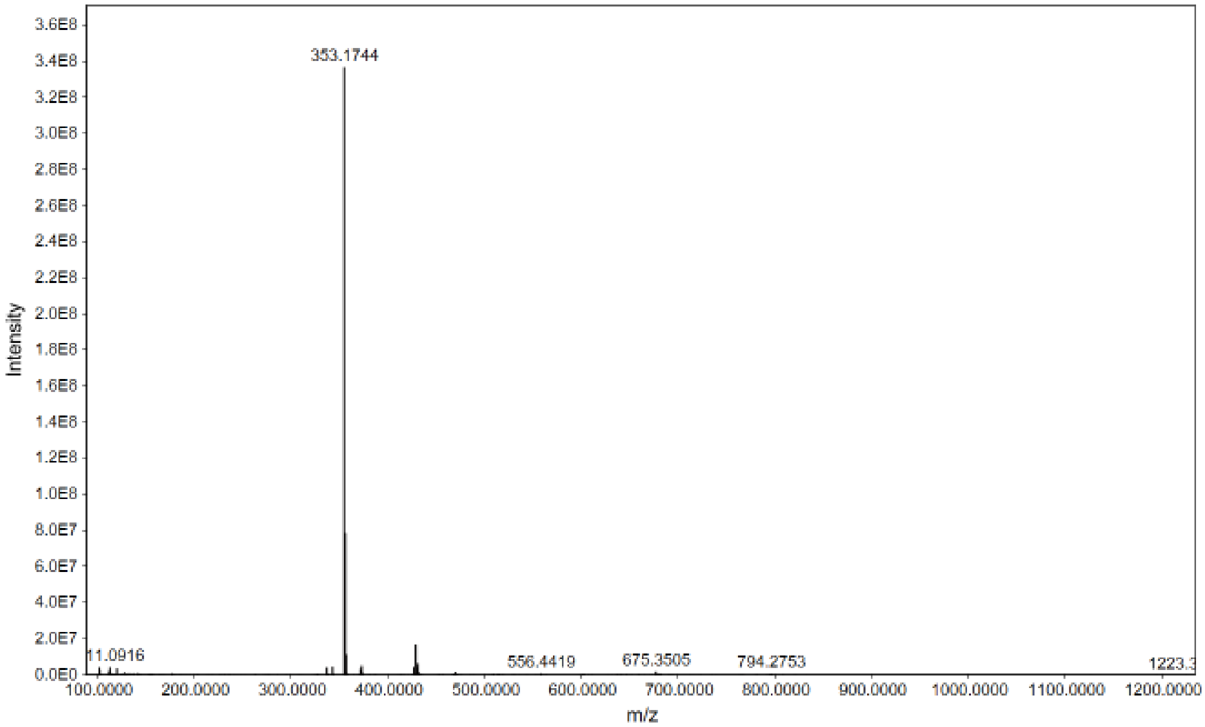

HRESIMS+ spectrum of **Kneglobularic acid B** in MeOH.

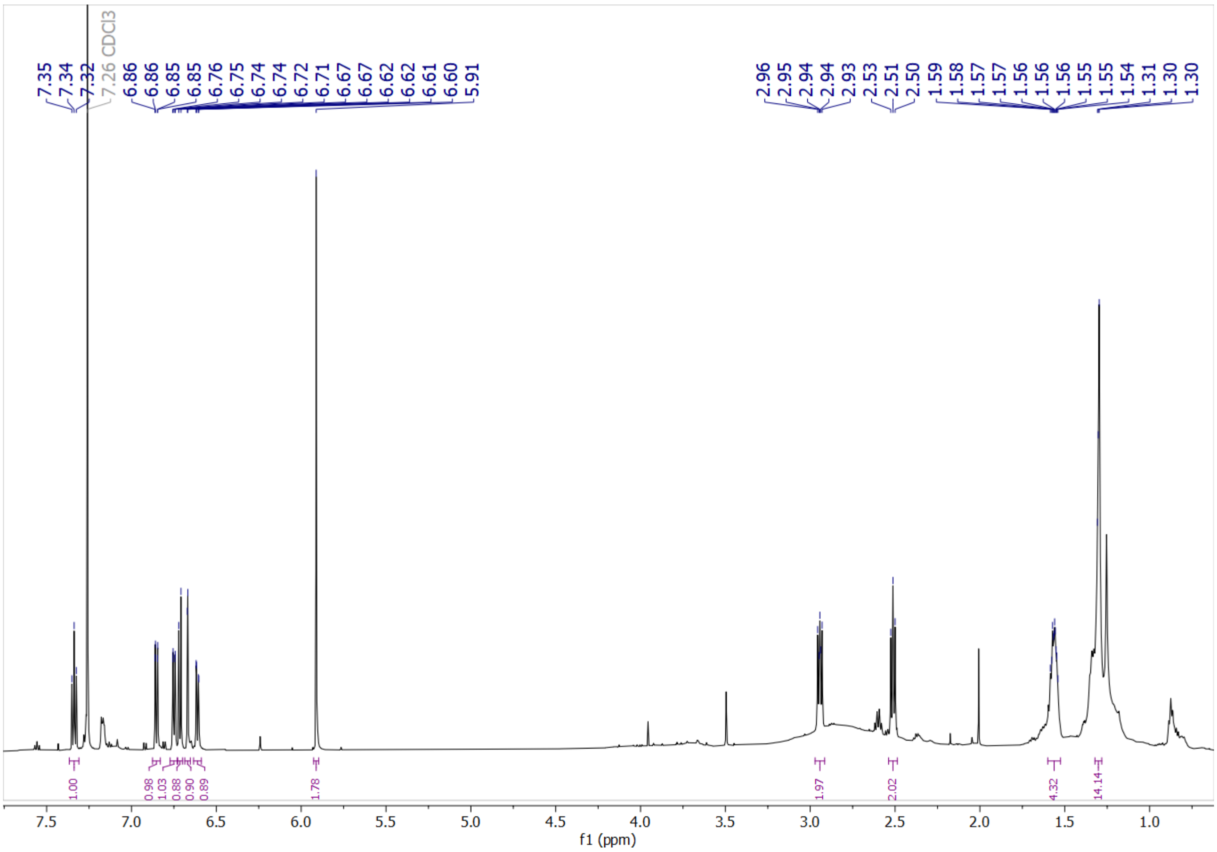

^{1}H NMR spectrum of **Kneglobularic acid B** in $CDCl_3$ at 600 MHz.

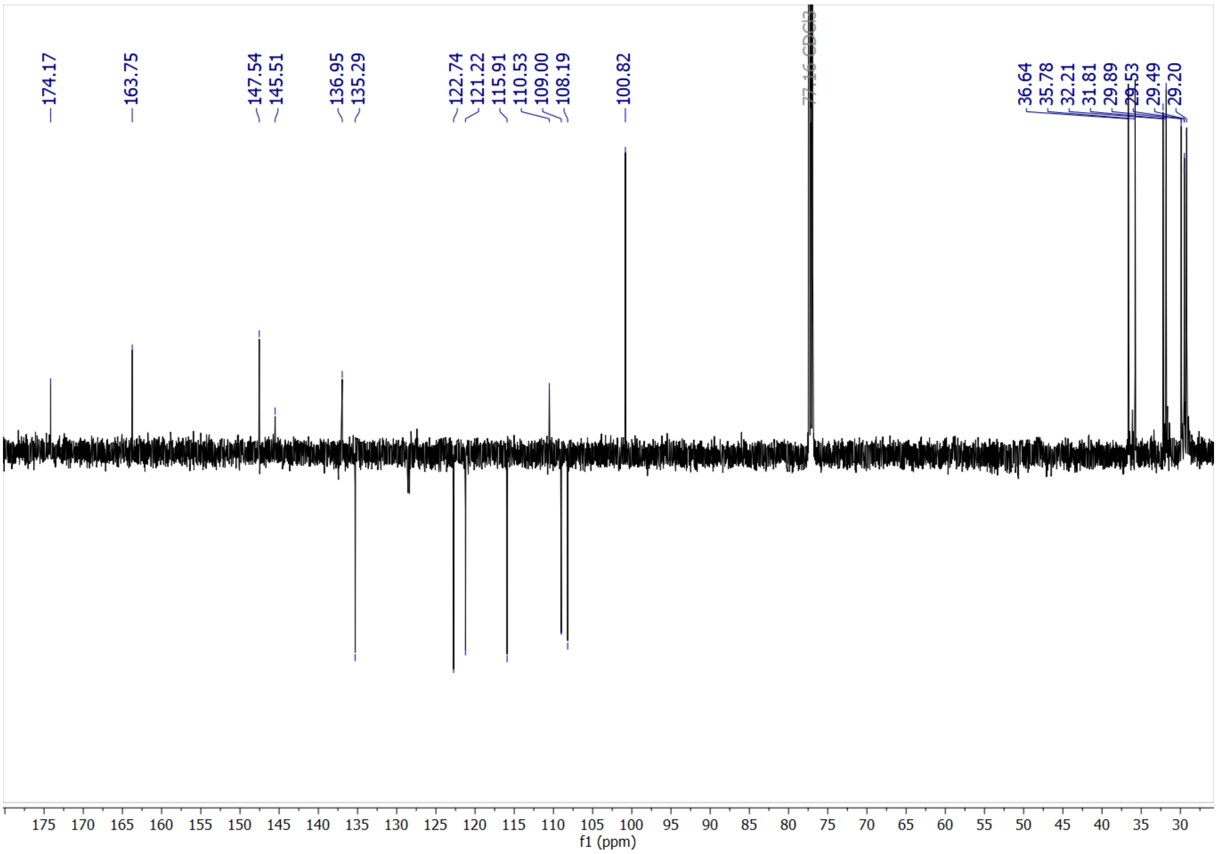

^{13}C DEPTQ NMR spectrum of **Kneglobularic acid B** in $CDCl_3$ at 151 MHz.

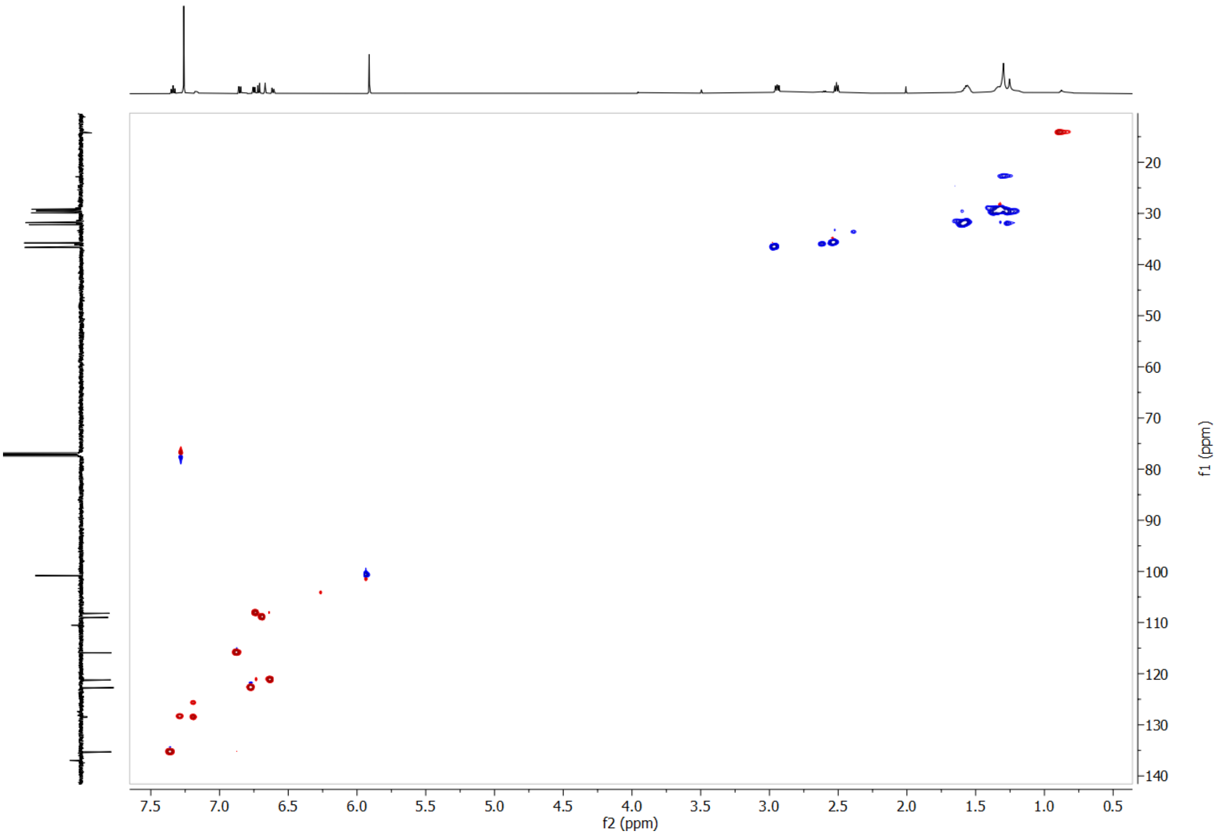

HSQC NMR spectrum of **Kneglobularic acid B** in $CDCl_3$ at 600 MHz.

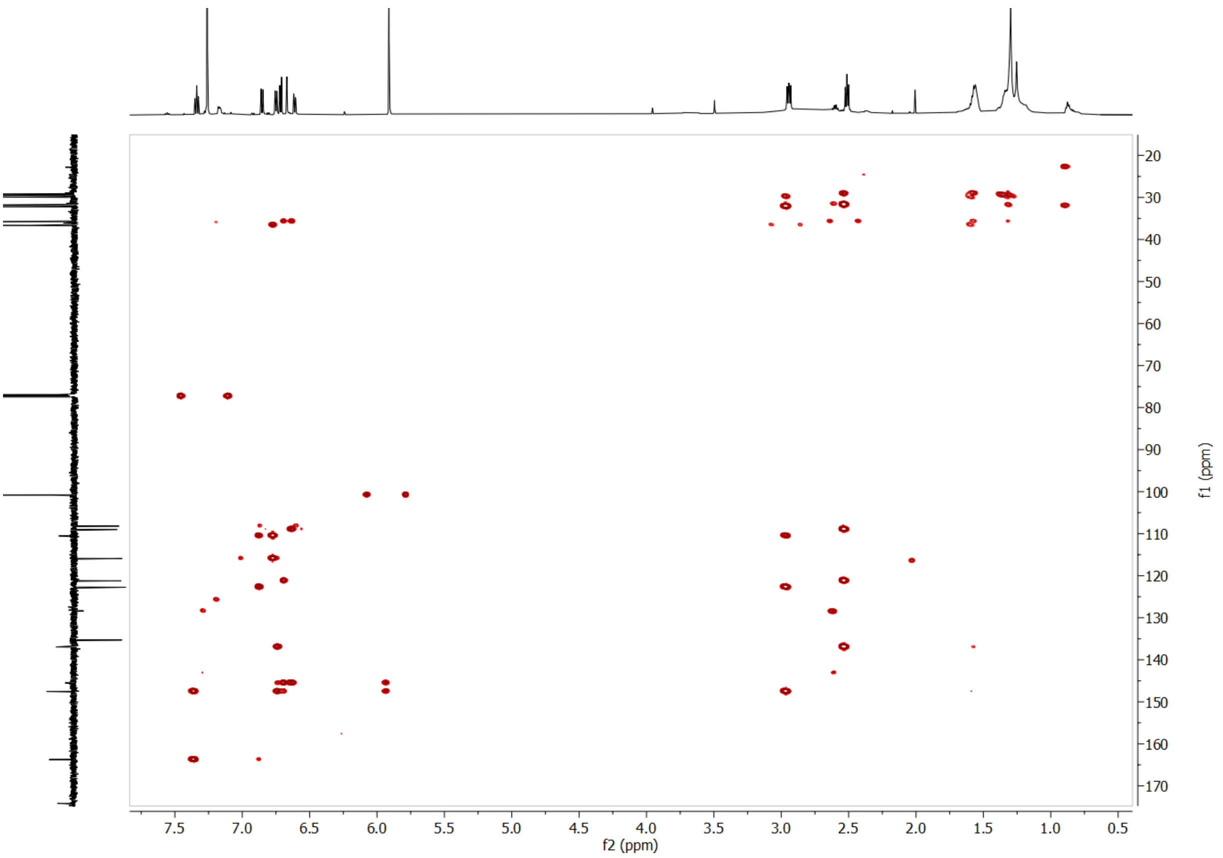

HMBC NMR spectrum of **Kneglobularic acid B** in $CDCl_3$ at 600 MHz.

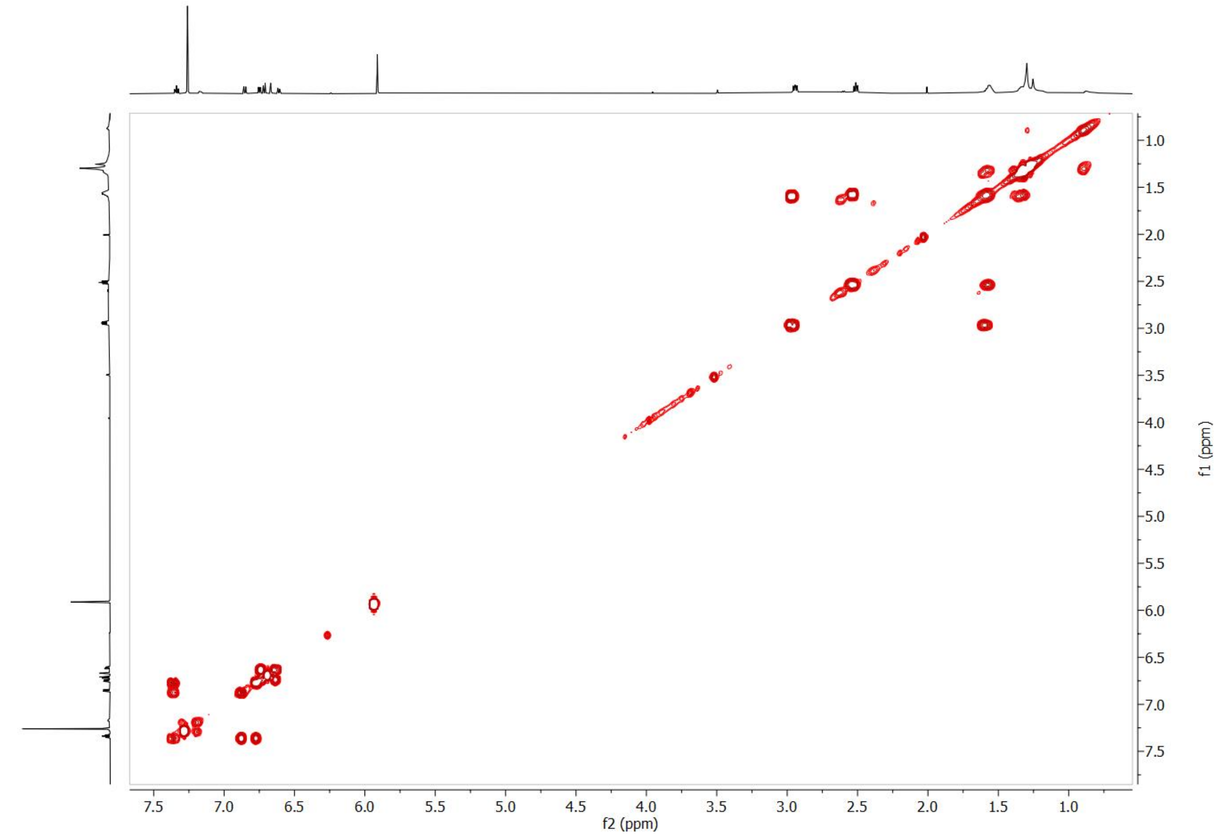

COSY NMR spectrum of **Kneglobularic acid B** in $CDCl_3$ at 600 MHz.

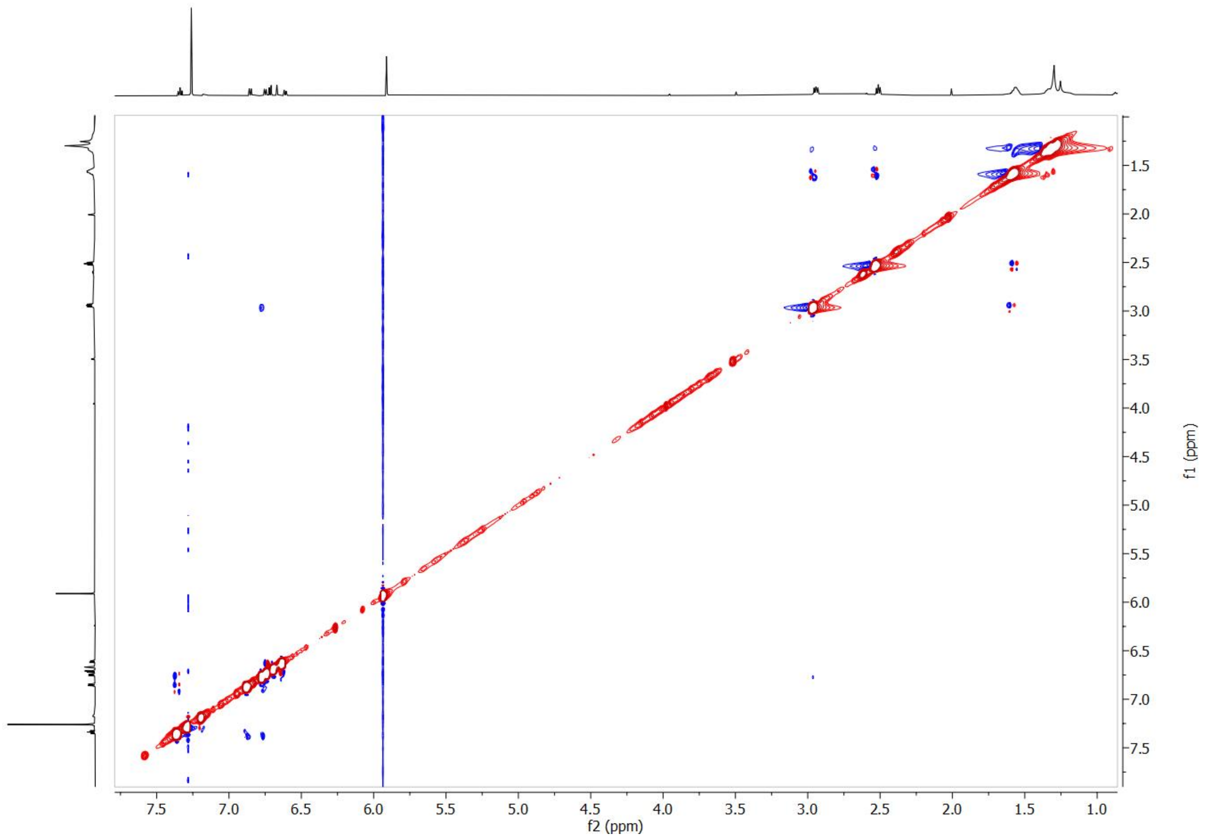

ROESY NMR spectrum of **Kneglobularic acid B** in $CDCl_3$ at 600 MHz.

10. Khookerianic acid C/Kneglobularic acid A:

Experimental:

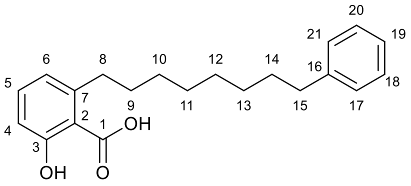

Khookerianic acid C/Kneglobularic acid A (**10**) Green amorphous solid; UV (MeOH) λ_{max} (log ε) 191 (4.58), 212 (4.58), 310 (3.55) nm; ^{1}H NMR ($CDCl_3$, 600 MHz) δ 7.35 (1H, t, J = 7.9 Hz), 7.28 – 7.24 (2H, m), 7.19 – 7.15 (3H, m), 6.86 (1H, dd, J = 8.4, 1.2 Hz), 6.76 (1H, dd, J = 7.5, 1.2 Hz), 2.96 (2H, t, J = 7.9 Hz), 2.60 (2H, t, J = 7.8 Hz), 1.62 – 1.57 (4H, m), 1.36 – 1.29 (8H, m); ^{13}C NMR ($CDCl_3$, 151 MHz) δ 175.1, 163.8, 147.7, 143.1, 135.5, 128.5, 128.4, 125.7, 122.8, 116.0, 110.5, 36.6, 36.1, 32.2, 31.6, 29.9, 29.6, 29.5, 29.4 (NP-MRD ID: NP0333024); HRESIMS *m/z* 325.1808 $[M-H]^-$ (calcd for $C_{21}H_{25}O_3^-$ 325.1809, Δ = -0.31 ppm), *m/z* 309.1845 $[M-H_2O+H]^+$ (calcd for $C_{21}H_{25}O_2^+$ 309.1849, Δ = -1.29 ppm), MS/MS spectrum: CCMSLIB00012475063.

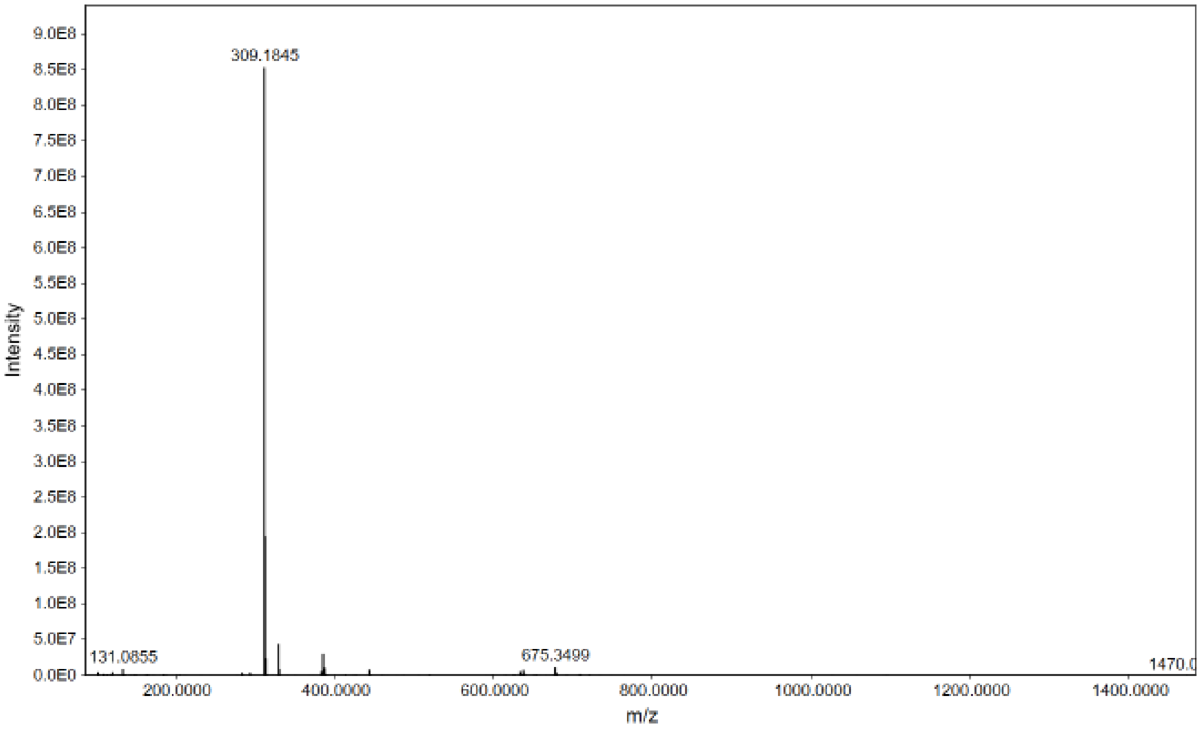

HRESIMS+ spectrum of **Khookerianic acid C/Kneglobularic acid A** in MeOH.

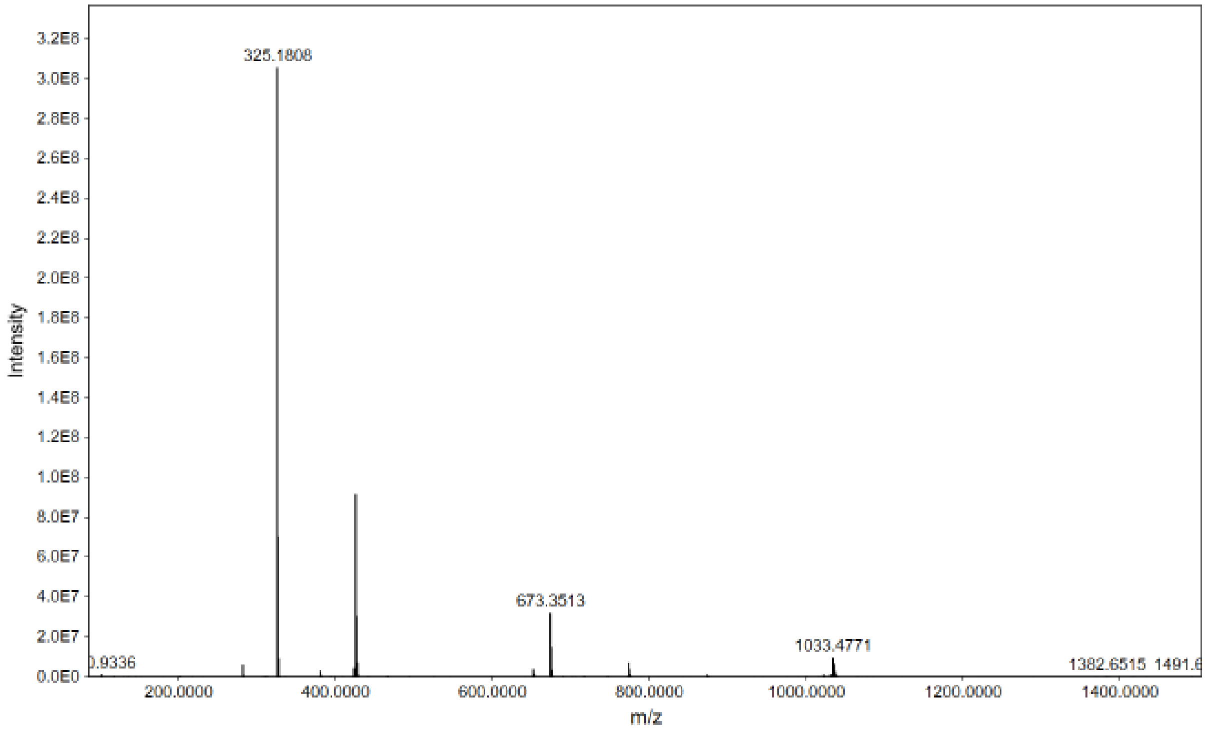

HRESIMS- spectrum of **Khookerianic acid C/Kneglobularic acid A** in MeOH.

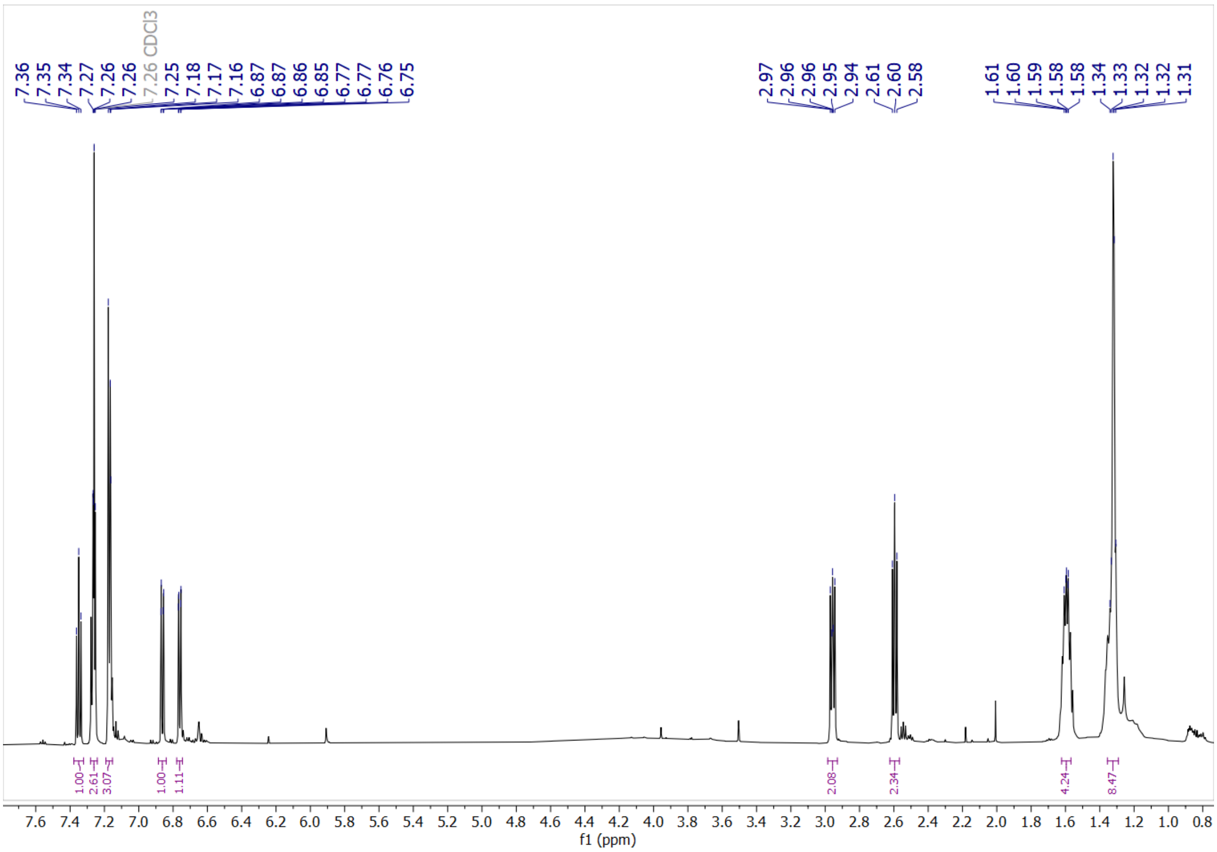

^{1}H NMR spectrum of **Khookerianic acid C/Kneglobularic acid A** in $CDCl_3$ at 600 MHz.

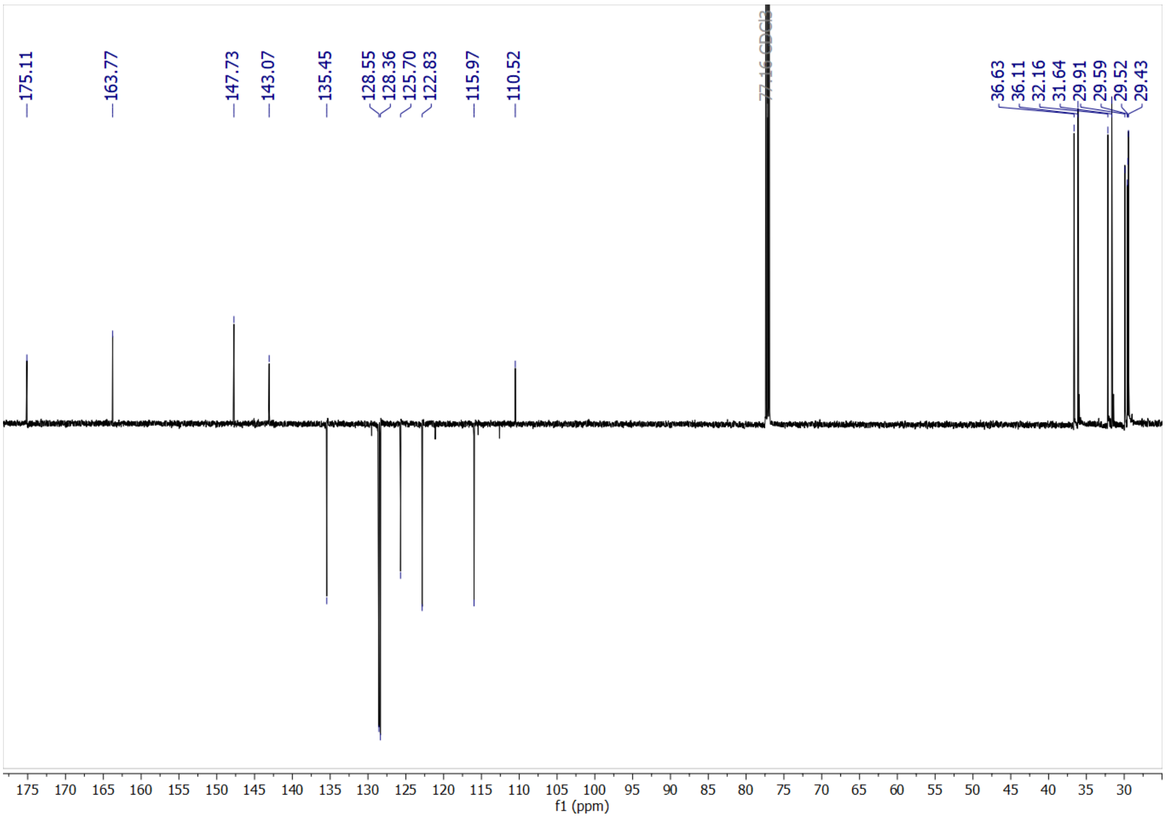

^{13}C DEPTQ NMR spectrum of **Khookerianic acid C/Kneglobularic acid A** in $CDCl_3$ at 151 MHz.

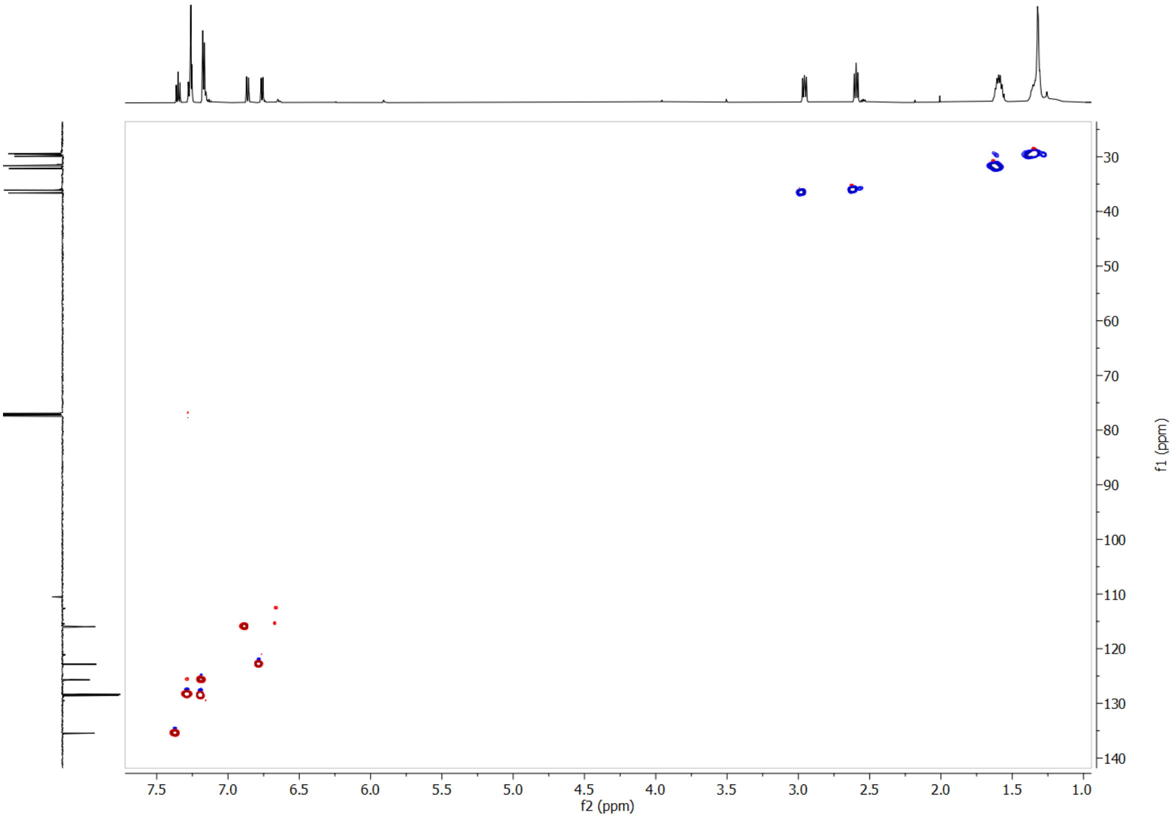

HSQC NMR spectrum of **Khookerianic acid C/Kneglobularic acid A** in $CDCl_3$ at 600 MHz.

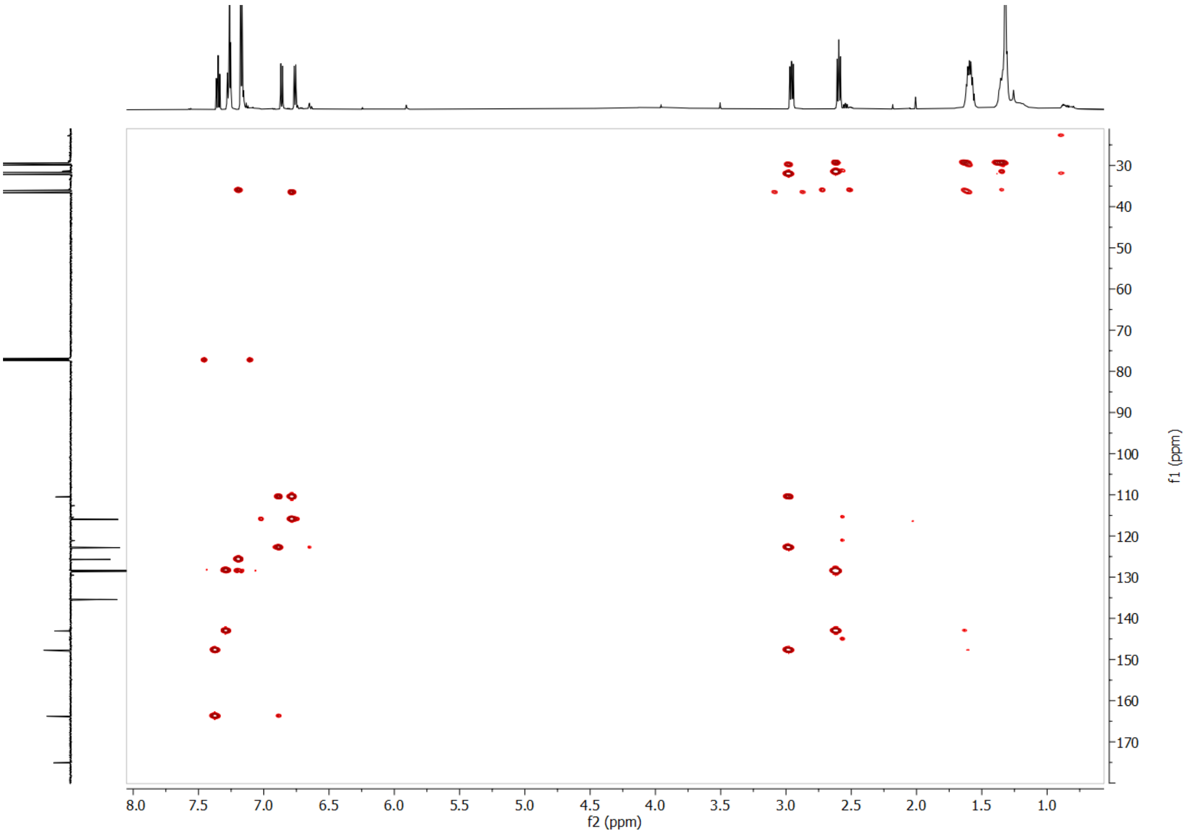

HMBC NMR spectrum of **Khookerianic acid C/Kneglobularic acid A** in $CDCl_3$ at 600 MHz.

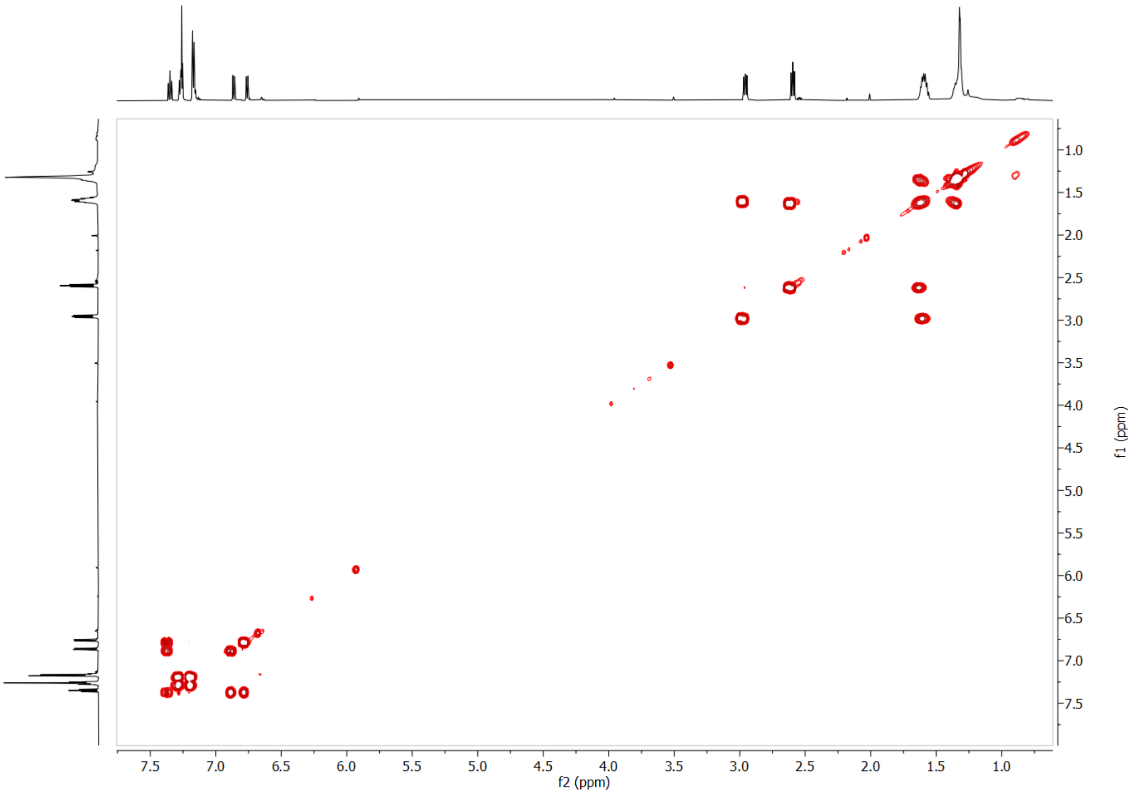

COSY NMR spectrum of **Khookerianic acid C/Kneglobularic acid A** in $CDCl_3$ at 600 MHz.

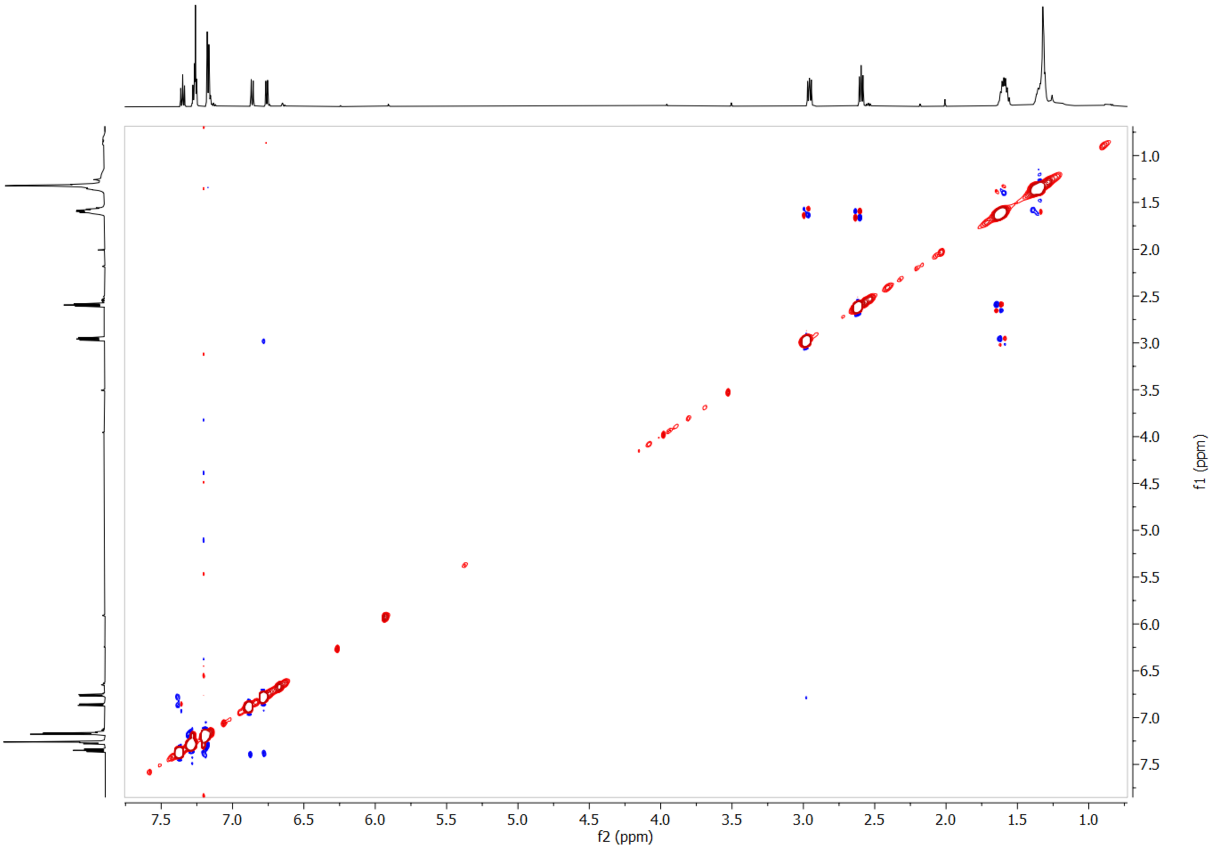

ROESY NMR spectrum of **Khookerianic acid C/Kneglobularic acid A** in $CDCl_3$ at 600 MHz.

11. Anagigantic acid:

Experimental:

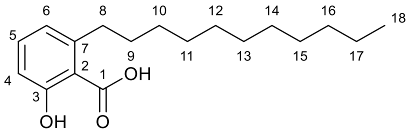

Anagigantic acid (**11**) Dark-green amorphous solid; UV (MeOH) λ_{max} (log ε) 213 (4.46), 311 (3.58) nm; ^{1}H NMR ($CDCl_3$, 600 MHz) δ 7.35 (1H, t, J = 7.9 Hz), 6.86 (1H, d, J = 8.3 Hz), 6.77 (1H, d, J = 7.4 Hz), 2.96 (2H, t, J = 7.8 Hz), 1.59 (2H, p, J = 7.6 Hz), 1.36 (2H, dd, J = 10.3, 5.2 Hz), 1.27 (14H, d, J = 13.0 Hz), 0.87 (3H, t, J = 7.0 Hz); ^{13}C NMR ($CDCl_3$, 151 MHz) δ 175.45, 163.76, 147.85, 135.48, 122.86, 115.98, 110.55, 36.64, 32.19, 32.07, 29.97, 29.83, 29.80, 29.78, 29.65, 29.50, 22.84, 14.27 (NP-MRD ID: NP0333025); HRESIMS *m/z* 291.1964 $[M-H]^-$ (calcd for $C_{18}H_{27}O_3^-$ 291.1966, Δ = -0.69 ppm), *m/z* 293.2108 $[M+H]^+$ (calcd for $C_{18}H_{29}O_3^+$ 293.2111, Δ = -1.02 ppm), MS/MS spectrum: CCMSLIB00012475070.

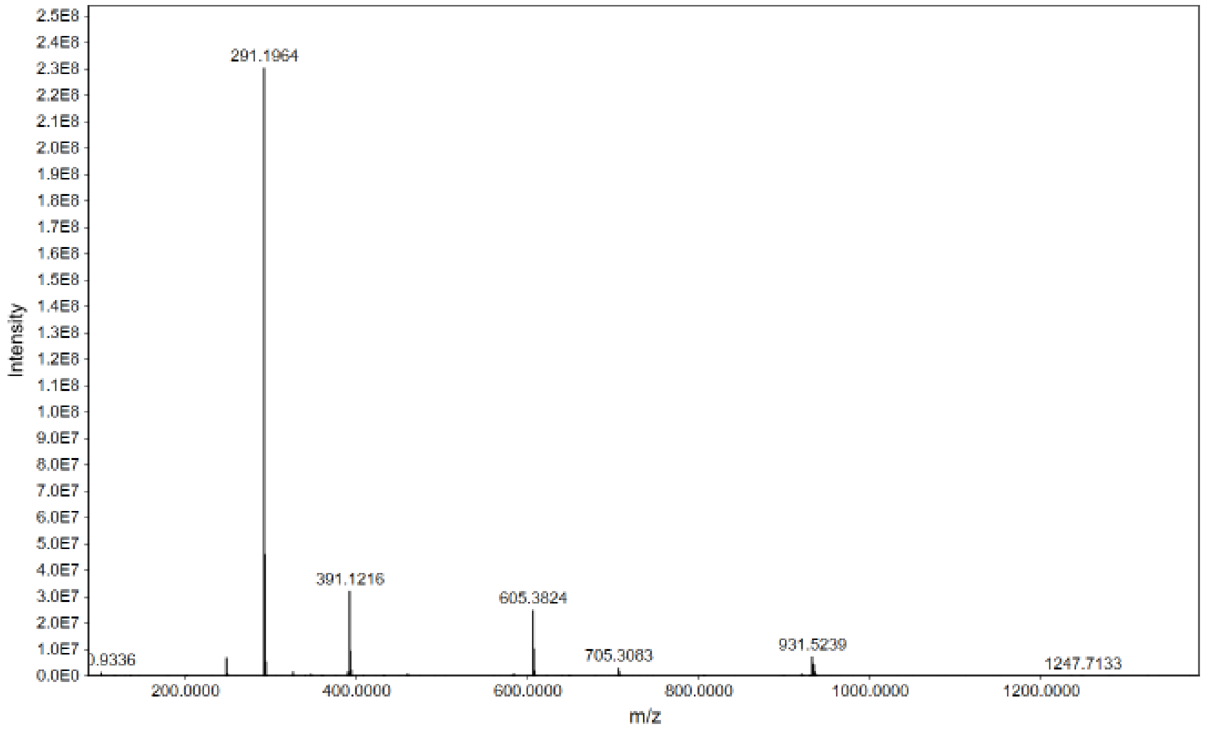

HRESIMS- spectrum of **Anagigantic acid** in MeOH.

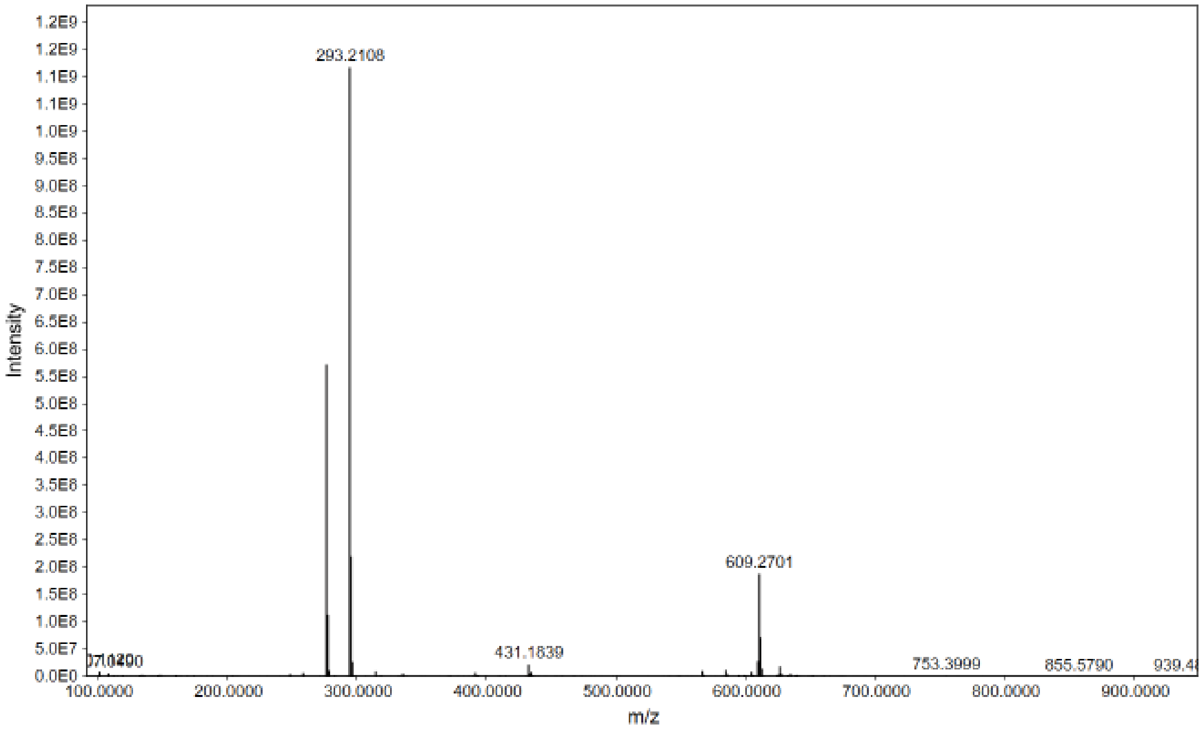

HRESIMS+ spectrum of **Anagigantic acid** in MeOH.

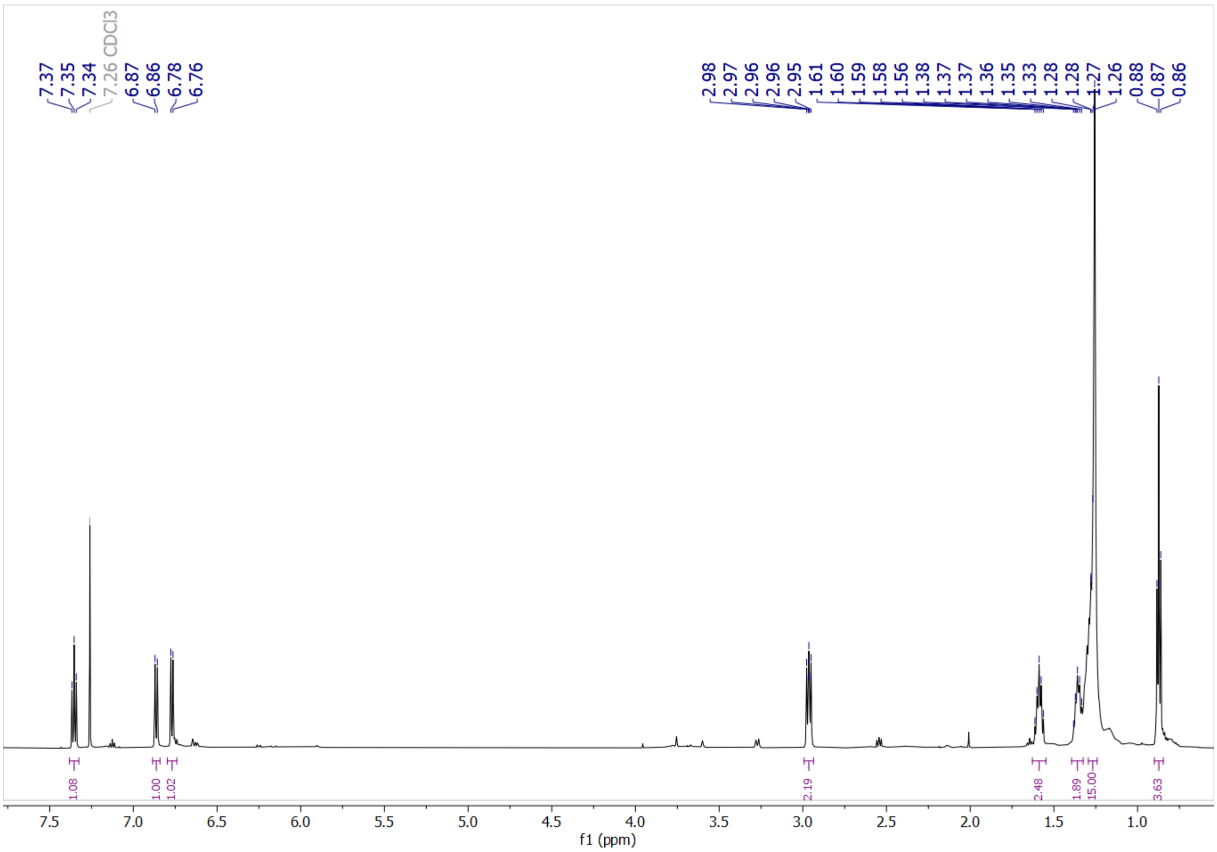

^{1}H NMR spectrum of **Anagigantic acid** in $CDCl_3$ at 600 MHz.

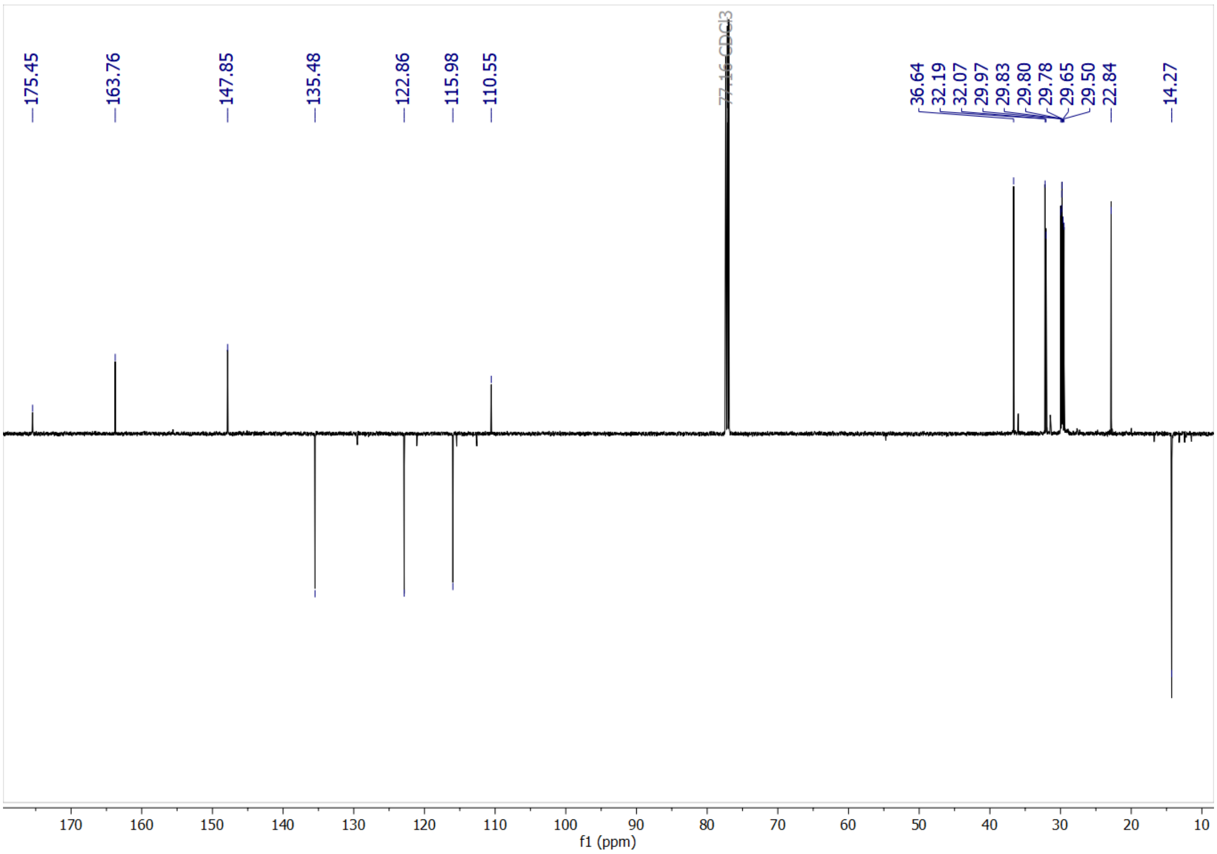

^{13}C DEPTQ NMR spectrum of **Anagigantic acid** in $CDCl_3$ at 151 MHz.

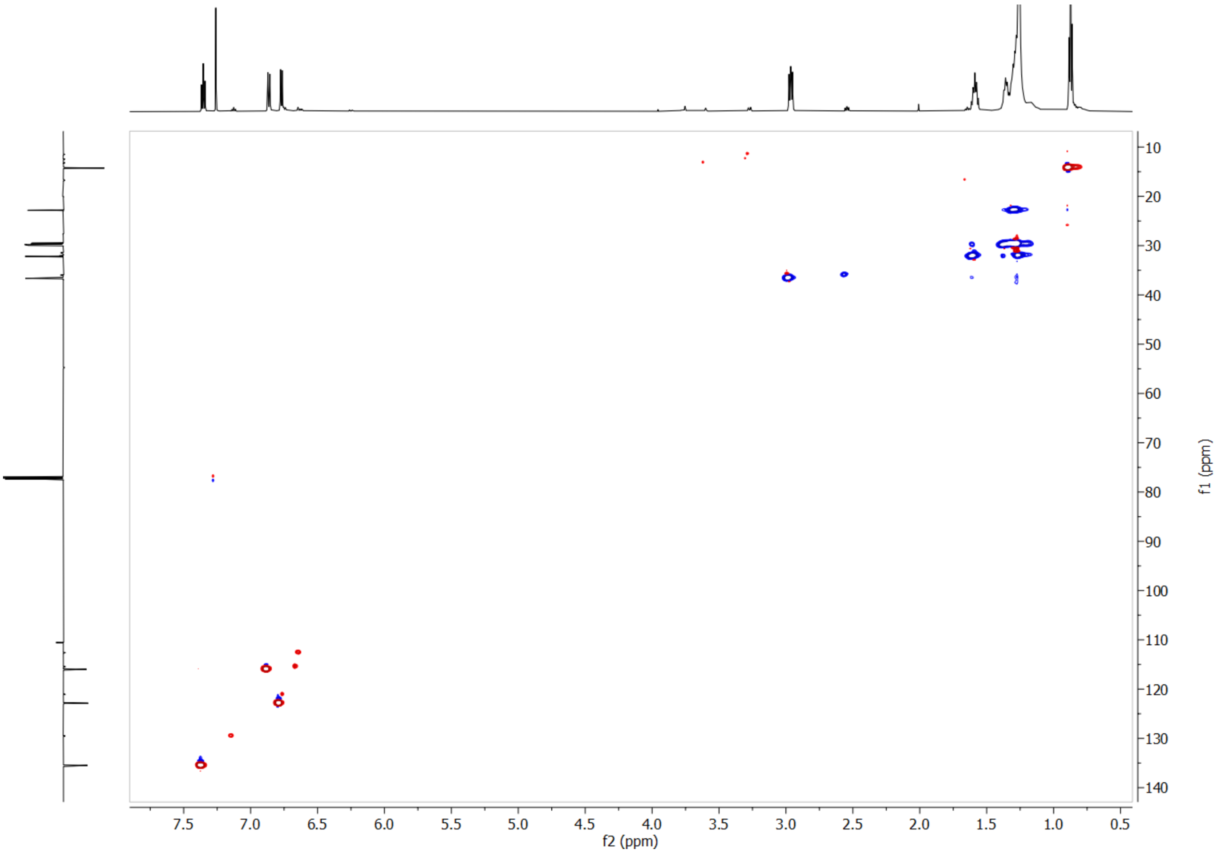

HSQC NMR spectrum of **Anagigantic acid** in $CDCl_3$ at 600 MHz.

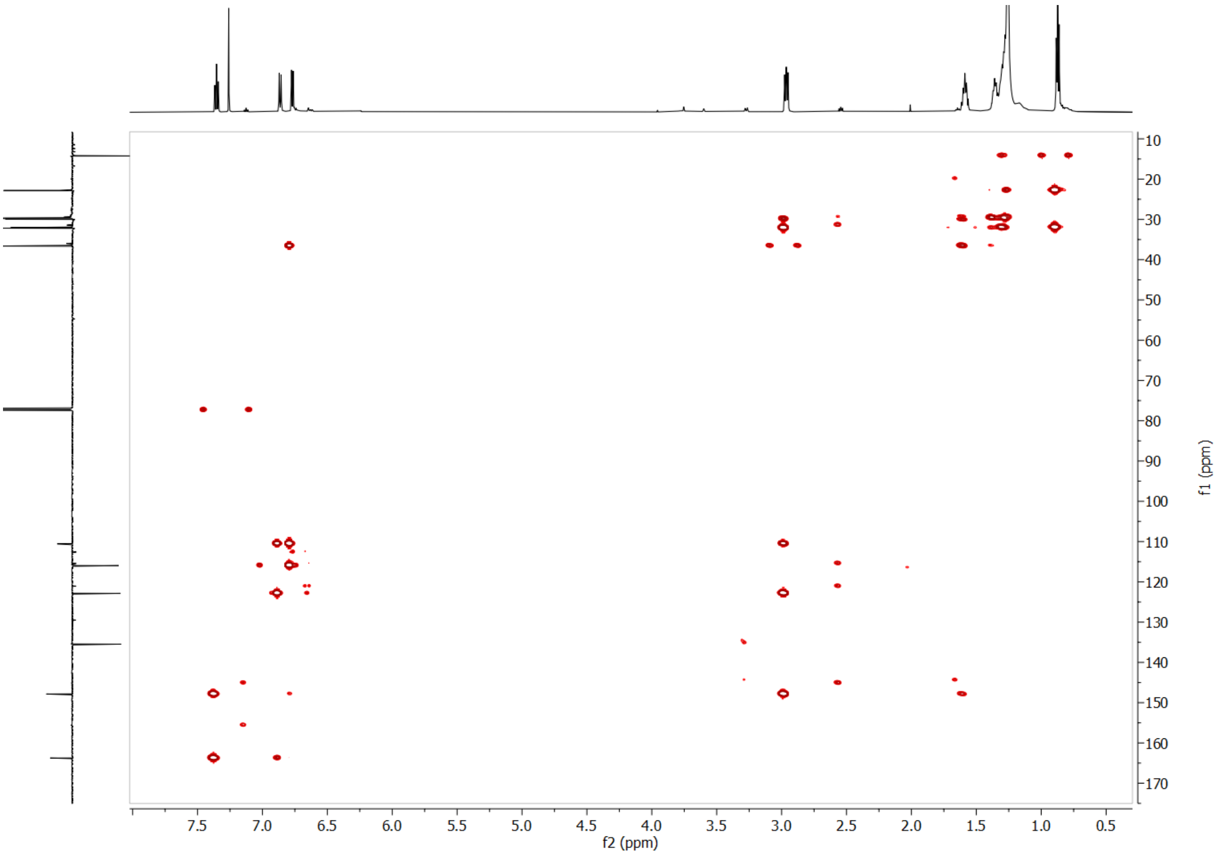

HMBC NMR spectrum of **Anagigantic acid** in $CDCl_3$ at 600 MHz.

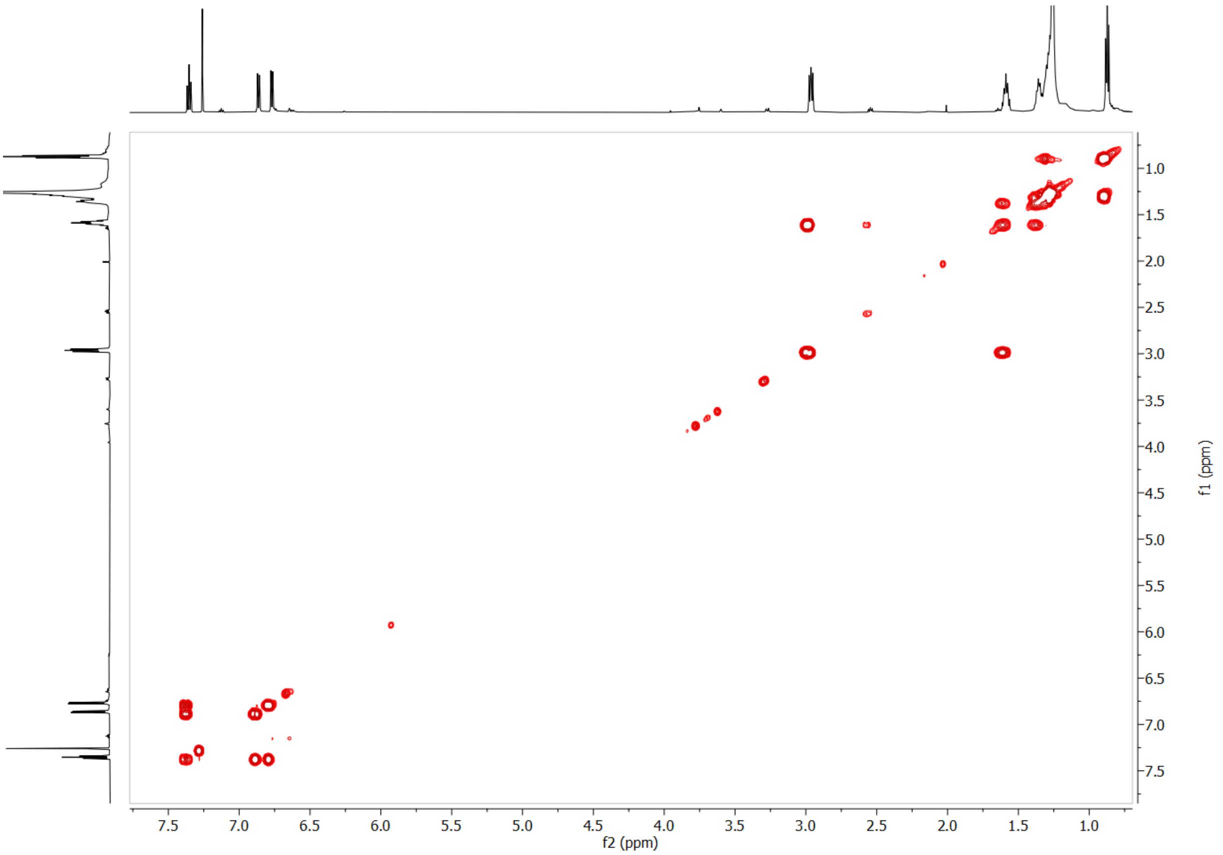

COSY NMR spectrum of **Anagigantic acid** in $CDCl_3$ at 600 MHz.

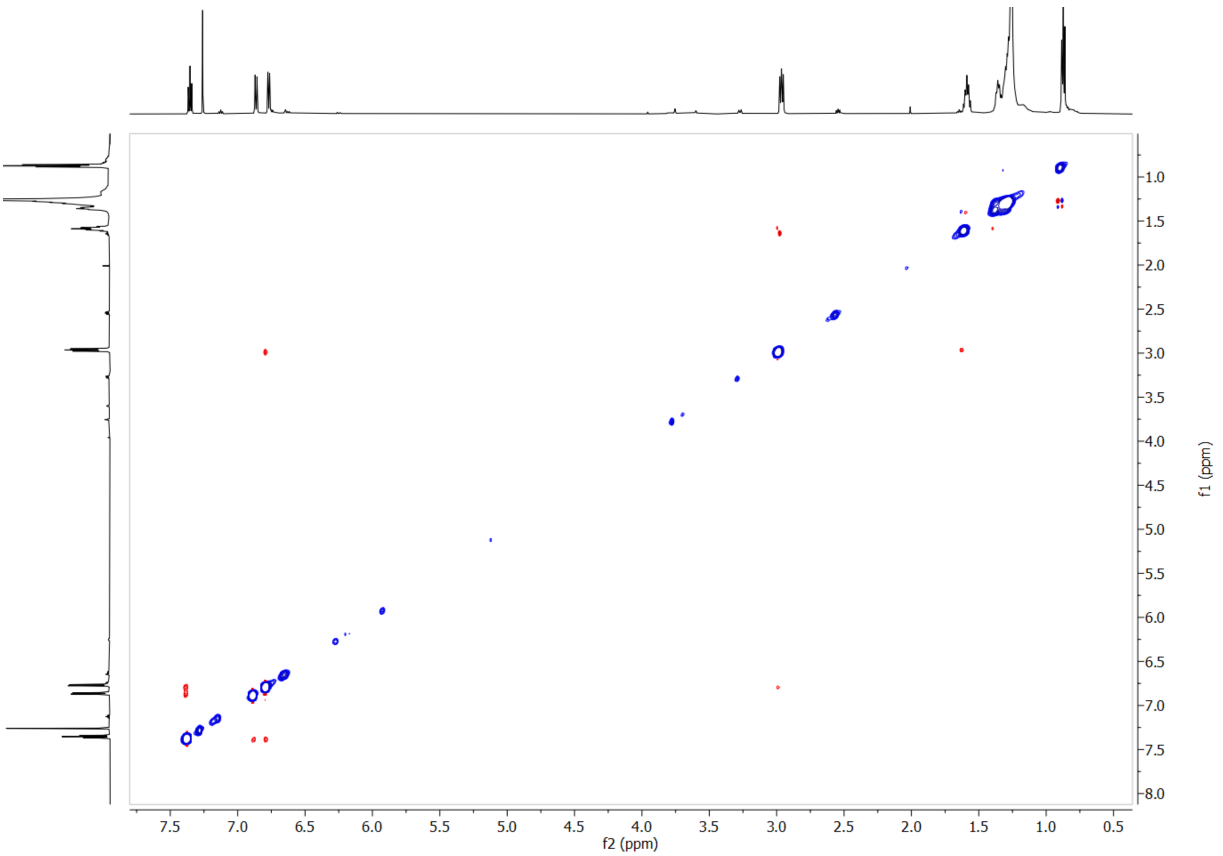

ROESY NMR spectrum of **Anagigantic acid** in $CDCl_3$ at 600 MHz.

12. 6-Tridecylsalicylic acid:

Experimental:

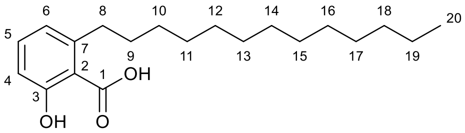

6-Tridecylsalicylic acid (**12**) Dark-green amorphous solid; UV (MeOH) λ_{max} (log ε) 225 (4.00), 311 (2.80) nm; ^{1}H NMR ($CDCl_3$, 600 MHz) δ 7.34 (1H, dd, J = 8.3, 7.5 Hz), 6.85 (1H, dd, J = 8.4, 1.2 Hz), 6.76 (1H, dd, J = 7.5, 1.2 Hz), 2.98 – 2.92 (2H, m), 1.58 (2H, p, J = 7.6 Hz), 1.35 (2H, s), 1.27 – 1.24 (18H, m), 0.88 (3H, t, J = 7.0 Hz); ^{13}C NMR ($CDCl_3$, 151 MHz) δ 174.5, 163.8, 147.7, 135.3, 122.8, 115.9, 110.5, 36.7, 32.2, 32.1, 30.0, 29.8, 29.8, 29.8, 29.8, 29.7, 29.5, 22.8, 14.3 (NP-MRD ID: NP0333026); HRESIMS *m/z* 319.2277 $[M-H]^-$ (calcd for $C_{20}H_{31}O_3^-$ 319.2279, Δ = -0.63 ppm), *m/z* 303.2318 $[M-H_2O+H]^+$ (calcd for $C_{20}H_{31}O_2^+$ 303.2319, Δ = -0.33 ppm), MS/MS spectrum: CCMSLIB00012475069.

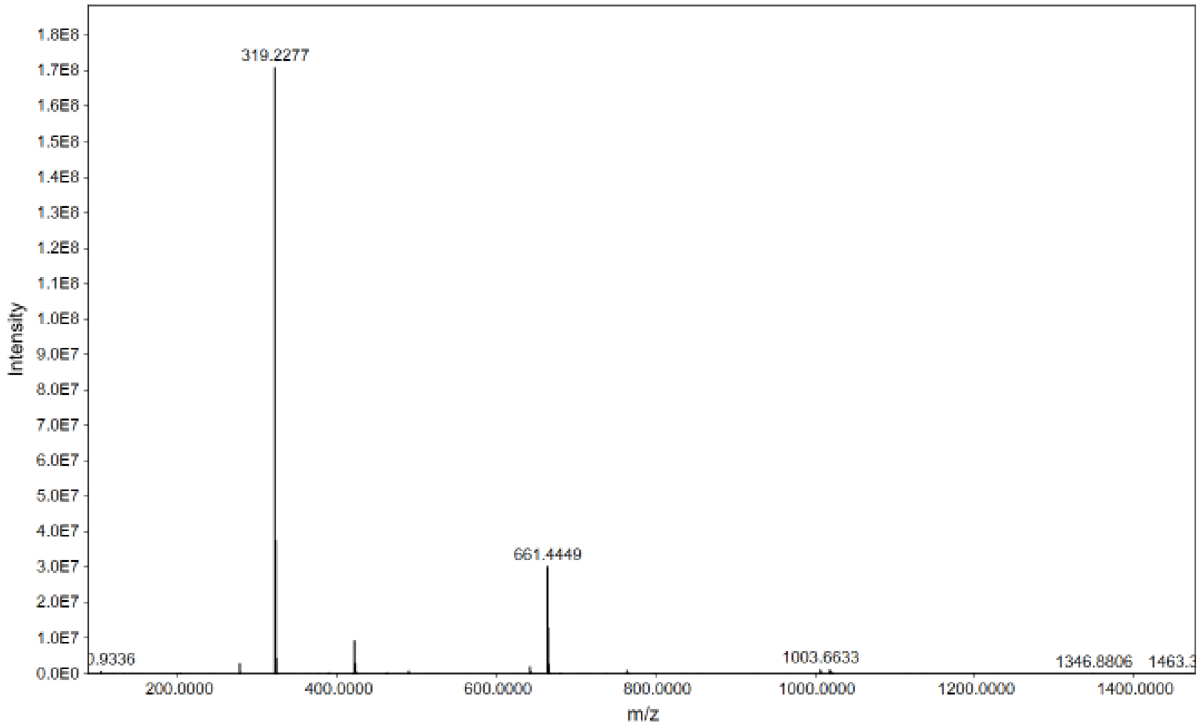

HRESIMS- spectrum of **6-Tridecylsalicylic acid** in MeOH.

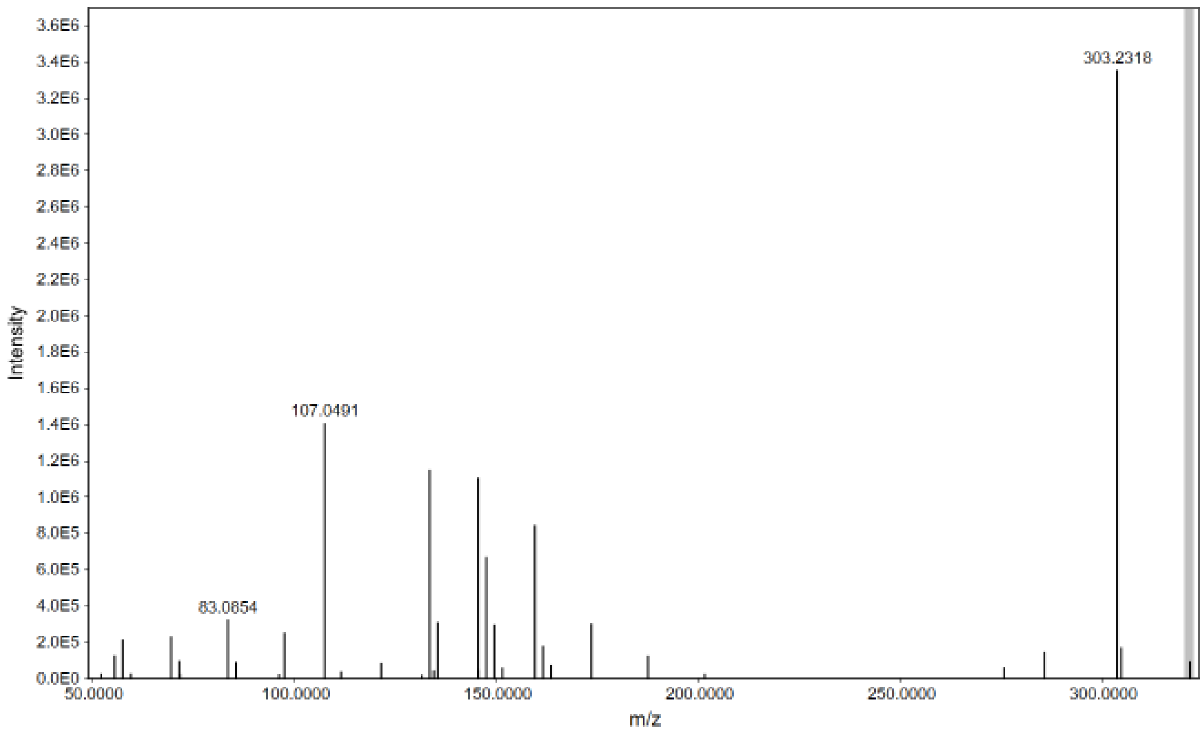

HRESIMS+ spectrum of **6-Tridecylsalicylic acid** in MeOH.

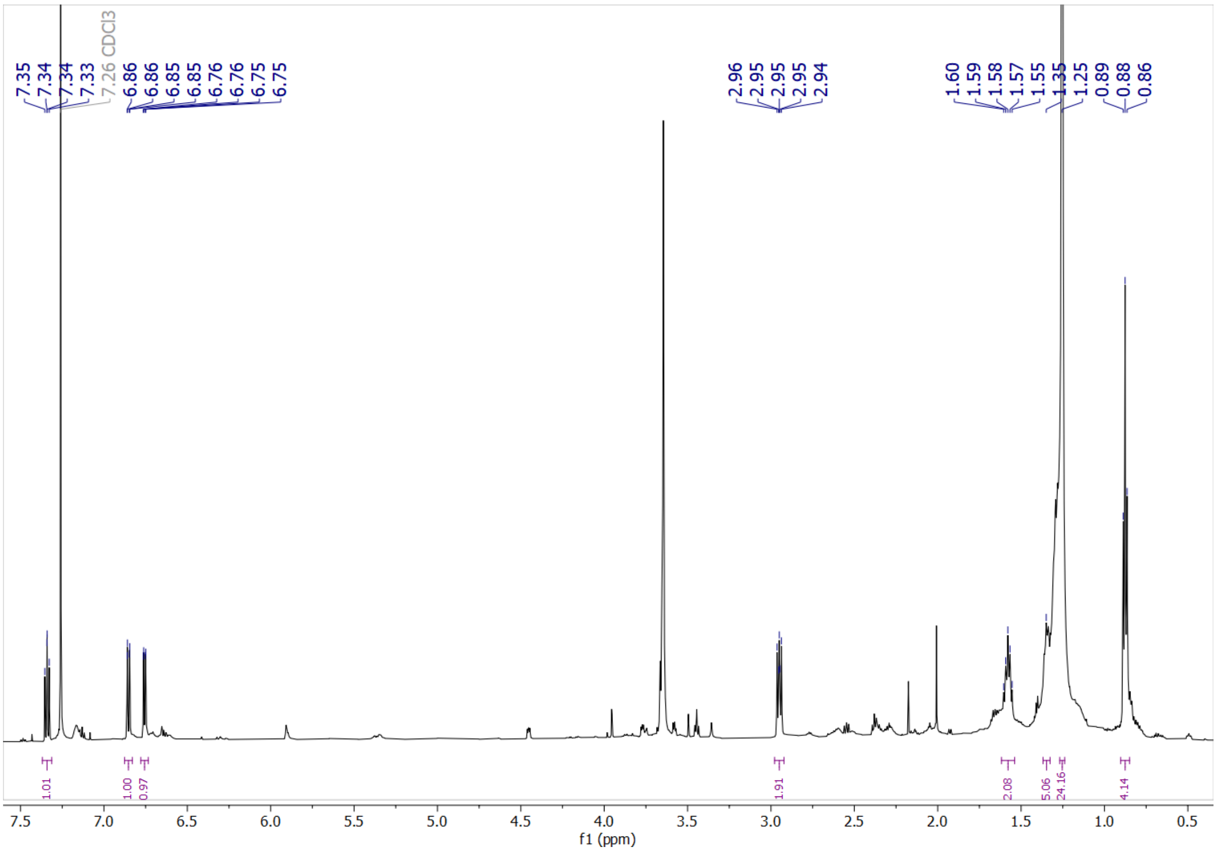

^{1}H NMR spectrum of **6-Tridecylsalicylic acid** in $CDCl_3$ at 600 MHz.

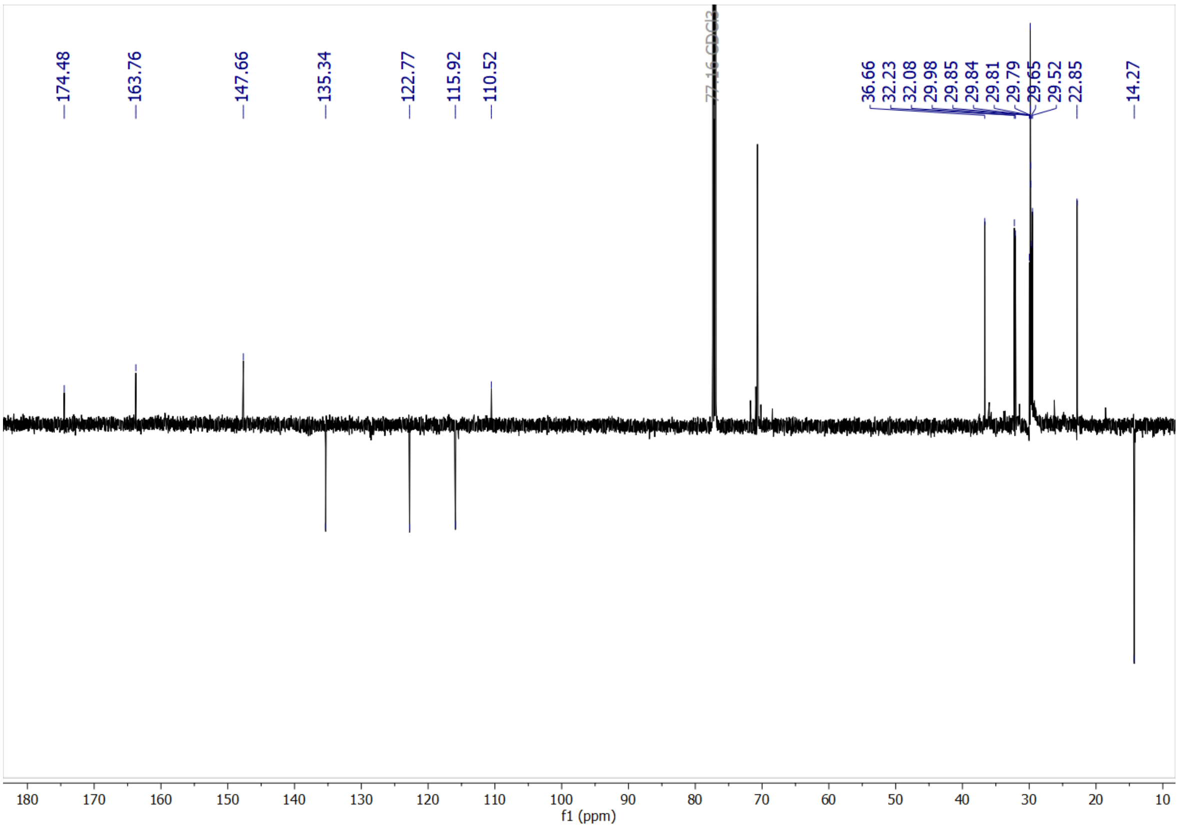

^{13}C DEPTQ NMR spectrum of **6-Tridecylsalicylic acid** in $CDCl_3$ at 151 MHz.

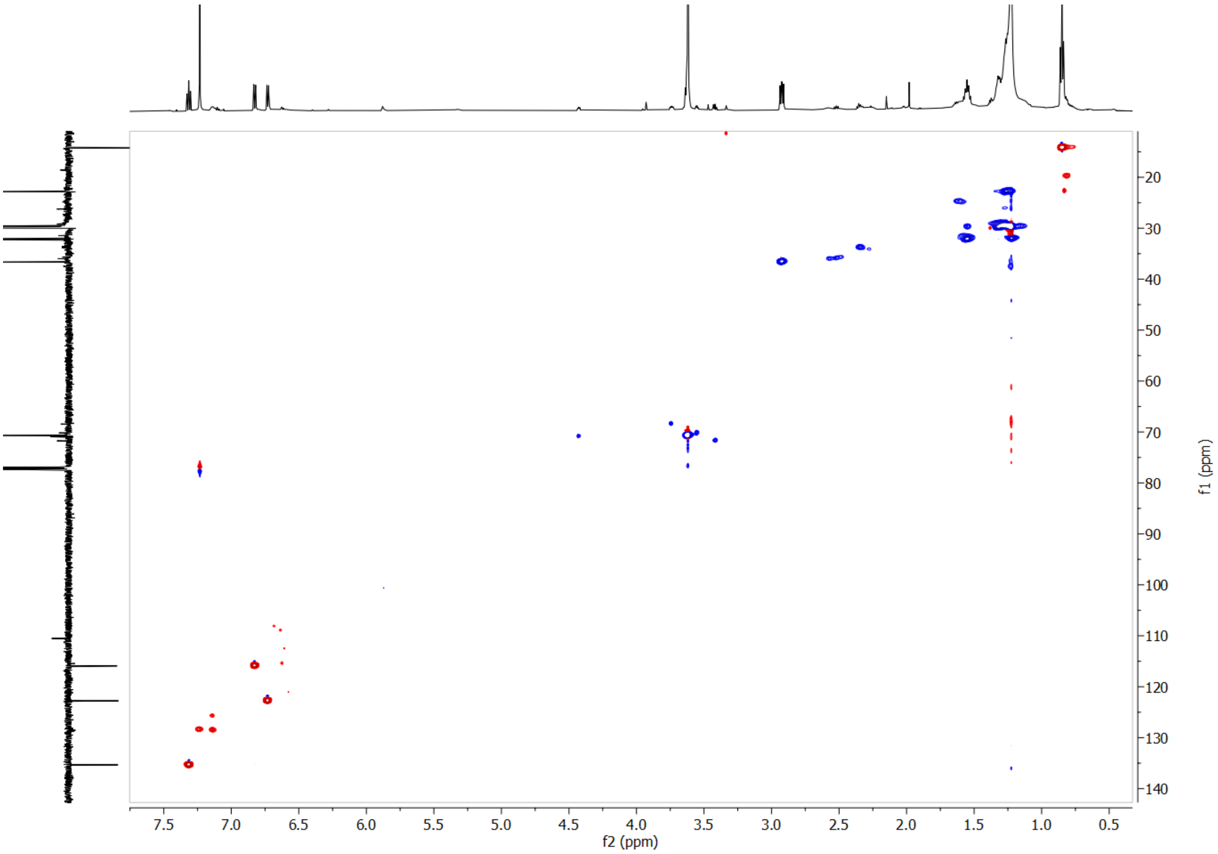

HSQC NMR spectrum of **6-Tridecylsalicylic acid** in $CDCl_3$ at 600 MHz.

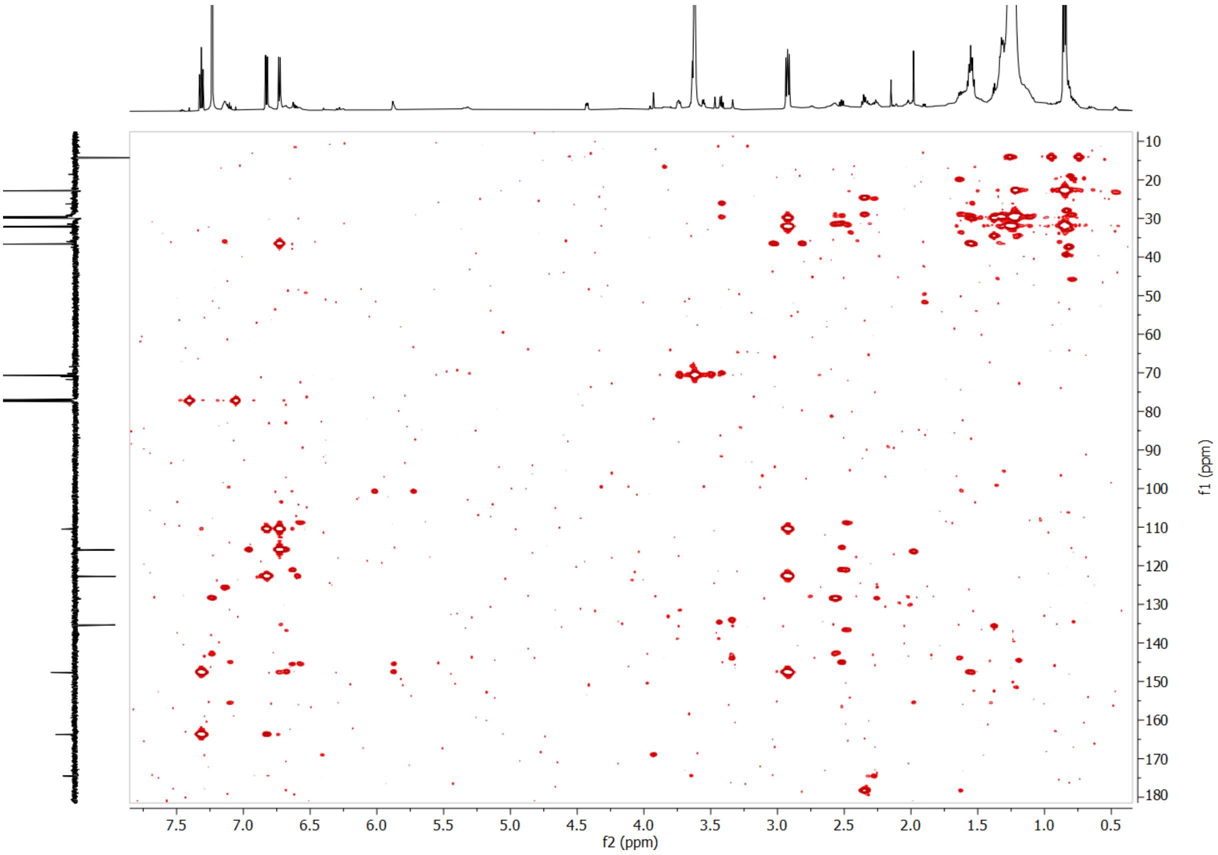

HMBC NMR spectrum of **6-Tridecylsalicylic acid** in $CDCl_3$ at 600 MHz.

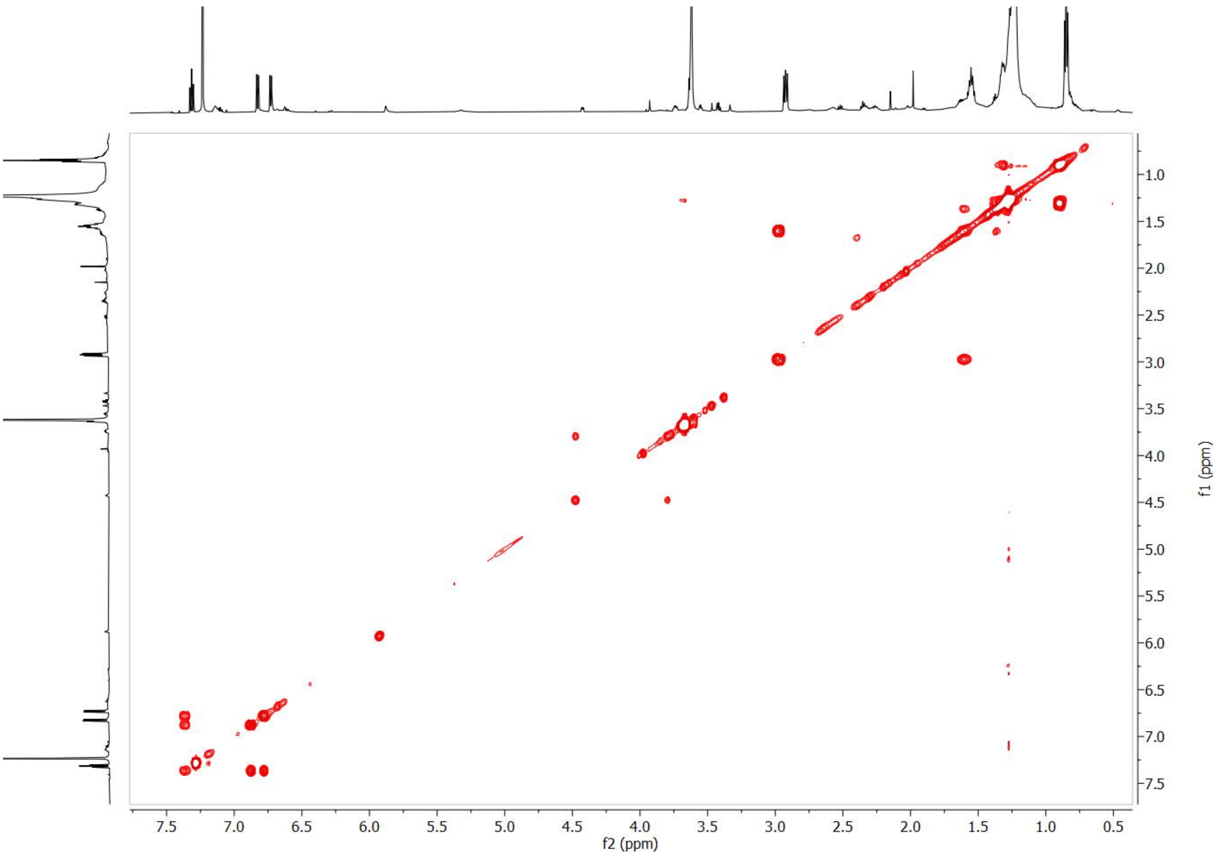

COSY NMR spectrum of **6-Tridecylsalicylic acid** in $CDCl_3$ at 600 MHz.

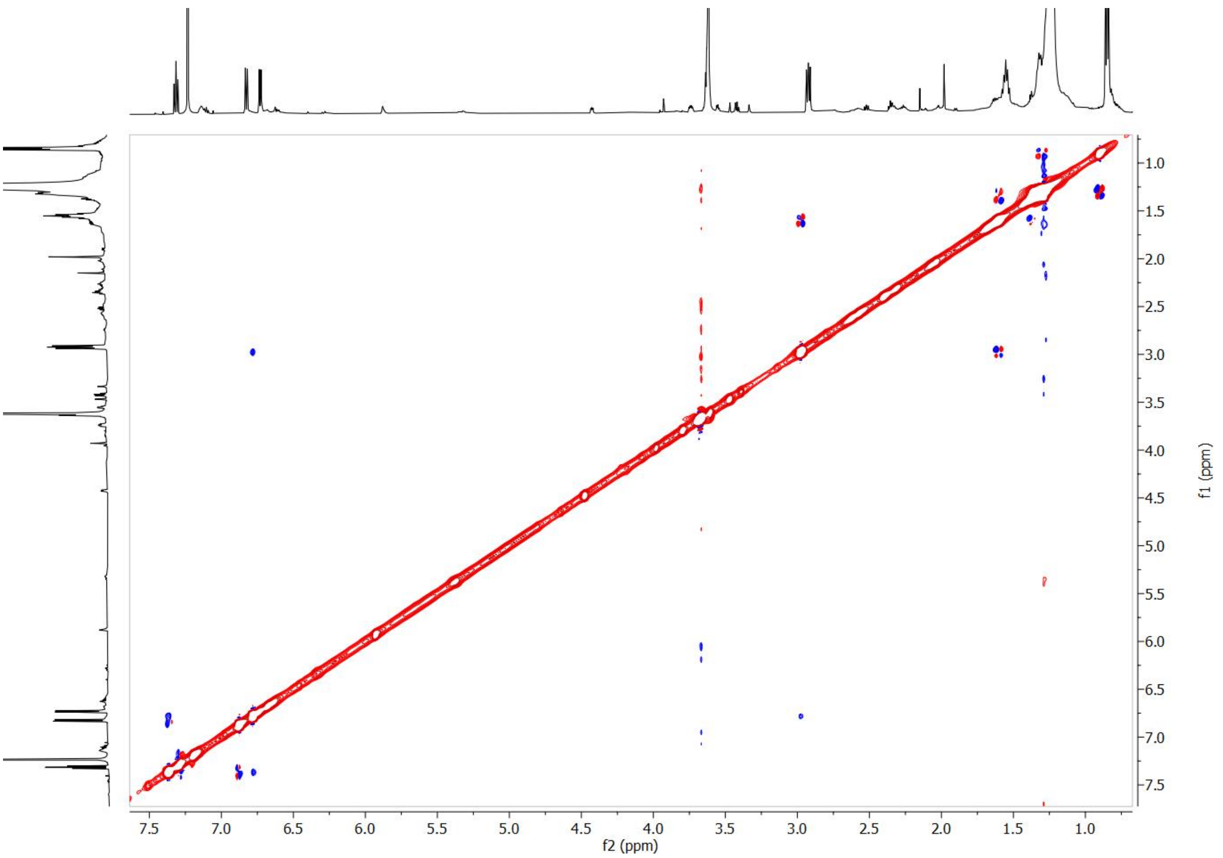

ROESY NMR spectrum of **6-Tridecylsalicylic acid** in $CDCl_3$ at 600 MHz.

Synthetic derivatives of 2,4-dihydroxyacetophenone

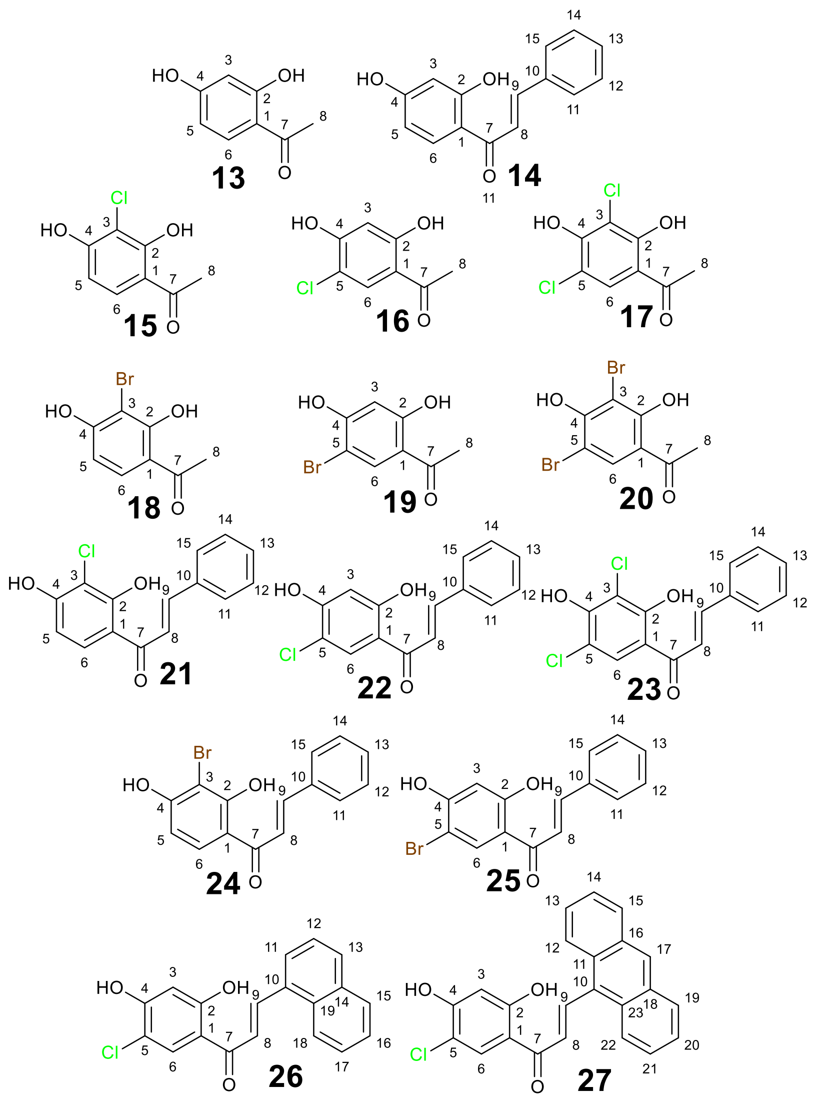

13. 2,4-dihydroxyacetophenone:

Experimental:

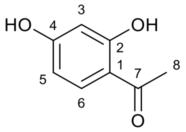

2,4-dihydroxyacetophenone (**13**) white solid; ^{1}H NMR (DMSO-d_6, 600 MHz) δ 12.60 (1H, s), 10.61 (1H, s), 7.75 (1H, d, J = 8.8 Hz), 6.37 (1H, dd, J = 8.8, 2.4 Hz), 6.24 (1H, d, J = 2.3 Hz), 2.52 (3H, s); ^{13}C NMR (DMSO-d_6, 151 MHz) δ 202.7, 164.9, 164.2, 133.7, 112.9, 108.1, 102.3, 26.4; HRESIMS *m/z* 153.0545 $[M+H]^+$ (calcd for $C_8H_9O_3^+$ 153.0546), *m/z* 151.0400 $[M-H]^-$ (calcd for $C_8H_7O_3^-$ 151.0401).

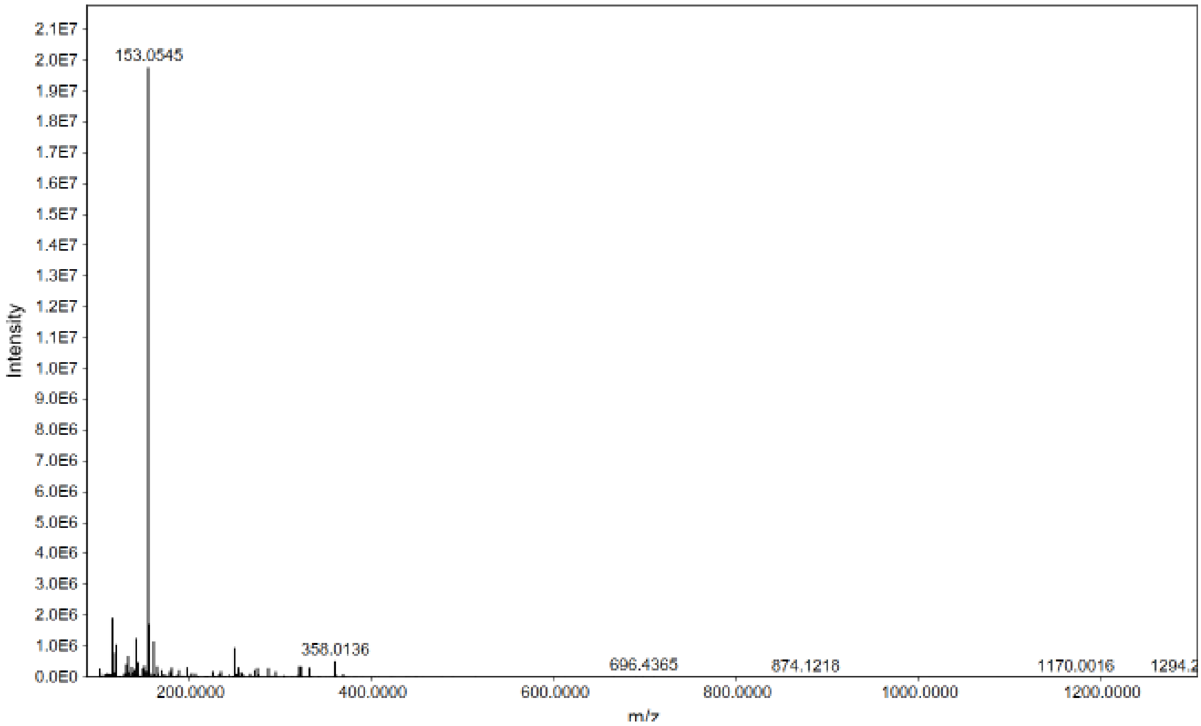

HRESIMS+ spectrum of **2,4-dihydroxyacetophenone** in MeOH.

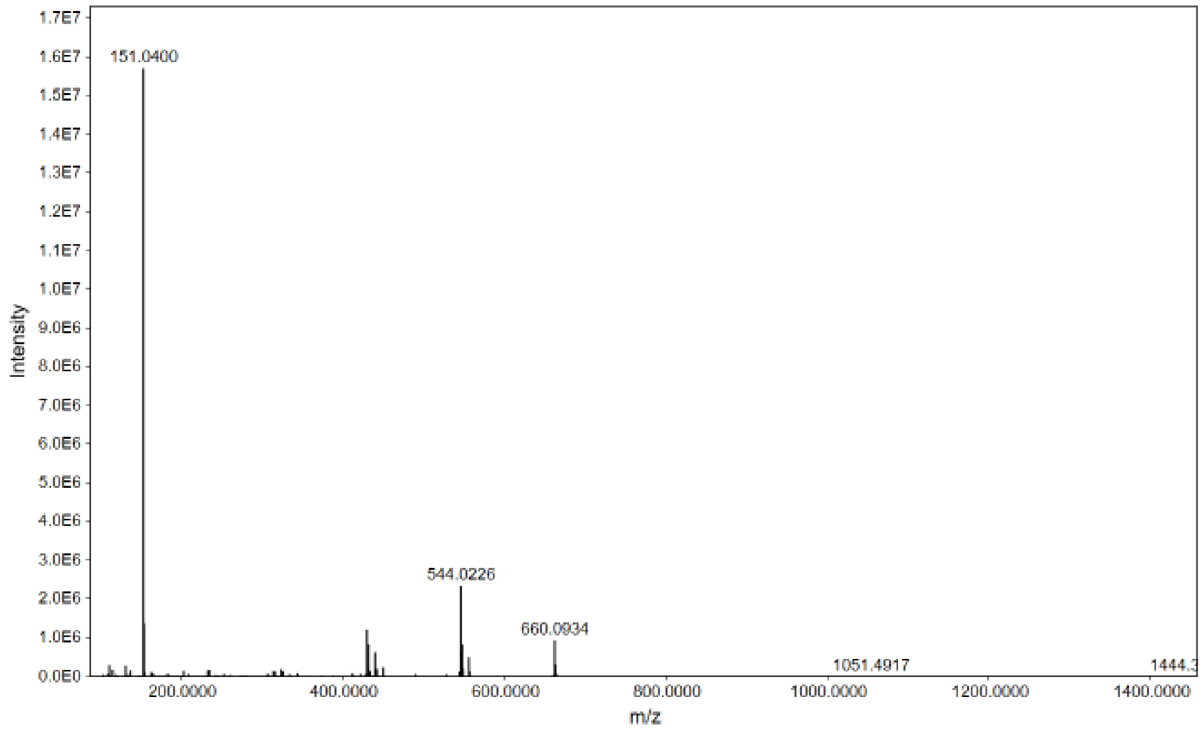

HRESIMS- spectrum of **2,4-dihydroxyacetophenone** in MeOH.

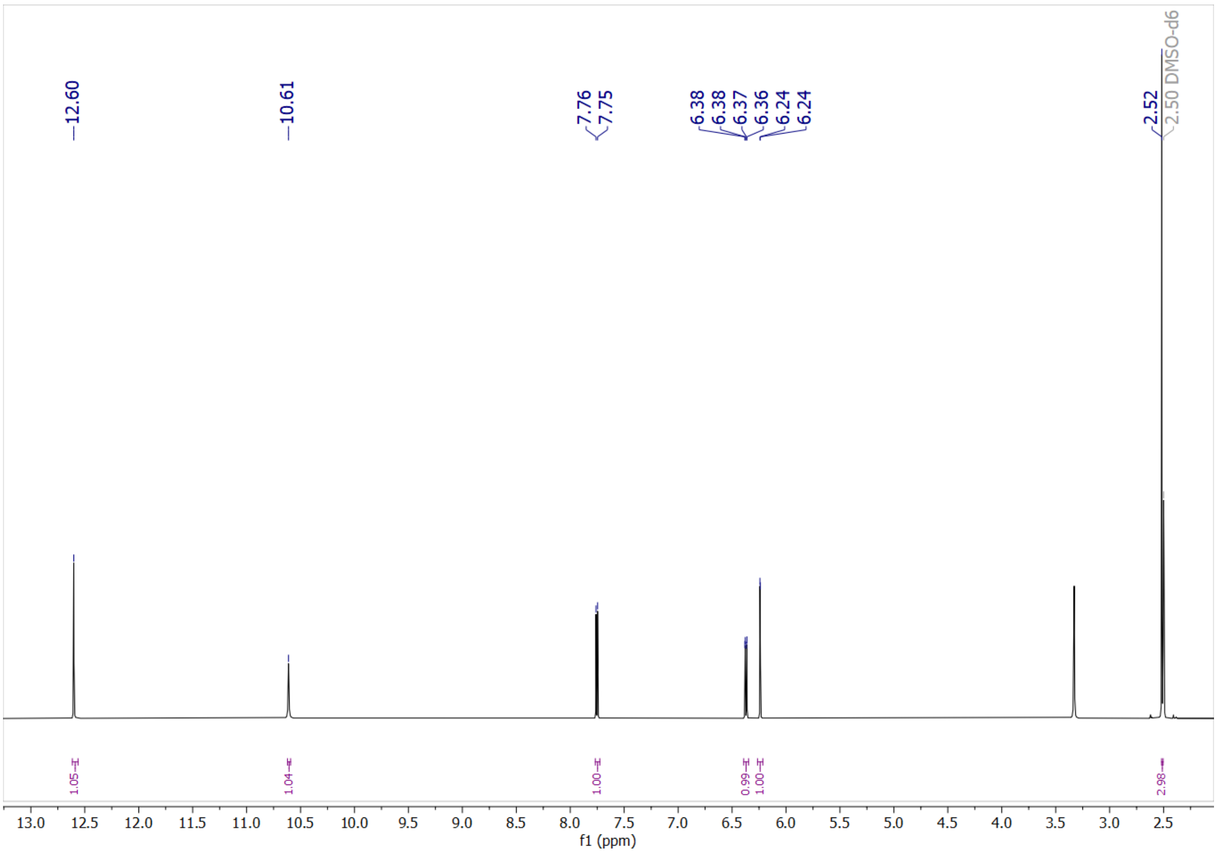

^{1}H NMR spectrum of **2,4-dihydroxyacetophenone** in DMSO-d_6 at 600 MHz.

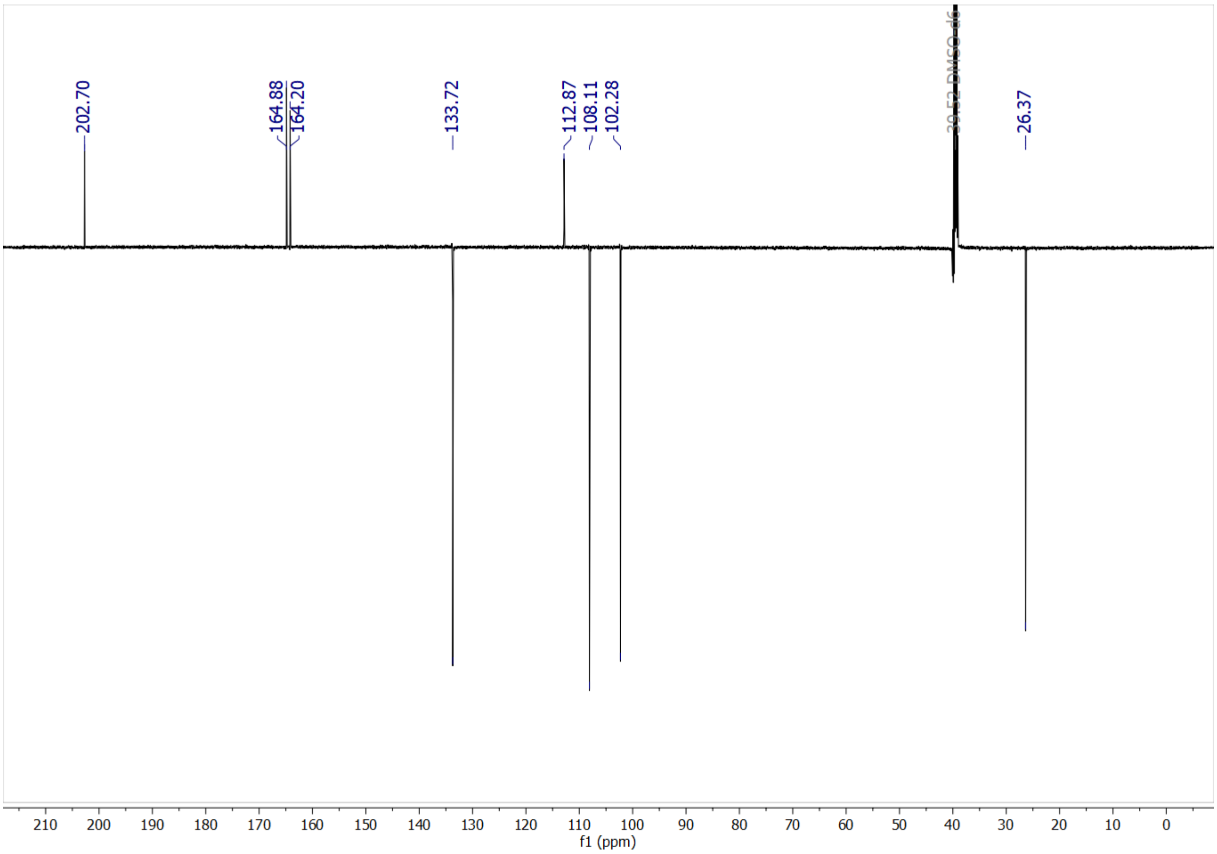

^{13}C DEPTQ NMR spectrum of **2,4-dihydroxyacetophenone** in DMSO-d_6 at 151 MHz.

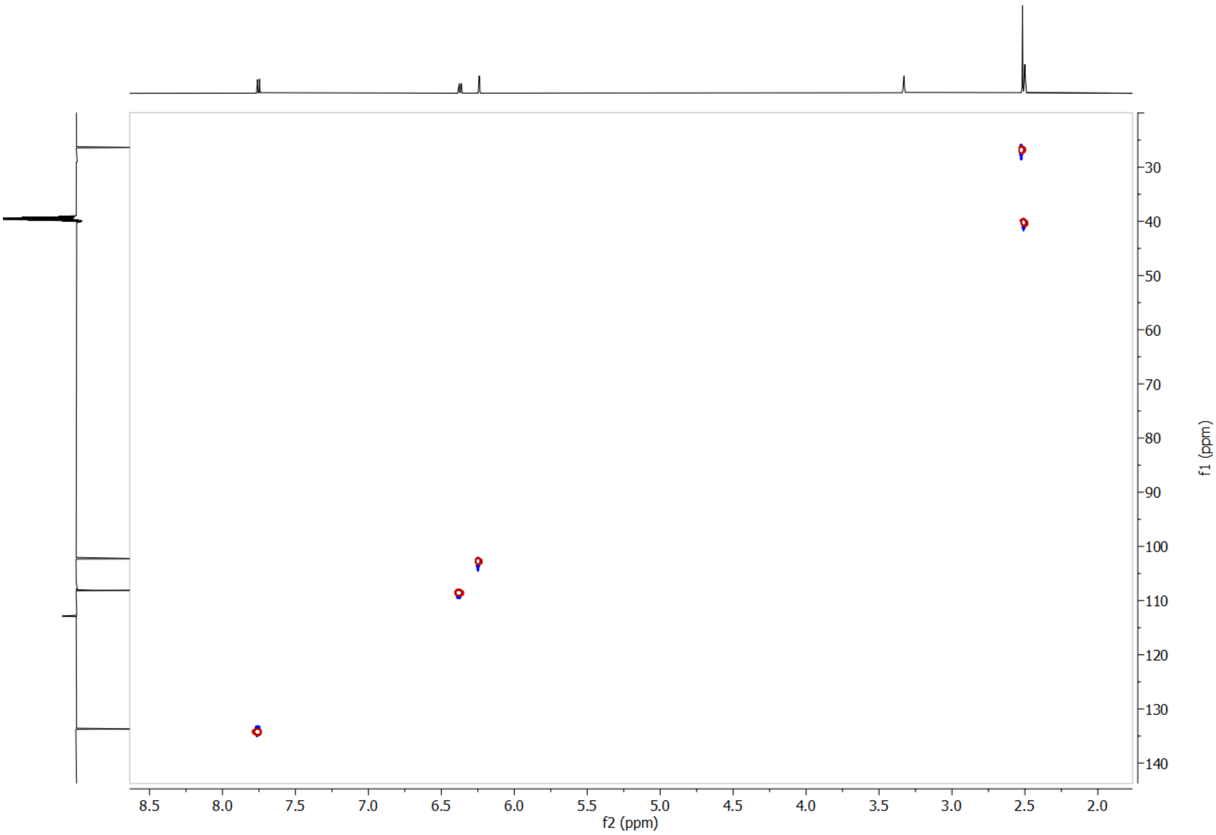

HSQC NMR spectrum of **2,4-dihydroxyacetophenone** in DMSO-d_6 at 600 MHz.

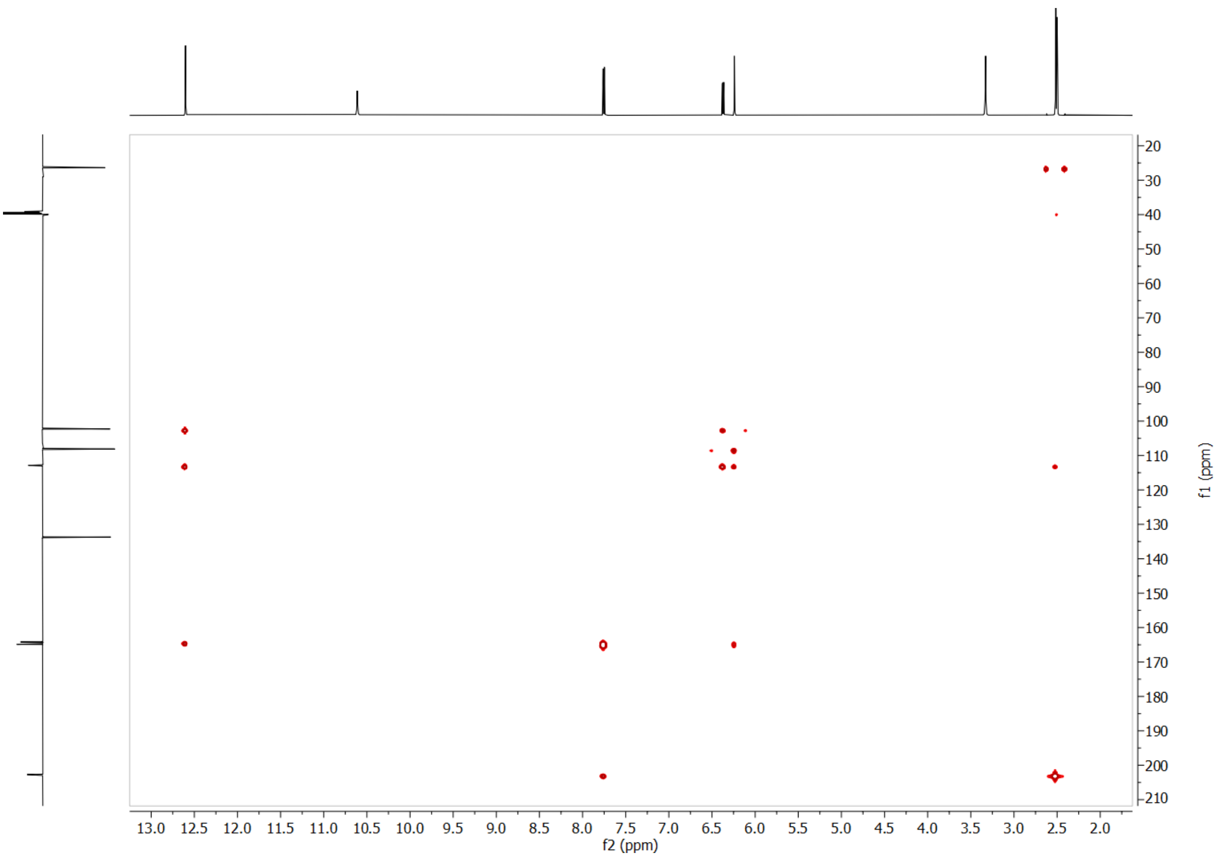

HMBC NMR spectrum of **2,4-dihydroxyacetophenone** in DMSO-d_6 at 600 MHz.

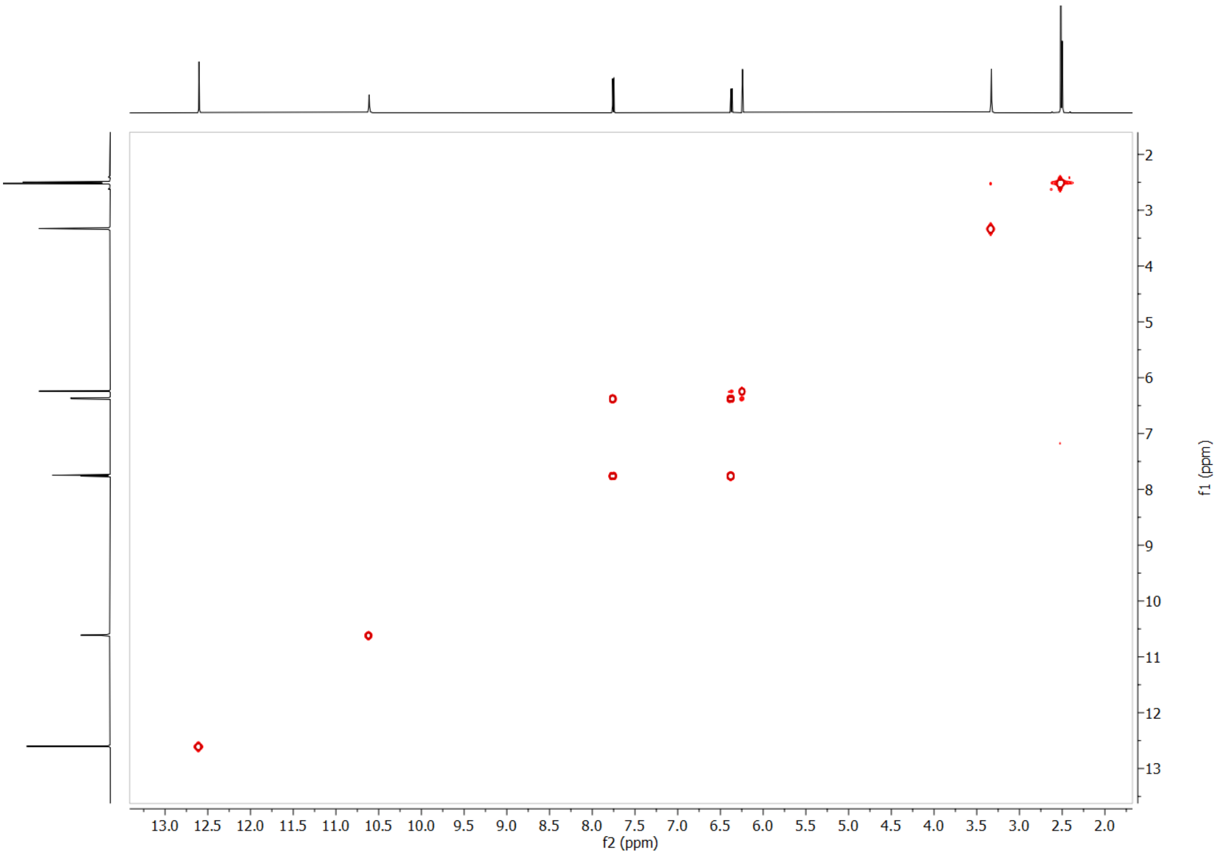

COSY NMR spectrum of **2,4-dihydroxyacetophenone** in DMSO-d_6 at 600 MHz.

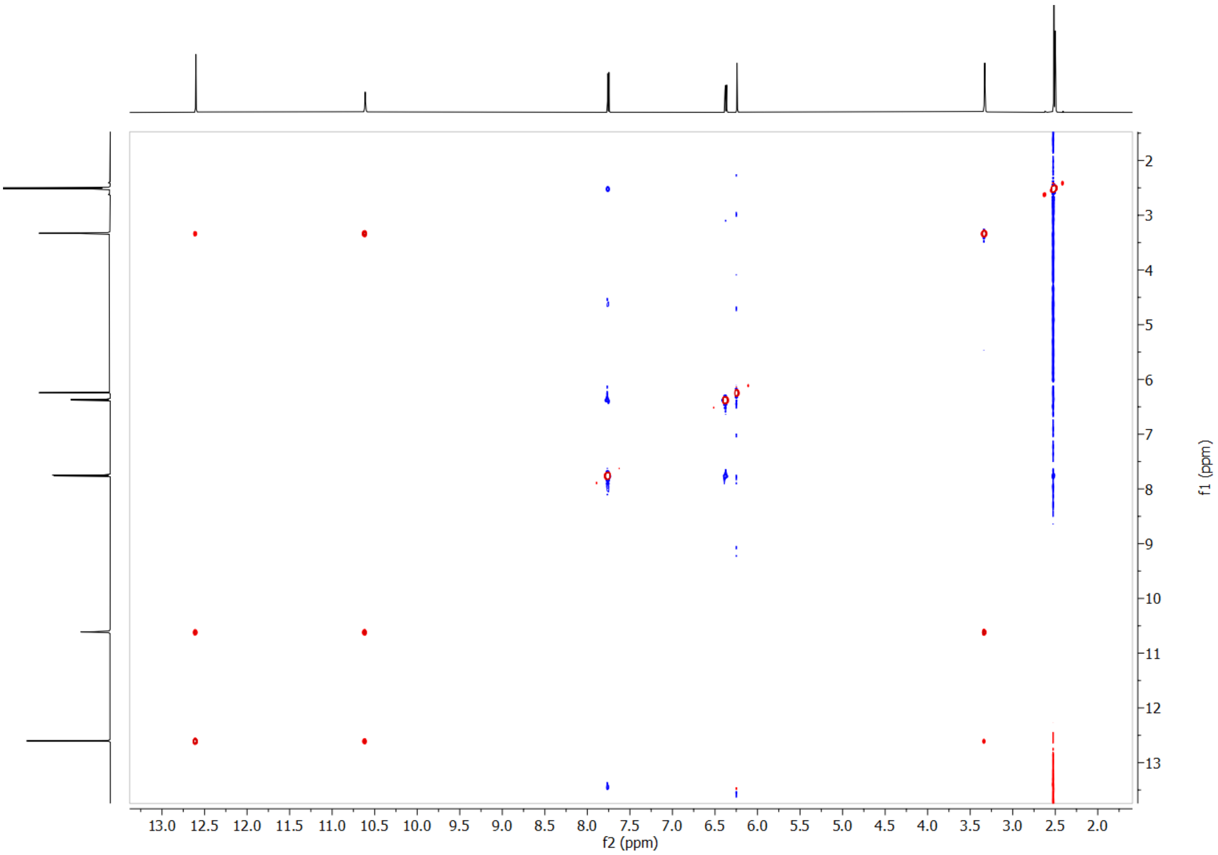

ROESY NMR spectrum of **2,4-dihydroxyacetophenone** in DMSO-d_6 at 600 MHz.

14. 2,4-dihydroxychalcone:

Experimental:

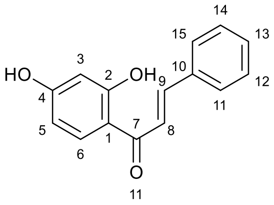

2,4-dihydroxychalcone (**14**) yellow solid; ^{1}H NMR (DMSO-d_6, 600 MHz) δ 13.38 (1H, d, J = 1.6 Hz), 10.75 (1H, s), 8.21 (1H, dd, J = 9.0, 3.3 Hz), 7.98 (1H, dd, J = 15.5, 3.4 Hz), 7.90 (2H, td, J = 4.9, 2.8 Hz), 7.80 (1H, dd, J = 15.4, 2.8 Hz), 7.47 (3H, td, J = 4.6, 2.0 Hz), 6.43 (1H, dt, J = 8.9, 2.2 Hz), 6.30 (1H, t, J = 2.0 Hz); ^{13}C NMR (DMSO-d_6, 151 MHz) δ 191.5, 165.8, 165.3, 143.7, 134.6, 133.2, 130.7, 129.0, 128.9, 121.3, 113.0, 108.3, 102.6; HRESIMS *m/z* 241.0856 $[M+H]^+$ (calcd for $C_{15}H_{13}O_3^+$ 241.0859), *m/z* 239.0711 $[M-H]^-$ (calcd for $C_{15}H_{11}O_3^-$ 239.0714).

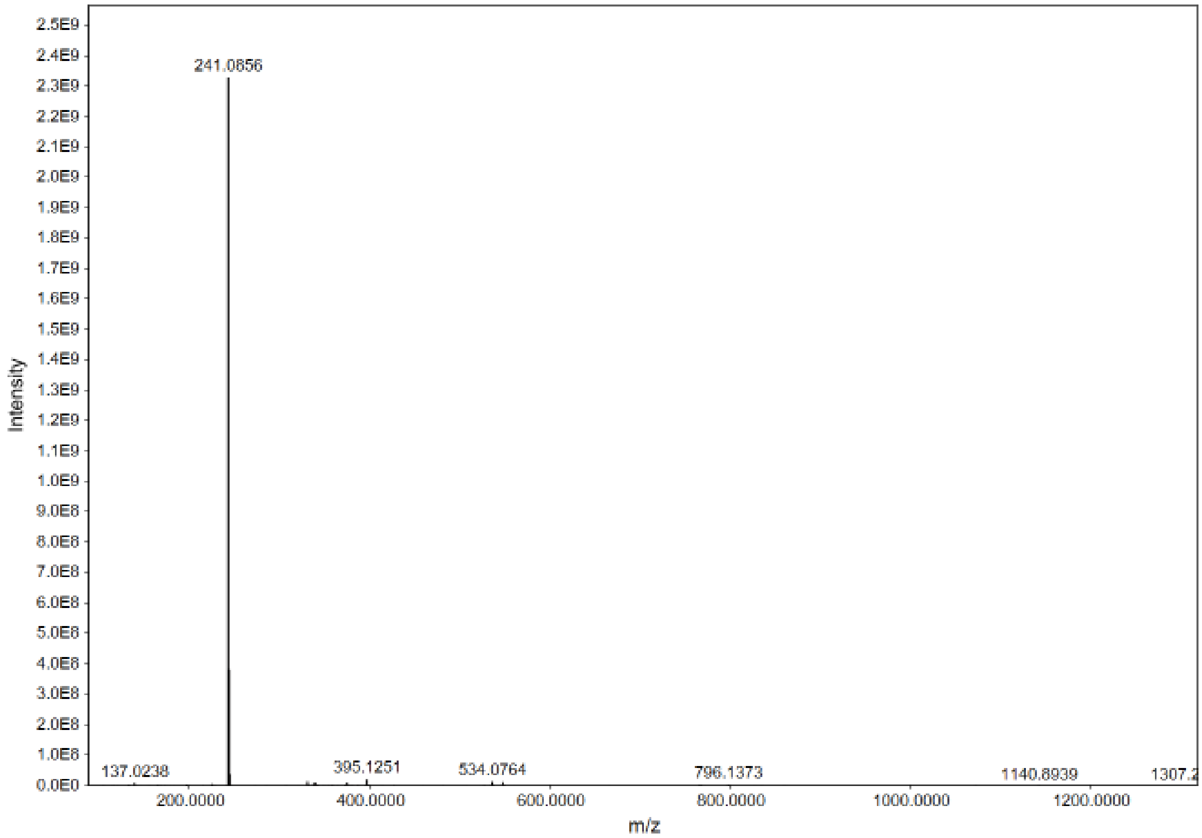

HRESIMS+ spectrum of **2,4-dihydroxychalcone** in MeOH.

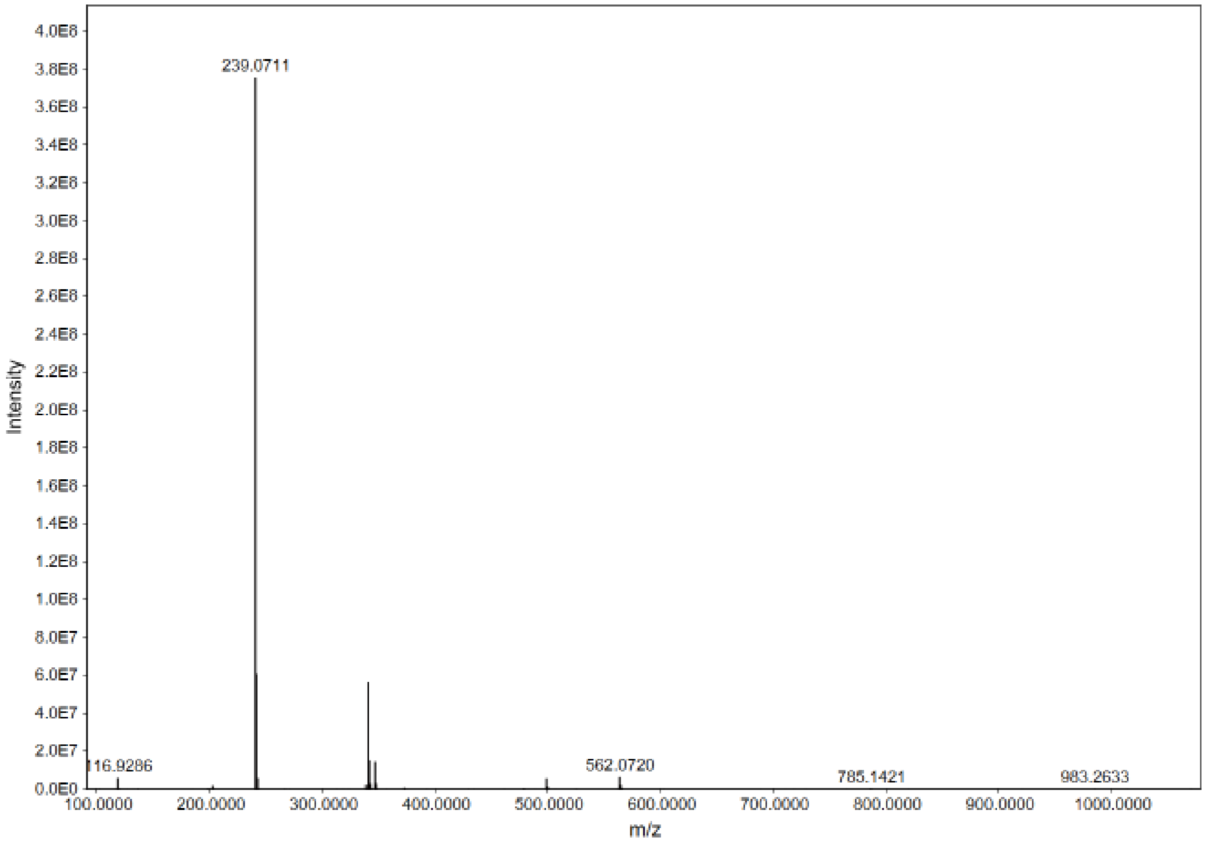

HRESIMS- spectrum of **2,4-dihydroxychalcone** in MeOH.

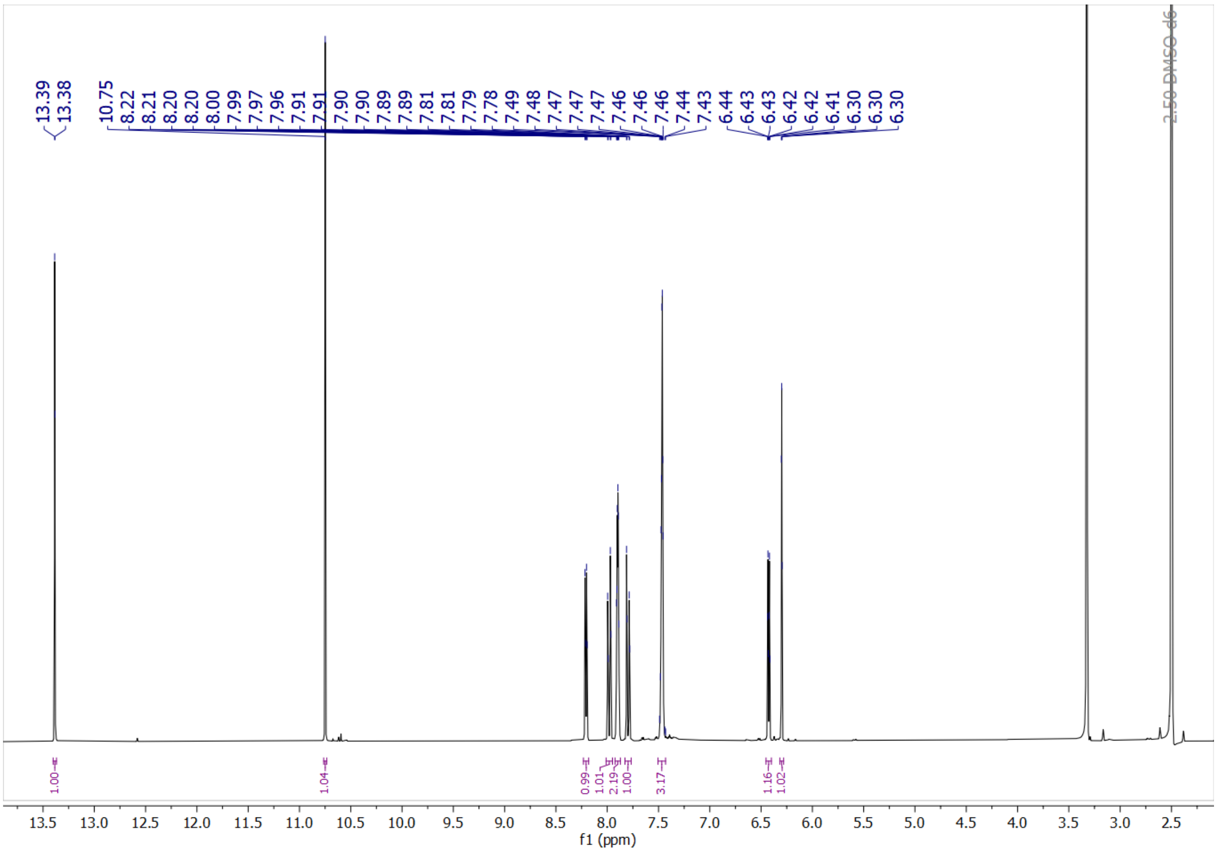

^{1}H NMR spectrum of **2,4-dihydroxychalcone** in DMSO-d_6 at 600 MHz.

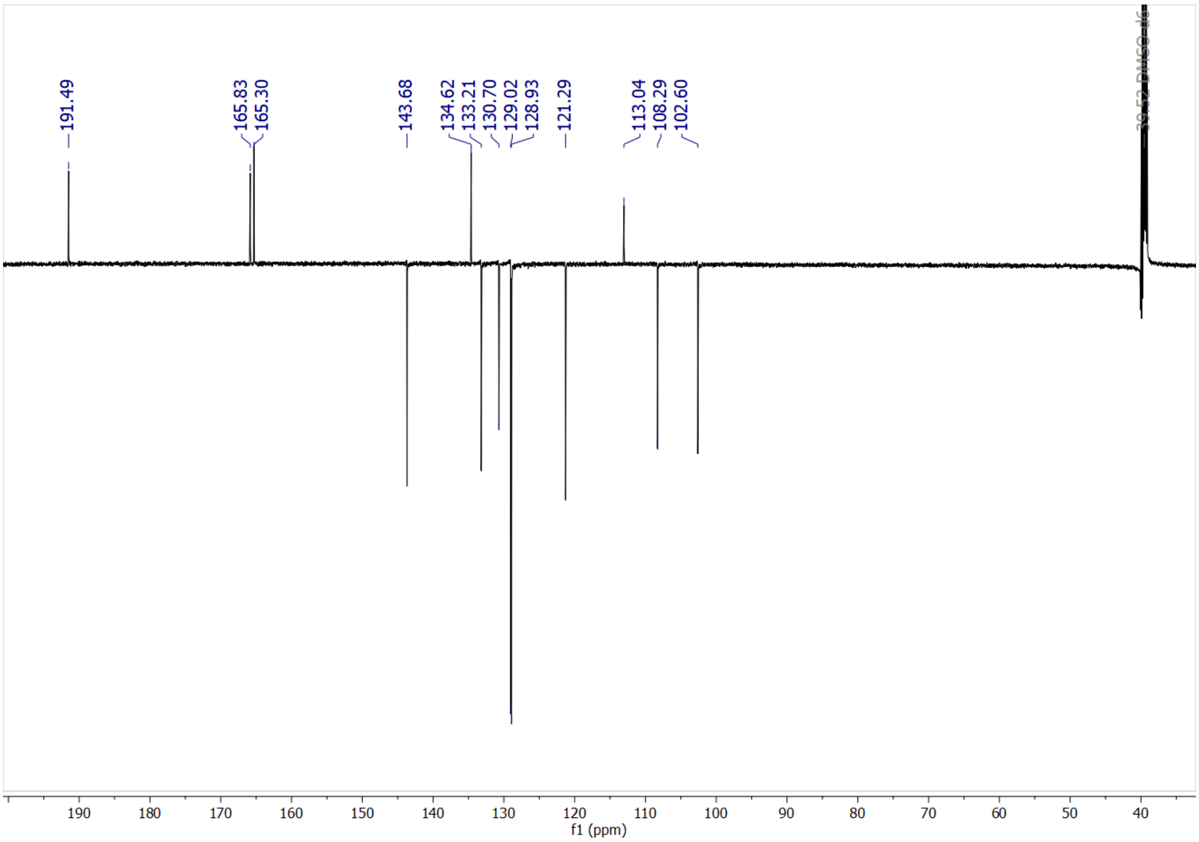

^{13}C DEPTQ NMR spectrum of **2,4-dihydroxychalcone** in DMSO-d_6 at 151 MHz.

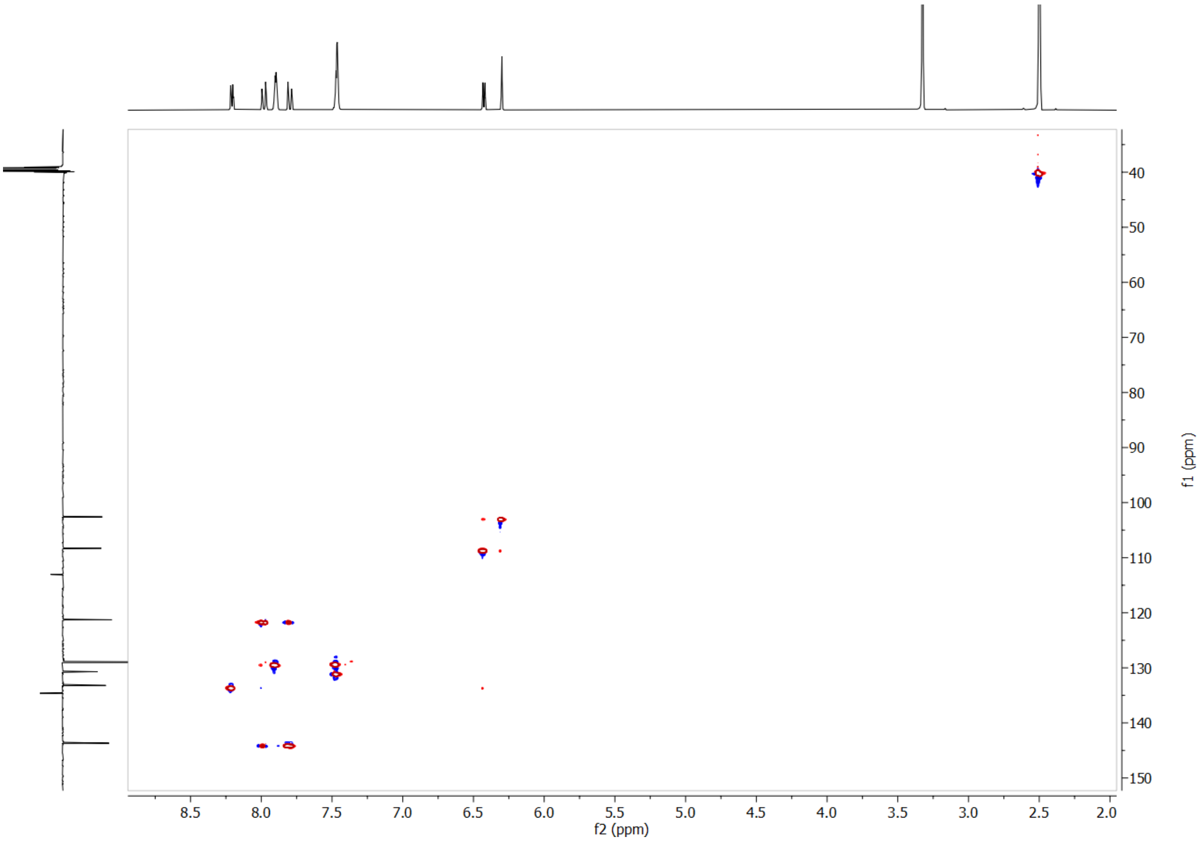

HSQC NMR spectrum of **2,4-dihydroxychalcone** in DMSO-d_6 at 600 MHz.

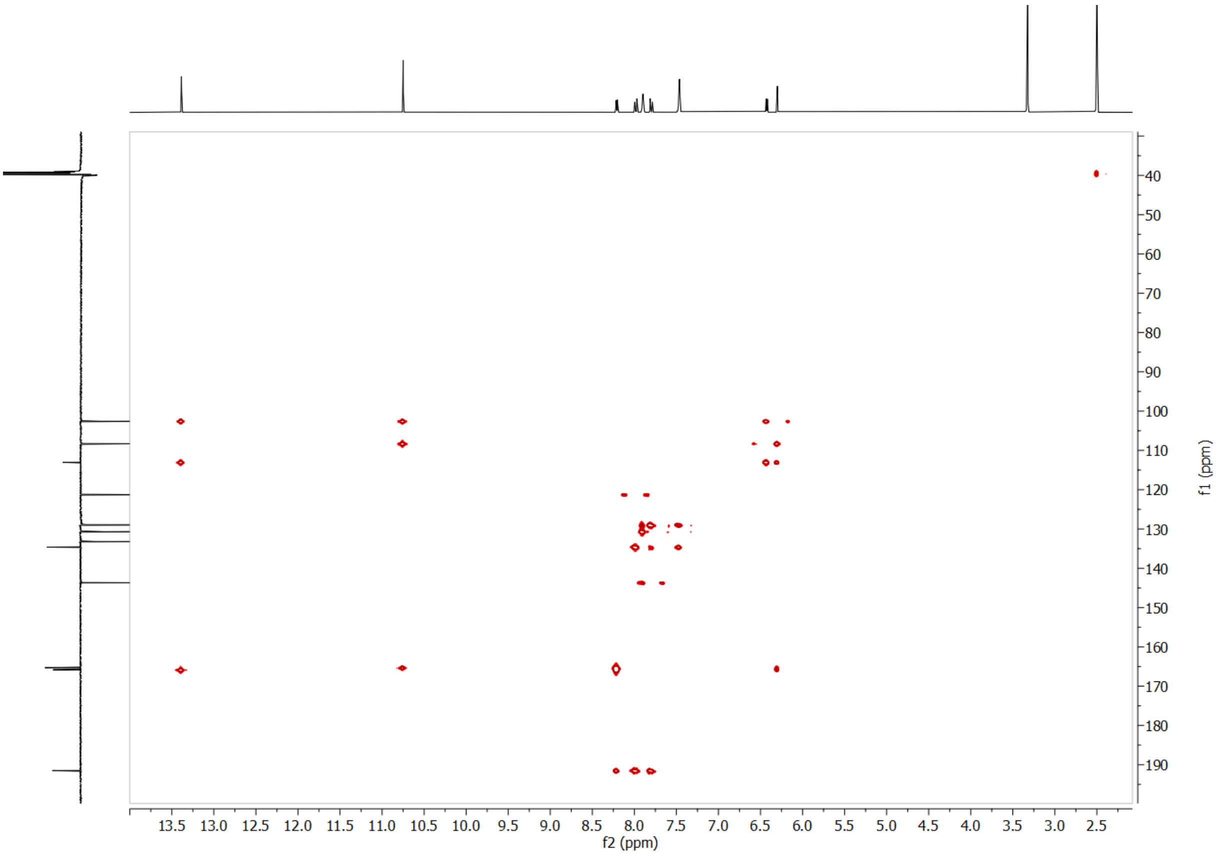

HMBC NMR spectrum of **2,4-dihydroxychalcone** in DMSO-d_6 at 600 MHz.

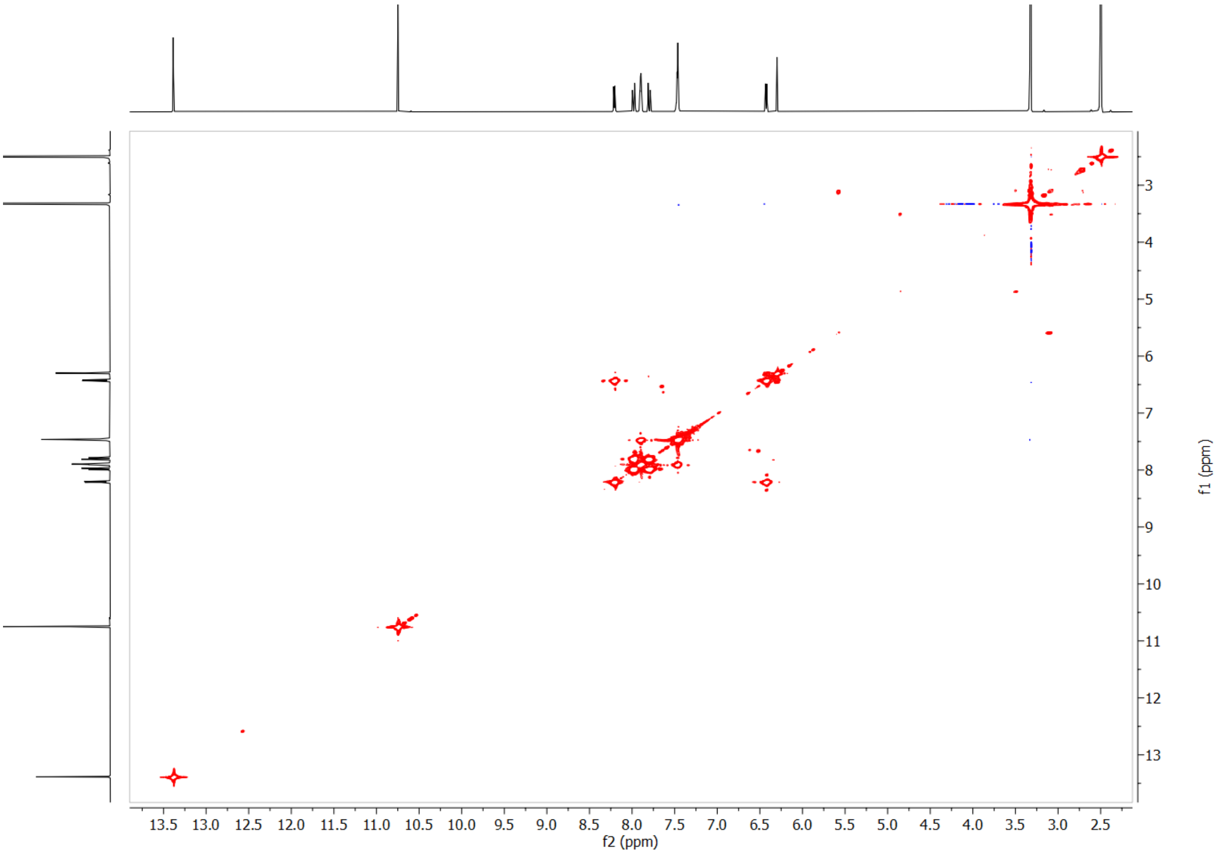

COSY NMR spectrum of **2,4-dihydroxychalcone** in DMSO-d_6 at 600 MHz.

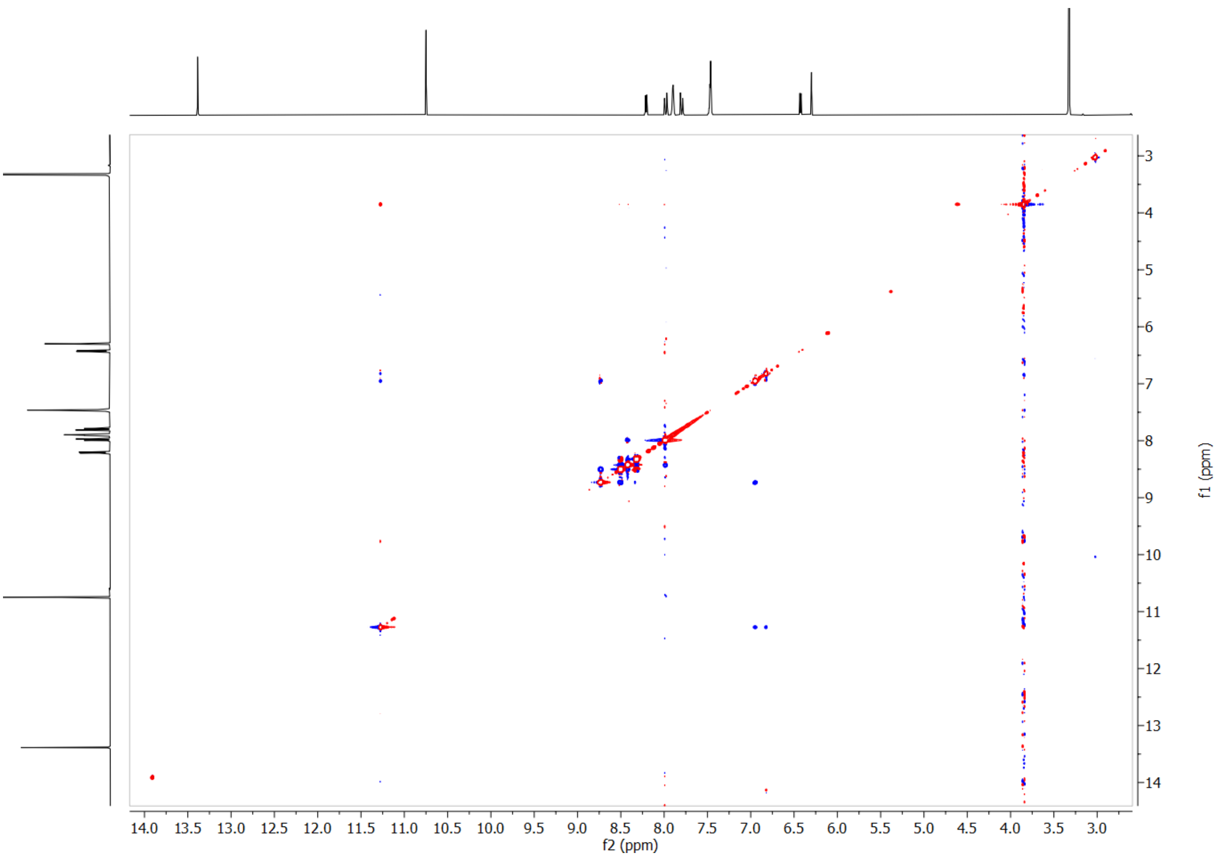

ROESY NMR spectrum of **2,4-dihydroxychalcone** in DMSO-d_6 at 600 MHz.

15. 3-chloro-2,4-dihydroxyacetophenone:

Experimental:

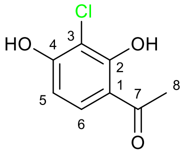

3-chloro-2,4-dihydroxyacetophenone (**15**) white solid; ^{1}H NMR (DMSO-d_6, 600 MHz) δ 13.36 (1H, s), 7.77 (1H, dd, J = 9.0, 0.9 Hz), 6.59 (1H, d, J = 8.9 Hz), 2.57 (3H, s); ^{13}C NMR (DMSO-d_6, 151 MHz) δ 203.4, 160.5, 159.9, 131.2, 113.0, 107.7, 106.7, 26.3; HRESIMS *m/z* 187.0154 $[M+H]^+$ (calcd for $C_8H_8ClO_3^+$ 187.0156), *m/z* 185.0010 $[M-H]^-$ (calcd for $C_8H_6ClO_3^-$ 185.0011).

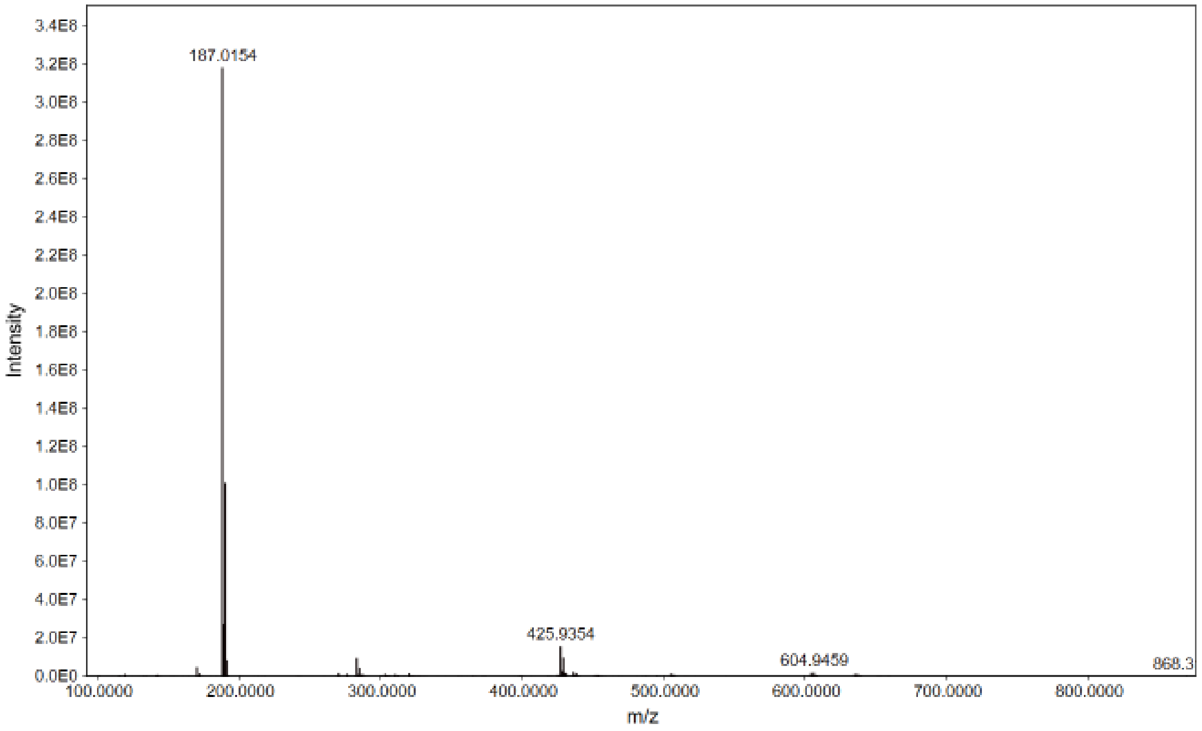

HRESIMS+ spectrum of **3-chloro-2,4-dihydroxyacetophenone** in MeOH.

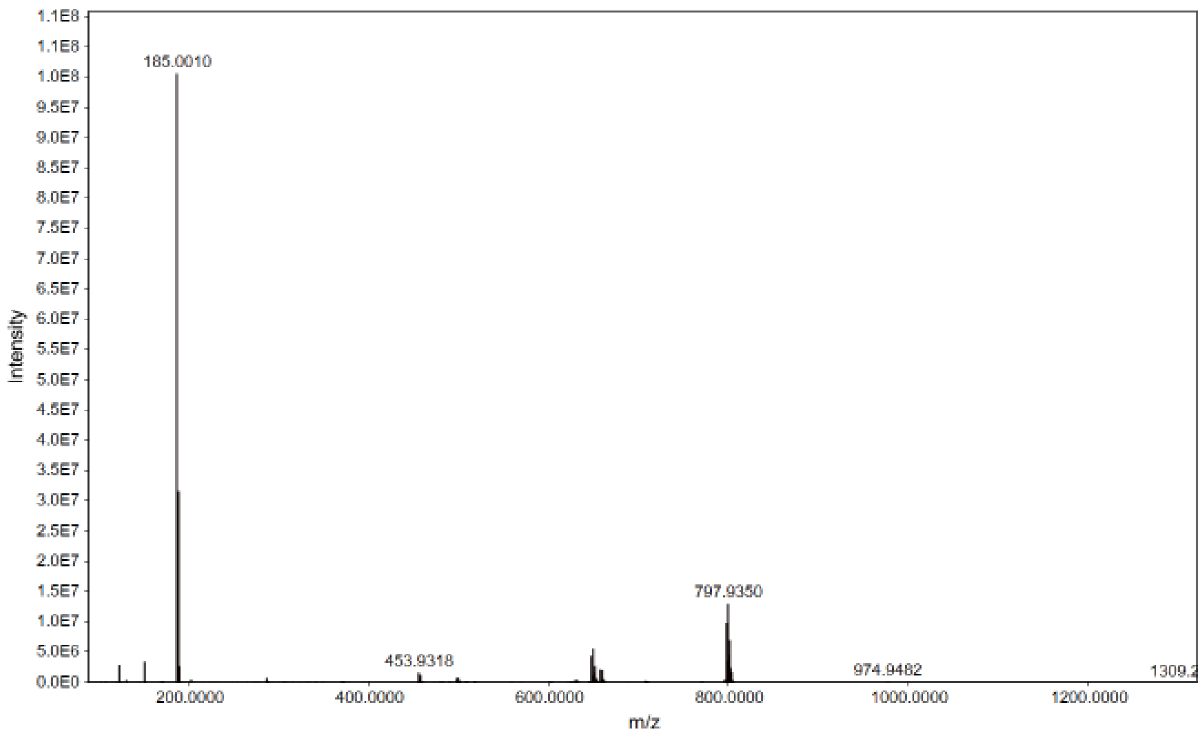

HRESIMS- spectrum of **3-chloro-2,4-dihydroxyacetophenone** in MeOH.

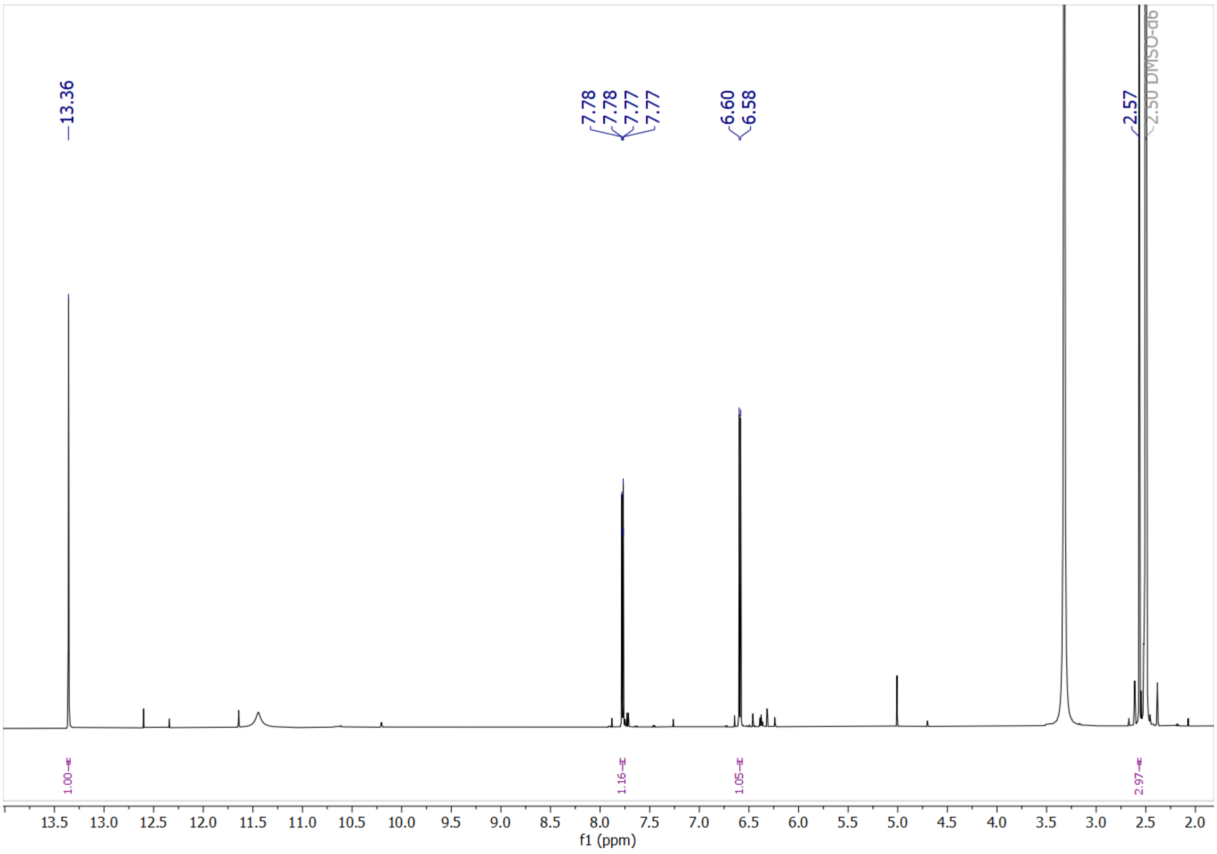

^{1}H NMR spectrum of **3-chloro-2,4-dihydroxyacetophenone** in DMSO-d_6 at 600 MHz.

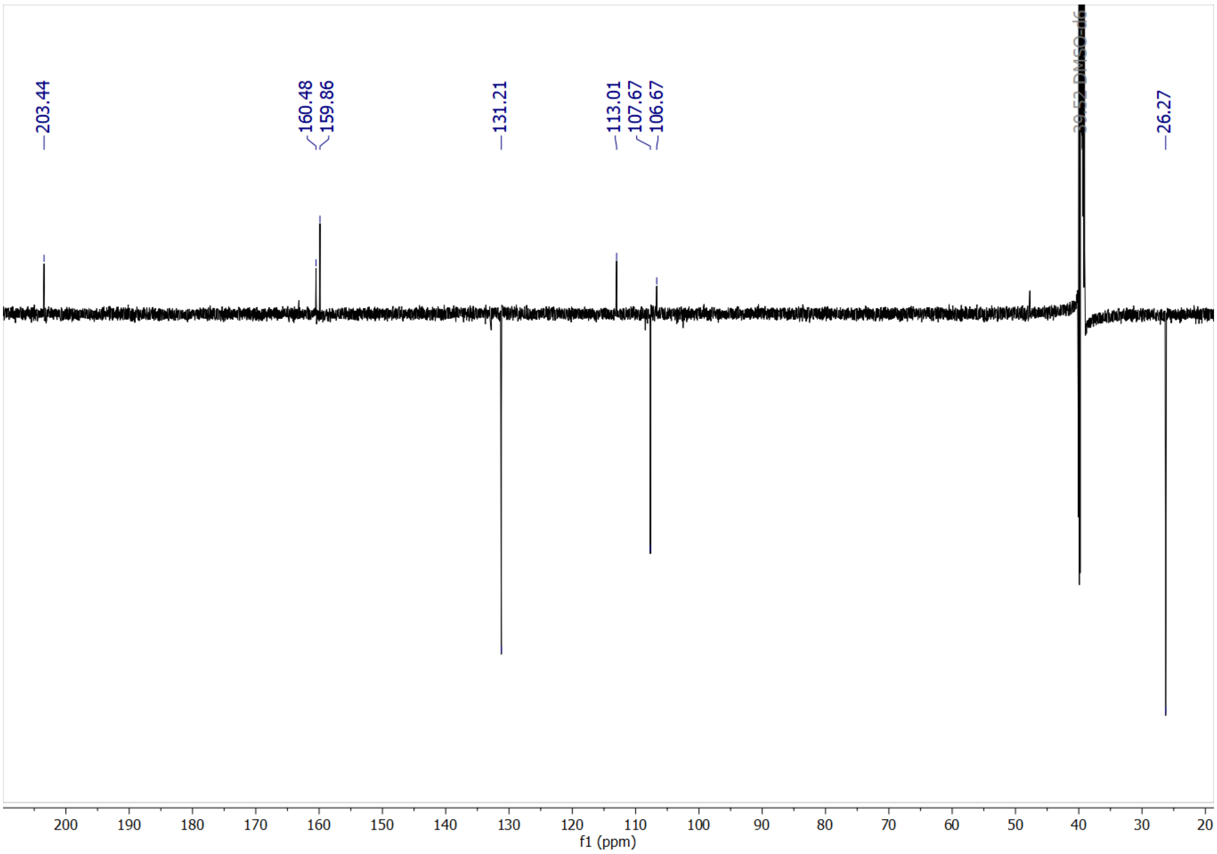

^{13}C DEPTQ NMR spectrum of **3-chloro-2,4-dihydroxyacetophenone** in DMSO-d_6 at 151 MHz.

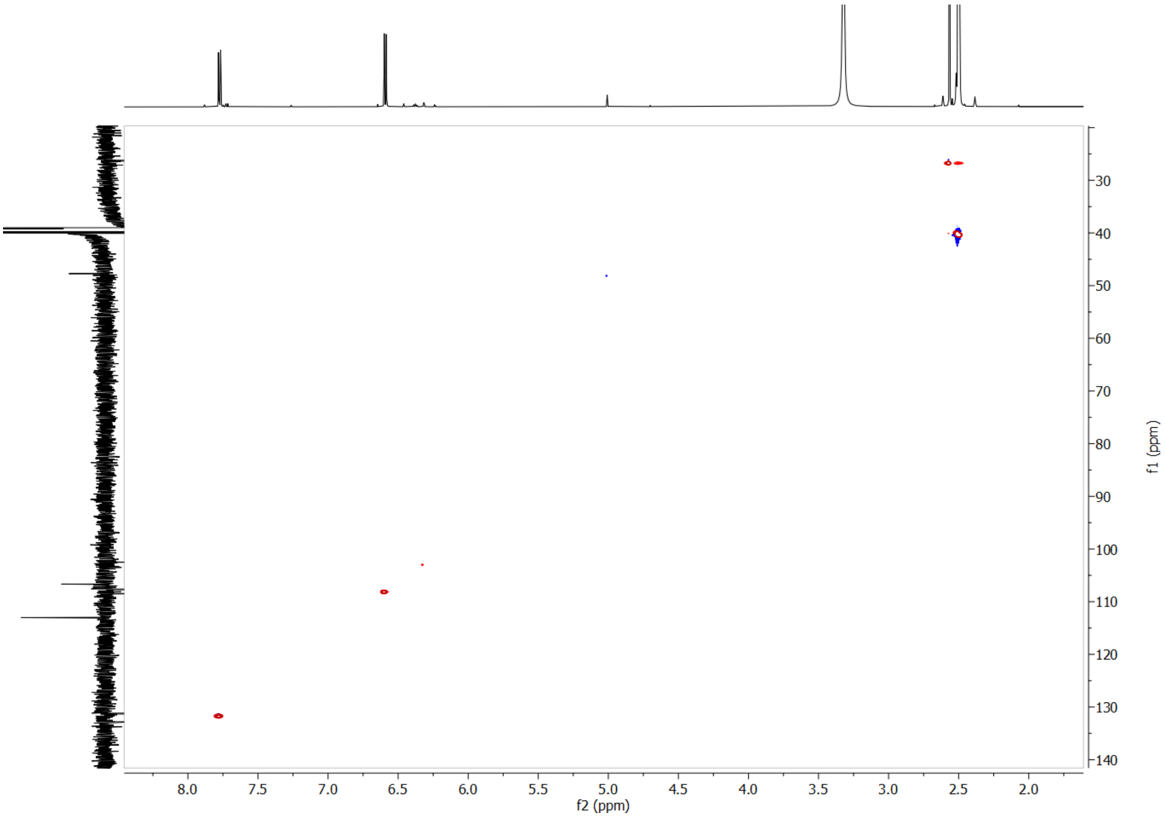

HSQC NMR spectrum of **3-chloro-2,4-dihydroxyacetophenone** in DMSO-d_6 at 600 MHz.

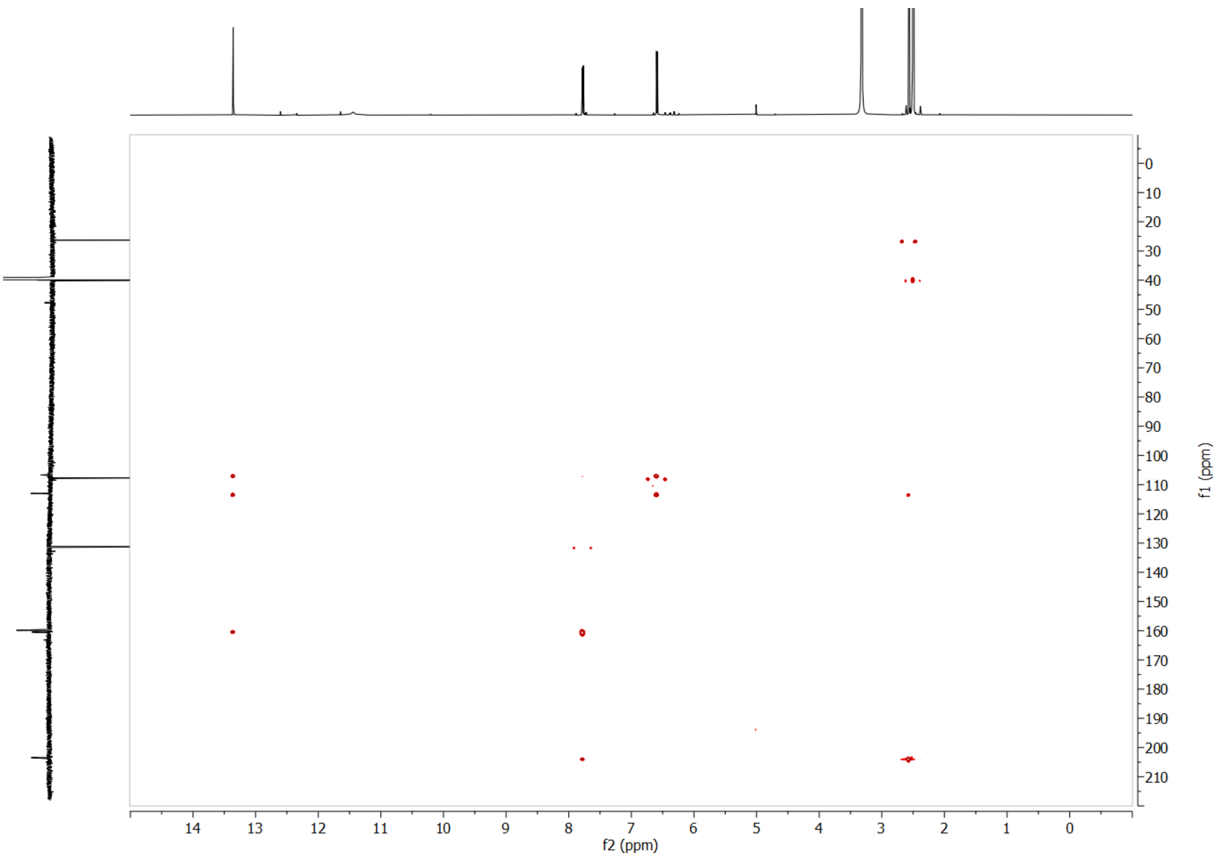

HMBC NMR spectrum of **3-chloro-2,4-dihydroxyacetophenone** in DMSO-d_6 at 600 MHz.

16. 5-chloro-2,4-dihydroxyacetophenone:

<u>Experimental:</u>

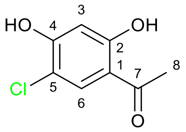

5-chloro-2,4-dihydroxyacetophenone (**16**) white solid; ^{1}H NMR (DMSO-d_6 600 MHz) δ 12.34 (1H, s), 11.43 (1H, s), 7.89 (1H, s), 6.47 (1H, s), 2.55 (3H, s); ^{13}C NMR (DMSO-d_6, 151 MHz) δ 202.1, 162.1, 159.9, 132.5, 113.7, 111.3, 103.5, 27.0; HRESIMS *m/z* 187.0155 $[M+H]^+$ (calcd for $C_8H_8ClO_3^+$ 187.0156), *m/z* 185.0009 $[M-H]^-$ (calcd for $C_8H_6ClO_3^-$ 185.0011).

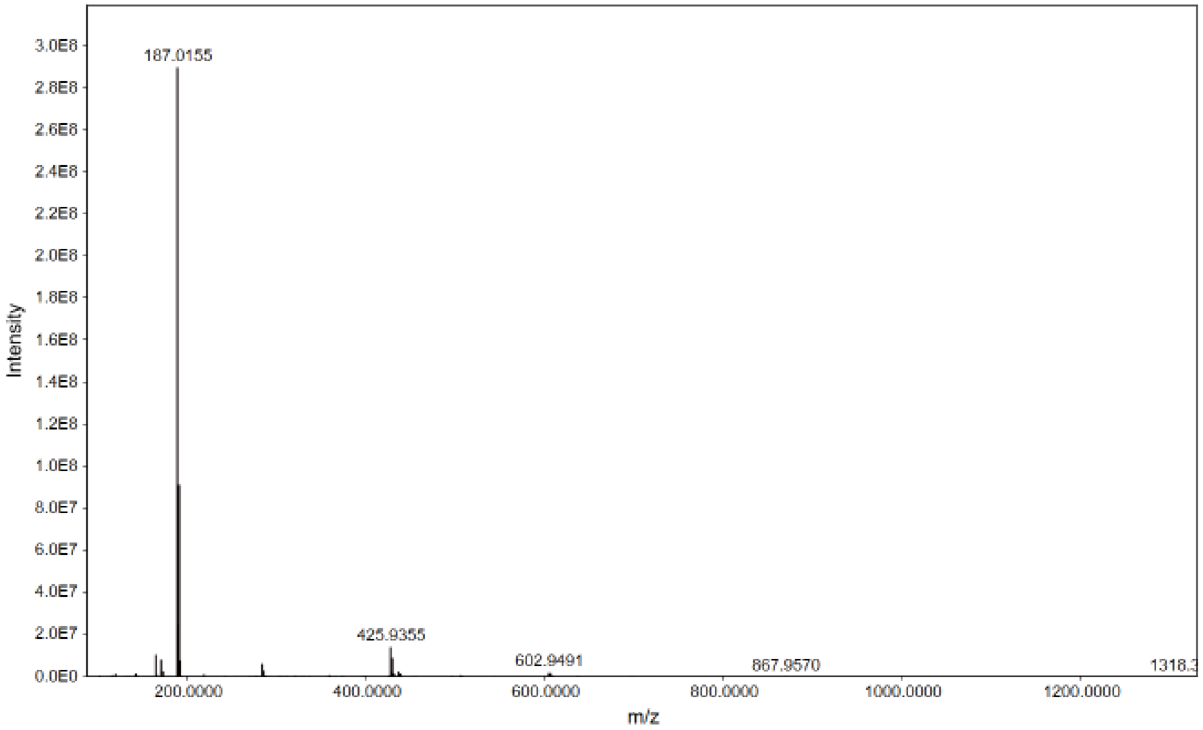

HRESIMS+ spectrum of **5-chloro-2,4-dihydroxyacetophenone** in MeOH.

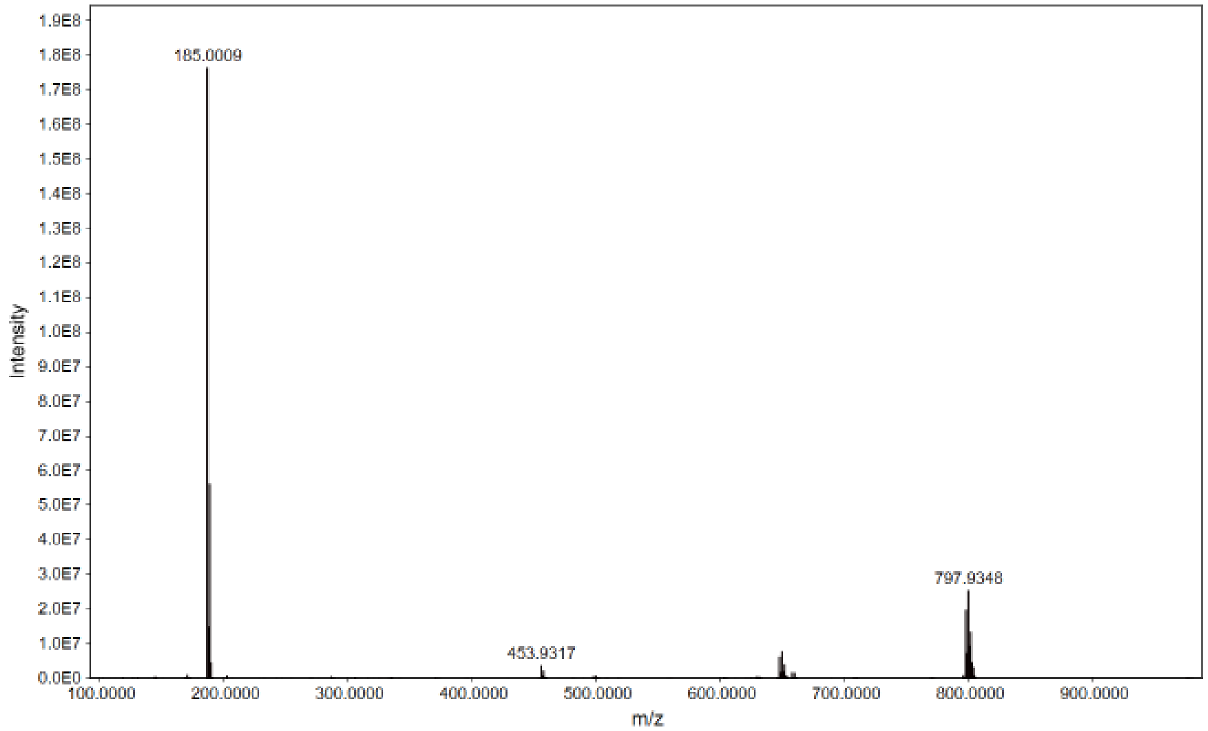

HRESIMS+ spectrum of **5-chloro-2,4-dihydroxyacetophenone** in MeOH.

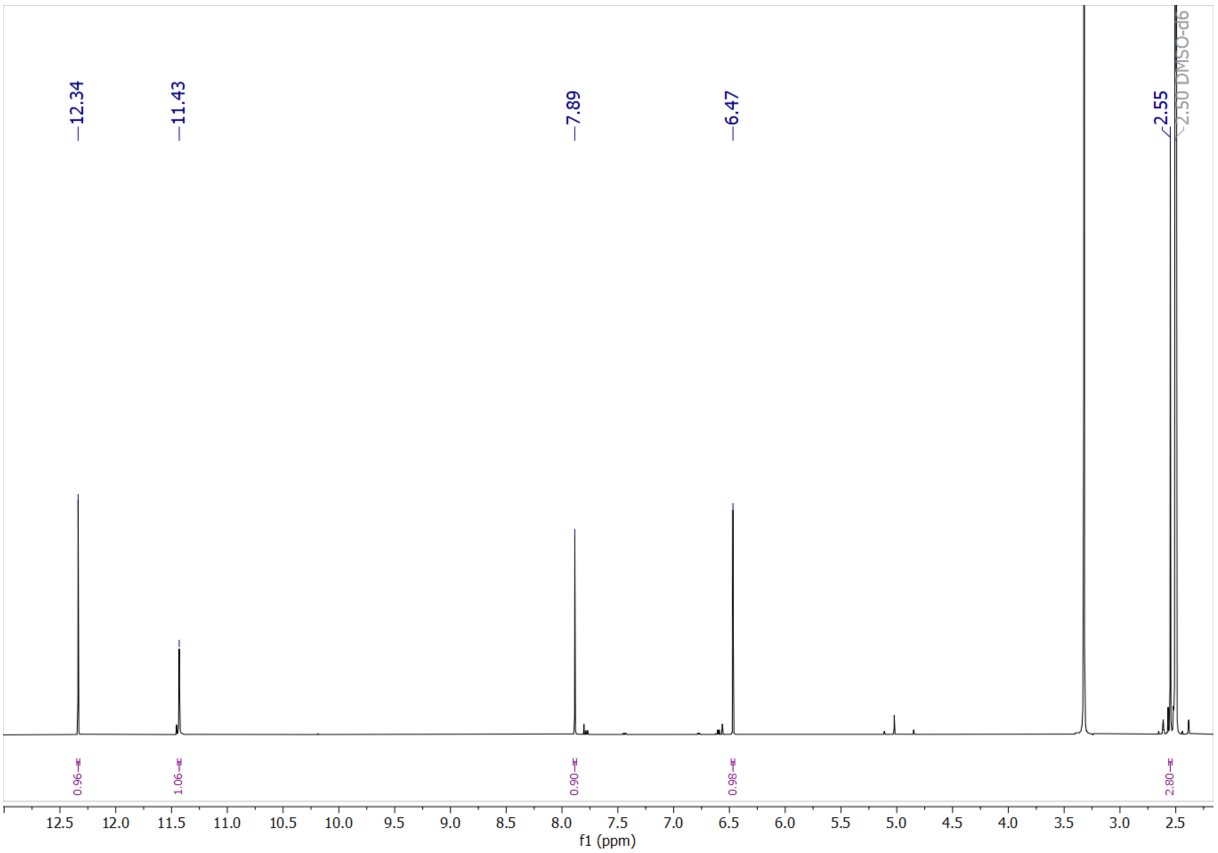

^{1}H NMR spectrum of **5-chloro-2,4-dihydroxyacetophenone** in DMSO-d_6 at 600 MHz.

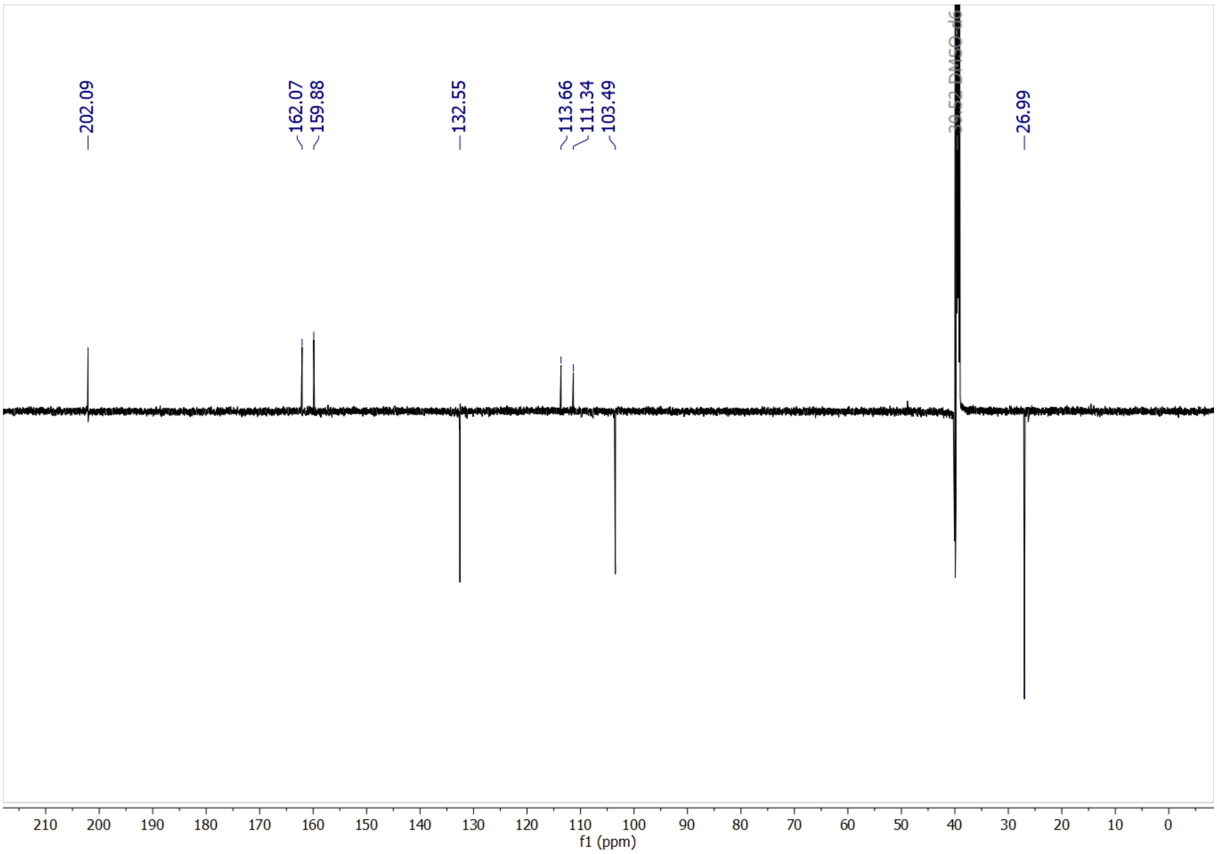

^{13}C DEPTQ NMR spectrum of **5-chloro-2,4-dihydroxyacetophenone** in DMSO-d_6 at 151 MHz.

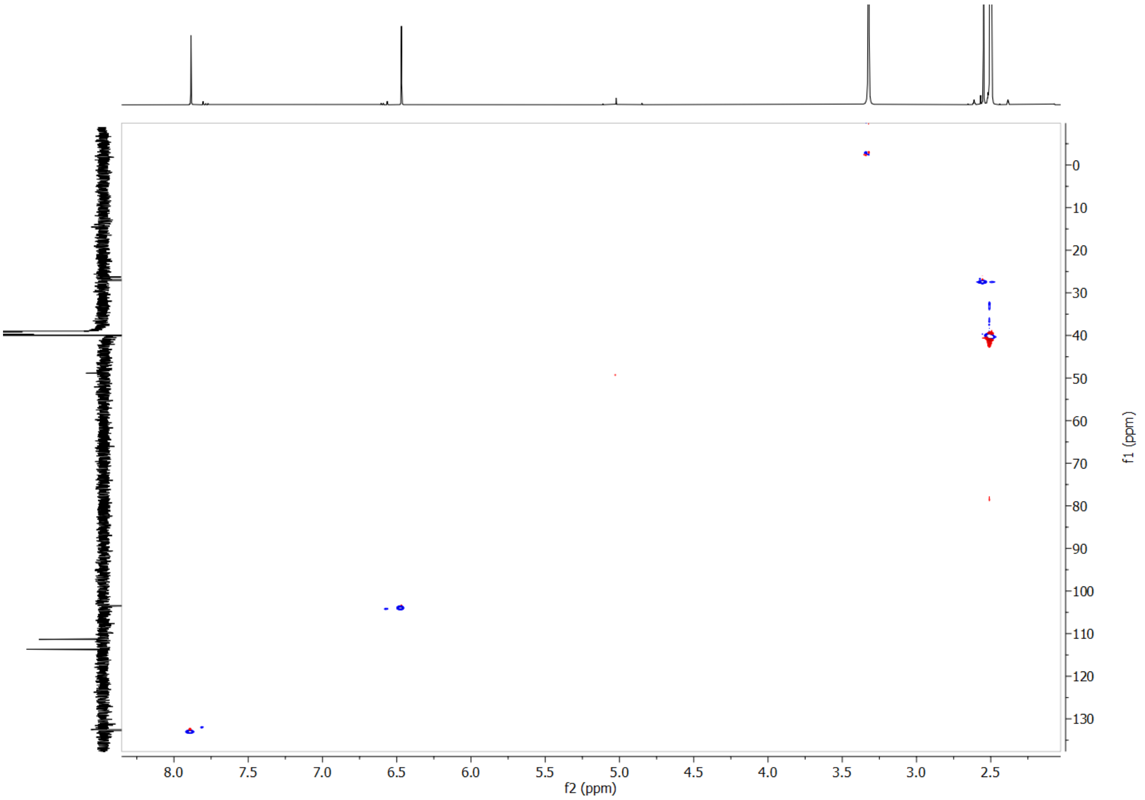

HSQC NMR spectrum of **5-chloro-2,4-dihydroxyacetophenone** in DMSO-d_6 at 600 MHz.

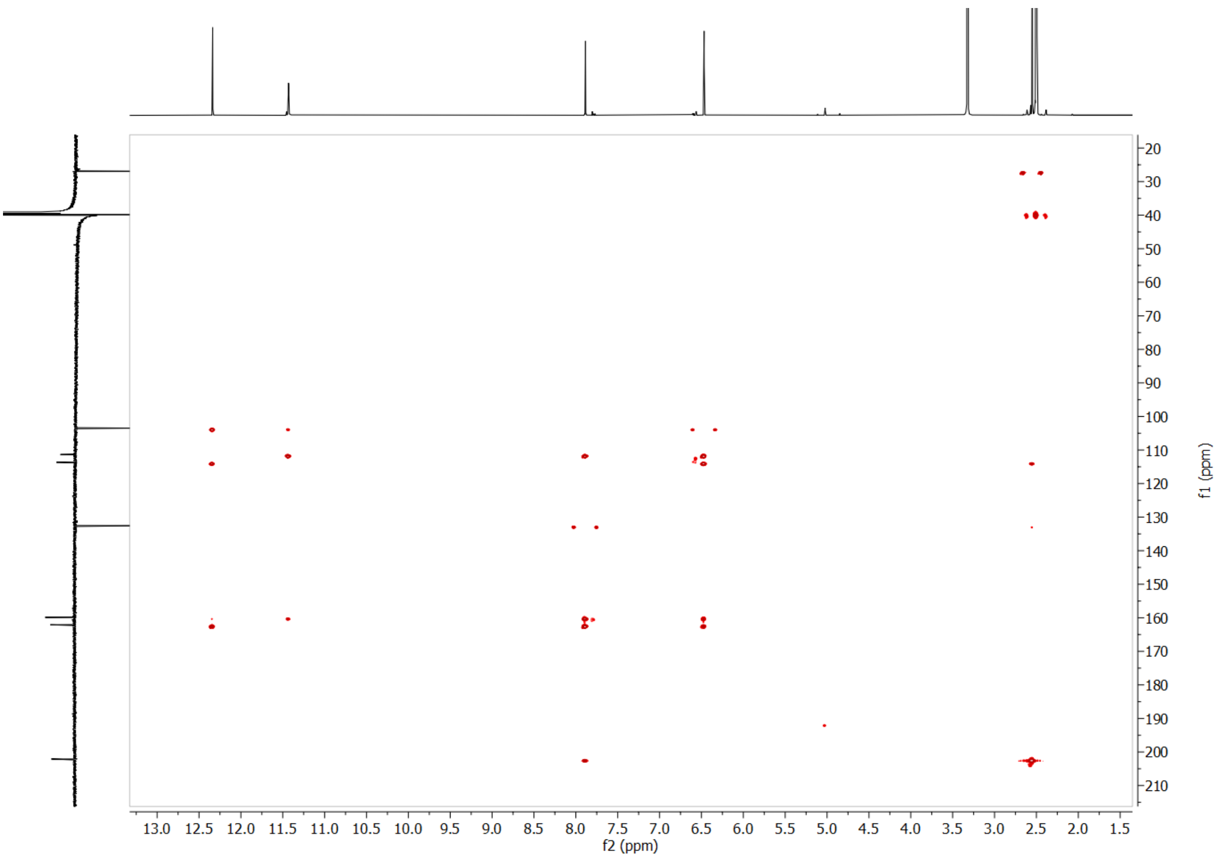

HMBC NMR spectrum of **5-chloro-2,4-dihydroxyacetophenone** in DMSO-d_6 at 600 MHz.

17. 3,5-di-chloro-2,4-dihydroxyacetophenone:

Experimental:

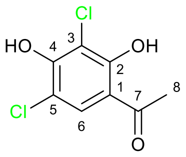

3,5-di-chloro-2,4-dihydroxyacetophenone (**17**) white solid; ^{1}H NMR (DMSO-d_6, 600 MHz) δ 13.18 (1H, s), 7.99 (1H, s), 2.61 (3H, s); ^{13}C NMR (DMSO-d_6, 151 MHz) δ 203.3, 158.2, 155.9, 130.4, 113.2, 112.2, 108.9, 26.6; HRESIMS *m/z* 220.9767 [M+H]$^+$ (calcd for $C_8H_7Cl_2O_3^+$ 220.9767), *m/z* 218.9619 [M-H]$^-$ (calcd for $C_8H_5Cl_2O_3^-$ 218.9621).

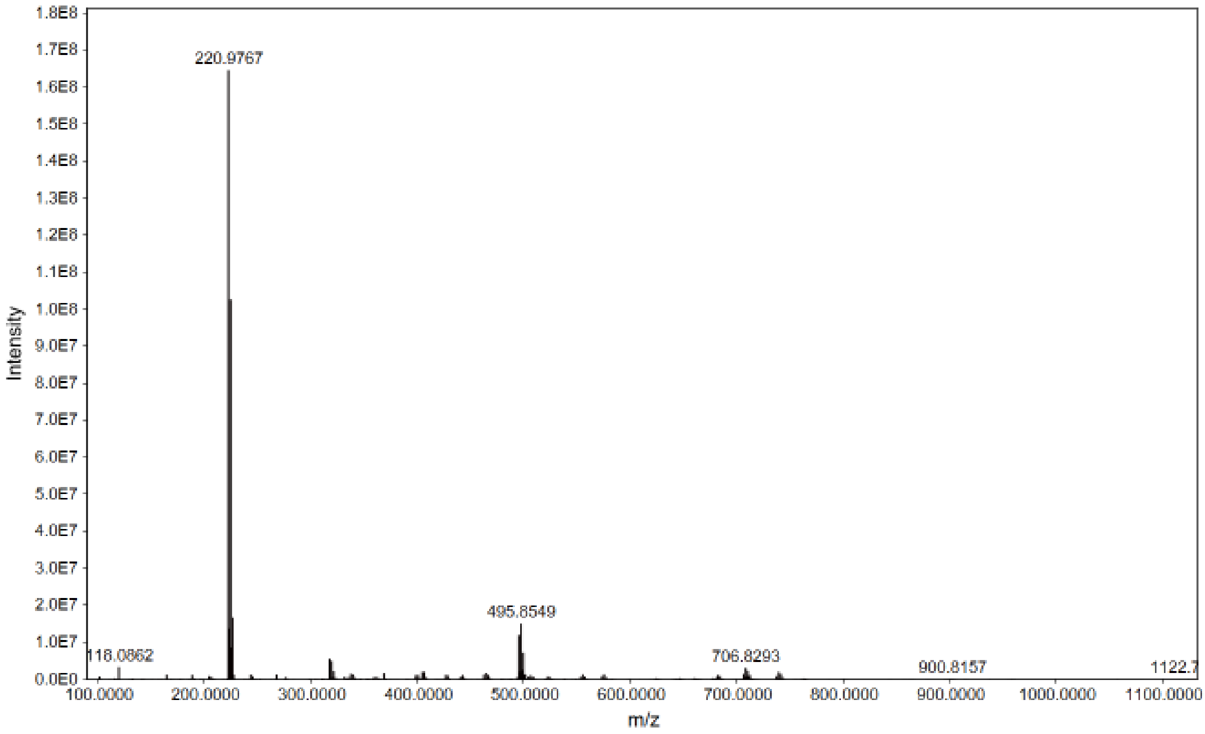

HRESIMS+ spectrum of **3,5-di-chloro-2,4-dihydroxyacetophenone** in MeOH.

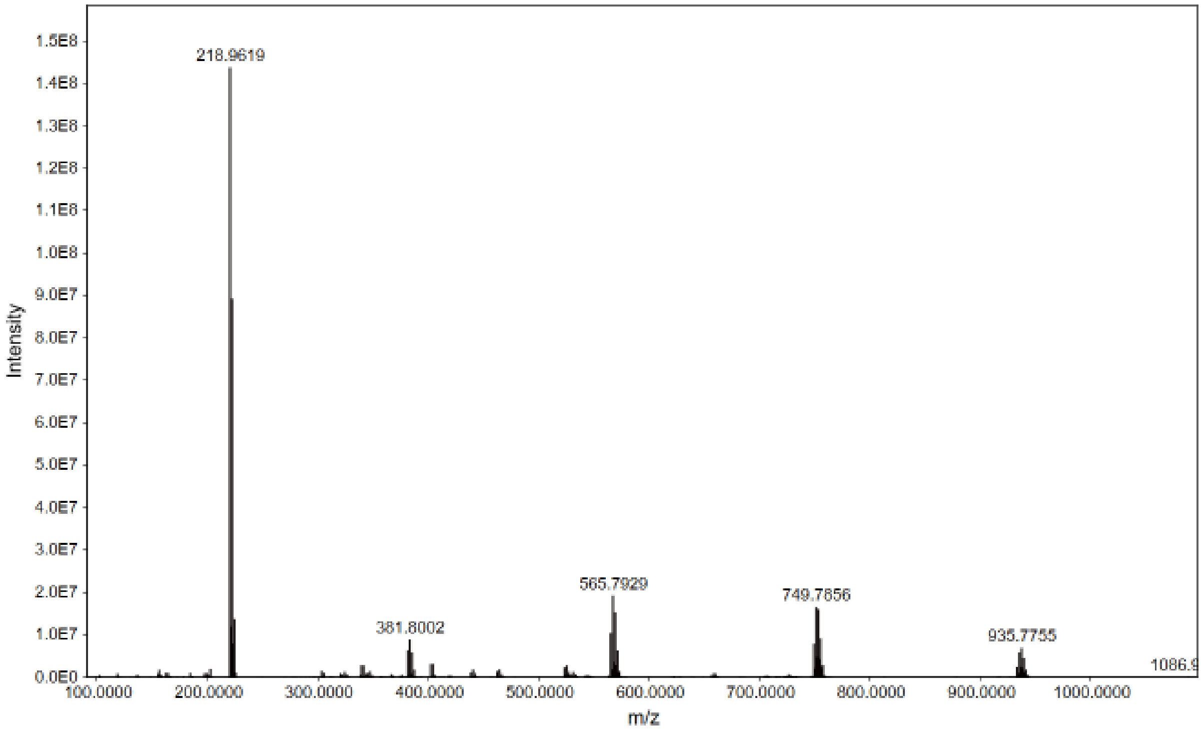

HRESIMS- spectrum of **3,5-di-chloro-2,4-dihydroxyacetophenone** in MeOH.

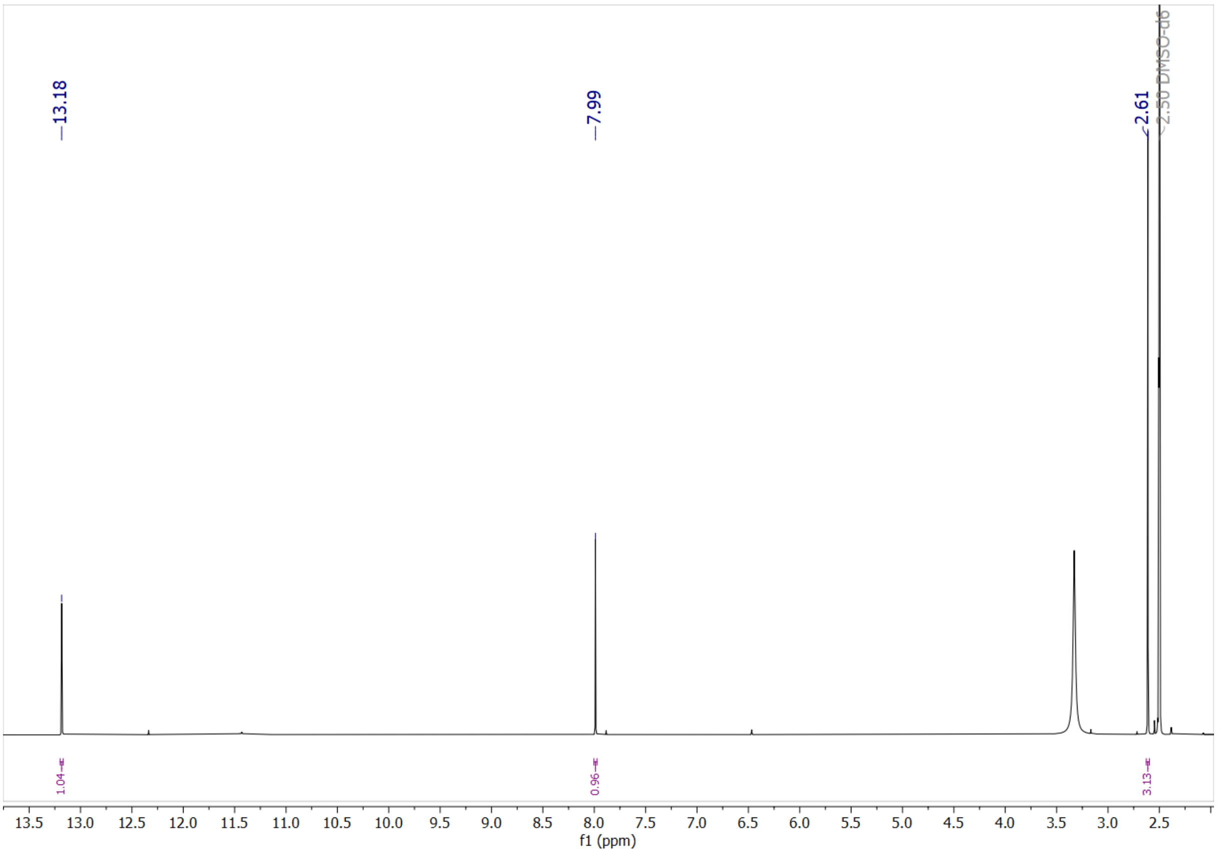

^{1}H NMR spectrum of **3,5-di-chloro-2,4-dihydroxyacetophenone** in DMSO-d_6 at 600 MHz.

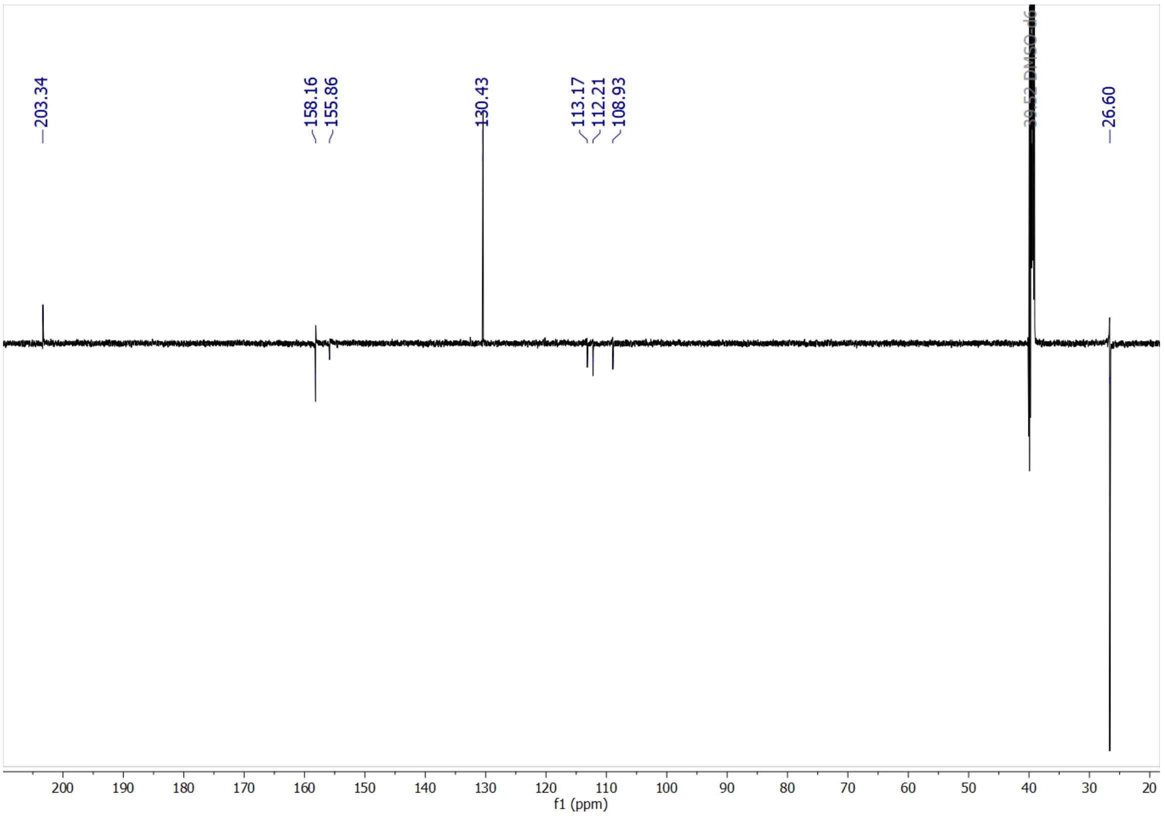

^{13}C DEPTQ NMR spectrum of **3,5-di-chloro-2,4-dihydroxyacetophenone** in DMSO-d_6 at 151 MHz.

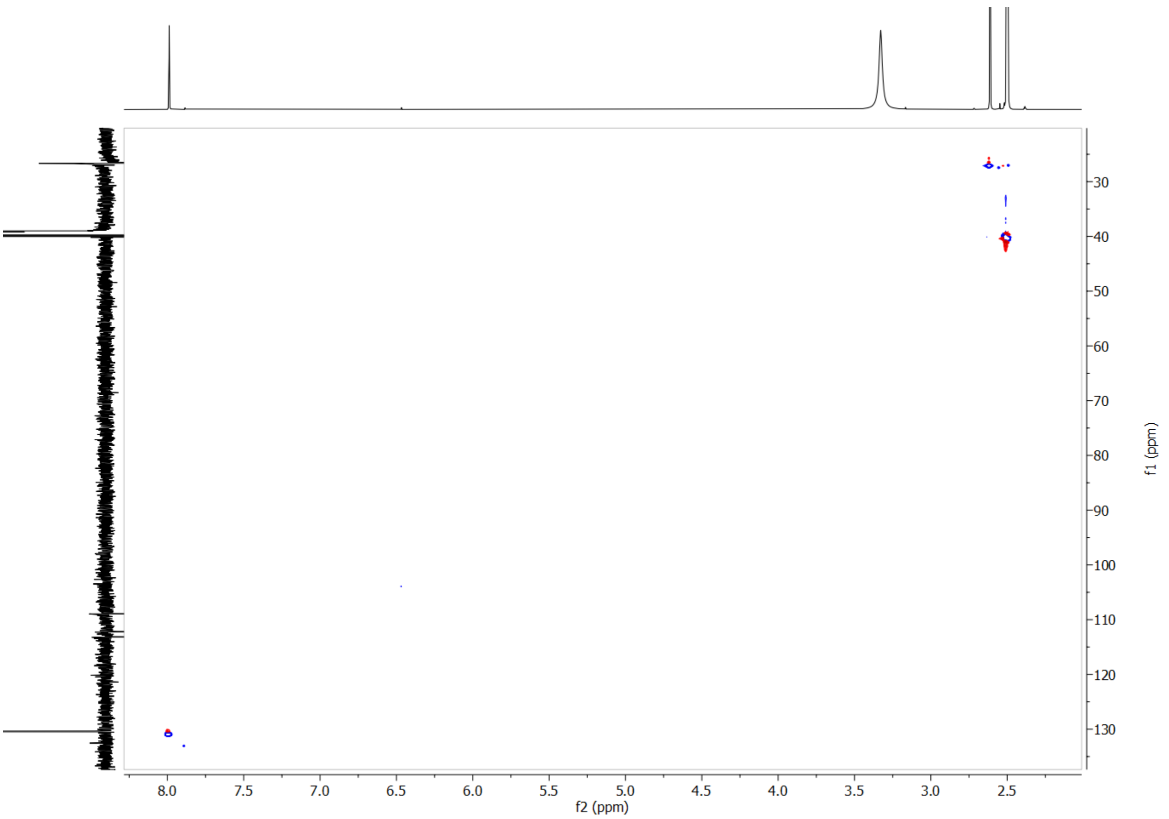

HSQC NMR spectrum of **3,5-di-chloro-2,4-dihydroxyacetophenone** in DMSO-d_6 at 600 MHz.

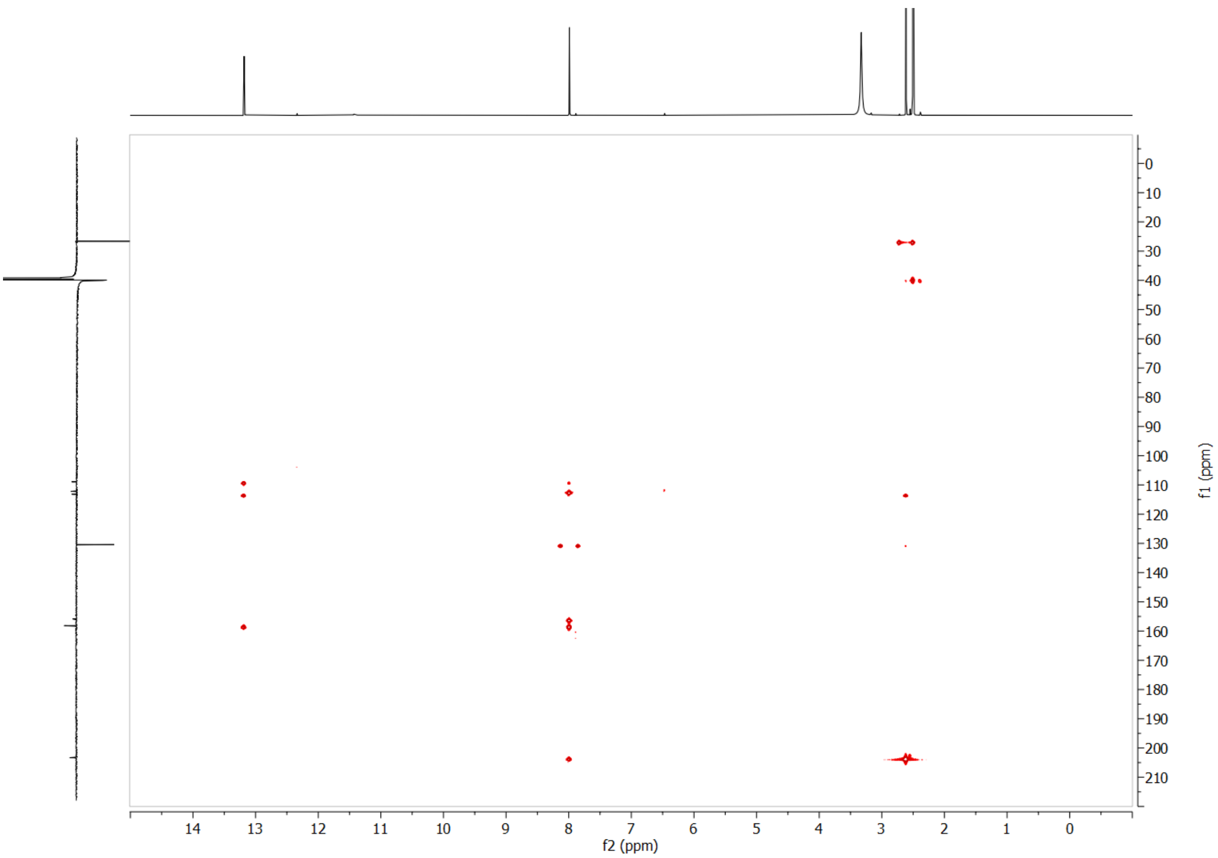

HMBC NMR spectrum of **3,5-di-chloro-2,4-dihydroxyacetophenone** in DMSO-d_6 at 600 MHz.

18. 3-bromo-2,4-dihydroxyacetophenone:

Experimental:

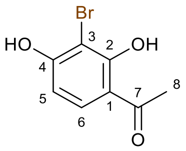

3-bromo-2,4-dihydroxyacetophenone (**18**) light brown solid; ^{1}H NMR (DMSO-d_6, 600 MHz) δ 13.50 (1H, s), 11.50 (1H, s), 7.81 (1H, d, J = 8.8 Hz), 6.59 (1H, d, J = 8.9 Hz), 2.57 (3H, s); ^{13}C NMR (DMSO-d_6, 151 MHz) δ 203.3, 161.6, 160.9, 132.1, 113.1, 107.6, 97.2, 26.2; HRESIMS *m/z* 230.9650 $[M+H]^+$ (calcd for $C_8H_8BrO_3^+$ 230.9651), *m/z* 228.9505 $[M-H]^-$ (calcd for $C_8H_6BrO_3^-$ 228.9506).

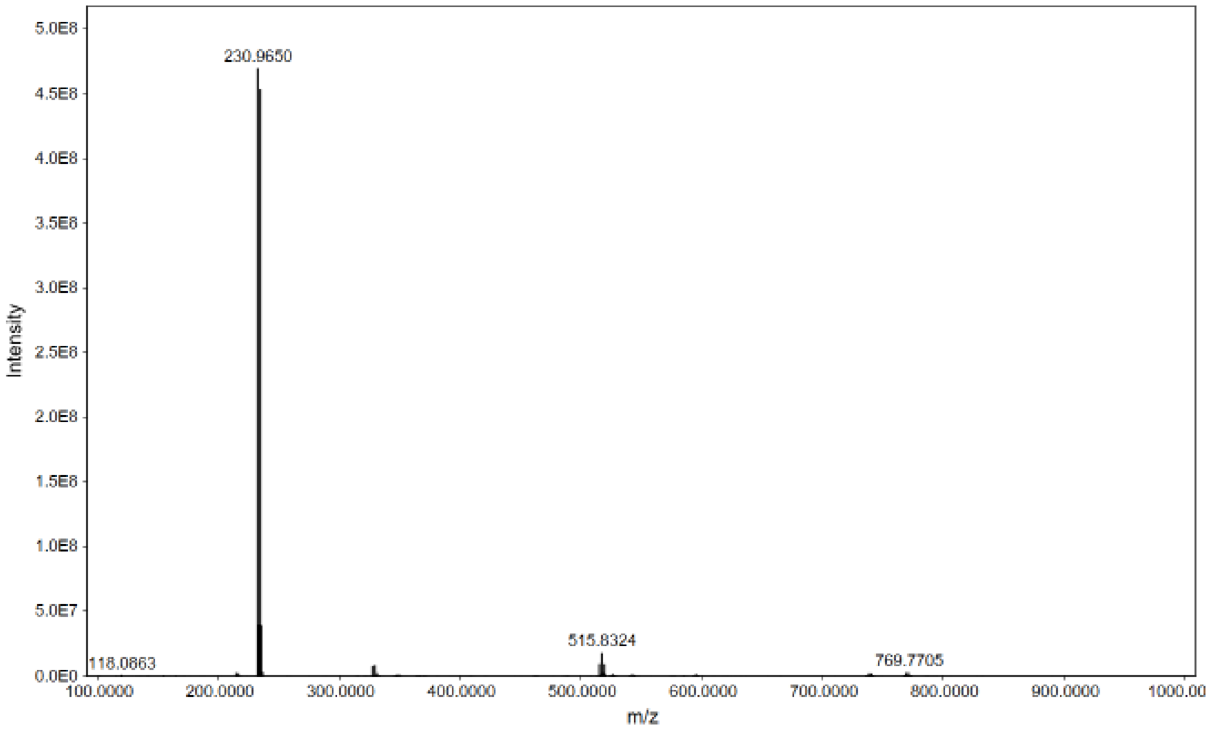

HRESIMS+ spectrum of **3-bromo-2,4-dihydroxyacetophenone** in MeOH.

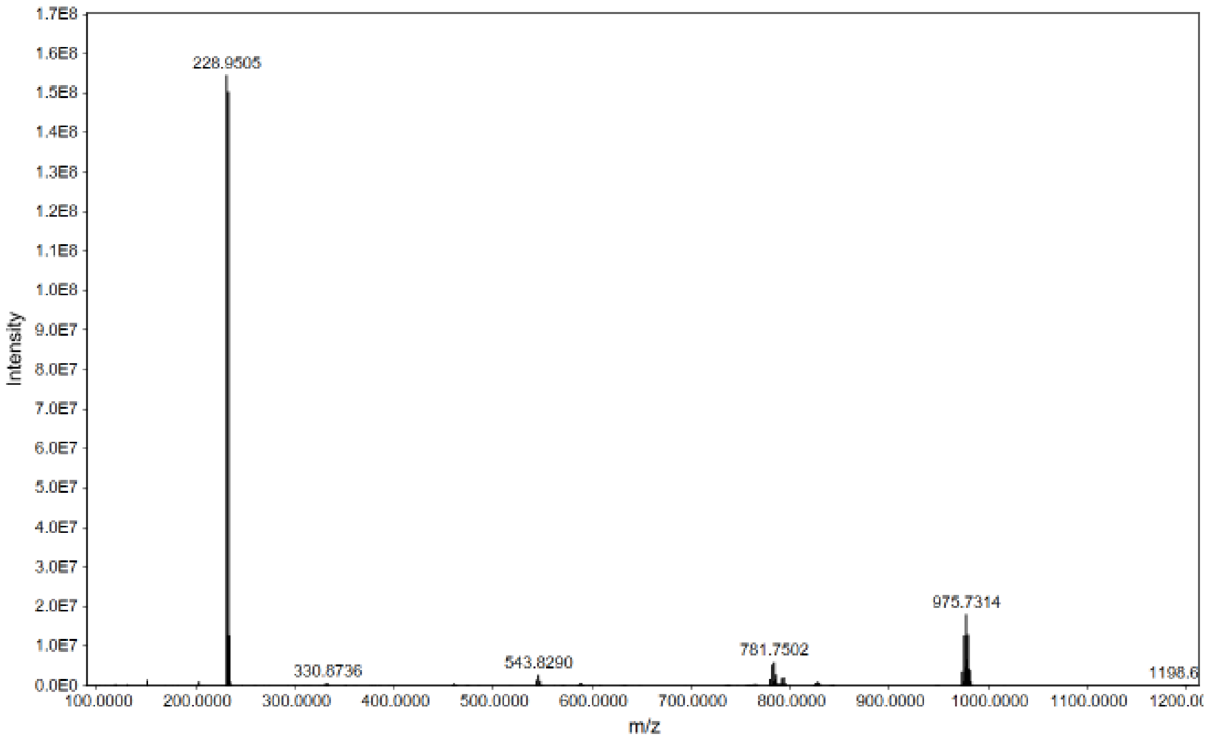

HRESIMS- spectrum of **3-bromo-2,4-dihydroxyacetophenone** in MeOH.

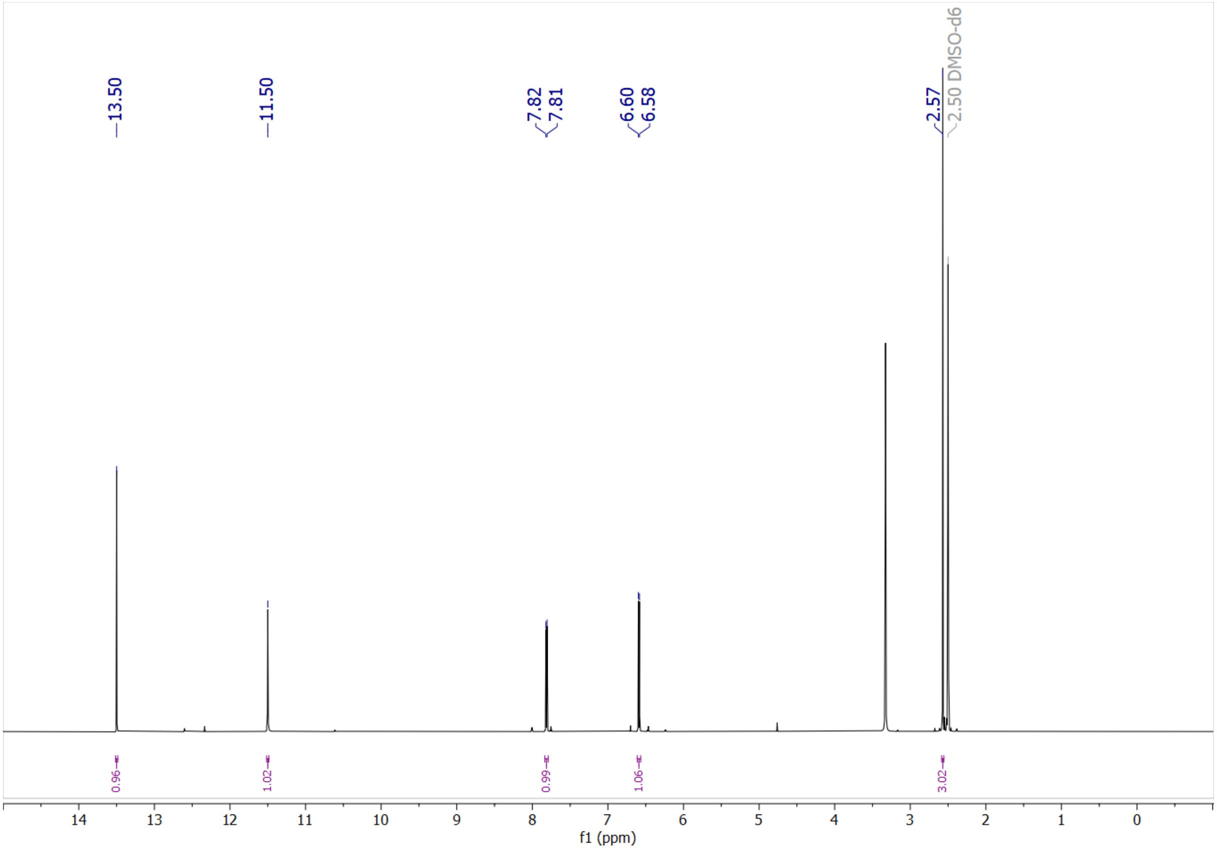

^{1}H NMR spectrum of **3-bromo-2,4-dihydroxyacetophenone** in DMSO-d_6 at 600 MHz.

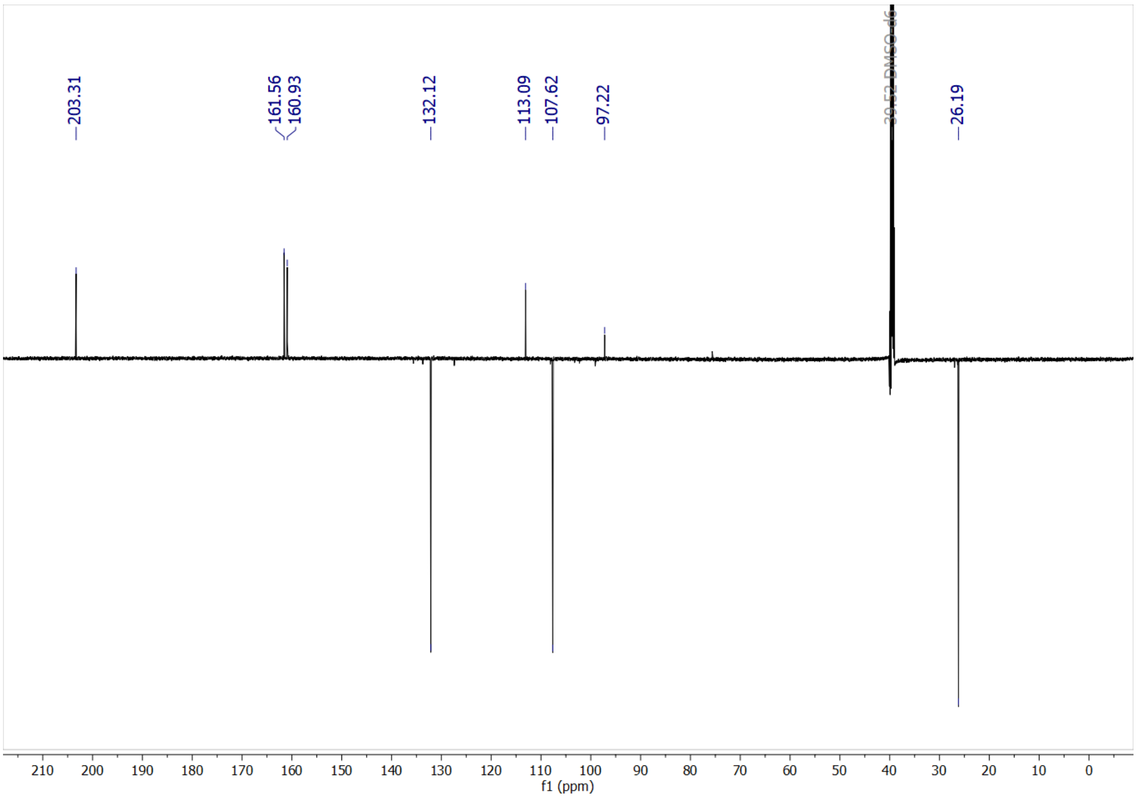

^{13}C DEPTQ NMR spectrum of **3-bromo-2,4-dihydroxyacetophenone** in DMSO-d_6 at 151 MHz.

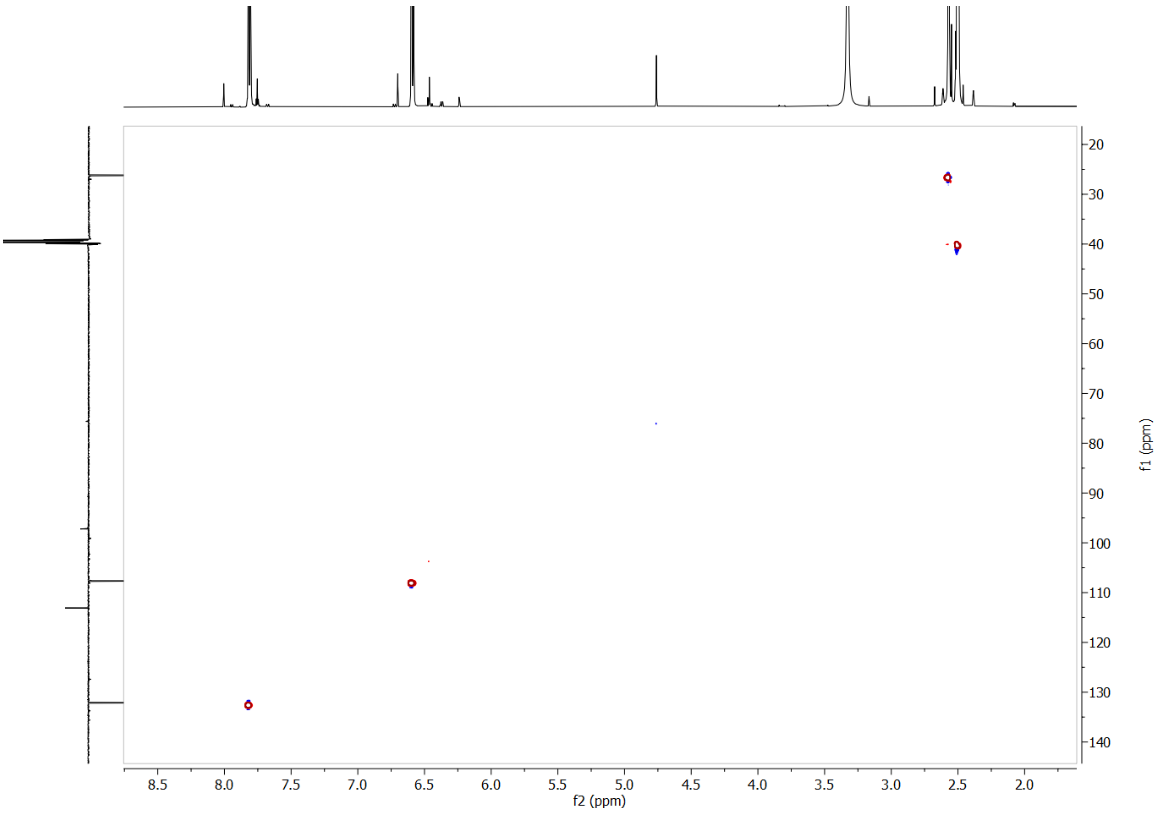

HSQC NMR spectrum of **3-bromo-2,4-dihydroxyacetophenone** in DMSO-d_6 at 600 MHz.

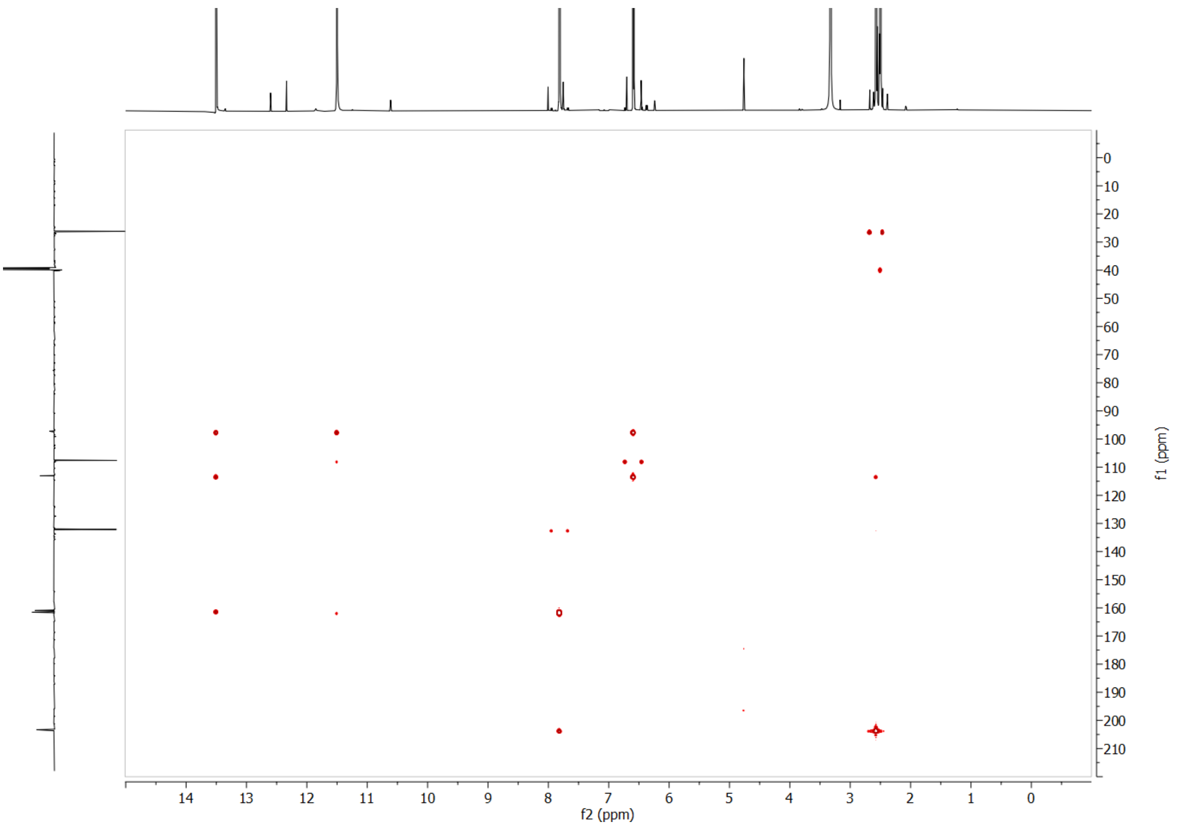

HMBC NMR spectrum of **3-bromo-2,4-dihydroxyacetophenone** in DMSO-d_6 at 600 MHz.

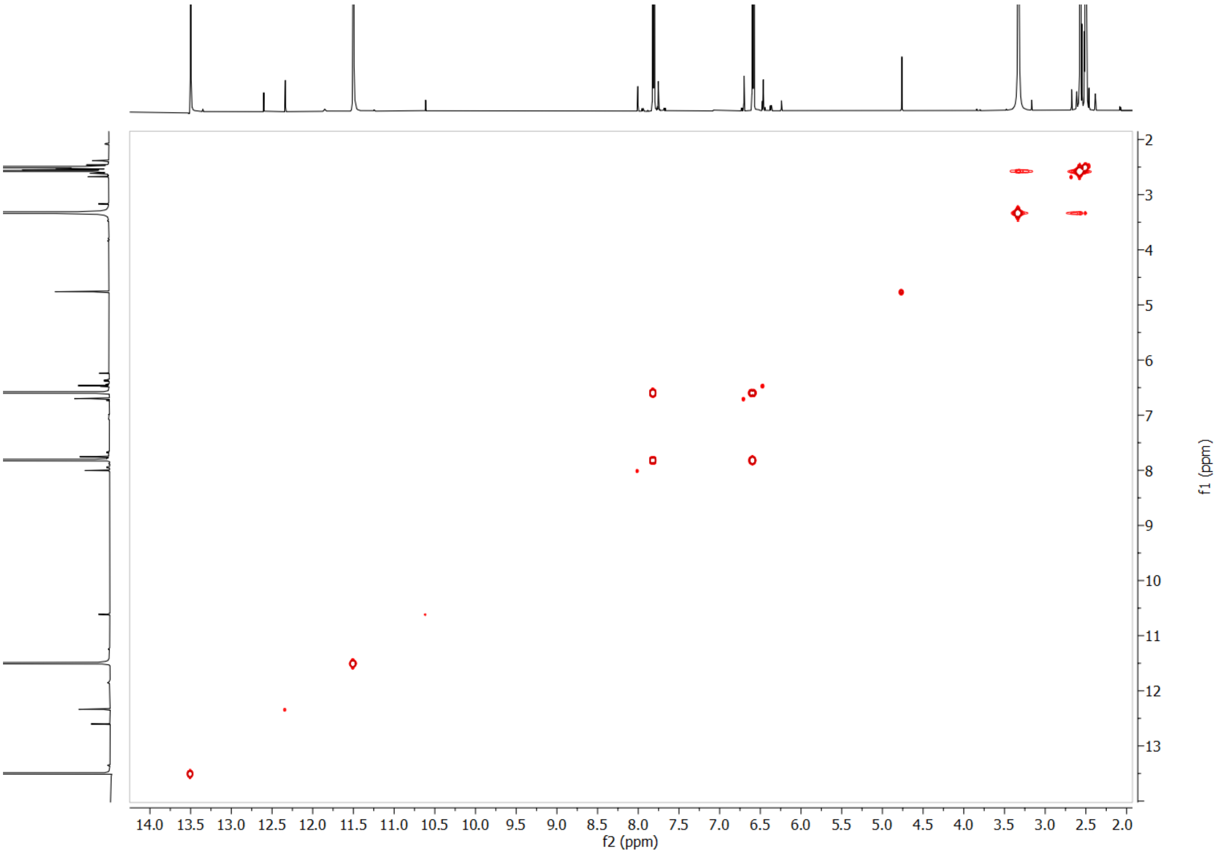

COSY NMR spectrum of **3-bromo-2,4-dihydroxyacetophenone** in DMSO-d_6 at 600 MHz.

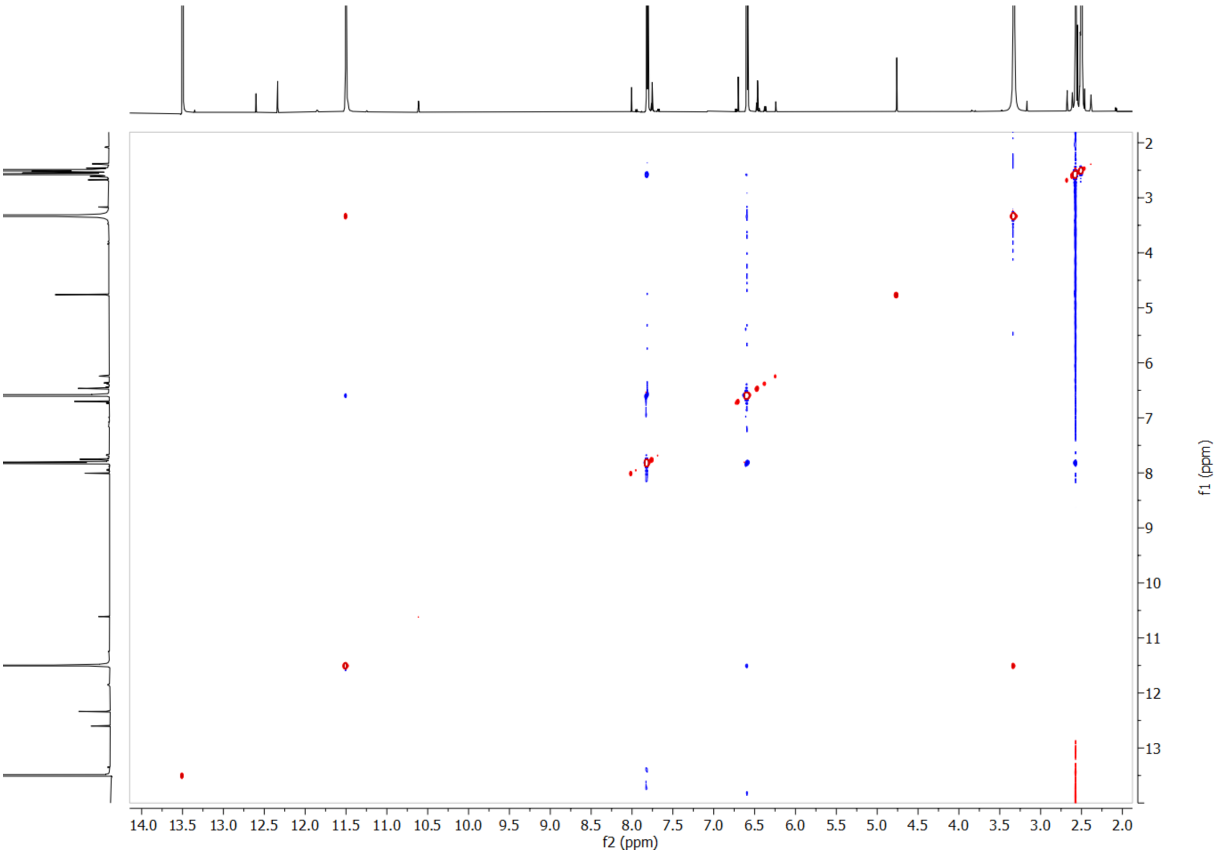

ROESY NMR spectrum of **3-bromo-2,4-dihydroxyacetophenone** in DMSO-d_6 at 600 MHz.

19. 5-bromo-2,4-dihydroxyacetophenone:

Experimental:

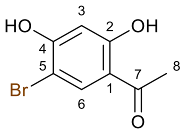

5-bromo-2,4-dihydroxyacetophenone (**19**) light yellow solid; ^{1}H NMR (DMSO-d_6, 600 MHz) δ 12.34 (1H, s), 11.48 (1H, s), 8.01 (1H, s), 6.47 (1H, s), 2.55 (3H, s); ^{13}C NMR (DMSO-d_6, 151 MHz) δ 202.0, 162.6, 160.8, 135.6, 114.4, 103.3, 100.0, 27.0; HRESIMS *m/z* 230.9650 $[M+H]^+$ (calcd for $C_8H_8BrO_3^+$ 230.9651), *m/z* 228.9505 $[M-H]^-$ (calcd for $C_8H_6BrO_3^-$ 228.9506).

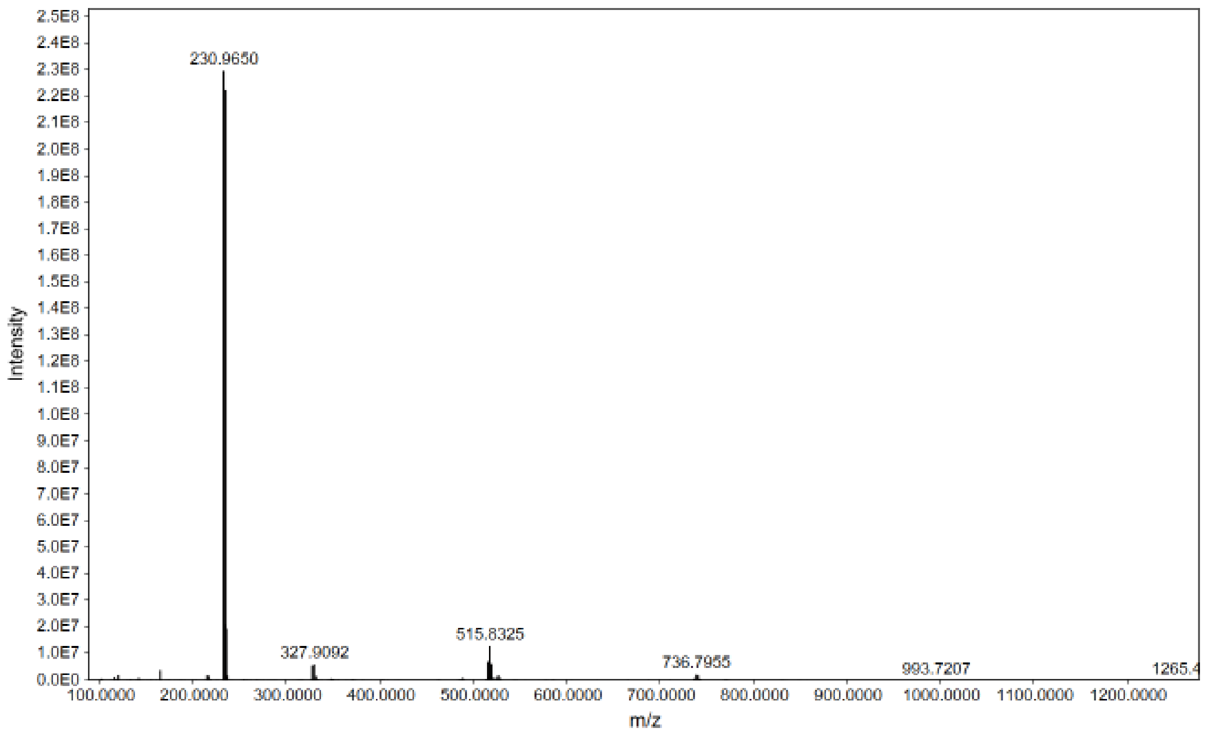

HRESIMS+ spectrum of **5-bromo-2,4-dihydroxyacetophenone** in MeOH.

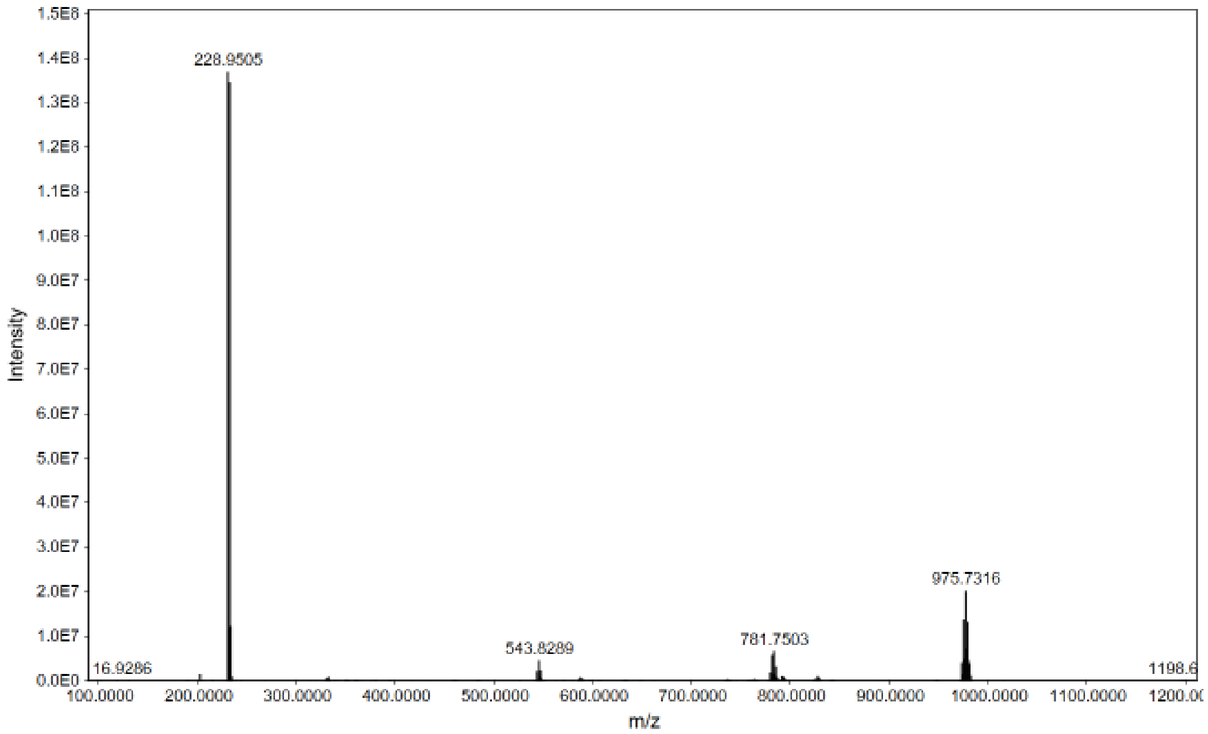

HRESIMS- spectrum of **5-bromo-2,4-dihydroxyacetophenone** in MeOH.

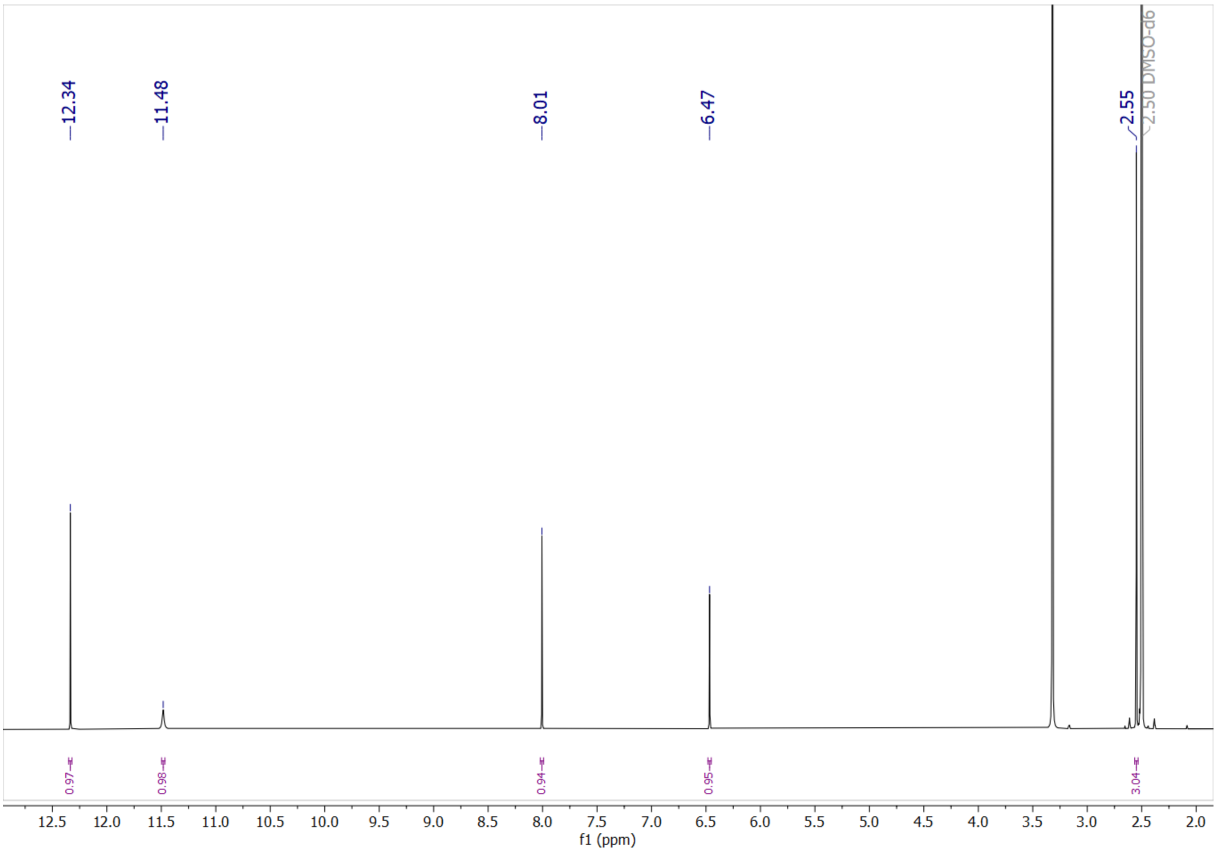

^{1}H NMR spectrum of **5-bromo-2,4-dihydroxyacetophenone** in DMSO-d_6 at 600 MHz.

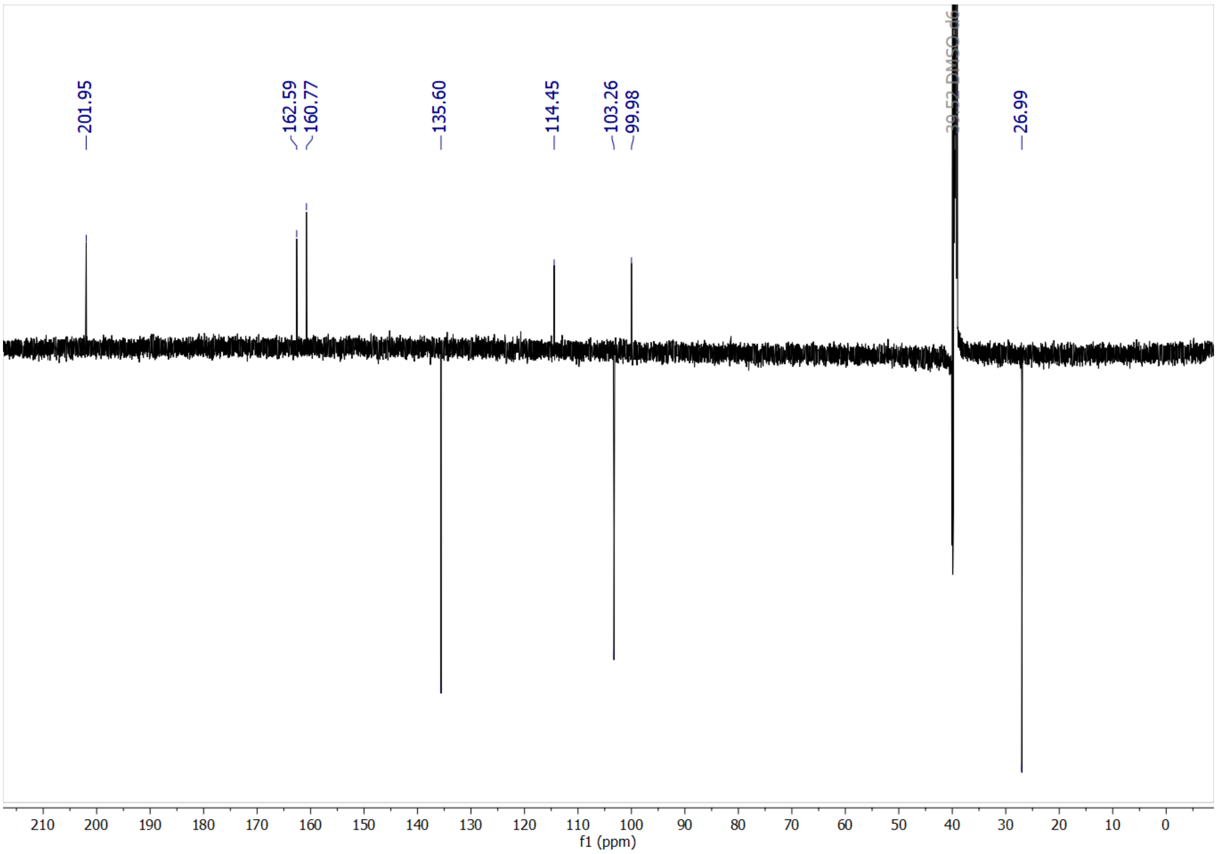

^{13}C DEPTQ NMR spectrum of **5-bromo-2,4-dihydroxyacetophenone** in DMSO-d_6 at 151 MHz.

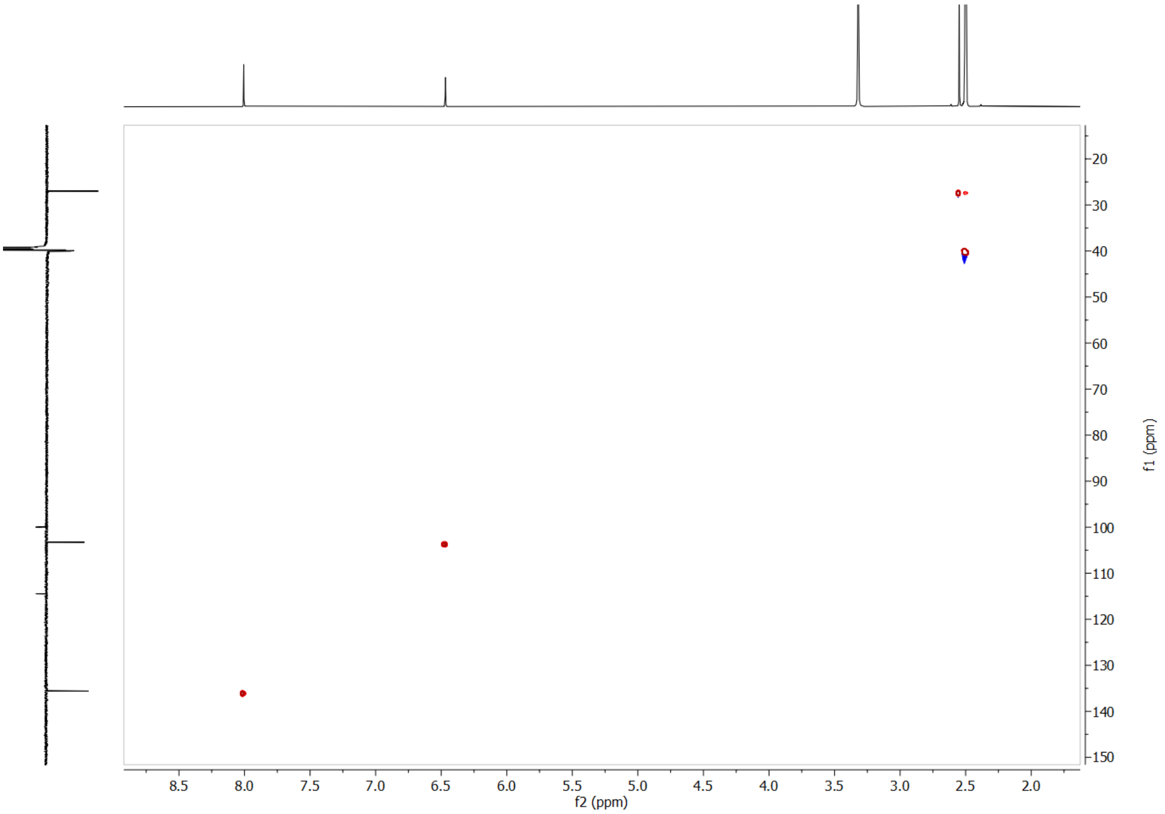

HSQC NMR spectrum of **5-bromo-2,4-dihydroxyacetophenone** in DMSO-d_6 at 600 MHz.

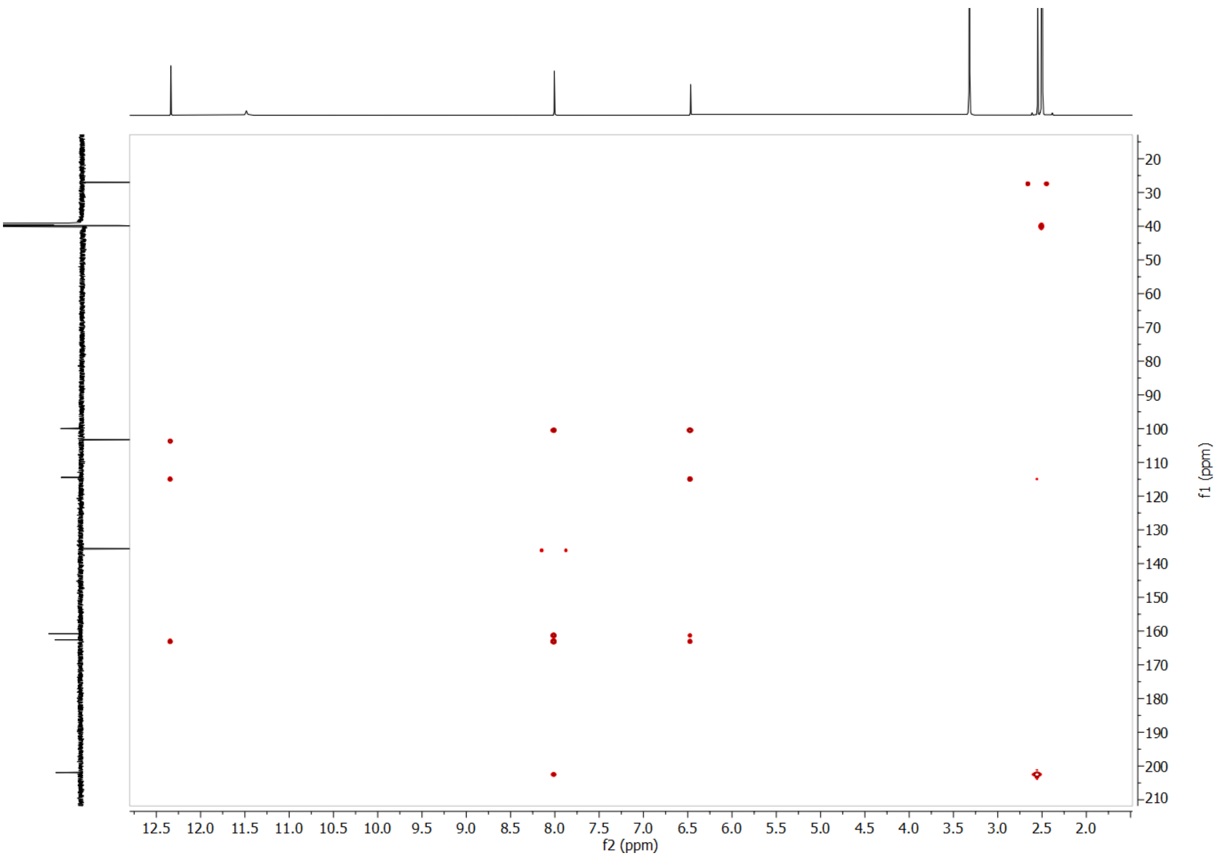

HMBC NMR spectrum of **5-bromo-2,4-dihydroxyacetophenone** in DMSO-d_6 at 600 MHz.

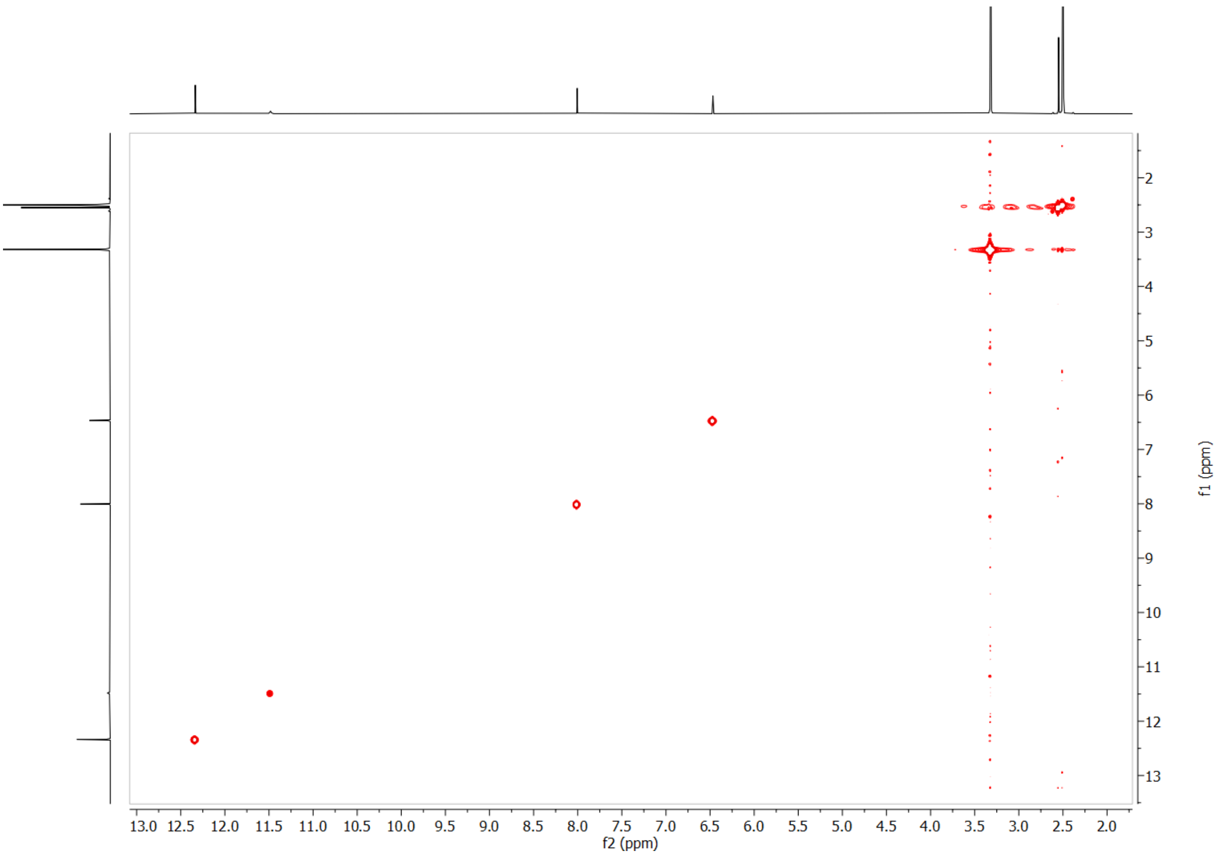

COSY NMR spectrum of **5-bromo-2,4-dihydroxyacetophenone** in DMSO-d_6 at 600 MHz.

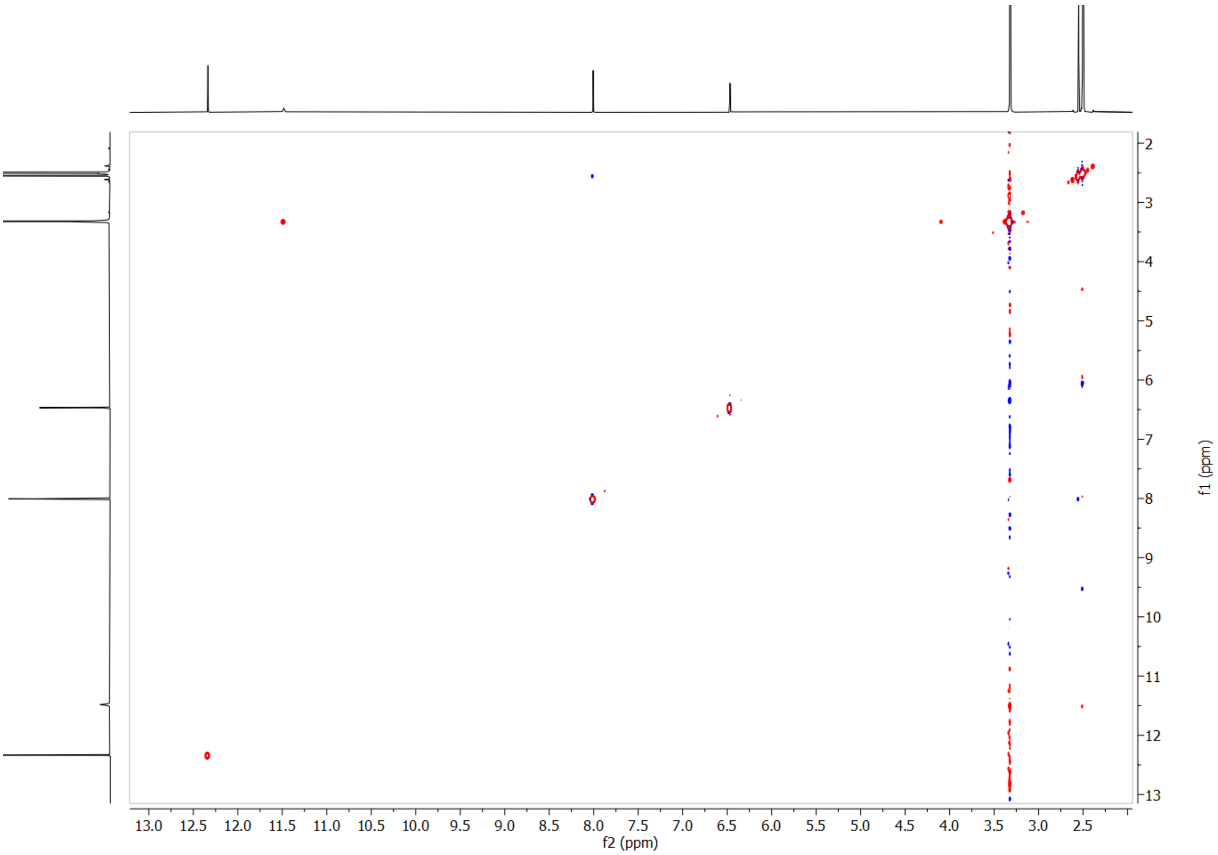

ROESY NMR spectrum of **5-bromo-2,4-dihydroxyacetophenone** in DMSO-d_6 at 600 MHz.

20. 3,5-di-bromo-2,4-dihydroxyacetophenone:

<u>Experimental:</u>

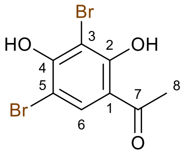

3,5-di-bromo-2,4-dihydroxyacetophenone (**20**) light yellow solid; ^{1}H NMR (DMSO-d_6, 600 MHz) δ 13.34 (1H, s), 12.34 (0H, s), 8.14 (1H, s), 2.62 (3H, s); ^{13}C NMR (DMSO-d_6, 151 MHz) δ 203.2, 159.7, 157.5, 134.3, 114.3, 100.8, 99.5, 26.5; HRESIMS *m/z* 308.8756 [M+H]$^+$ (calcd for $C_8H_7Br_2O_3^+$ 308.8756), *m/z* 306.8615 [M-H]$^-$ (calcd for $C_8H_5Br_2O_3^-$ 306.8611).

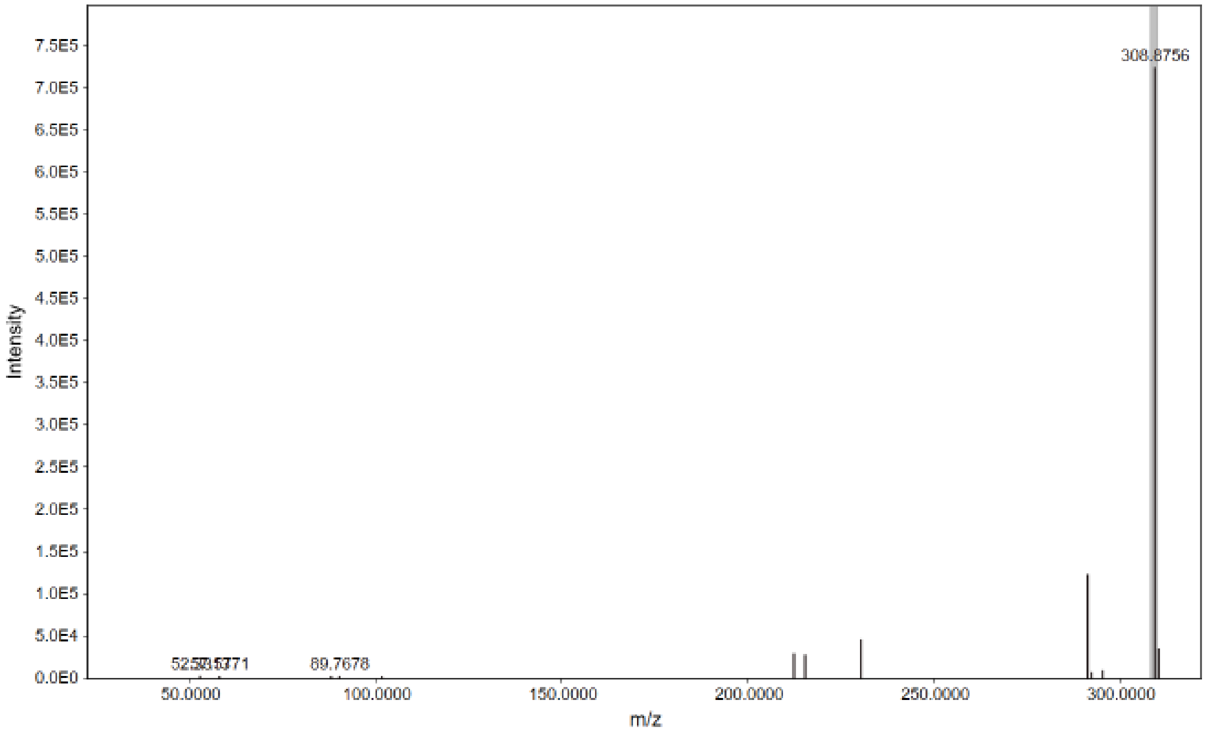

HRESIMS+ spectrum of **3,5-di-bromo-2,4-dihydroxyacetophenone** in MeOH.

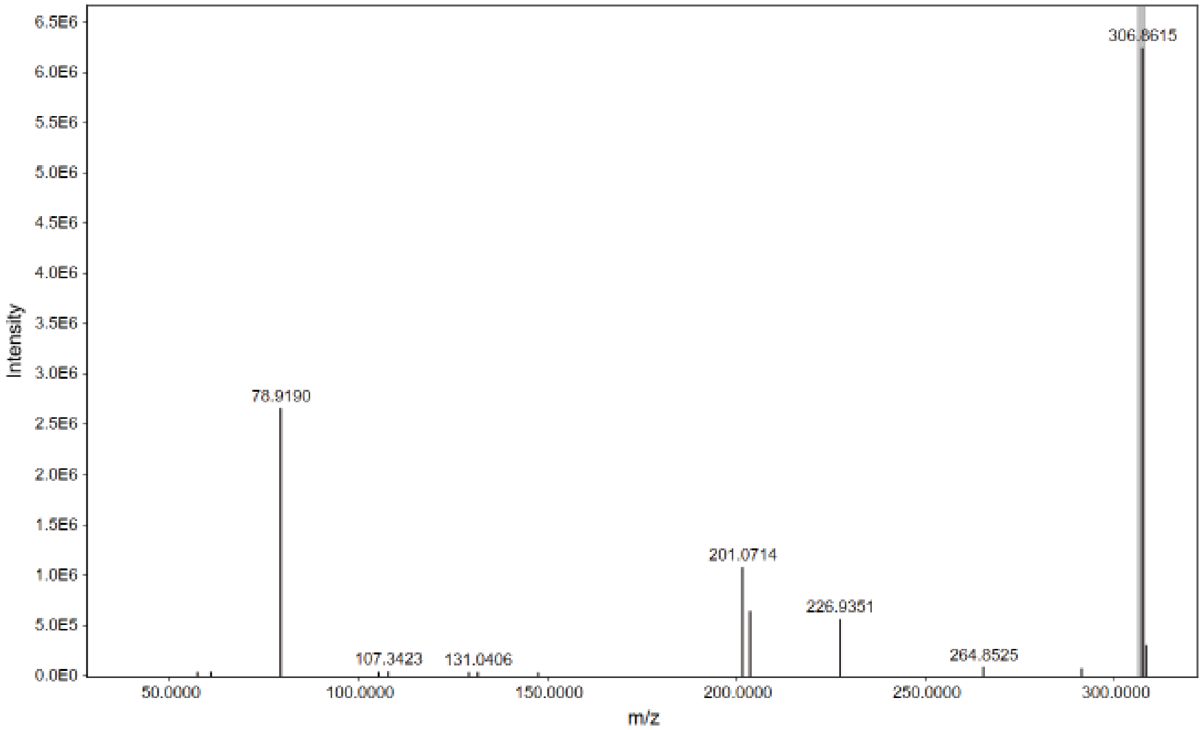

HRESIMS- spectrum of **3,5-di-bromo-2,4-dihydroxyacetophenone** in MeOH.

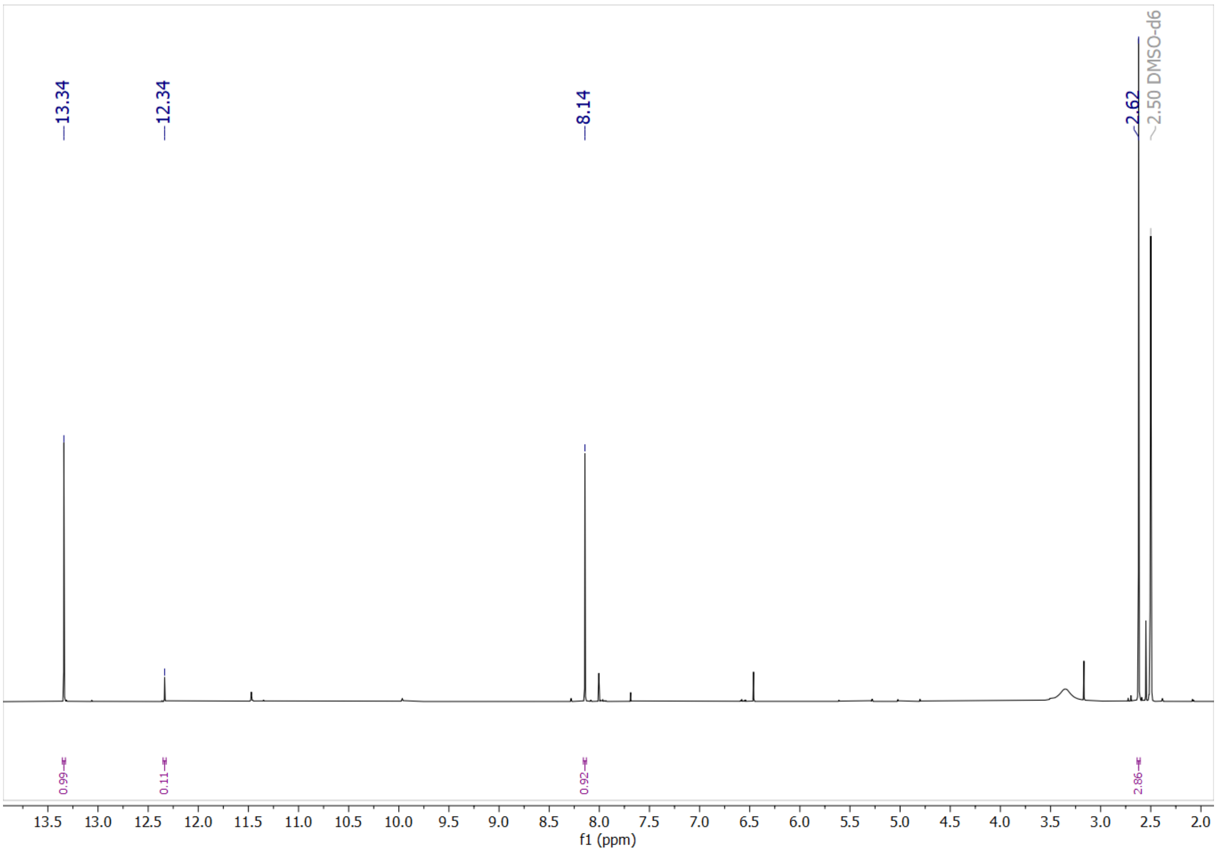

^{1}H NMR spectrum of **3,5-di-bromo-2,4-dihydroxyacetophenone** in DMSO-d_6 at 600 MHz.

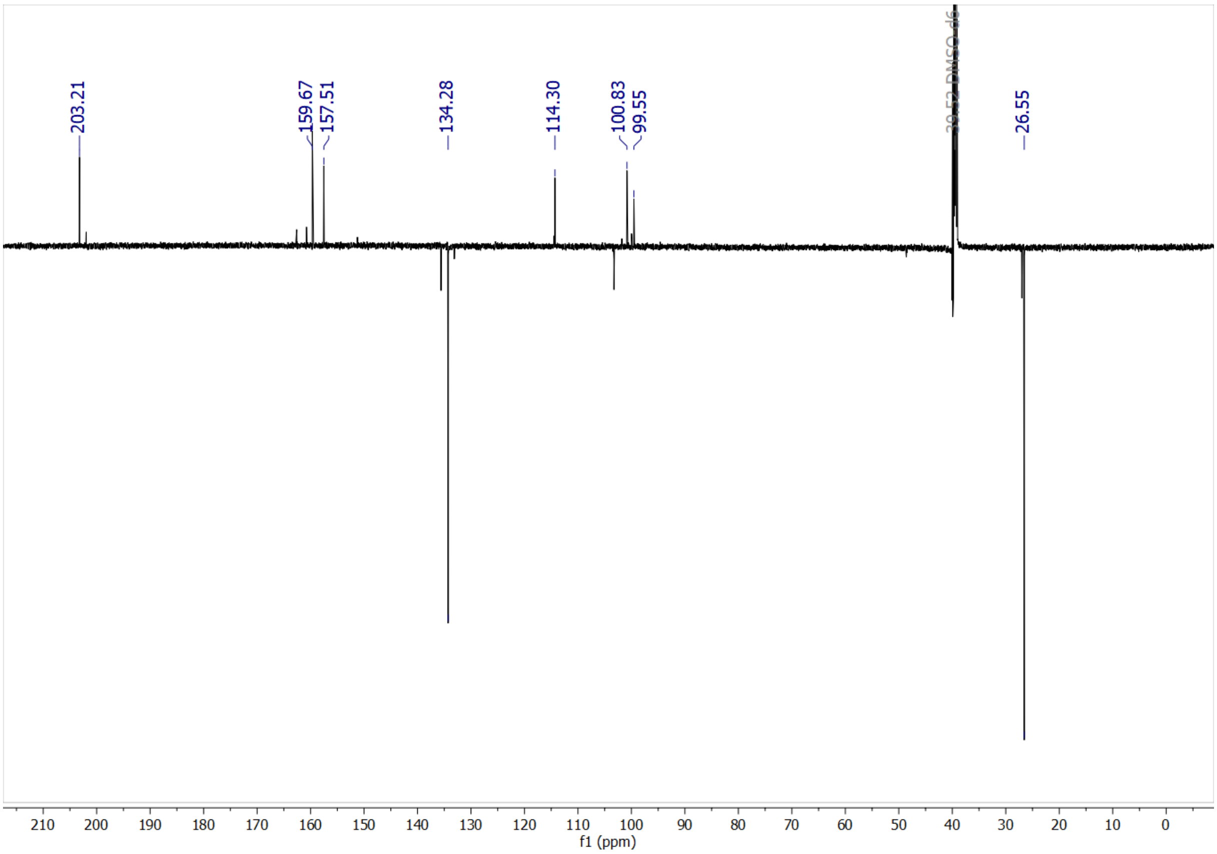

^{13}C DEPTQ NMR spectrum of **3,5-di-bromo-2,4-dihydroxyacetophenone** in DMSO-d_6 at 151 MHz.

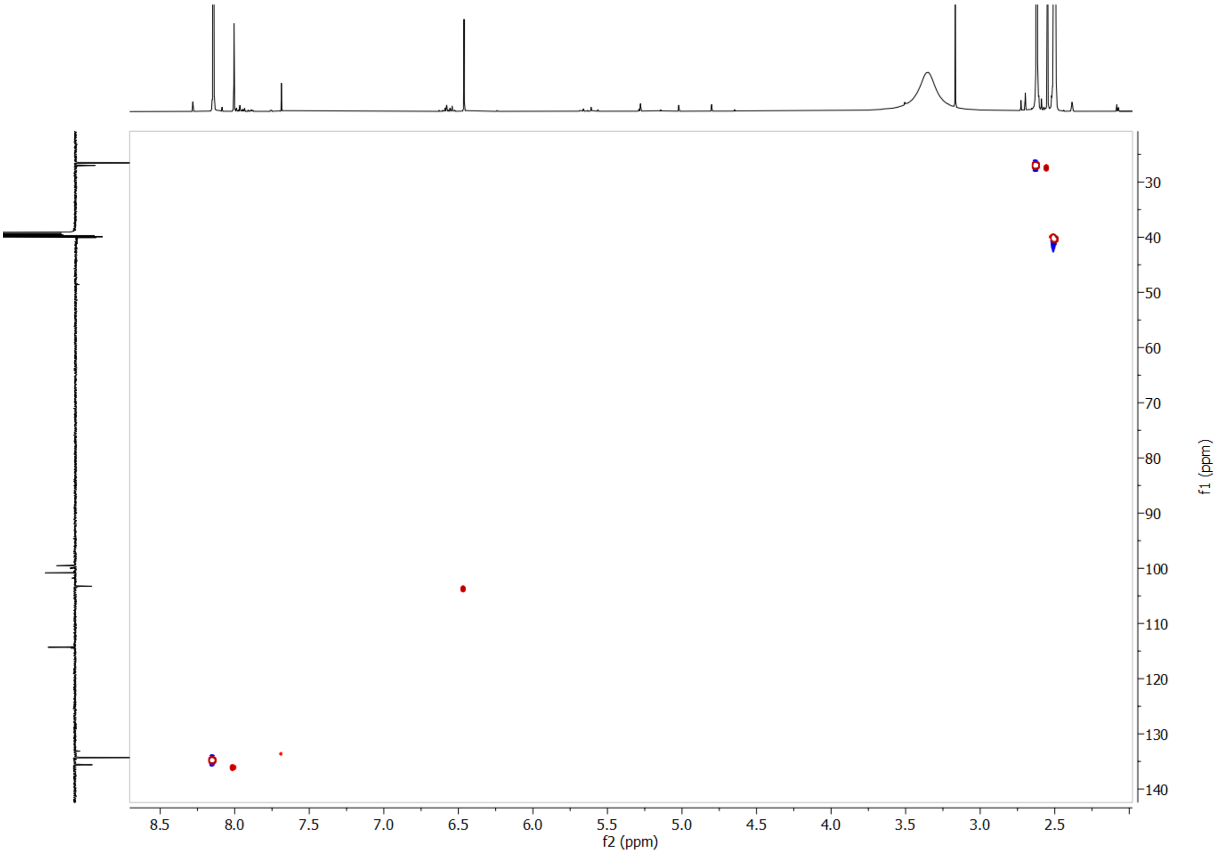

HSQC NMR spectrum of **3,5-di-bromo-2,4-dihydroxyacetophenone** in DMSO-d_6 at 600 MHz.

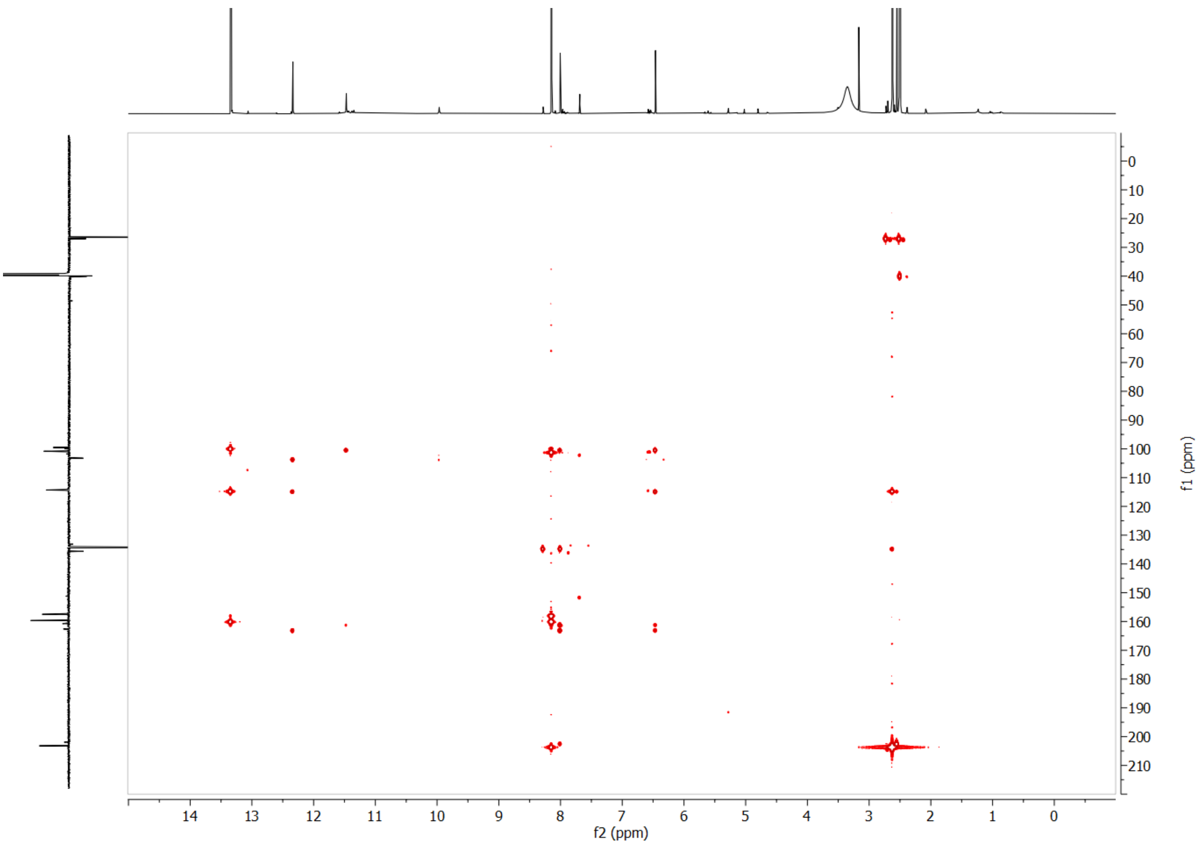

HMBC NMR spectrum of **3,5-di-bromo-2,4-dihydroxyacetophenone** in DMSO-d_6 at 600 MHz.

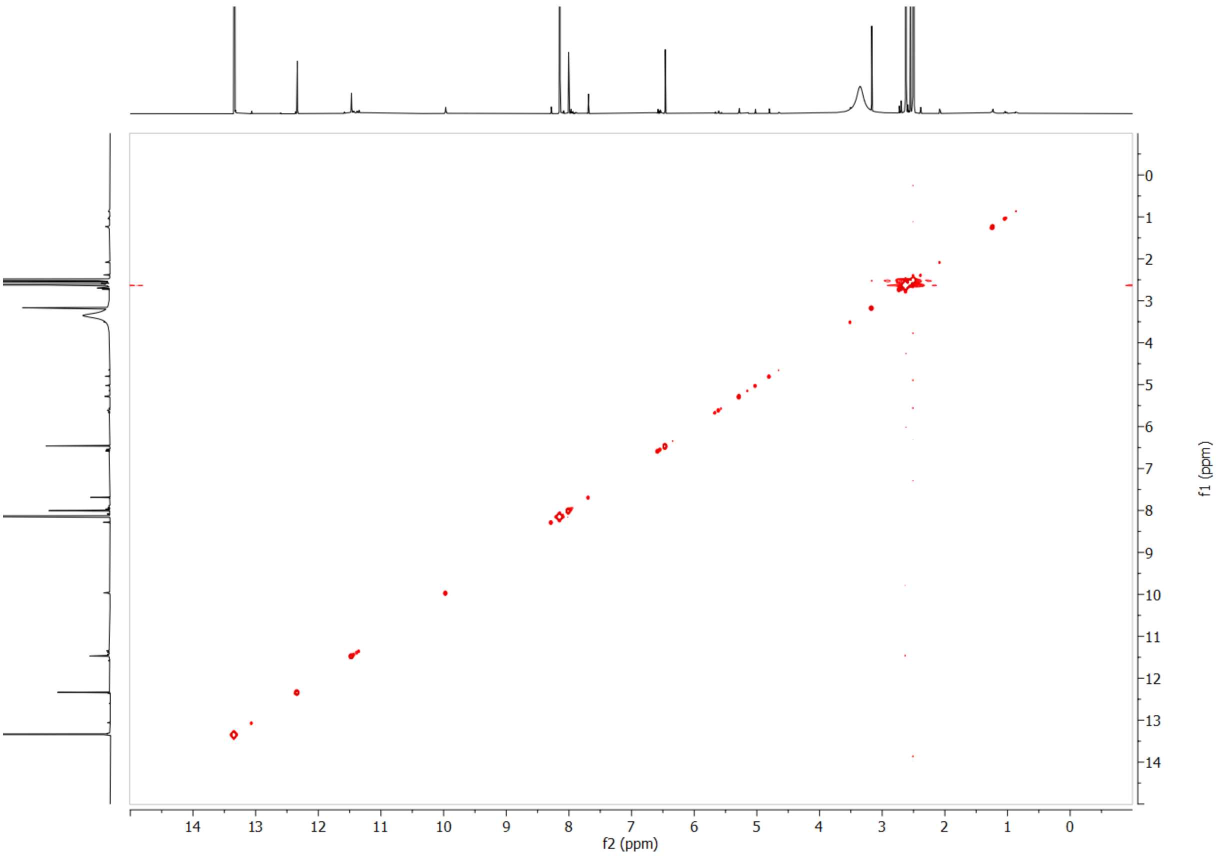

COSY NMR spectrum of **3,5-di-bromo-2,4-dihydroxyacetophenone** in DMSO-d_6 at 600 MHz.

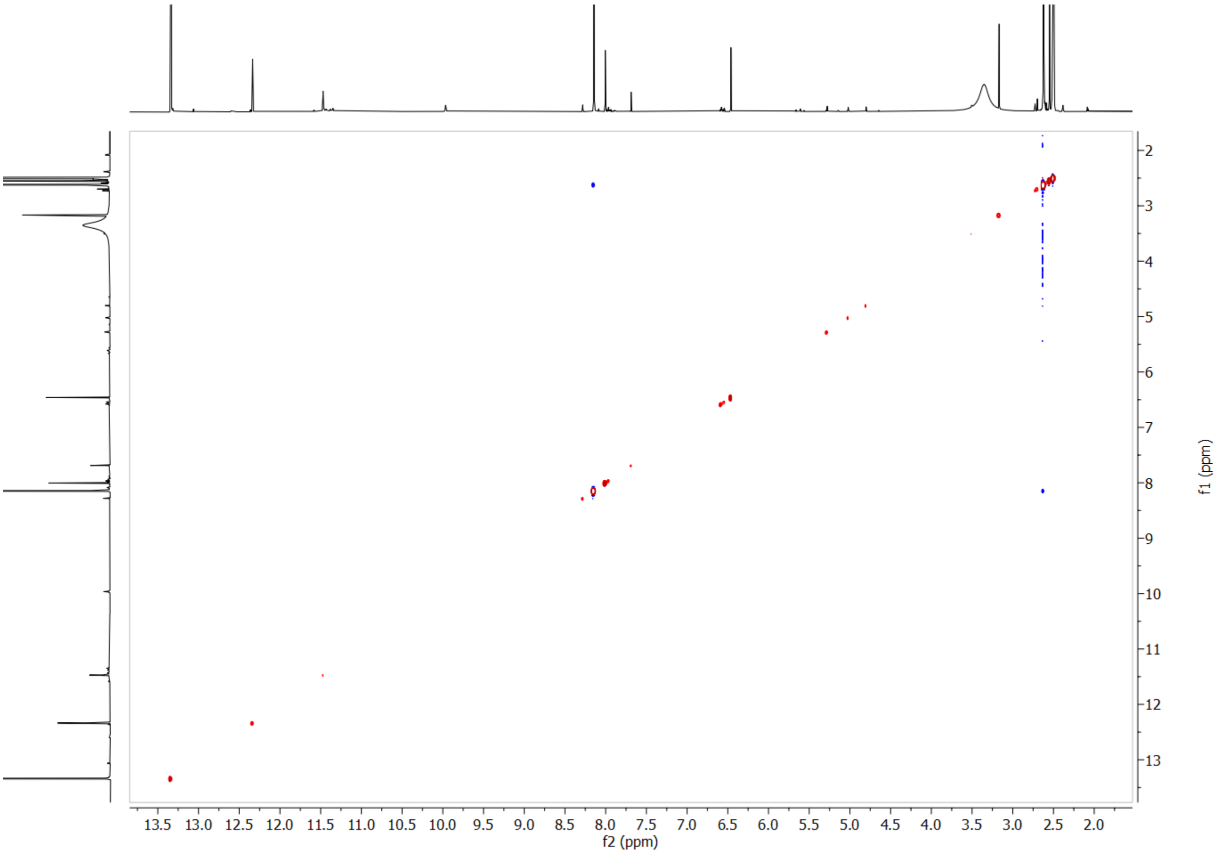

ROESY NMR spectrum of **3,5-di-bromo-2,4-dihydroxyacetophenone** in DMSO-d_6 at 600 MHz.

21. 3-chloro-2,4-dihydroxychalcone:

Experimental:

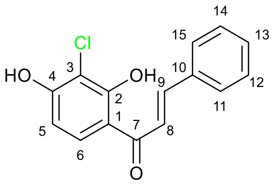

3-chloro-2,4-dihydroxychalcone (**21**) yellow solid; ^{1}H NMR (DMSO-d_6, 600 MHz) δ 14.21 (1H, s), 11.56 (1H, s), 8.23 (1H, d, J = 9.0 Hz), 8.00 (1H, d, J = 15.5 Hz), 7.92 (2H, dd, J = 6.6, 2.9 Hz), 7.86 (1H, d, J = 15.4 Hz), 7.50 – 7.46 (3H, m), 6.63 (1H, d, J = 8.9 Hz); ^{13}C NMR (DMSO-d_6, 151 MHz) δ 193.0, 161.5, 144.6, 134.5, 130.9, 130.6, 129.2, 129.0, 120.8, 113.1, 107.8, 106.9; HRESIMS *m/z* 275.0467 [M+H]$^+$ (calcd for $C_{15}H_{12}ClO_3^+$ 275.0469), *m/z* 273.0322 [M-H]$^-$ (calcd for $C_{15}H_{10}ClO_3^-$ 273.0324).

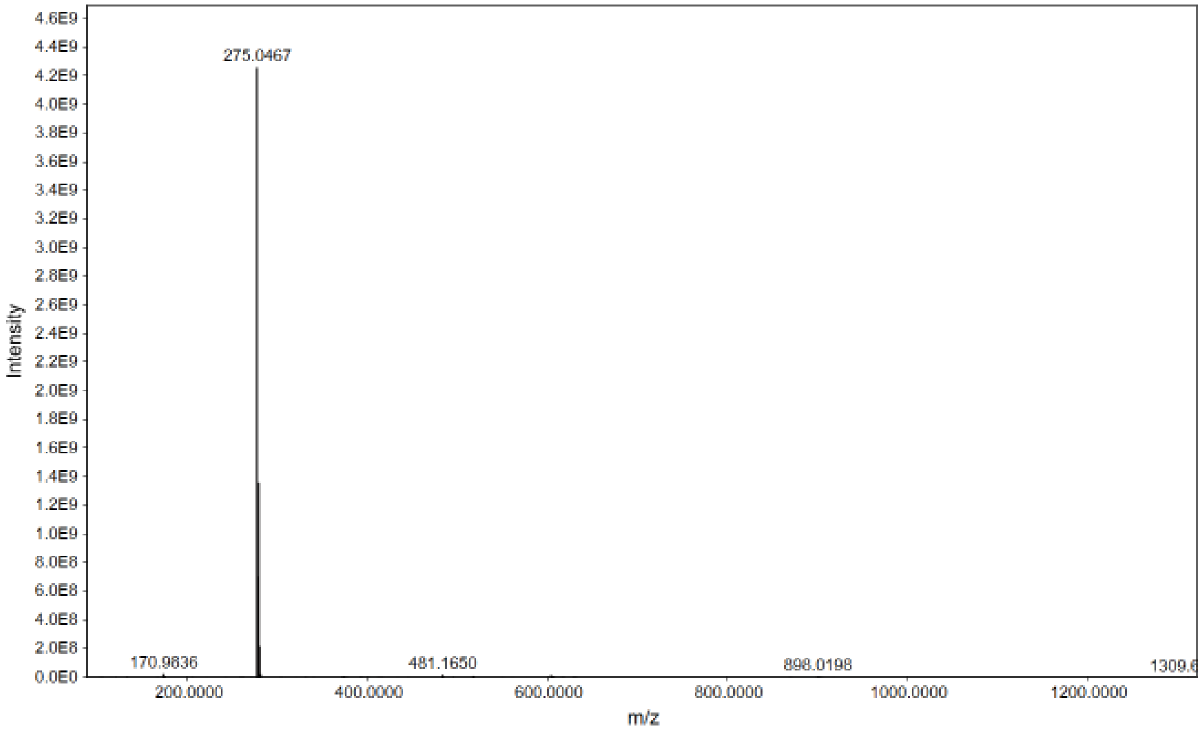

HRESIMS+ spectrum of **3-chloro-2,4-dihydroxychalcone** in MeOH.

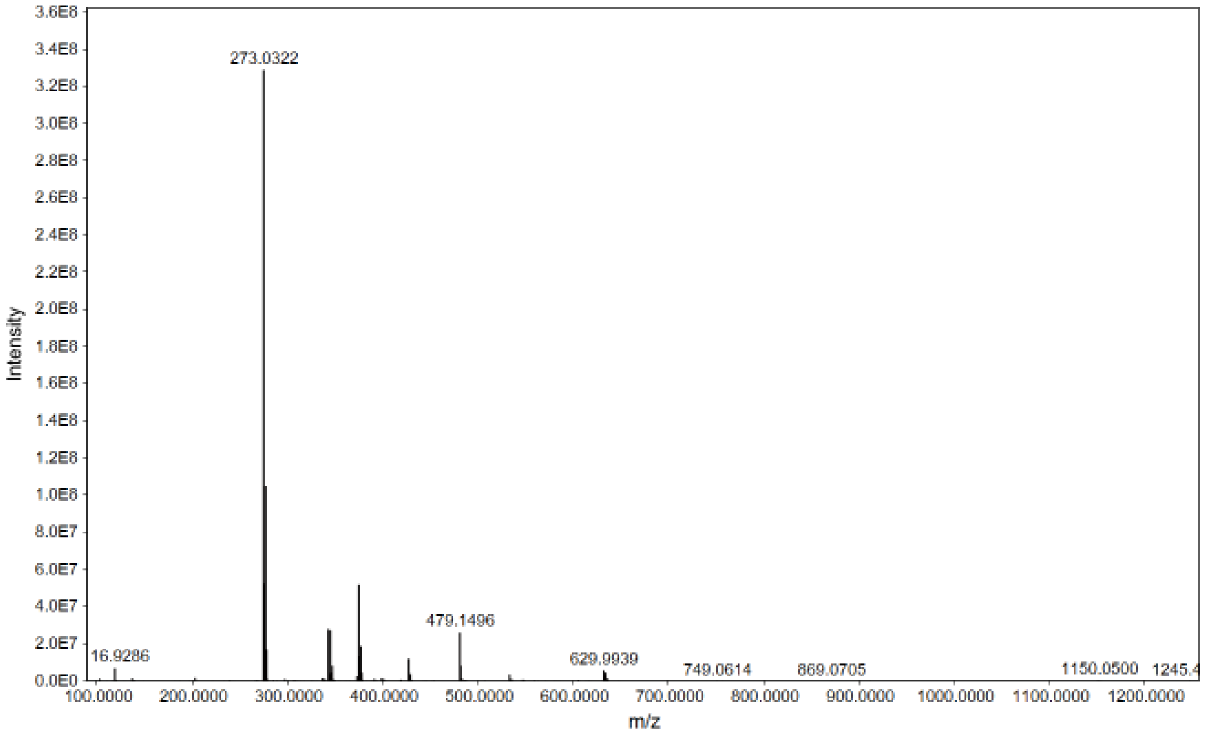

HRESIMS- spectrum of **3-chloro-2,4-dihydroxychalcone** in MeOH.

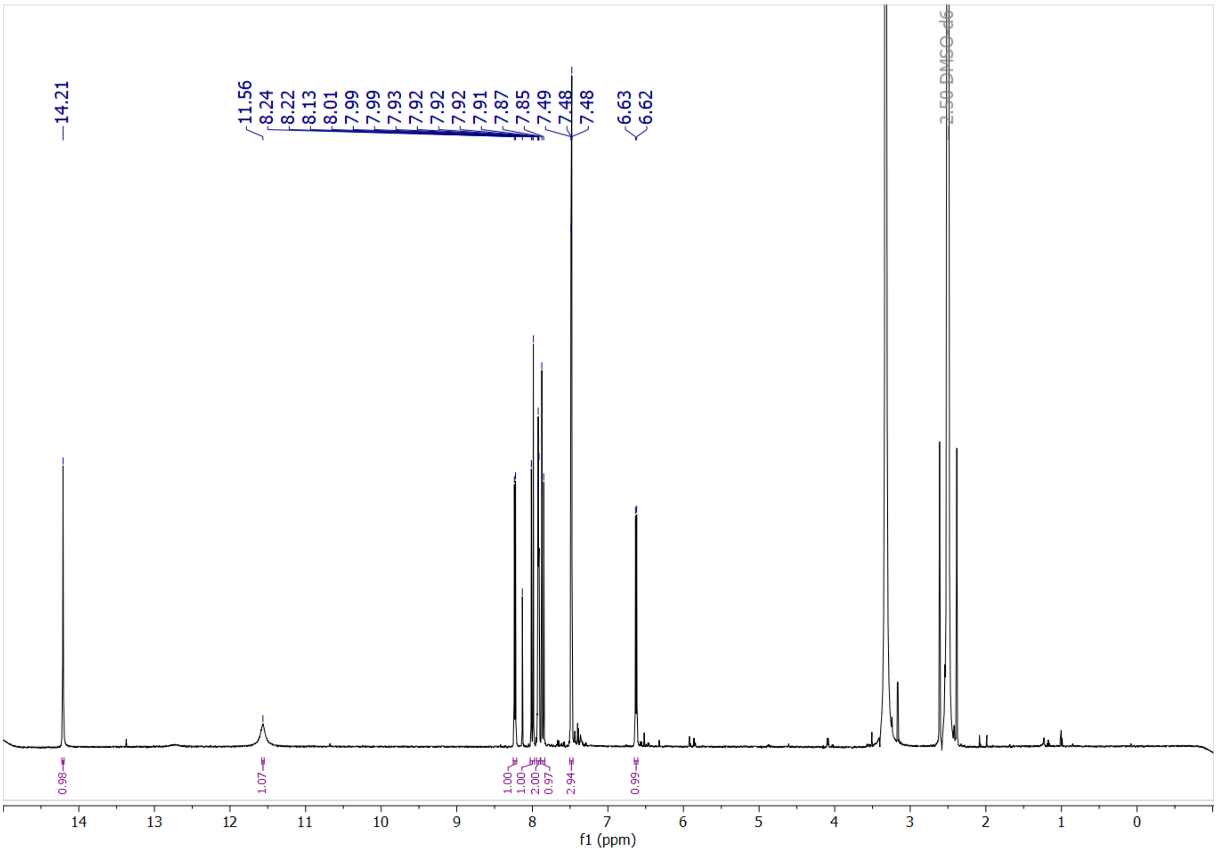

^{1}H NMR spectrum of **3-chloro-2,4-dihydroxychalcone** in DMSO-d_6 at 600 MHz.

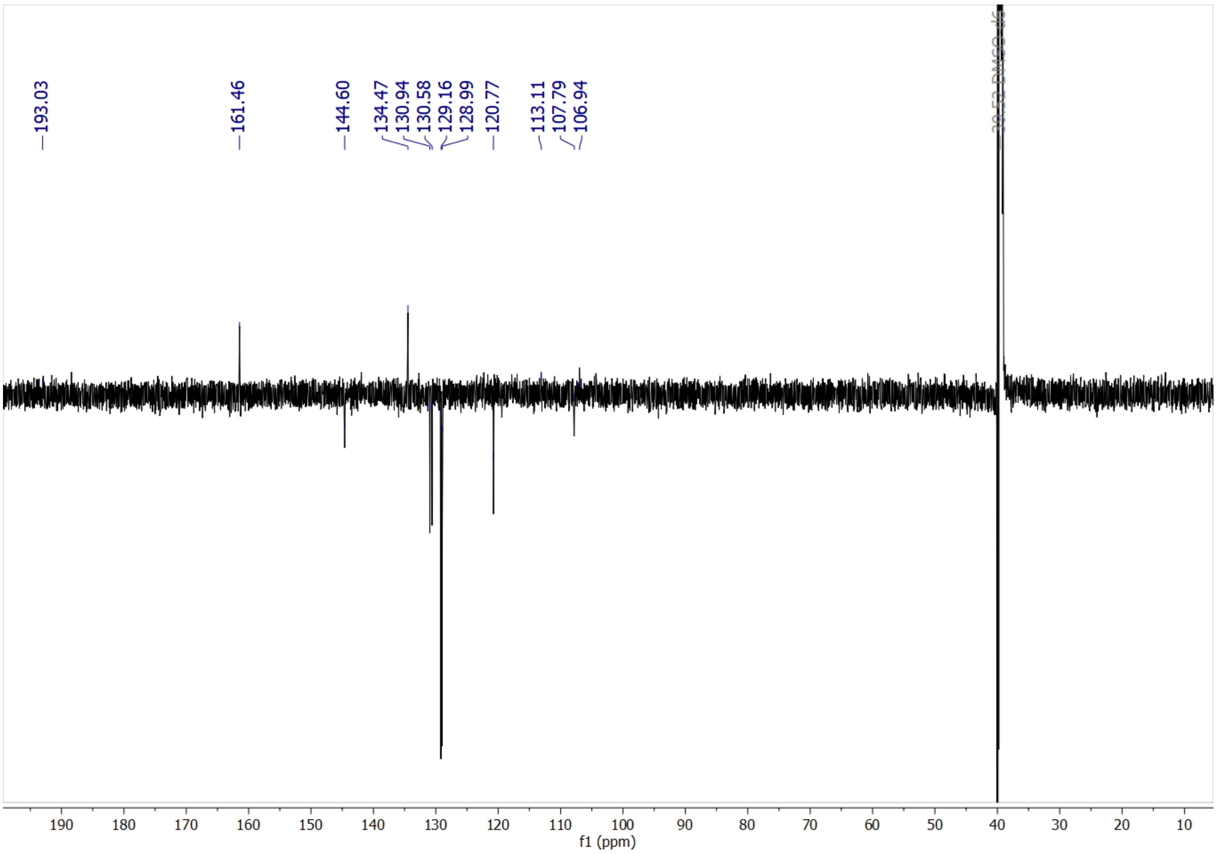

^{13}C DEPTQ NMR spectrum of **3-chloro-2,4-dihydroxychalcone** in DMSO-d_6 at 151 MHz.

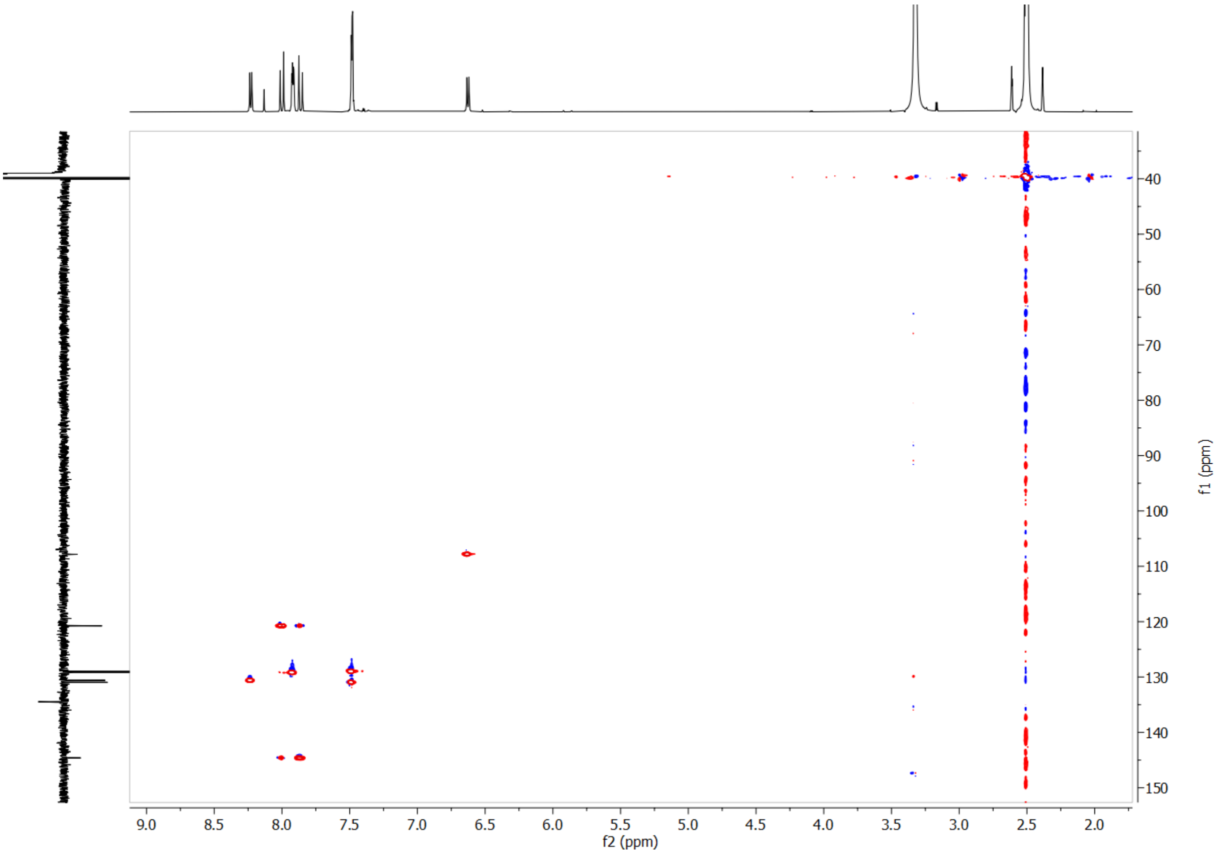

HSQC NMR spectrum of **3-chloro-2,4-dihydroxychalcone** in DMSO-d_6 at 600 MHz.

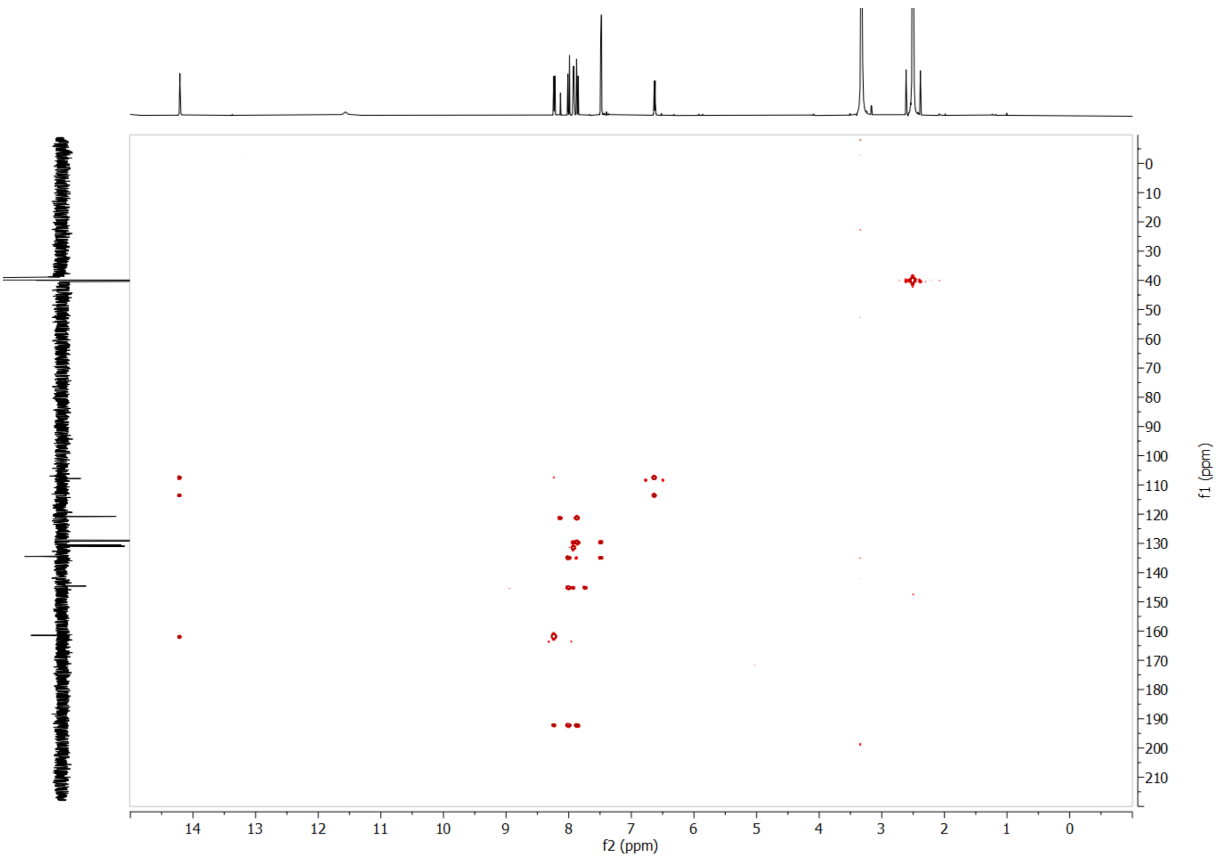

HMBC NMR spectrum of **3-chloro-2,4-dihydroxychalcone** in DMSO-d_6 at 600 MHz.

22. 5-chloro-2,4-dihydroxychalcone:

Experimental:

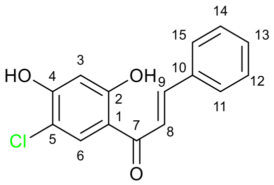

5-chloro-2,4-dihydroxychalcone (**22**) yellow solid; ^{1}H NMR (DMSO-d_6, 600 MHz) δ 13.23 (1H, s), 8.42 (1H, s), 8.04 (1H, d, J = 15.4 Hz), 7.95 (2H, dd, J = 6.6, 3.0 Hz), 7.81 (1H, d, J = 15.4 Hz), 7.47 (3H, ddt, J = 5.7, 3.9, 2.2 Hz), 6.50 (1H, s); ^{13}C NMR (DMSO-d_6, 151 MHz) δ 191.09, 163.8, 144.4, 134.6, 132.0, 130.8, 129.3, 128.9, 121.3, 113.6, 111.9, 103.7; HRESIMS *m/z* 275.0467 $[M+H]^+$ (calcd for $C_{15}H_{12}ClO_3^+$ 275.0469), *m/z* 273.0323 $[M-H]^-$ (calcd for $C_{15}H_{10}ClO_3^-$ 273.0324).

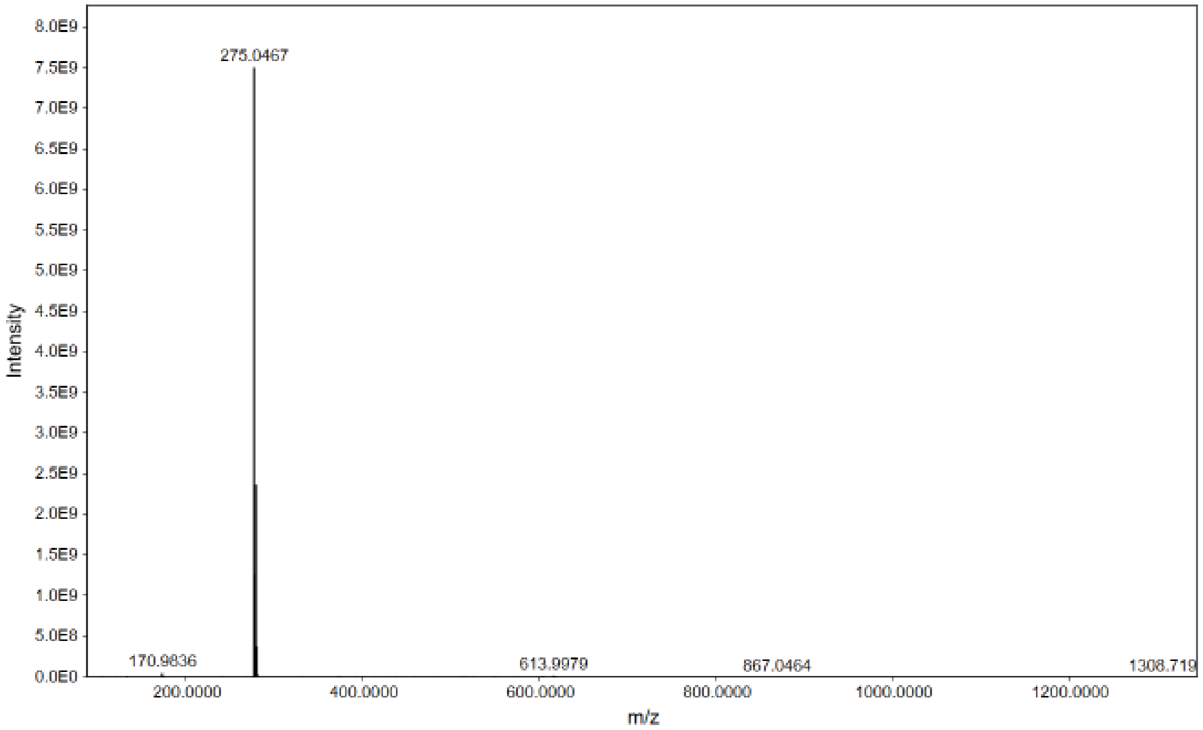

HRESIMS+ spectrum of **5-chloro-2,4-dihydroxychalcone** in MeOH.

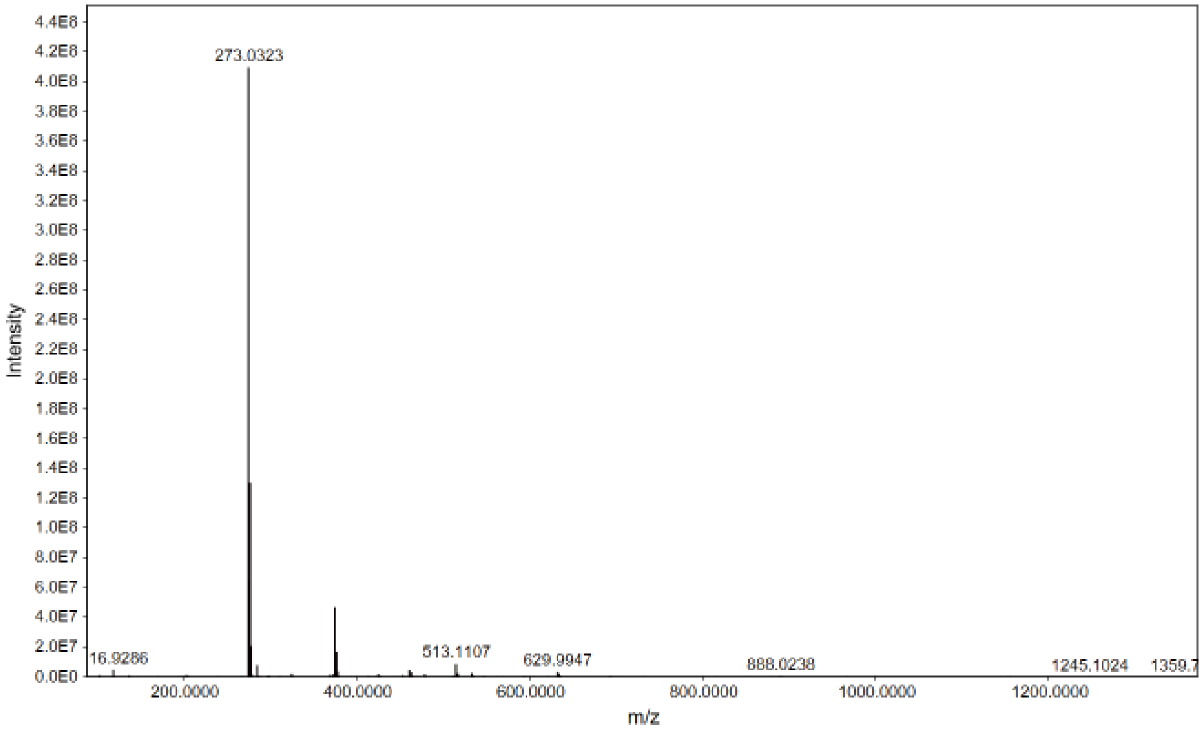

HRESIMS- spectrum of **5-chloro-2,4-dihydroxychalcone** in MeOH.

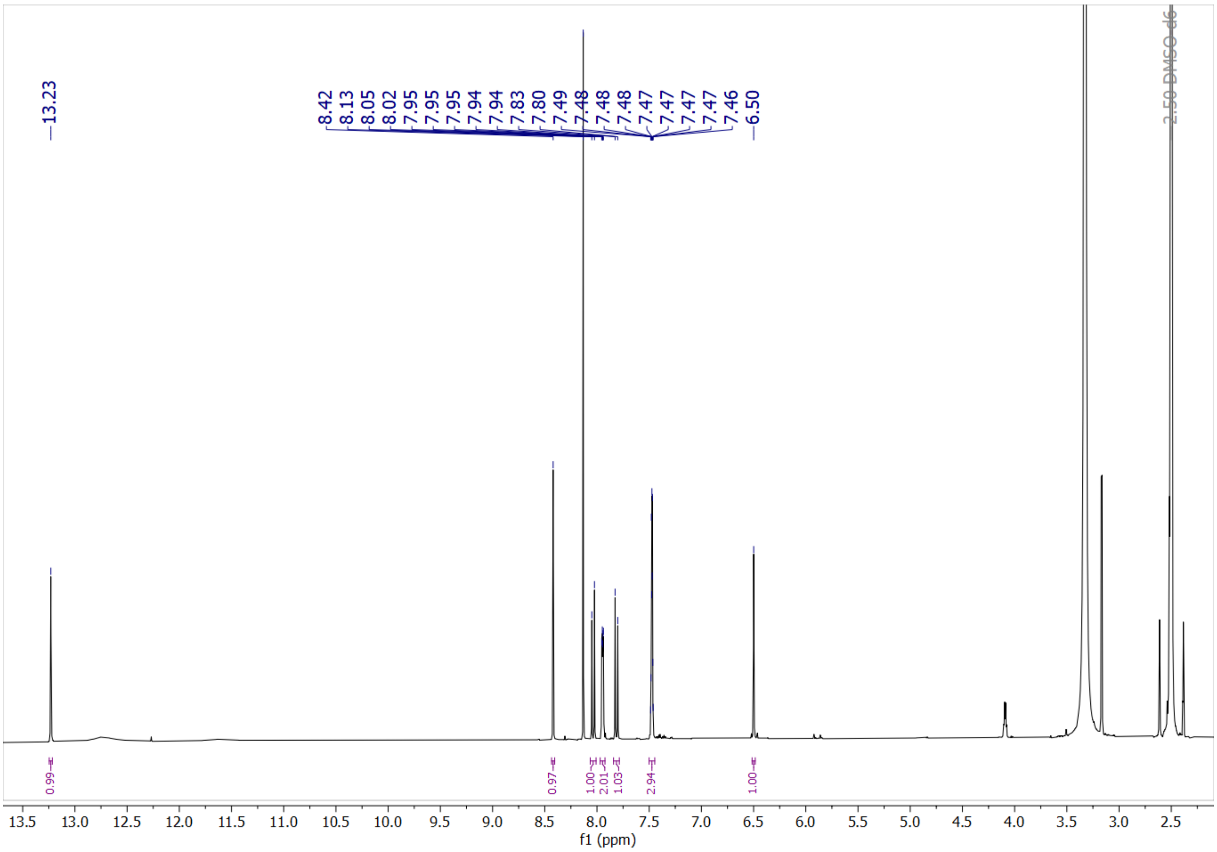

^{1}H NMR spectrum of **5-chloro-2,4-dihydroxychalcone** in DMSO-d_6 at 600 MHz.

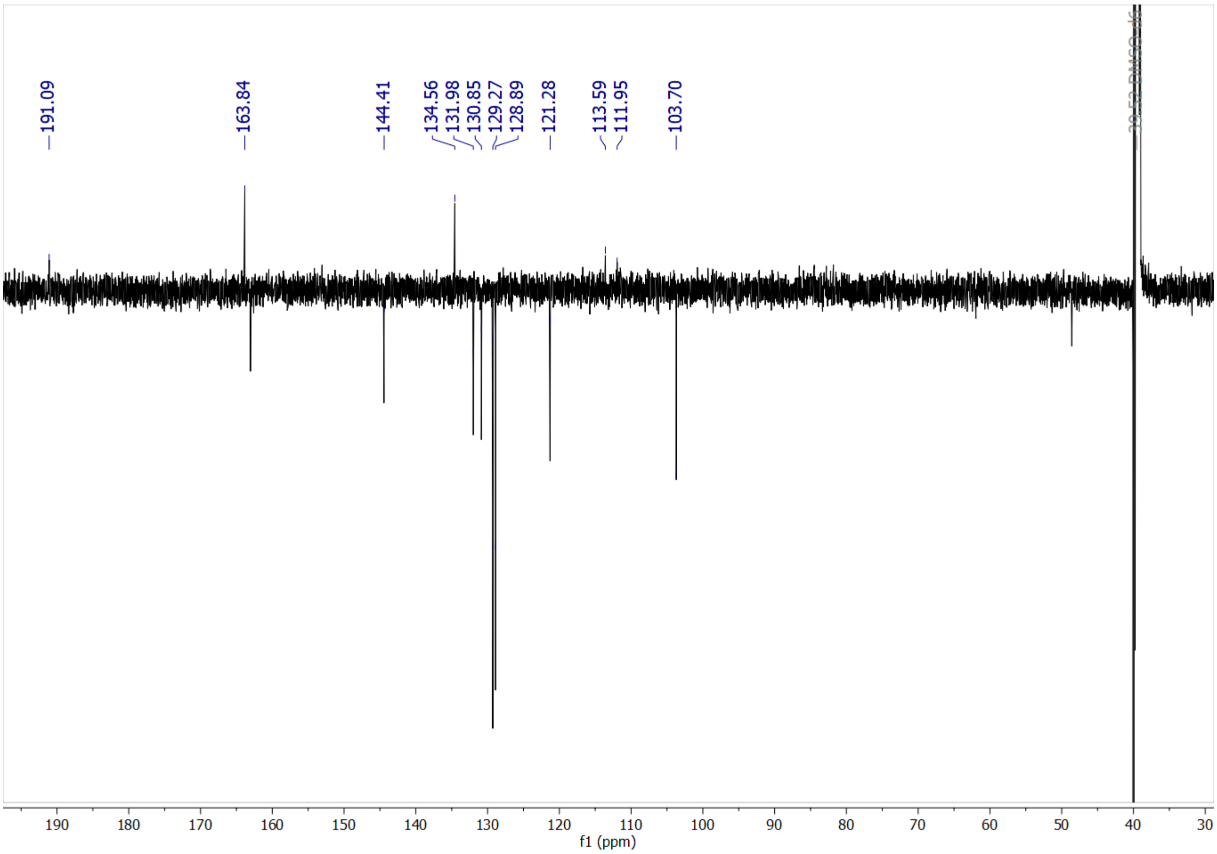

^{13}C DEPTQ NMR spectrum of **5-chloro-2,4-dihydroxychalcone** in DMSO-d_6 at 151 MHz.

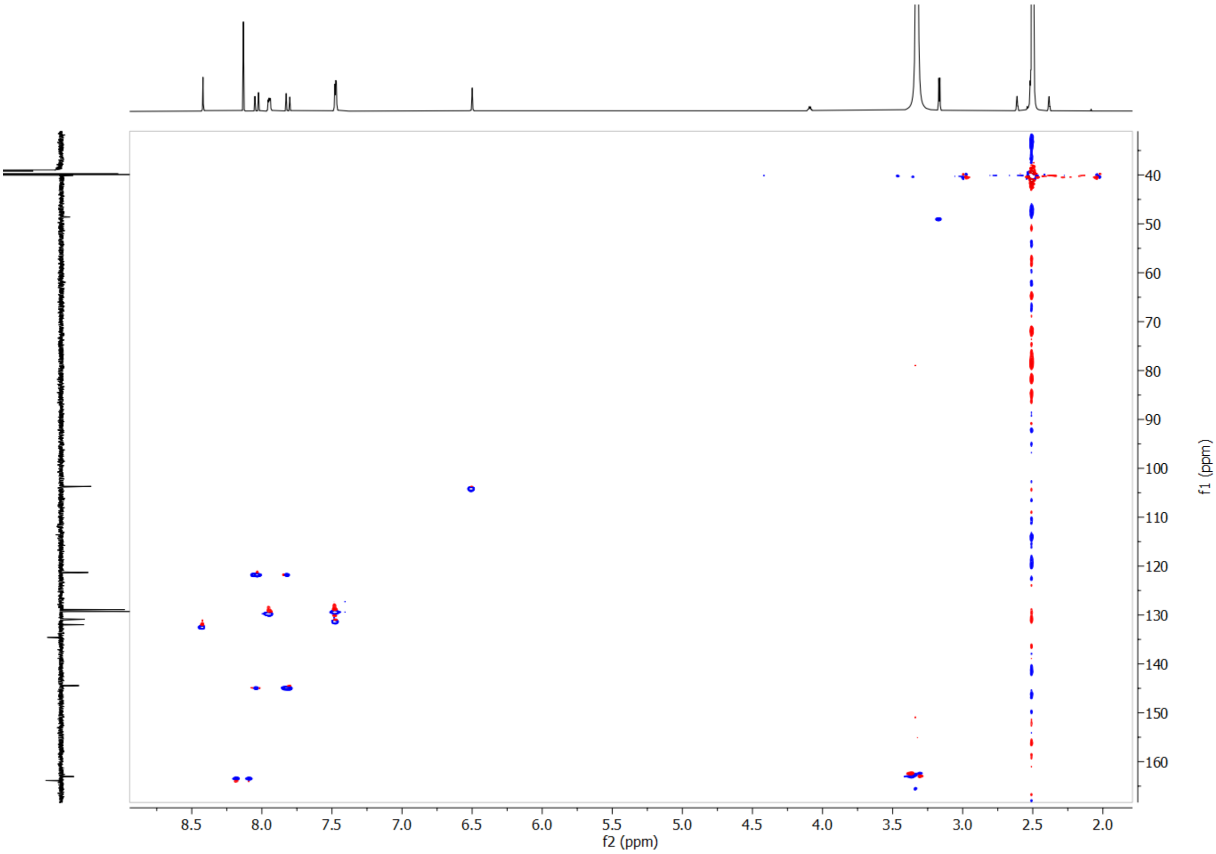

HSQC NMR spectrum of **5-chloro-2,4-dihydroxychalcone** in DMSO-d_6 at 600 MHz.

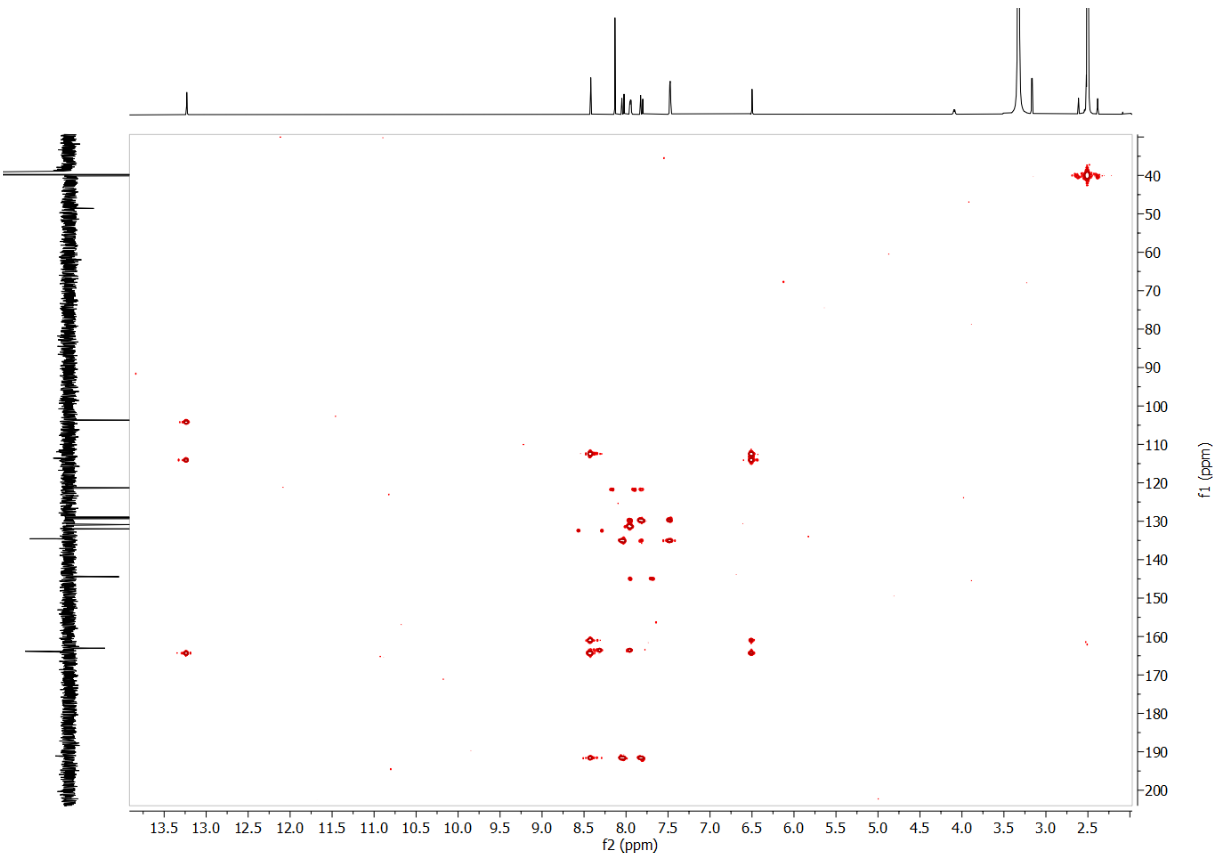

HMBC NMR spectrum of **5-chloro-2,4-dihydroxychalcone** in DMSO-d_6 at 600 MHz.

23. 3,5-di-chloro-2,4-dihydroxychalcone:

Experimental:

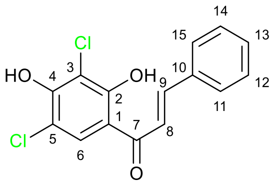

3,5-di-chloro-2,4-dihydroxychalcone (**23**) yellow solid; ^{1}H NMR (DMSO-d_6, 600 MHz) δ 14.15 (1H, s), 8.53 (1H, s), 8.08 (1H, d, J = 15.4 Hz), 8.00 – 7.96 (2H, m), 7.88 (1H, d, J = 15.4 Hz), 7.49 (3H, dd, J = 4.8, 1.9 Hz); ^{13}C NMR (DMSO-d_6, 151 MHz) δ 190.1, 159.9, 144.3, 134.4, 131.1, 129.6, 129.5, 128.9, 120.7; HRESIMS *m/z* 309.0078 $[M+H]^+$ (calcd for $C_{15}H_{11}Cl_2O_3^+$ 309.0080), *m/z* 306.9933 $[M-H]^-$ (calcd for $C_{15}H_9Cl_2O_3^-$ 306.9934).

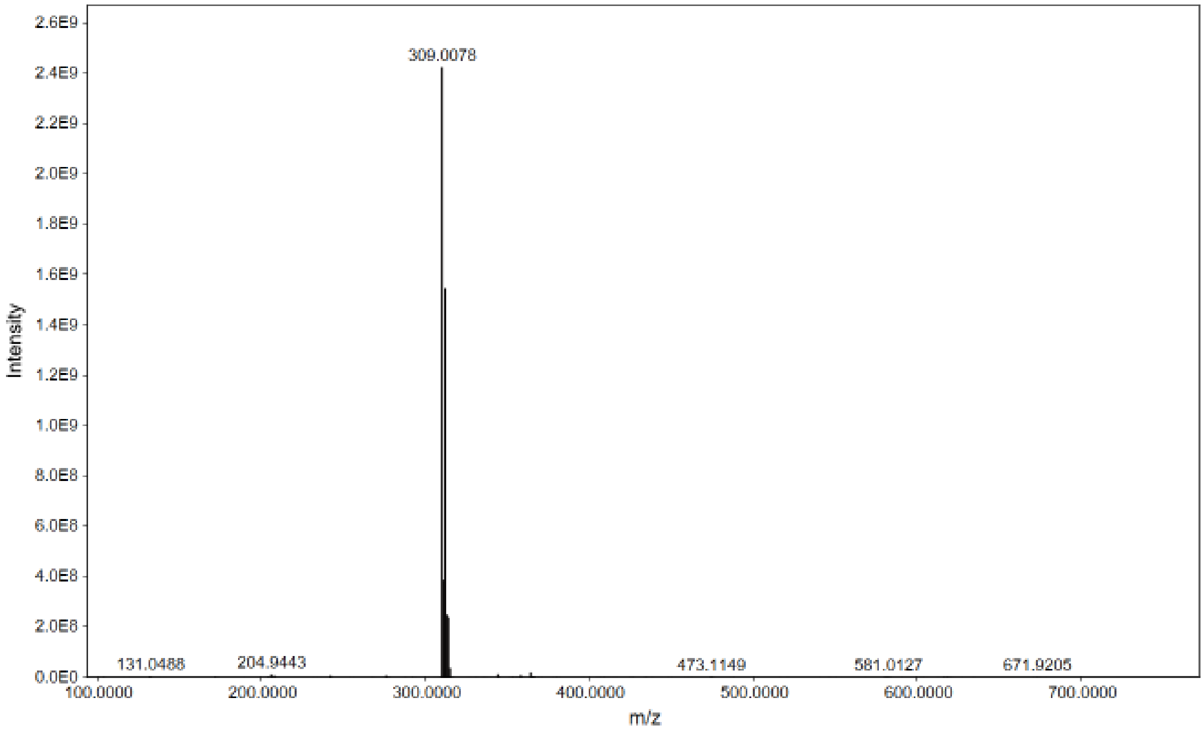

HRESIMS+ spectrum of **3,5-di-chloro-2,4-dihydroxychalcone** in MeOH.

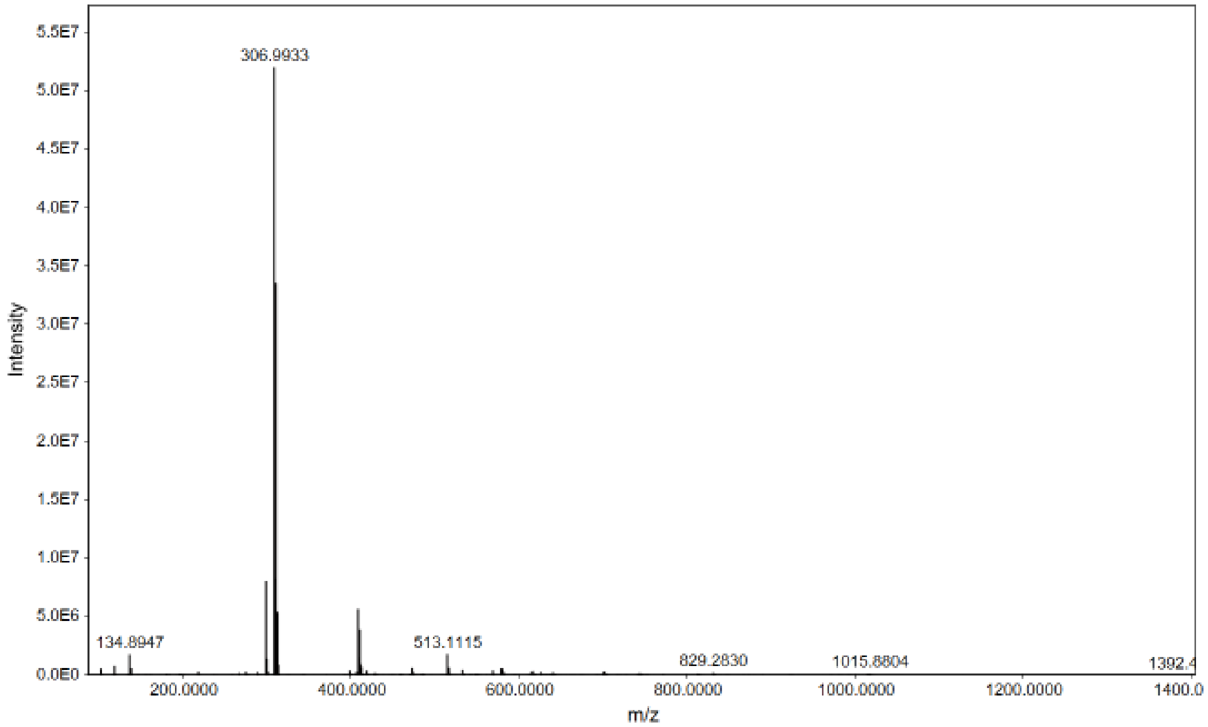

HRESIMS- spectrum of **3,5-di-chloro-2,4-dihydroxychalcone** in MeOH.

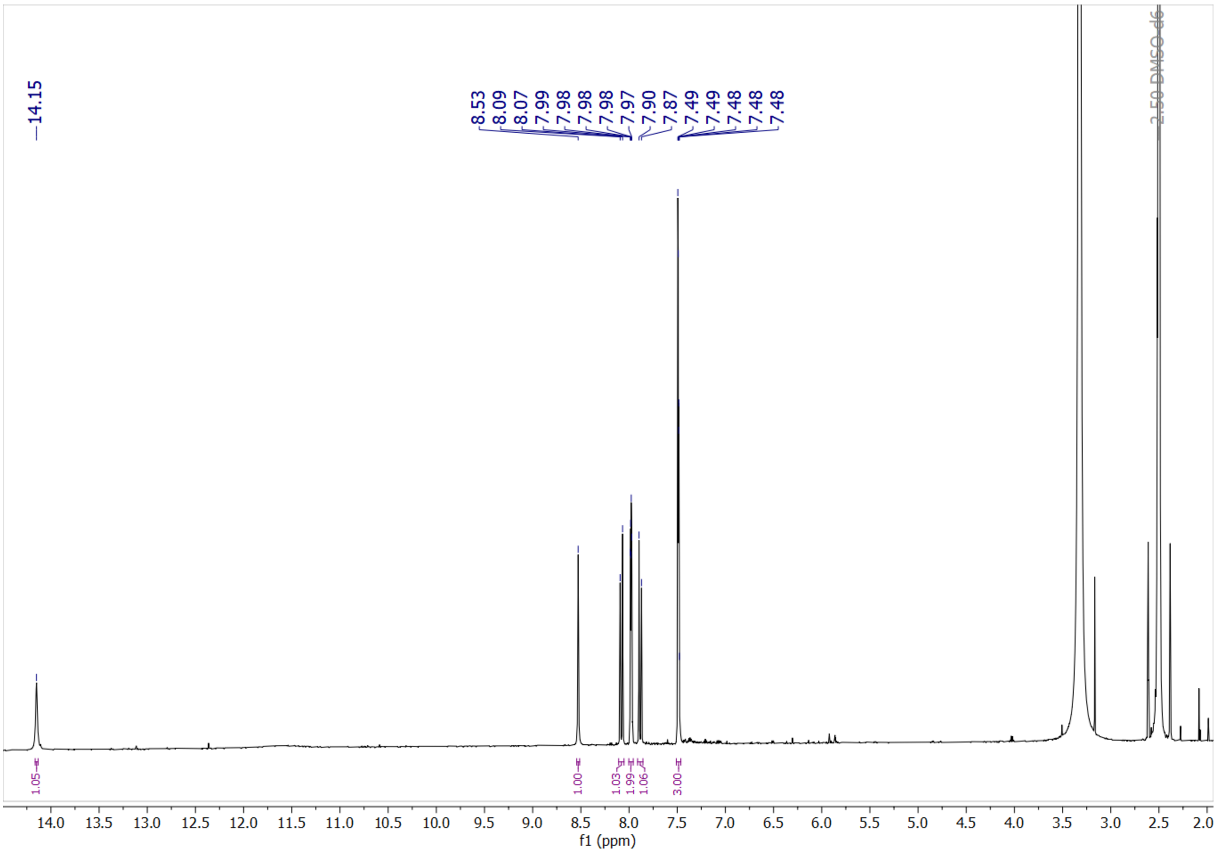

^{1}H NMR spectrum of **3,5-di-chloro-2,4-dihydroxychalcone** in DMSO-d_6 at 600 MHz.

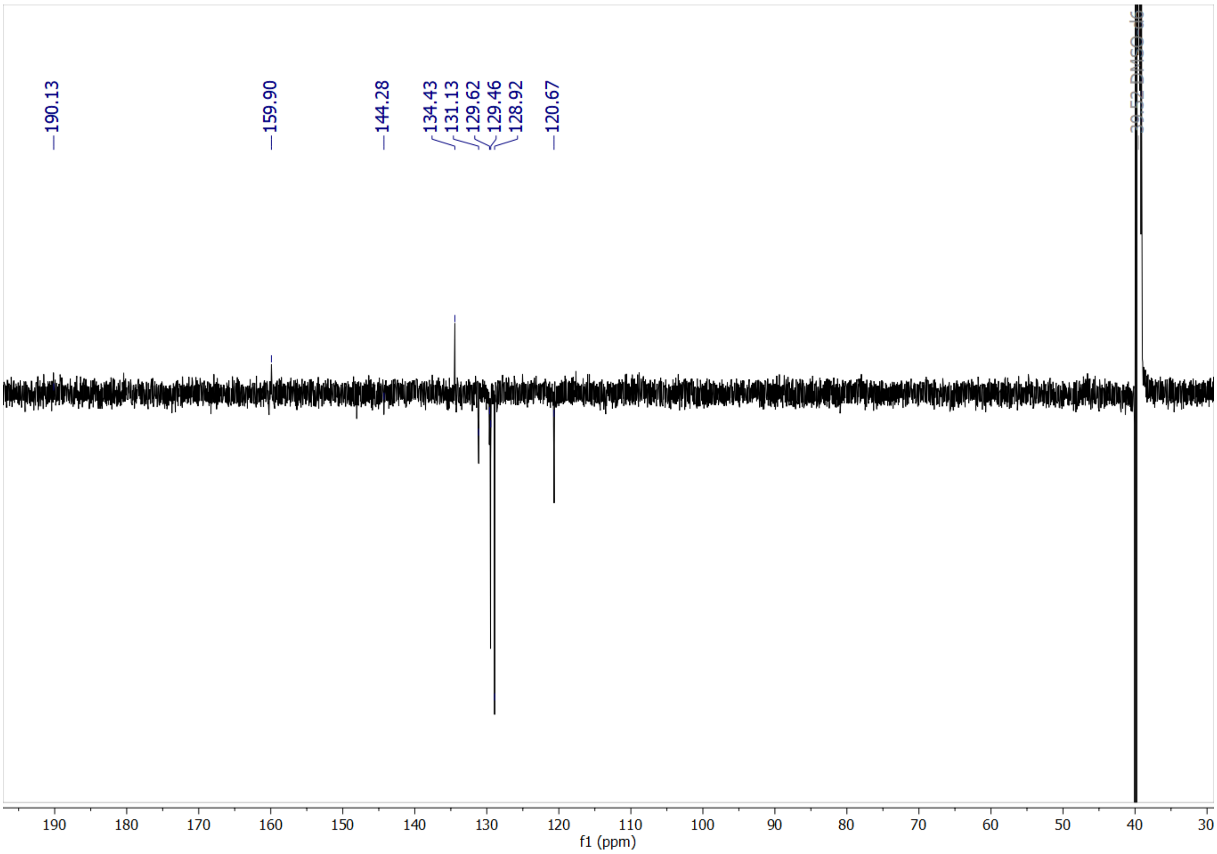

^{13}C DEPTQ NMR spectrum of **3,5-di-chloro-2,4-dihydroxychalcone** in DMSO-d_6 at 151 MHz.

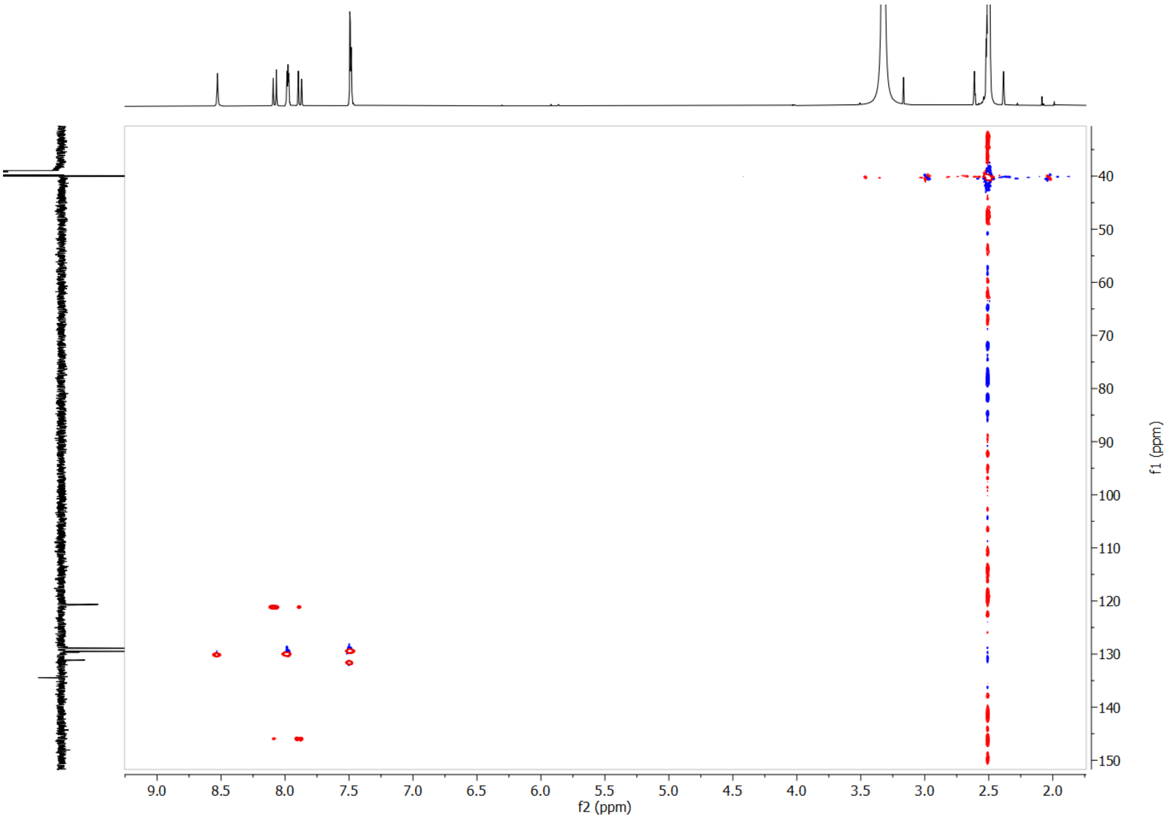

HSQC NMR spectrum of **3,5-di-chloro-2,4-dihydroxychalcone** in DMSO-d_6 at 600 MHz.

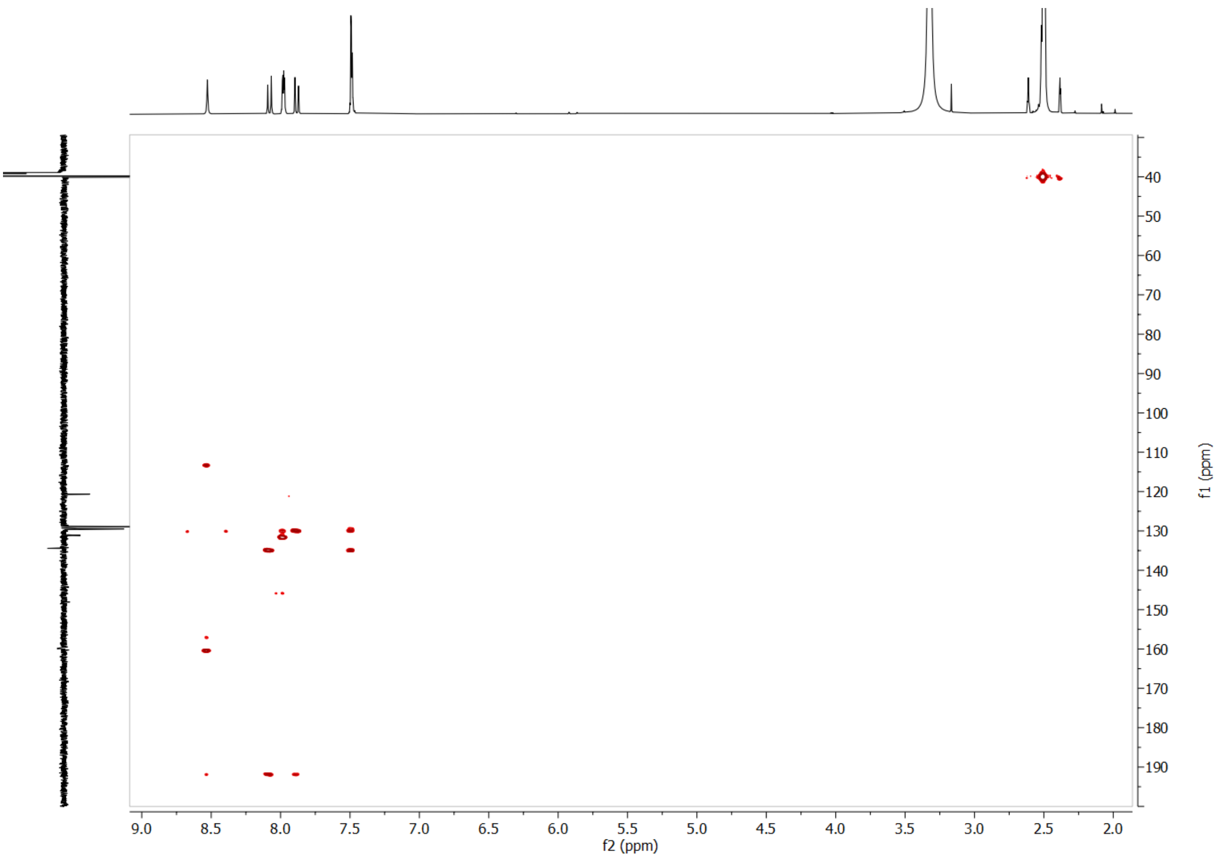

HMBC NMR spectrum of **3,5-di-chloro-2,4-dihydroxychalcone** in DMSO-d_6 at 600 MHz.

24. 3-bromo-2,4-dihydroxychalcone:

Experimental:

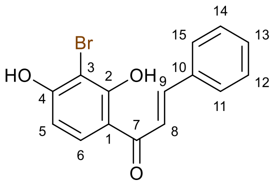

3-bromo-2,4-dihydroxychalcone (**24**) light brown solid; ^{1}H NMR (DMSO-d_6, 600 MHz) δ 14.34 (1H, s), 13.50 (1H, s), 8.27 (1H, d, J = 9.0 Hz), 8.01 (1H, d, J = 15.4 Hz), 7.92 (2H, dd, J = 6.6, 2.9 Hz), 7.87 (1H, d, J = 15.4 Hz), 7.50 – 7.46 (3H, m), 6.63 (2H, dd, J = 28.9, 8.8 Hz); ^{13}C NMR (DMSO-d_6, 151 MHz) δ 191.7, 162.5, 161.6, 144.7, 134.5, 131.5, 131.0, 129.2, 129.0, 120.7, 113.3, 107.7, 97.6; HRESIMS *m/z* 318.9964 $[M+H]^+$ (calcd for $C_{15}H_{12}BrO_3^+$ 318.9964), *m/z* 316.9818 $[M-H]^-$ (calcd for $C_{15}H_{10}BrO_3^-$ 316.9819).

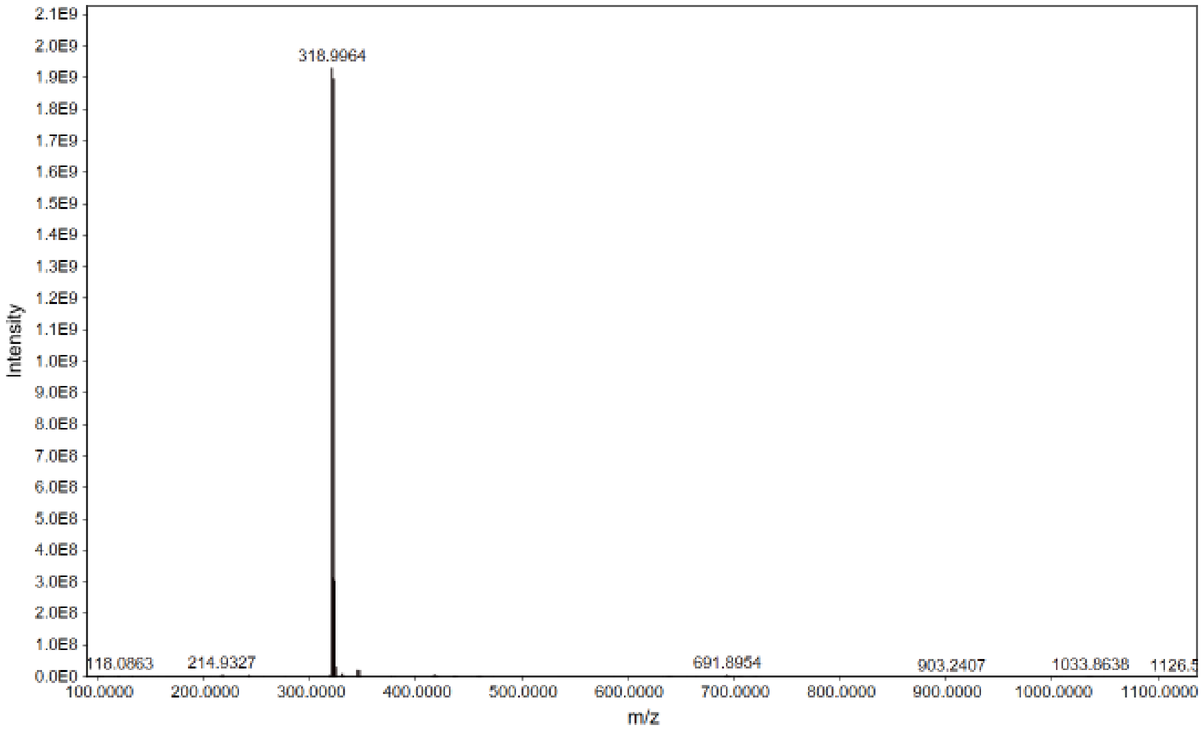

HRESIMS+ spectrum of **3-bromo-2,4-dihydroxychalcone** in MeOH.

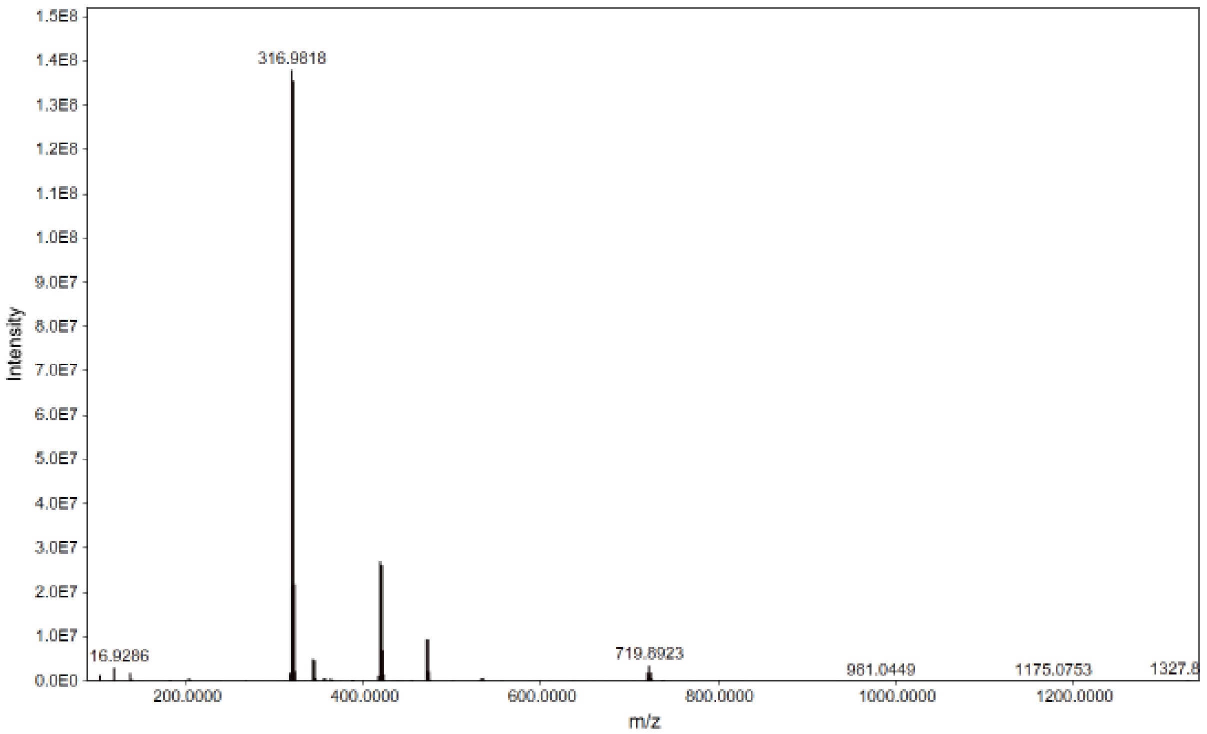

HRESIMS- spectrum of **3-bromo-2,4-dihydroxychalcone** in MeOH.

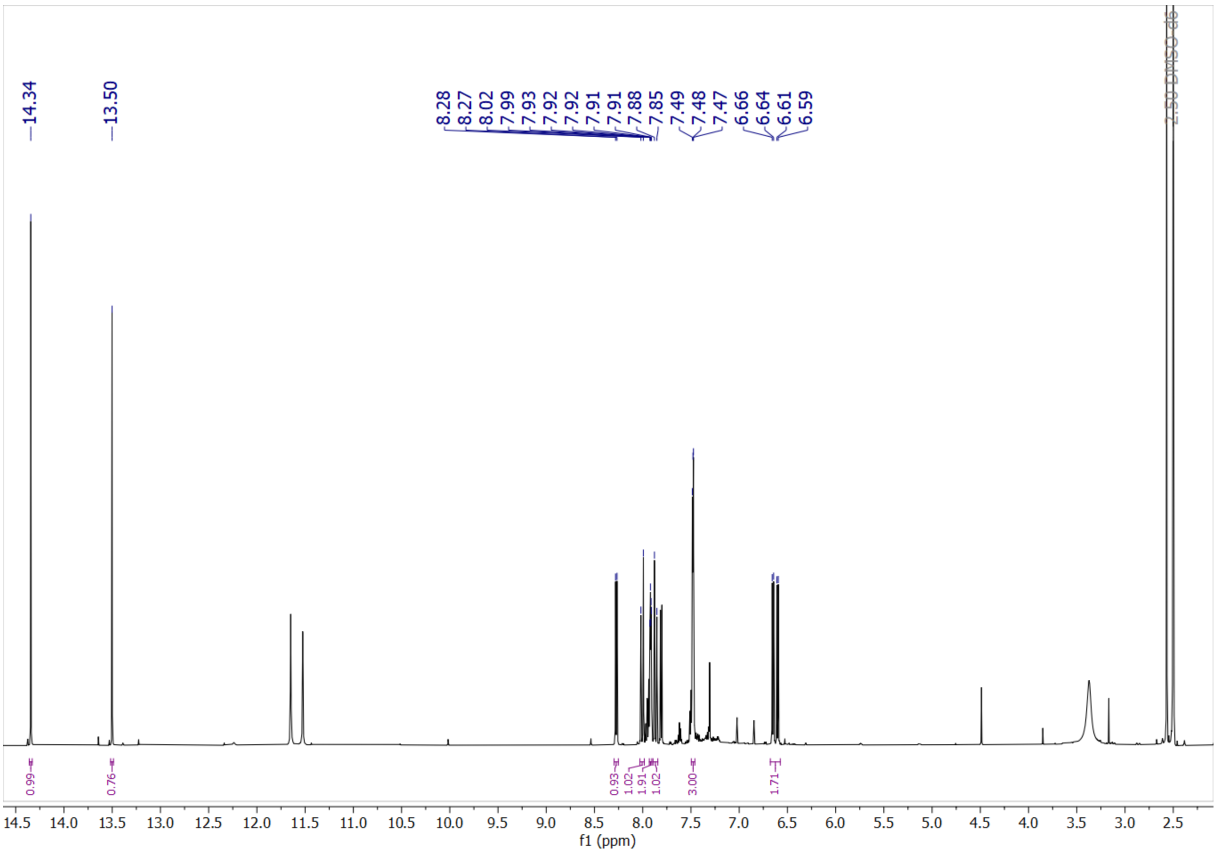

^{1}H NMR spectrum of **3-bromo-2,4-dihydroxychalcone** in DMSO-d_6 at 600 MHz.

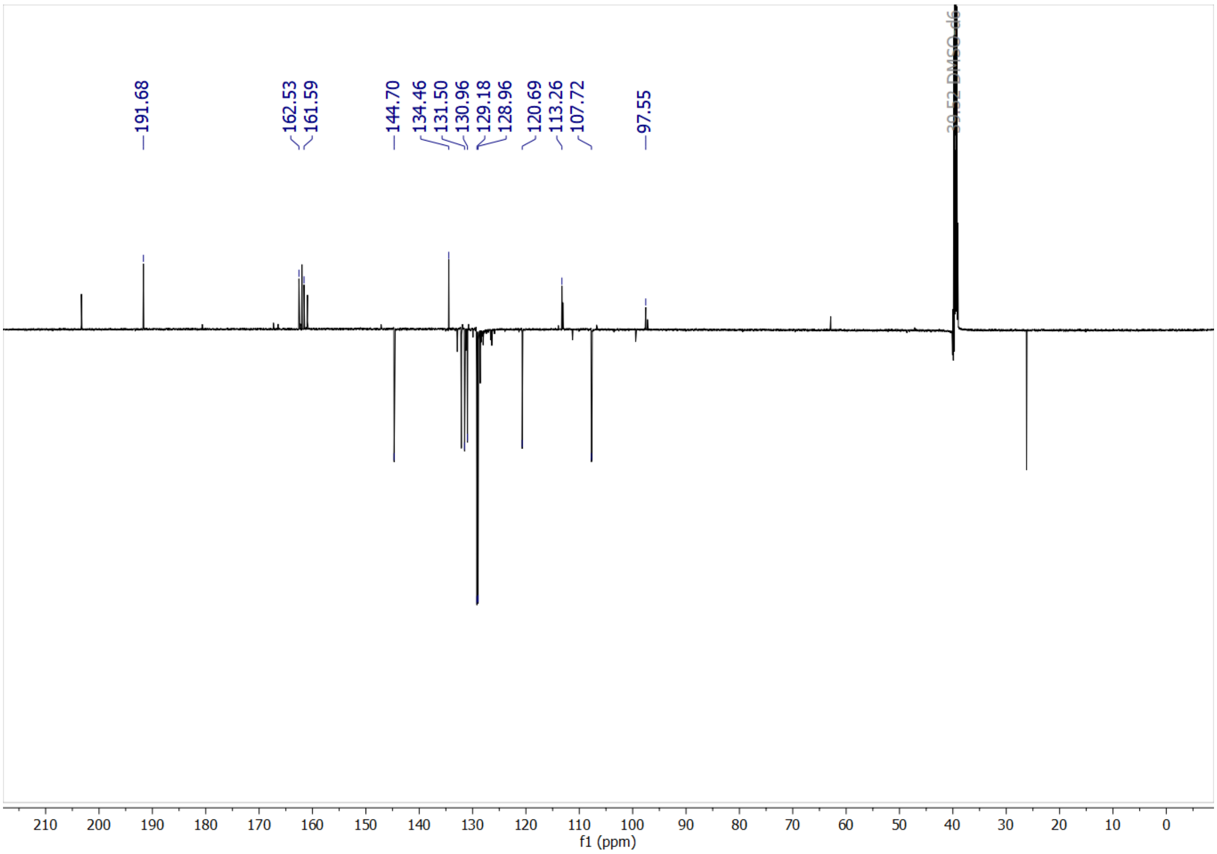

^{13}C DEPTQ NMR spectrum of **3-bromo-2,4-dihydroxychalcone** in DMSO-d_6 at 151 MHz.

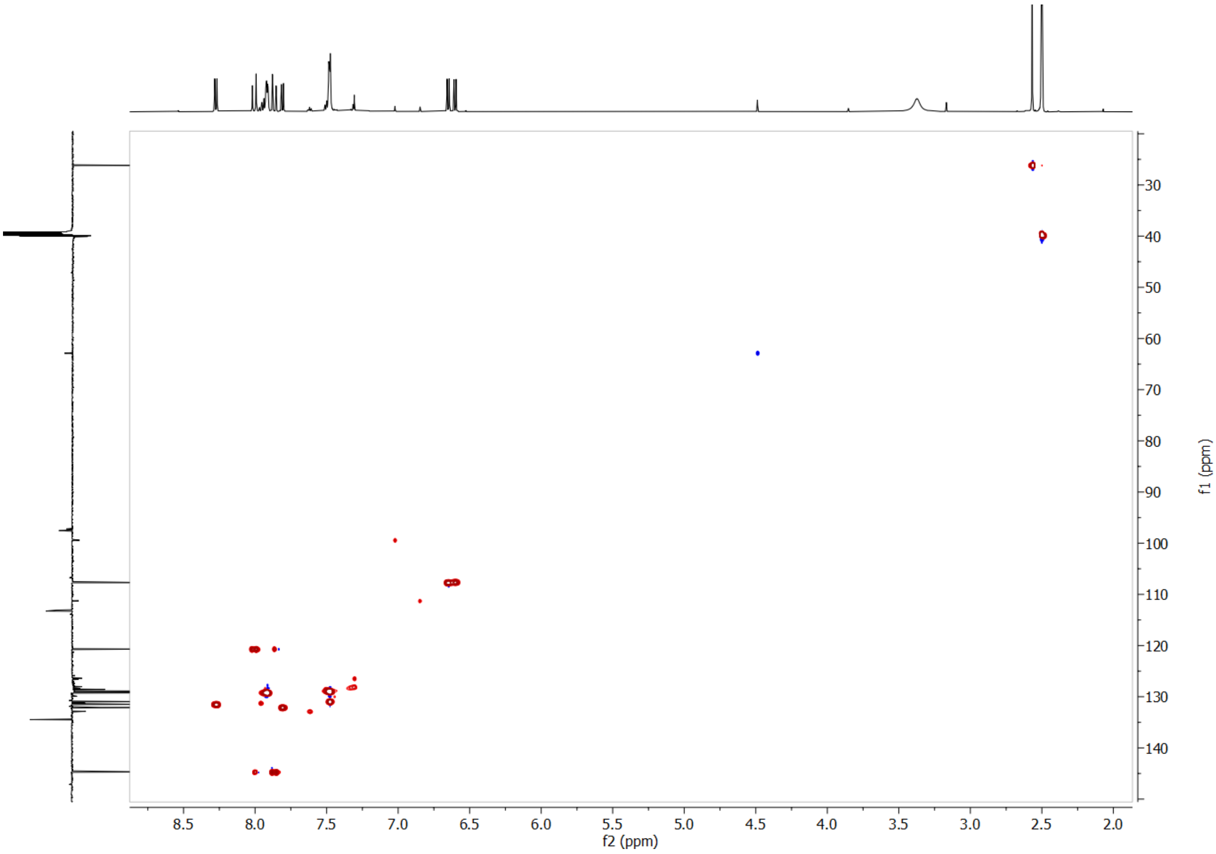

HSQC NMR spectrum of **3-bromo-2,4-dihydroxychalcone** in DMSO-d_6 at 600 MHz.

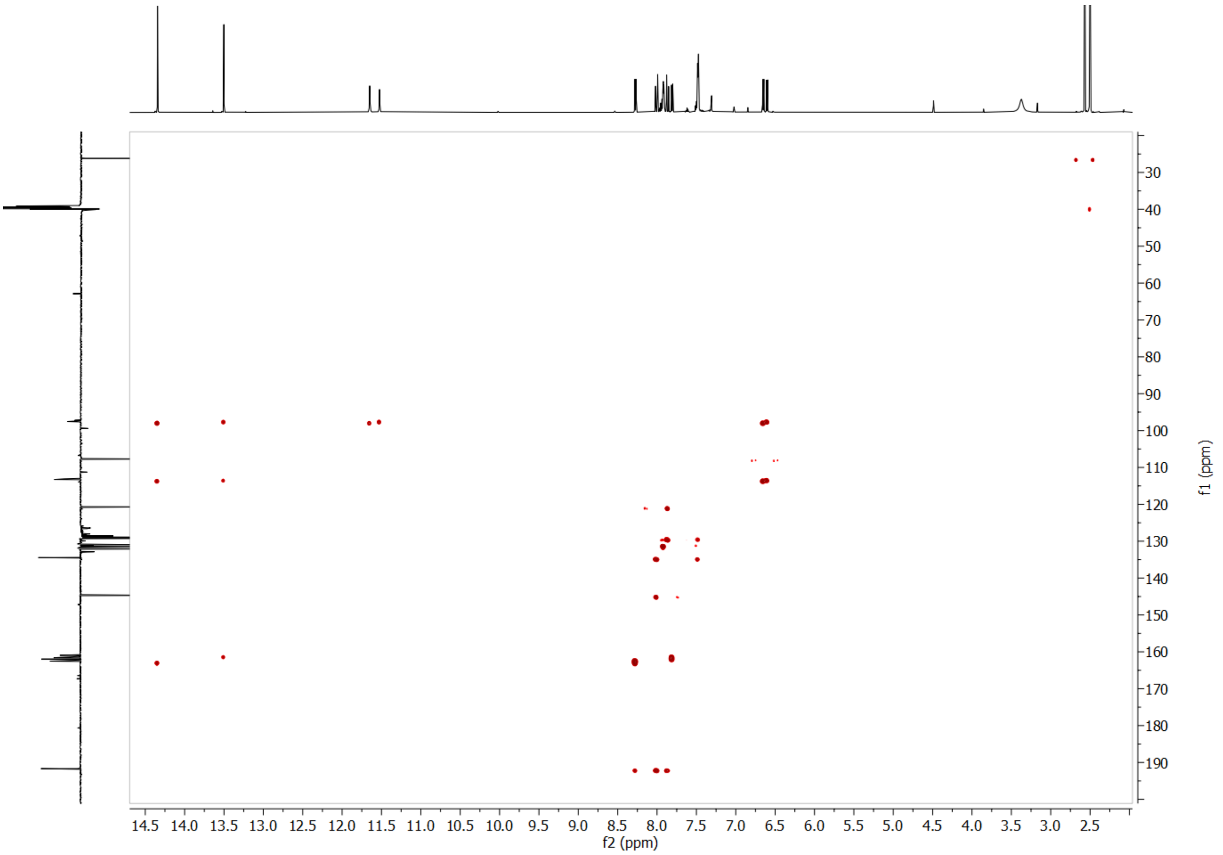

HMBC NMR spectrum of **3-bromo-2,4-dihydroxychalcone** in DMSO-d_6 at 600 MHz.

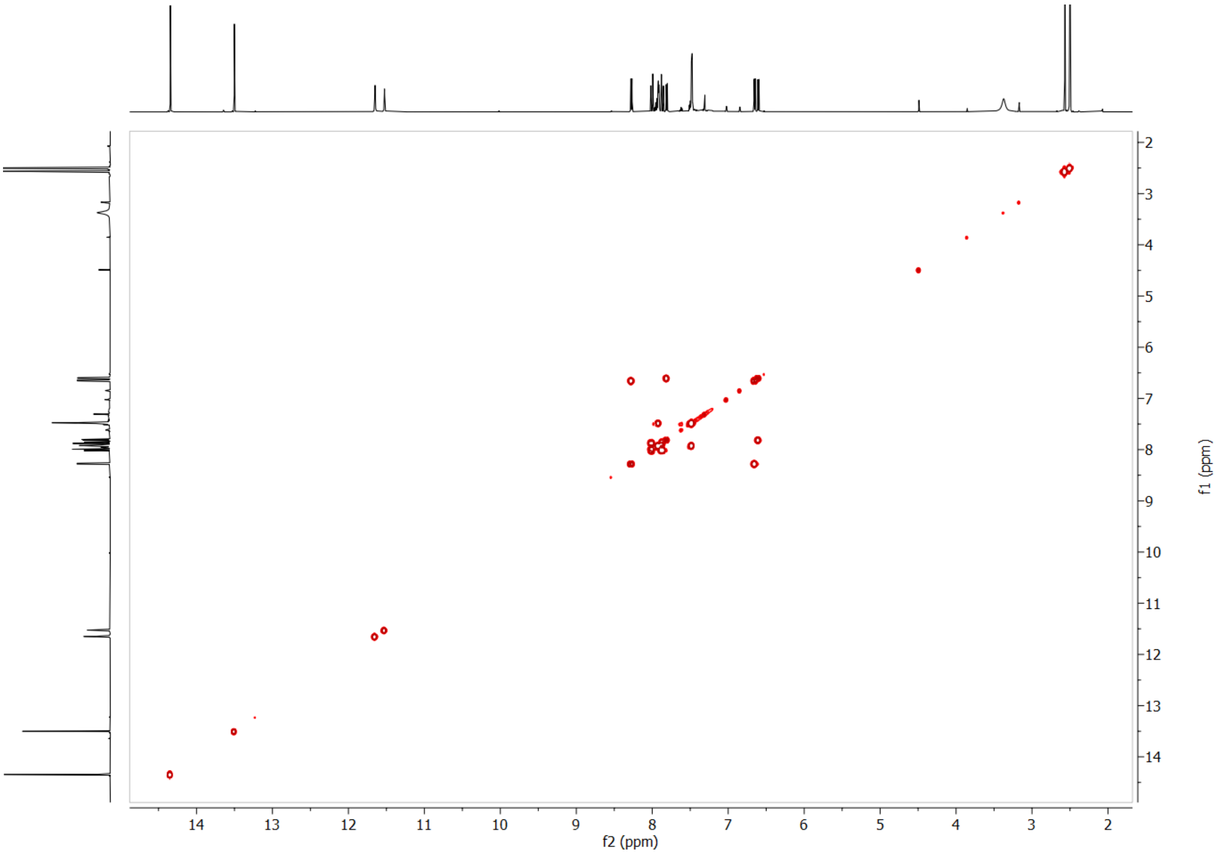

COSY NMR spectrum of **3-bromo-2,4-dihydroxychalcone** in DMSO-d_6 at 600 MHz.

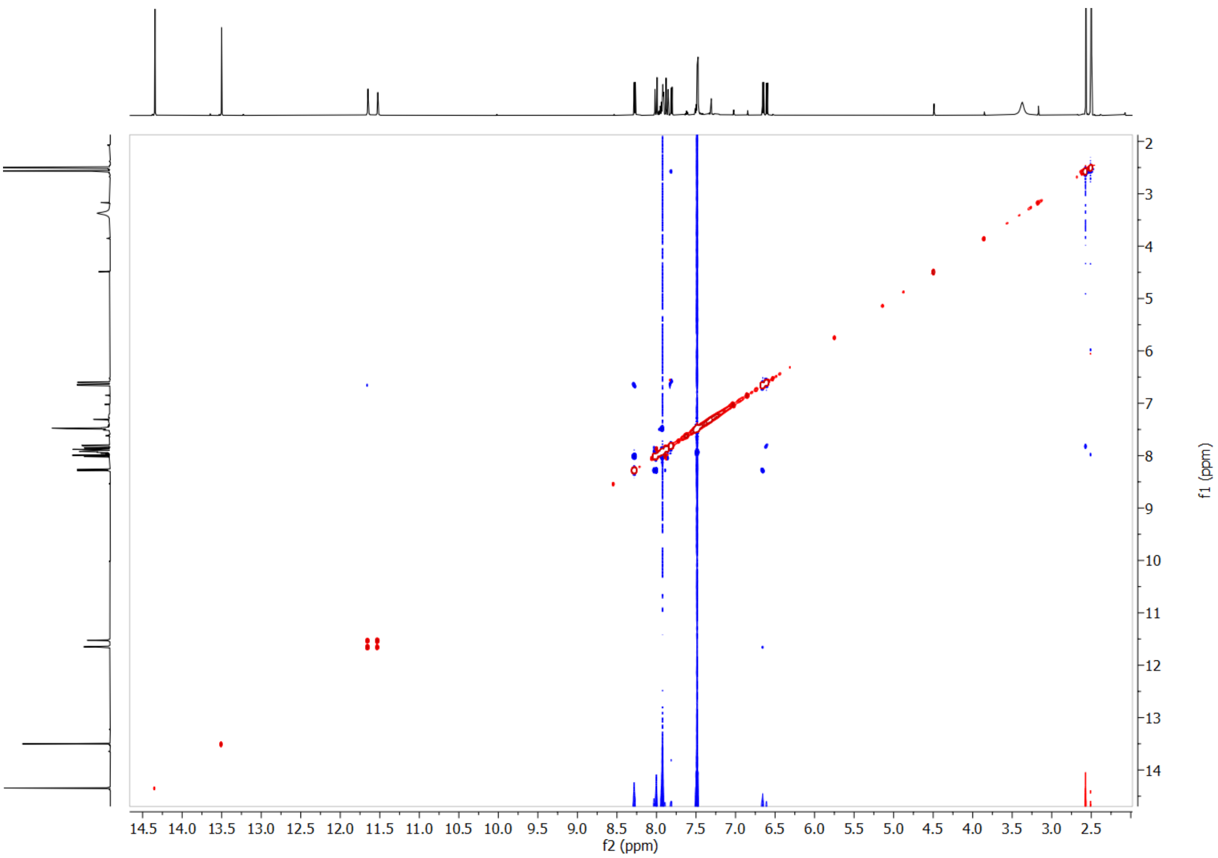

ROESY NMR spectrum of **3-bromo-2,4-dihydroxychalcone** in DMSO-d_6 at 600 MHz.

25. 5-bromo-2,4-dihydroxychalcone:

Experimental:

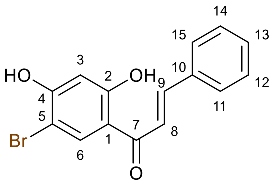

5-bromo-2,4-dihydroxychalcone (**25**) light brown solid; ^{1}H NMR (DMSO-d_6, 600 MHz) δ 13.23 (1H, s), 11.62 (1H, s), 8.53 (1H, s), 8.04 (1H, d, J = 15.4 Hz), 7.96 – 7.92 (2H, m), 7.81 (1H, d, J = 15.4 Hz), 7.46 (3H, t, J = 3.1 Hz), 6.52 (1H, d, J = 1.1 Hz); ^{13}C NMR (DMSO-d_6, 151 MHz) δ 191.1, 164.3, 161.2, 144.5, 135.0, 134.6, 130.8, 129.3, 128.9, 121.3, 114.5, 103.5, 100.6; HRESIMS *m/z* 318.9963 [M+H]$^+$ (calcd for $C_{15}H_{12}BrO_3^+$ 318.9964), *m/z* 316.9819 [M-H]$^-$ (calcd for $C_{15}H_{10}BrO_3^-$ 316.9819).

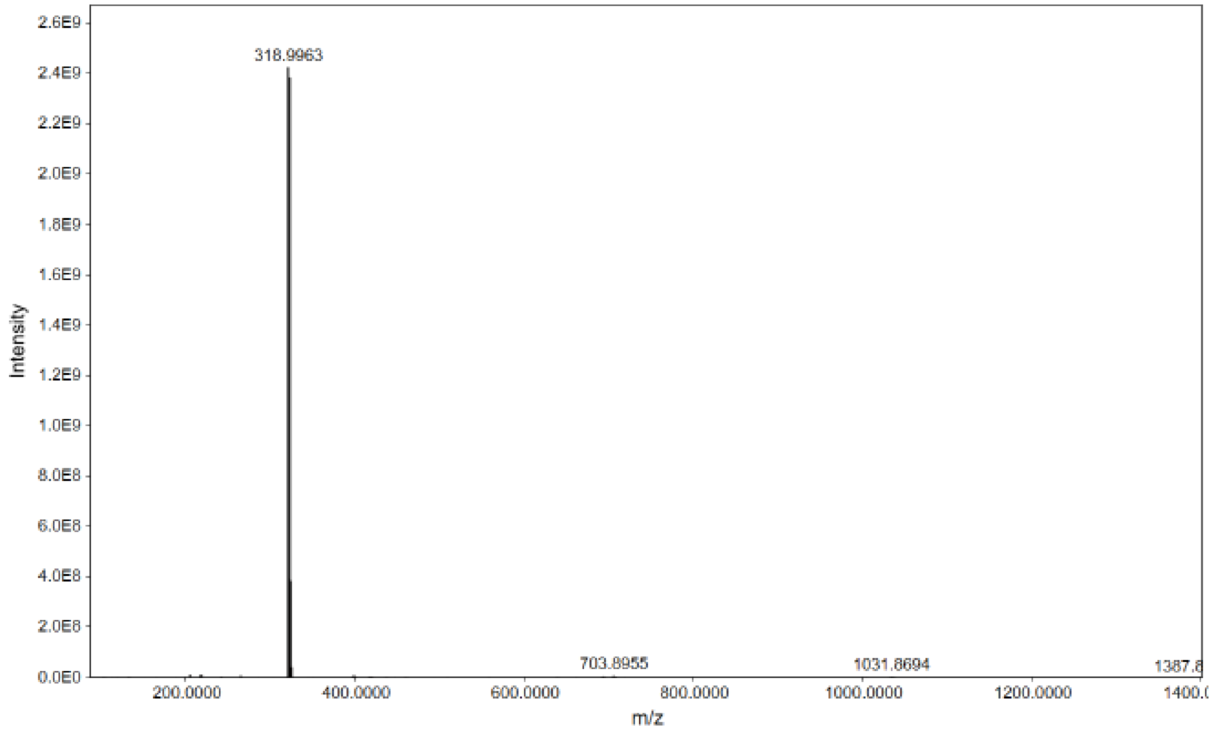

HRESIMS+ spectrum of **5-bromo-2,4-dihydroxychalcone** in MeOH.

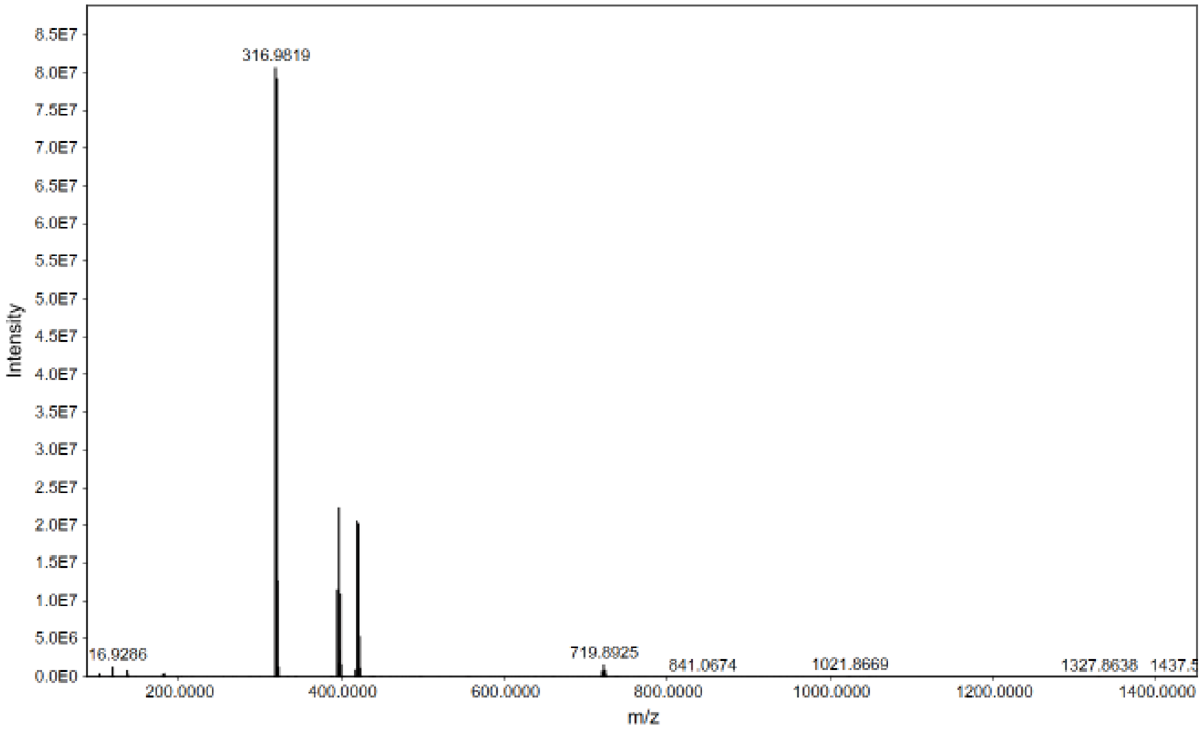

HRESIMS- spectrum of **5-bromo-2,4-dihydroxychalcone** in MeOH.

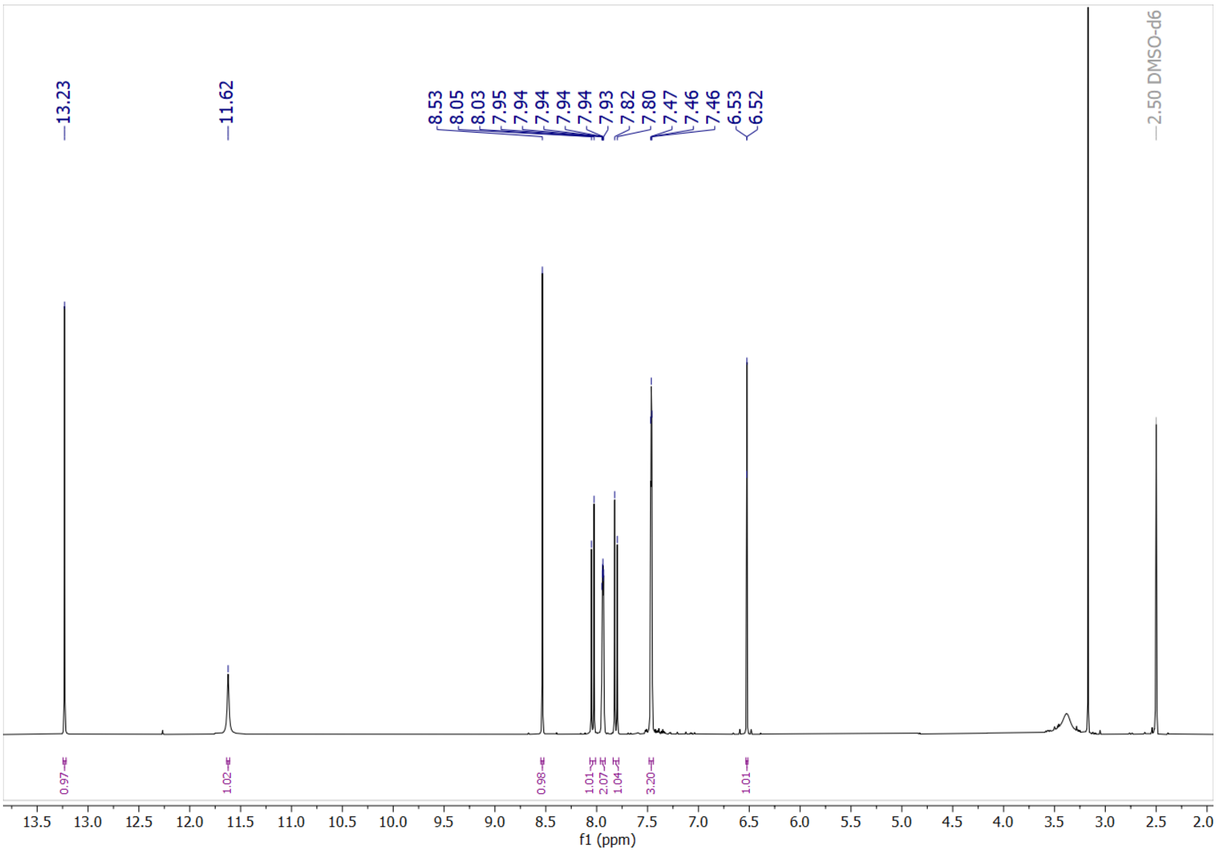

^{1}H NMR spectrum of **5-bromo-2,4-dihydroxychalcone** in DMSO-d_6 at 600 MHz.

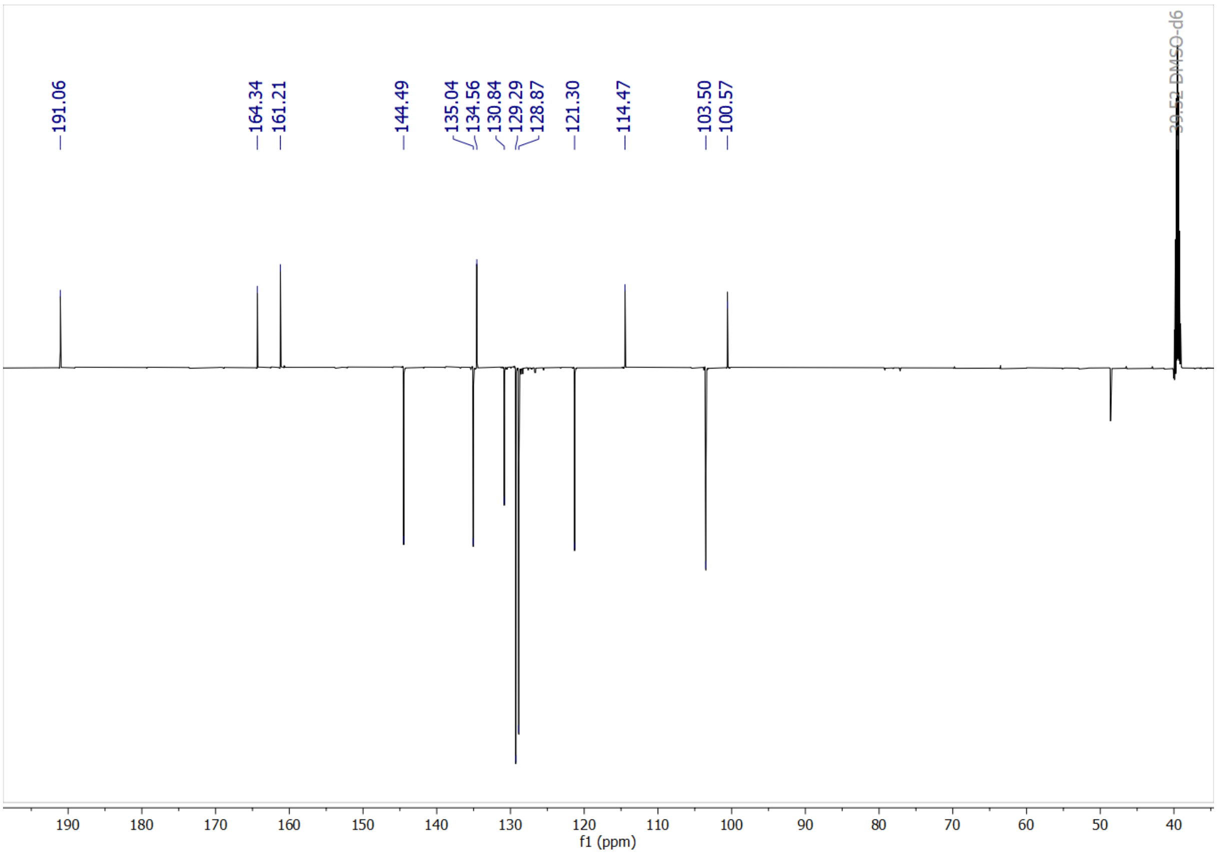

^{13}C DEPTQ NMR spectrum of **5-bromo-2,4-dihydroxychalcone** in DMSO-d_6 at 151 MHz.

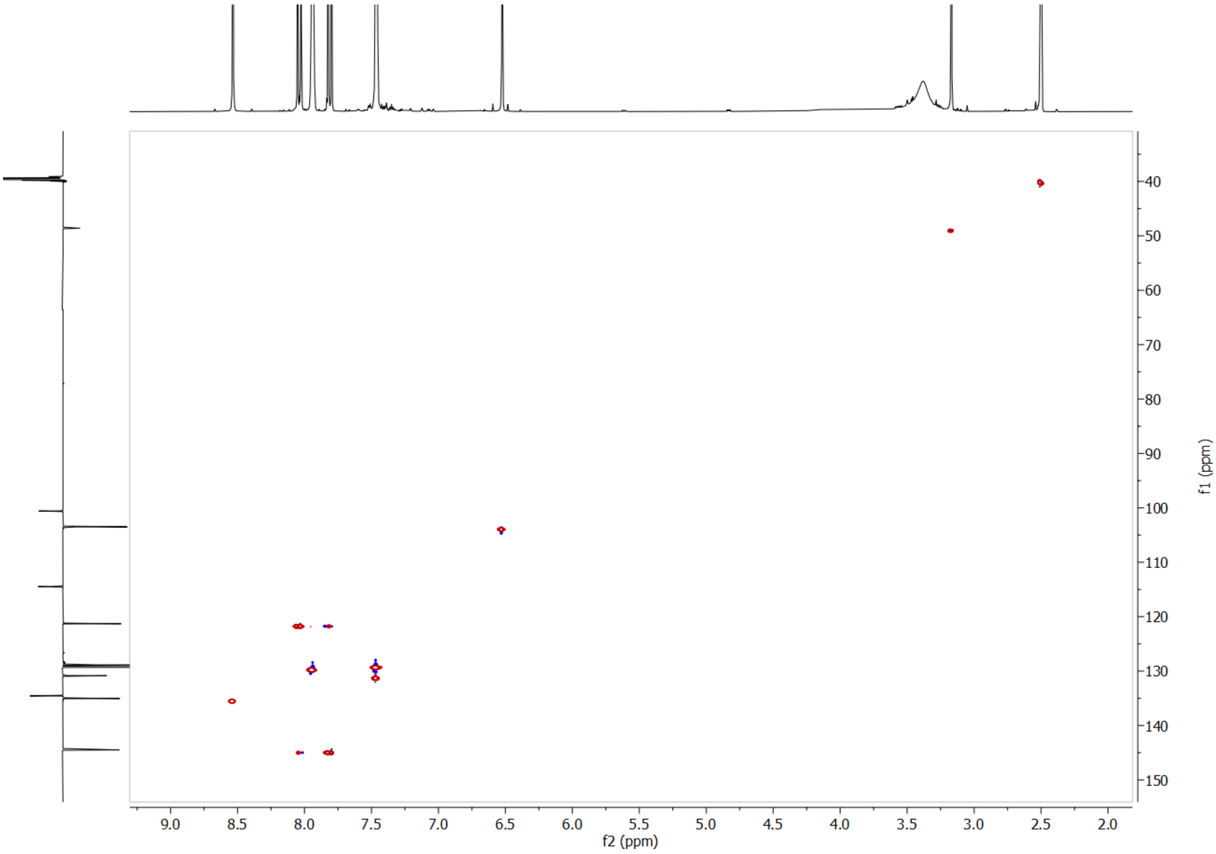

HSQC NMR spectrum of **5-bromo-2,4-dihydroxychalcone** in DMSO-d_6 at 600 MHz.

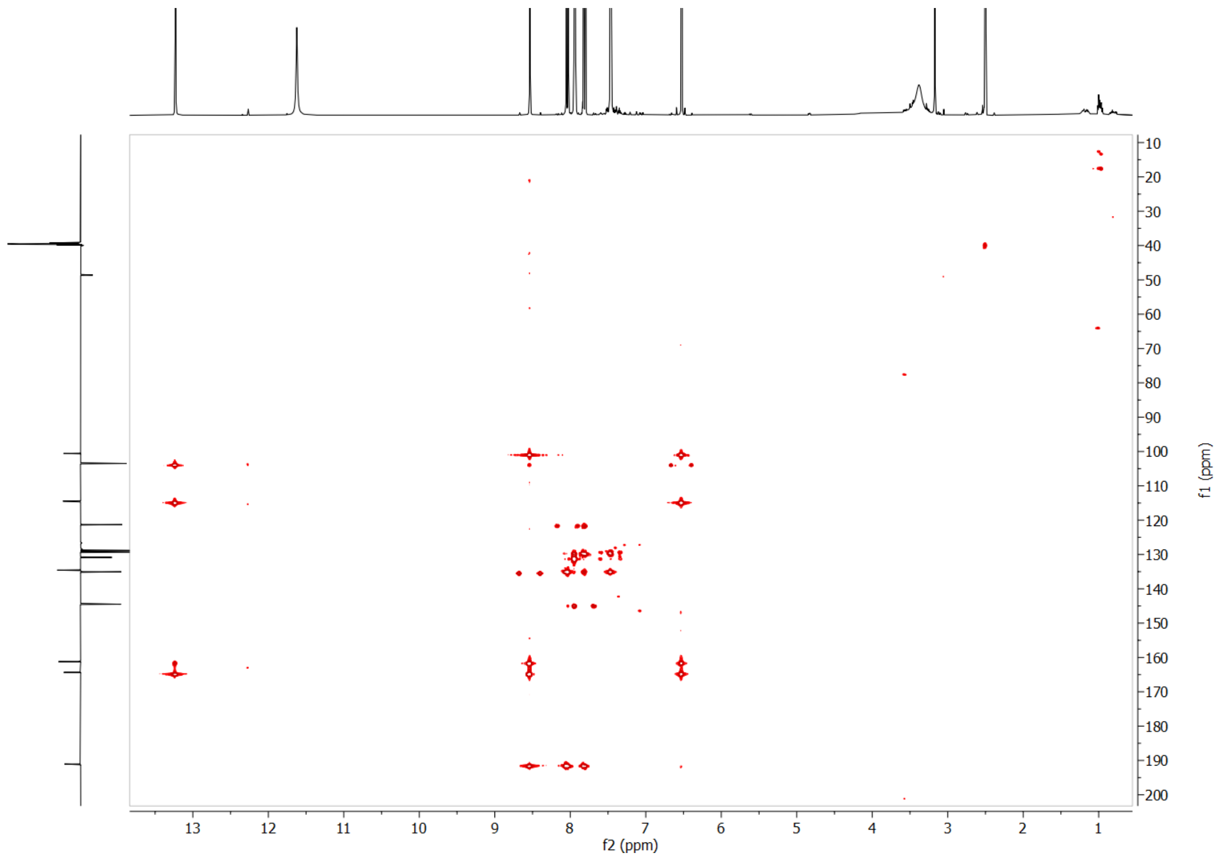

HMBC NMR spectrum of **5-bromo-2,4-dihydroxychalcone** in DMSO-d_6 at 600 MHz.

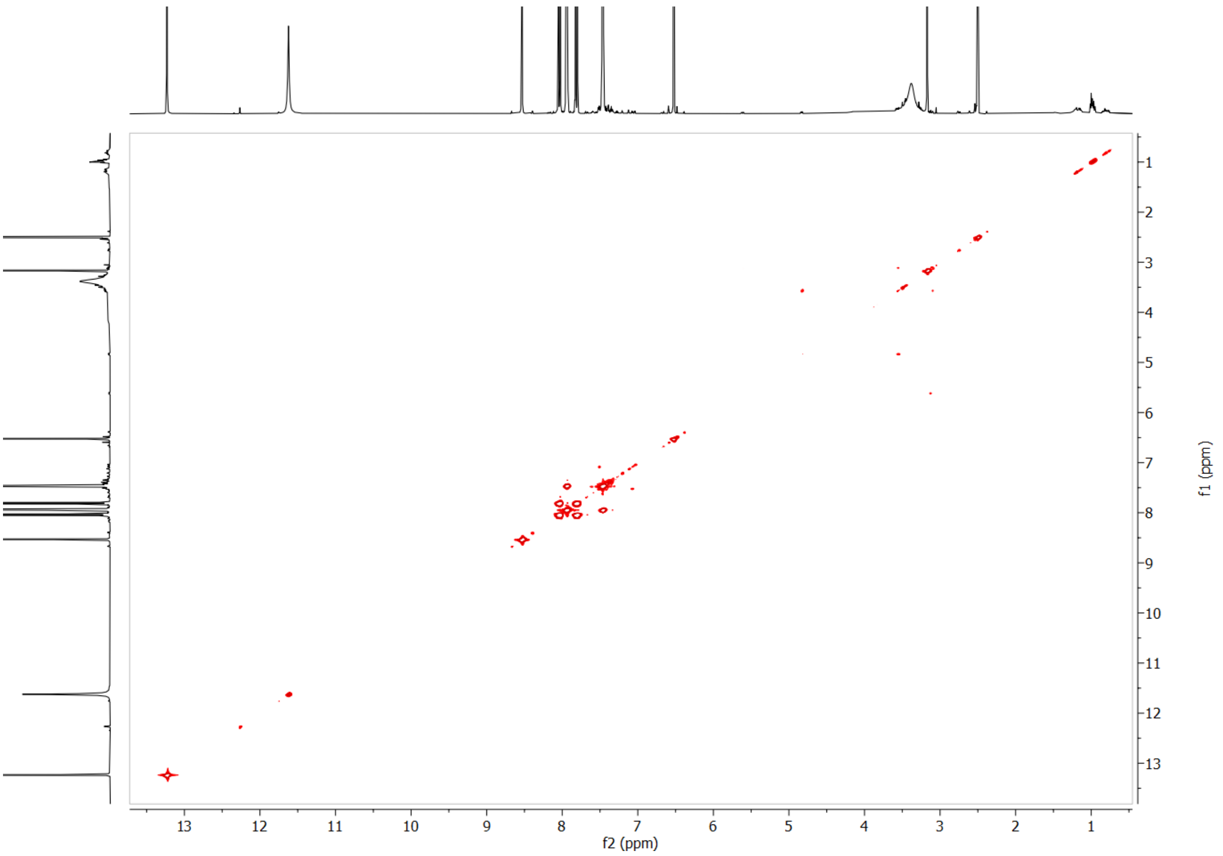

COSY NMR spectrum of **5-bromo-2,4-dihydroxychalcone** in DMSO-d_6 at 600 MHz.

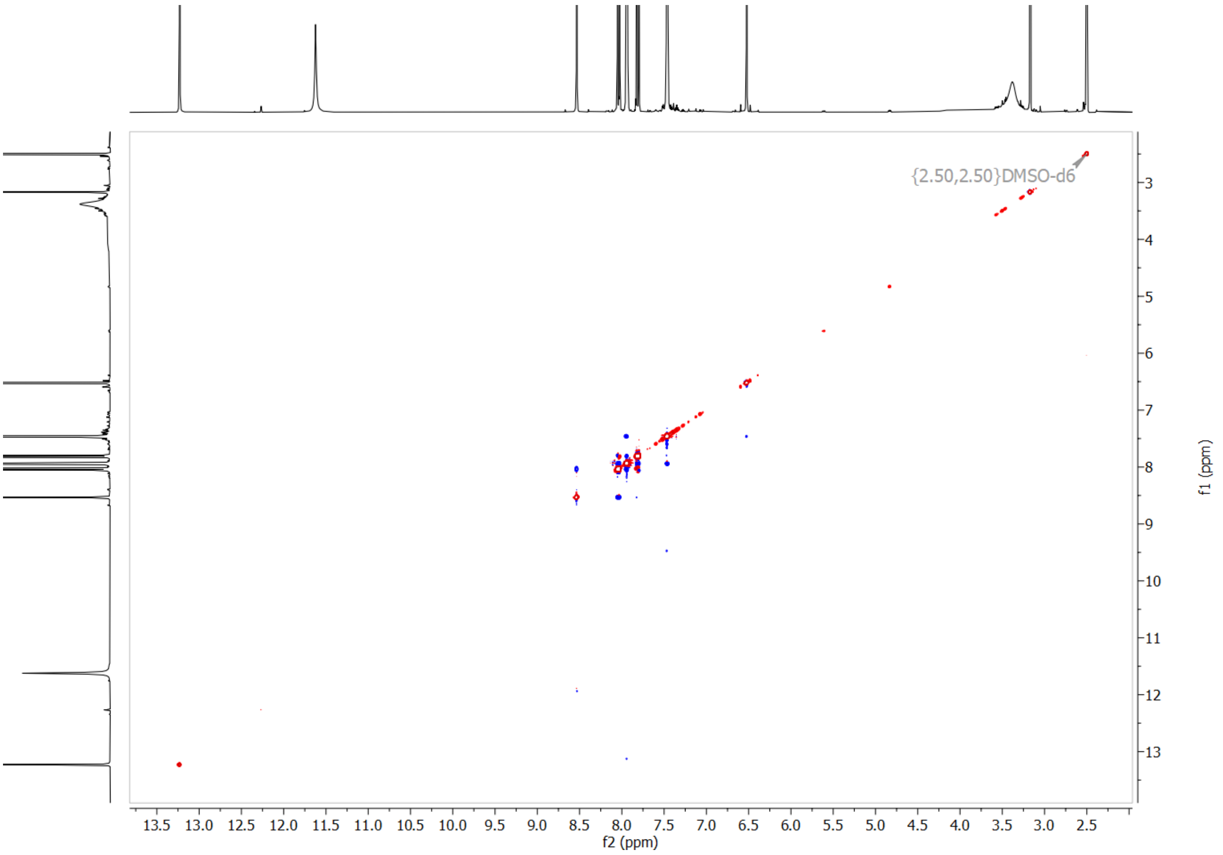

ROESY NMR spectrum of **5-bromo-2,4-dihydroxychalcone** in DMSO-d_6 at 600 MHz.

26. (E)-1-(5-chloro-2,4-dihydroxyphenyl)-3-(naphthalen-1-yl)prop-2-en-1-one:

Experimental:

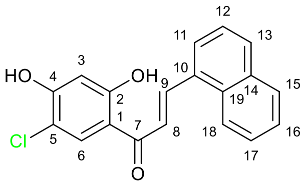

(E)-1-(5-chloro-2,4-dihydroxyphenyl)-3-(naphthalen-1-yl)prop-2-en-1-one (**26**) yellow solid; ^{1}H NMR (DMSO-d_6, 600 MHz) δ 13.19 (1H, s), 8.64 (1H, dd, J = 15.2, 3.5 Hz), 8.43 (1H, d, J = 3.9 Hz), 8.35 (1H, d, J = 7.1 Hz), 8.30 (1H, d, J = 8.4 Hz), 8.13 (1H, dd, J = 15.2, 4.3 Hz), 8.08 (1H, d, J = 8.0 Hz), 8.01 (1H, d, J = 8.0 Hz), 7.69 – 7.56 (3H, m), 6.54 (1H, d, J = 2.4 Hz); ^{13}C NMR (DMSO-d_6, 151 MHz) δ 190.9, 163.8, 160.4, 140.0, 133.4, 132.1, 131.3, 131.2, 131.0, 128.9, 127.4, 126.3, 126.2, 125.7, 123.7, 122.9, 113.8, 112.0, 103.8; HRESIMS *m/z* 325.0626 [M+H]$^+$ (calcd for $C_{19}H_{14}ClO_3^+$ 325.0626), *m/z* 323.0477 [M-H]$^-$ (calcd for $C_{29}H_{12}ClO_3^-$ 323.0480).

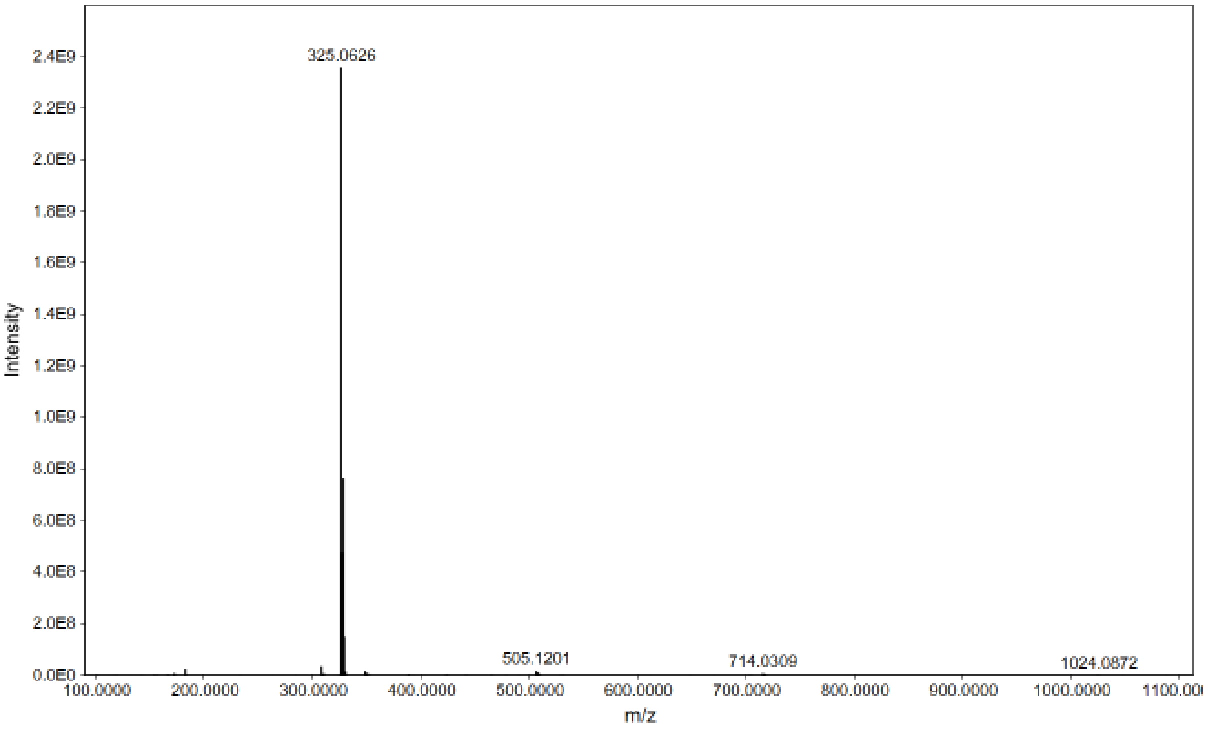

HRESIMS+ spectrum of **(E)-1-(5-chloro-2,4-dihydroxyphenyl)-3-(naphthalen-1-yl)prop-2-en-1-one** in MeOH.

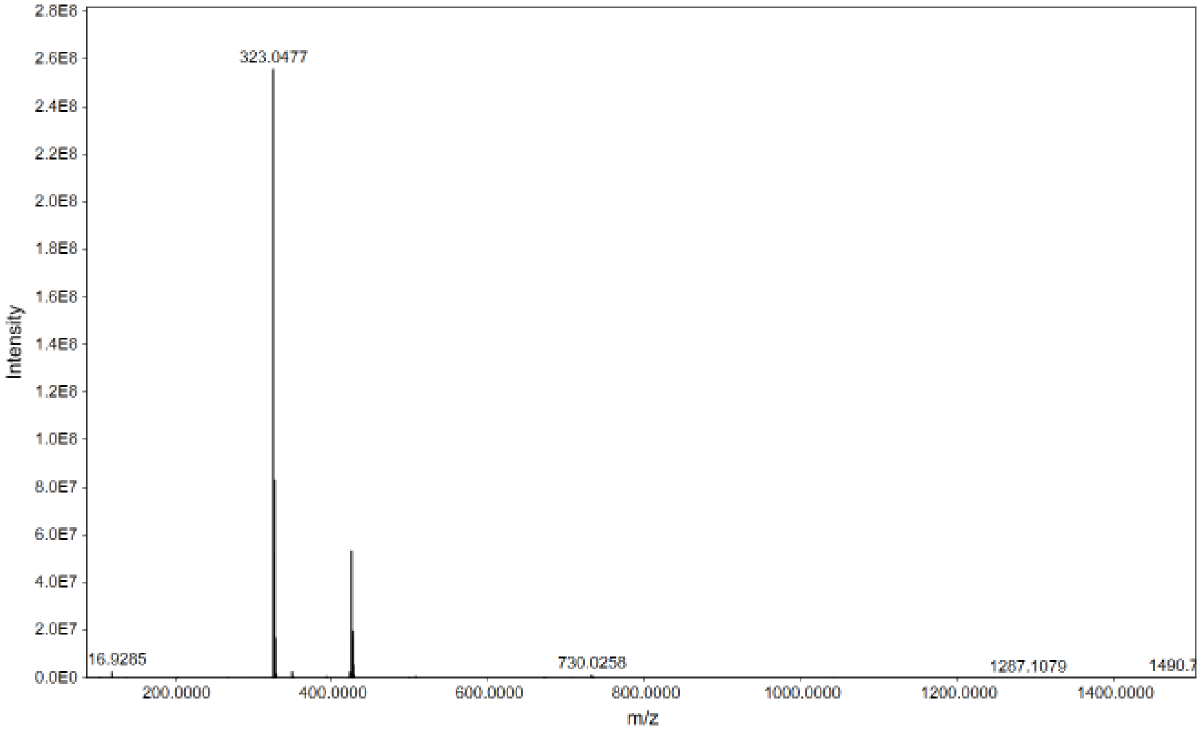

HRESIMS- spectrum of **(E)-1-(5-chloro-2,4-dihydroxyphenyl)-3-(naphthalen-1-yl)prop-2-en-1-one** in MeOH.

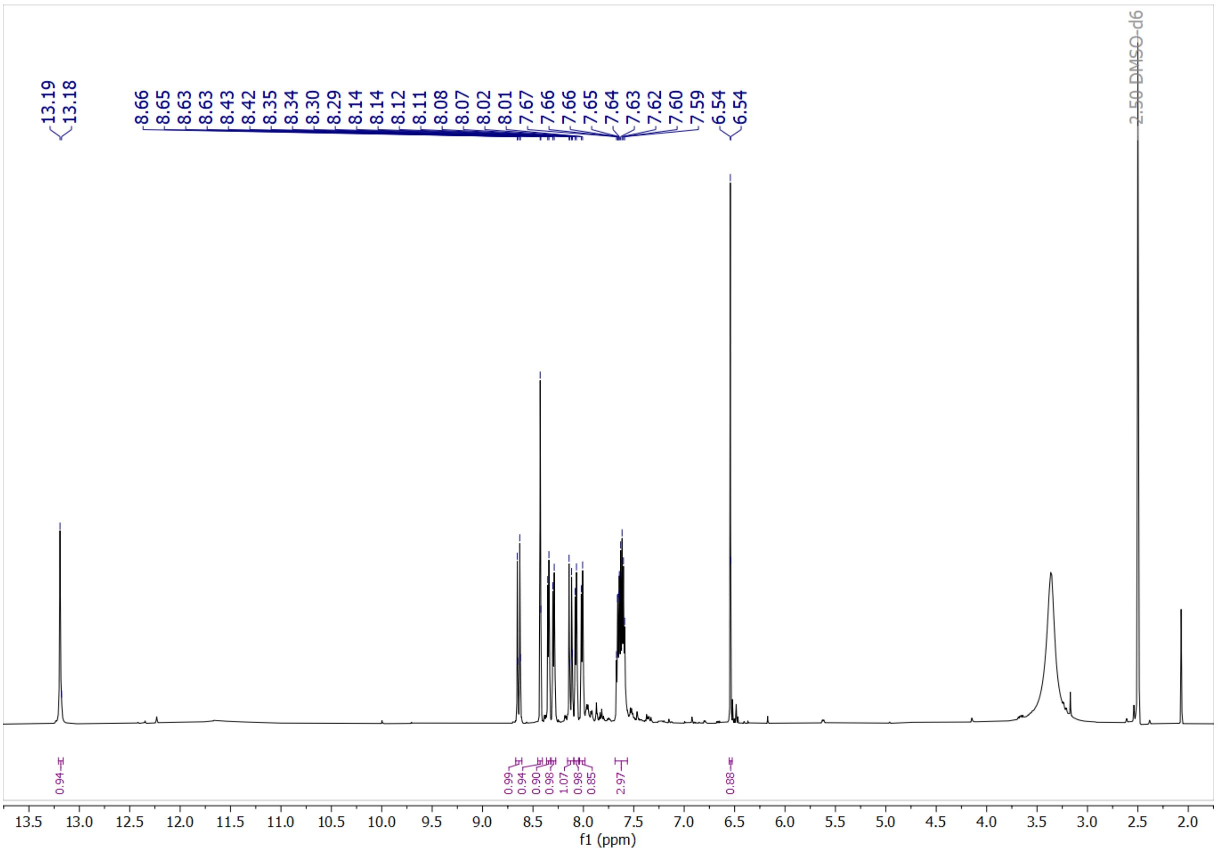

^{1}H NMR spectrum of **(E)-1-(5-chloro-2,4-dihydroxyphenyl)-3-(naphthalen-1-yl)prop-2-en-1-one** in DMSO-d_6 at 600 MHz.

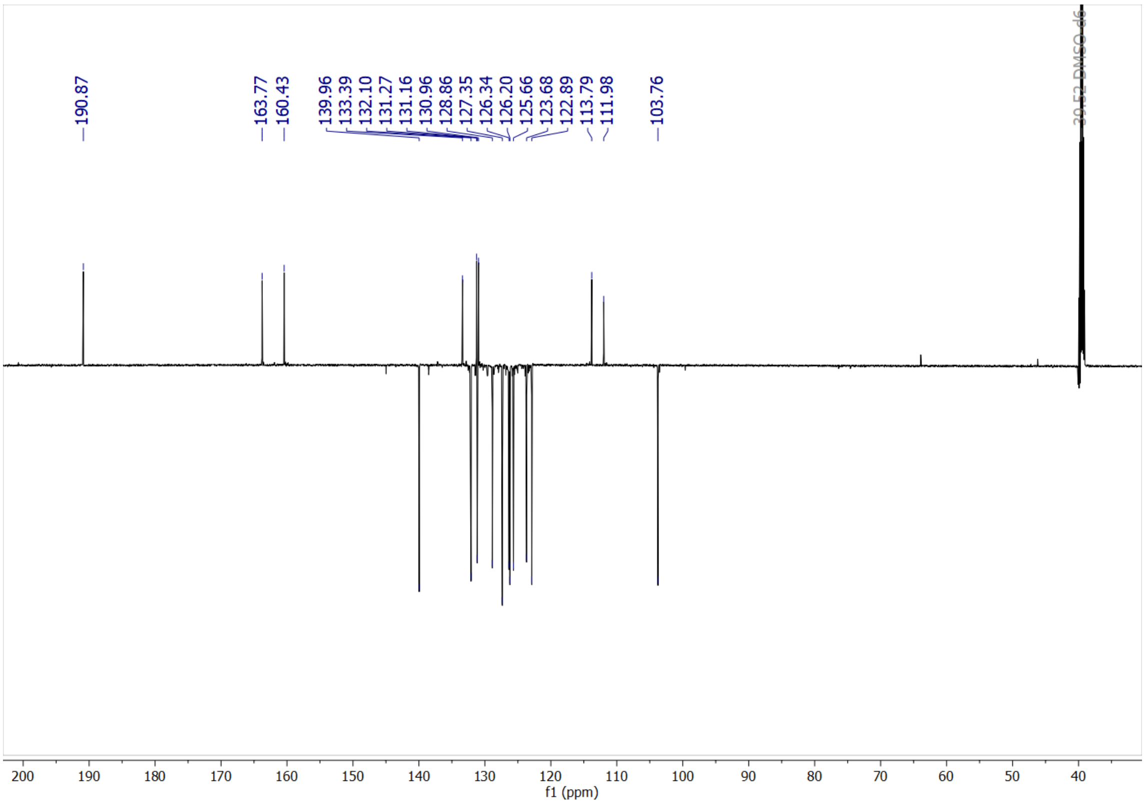

^{13}C DEPTQ NMR spectrum of **(E)-1-(5-chloro-2,4-dihydroxyphenyl)-3-(naphthalen-1-yl)prop-2-en-1-one** in DMSO-d_6 at 151 MHz.

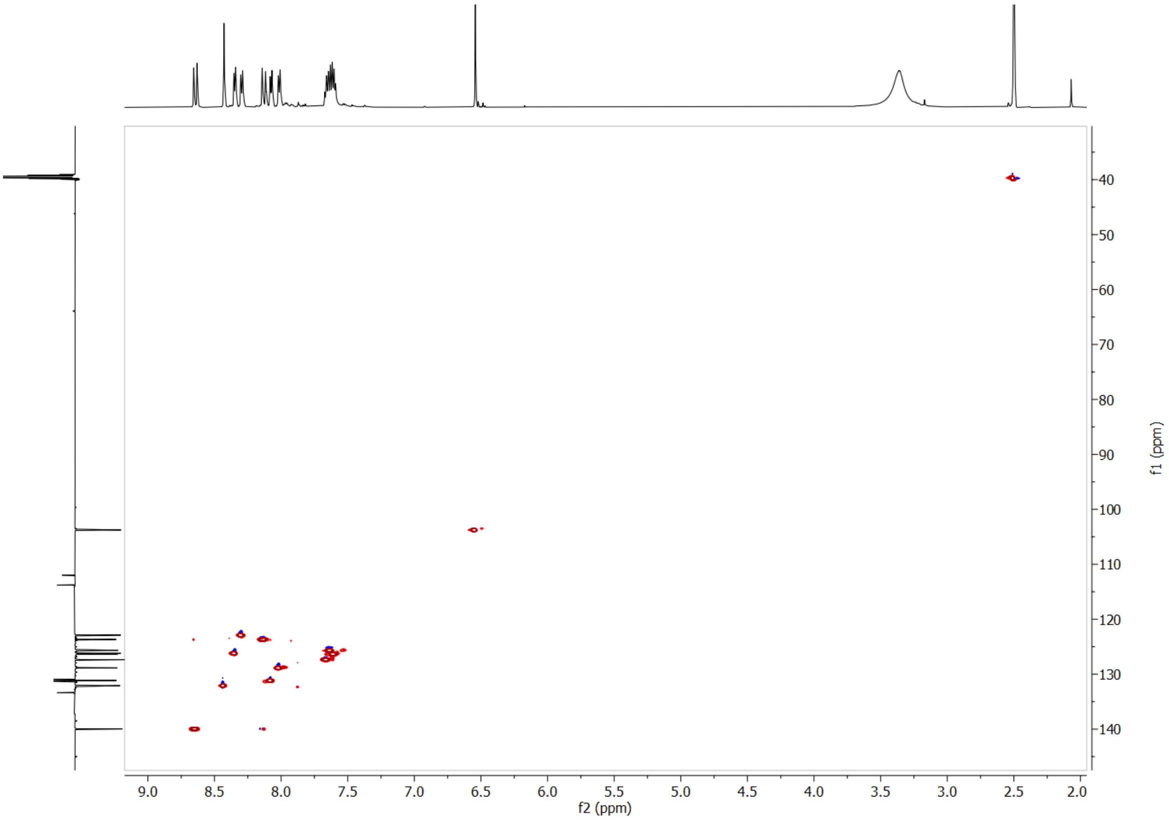

HSQC NMR spectrum of **(E)-1-(5-chloro-2,4-dihydroxyphenyl)-3-(naphthalen-1-yl)prop-2-en-1-one** in DMSO-d_6 at 600 MHz.

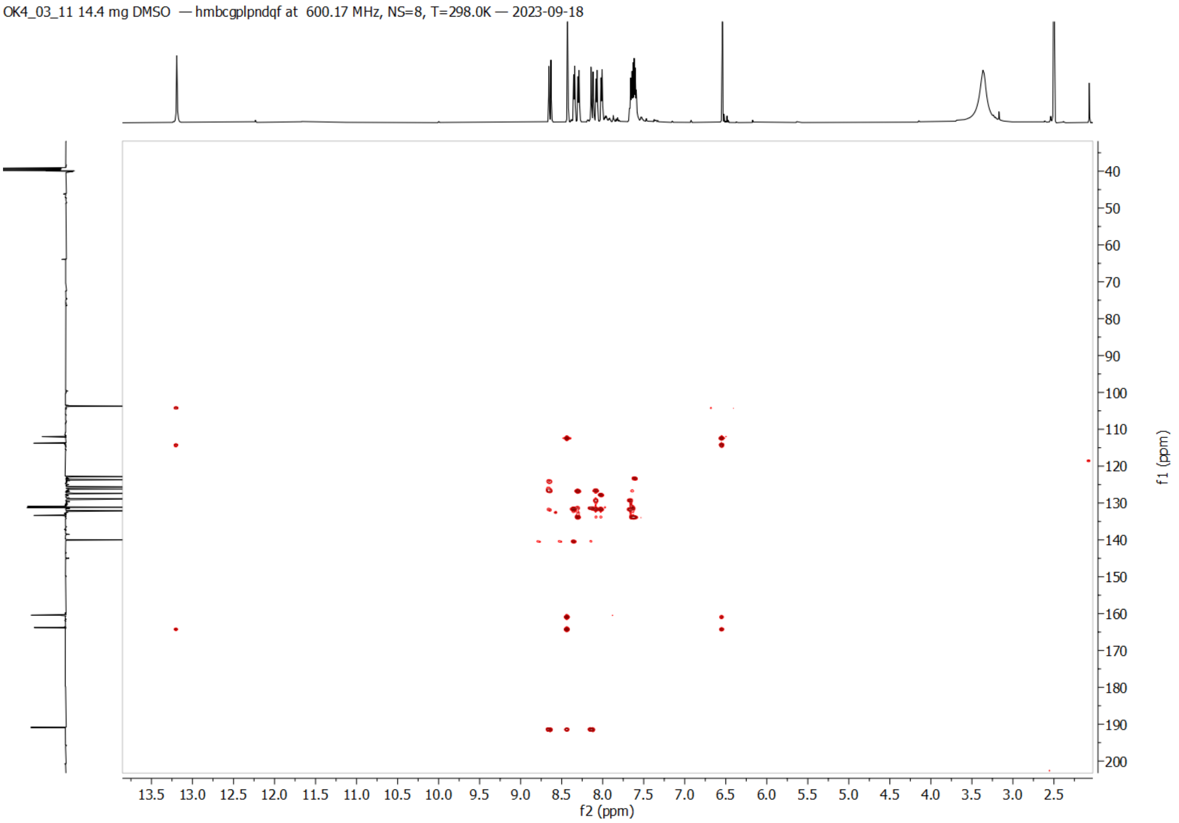

HMBC NMR spectrum of **(E)-1-(5-chloro-2,4-dihydroxyphenyl)-3-(naphthalen-1-yl)prop-2-en-1-one** in DMSO-d_6 at 600 MHz.

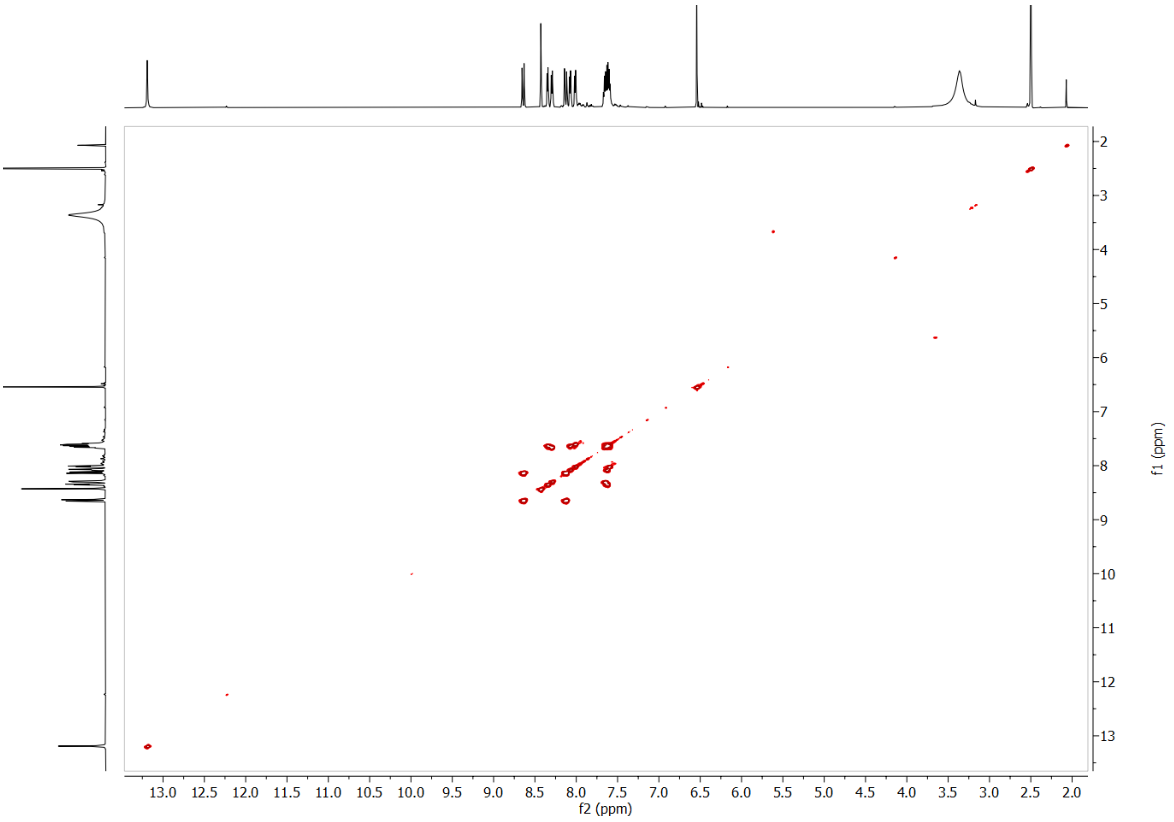

COSY NMR spectrum of **(E)-1-(5-chloro-2,4-dihydroxyphenyl)-3-(naphthalen-1-yl)prop-2-en-1-one** in DMSO-d_6 at 600 MHz.

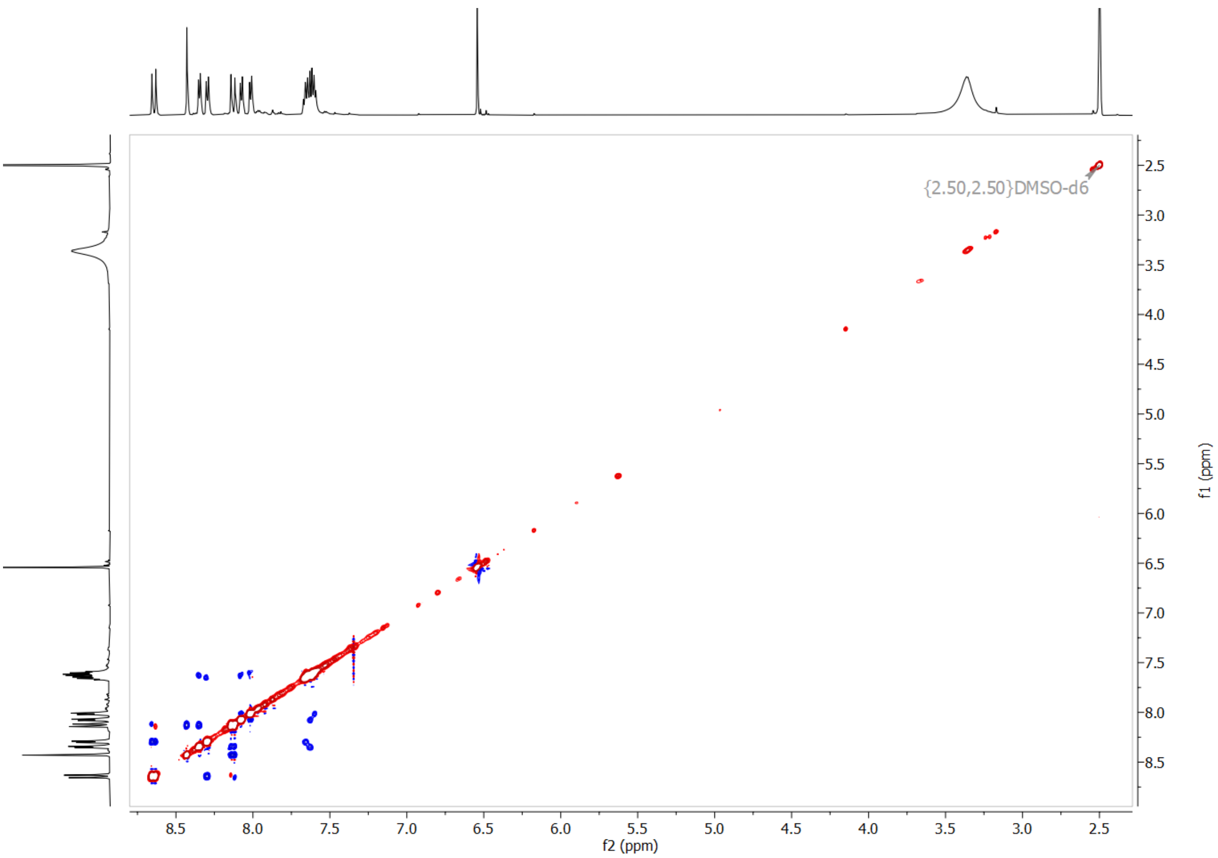

ROESY NMR spectrum of **(E)-1-(5-chloro-2,4-dihydroxyphenyl)-3-(naphthalen-1-yl)prop-2-en-1-one** in DMSO-d_6 at 600 MHz.

27. (E)-3-(anthracen-9-yl)-1-(5-chloro-2,4-dihydroxyphenyl)prop-2-en-1-one:

Experimental:

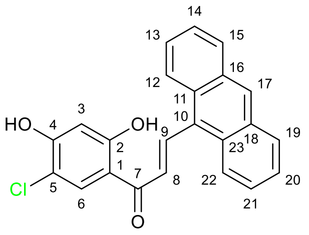

(E)-3-(anthracen-9-yl)-1-(5-chloro-2,4-dihydroxyphenyl)prop-2-en-1-one (**27**) yellow solid; ^{1}H NMR (DMSO-d_6, 600 MHz) δ 13.28 (1H, s), 8.64 (1H, s), 8.35 (2H, dd, J = 8.7, 1.3 Hz), 8.26 (1H, d, J = 15.8 Hz), 8.16 – 8.11 (3H, m), 7.94 (1H, dd, J = 14.6, 11.1 Hz), 7.66 (1H, d, J = 14.7 Hz), 7.58 (4H, dddd, J = 15.6, 7.8, 6.5, 1.4 Hz), 7.08 (1H, dd, J = 15.7, 11.1 Hz), 6.46 (1H, s); ^{13}C NMR (DMSO-d_6, 151 MHz) δ 189.4, 163.5, 139.7, 131.9, 131.4, 130.9, 129.9, 128.9, 128.2, 126.8, 125.6, 125.2, 118.1, 113.6, 103.8; HRESIMS *m/z* 375.0780 $[M+H]^+$ (calcd for $C_{23}H_{16}ClO_3^+$ 375.0782), *m/z* 373.0635 $[M-H]^-$ (calcd for $C_{23}H_{14}ClO_3^-$ 373.0637).

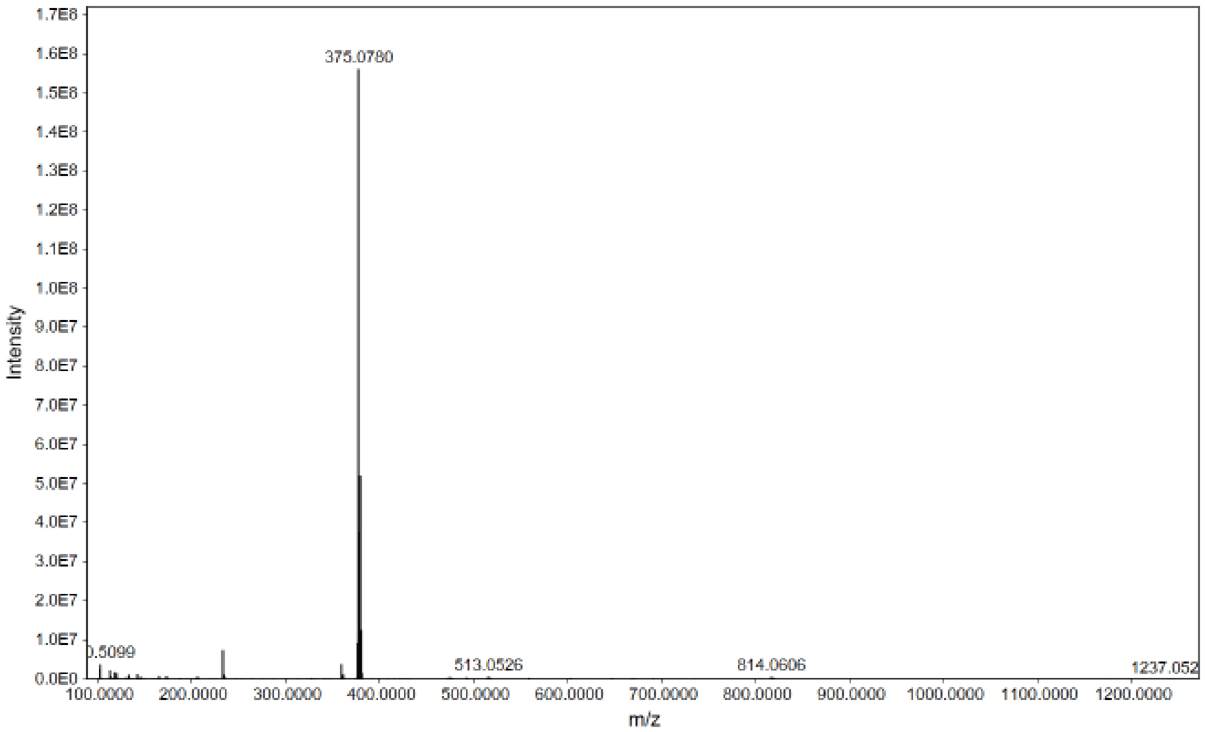

HRESIMS+ spectrum of **(E)-3-(anthracen-9-yl)-1-(5-chloro-2,4-dihydroxyphenyl)prop-2-en-1-one** in MeOH.

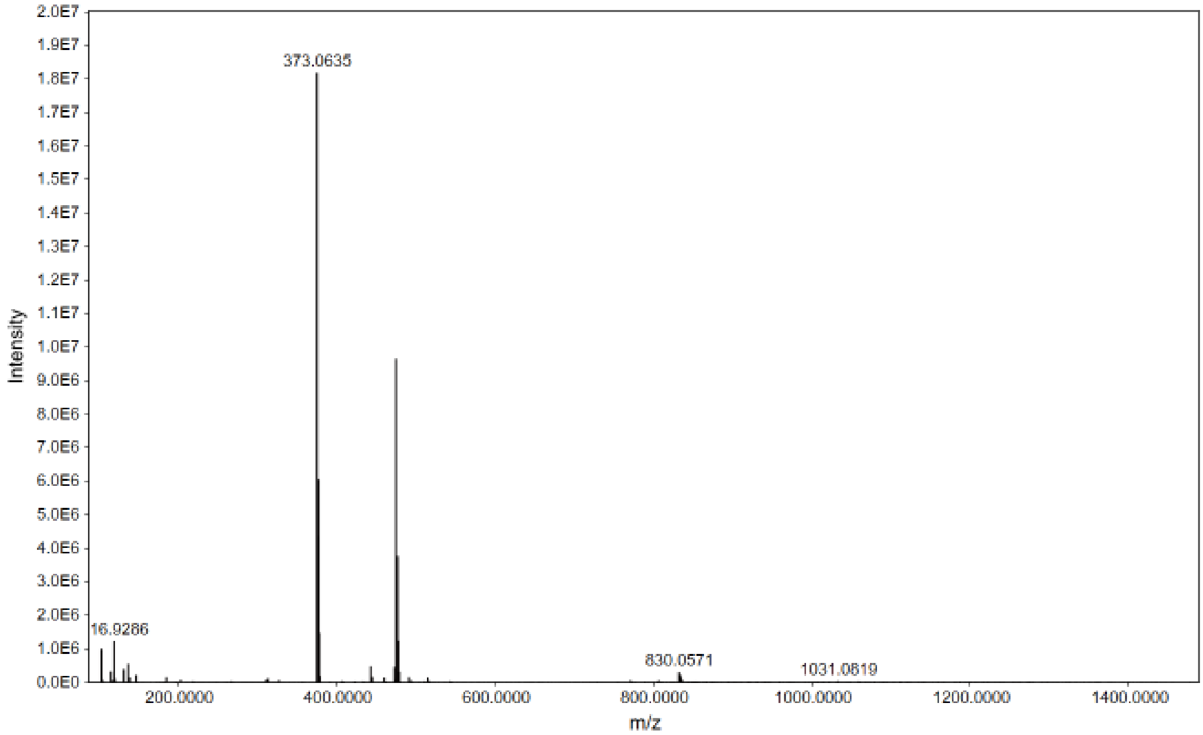

HRESIMS- spectrum of **(E)-3-(anthracen-9-yl)-1-(5-chloro-2,4-dihydroxyphenyl)prop-2-en-1-one** in MeOH.

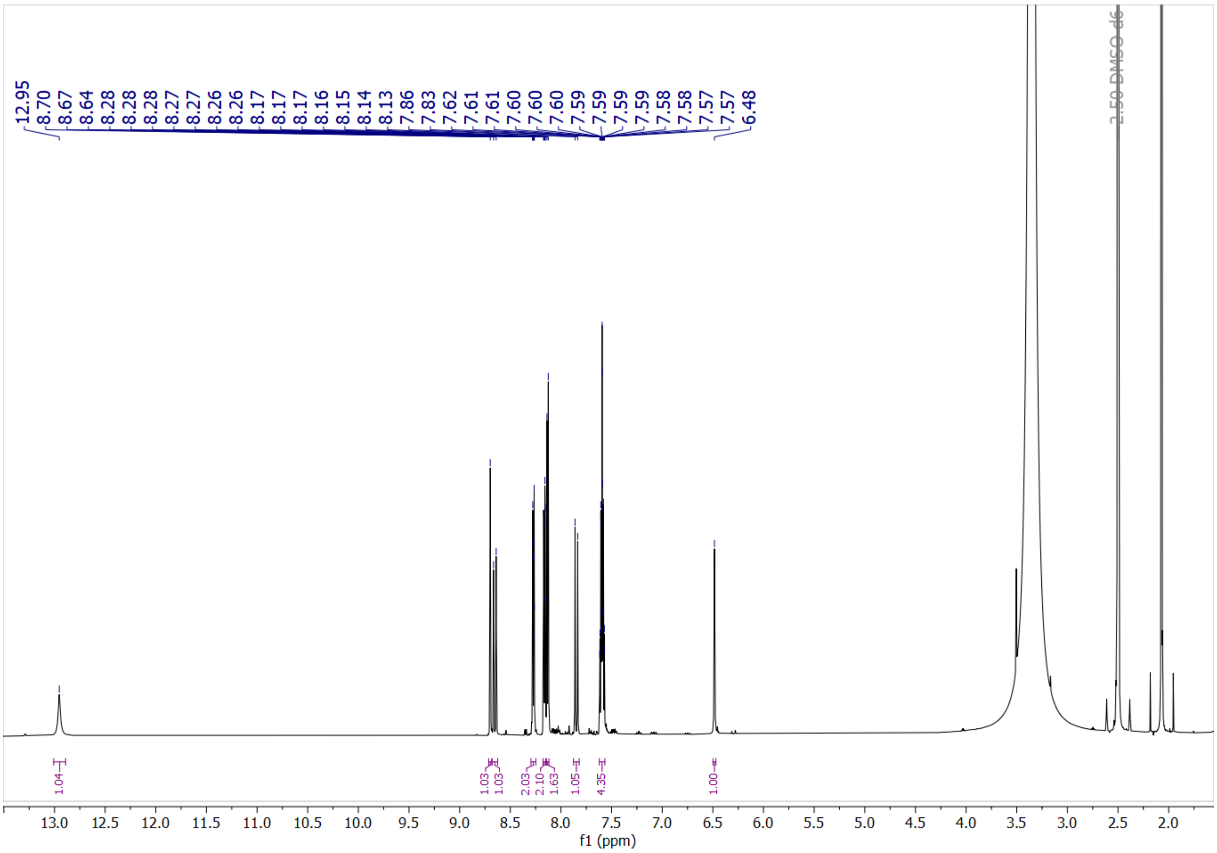

^{1}H NMR spectrum of **(E)-3-(anthracen-9-yl)-1-(5-chloro-2,4-dihydroxyphenyl)prop-2-en-1-one** in DMSO-d_6 at 600 MHz.

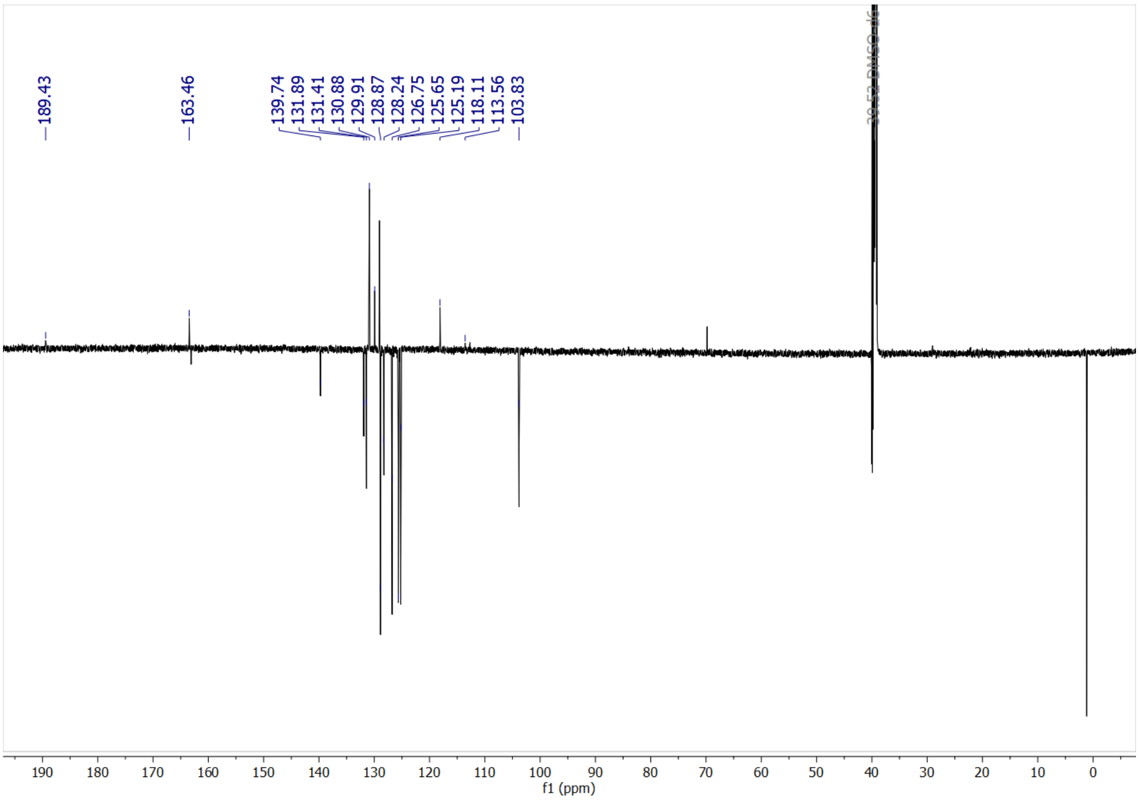

^{13}C DEPTQ NMR spectrum of **(E)-3-(anthracen-9-yl)-1-(5-chloro-2,4-dihydroxyphenyl)prop-2-en-1-one** in DMSO-d_6 at 151 MHz.

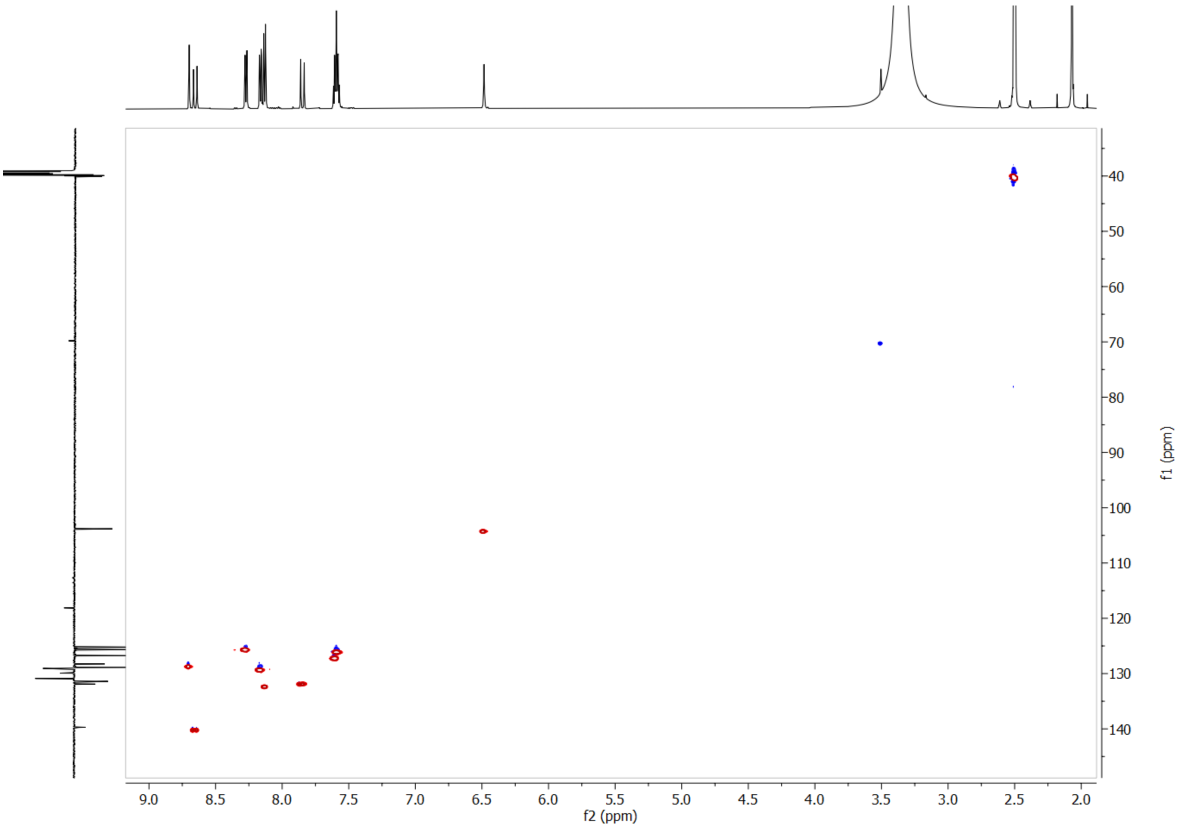

HSQC NMR spectrum of **(E)-3-(anthracen-9-yl)-1-(5-chloro-2,4-dihydroxyphenyl)prop-2-en-1-one** in DMSO-d_6 at 600 MHz.

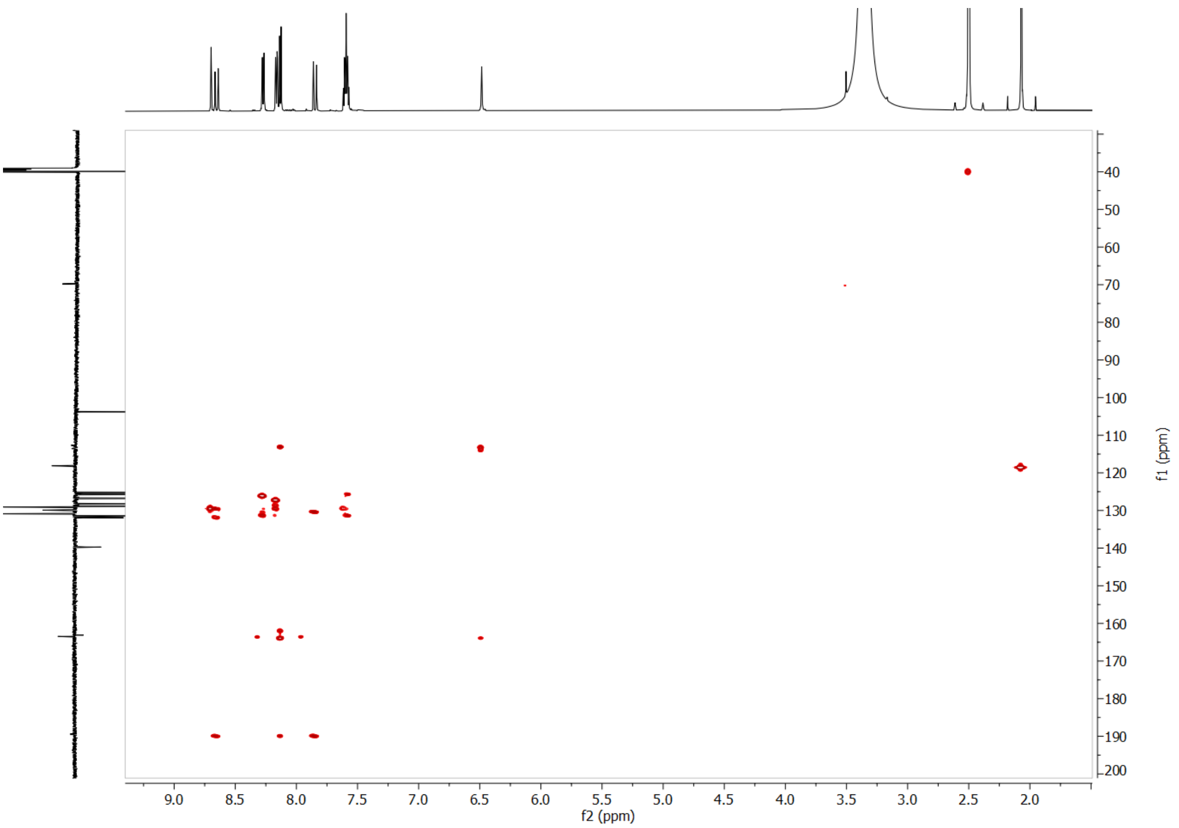

HMBC NMR spectrum of **(E)-3-(anthracen-9-yl)-1-(5-chloro-2,4-dihydroxyphenyl)prop-2-en-1-one** in DMSO-d_6 at 600 MHz.

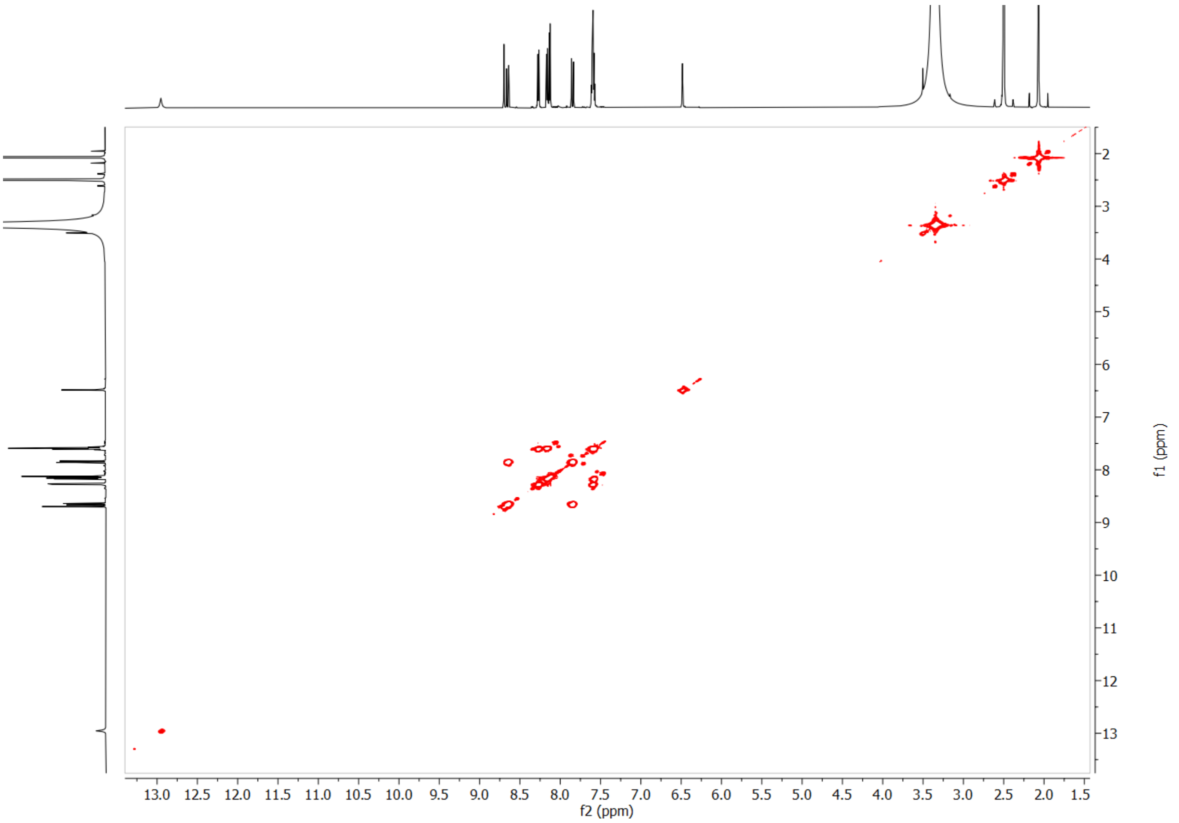

COSY NMR spectrum of **(E)-3-(anthracen-9-yl)-1-(5-chloro-2,4-dihydroxyphenyl)prop-2-en-1-one** in DMSO-d_6 at 600 MHz.

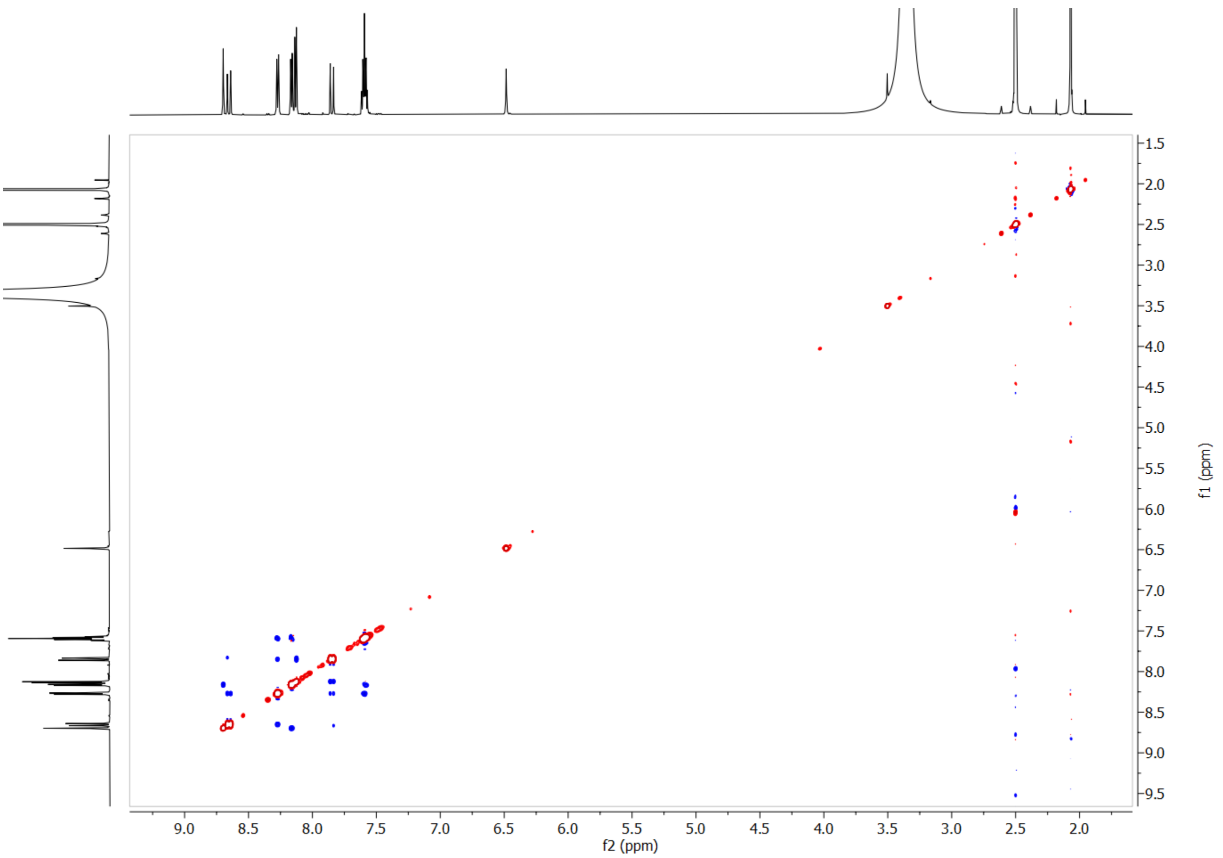

ROESY NMR spectrum of **(E)-3-(anthracen-9-yl)-1-(5-chloro-2,4-dihydroxyphenyl)prop-2-en-1-one** in DMSO-d_6 at 600 MHz.

28. 1-(3-chloro-4,6-dihydroxy-2-(6-phenylhexyl)phenyl)ethan-1-one:

Experimental:

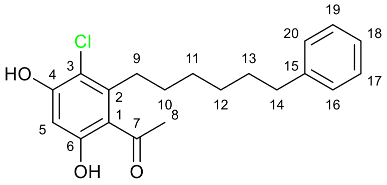

1-(3-chloro-4,6-dihydroxy-2-(6-phenylhexyl)phenyl)ethan-1-one (**28**); ^{1}H NMR (DMSO-d_6, 600 MHz) δ 10.23 (1H, s), 9.98 (1H, s), 7.29 – 7.23 (2H, m), 7.20 – 7.12 (3H, m), 6.44 (1H, s), 2.55 (2H, t, J = 7.8 Hz), 2.49 – 2.46 (2H, m), 2.39 (3H, s), 1.55 (2H, p, J = 7.4 Hz), 1.42 (2H, p, J = 6.9 Hz), 1.30 (4H, dq, J = 9.4, 4.1 Hz); ^{13}C NMR (DMSO-d_6, 151 MHz) δ 203.4, 154.4, 154.0, 142.3, 138.6, 128.2, 128.2, 125.6, 122.0, 111.2, 101.3, 35.1, 32.4, 30.9, 30.2, 29.4, 28.9, 28.2; HRESIMS *m/z* 347.1408 $[M+H]^+$ (calcd for $C_{20}H_{24}ClO_3^+$ 347.1408), *m/z* 345.1269 $[M-H]^-$ (calcd for $C_{20}H_{22}ClO_3^-$ 345.1263).

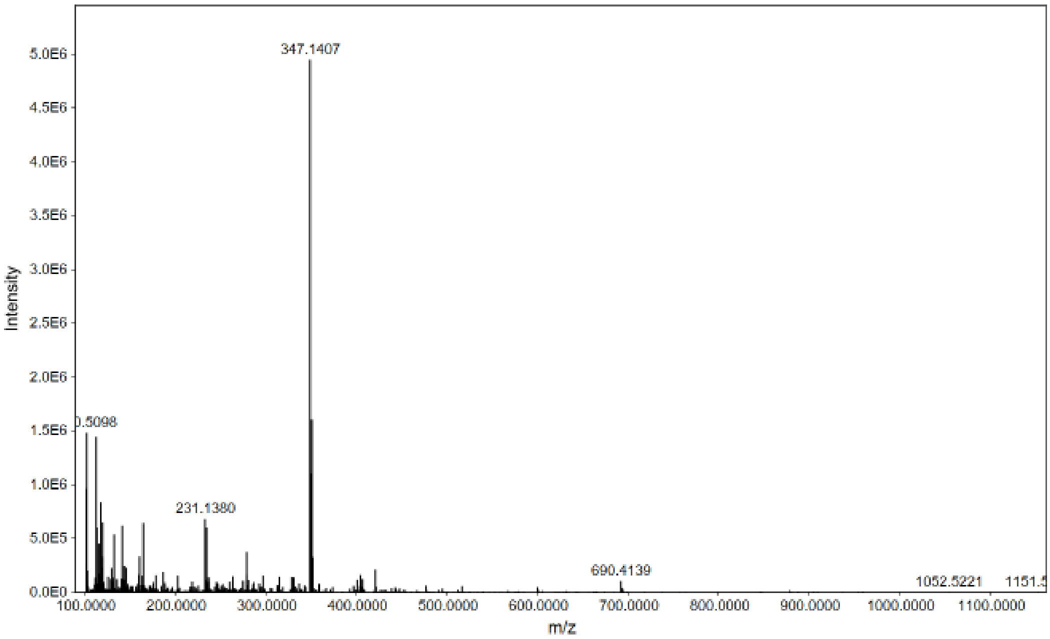

HRESIMS+ spectrum of **1-(3-chloro-4,6-dihydroxy-2-(6-phenylhexyl)phenyl)ethan-1-one** in MeOH.

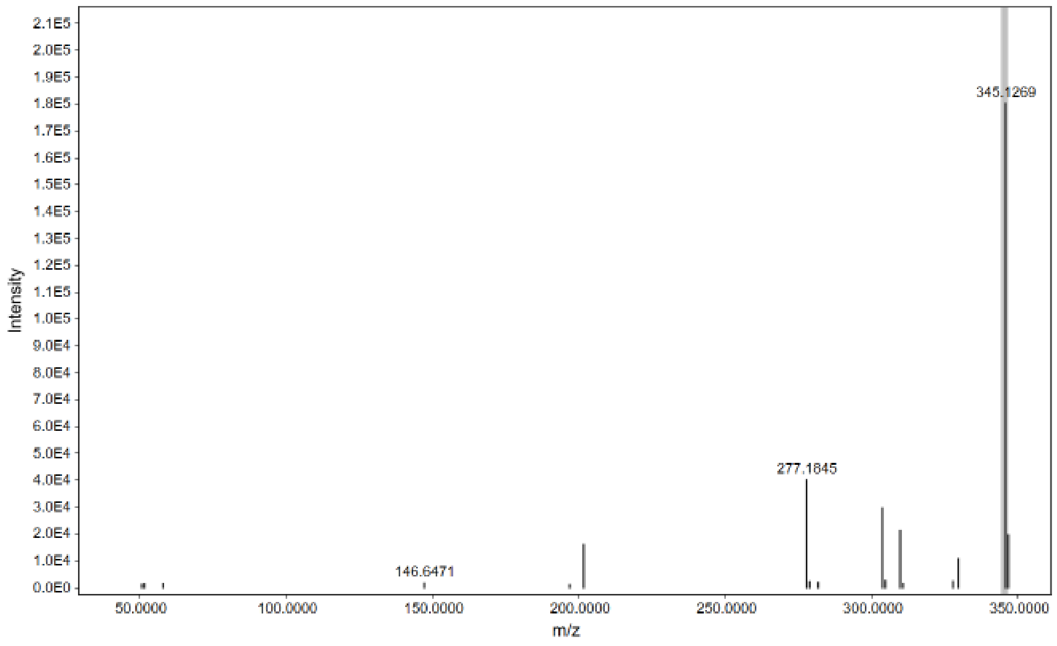

HRESIMS- spectrum of **1-(3-chloro-4,6-dihydroxy-2-(6-phenylhexyl)phenyl)ethan-1-one** in MeOH.

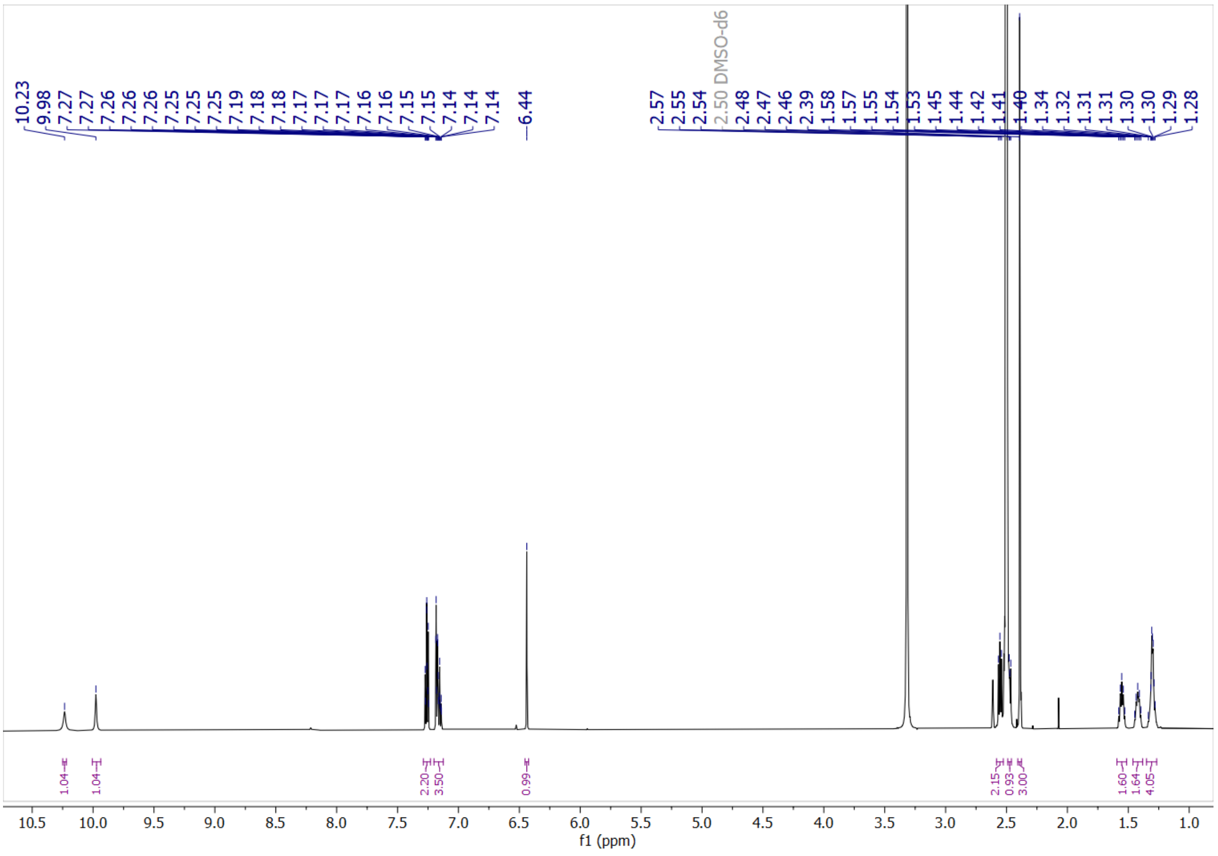

^{1}H NMR spectrum of **1-(3-chloro-4,6-dihydroxy-2-(6-phenylhexyl)phenyl)ethan-1-one** in DMSO-d_6 at 600 MHz.

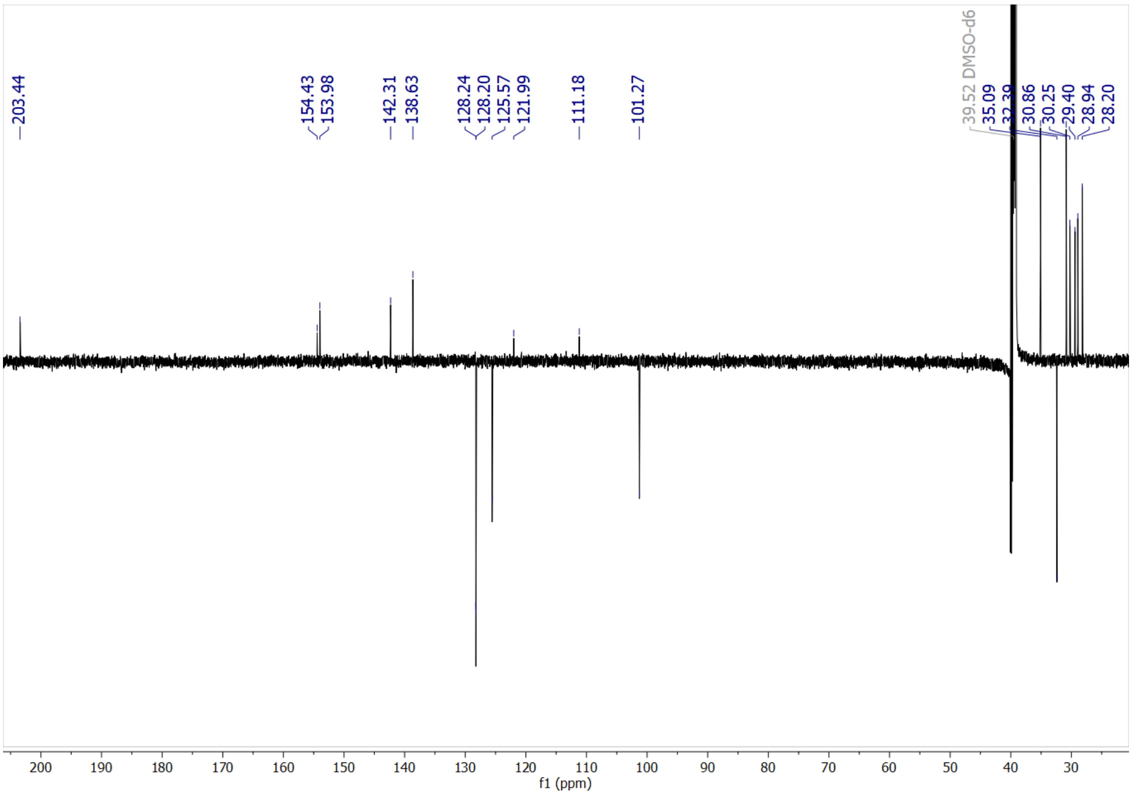

^{13}C DEPTQ NMR spectrum of **1-(3-chloro-4,6-dihydroxy-2-(6-phenylhexyl)phenyl)ethan-1-one** in DMSO-d_6 at 151 MHz.

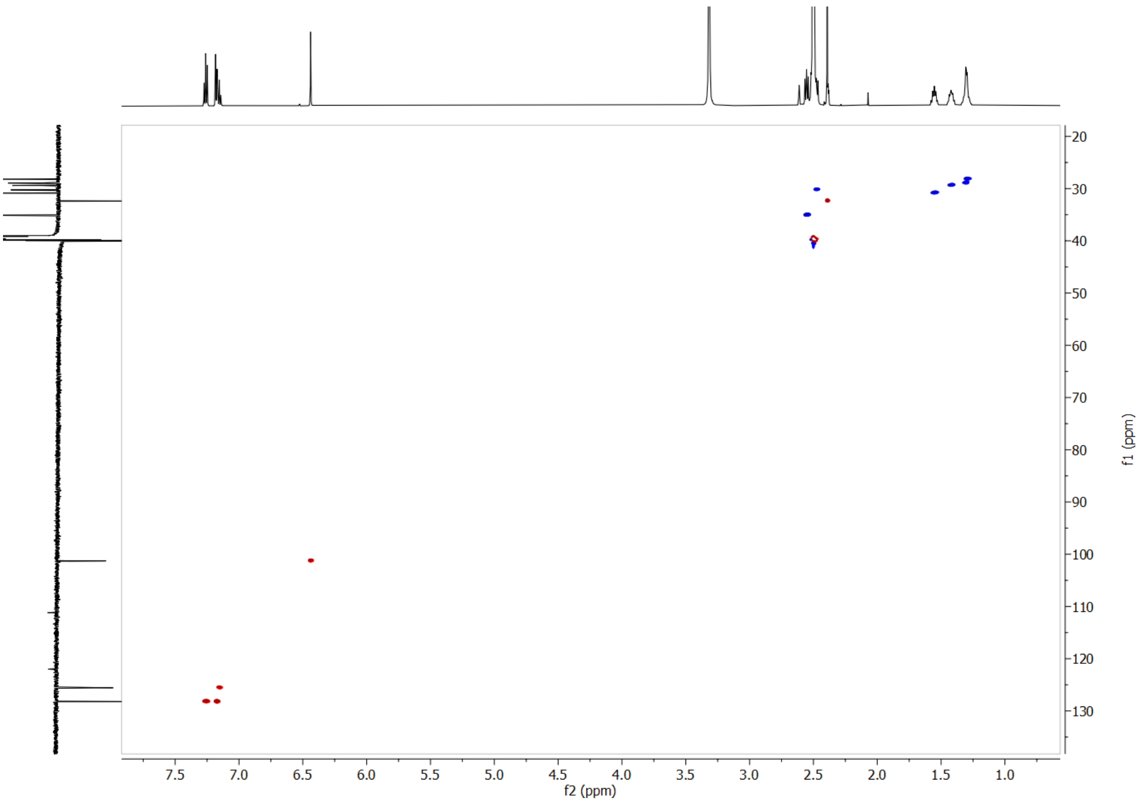

HSQC NMR spectrum of **1-(3-chloro-4,6-dihydroxy-2-(6-phenylhexyl)phenyl)ethan-1-one** in DMSO-d_6 at 600 MHz.

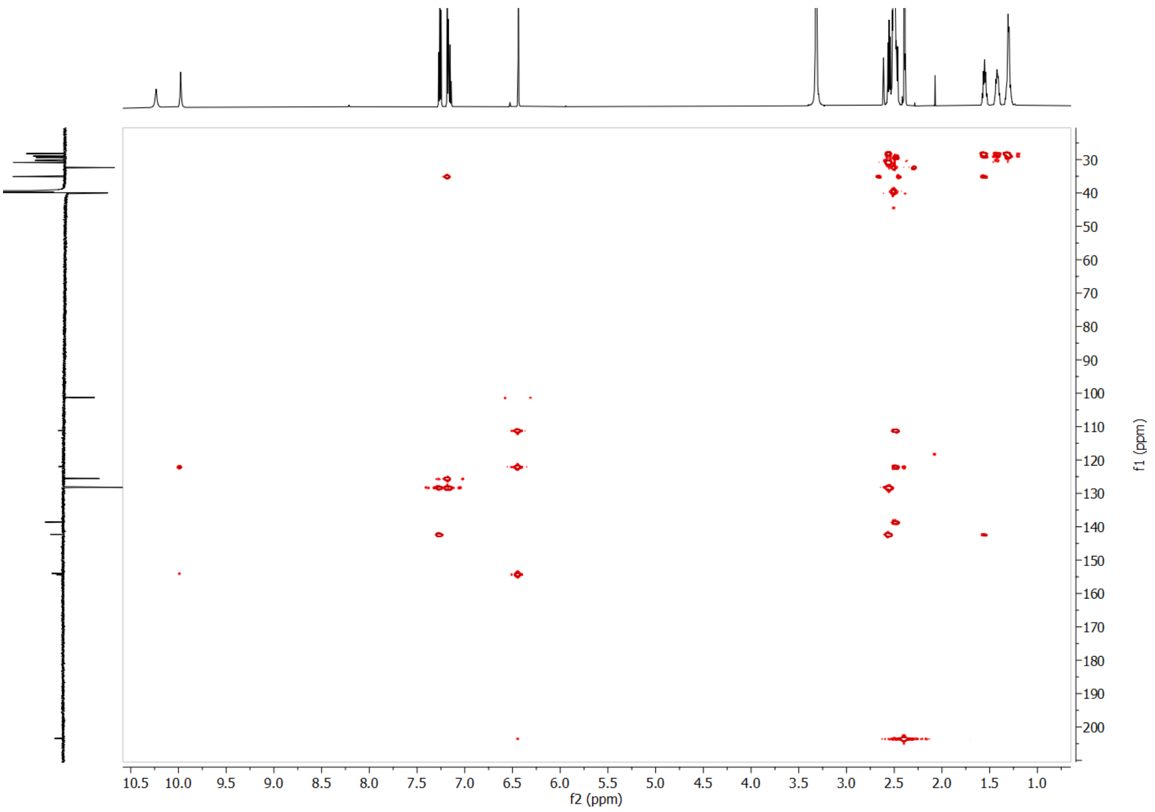

HMBC NMR spectrum of **1-(3-chloro-4,6-dihydroxy-2-(6-phenylhexyl)phenyl)ethan-1-one** in DMSO-d_6 at 600 MHz.

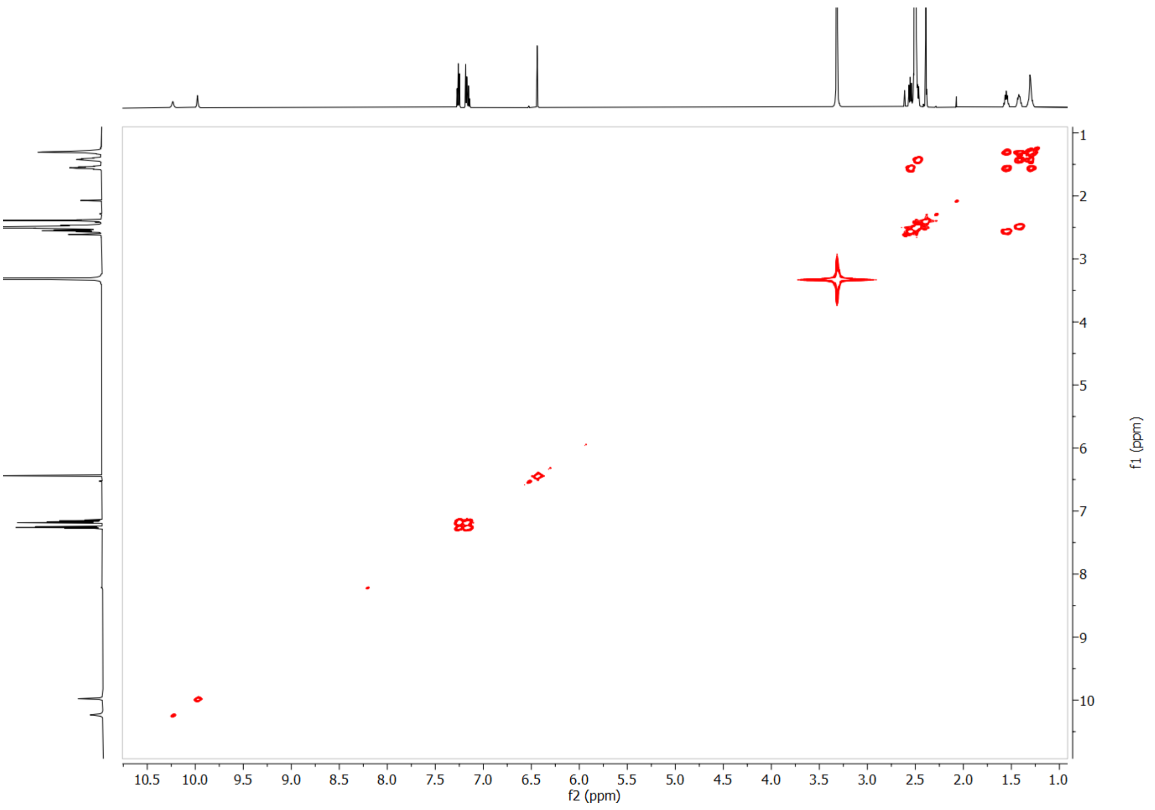

COSY NMR spectrum of **1-(3-chloro-4,6-dihydroxy-2-(6-phenylhexyl)phenyl)ethan-1-one** in DMSO-d_6 at 600 MHz.

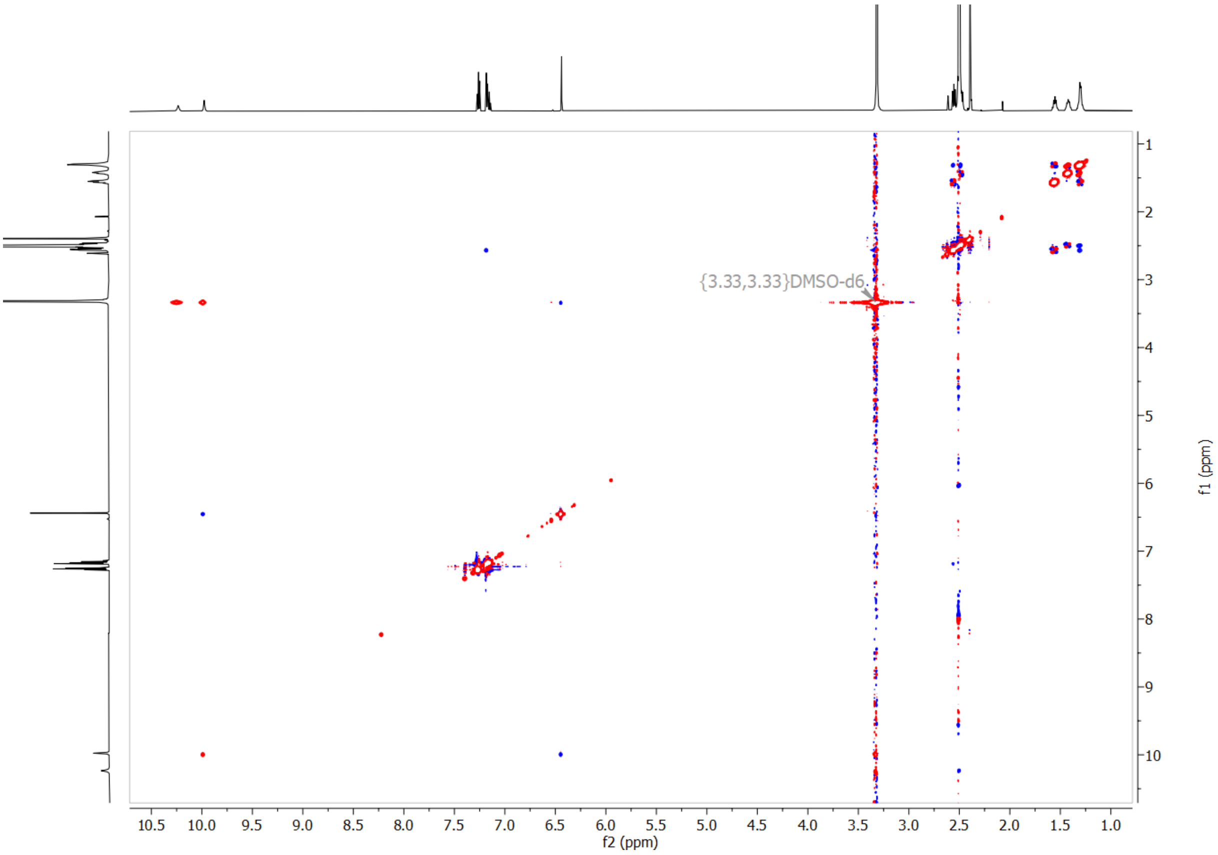

ROESY NMR spectrum of **1-(3-chloro-4,6-dihydroxy-2-(6-phenylhexyl)phenyl)ethan-1-one** in DMSO-d_6 at 600 MHz.

29. 1-(3,5-dichloro-2,4-dihydroxy-6-(6-phenylhexyl)phenyl)ethan-1-one:

Experimental:

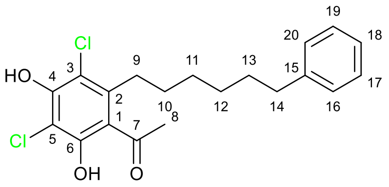

1-(3,5-dichloro-2,4-dihydroxy-6-(6-phenylhexyl)phenyl)ethan-1-one (**29**) ^{1}H NMR (DMSO-d_6, 600 MHz) δ 10.12 (1H, s), 8.13 (1H, s), 7.26 (2H, t, J = 7.6 Hz), 7.20 – 7.13 (3H, m), 2.56 (2H, t, J = 7.5 Hz), 2.52 (2H, t, J = 1.9 Hz), 2.43 (3H, s), 1.56 (2H, p, J = 7.5 Hz), 1.44 (2H, p, J = 7.8 Hz), 1.31 (4H, dq, J = 11.5, 5.6 Hz); ^{13}C NMR (DMSO-d_6,151 MHz) δ 201.9, 142.2, 142.2, 128.2, 128.0, 125.6, 35.0, 30.8, 28.5, 28.1, 28.1 (partial data, sample quantity too low for adequate ^{13}C NMR resolution); HRESIMS+/- no corresponding ion observed (calcd for $C_{20}H_{23}Cl_2O_3^+$ 381.1019), (calcd for $C_{20}H_{21}Cl_2O_3^-$ 379.0873).

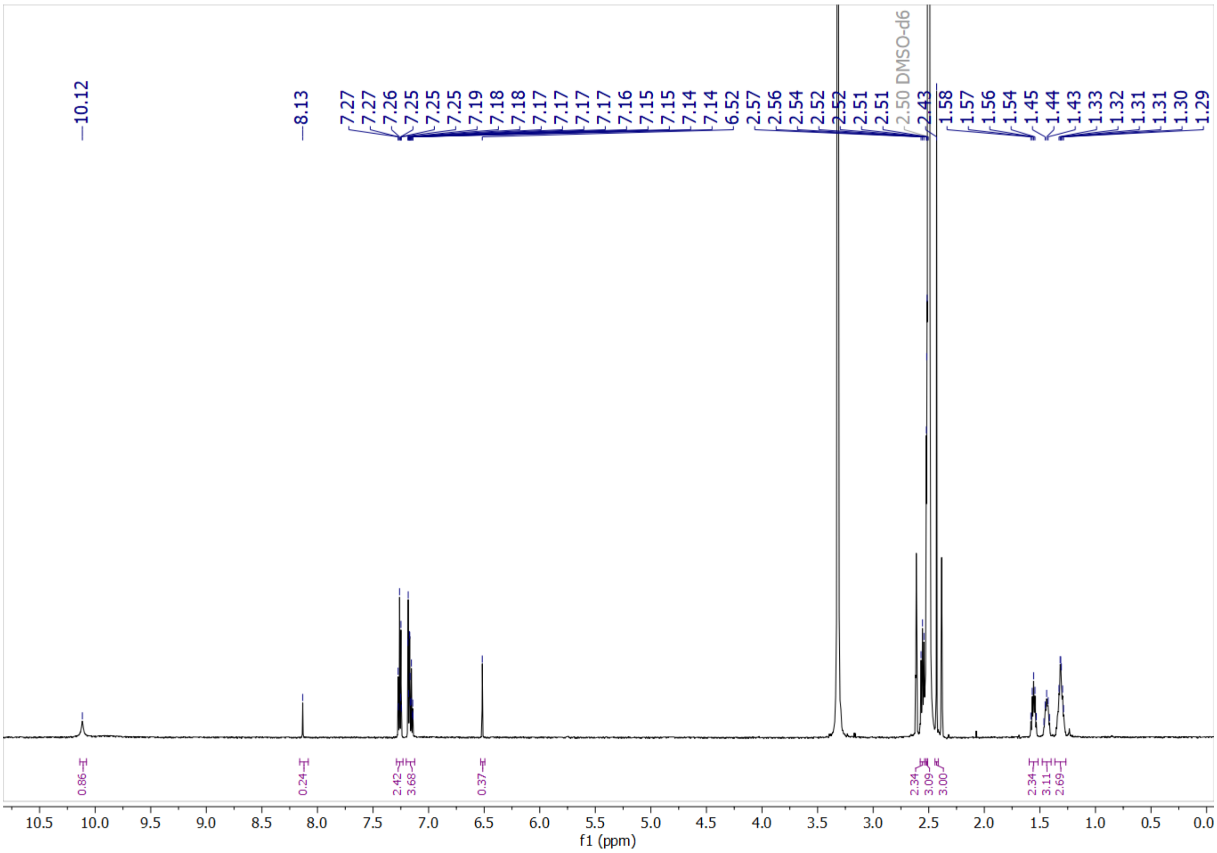

^{1}H NMR spectrum of **1-(3,5-dichloro-2,4-dihydroxy-6-(6-phenylhexyl)phenyl)ethan-1-one** in DMSO-d_6 at 600 MHz.

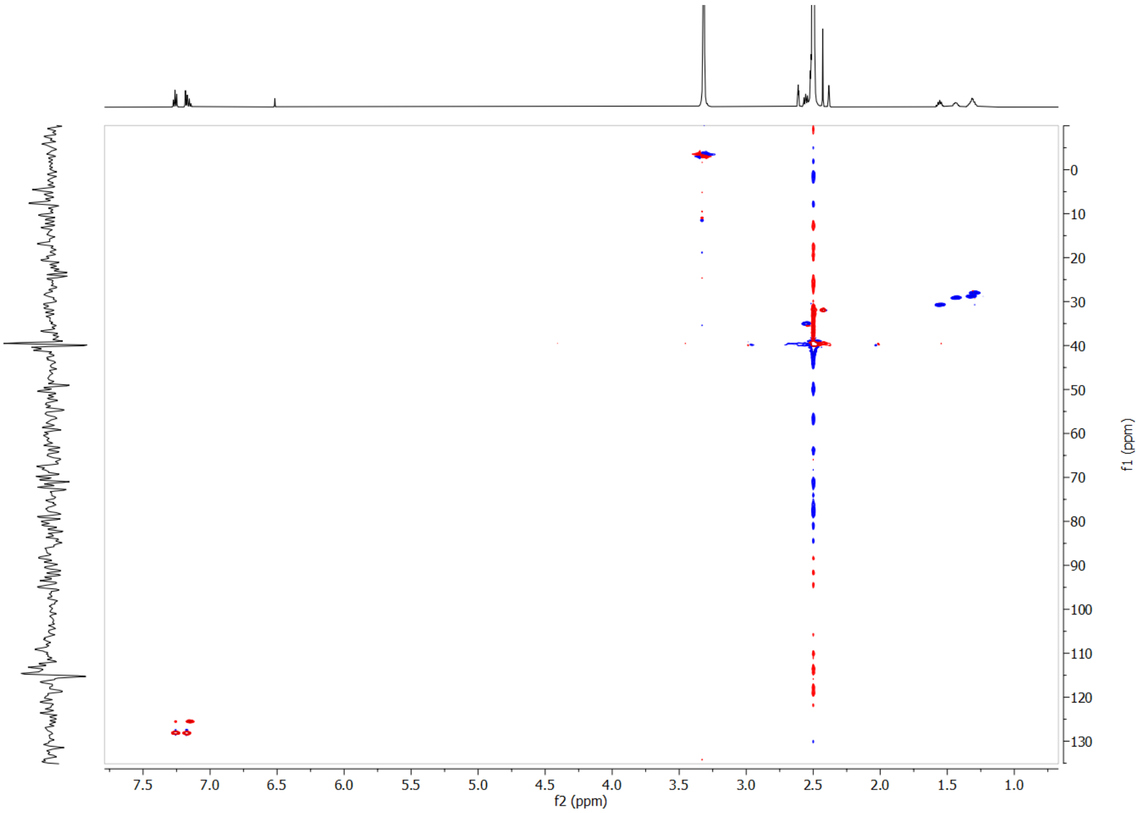

HSQC NMR spectrum of **1-(3,5-dichloro-2,4-dihydroxy-6-(6-phenylhexyl)phenyl)ethan-1-one** in DMSO-d_6 at 600 MHz.

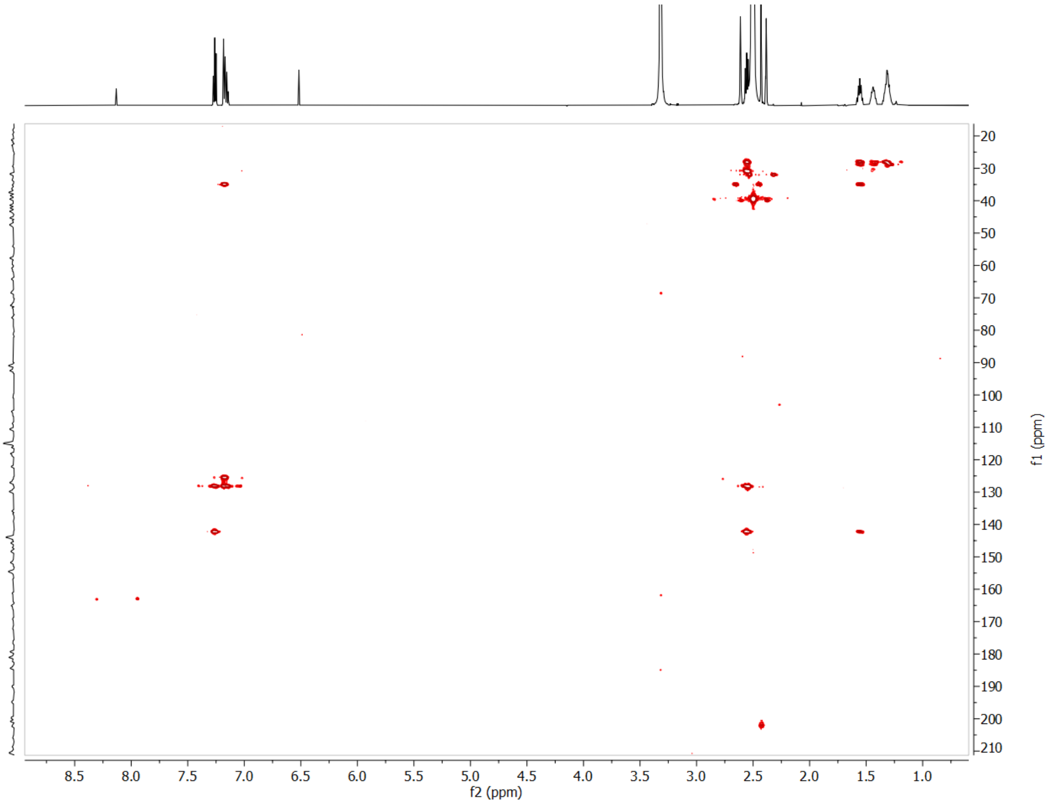

HMBC NMR spectrum of **1-(3,5-dichloro-2,4-dihydroxy-6-(6-phenylhexyl)phenyl)ethan-1-one** in DMSO-d_6 at 600 MHz.

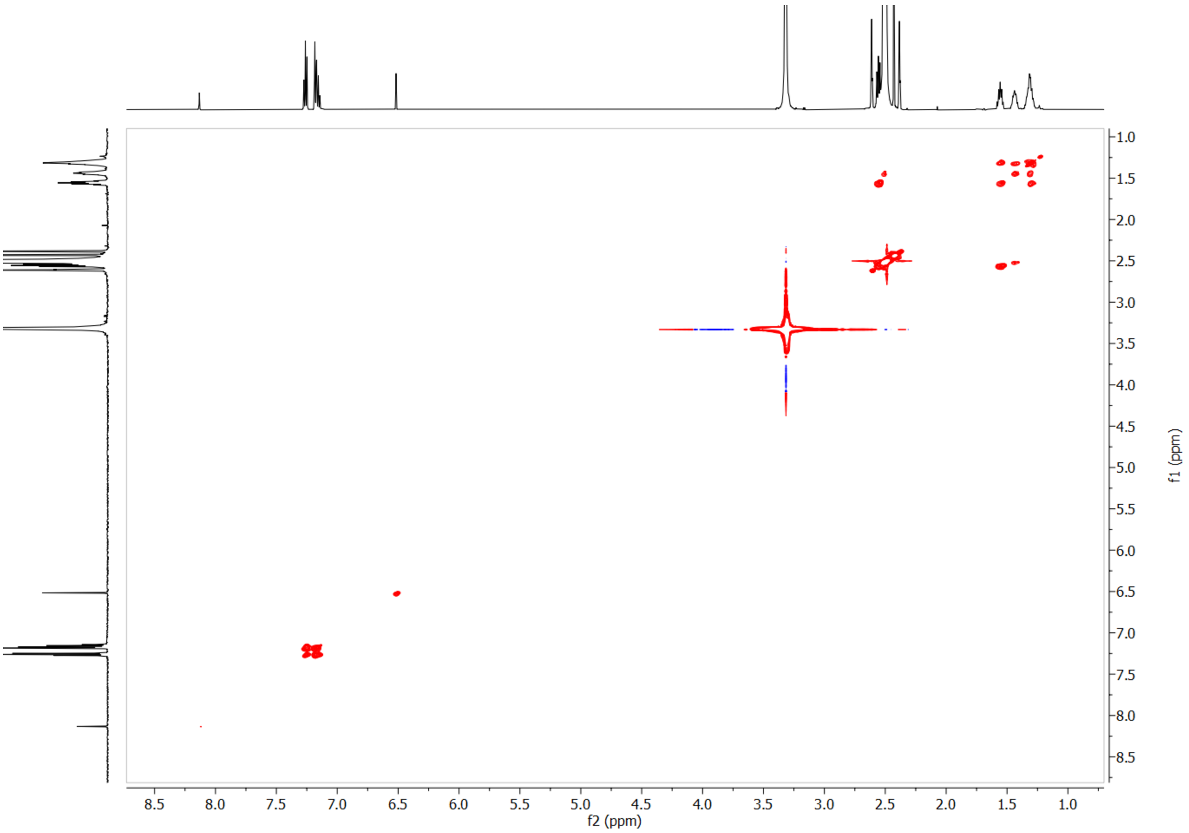

COSY NMR spectrum of **1-(3,5-dichloro-2,4-dihydroxy-6-(6-phenylhexyl)phenyl)ethan-1-one** in DMSO-d_6 at 600 MHz.

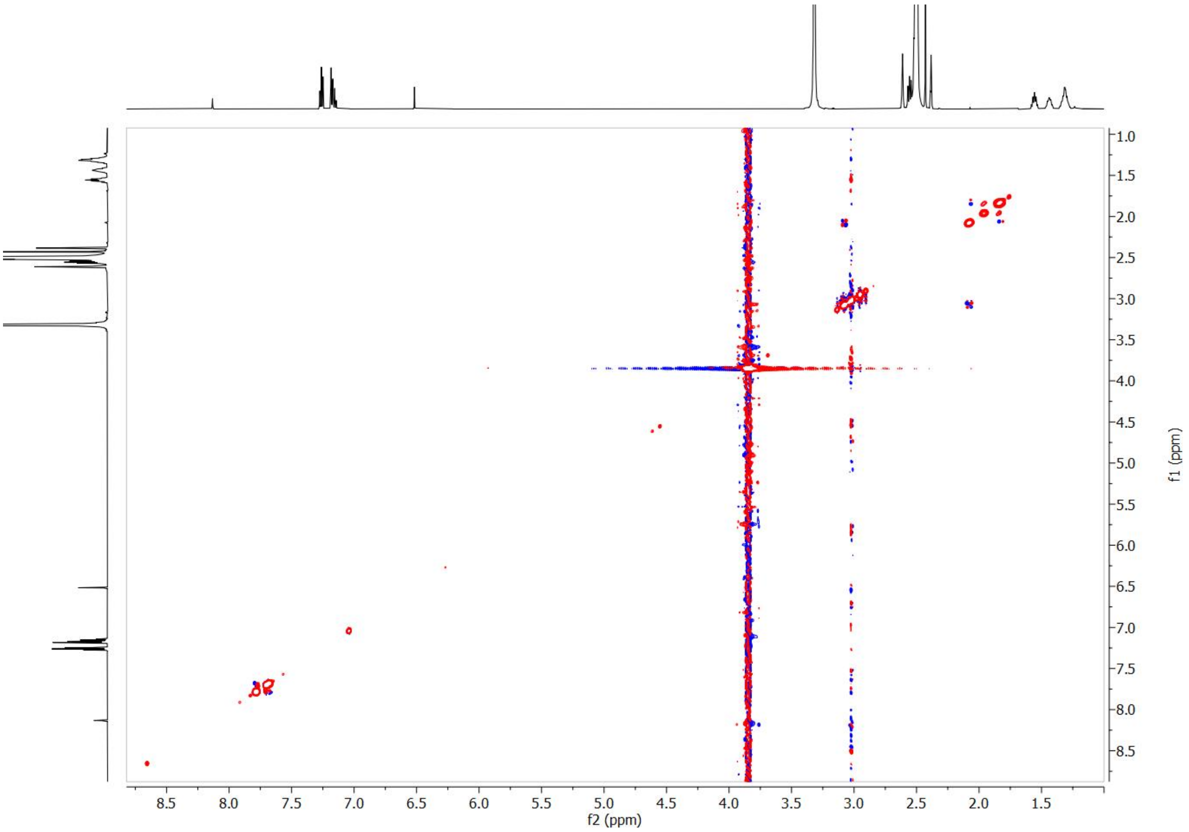

ROESY NMR spectrum of **1-(3,5-dichloro-2,4-dihydroxy-6-(6-phenylhexyl)phenyl)ethan-1-one** in DMSO-d_6 at 600 MHz.
